# Tethered Aryl Groups Increase the Activity of Anti-Proliferative Thieno[2,3-*b*]Pyridines by Targeting a Lipophilic Region in the Active Site of PI-PLC

**DOI:** 10.3390/pharmaceutics13122020

**Published:** 2021-11-26

**Authors:** Natalie A. Haverkate, Euphemia Leung, Lisa I. Pilkington, David Barker

**Affiliations:** 1 School of Chemical Sciences, University of Auckland, Auckland 1010, New Zealand; natalie.haverkate@auckland.ac.nz (N.A.H.); lisa.pilkington@auckland.ac.nz (L.I.P.); 2 Auckland Cancer Society Research Centre, University of Auckland, Auckland 1023, New Zealand; e.leung@auckland.ac.nz; 3 Department of Molecular Medicine and Pathology, University of Auckland, Auckland 1023, New Zealand; 4 The MacDiarmid Institute for Advanced Materials and Nanotechnology, Victoria University of Wellington, Wellington 6012, New Zealand

**Keywords:** thieno[2,3-*b*]pyridines, anti-proliferative, α,β-unsaturated ketones, heterocycles

## Abstract

The compounds 2-amino-3-carboxamido-thieno[2,3-*b*]pyridines have demonstrated excellent anti-proliferative activity against human cancer cell lines, including the triple-negative breast cancer cell line MDA-MB-231. In this study, 81 novel thieno[2,3-*b*]pyridines were synthesised in four series to further improve their anti-proliferative activity, in particular by targeting an adjacent lipophilic pocket in the putative target enzyme phosphoinositide phospholipase C (PI-PLC). Overall, it was found that appending a propyl-aryl group at C-5 on 2-amino-3-carboxamido-thieno[2,3-*b*]pyridine resulted in compounds with potent biological activity, exhibiting IC_50_ values in the nanomolar range. The propyl linker could be an α,β-unsaturated ketone or a saturated propyl ketone, but the highest activity was obtained when allylic alcohols were the tether between thieno[2,3-*b*]pyridine and the appended aryl group, with compound **21r** having IC_50_ values lower than 50 nM. Compounds with one extra carbon in the tether (i.e., a four-atom chain) were found to be considerably less active. Molecular modelling revealed this propyl tether places the newly introduced aryl ring in an untargeted lipophilic pocket within the active site of the phosphoinositide phospholipase C (PI-PLC) enzyme.

## 1. Introduction

The anti-proliferative thieno[2,3-*b*]pyridines have been extensively described in the literature, from their discovery in 2009 as potential inhibitors of PI-PLC [1] to the more recent analogue series that have exhibited increasingly potent anti-proliferative activity against triple-negative breast cancer cell lines, with IC_50_ values in the nM range, inducing excellent cell growth inhibition. These findings have increased the interest in these compounds for the development of anti-cancer drugs [2,3,4].

The mechanism of action by which thienopyridines exert their cytotoxic effect likely involves the inhibition of the phosphoinositide phospholipase C (PI-PLC) enzyme. This enzyme is implicated in many cellular pathways that result in cell proliferation, and its expression is known to be upregulated in many cancers, including the aforementioned triple-negative cancer cell lines [5]. The effects of thienopyridines on cell growth and morphology include severe growth restriction and rounding and blebbing of the plasma membrane. This was corroborated by biological studies that showed that the knockdown of PLC-δ_1_ and δ_3_ isoforms in MDA-MB-231 cells had similar effects on cell morphology [6].

Recent studies have shown that thienopyridines interact with a variety of other enzymes, including tyrosyl-DNA phosphodiesterase I (TDP1), A_2A_ receptor antagonists (A_2A_AR), G-protein coupled receptors (GPCRs), P2Y_12_ receptors, copper trafficking proteins Atox or tubulin [2,7,8,9,10,11,12,13]. Therefore, the anti-proliferative activity of thienopyridines cannot be solely attributed to PI-PLC inhibition, though PI-PLC has been validated as a legitimate target [6].

Other recent studies have also shown that thienopyridine-containing compounds with varying structures have a variety of other biological activities. These include moderate anti-bacterial activity [14], anti-fungal activity [15], and anti-proliferative activity towards multidrug-resistant leukaemia [16]. Therefore, thienopyridines are useful and highly modifiable heterocyclics for pharmaceutical design.

Studies have been undertaken over the past decade to provide an understanding of the effects of features on the thieno[2,3-*b*]pyridine peripheral structure, enhancing and refining the functional groups that are known to contribute to the biological effects and establishing reliable SAR.

**Figure 1:**
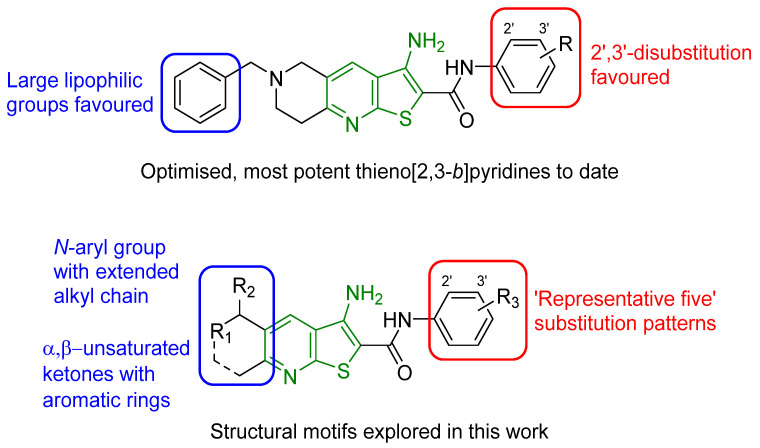
Structure of an *N*-benzyl thieno[2,3-*b*]naphthyridine with the general SAR and thienopyridine motif coloured in green (**top**) and strategies explored in this work to improve the activity of thieno[2,3-*b*]pyridines and probe the size limits of the PI-PLC enzyme (**bottom**).

Recently published, the most potent anti-proliferative thieno[2,3-*b*]pyridines reported are those compounds containing an *N*-benzylpiperidine ring fused to thieno[2,3-*b*]pyridine (Figure 1, blue) and 2′-Me-3′-Cl and 1′-naphthyl substitution patterns on the arylcarboxamide ring (Figure 1, red). These analogues demonstrated excellent IC_50_ values between 162 and 644 nM in HCT-116 and MDA-MB-231 cancer cells. This is consistent with the established pattern that 2′,3′-disubstituted aryl carboxamide-containing thienopyridines have the highest anti-proliferative activity among all the studied derivatives and that aromatic rings appropriately positioned off the pyridine ring favourably impact the anti-proliferative activity (Figure 1) [17].

The discovery that large lipophilic groups appended to the pyridine ring of the thienopyridine enhanced the molecule’s biological activity opened up a new area of exploration [4,17]. Following on from this, it was of particular importance to identify the motifs that would maximise hydrophobic interactions between thienopyridines and the PI-PLC active site, thereby providing enhanced biological potency. The targeted analogues included those exhibiting variations in the appended lipophilic groups as well as in the length and nature of the tether between these groups and the thienopyridine.

Herein, we report the synthesis, molecular modelling, and anti-proliferative activity of a comprehensive set of thienopyridine derivatives that fulfil the aforementioned proposed modifications (Figure 1), expanding on the potential effects of lipophilic groups appended to the pyridine ring.

## 2. Materials and Methods

### 2.1. Synthesis of the Compounds

General Details: All reactions were carried out under a nitrogen atmosphere in dry, freshly distilled solvents unless otherwise noted. All NMR spectra were recorded on a Bruker Avance 400 MHz spectrometer at ambient temperature. Chemical shifts are reported relative to the solvent peak of DMSO (δ 2.50 for ^1^H and δ 39.5 for ^13^C). ^1^H NMR data are reported as position (δ), relative integral, multiplicity (s, singlet; d, doublet; t, triplet; q, quartet; dd, doublet of doublets; dt, doublet of triplets; tt, triplet of triplets; m, multiplet; br, broad peak; qd, quartet of doublets), coupling constant (*J*, Hz), and the assignment of the atom. ^13^C NMR data are reported as position (δ) and assignment of the atom. All NMR assignments were performed with HSQC and HMBC experiments. High-resolution mass spectroscopy (HRMS) was carried out by either chemical ionization (CI) or electrospray ionization (ESI) on a MicroTOF-Q mass spectrometer. Unless noted, chemical reagents were used as purchased. General procedures, synthetic experimental methods and full characterization data (including copies of NMR spectra for all synthesized final compounds) can be found in the Supporting Information.

### 2.2. Cell Proliferation Assay

The synthesised thieno[2,3-*b*]pyridine final products were measured for their anti-proliferative activity against triple-negative breast cancer MDA-MB-231 and colorectal cancer HCT-116 cells (purchased from the American Type Culture Collection) using the ^3^H-thymidine incorporation assay. In total, 3000 cells were seeded in each well of 96-well plates with varying concentrations of thieno[2,3-*b*]pyridines for three days. As described in detail previously by Leung et al. [7], the experiments were performed in triplicate with a minimum of two experimental repeats. We added 0.04 μCi of 3H-thymidine to each well 5 h prior to harvest, after which the cells were harvested onto glass fibre filters using an automated TomTec harvester. The filters were incubated with Betaplate Scint, and thymidine incorporation was determined with a Trilux/Betaplate counter. The effects of the inhibitors on the incorporation of 3H-thymidine into DNA were determined relative to the control samples, with the positive control being a previously known active compound (thieno[2,3-*b*]pyridine, DJ0081, compound **7n** in Ref 4), and the negative control being cells in a well with no inhibitor.

### 2.3. Molecular Modelling

The thieno[2,3-*b*]pyridines synthesised in this study were docked into the mammalian PI-PLC-δ_1_ crystal structure, which was obtained from the Protein Data Bank (PDB ID: 1DJX-04, from *Rattus norvegicus*). The software GOLD suite version 5.8.1 was used to prepare the crystal structure for docking, by the addition of hydrogen atoms and the removal of water molecules, and the co-crystallised ligand (D-myo-inositol-1,4,5-triphosphate, IP3). Basic amino acids were assumed to be protonated, and acidic amino acids deprotonated in order to closely resemble a cell’s in vivo environment. The coordinates of the binding pocket were located at the Ca^2+^ ion, i.e., x = 126.257, y = 38.394, z = 22.370, as stated in the literature, with a 10 Å radius. ChemDraw 3D 15.0 was used to build the thieno[2,3-*b*]pyridines and to perform energy minimisation (MM2) of all structures. For each ligand to be docked, 50 docking runs were allowed at 100% search efficiency. The scoring functions ChemPLP, GoldScore, ChemScore and ASP were implemented to validate the predicted binding modes and relative energies of the ligands using the GOLD suite version (CSD, Cambridge, UK) 5.8.1.

## 3. Results and Discussion

### 3.1. Synthesis of N-Phenylethyl Thieno[2,3-b]Naphthyridine Derivatives (Series 1)

The purpose of the first series investigated in this work was to explore the theoretical size limit of the thienopyridines and ascertain whether moving the lipophilic aromatic ring further from the thienopyridine core would result in diminished, similar or improved biological activity. This resulted in the proposed synthesis of novel *N*-phenylethyl thieno[2,3-*b*]naphthyridine compounds, which are longer than the *N*-Bn analogues by one carbon.

**Scheme 1:**
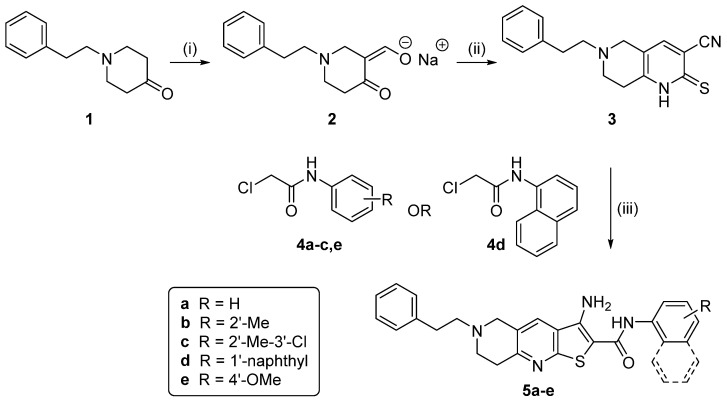
Synthesis of thieno[2,3-*b*]naphthyridines **5a–e**. Reagents and conditions: (i) 1-phenethylpiperidin-4-one **1** (1.0 equiv.), ethyl formate (1.0 equiv.), Na (1.0 equiv.), abs. EtOH (cat.), Et_2_O, r.t., 24 h, **2** 63%; (ii) salt **2** (1.0 equiv.), cyanothioacetamide (1.0 equiv.), piperidinium acetate soln. (cat.), H_2_O, reflux, 24 h, glacial AcOH (cat.), 24 h, **3** quant.; (iii) carbonitrile **3** (1.0 equiv.), 2-chloro-*N*-arylacetamides **4a–e** (1.0 equiv.), Na_2_CO_3_ (2 equiv.), abs. EtOH, reflux, 48 h, **5a–e** 25–71%.

The synthetic methods required to access these analogues proceeded in a manner analogous to that proposed in previous studies using established methods [3,4]. We converted 1-phenethylpiperidin-4-one **1** into enolate salt **2** using sodium ethoxide and ethyl formate, which was used in the following reaction, without further purification, with cyanothioacetamide in piperidinium acetate solution and provided the required carbonitrile **3**. Reaction of **3** with sodium carbonate in absolute ethanol, at reflux, with chloroacetamides **4a–e** [4] gave the *N*-phenylethyl thieno[2,3-*b*]naphthyridine derivatives **5a–e** (Scheme 1). Five selected chloroarylacetamides with substitution patterns R = H, 2′-Me, 2′-Me-3′-Cl, 1′-naphthyl and 4′-OMe (hereafter collectively referred to as the ‘representative five’) were chosen to perform these couplings, as they are typically associated with a range of activities and have previously been used by us to understand the interactions of the compounds with PI-PLC [17]. The 2′-Me-3′-Cl-phenyl and 1′-naphthyl substitution patterns typically impart the best anti-proliferative activity, 4′-OMe-phenyl the worst, whilst R = H (phenyl) and 2′-Me-phenyl moderate activity. It was considered that if new compounds displayed activities that did not match our previous results, this might indicate the compounds were interacting with off-target proteins.

The structure of all compounds was confirmed using ^1^H and ^13^C NMR spectra, infrared spectra, and high-resolution mass spectrometry. Full details and spectra can be found in the Supporting Information.

### 3.2. Anti-Proliferative Activity of Series 1

After the synthesis of series 1, the anti-proliferative activity against two cancer cell lines, MDA-MB-231 and HCT-116, were assessed (Table 1). These cell lines were used as they have previously been shown to be affected by antiproliferative thieno[2,3-*b*]pyridines [17,18,19,20]. Unfortunately, it was found that the antiproliferative activity of thienopyridines **5a–e**, overall, was poor. The most active analogue was 2′-Me-3′-Cl-phenyl derivative **5c**, which showed a maximum of 26.3% growth inhibition in MDA-MB-231 cells. This anti-proliferative activity was considerably lower than that of the previously tested 2′-Me-3′-Cl-phenyl substituted *N*-Bn thienopyridine, which showed up to 88.3% growth inhibition in the same cell line. Therefore, whilst this result fit the established SAR leading to the best anti-proliferative activity generally found for 2′-Me-3′-Cl-phenyl substituted thienopyridines, it showed that extending the distance from the thienopyridine to the phenyl ring from three atoms to four reduced the activity. Molecular modelling later suggested a reduced binding for these longer molecules (see below).

Because of the poor anti-proliferative activity of thienopyridines **5a–e**, *N*-phenylethyl compounds were not further investigated. Instead, we focused on series 2, which comprised previously unexplored α,β-unsaturated ketone-containing thienopyridines.

### 3.3. Synthesis of α,β-Unsaturated-Ketone Containing Thienopyridine Derivatives (Series 2)

**Figure 2:**
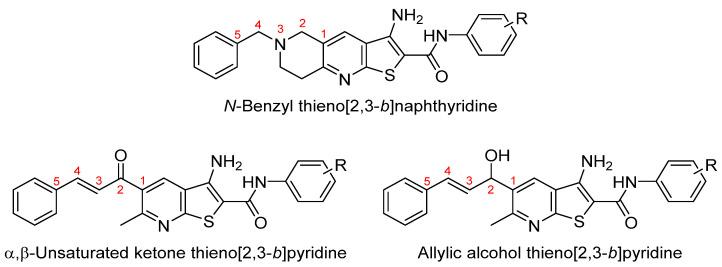
Size comparison between N-benzyl, targeted α,β-unsaturated, and allylic alcohol thieno[2,3-*b*]pyridines.

The next series to be explored, series 2, was chosen because of the similar atom length from the appended phenyl ring to the thieno[2,3-*b*]pyridine core in its compounds compared to the previously synthesised (and active) *N*-Bn thienonaphthyridines (Figure 2) [17]. It was thought that this would result in similarly biologically active compounds, as the cinnamoyl group could potentially fit into the same lipophilic pocket in the PI-PLC active site that was identified in previous molecular modelling studies [21]. It was also established through additional docking studies that the presence of a ketone or alcohol in the 2-position (Figure 2) in cyclic derivatives leads to hydrogen bonding interactions between the oxygen of the ketone and the amino acids ARG549 and LYS438 [18,22]. Therefore, the incorporation of both a carbonyl group in the 2-position and the aromatic ring of the cinnamoyl-type group appended to the pyridine ring was hypothesised to enhance the binding and therefore potentially increase the anti-proliferative activity.

It was hypothesised that thienopyridines fulfilling this criterion could be accessed by performing aldol condensations on the methyl ketone carbonitrile fragment, which could then be transformed into a variety of thieno[2,3-*b*]pyridine α,β-unsaturated ketone final products. The aforementioned ‘representative five’ substitution patterns were again chosen for the phenyl carboxamide ring. Benzaldehydes that contained polar heteroatoms, e.g., oxygen atoms that could be deprotected giving free phenols, were chosen, so as to potentially increase the solubility of these compounds.

**Scheme 2:**
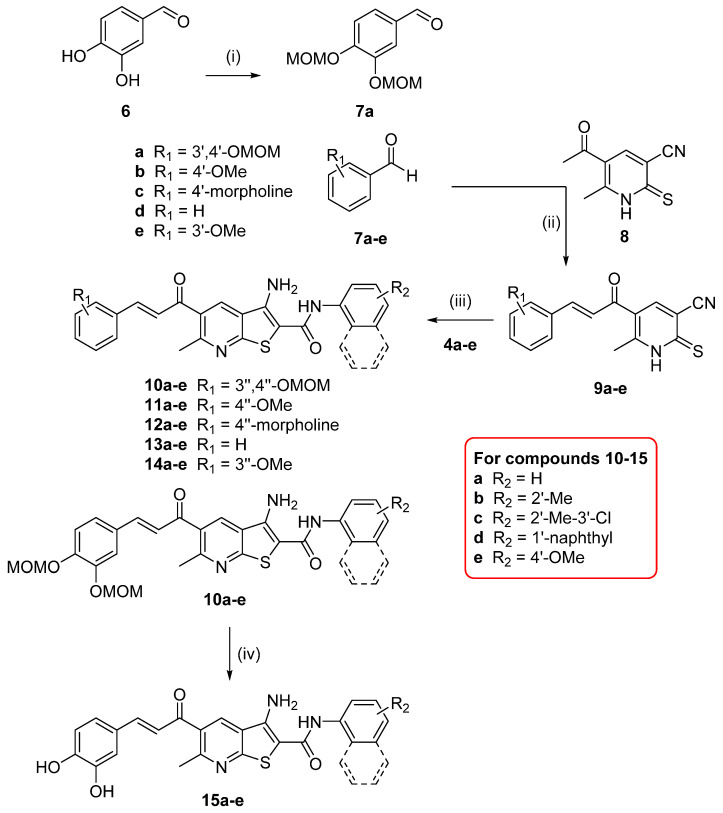
Synthesis of (*E*)-3-amino-5-(3-(4′’-methoxyphenyl)acryloyl)-6-methyl-*N*-phenylthieno[2,3-*b*]pyridine-2-carboxamides **10a–e**, **11a–e**, **12a–e**, **13a–e**, **14a–e** and **15a–e**. Reagents and conditions. (i) benzaldehyde **6** (1.0 equiv), MOMCl (2.6 equiv.), DIPEA (4.0 equiv), CH_2_Cl_2_, r.t., 24 h **7a** 83%; (ii) benzaldehydes **7a–e** (1.0 equiv.), carbonitrile **8** (1.0 equiv.), KOH (5.0 equiv.), EtOH/MeOH, r.t., 24 h; 2 M HCl **9a–e** 60%-quant; (iii) carbonitriles **9a–e** (1.0 equiv.), acetamides **4a–e** (1.0 equiv.), Na_2_CO_3_ (2 equiv.), EtOH, reflux, 48 h, **10a–e**, **11a–e**, **12a–e**, **13a–e** and **14a–e** 10%-quant.; (iv) **10a–e** (1.0 equiv.), 6 M HCl, MeOH, r.t., 24 h **15a–e** 38–60%.

The synthesis of these analogues began with MOM protection of benzaldehyde **6**, which proceeded under standard conditions, supplying di-MOM-ether benzaldehyde **7a**. Benzaldehydes **7a–e** were reacted in an aldol condensation with known carbonitrile **8** [17] in the presence of a strong base in a methanol/ethanol mixture at r.t. for 24 h to give the α,β-unsaturated ketone carbonitriles **9a–e** (Scheme 2).

Carbonitriles **9a–e** were then coupled with chloroarylacetamides **4a–e** in the aforementioned conditions, producing the desired thienopyridines **10a–e**, **11a–e**, **12a–e**, **13a–e** and **14a–e**. Additionally, the removal of the MOM ethers in **10a–e** using 6 M HCl in MeOH provided the catechols **15a–e**.

Following the successful preparation of thieno[2,3-*b*]pyridines **10a–e**, **11a–e**, **12a–e**, **13a–e**, **14a–e** and **15a–e**, a new set of compounds was proposed that would only incorporate the aforementioned 2′-Me-3′-Cl-phenyl substitution pattern, as these have been proven to consistently show improved anti-proliferative activity. This would enable the synthesis of a large novel series with variation on the new cinnamoyl group, whose activity could then be easily compared with the anti-proliferative activity of the thienopyridines discussed above.

**Scheme 3:**
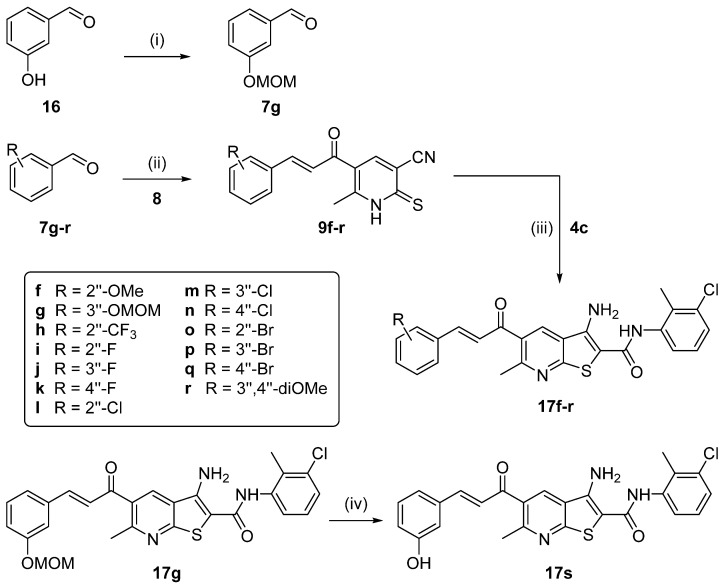
Synthesis of (*E*)-3-amino-*N*-(3′-chloro-2′-methylphenyl)-5-cinnamoyl-6-methylthieno[2,3-*b*]pyridine-2-carboxamides **17f–s**. Reagents and conditions: (i) benzaldehyde **16** (1.0 equiv), MOMCl (2.6 equiv.), DIPEA (4.0 equiv), CH_2_Cl_2_, r.t., 24 h **7g** 85%; (ii) benzaldehydes **7f–r** (1.0 equiv.), carbonitrile **8** (1.0 equiv.), KOH (5.0 equiv.), EtOH/MeOH, r.t., 24 h; 2 M HCl **9f–r** 42–63%; (iii) carbonitriles **9f–r** (1.0 equiv.), acetamide **4c** (1.0 equiv.), Na_2_CO_3_ (2 equiv.), EtOH, reflux, 48 h, **17f-r** 52–99%; (iv) **17g** (1.0 equiv.), 6 M HCl, MeOH, r.t., 24 h **17s** quant.

To this end, a range of additional carbonitriles **9f–r** was subsequently synthesised, incorporating halides and additional groups with various heteroatoms at different positions on the cinnamoyl ring (Scheme 3). The aldol condensations using selected benzaldehydes **7f–r** and carbonitrile **8** proceeded as described above to produce the compounds **9f–r**. Prior to its reaction with **8**, MOM-ether **7g** was synthesised from alcohol **16** with 85% yield, employing the aforementioned conditions.

The subsequent synthesis of the coupled thienopyridines **17f–r** similarly proceeded by heating at reflux in ethanol, in the presence of Na_2_CO_3_. Compound **17s** was then obtained from acid deprotection of the MOM ethers to complete series 2.

### 3.4. Anti-Proliferative Activity of Series 2

The anti-proliferative activity of series 2 compounds was then assessed, and IC_50_ values were calculated for the most active compounds. Since the incorporation of the cinnamoyl group was a novel modification for thienopyridines, it was unknown what substitution patterns were favoured on the cinnamoyl ring and if the α,β-unsaturated ketone linker would be tolerated.

Pleasingly, it was found that many of the compounds exhibited excellent anti-proliferative activity (Table 2). The results followed the known trend that 2′,3′-disubstitution patterns on the phenyl carboxamide ring result in higher efficacy, as compounds **10c**, **11c**, **11d**, **12c**, **12d**, **13c**, **13d**, **14c**, **14d**, **15c**, **15d** and the majority of **17** demonstrated excellent anti-proliferative activity (up to 99.4% cell growth inhibition when cells were treated with compounds at the concentration of 1 μM). In particular, compound **11d** exhibited an IC_50_ value of 79 nM in HCT-116 cancer cells, which is an improvement with respect to the IC_50_ of the previously reported most active known thieno[2,3-*b*]pyridines [4,17].

It was discovered that the phenyl ring in the cinnamoyl group favoured *ortho*- and *para*-substitutions (e.g., **17f,h,k,l,o,n**), with *meta*-substitution (e.g., **13c–d, 14c–d, 17j,s**) resulting in more potent activity if smaller groups were utilised.

These results indicated that the hypothesis regarding the effect of a similar distance between the appended phenyl ring and the thienopyridine in series 2 compounds to that in the biologically active *N*-Bn thienopyridines was correct.

### 3.5. Synthesis of Saturated Ketone-Containing Thienopyridine Derivatives (Series 3)

The excellent anti-proliferative results obtained for the series 2 α,β-unsaturated ketones demonstrated that they were worth investigating further. However, it was noted that α,β-unsaturated ketones represent a specific moiety (enones) that makes them Pan Assay Interference Compounds (PAINs), having the ability to interact covalently with a variety of targets, including various off-target proteins. That being said, there are FDA-approved drugs currently on the market that incorporate this moiety as a key part of their structure [23]. To expand further on this series and ascertain whether their improved biological activity was due to the enone moiety unselectively reacting with a range of proteins, alkene reduction of the α,β-unsaturated ketones to obtain saturated ketone products was proposed.

**Scheme 4:**
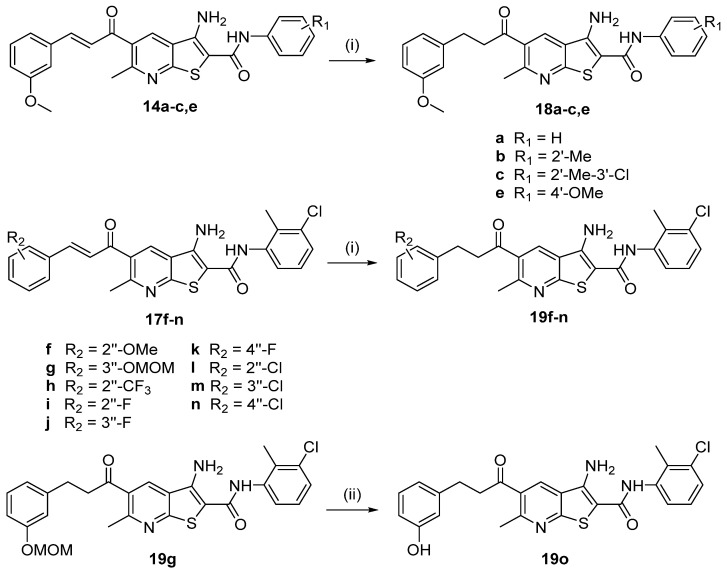
Synthesis of 3-amino-6-methyl-*N*-phenyl-5-(3-phenylpropanoyl)thieno[2,3-*b*]pyridine-2-carboxamides **18a–c,e** and **19f–o**. Reagents and conditions: (i) thieno[2,3-*b*]pyridines **14a–c,e** and **17f–n** (1.0 equiv.), 10% Pd/C (10–20% weight of SM), H_2_, MeOH/THF, 48–96 h, **18a–c, e** and **19f–n** 33%-quant; (ii) **19g** (1.0 equiv.), 6 M HCl, MeOH, r.t., 24 h, **19o** quant.

The reduction of enones **14a–c,e** and **17f–n** proceeded with catalytic hydrogenation using 10% Pd/C in THF/MeOH, to give the saturated ketones **18a–c,e** and **19f–o**, thus completing series 3 (Scheme 4). Attempts to perform catalytic hydrogenation on compounds **14d** and **17r** were unsuccessful. This transformation could also not be performed on bromide-containing analogues, as the conditions led to reductive debromination of the aromatic substituent.

### 3.6. Anti-Proliferative Activity of Series 3

The anti-proliferative activity of series 3 compounds was measured along with the IC_50_ values for the most active compounds. Pleasingly, as can be seen in Table 3, most series 3 analogues demonstrated anti-proliferative activity similar to that of their unsaturated series 2 counterparts. In particular, **18c** maintained the excellent anti-proliferative activity of its parent compound, **14c**, as **18c** showed 97.8% cell growth inhibition in HCT-116 cells (compared to 97.4% growth inhibition for **14c** when added at the concentration of 1 µM).

Compounds **19f–o** also showed comparable, if slightly decreased, anti-proliferative activity to that of compounds **17f–o**, demonstrating that the conversion of the α,β-unsaturated moiety to the saturated ketone did not negatively impact the anti-proliferative activity. This also demonstrated that any enhanced activity of series 2 compounds was most likely due to the size and/or shape of the molecule rather than to the introduction of an electrophilic enone group.

### 3.7. Synthesis of Allylic Alcohol Containing Thienopyridine Derivatives (Series 4)

**Scheme 5:**
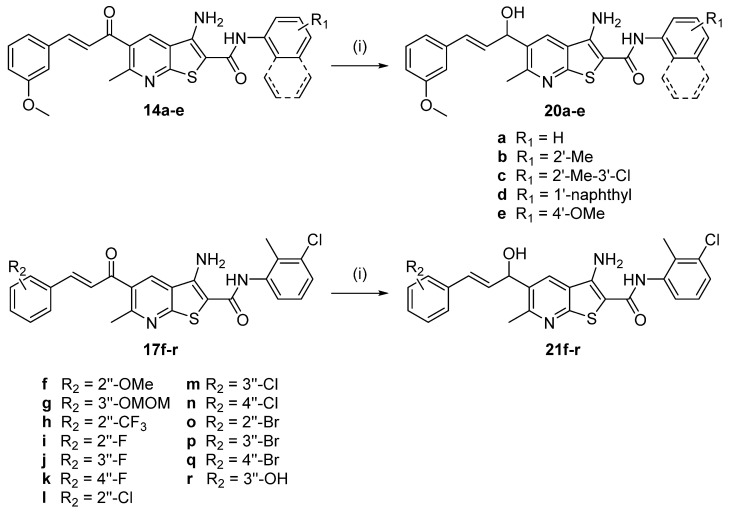
Synthesis of (*E*)-3-amino-5-(1-hydroxy-3-phenylallyl)-6-methyl-*N*-phenylthieno[2,3-*b*]pyridine-2-carboxamides **20a–e** and **21f–r**. Reagents and conditions: (i) thieno[2,3-*b*]pyridines **14a–e** and **17f–r** (1.0 equiv.), CeCl_3_ (1.1 equiv.), NaBH_4_ (1 equiv.), 2:1 THF/MeOH, 10–20 min, **20a–e** and **21f–r** 20%-quant.

With the knowledge that the enone group was not responsible for the increased bioactivity, we decided to perform the reduction of the ketone as the next transformation. As previously mentioned, alcohols at this position previously demonstrated good anti-proliferative activity and better solubility than thieno[2,3-*b*]pyridine derivatives that do not contain alcohols at this position [17,18,22]. In the first synthetic attempt, the required allylic alcohols **20a–e** were obtained by Luche reduction of enones **14a–e** (Scheme 5). CeCl_3_ was used as the Lewis acid, and the reactions were performed in 2:1 THF/MeOH mixtures using NaBH_4_ as the hydride reducing agent. The remaining series 2 compounds that used solely the 2′-Me-3′-Cl-phenyl substitution pattern on the phenyl carboxamide were then also subjected to the same conditions to produce allylic alcohols **21f–r** from α,β-unsaturated ketones **17f–r** and complete series 4.

### 3.8. Anti-Proliferative Activity of Series 4

The results if the anti-proliferative assays showed that the allylic alcohols in series 4 also demonstrated excellent anti-proliferative activity, with all compounds except **20e** (4′-OMe substituted) exhibiting at least 87.6% growth inhibition in both HCT-116 and MDA-MB-231 cells when added at 1 μM concentration (Table 4). In particular, compounds **21f,g,i,j,r** also exhibited IC_50_ values in the low nanomolar range, the lowest being that of **21r** (3″-OMe-substituted), which presented IC_50_ values of 25 nM and 51nM for HCT-116 and MDA-MB-231 cells, respectively.

Overall, series 4 compounds showed the best anti-proliferative activity out of all the series tested and appear to be among the best performing thieno[2,3-*b*]pyridines to date. The improved activity of the allylic alcohols with respect to the α,β-unsaturated ketone derivatives could be due to the decreased planarity of the compounds and/or to improved aqueous solubility due to the introduction of the more polar hydroxyl group. The cLogP values of the alcohols **20/21** were on average 0.7 lower than those of their ketone **14/17** counterparts, providing them with improved drug-like properties.

### 3.9. Molecular Modelling Study

The 81 thieno[2,3-*b*]pyridines synthesised in this study were also docked into the mammalian PI-PLC-δ_1_ crystal structure to further investigate the effect of new substitution patterns on the docking and visualise the results. Docking studies were performed using the four scoring functions in the GOLD software suite. Thienopyridines are known to form hydrogen bonds with amino acid residues in the PLC-δ_1_ active site, commonly including GLU341, HIS311, HIS356, ARG549 and LYS438, and many of these interactions were observed in this molecular modelling study.

**Figure 3:**
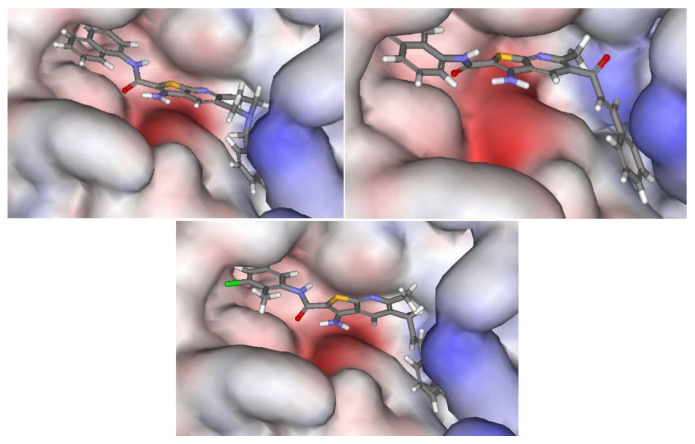
The docked configuration of a naphthyl-substituted *N*-Bn thienopyridine (**top left**), the novel enone **13d** (**top right**) and the novel allylic alcohol **21r** (**bottom centre**) in the binding site of PLC-δ_1_ using ChemPLP. The naphthyl group is docked in the upper left hydrophobic pocket, and the *N*-Bn/cinnamoyl ring in the bottom left hydrophobic pocket. Red areas depict a partial negative charge on the surface, blue areas depict a partial positive charge, and grey areas are neutral/lipophilic.

As can be seen in Figure 3, the docking conformation of the new α,β-unsaturated ketones was comparable to that of the previously synthesised *N*-Bn analogues, with the *N*-Bn ring and the cinnamoyl group consistently docked into the narrow hydrophobic cleft shown in the bottom right of the Figure. The similarity in binding suggests this is why these analogues have comparable activity.

Multiple new π and hydrogen bond interactions were also observed between the new cinnamoyl group, substitutions on the cinnamoyl ring (e.g., hydroxy groups) and amino acid residues of the PLC-δ_1_ active site (Figure 3). The scoring functions consistently gave high scores for the enones, and this was conserved between the algorithms, which was consistent with the excellent anti-proliferative activity of these compounds. The enone and enone derivatives were found to have mean ChemPLP scores between 82.44 and 90.04.

It was also consistently observed that *para*-substitution (4′-OMe) on the phenyl carboxamide rings led to a decrease in scoring across all the algorithms compared to the other ‘representative five’ substitution patterns, which was in line with previous findings. This was observed for all the docked derivatives that incorporated the 4′-OMe-phenyl substitution pattern. In docking studies, this was proposed to be due to the 4′-OMe group pushing the thieno[2,3-*b*]pyridines out of their normal central position in the active site, which subsequently results in the loss of important hydrophobic and hydrogen bonding interactions between the ligand and the active site.

**Figure 4:**
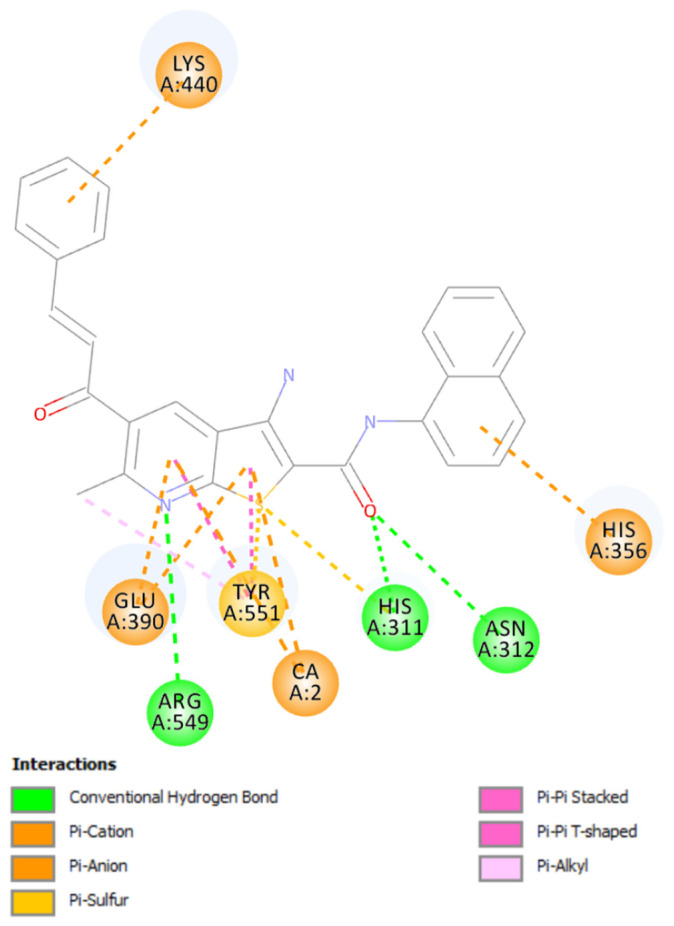
Observed interactions between **13d** and the PLC-δ_1_ active site (using ChemPLP algorithm in GOLD).

**Figure 5:**
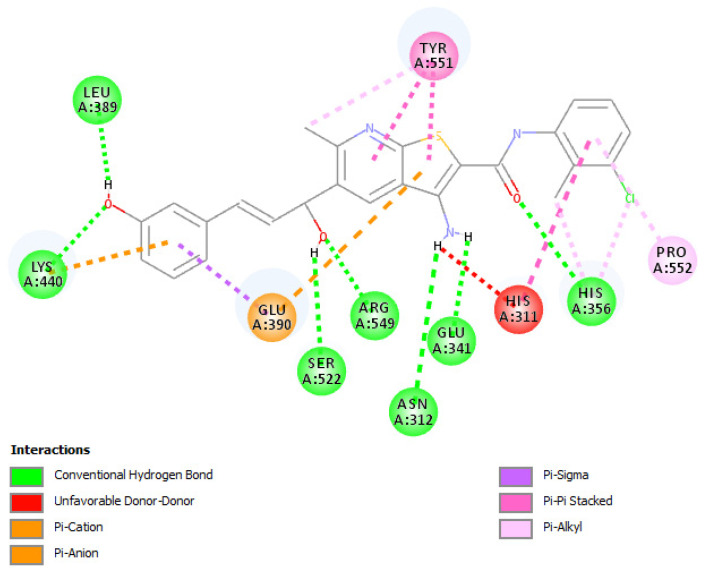
Observed interactions between **21r** and the PLC-δ_1_ active site (using ChemPLP algorithm in GOLD).

The observation of the interactions between the allylic alcohol **21r** (which had excellent anti-proliferative activity) and the PLC-δ_1_ active site (Figure 4 and Figure 5) showed that, although a unfavourable donor–donor interaction was observed, many π and hydrogen bond interactions were also present around the core structure. Notably, the 3″-OMe group on the cinnamoyl phenyl ring was involved in new hydrogen bond interactions with LEU389 and LYS440, and the allylic alcohol was involved in interactions with ARG549 and SER522, which were all highly favourable. These interactions were also apparent in the parent enone **17s**, though the enone carbonyl hydrogen bonded with different amino acids, which provides a possible explanation as to why enone **17s** also showed an excellent anti-proliferative activity. These additional interactions could also help to explain why this series of thieno[2,3-*b*]pyridine analogues, particularly the allylic alcohols, showed such good anti-proliferative activity overall.

## 4. Conclusions

This study focused on the preparation of novel thieno[2,3-*b*]pyridines with additional tethered aryl groups reaching into a lipophilic pocket within the PI-PLC active site. These new compounds were prepared to ascertain the size limit and improve the anti-proliferative activity of this class of compounds. In total, 81 compounds in four series were synthesised, tested for their anti-proliferative activity and studied using molecular modelling by docking them into the active site of PLC-δ_1_. It was found that the *N*-phenylethyl thieno[2,3-*b*]naphthyridines **5a–e** (series 1) demonstrated poor antiproliferative activity, which was hypothesised to be due to increased molecular length, which leads to poor binding with the target enzyme. This reduced binding was suggested by the disputed docking poses in the active site between the fitness functions. In contrast, many of the enones **10a–e**, **11a–e**, **12a–e**, **13a–e**, **14a–e** and **15a–e** (series 2), showed excellent anti-proliferative activity. Their derivatives, saturated ketones **18a–c,e** and **19f–o** (series 3) and allylic alcohols **20a–e** and **21f–r** (series 4), also demonstrated comparable activity and exhibited mean relative cell growth between 1.63 and 62.75%, surpassing in activity the thienopyridines previously considered as the most active. Overall, we found that the incorporation of any propyl-aryl group such as enones, saturated ketones or allylic alcohols increased the compounds’ anti-proliferative activity. These results show that the judicious placement of an aryl group in an adjacent lipophilic pocket in the active site of PI-PLC leads to an increase in thienopyridines’ overall activity.

## Figures and Tables

**Table 1:** Anti-proliferative activity (at 1 µM) of series 1 compounds in HCT-116 and MDA-MB-231 cancer cell lines; ^a^ see Section 2.3 for details of controls.

	Mean Relative Growth of 1 μM in Cancer Cell Line (%)	
	HCT-116	MDA-MB-231
**Positive control ^a^**	3.5	7.2
**Negative control**	100	100
**5a**	81.7	82.6
**5b**	91.2	85.4
**5c**	86.9	73.7
**5d**	88.9	99.0
**5e**	94.0	99.0

**Table 2:** Anti-proliferative activity (at 1 µM) of series 2 compounds in HCT-116 and MDA-MB-231 cancer cells; ^a^ see Section 2.3 for details of the controls.

	Mean Relative Growth of Cancer Cell Line in 1 μM Solution of Compound (%)		IC_50_ (nM)	
	HCT-116	MDA-MB-231	HCT-116	MDA-MB-231
**Positive control ^a^**	3.5	7.2		
**Negative control**	100	100		
**10a**	39.9	19.0	507	315
**10b**	74.2	46.4		
**10c**	2.0	2.3	391	403
**10d**	28.6	13.5	687	646
**10e**	78.8	41.4		
**11a**	46.0	45.4		
**11b**	65.8	76.7		
**11c**	3.0	5.8	154	182
**11d**	2.0	43.1	79	762
**11e**	99.2	96.3		
**12a**	7.7	19.2		
**12b**	3.7	18.6		
**12c**	1.4	5.3		
**12d**	2.5	6.9		
**12e**	82.5	51.5		
**13a**	40.3	40.4		
**13b**	78.7	68.3		
**13c**	1.6	2.6	461	436
**13d**	0.6	1.6	468	379
**13e**	51.0	53.2		
**14a**	43.7	39.0		
**14b**	63.1	66.0		
**14c**	2.6	8.6	489	477
**14d**	2.5	8.1	531	549
**14e**	60.7	60.2		
**15a**	0.6	2.6		
**15b**	22.8	10.7		
**15c**	0.7	1.7		
**15d**	0.9	4.4		
**15e**	9.1	5.2		
**17f**	1.9	3.9	242	270
**17g**	40.0	18.1		
**17h**	3.3	9.4	526	507
**17i**	2.2	4.1	165	189
**17j**	9.3	9.0	519	421
**17k**	4.3	4.4	394	236
**17l**	3.2	5.5	537	457
**17m**	37.2	25.5		
**17n**	7.4	10.2	515	345
**17o**	4.0	8.0	535	524
**17p**	33.5	15.9		
**17q**	21.3	19.0	525	466
**17r**	2.4	4.2	135	138
**17s**	1.4	2.0	137	103

**Table 3:** Anti-proliferative activity of series 3 compounds (1 µM concentration) in HCT-116 and MDA-MB-231 cancer cells; ^a^ see Section 2.3 for details of the controls.

	Mean Relative Growth of Cancer Cell Line in 1 μM Solution of Compound (%)		IC_50_ nM (if Present)	
	HCT-116	MDA-MB-231	HCT-116	MDA-MB-231
**Positive control ^a^**	3.5	7.2		
**Negative control**	100	100		
**18a**	83.2	58.6		
**18b**	91.7	87.5		
**18c**	2.2	12.3		
**18e**	73.9	80.5		
**19f**	10.5	12.8	968	949
**19g**	3.6	16.3		
**19h**	32.8	33.4		
**19i**	4.4	8.3	124	158
**19j**	2.7	14.5	506	564
**19k**	10.0	13.9	739	336
**19l**	5.6	13.1	393	431
**19m**	17.3	23.8	628	717
**19n**	46.2	31.6		
**19o**	1.2	6.1	442	518

**Table 4:** Anti-proliferative activity of series 4 compounds (1 µM concentration) in HCT-116 and MDA-MB-231 cancer cells; ^a^ see Section 2.3 for details of the controls.

	Mean Relative Growth of Cancer Cell Line in 1 μM Solution of Compound (%)		IC_50_ nM (if Present)	
	HCT-116	MDA-MB-231	HCT-116	MDA-MB-231
**Positive control ^a^**	3.5	7.2		
**Negative control**	100	100		
**20a**	5.6	4.8	407	440
**20b**	8.4	8.0	501	626
**20c**	3.5	3.5	228	341
**20d**	2.5	2.7	231	400
**20e**	92.2	84.1		
**21f**	1.6	3.9	55	92
**21g**	0.4	3.9	47	109
**21h**	1.6	4.2	128	141
**21i**	1.1	2.9	32	36
**21j**	1.8	4.5	50	104
**21k**	2.2	4.1	94	131
**21l**	1.6	3.7	136	140
**21m**	1.9	5.7	130	181
**21n**	2.2	4.9	137	168
**21o**	1.2	3.3	114	138
**21p**	3.0	12.4	156	223
**21q**	2.2	5.4	146	317
**21r**	0.4	3.5	25	51

## Data Availability

Data are contained within the article.

## Supplementary Materials

The experimental procedures and characterisation data for all synthesised compounds are available online at https://www.mdpi.com/article/10.3390/pharmaceutics13122020/s1.
