# Supplementary material for: Tethered Aryl Groups Increase the Activity of Anti-Proliferative Thieno[2,3-b]Pyridines by Targeting a Lipophilic Region in the Active Site of PI-PLC
Source: Pharmaceutics. 2021 Nov 26;13(12):2020. doi: 10.3390/pharmaceutics13122020 (PMC8705770; doi:10.3390/pharmaceutics13122020)
Supplement: Supplementary file 1 [file pharmaceutics-13-02020-s001.zip › pharmaceutics-1461944-supplementary.pdf]

Supporting Information for:

Tethered aryl groups increase activity of anti-proliferative thieno[2,3-*b*]pyridines by targeting a lipophilic region in the active site of PI-PLC

*Natalie A. Haverkate[1], Euphemia Leung[2], Lisa I. Pilkington[1], David Barker[1,3]**

[1]School of Chemical Sciences, University of Auckland, Auckland 1010, New Zealand

[2]Auckland Cancer Society Research Centre and Department of Molecular Medicine and Pathology, University of Auckland, Grafton, Auckland 1023, New Zealand

[3]The MacDiarmid Institute for Advanced Materials and Nanotechnology, Victoria University of Wellington, Wellington 6012, New Zealand

*Corresponding author. Tel.: +64-9-373-7599; e-mail: d.barker@auckland.ac.nz

Table of Contents:

General synthetic procedures S2

Synthetic procedures and compound characterisation data S3-S86

^{1}H and ^{13}C NMR spectra for compounds S87-S248

References S249

*General Procedure A: Synthesis of thieno[2,3-*b*]pyridine derivatives **5a-e, 10a-e, 11a-e, 12a-e, 13a-e** and **14a-e***

A mix of bicyclic carbonitrile (1 equiv.), 2-chlorophenylacetamide (1 equiv.), and anhydrous sodium carbonate (1.06-2 equiv.) in absolute ethanol (4.00 mL per mmol chloride) was heated at reflux for 24-72 h. The ethanol was then removed *in vacuo* and the remaining residue recrystallised from methanol to give the desired compound.

*General Procedure B: Synthesis of MOM-protected benzaldehydes 7**a** and 7**g***

To a substituted hydroxybenzaldehyde (1 equiv.) in dry CH_2Cl_2 (10 mL/g benzaldehyde) under an atmosphere of nitrogen was added DIPEA (4 equiv.) dropwise, then MOMCl (2.6 equiv.) and the reaction mixture was stirred at r.t. over 24 h. This was quenched with sat. NH_4Cl, extracted with CH_2Cl_2, washed with brine and dried with $MgSO_4$ to give the crude product. This was purified using flash chromatography (3:1 petroleum ether : ethyl acetate) to give the desired compound.

*General Procedure C: Synthesis of α,β-unsaturated ketone carbonitriles **9a-r***

To a mixture of carbonitrile **8** (1 equiv.) and substituted benzaldehyde (1 equiv.) in AR absolute ethanol was added anhydrous potassium hydroxide (5 equiv.) in dry methanol and left to stir at r.t. under an atmosphere of N_2 for 24 h. The resulting solid was filtered, washed with petroleum ether, and collected. The solid was dissolved in water, acidified with 2 M HCl and extracted with ethyl acetate, washed with brine and dried *in vacuo* to give the crude product. The residue was purified using flash chromatography to give the desired product.

*General Procedure D: Synthesis of thieno[2,3-*b*]pyridine alcohols **15a-e, 17s** and **19o***

To a solution of a MOM-protected thienopyridine (1 equiv.) in methanol (16 mL/mmol) was added 6 M HCl (16 mL/mmol) dropwise and stirred at r.t. for 24 h. The mixture was then diluted with water and extracted with ethyl acetate. The organic layer was washed with water and dried with $MgSO_4$ to give the crude product. This was then washed with dichloromethane to crystallise the solid out. The residue was purified using flash chromatography to give the desired product.

*General Procedure E: Synthesis of saturated ketones **18a-c,e** and **19f-o***

A solution of α,β-unsaturated ketone (1 equiv.) and 10% palladium on carbon (10-20% by weight of ketone) in dry methanol or tetrahydrofuran (20 mL/mmol) was stirred under an atmosphere of hydrogen for 48-96 h. The mixture was then filtered through Celite and washed with further methanol. The solvent was removed *in vacuo* to give the desired compound.

*General Procedure F: Synthesis of allylic alcohols **20a-e** and **21f-r***

To a stirred solution of α,β-unsaturated ketone (1 equiv.) in 2:1 THF:MeOH (55 mL/mmol ketone) was added cerium chloride (1.1 equiv.) under an atmosphere of N_2. The mixture was stirred until all the cerium chloride dissolved, then sodium borohydride (1.0 equiv.) was added in small portions over 10 min. This was left to stir at r.t. for 10 min, monitored by TLC, before quenching with 2 M HCl. The mixture was extracted with ethyl acetate and washed with sat. aq. $NaHCO_3$, brine, dried with $MgSO_4$, and the solvent removed *in vacuo* to give the crude product, which was purified using flash chromatography to give the desired product.

Sodium (*E*)- and (*Z*)-(4-oxo-1-phenethylpiperidin-3-ylidene)methanolate 2

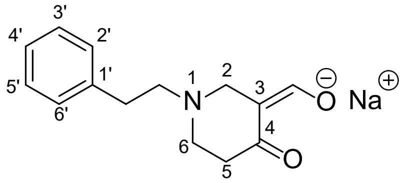

To a mix of sodium metal (0.23 g, 9.84 mmol) in dry diethyl ether (45.0 mL) under an atmosphere of N_2, was added dropwise a solution of 1-phenethylpiperidin-4-one **1** (2.00 g, 9.84 mmol) and ethyl formate (0.79 mL, 9.84 mmol) in diethyl ether (2.0 mL). A catalytic amount of absolute ethanol (0.05 mL) was then added as an initiator and the mixture was stirred at r.t. for 24 h. The resulting solid was filtered, washed with diethyl ether and dried *in vacuo* to give the *title compound* **2** (1.57 g, 63%) as a white solid. m.p. 168-170 °C. δ_H (400 MHz, D_2O) 2.38 (2H, t, J = 6.2 Hz, H-5), 2.75-2.82 (4H, m, H-6 and 1-CH_2), 2.88-2.92 (2H, m, 1'-CH_2), 3.31 (2H, s, H-2), 7.31 (1H, tt, J = 7.0, 1.6 Hz, H-4'), 7.35-7.42 (4H, m, H-2', H-3', H-5' and H-6'), 8.48 and 9.09 (1H, s, *E*/*Z* isomers, 3-CH). δ_C (100 MHz, D_2O) 32.6 (1'-CH_2), 35.1 (C-5), 49.4 and 49.5 (C-2 and C-6), 58.7 (1-CH_2), 109.4 (C-3), 126.4 (C-4'), 128.75 and 128.84 (C-2', C-3', C-5' and C-6'), 140.3 (C-1'), 180.8 (3-CH), 194.1 (C-4). ν_{max} (ATR)/cm^{-1} 3214 (C-H aromatic), 2925 (C-H alkane), 1620 (C=O carbonyl), 1595 (C=C aromatic), 1482 (-C-H bending), 1082 (C-N aliphatic). *m/z* (ESI$^-$): 230 (M$^-$, 100%), 113 (45%). HRMS (ESI$^-$) found (M$^-$): 230.1188 $C_{14}H_{16}NO_2^-$ requires 230.1187.

6-Phenethyl-2-thioxo-1,2,5,6,7,8-hexahydro-1,6-naphthyridine-3-carbonitrile 3

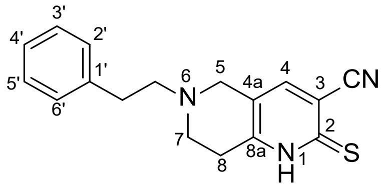

Firstly, a piperidinium acetate solution was prepared by combining acetic acid (20% by vol.), water (45%), and piperidine (35%). A mixture of sodium enolate **2** (1.20 g, 5.13 mmol), cyanothioacetamide (0.51 g, 5.13 mmol), and piperidinium acetate solution (0.45 mL) in water (5.0 mL) was heated at reflux for 24 h before being acidified with glacial acetic acid (0.70 mL). The reaction mixture was allowed to cool to r.t. and stirred for a further 12 h before the residue was filtered off, washed with ice water and collected to give the *title compound* **3** (1.51 g, quant.) as a brown solid. m.p. 192-194 °C. δ_H (400 MHz, $(CD_3)_2SO$) 2.67-2.79 (8H, m, H-7, H-8, 6-CH_2 and 1'-CH_2), 3.43 (2H, s, H-5), 7.15-7.29 (5H, m, 5 × Ar-CH), 7.86 (1H, s, H-4). NH not observed. δ_C (100 MHz, $(CD_3)_2SO$) 27.0 (C-8), 32.7 (1'-CH_2), 47.7 (C-7), 51.5 (C-5), 58.4 (6-CH_2), 113.4 (C-3), 117.1 (CN), 119.8 (C-4a), 125.9 (C-4'), 128.2 (C-3' and C-5'), 128.6 (C-2' and C-6'), 140.1 (C-1'), 143.4 (C-4), 150.9 (C-8a), 175.9 (C-2). ν_{max} (ATR)/cm^{-1} 3195 (N-H amine), 3025 (C-H aromatic), 2808 (C-H alkane), 2222 (C-N nitrile), 1595 (C=C aromatic), 1484 (-C-H bending), 1173 (C-N aliphatic). *m/z* (ESI^+): 318 (MNa^+, 100%), 269 (20%), 134 (85%). HRMS (ESI^+) found (MNa^+): 318.1031 $C_{17}H_{17}N_3NaS$ requires 318.1035.

3-Amino-6-phenethyl-*N*-phenyl-5,6,7,8-tetrahydrothieno[2,3-*b*][1,6]naphthyridine-2-carboxamide 5a

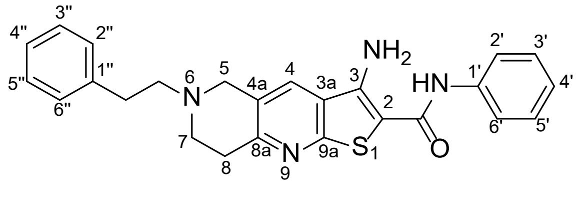

The reaction was carried out following General Procedure **A** using carbonitrile **3** (0.2 g, 0.68 mmol), chloride **4a** (0.11 g, 0.68 mmol) and anhydrous sodium carbonate (0.14 g, 1.35 mmol) in absolute ethanol (3.00 mL) for 48 h to give the *title compound* **5a** (0.12 g, 43%) as an orange solid. m.p. > 230 °C. δ_H (400 MHz, $(CD_3)_2SO$) 2.75-2.79 (2H, m, 6-CH_2), 2.85-2.90 (4H, m, H-7 and 1''-CH_2), 3.04 (2H, t, J = 5.8 Hz, H-8), 3.78 (2H, s, H-5), 7.06 (1H, t, J = 7.6 Hz, H-4'), 7.16-7.21 (1H, m, H-4''), 7.21-7.33 (8H, m, H-2'', H-3', H-3'', H-5', H-5'', H-6'' and NH_2),

7.70 (2H, dd, J = 7.6, 1.1 Hz, H-2' and H-6'), 8.18 (1H, s, H-4), 9.37 (1H, br s, NH). δ_C (100 MHz, $(CD_3)_2SO$) 32.3 (C-8), 33.0 (1''-CH_2), 49.8 (C-7), 54.6 (C-5), 58.9 (6-CH_2), 96.0 (C-2), 121.1 (C-2' and C-6'), 123.3 (C-4'), 124.2 (C-3a), 125.8 (C-4''), 126.4 (C-4a), 128.2 (C-3' and C-5'), 128.3 (C-3'' and C-5''), 128.6 (C-4), 128.7 (C-2'' and C-6''), 139.0 (C-1'), 140.3 (C-1''), 146.8 (C-3), 156.5 (C-8a), 156.8 (C-9a), 164.0 (2-CONH). ν_{max} (ATR)/cm^{-1} 3427 (N-H amide), 3215 (N-H amine), 3024 (C-H aromatic), 2823 (C-H alkane), 1595 (C=O amide), 1485 (C=C aromatic), 1453 (-C-H bending), 1283 (C-N aromatic), 1124 (C-N aliphatic). *m/z* (ESI^+): 429 (MH^+, 100%), 227 (10%), 101 (10%). HRMS (ESI^+) found (MH^+): 429.1736 $C_{25}H_{25}N_4OS$ requires 429.1744.

3-Amino-6-phenethyl-*N*-(*o*-tolyl)-5,6,7,8-tetrahydrothieno[2,3-*b*][1,6]naphthyridine-2-carboxamide 5b

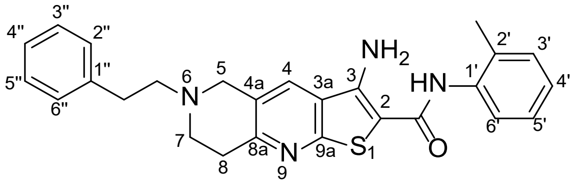

The reaction was carried out following General Procedure **A** using carbonitrile **3** (0.10 g, 0.34 mmol), chloride **4b** (74.0 mg, 0.34 mmol) and anhydrous sodium carbonate (72.0 mg, 0.68 mmol) in absolute ethanol (3.00 mL) for 48 h to give the *title compound* **5b** (56.0 mg, 37%) as a light brown solid. m.p. decomp. δ_H (400 MHz, $(CD_3)_2SO$) 2.22 (3H, s, 2'-CH_3), 2.75-2.79 (2H, m, 6-CH_2), 2.85-2.90 (4H, m, H-7 and 1''-CH_2), 3.03 (2H, t, J = 5.8 Hz, H-8), 3.78 (2H, s, H-5), 7.14-7.32 (11H, 9 × Ar-CH and NH_2), 8.16 (1H, s, H-4), 9.07 (1H, br s, NH). δ_C (100 MHz, $(CD_3)_2SO$) 17.9 (2'-CH_2), 32.3 (C-8), 33.0 (1''-CH_2), 49.9 (C-7), 54.6 (C-5), 58.9 (6-CH_2), 96.4 (C-2), 124.4 (C-3a), 125.8 (C-4''), 125.9 (C-4' and C-5'), 126.4 (C-4a), 126.8 (C-6'), 128.2 (C-3'' and C-5''), 128.6 (C-4), 128.7 (C-2'' and C-6''), 130.1 (C-3'), 133.9 (C-2'), 136.4 (C-1'), 140.3 (C-1''), 146.2 (C-3), 156.5 (C-8a), 156.6 (C-9a), 164.0 (2-CONH). ν_{max} (ATR)/cm^{-1} 3435 (N-H amide), 3326 (N-H amine), 3189 (C-H aromatic), 2951 (C-H alkane), 1600 (C=O amide), 1580 (C=C aromatic), 1452 (-C-H bending), 1244 (C-N aromatic), 1111 (C-N aliphatic). *m/z* (ESI^+): 443 (MH^+, 100%), 227 (10%), 101 (10%). HRMS (ESI^+) found (MH^+): 443.1897 $C_{26}H_{27}N_4OS$ requires 443.1900.

3-Amino-*N*-(3'-chloro-2'-methylphenyl)-6-phenethyl-5,6,7,8-tetrahydrothieno[2,3-*b*][1,6]naphthyridine-2-carboxamide 5c

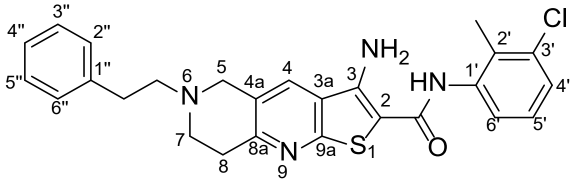

The reaction was carried out following General Procedure **A** using carbonitrile **3** (0.10 g, 0.34 mmol), chloride **4c** (74.0 mg, 0.34 mmol) and anhydrous sodium carbonate (72.0 mg, 0.68 mmol) in absolute ethanol (3.00 mL) for 48 h to give the *title compound* **5c** (0.11 g, 69%) as a mustard solid. m.p. > 230 °C. δ_H (400 MHz, $(CD_3)_2SO$) 2.23 (3H, s, 2'-CH_3), 2.77-2.79 (2H, m, 6-CH_2), 2.85-2.89 (4H, m, H-7 and 1"-CH_2), 3.03 (2H, t, J = 5.7 Hz, H-8), 3.78 (2H, s, H-5), 7.19-7.22 (4H, H-4", H-5' and NH_2), 7.28-7.29 (6H, m, H-2", H-3", H-4', H-5", H-6' and H-6"), 8.16 (1H, s, H-4), 9.31 (1H, br s, NH). δ_C (100 MHz, $(CD_3)_2SO$) 15.4 (2'-CH_2), 32.3 (C-8), 33.0 (1"-CH_2), 49.9 (C-7), 54.6 (C-5), 58.9 (6-CH_2), 124.4 (C-3a), 125.8 (C-4" and C-6'), 126.4 (C-4' and C-4a), 126.6 (C-5'), 128.2 (C-3" and C-5"), 128.6 (C-4), 128.7 (C-2" and C-6"), 132.2 (C-2'), 133.5 (C-3'), 138.7 (C-1'), 140.3 (C-1"), 146.1 (C-3), 156.6 (C-8a and C-9a), 164.2 (2-CONH). C-2 not observed. ν_{max} (ATR)/cm^{-1} 3433 (N-H amide), 3320 (N-H amine), 3028 (C-H aromatic), 2940 (C-H alkane), 1604 (C=O amide), 1573 (C=C aromatic), 1466 (-C-H bending), 1242 (C-N aromatic), 1112 (C-N aliphatic), 698 (C-Cl). *m/z* (ESI$^+$): 479 ($^{37}ClMH^+$, 40%), 477 ($^{35}ClMH^+$, 100%), 227 (10%). HRMS (ESI$^+$) found ($^{37}ClMH^+$): 479.1480 $C_{26}H_{26}{}^{37}ClN_4OS$ requires 479.1489. Found ($^{35}ClMH^+$): 477.1504 $C_{26}H_{26}{}^{35}ClN_4OS$ requires 477.1510.

3-Amino-*N*-(naphthalen-1'-yl)-6-phenethyl-5,6,7,8-tetrahydrothieno[2,3-*b*][1,6]naphthyridine-2-carboxamide 5d

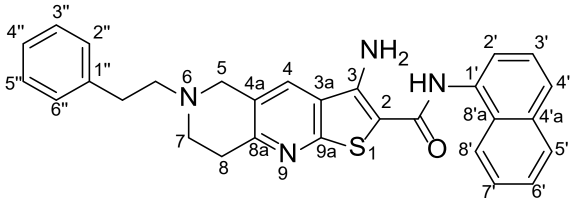

The reaction was carried out following General Procedure **A** using carbonitrile **3** (0.10 g, 0.34 mmol), chloride **4d** (74.0 mg, 0.34 mmol) and anhydrous sodium carbonate (72.0 mg, 0.68 mmol) in absolute ethanol (3.00 mL) for 48 h to give the *title compound* **5d** (40.0 mg, 25%) as

a brown solid. m.p. > 230 °C. δ_H (400 MHz, $(CD_3)_2SO$) 2.77-2.80 (2H, m, 6-CH_2), 2.86-2.92 (4H, m, H-7 and 1''-CH_2), 3.04-3.06 (2H, m, H-8), 3.80 (2H, s, H-5), 7.18-7.23 (3H, m, H-4'' and NH_2), 7.28-7.30 (4H, m, H-2'', H-3'', H-5'' and H-6''), 7.52-7.54 (4H, m, 4 × Ar-CH), 7.81-7.82 (1H, m, Ar-CH), 7.94-7.97 (2H, m, 2 × Ar-CH), 8.18 (1H, s, H-4), 9.66 (1H, br s, NH). δ_C (100 MHz, $(CD_3)_2SO$) 32.3 (C-8), 33.0 (1''-CH_2), 49.9 (C-7), 54.7 (C-5), 58.9 (6-CH_2), 123.5 (Ar-CH), 124.4 (Ar-CH), 124.5 (C-3a), 125.5 (Ar-CH), 125.6 (Ar-CH), 125.9 (C-4'' and Ar-CH), 126.3 (Ar-CH), 126.4 (C-4a), 128.0 (Ar-CH), 128.2 (C-3'' and C-5''), 128.6 (C-4), 128.7 (C-2'' and C-6''), 129.7 (Ar-C), 133.7 (C-1' and Ar-C), 140.4 (C-1''), 146.4 (C-3), 156.7 (C-8a and C-9a), 164.9 (2-CONH). C-2 not observed. ν_{max} (ATR)/cm^{-1} 3433 (N-H amide), 3322 (N-H amine), 3023 (C-H aromatic), 2934 (C-H alkane), 1603 (C=O amide), 1503 (C=C aromatic), 1466 (-C-H bending), 1262 (C-N aromatic), 1119 (C-N aliphatic). *m/z* (ESI^+): 479 (MH^+, 100%), 227 (25%), 101 (10%). HRMS (ESI^+) found (MH^+): 479.1889 $C_{29}H_{27}N_4OS$ requires 479.1900.

3-Amino-*N*-(4'-methoxyphenyl)-6-phenethyl-5,6,7,8-tetrahydrothieno[2,3-*b*][1,6]naphthyridine-2-carboxamide 5e

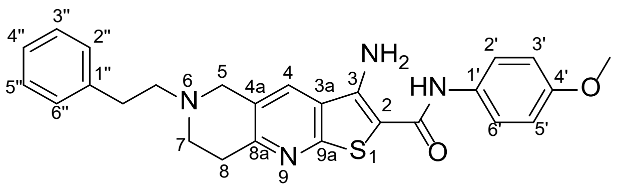

The reaction was carried out following General Procedure **A** using carbonitrile **3** (0.10 g, 0.34 mmol), chloride **4e** (68.0 mg, 0.34 mmol) and anhydrous sodium carbonate (72.0 mg, 0.68 mmol) in absolute ethanol (3.00 mL) for 48 h to give the *title compound* **5e** (0.11 g, 71%) as a yellow solid. m.p. > 230 °C. δ_H (400 MHz, $(CD_3)_2SO$) 2.75-2.77 (2H, m, 6-CH_2), 2.85-2.88 (4H, m, H-7 and 1''-CH_2), 3.02-3.03 (2H, m, H-8), 3.74 (3H, s, 4'-OCH_3), 3.77 (2H, s, H-5), 6.89 (2H, d, J = 7.4 Hz, H-3' and H-5'), 7.18-7.20 (1H, m, H-4''), 7.26-7.28 (6H, m, H-2'', H-3'', H-5'', H-6'' and NH_2), 7.56 (2H, d, J = 7.4 Hz, H-2' and H-6'), 8.18 (1H, s, H-4), 9.27 (1H, br s, NH). δ_C (100 MHz, $(CD_3)_2SO$) 32.3 (C-8), 33.0 (1''-CH_2), 49.9 (C-7), 54.6 (C-5), 55.1 (4'-OCH_3), 58.9 (6-CH_2), 96.1 (C-2), 113.5 (C-3' and C-5'), 122.9 (C-2' and C-6'), 124.3 (C-3a), 125.8 (C-4''), 126.4 (C-4a), 128.2 (C-3'' and C-5''), 128.6 (C-4), 128.7 (C-2'' and C-6''), 131.9 (C-1'), 140.3 (C-1''), 146.4 (C-3), 155.4 (C-4'), 156.5 (C-8a), 156.6 (C-9a), 163.8 (2-CONH). ν_{max} (ATR)/cm^{-1} 3430 (N-H amide), 3317 (N-H amine), 2946 (C-H aromatic), 2830 (C-H alkane), 1594 (C=O amide), 1495 (C=C aromatic), 1411 (-C-H bending), 1233 (C-N aromatic),

1108 (C-O ether), 1034 (C-N aliphatic). *m/z* (ESI^+): 459 (MH^+, 100%), 227 (15%), 101 (10%). HRMS (ESI^+) found (MH^+): 459.1847 $C_{26}H_{27}N_4O_2S$ requires 459.1849.

3,4-Bis(methoxymethoxy)benzaldehyde 7a

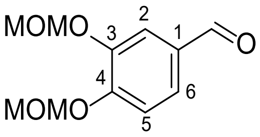

The reaction was carried out following General Procedure **B** using 3,4-dihydroxybenzaldehyde **6** (1.00 g, 7.24 mmol), DIPEA (5.00 mL, 29.0 mmol) and MOMCl (1.43 mL, 19.0 mmol) in dry CH_2Cl_2 (10.0 mL) and purified using flash chromatography (3:1 petroleum ether : ethyl acetate) to give the *title compound* **7a** (1.34 g, 83%) as a white solid. mp. 53-55 °C. Lit m.p. 51-52 °C.[1] δ_H (400 MHz, $CDCl_3$) 3.52 (3H, s, CH_3), 3.53 (3H, s, CH_3), 5.30 (2H, s, CH_2), 5.33 (2H, s, CH_2), 7.29 (1H, d, J = 8.4 Hz, H-5), 7.51 (1H, dd, J = 8.4, 1.9 Hz, H-6), 7.68 (1H, d, J = 1.9 Hz, H-2), 9.87 (1H, s, CH=O). The ^1H NMR values were in agreement with the literature values.[1]

3-(Methoxymethoxy)benzaldehyde 7g

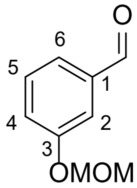

The reaction was carried out following General Procedure **B** using 3-hydroxybenzaldehyde **16** (0.20 g, 1.64 mmol), DIPEA (1.10 mL, 6.56 mmol) and MOMCl (0.32 mL, 4.26 mmol) in dry CH_2Cl_2 (2.0 mL) and purified using flash chromatography (4:1 petroleum ether : ethyl acetate) to give the *title compound* **80i** (0.23 g, 85%) as a colourless oil. δ_H (400 MHz, $CDCl_3$) 3.49 (3H, s, $MOMCH_3$), 5.23 (2H, s, $MOMCH_2$), 7.29-7.32 (1H, m, H-4), 7.46 (1H, t, J = 7.6 Hz, H-5), 7.52-7.55 (2H, m, H-2 and H-6), 9.98 (1H, s, COH). The ^1H NMR values were in agreement with the literature values.[2]

(*E*)-5-(3-(3',4'-Bis(methoxymethoxy)phenyl)acryloyl)-6-methyl-2-thioxo-1,2-dihydropyridine-3-carbonitrile 9a

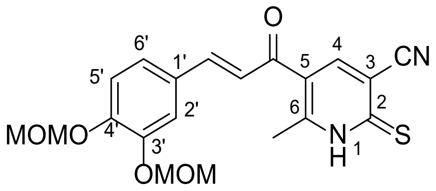

The reaction was carried out following General Procedure **C** using carbonitrile **8** (0.15 g, 0.78 mmol), 3,4-bis(methoxymethoxy)benzaldehyde **7a** (0.18 g, 0.78 mmol) in dry absolute ethanol (4.8 mL), anhydrous potassium hydroxide (0.22 g, 3.90 mmol) in dry methanol (1.95 mL) to give the crude product. This was purified using flash chromatography (19:1 dichloromethane : methanol) to give the *title compound* **9a** (0.20 g, 65%) as a yellow solid. m.p. 200-202 °C. δ_H (400 MHz, $(CD_3)_2SO$) 2.62 (3H, s, 6-CH_3), 3.41 and 3.43 (6H, s, 2 × $MOMCH_3$), 5.26 and 5.27 (4H, s, 2 × $MOMCH_2$), 7.16 (1H, d, *J* = 8.5 Hz, H-5'), 7.46-7.49 (2H, m, H-6' and 5-COC<u>H</u>CH), 7.56-7.60 (2H, m, H-2' and 5-COCHC<u>H</u>), 8.59 (1H, s, H-4), 14.30 (1H, br s, NH). δ_C (100 MHz, $(CD_3)_2SO$) 19.0 (6-CH_3), 55.88 and 55.92 (2 × $MOMCH_3$), 94.4 and 94.9 (2 × $MOMCH_2$), 113.3 (C-3), 116.3 (C-5'), 116.7 (CN), 117.4 (C-2'), 122.45 and 122.48 (C-5 and 5-CO<u>C</u>HCH), 124.5 (C-6'), 128.5 (C-1'), 144.0 (C-4), 144.9 (5-COCH<u>C</u>H), 146.6 (C-3'), 149.6 (C-4'), 157.0 (C-6), 178.9 (C-2), 187.3 (5-CO). ν_{max} (ATR)/cm^{-1} 3180 (N-H amine), 2966 (C-H aromatic), 2839 (C-H alkane), 2235 (CN nitrile), 1657 (C=O carbonyl), 1571 (C=C aromatic), 1507 (-C-H bending), 1181 (C-O ether), 1078 (C-N aromatic). *m/z* (ESI^+): 423 (MNa^+, 100%), 381 (15%). HRMS (ESI^+) found (MNa^+): 423.0983 $C_{20}H_{20}N_2NaO_5S$ requires 423.0985.

(*E*)-5-(3-(4'-Methoxyphenyl)acryloyl)-6-methyl-2-thioxo-1,2-dihydropyridine-3-carbonitrile 9b

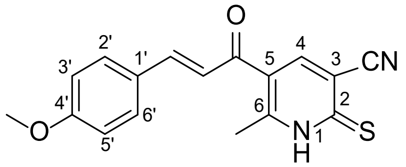

The reaction was carried out following General Procedure **C** using carbonitrile **8** (0.20 g, 1.04 mmol), *p*-methoxybenzaldehyde **7b** (0.14 g, 1.04 mmol) in dry absolute ethanol (5.2 mL), anhydrous potassium hydroxide (0.30 g, 5.20 mmol) in dry methanol (2.08 mL) and purified

with flash chromatography (19:1 dichloromethane : methanol) to give the *title compound* **9b** (0.20 g, quant.) as a yellow solid. m.p. > 230 °C. δ_H (400 MHz, $(CD_3)_2SO$) 2.60 (3H, s, 6-CH_3), 3.82 (3H, s, 4'-OCH_3), 7.01 (2H, d, J = 8.8 Hz, H-3' and H-5'), 7.50 (1H, d, J = 15.7 Hz, 5-COCHC<u>H</u>), 7.61 (1H, d, J = 15.7 Hz, 5-COC<u>H</u>CH), 7.82 (2H, d, J = 8.8 Hz, H-2' and H-6'), 8.55 (1H, s, H-4), 14.30 (1H, br s, NH). δ_C (100 MHz, $(CD_3)_2SO$) 19.9 (6-CH_3), 55.4 (4'-OCH_3), 113.7 (C-3), 114.4 (C-3' and C-5'), 116.7 (CN), 121.5 (5-CO<u>C</u>HCH), 122.3 (C-5), 127.2 (C-1'), 130.9 (C-2' and C-6'), 143.6 (C-4), 144.6 (5-COCH<u>C</u>H), 157.4 (C-6), 161.5 (C-4'), 180.5 (C-2), 187.2 (5-CO). ν_{max} (ATR)/cm^{-1} 3386 (N-H amine), 2971 (C-H aromatic), 2845 (C-H alkane), 2236 (CN nitrile), 1658 (C=O carbonyl), 1576 (C=C aromatic), 1509 (-C-H bending), 1169 (C-O ether), 1077 (C-N aromatic). *m/z* (ESI^+): 333 (MNa^+, 100%), 101 (30%). HRMS (ESI^+) found (MNa^+): 333.0659 $C_{17}H_{14}N_2NaO_2S$ requires 333.0668.

(*E*)-6-Methyl-5-(3-(4'-morpholinophenyl)acryloyl)-2-thioxo-1,2-dihydropyridine-3-carbonitrile 9c

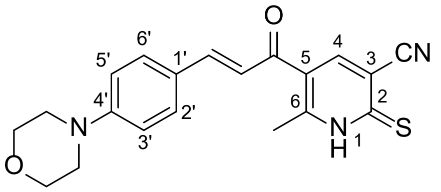

The reaction was carried out following General Procedure **C** using carbonitrile **8** (0.10 g, 0.52 mmol), 4-morpholinobenzaldehyde **7c** (0.10 g, 0.52 mmol) in dry absolute ethanol (3.2 mL), anhydrous potassium hydroxide (0.15 g, 2.60 mmol) in dry methanol (1.3 mL) to give the crude product. This was purified using flash chromatography (19:1 dichloromethane : methanol) to give the *title compound* **9c** (0.11 g, 60%) as an orange solid. m.p. > 230 °C. δ_H (400 MHz, $(CD_3)_2SO$) 2.62 (3H, s, 6-CH_3), 3.27 (4H, t, J = 4.8 Hz, 2 × NC<u>H</u>$_2$CH$_2$O), 3.74 (4H, t, J = 4.8 Hz, 2 × NCH$_2$C<u>H</u>$_2$O), 6.98 (2H, d, J = 8.9 Hz, H-3' and H-5'), 7.41 (1H, d, J = 15.9 Hz, 5-COC<u>H</u>CH), 7.59 (1H, d, J = 15.9 Hz, 5-COCHC<u>H</u>), 7.72 (2H, d, J = 8.9 Hz, H-2' and H-6'), 8.62 (1H, s, H-4), 14.26 (1H, br s, NH). δ_C (100 MHz, $(CD_3)_2SO$) 19.0 (6-CH_3), 47.0 (2 × *N*-CH_2), 65.9 (2 × *O*-CH_2), 113.3 (C-3), 113.9 (C-3' and C-5'), 116.7 (CN), 119.6 (5-CO<u>C</u>HCH), 122.8 (C-5), 124.4 (C-1'), 130.8 (C-2' and C-6'), 144.0 (C-4), 145.5 (5-COCH<u>C</u>H), 152.8 (C-4'), 157.6 (C-6), 178.7 (C-2), 186.9 (5-CO). ν_{max} (ATR)/cm^{-1} 3207 (N-H amine), 2841 (C-H aromatic), 2233 (CN nitrile), 1656 (C=O carbonyl), 1579 (C=C aromatic), 1514 (-C-H bending), 1171 (C-O ether), 1050 (C-N aromatic). *m/z* (ESI^+): 388 (MNa^+, 100%), 217 (20%). HRMS (ESI^+) found (MNa^+): 388.1077 $C_{20}H_{19}N_3NaO_2S$ requires 388.1090.

5-Cinnamoyl-6-methyl-2-thioxo-1,2-dihydropyridine-3-carbonitrile 9d

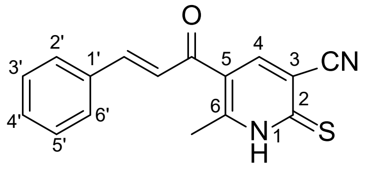

The reaction was carried out following General Procedure **C** using carbonitrile **8** (0.20 g, 1.04 mmol), benzaldehyde **7d** (0.11 g, 1.04 mmol) in dry absolute ethanol (4.4 mL), anhydrous potassium hydroxide (0.29 g, 5.20 mmol) in dry methanol (1.6 mL) to give the *title compound* **9d** (0.18 g, 61%) as a yellow solid. m.p. > 230 °C. δ_H (400 MHz, $(CD_3)_2SO$) 2.64 (3H, s, 6-CH_3), 7.46 (3H, m, H-3', H-4' and H-5'), 7.66 (2H, br s, 5-COCHC$\underline{H}$ and 5-COC$\underline{H}$CH), 7.84-7.87 (2H, m, H-2' and H-6'), 8.70 (1H, s, H-4), 14.30 (1H, br s, NH). δ_C (100 MHz, $(CD_3)_2SO$) 19.2 (6-CH_3), 113.4 (C-3), 116.3 (CN), 122.1 (C-5), 123.8 (5-CO$\underline{C}$HCH), 128.9 and 129.0 (C-2', C-3', C-5' and C-6'), 130.8 (C-4'), 134.5 (C-1'), 144.1 (C-4), 144.7 (5-COCH$\underline{C}$H), 157.5 (C-6), 179.0 (C-2), 187.1 (5-CO). ν_{max} (ATR)/cm^{-1} 3154 (N-H amine), 2925 (C-H aromatic), 2855 (C-H alkane), 2237 (CN nitrile), 1662 (C=O carbonyl), 1587 (C=C aromatic), 1493 (-C-H bending), 1184 (C-O ether), 1077 (C-N aromatic). *m/z* (ESI^+): 303 (MNa^+, 80%), 237 (100%), 215 (50%). HRMS (ESI^+) found (MNa^+): 303.0556 $C_{16}H_{12}N_2NaOS$ requires 303.0563.

(*E*)-5-(3-(3'-Methoxyphenyl)acryloyl)-6-methyl-2-thioxo-1,2-dihydropyridine-3-carbonitrile 9e

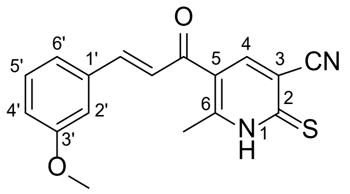

The reaction was carried out following General Procedure **C** using carbonitrile **8** (0.30 g, 1.56 mmol), *m*-anisaldehyde **7e** (0.21 g, 1.56 mmol) in dry absolute ethanol (7.6 mL), anhydrous potassium hydroxide (0.44 g, 7.80 mmol) in dry methanol (2.90 mL) to give the *title compound* **9e** (0.37 g, 77%) as a yellow solid. m.p. > 230 °C. δ_H (400 MHz, $(CD_3)_2SO$) 2.64 (3H, s, 6-CH_3), 3.82 (3H, s, 3'-OCH_3), 7.04 (1H, dd, J = 7.9, 1.1 Hz, H-4'), 7.39 (1H, t, J = 7.9 Hz, H-5'), 7.42-7.44 (2H, m, H-2' and H-6'), 7.61 (1H, d, J = 15.9 Hz, 5-COCHC$\underline{H}$ or 5-COC$\underline{H}$CH), 7.66 (1H, d, J = 15.9 Hz, 5-COCHC$\underline{H}$ or 5-COC$\underline{H}$CH), 8.67 (1H, s, H-4), 14.30 (1H, br s, NH). δ_C (100 MHz, $(CD_3)_2SO$) 19.2 (6-CH_3), 55.3 (3'-OCH_3), 113.3 (C-3), 113.9 (C-2'), 116.6 (C-4'

and CN), 121.6 (C-6'), 122.1 (C-5), 124.1 (5-COCHCH), 129.9 (C-5'), 135.8 (C-1'), 144.1 (C-4), 144.7 (5-COCHCH), 157.4 (C-6), 159.6 (C-3'), 179.1 (C-2), 187.2 (5-CO). ν_{max} (ATR)/cm^{-1} 3187 (N-H amine), 2978 (C-H aromatic), 2844 (C-H alkane), 2224 (CN nitrile), 1661 (C=O carbonyl), 1588 (C=C aromatic), 1508 (-C-H bending), 1182 (C-O ether) 1078 (C-N aromatic). *m/z* (ESI$^+$): 333 (MNa$^+$, 100%). HRMS (ESI$^+$) found (MNa$^+$): 333.0658 $C_{17}H_{14}N_2NaO_2S$ requires 333.0668.

(*E*)-5-(3-(2'-Methoxyphenyl)acryloyl)-6-methyl-2-thioxo-1,2-dihydropyridine-3-carbonitrile 9f

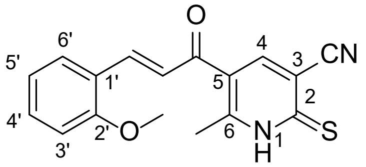

The reaction was carried out following General Procedure **C** using carbonitrile **8** (0.15 g, 0.78 mmol), 2-methoxybenzaldehyde **7f** (0.09 mL, 0.78 mmol) in dry absolute ethanol (4.8 mL), anhydrous potassium hydroxide (0.22 g, 3.90 mmol) in dry methanol (1.95 mL) to give the *title compound* **9f** (0.17 g, 57%) as a yellow solid. m.p. > 230 °C. δ_H (400 MHz, $(CD_3)_2SO$) 2.64 (3H, s, 6-CH_3), 3.88 (3H, s, 2'-OCH_3), 7.03 (1H, t, *J* = 7.5 Hz, H-5'), 7.11 (1H, t, *J* = 7.5 Hz, H-3'), 7.46 (1H, td, *J* = 7.5, 1.9 Hz, H-4'), 7.61 (1H, d, *J* = 15.9 Hz, 5-COCHCH), 7.96 (1H, dd, *J* = 7.5, 1.9 Hz, H-6'), 7.97 (1H, d, *J* = 15.9 Hz, 5-COCHCH), 8.67 (1H, s, H-4), 14.30 (1H, br s, NH). δ_C (100 MHz, $(CD_3)_2SO$) 19.3 (6-CH_3), 55.7 (2'-OCH_3), 111.8 (C-3'), 113.3 (C-3), 116.6 (CN), 120.6 (C-5'), 122.3 (C-5), 122.7 (C-1'), 123.4 (5-COCHCH), 128.3 (C-6'), 132.6 (C-4'), 138.8 (5-COCHCH), 144.0 (C-4), 157.5 (C-6), 158.2 (C-2'), 179.1 (C-2), 187.0 (5-CO). ν_{max} (ATR)/cm^{-1} 3170 (N-H amine), 2980 (C-H aromatic), 2830 (C-H alkane), 2224 (CN nitrile), 1660 (C=O carbonyl), 1574 (C=C aromatic), 1504 (-C-H bending), 1172 (C-O ether), 1084 (C-N aromatic). *m/z* (ESI$^+$): 333 (MNa$^+$, 100%). HRMS (ESI$^+$) found (MNa$^+$): 333.0659 $C_{17}H_{14}N_2NaO_2S$ requires 333.0668.

(*E*)-5-(3-(3'-(Methoxymethoxy)phenyl)acryloyl)-6-methyl-2-thioxo-1,2-dihydropyridine-3-carbonitrile 9g

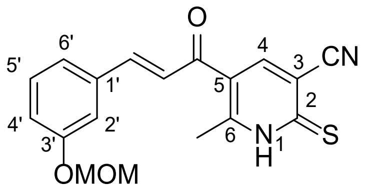

The reaction was carried out following General Procedure **C** using carbonitrile **8** (0.23 g, 1.20 mmol), benzaldehyde **7g** (0.20 g, 1.20 mmol) in dry absolute ethanol (7.4 mL), anhydrous potassium hydroxide (0.34 g, 6.3 mmol) in dry methanol (3.0 mL) to give the *title compound* **9g** (0.18 g, 44%) as a yellow solid. m.p. 209-211 °C. δ_H (400 MHz, $(CD_3)_2SO$) 2.64 (3H, s, 6-CH_3), 3.39 (3H, s, $MOMCH_3$), 5.25 (2H, s, $MOMCH_2$), 7.13 (1H, dd, *J* = 7.9, 2.2 Hz, H-4'), 7.38 (1H, t, *J* = 7.9 Hz, H-5'), 7.47-7.50 (2H, m, H-2' and H-6'), 7.58-7.66 (2H, m, 5-COC$\underline{H}$CH and 5-COCHC$\underline{H}$), 8.66 (1H, s, H-4), 14.31 (1H, br s, NH). δ_C (100 MHz, $(CD_3)_2SO$) 19.2 (6-CH_3), 55.7 ($MOMCH_3$), 93.8 ($MOMCH_2$), 113.3 (C-3), 116.4 (C-2'), 116.7 (CN), 118.4 (C-4'), 122.1 (C-5), 122.7 (C-6'), 124.2 (5-CO$\underline{C}$HCH), 130.0 (C-5'), 135.9 (C-1'), 144.1 (C-4), 144.5 (5-COCH$\underline{C}$H), 157.0 (C-3'), 157.5 (C-6), 179.1 (C-2), 187.2 (5-CO). ν_{max} (ATR)/cm^{-1} 3147 (N-H amine), 2923 (C-H aromatic), 2848 (C-H alkane), 2240 (CN nitrile), 1662 (C=O carbonyl), 1593 (C=C aromatic), 1509 (-C-H bending), 1174 (C-O ether) 1010 (C-N aromatic). *m/z* (ESI$^+$): 363 (MNa$^+$, 100%). HRMS (ESI$^+$) found (MNa$^+$): 363.0770 $C_{18}H_{16}N_2NaO_3S$ requires 363.0774.

(*E*)-6-Methyl-2-thioxo-5-(3-(2'-(trifluoromethyl)phenyl)acryloyl)-1,2-dihydropyridine-3-carbonitrile 9h

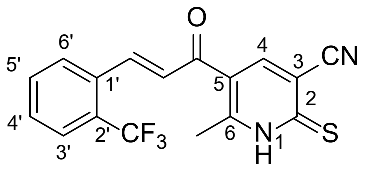

The reaction was carried out following General Procedure **C** using carbonitrile **8** (0.20 g, 1.04 mmol), 2-(trifluoromethyl)benzaldehyde **7h** (0.18 g, 1.04 mmol) in dry absolute ethanol (6.4 mL), anhydrous potassium hydroxide (0.29 g, 5.2 mmol) in dry methanol (2.60 mL) to give the *title compound* **9h** (0.18 g, 49%) as a yellow solid. m.p. > 230 °C. δ_H (400 MHz, $(CD_3)_2SO$) 2.69 (3H, s, 6-CH_3), 7.68 (1H, t, *J* = 7.5 Hz, H-4'), 7.78-7.91 (4H, m, H-3', H-5', 5-COC$\underline{H}$CH,

and 5-COCHC<u>H</u>), 8.32 (1H, d, J = 7.5 Hz, H-6'), 8.82 (1H, s, H-4), 14.35 (1H, br s, NH). δ_C (100 MHz, $(CD_3)_2SO$) 19.6 (6-CH_3), 113.3 (C-3), 116.7 (CN), 121.3 (C-5), 124.6 (q, $^1J_{F/C}$ = 273.5 Hz, CF_3), 127.2 (q, $^3J_{F/C}$ = 5.7 Hz, C-3'), 127.4 (5-CO<u>C</u>HCH and 5-COCH<u>C</u>H), 128.9 (C-6'), 130.6 (q, $^2J_{F/C}$ = 29.4 Hz, C-2'), 131.0 (C-4'), 132.8 (C-5'), 138.5 (C-1'), 144.3 (C-4), 157.3 (C-6), 179.4 (C-2), 186.1 (5-CO). ν_{max} (ATR)/cm^{-1} 3064 (N-H amine), 2968 (C-H aromatic), 2845 (C-H alkane), 2241 (CN nitrile), 1669 (C=O carbonyl), 1595 (C=C aromatic), 1509 (-C-H bending), 1313 (C-F), 1079 (C-N aromatic). *m/z* (ESI^+): 371 (MNa^+, 100%). HRMS (ESI^+) found (MNa^+): 371.0441 $C_{17}H_{11}F_3N_2NaOS$ requires 371.0436.

(*E*)-5-(3-(2'-Fluorophenyl)acryloyl)-6-methyl-2-thioxo-1,2-dihydropyridine-3-carbonitrile 9i

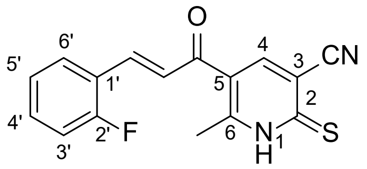

The reaction was carried out following General Procedure **C** using carbonitrile **8** (0.15 g, 0.78 mmol), 2-fluorobenzaldehyde **7i** (0.08 mL, 0.78 mmol) in dry absolute ethanol (4.8 mL), anhydrous potassium hydroxide (0.22 g, 3.90 mmol) in dry methanol (1.95 mL) to give the *title compound* **9i** (96.0 mg, 42%) as a yellow solid. m.p. > 230 °C. δ_H (400 MHz, $(CD_3)_2SO$) 2.66 (3H, s, 6-CH_3), 7.29-7.34 (2H, m, H-3' and H-5'), 7.50-7.56 (1H, m, H-4'), 7.72 (1H, d, J = 15.9 Hz, 5-COC<u>H</u>CH), 7.76 (1H, d, J = 15.9 Hz, 5-COCHC<u>H</u>), 8.10 (1H, td, J = 7.7, 1.4 Hz, H-6'), 8.73 (1H, s, H-4), 14.33(1H, br s, NH). δ_C (100 MHz, $(CD_3)_2SO$) 19.3 (6-CH_3), 113.4 (C-3), 116.1 (d, $^2J_{F/C}$ = 24.1 Hz, C-3'), 116.6 (CN), 122.1 (d, $^2J_{F/C}$ = 11.0 Hz, C-1'), 122.2 (C-5), 124.9 (C-5'), 125.7 (5-CO<u>C</u>HCH), 129.0 (d, $^3J_{F/C}$ = 2.1 Hz, C-6'), 133.0 (d, $^3J_{F/C}$ = 8.8 Hz, C-4'), 135.4 (5-COCH<u>C</u>H), 144.2 (C-4), 157.4 (C-6), 160.4 (d, $^1J_{F/C}$ = 253.2 Hz, C-2'), 179.2 (C-2), 186.6 (5-CO). ν_{max} (ATR)/cm^{-1} 3149 (N-H amine), 2949 (C-H aromatic), 2855 (C-H alkane), 2232 (CN nitrile), 1667 (C=O carbonyl), 1591 (C=C aromatic), 1503 (-C-H bending), 1219 (C-F), 1181 (C-O ether), 1082 (C-N aromatic). *m/z* (ESI^+): 321 (MNa^+, 100%). HRMS (ESI^+) found (MNa^+): 321.0472 $C_{16}H_{11}FN_2NaOS$ requires 321.0468.

(*E*)-5-(3-(3'-Fluorophenyl)acryloyl)-6-methyl-2-thioxo-1,2-dihydropyridine-3-carbonitrile 9j

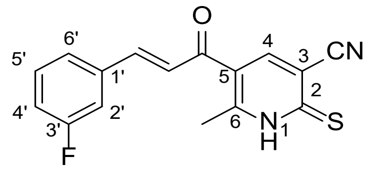

The reaction was carried out following General Procedure **C** using carbonitrile **8** (0.15 g, 0.78 mmol), 3-fluorobenzaldehyde **7j** (0.08 mL, 0.78 mmol) in dry absolute ethanol (4.8 mL), anhydrous potassium hydroxide (0.22 g, 3.90 mmol) in dry methanol (1.95 mL) to give the *title compound* **9j** (0.13 g, 57%) as a yellow solid. m.p. > 230 °C. δ_H (400 MHz, $(CD_3)_2SO$) 2.66 (3H, s, 6-CH_3), 7.30 (1H, td, *J* = 8.7, 2.5 Hz, H-4'), 7.50 (1H, q, *J* = 7.6 Hz, H-5'), 7.65 (1H, d, *J* = 15.9 Hz, 5-COCHCH̲), 7.67 (1H, d, *J* = 7.6 Hz, H-6'), 7.74 (1H, d, *J* = 15.9 Hz, 5-COCH̲CH), 7.81 (1H, d, *J* = 9.9 Hz, H-2'), 8.73 (1H, s, H-4), 14.32 (1H, br s, NH). δ_C (100 MHz, $(CD_3)_2SO$) 19.3 (6-CH_3), 113.4 (C-3), 114.7 (d, $^2J_{F/C}$ = 22.0 Hz, C-2'), 116.6 (CN), 117.5 (d, $^2J_{F/C}$ = 22.0 Hz, C-4'), 121.8 (C-5), 125.0 (5-COC̲HCH), 125.8 (d, $^4J_{F/C}$ = 2.3 Hz, C-6'), 130.8 (d, $^3J_{F/C}$ = 8.5 Hz, C-5'), 137.0 (d, $^3J_{F/C}$ = 8.5 Hz, C-1'), 143.1 (5-COCHC̲H), 144.2 (C-4), 157.8 (C-6), 162.5 (d, $^1J_{F/C}$ = 243.6 Hz, C-3'), 179.1 (C-2), 186.8 (5-CO). ν_{max} (ATR)/cm^{-1} 3088 (N-H amine), 2923 (C-H aromatic), 2853 (C-H alkane), 2236 (CN nitrile), 1667 (C=O carbonyl), 1592 (C=C aromatic), 1507 (-C-H bending), 1231 (C-F), 1075 (C-N aromatic). *m/z* (ESI^+): 321 (MNa^+, 100%). HRMS (ESI^+) found (MNa^+): 321.0462 $C_{16}H_{11}FN_2NaOS$ requires 321.0468.

(*E*)-5-(3-(4'-Fluorophenyl)acryloyl)-6-methyl-2-thioxo-1,2-dihydropyridine-3-carbonitrile 9k

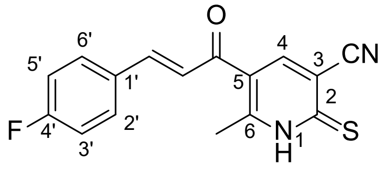

The reaction was carried out following General Procedure **C** using carbonitrile **8** (0.20 g, 1.04 mmol), 4-fluorobenzaldehyde **7k** (0.13 g, 1.04 mmol) in dry absolute ethanol (6.4 mL), anhydrous potassium hydroxide (0.29 g, 5.2 mmol) in dry methanol (2.60 mL) to give the *title compound* **9k** (0.18 g, 58%) as a yellow solid. m.p. > 230 °C. δ_H (400 MHz, $(CD_3)_2SO$) 2.65 (3H, s, 6-CH_3), 7.31 (2H, t, *J* = 8.8 Hz, H-3' and H-5'), 7.61 (1H, d, *J* = 15.9 Hz, 5-COCHCH̲

or 5-COC<u>H</u>CH), 7.67 (1H, d, J = 15.9 Hz, 5-COCHC<u>H</u> or 5-COC<u>H</u>CH), 7.93-7.96 (2H, m, H-2' and H-6'), 8.70 (1H, s, H-4), 14.32 (1H, br s, NH). δ_C (100 MHz, $(CD_3)_2SO$) 19.2 (6-CH_3), 113.4 (C-3), 115.9 (d, $^2J_{F/C}$ = 21.5 Hz, C-3' and C-5'), 116.6 (CN), 122.1 (C-5), 123.7 (5-CO<u>C</u>HCH), 131.1 ($^4J_{F/C}$ = 3.1 Hz, C-1'), 131.4 (d, $^3J_{F/C}$ = 8.5 Hz, C-2' and C-6'), 143.5 (5-COCH<u>C</u>H), 144.1 (C-4), 157.5 (C-6), 163.8 (d, $^1J_{F/C}$ = 250.1 Hz, C-4'), 179.2 (C-2), 187.0 (5-CO). ν_{max} (ATR)/cm^{-1} 3050 (N-H amine), 2926 (C-H aromatic), 2855 (C-H alkane), 2233 (CN nitrile), 1668 (C=O carbonyl), 1588 (C=C aromatic), 1505 (-C-H bending), 1223 (C-F), 1086 (C-N aromatic). *m/z* (ESI^+): 321 (MNa^+, 100%). HRMS (ESI^+) found (MNa^+): 321.0460 $C_{16}H_{11}FN_2NaOS$ requires 321.0468.

(*E*)-5-(3-(2'-Chlorophenyl)acryloyl)-6-methyl-2-thioxo-1,2-dihydropyridine-3-carbonitrile 9l

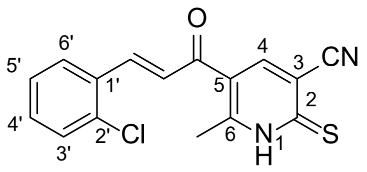

The reaction was carried out following General Procedure **C** using carbonitrile **8** (0.15 g, 0.78 mmol), 2-chlorobenzaldehyde **7l** (0.09 mL, 0.78 mmol) in dry absolute ethanol (4.8 mL), anhydrous potassium hydroxide (0.22 g, 3.90 mmol) in dry methanol (1.95 mL) to give the *title compound* **9l** (0.11 g, 44%) as a yellow solid. m.p. > 230 °C. δ_H (400 MHz, $(CD_3)_2SO$) 2.68 (3H, s, 6-CH_3), 7.45-7.50 (2H, m, H-4' and H-5'), 7.58 (1H, dd, J = 7.3, 1.7 Hz, H-3'), 7.77 (1H, d, J = 15.7 Hz, 5-COC<u>H</u>CH), 7.96 (1H, d, J = 15.7 Hz, 5-COCHC<u>H</u>), 8.19 (1H, dd, J = 7.3, 1.7 Hz, H-6'), 8.79 (1H, s, H-4), 14.34 (1H, br s, NH). δ_C (100 MHz, $(CD_3)_2SO$) 19.4 (6-CH_3), 113.3 (C-3), 116.6 (CN), 121.6 (C-5), 126.1 (5-CO<u>C</u>HCH), 127.6 (C-5'), 128.7 (C-6'), 130.0 (C-3'), 132.1 (C-4'), 132.2 (C-1'), 134.4 (C-2'), 138.7 (5-COCH<u>C</u>H), 144.2 (C-4), 157.1 (C-6), 179.2 (C-2), 186.3 (5-CO). ν_{max} (ATR)/cm^{-1} 3091 (N-H amine), 2922 (C-H aromatic), 2858 (C-H alkane), 2230 (CN nitrile), 1663 (C=O carbonyl), 1596 (C=C aromatic), 1506 (-C-H bending), 1181 (C-O ether), 1085 (C-N aromatic), 759 (C-Cl). *m/z* (ESI^+): 339 ($^{37}ClMNa^+$, 40%), 337 ($^{35}ClMNa^+$, 100%). HRMS (ESI^+) found ($^{37}ClMNa^+$): 339.0128 $C_{16}H_{11}{}^{37}ClN_2NaOS$ requires 339.0146. Found ($^{35}ClMNa^+$): 337.0154 $C_{16}H_{11}{}^{35}ClN_2NaOS$ requires 337.0173.

(*E*)-5-(3-(3'-Chlorophenyl)acryloyl)-6-methyl-2-thioxo-1,2-dihydropyridine-3-carbonitrile 9m

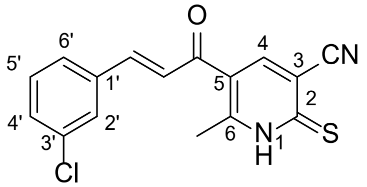

The reaction was carried out following General Procedure **C** using carbonitrile **8** (0.10 g, 0.52 mmol), 3-chlorobenzaldehyde **7m** (73.0 mg, 0.52 mmol) in dry absolute ethanol (3.2 mL), anhydrous potassium hydroxide (0.14 g, 2.60 mmol) in dry methanol (1.3 mL) to give the *title compound* **9m** (0.10 g, 63%) as a yellow solid. m.p. > 230 °C. δ_H (400 MHz, $(CD_3)_2SO$) 2.66 (3H, s, 6-CH_3), 7.46-7.53 (2H, m, H-4' and H-5'), 7.63 (1H, d, J = 15.9 Hz, 5-COC$\underline{H}$CH), 7.75 (1H, d, J = 15.9 Hz, 5-COCHC$\underline{H}$), 7.80 (1H, dt, J = 7.3, 1.6 Hz, H-6'), 8.02 (1H, t, J = 1.6 Hz, H-2'), 8.73 (1H, s, H-4), 14.33 (1H, br s, NH). δ_C (100 MHz, $(CD_3)_2SO$) 19.3 (6-CH_3), 113.4 (C-3), 116.6 (CN), 121.5 (C-5), 125.1 (5-CO$\underline{C}$HCH), 127.8 (C-6'), 129.3 (C-2'), 130.3 (C-4'), 130.7 (C-5'), 133.8 (C-3'), 136.7 (C-1'), 142.9 (5-COCH$\underline{C}$H), 144.4 (C-4), 157.8 (C-6), 179.1 (C-2), 187.4 (5-CO). ν_{max} (ATR)/cm^{-1} 3180 (N-H amine), 2976 (C-H aromatic), 2889 (C-H alkane), 2234 (CN nitrile), 1663 (C=O carbonyl), 1587 (C=C aromatic), 1502 (-C-H bending), 1074 (C-N aromatic), 799 (C-Cl). *m/z* (ESI^+): 339 ($^{37}ClMNa^+$, 40%), 337 ($^{35}ClMNa^+$, 100%). HRMS (ESI^+) found ($^{37}ClMNa^+$): 339.0149 $C_{16}H_{11}{}^{37}ClN_2NaOS$ requires 339.0146. Found ($^{35}ClMNa^+$): 337.0171 $C_{16}H_{11}{}^{35}ClN_2NaOS$ requires 337.0173.

(*E*)-5-(3-(4'-Chlorophenyl)acryloyl)-6-methyl-2-thioxo-1,2-dihydropyridine-3-carbonitrile 9n

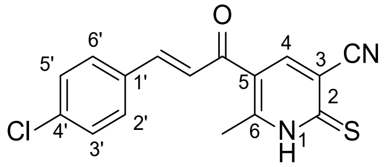

The reaction was carried out following General Procedure **C** using carbonitrile **8** (0.15 g, 0.78 mmol), 4-chlorobenzaldehyde **7n** (0.11 g, 0.78 mmol) in dry absolute ethanol (4.8 mL), anhydrous potassium hydroxide (0.22 g, 3.90 mmol) in dry methanol (1.95 mL) to give the *title compound* **9n** (0.14 g, 55%) as a yellow solid. m.p. > 230 °C. δ_H (400 MHz, $(CD_3)_2SO$) 2.65 (3H, s, 6-CH_3), 7.53 (2H, d, J = 8.4 Hz, H-3' and H-5'), 7.64 (1H, d, J = 15.9 Hz, 5-COCHC$\underline{H}$),

7.70 (1H, d, J = 15.9 Hz, 5-CO<u>CH</u>CH), 7.90 (2H, d, J = 8.4 Hz, H-2' and H-6'), 8.71 (1H, s, H-4), 14.31 (1H, br s, NH). δ_C (100 MHz, $(CD_3)_2SO$) 19.2 (6-CH_3), 113.3 (C-3), 116.6 (CN), 121.9 (C-5), 124.4 (5-CO<u>C</u>HCH), 128.9 (C-3' and C-5'), 130.7 (C-2' and C-6'), 133.5 (C-1'), 135.3 (C-4'), 143.2 (5-COCH<u>C</u>H), 144.1 (C-4), 157.6 (C-6), 179.1 (C-2), 186.9 (5-CO). ν_{max} (ATR)/cm^{-1} 3230 (N-H amine), 2923 (C-H aromatic), 2853 (C-H alkane), 2225 (CN nitrile), 1660 (C=O carbonyl), 1587 (C=C aromatic), 1492 (-C-H bending), 1079 (C-N aromatic), 820 (C-Cl). *m/z* (ESI^+): 339 ($^{37}ClMNa^+$, 40%), 337 ($^{35}ClMNa^+$, 100%). HRMS (ESI^+) found ($^{37}ClMNa^+$): 339.0141 $C_{16}H_{11}{}^{37}ClN_2NaOS$ requires 339.0146. Found ($^{35}ClMNa^+$): 337.0166 $C_{16}H_{11}{}^{35}ClN_2NaOS$ requires 337.0173.

(*E*)-5-(3-(2'-Bromophenyl)acryloyl)-6-methyl-2-thioxo-1,2-dihydropyridine-3-carbonitrile 9o

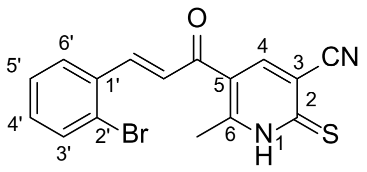

The reaction was carried out following General Procedure **C** using carbonitrile **8** (0.20 g, 1.04 mmol), 2-bromobenzaldehyde **7o** (0.12 mL, 1.04 mmol) in dry absolute ethanol (6.4 mL), anhydrous potassium hydroxide (0.29 g, 5.20 mmol) in dry methanol (2.6 mL) to give the *title compound* **9o** (0.20 g, 54%) as a yellow solid. m.p. > 230 °C. δ_H (400 MHz, $(CD_3)_2SO$) 2.69 (3H, s, 6-CH_3), 7.40 (1H, t, J = 7.8 Hz, H-4'), 7.50 (1H, t, J = 7.8 Hz, H-5'), 7.74 (1H, dd, J = 7.8, 1.1 Hz, H-3'), 7.74 (1H, d, J = 15.7 Hz, 5-CO<u>CH</u>CH), 7.92 (1H, d, J = 15.7 Hz, 5-COCHC<u>H</u>), 8.17 (1H, dd, J = 7.8, 1.1 Hz, H-6'), 8.80 (1H, s, H-4), 14.34 (1H, br s, NH). δ_C (100 MHz, $(CD_3)_2SO$) 19.4 (6-CH_3), 113.3 (C-3), 116.6 (CN), 121.2 (C-5), 124.7 (C-2'), 126.4 (5-CO<u>C</u>HCH), 128.3 (C-5'), 128.9 (C-6'), 132.6 (C-4'), 133.2 (C-3'), 140.8 (C-1'), 141.6 (5-COCH<u>C</u>H), 144.2 (C-4), 157.1 (C-6), 179.0 (C-2), 186.3 (5-CO). ν_{max} (ATR)/cm^{-1} 3189 (N-H amine), 2974 (C-H aromatic), 2844 (C-H alkane), 2231 (CN nitrile), 1662 (C=O carbonyl), 1595 (C=C aromatic), 1507 (-C-H bending), 1085 (C-N aromatic), 690 (C-Br). *m/z* (ESI^+): 383 ($^{81}BrMNa^+$, 100%), 381 ($^{79}BrMNa^+$, 98%). HRMS (ESI^+) found ($^{81}BrMNa^+$): 382.9638 $C_{16}H_{11}{}^{81}BrN_2NaOS$ requires 382.9648. Found ($^{79}BrMNa^+$) 380.9661 $C_{16}H_{11}{}^{79}BrN_2NaOS$ requires 380.9668.

(*E*)-5-(3-(3'-Bromophenyl)acryloyl)-6-methyl-2-thioxo-1,2-dihydropyridine-3-carbonitrile 9p

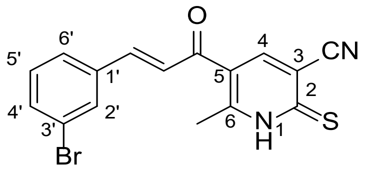

The reaction was carried out following General Procedure **C** using carbonitrile **8** (0.15 g, 0.78 mmol), 3-bromobenzaldehyde **7p** (0.14 g, 0.78 mmol) in dry absolute ethanol (4.8 mL), anhydrous potassium hydroxide (0.22 g, 3.90 mmol) in dry methanol (1.95 mL) to give the *title compound* **9p** (0.15 g, 55%) as a yellow solid. m.p. > 230 °C. δ_H (400 MHz, $(CD_3)_2SO$) 2.65 (3H, s, 6-CH_3), 7.42 (1H, t, *J* = 7.9 Hz, H-5'), 7.62 (1H, d, *J* = 15.9 Hz, 5-COCHC$\underline{H}$), 7.65 (1H, dd, *J* = 7.9, 1.6 Hz, H-4'), 7.74 (1H, d, *J* = 15.9 Hz, 5-COC$\underline{H}$CH), 7.84 (1H, dt, *J* = 7.9, 1.6 Hz, H-6'), 8.15 (1H, t, *J* = 1.6 Hz, H-2'), 8.73 (1H, s, H-4), 14.33 (1H, br s, NH). δ_C (100 MHz, $(CD_3)_2SO$) 19.3 (6-CH_3), 113.3 (C-3), 116.6 (CN), 121.8 (C-5), 122.3 (C-3'), 125.1 (5-CO$\underline{C}$HCH), 128.3 (C-6'), 131.0 (C-5'), 131.5 (C-2'), 133.2 (C-4'), 137.0 (C-1'), 142.9 (5-COCH$\underline{C}$H), 144.2 (C-4), 157.8 (C-6), 179.1 (C-2), 186.8 (5-CO). ν_{max} (ATR)/cm^{-1} 3187 (N-H amine), 2990 (C-H aromatic), 2844 (C-H alkane), 2235 (CN nitrile), 1663 (C=O carbonyl), 1587 (C=C aromatic), 1503 (-C-H bending), 1182 (C-O ether), 1081 (C-N aromatic), 670 (C-Br). *m/z* (ESI^+): 383 ($^{81}BrMNa^+$, 100%), 381 ($^{79}BrMNa^+$, 98%). HRMS (ESI^+) found ($^{81}BrMNa^+$): 382.9645 $C_{16}H_{11}{}^{81}BrN_2NaOS$ requires 382.9648. Found ($^{79}BrMNa^+$) 380.9659 $C_{16}H_{11}{}^{79}BrN_2NaOS$ requires 380.9668.

(*E*)-5-(3-(4'-Bromophenyl)acryloyl)-6-methyl-2-thioxo-1,2-dihydropyridine-3-carbonitrile 9q

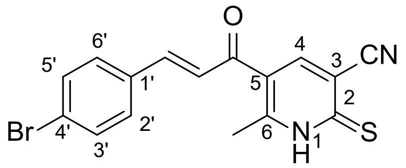

The reaction was carried out following General Procedure **C** using carbonitrile **8** (0.15 g, 0.78 mmol), 4-bromobenzaldehyde **7q** (0.11 g, 0.78 mmol) in dry absolute ethanol (4.8 mL), anhydrous potassium hydroxide (0.22 g, 3.90 mmol) in dry methanol (1.95 mL) to give the *title compound* **9q** (0.17 g, 61%) as a yellow solid. m.p. > 230 °C. δ_H (400 MHz, $(CD_3)_2SO$) 2.65 (3H, s, 6-CH_3), 7.62 (1H, d, *J* = 15.9 Hz, 5-COCHC$\underline{H}$), 7.66 (2H, d, *J* = 8.6 Hz, H-3' and H-

5'), 7.71 (1H, d, *J* = 15.9 Hz, 5-COC$\underline{H}$CH), 7.83 (2H, d, *J* = 8.6 Hz, H-2' and H-6'), 8.72 (1H, s, H-4), 14.32 (1H, br s, NH). δ_C (100 MHz, $(CD_3)_2SO$) 19.2 (6-CH_3), 113.4 (C-3), 116.6 (CN), 121.9 (C-5), 124.2 (C-4'), 124.5 (5-CO$\underline{C}$HCH), 130.9 (C-2' and C-6'), 131.9 (C-3' and C-5'), 133.8 (C-1'), 143.3 (5-COCH$\underline{C}$H), 144.2 (C-4), 157.7 (C-6), 179.1 (C-2), 186.9 (5-CO). ν_{max} (ATR)/cm^{-1} 3096 (N-H amine), 2922 (C-H aromatic), 2854 (C-H alkane), 2239 (CN nitrile), 1659 (C=O carbonyl), 1592 (C=C aromatic), 1509 (-C-H bending), 1083 (C-N aromatic), 694 (C-Br). *m/z* (ESI^+): 383 ($^{81}BrMNa^+$, 100%), 381 ($^{79}BrMNa^+$, 98%). HRMS (ESI^+) found ($^{81}BrMNa^+$): 382.9645 $C_{16}H_{11}{}^{81}BrN_2NaOS$ requires 382.9648. Found ($^{79}BrMNa^+$) 380.9660 $C_{16}H_{11}{}^{79}BrN_2NaOS$ requires 380.9668.

(*E*)-5-(3-(3',4'-Dimethoxyphenyl)acryloyl)-6-methyl-2-thioxo-1,2-dihydropyridine-3-carbonitrile 9r

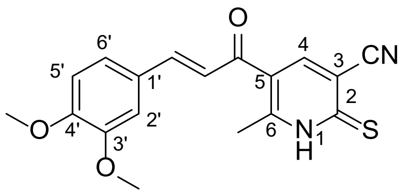

The reaction was carried out following General Procedure **C** using carbonitrile **8** (0.15 g, 0.78 mmol), 3,4-methoxybenzaldehyde **7r** (0.12 mL, 0.78 mmol) in dry absolute ethanol (4.8 mL), anhydrous potassium hydroxide (0.22 g, 3.90 mmol) in dry methanol (1.95 mL) to give the *title compound* **9r** (0.14 g, 50%) as a yellow solid. m.p. > 230 °C. δ_H (400 MHz, $(CD_3)_2SO$) 2.62 (3H, s, 6-CH_3), 3.82 (3H, s, 4'-OCH_3), 3.84 (3H, s, 3'-OCH_3), 7.02 (1H, d, *J* = 8.3 Hz, H-5'), 7.40 (1H, dd, *J* = 8.3, 1.9 Hz, H-6'), 7.46 (1H, d, *J* = 1.9 Hz, H-2'), 7.47 (1H, d, *J* = 15.9 Hz, 5-COC$\underline{H}$CH), 7.61 (1H, d, *J* = 15.9 Hz, 5-COCHC$\underline{H}$), 8.59 (1H, s, H-4), 14.30 (1H, br s, NH). δ_C (100 MHz, $(CD_3)_2SO$) 19.0 (6-CH_3), 55.6 and 55.8 (3'-OCH_3 and 4'-OCH_3), 111.3 (C-2'), 111.6 (C-5'), 113.3 (C-3), 116.7 (CN), 121.6 (5-CO$\underline{C}$HCH), 122.6 (C-5), 124.0 (C-6'), 127.2 (C-1'), 143.9 (C-4), 144.6 (5-COCH$\underline{C}$H), 148.9 (C-3'), 151.5 (C-4'), 156.9 (C-6), 178.8 (C-2), 187.3 (5-CO). ν_{max} (ATR)/cm^{-1} 3170 (N-H amine), 2927 (C-H aromatic), 2850 (C-H alkane), 2228 (CN nitrile), 1652 (C=O carbonyl), 1575 (C=C aromatic), 1509 (-C-H bending), 1184 (C-O ether), 1083 (C-N aromatic). *m/z* (ESI^+): 363 (MNa^+, 100%). HRMS (ESI^+) found (MNa^+): 363.0764 $C_{18}H_{16}N_2NaO_3S$ requires 363.0774.

(*E*)-3-Amino-5-(3-(3'',4''-bis(methoxymethoxy)phenyl)acryloyl)-6-methyl-*N*-phenylthieno[2,3-*b*]pyridine-2-carboxamide 10a

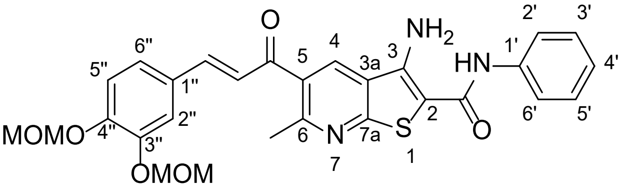

The reaction was carried out following General Procedure **A** using carbonitrile **9a** (0.10 g, 0.25 mmol), chloride **4a** (42 mg, 0.25 mmol) and anhydrous sodium carbonate (52 mg, 0.50 mmol) in absolute ethanol (2.00 mL) for 48 h to give the *title compound* **10a** (83.0 mg, 64%) as a white solid. m.p. 185-187 °C. δ_H (400 MHz, $(CD_3)_2SO$) 2.67 (3H, s, 6-CH_3), 3.40 and 3.42 (6H, s, 2 × MOMCH_3), 5.25 and 5.27 (4H, s, 2 × MOMCH_2), 7.06 (1H, t, J = 7.5 Hz, H-4'), 7.18 (1H, d, J = 8.0 Hz, H-5''), 7.31 (2H, d, J = 7.5 Hz, H-3' and H-5'), 7.36 (2H, d, J = 15.9 Hz, 5-CO$\underline{H}$CH), 7.44 (2H, br s, NH_2), 7.46 (1H, dd, J = 8.0, 1.5 Hz, H-6''), 7.53 (1H, d, J = 15.9 Hz, 5-COCHC$\underline{H}$), 7.57 (1H, d, J = 1.5 Hz, H-2''), 8.77 (1H, s, H-4), 9.48 (1H, br s, NH). δ_C (100 MHz, $(CD_3)_2SO$) 23.7 (6-CH_3), 55.9 (2 × MOMCH_3), 94.4 and 94.8 (2 × MOMCH_2), 116.4 (C-5''), 117.1 (C-2''), 121.2 (C-2' and C-6'), 123.3 (C-4'), 123.6 (C-3a), 124.2 (C-6''), 124.5 (5-CO$\underline{C}$HCH), 124.2 and 124.5 (C-1'', C-3' and C-5'), 130.3 (C-5), 131.0 (C-4), 138.7 (C-1'), 146.0 (5-COCH$\underline{C}$H), 146.7 and 146.8 (C-3 and C-3''), 149.7 (C-4''), 157.2 (C-6), 159.7 (C-7a), 163.6 (2-CONH), 193.9 (5-CO). C-2 not observed. ν_{max} (ATR)/cm^{-1} 3451 (N-H amide), 3325 (N-H amine), 2965 (C-H aromatic), 2844 (C-H alkane), 1664 (C=O carbonyl), 1638 (C=O amide), 1595 (C=C aromatic), 1436 (-C-H bending), 1254 (C-N aromatic), 1149 (C-O ether), 1069 (C-N aliphatic). *m/z* (ESI^+): 556 (MNa^+, 100%), 381 (35%). HRMS (ESI^+) found (MNa^+): 556.1500 $C_{28}H_{27}N_3NaO_6S$ requires 556.1513.

(*E*)-3-Amino-5-(3-(3'',4''-bis(methoxymethoxy)phenyl)acryloyl)-6-methyl-*N*-(*o*-tolyl)thieno[2,3-*b*]pyridine-2-carboxamide 10b

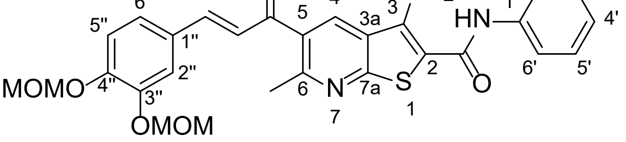

The reaction was carried out following General Procedure **A** using carbonitrile **9a** (0.10 g, 0.25 mmol), chloride **4b** (46.0 mg, 0.25 mmol) and anhydrous sodium carbonate (53.0 mg, 0.50

mmol) in absolute ethanol (3.00 mL) for 48 h to give the *title compound* **10b** (78.0 mg, 56%) as a mustard solid. m.p. decomp. δ_H (400 MHz, $(CD_3)_2SO$) 2.23 (3H, s, 2'-CH_3), 2.67 (3H, s, 6-CH_3), 3.40 and 3.42 (6H, s, 2 × MOMCH_3), 5.25 and 5.27 (4H, s, 2 × MOMCH_2), 7.15-7.20 (3H, m, H-4', H-5' and H-5"), 7.26 (1H, d, *J* = 7.5 Hz, H-3'), 7.32-7.37 (4H, m, H-6', 5-COC<u>H</u>CH and NH_2), 7.45 (1H, dd, *J* = 7.5, 2.1 Hz, H-6"), 7.52 (1H, d, *J* = 15.9 Hz, 5-COCHC<u>H</u>), 7.57 (1H, d, *J* = 2.1 Hz, H-2"), 8.76 (1H, s, H-4), 9.18 (1H, br s, NH). δ_C (100 MHz, $(CD_3)_2SO$) 18.0 (2'-CH_3), 23.7 (6-CH_3), 55.9 (2 × MOMCH_3), 94.4 and 94.8 (2 × MOMCH_2), 116.4 (C-5"), 117.1 (C-2"), 124.7 (C-3a), 124.2 (C-6"), 124.6 (5-CO<u>C</u>HCH), 125.9 (C-4' and C-5'), 126.8 (C-6'), 128.4 (C-1"), 130.1 and 130.2 (C-3' and C-5), 131.0 (C-4), 133.9 (C-2'), 137.1 (C-1'), 146.0 (5-COCH<u>C</u>H), 146.7 (C-3 and C-3"), 149.6 (C-4"), 157.3 (C-6), 159.3 (C-7a), 163.6 (2-CONH), 193.6 (5-CO). C-2 not observed. ν_{max} (ATR)/cm^{-1} 3427 (N-H amide), 3332 (N-H amine), 2917 (C-H aromatic), 2856 (C-H alkane), 1665 (C=O carbonyl), 1643 (C=O amide), 1584 (C=C aromatic), 1452 (-C-H bending), 1254 (C-N aromatic), 1149 (C-O ether), 1066 (C-N aliphatic). *m/z* (ESI^+): 570 (MNa^+, 100%), 381 (15%). HRMS (ESI^+) found (MNa^+): 570.1671 $C_{29}H_{29}N_3NaO_6S$ requires 570.1669.

(*E*)-3-Amino-5-(3-(3'',4''-bis(methoxymethoxy)phenyl)acryloyl)-*N*-(3'-chloro-2'-methylphenyl)-6-methylthieno[2,3-*b*]pyridine-2-carboxamide 10c

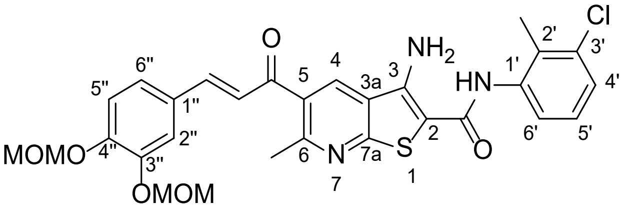

The reaction was carried out following General Procedure **A** using carbonitrile **9a** (0.15 g, 0.48 mmol), chloride **4c** (0.10 g, 0.25 mmol) and anhydrous sodium carbonate (0.10 g, 0.50 mmol) in absolute ethanol (3.00 mL) for 48 h to give the *title compound* **10c** (0.13 g, 83%) as a yellow solid. m.p. 156-158 °C. δ_H (400 MHz, $(CD_3)_2SO$) 2.25 (3H, s, 2'-CH_3), 2.67 (3H, s, 6-CH_3), 3.40 and 3.42 (6H, s, 2 × MOMCH_3), 5.25 and 5.27 (4H, s, 2 × MOMCH_2), 7.17 (1H, d, *J* = 8.5 Hz, H-5"), 7.22 (1H, d, *J* = 8.0 Hz, H-5'), 7.29-7.37 (5H, m, H-4' and H-6', 5-COC<u>H</u>CH and NH_2), 7.45 (1H, dd, *J* = 8.5, 2.1 Hz, H-6"), 7.52 (1H, d, *J* = 15.9 Hz, 5-COCHC<u>H</u>), 7.57 (1H, d, *J* = 2.1 Hz, H-2"), 8.75 (1H, s, H-4), 9.43 (1H, br s, NH). δ_C (100 MHz, $(CD_3)_2SO$) 15.4 (2'-CH_3), 23.7 (6-CH_3), 55.9 (2 × MOMCH_3), 94.4 and 94.8 (2 × MOMCH_2), 116.4 (C-5"), 117.1 (C-2"), 123.7 (C-3a), 124.2 (C-6"), 124.6 (5-CO<u>C</u>HCH), 124.7 (C-4' or C-6'), 125.9 (C-

4' or C-6'), 126.6 (C-5'), 128.4 (C-1"), 130.2 (C-5), 131.0 (C-4), 132.2 (C-2'), 133.6 (C-3'), 139.2 (C-1'), 146.0 (C-3 and 5-COCHC̲H), 146.7 (C-3"), 149.6 (C-4"), 157.2 (C-6), 159.4 (C-7a), 164.0 (2-CONH), 193.6 (5-CO). C-2 not observed. ν_{max} (ATR)/cm^{-1} 3413 (N-H amide), 3319 (N-H amine), 2896 (C-H alkane), 1717 (C=O carbonyl), 1651 (C=O amide), 1577 (C=C aromatic), 1429 (-C-H bending), 1252 (C-N aromatic), 1167 (C-O ether), 1077 (C-N aliphatic), 752 (C-Cl). *m/z* (ESI$^+$): 606 (^{37}ClMNa$^+$, 40%), 604 (^{35}ClMNa$^+$, 100%), 252 (20%). HRMS (ESI$^+$) found (^{37}ClMNa$^+$): 606.1255 $C_{29}H_{28}{}^{37}ClN_3NaO_6S$ requires 606.1262. Found (^{35}ClMNa$^+$): 604.1280 $C_{29}H_{28}{}^{35}ClN_3NaO_6S$ requires 604.1280.

(*E*)-3-Amino-5-(3-(3'',4''-bis(methoxymethoxy)phenyl)acryloyl)-6-methyl-*N*-(naphthalen-1'-yl)thieno[2,3-*b*]pyridine-2-carboxamide 10d

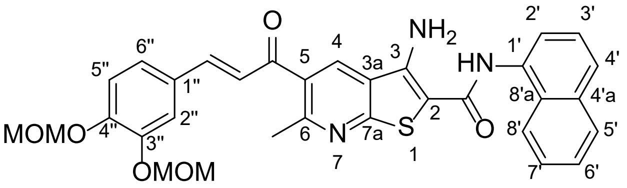

The reaction was carried out following General Procedure **A** using carbonitrile **9a** (0.10 g, 0.25 mmol), chloride **4d** (55.0 mg, 0.25 mmol) and anhydrous sodium carbonate (53.0 mg, 0.50 mmol) in absolute ethanol (3.00 mL) for 48 h to give the *title compound* **10d** (0.13 g, 87%) as a brown solid. m.p. decomp. δ_H (400 MHz, $(CD_3)_2SO$) 2.69 (3H, s, 6-CH_3), 3.41 and 3.42 (6H, s, 2 × MOMCH_3), 5.26 and 5.27 (4H, s, 2 × MOMCH_2), 7.18 (1H, d, J = 8.5 Hz, H-5"), 7.36-7.39 (3H, m, 5-COCH̲CH and NH_2), 7.46 (1H, dd, J = 8.5, 1.9 Hz, H-6"), 7.51-7.58 (6H, m, H-2", 5-COCHCH̲ and 4 × Ar-CH), 7.82-7.84 (1H, m, Ar-CH), 7.95-7.97 (2H, m, 2 × Ar-CH), 8.78 (1H, s, H-4), 9.76 (1H, br s, NH). δ_C (100 MHz, $(CD_3)_2SO$) 23.8 (6-CH_3), 55.9 (2 × MOMCH_3), 94.4 and 94.8 (2 × MOMCH_2), 116.4 (C-5"), 117.1 (C-2"), 123.5 (Ar-CH), 123.7 (C-3a), 124.2 (Ar-CH), 124.5 (Ar-CH and C-6"), 124.8 (5-COC̲HCH), 125.5 (Ar-CH), 125.9 (Ar-CH), 126.3 (Ar-CH), 127.9 (Ar-CH), 128.4 (C-1"), 129.7 (Ar-C), 130.2 (C-5), 131.0 (C-4), 133.7 (Ar-C and C-1'), 146.0 (5-COCHC̲H), 146.7 (C-3 and C-3"), 149.6 (C-4"), 157.2 (C-6), 159.5 (C-7a), 164.7 (2-CONH), 193.5 (5-CO). C-2 not observed. ν_{max} (ATR)/cm^{-1} 3420 (N-H amide), 3325 (N-H amine), 2925 (C-H aromatic), 2840 (C-H alkane), 1717 (C=O carbonyl), 1631 (C=O amide), 1589 (C=C aromatic), 1436 (-C-H bending), 1255 (C-N aromatic), 1152 (C-O ether), 1069 (C-N aliphatic). *m/z* (ESI$^+$): 606 (MNa$^+$, 100%). HRMS (ESI$^+$) found (MNa$^+$): 606.1668 $C_{32}H_{29}N_3NaO_6S$ requires 606.1669.

(E)-3-Amino-5-(3-(3'',4''-bis(methoxymethoxy)phenyl)acryloyl)-N-(4'-methoxyphenyl)-6-methylthieno[2,3-b]pyridine-2-carboxamide 10e

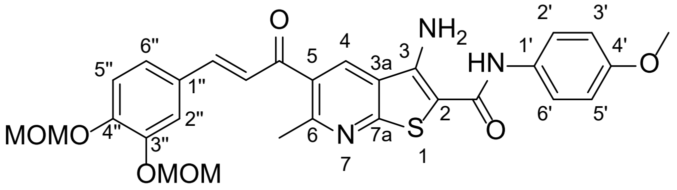

The reaction was carried out following General Procedure **A** using carbonitrile **9a** (0.10 g, 0.25 mmol), chloride **4e** (50.0 mg, 0.25 mmol) and anhydrous sodium carbonate (53.0 mg, 0.50 mmol) in absolute ethanol (3.00 mL) for 48 h to give the *title compound* **10e** (0.14 g, quant.) as a white solid. m.p. 183-185 °C. δ_H (400 MHz, $(CD_3)_2SO$) 2.67 (3H, s, 6-CH_3), 3.40 and 3.42 (6H, s, 2 × MOMCH_3), 3.74 (3H, s, 4'-OCH_3), 5.25 and 5.26 (4H, s, 2 × MOMCH_2), 6.90 (2H, d, J = 8.7 Hz, H-3' and H-5'), 7.17 (1H, d, J = 8.7 Hz, H-5''), 7.34-7.46 (4H, m, H-6'', 5-CO<u>CH</u>CH and NH_2), 7.50-7.59 (4H, m, H-2'', H-2', H-6' and 5-COCHC<u>H</u>), 8.78 (1H, s, H-4), 9.37 (1H, br s, NH). δ_C (100 MHz, $(CD_3)_2SO$) 23.8 (6-CH_3), 55.2 (4'-OCH_3), 55.9 (2 × MOMCH_3), 94.5 and 94.9 (2 × MOMCH_2), 96.7 (C-2), 113.6 (C-3' and C-5'), 116.5 (C-5''), 117.2 (C-2''), 123.0 (C-2' and C-6'), 123.6 (C-3a), 124.3 (C-6''), 124.6 (5-CO<u>C</u>HCH), 128.5 (C-1''), 130.3 (C-5), 131.1 (C-4), 131.9 (C-1'), 146.0 (5-COCH<u>C</u>H), 146.7 and 146.8 (C-3 and C-3''), 149.7 (C-4''), 155.6 (C-4'), 157.5 (C-6), 159.4 (C-7a), 163.6 (2-CONH), 193.5 (5-CO). ν_{max} (ATR)/cm^{-1} 3444 (N-H amide), 3325 (N-H amine), 2996 (C-H aromatic), 2830 (C-H alkane), 1665 (C=O carbonyl), 1633 (C=O amide), 1590 (C=C aromatic), 1504 (-C-H bending), 1246 (C-N aromatic), 1149 (C-O ether), 1061 (C-N aliphatic). m/z (ESI^+): 586 (MNa^+, 100%). HRMS (ESI^+) found (MNa^+): 586.1622 $C_{29}H_{29}N_3NaO_7S$ requires 586.1618.

(E)-3-Amino-5-(3-(4''-methoxyphenyl)acryloyl)-6-methyl-N-phenylthieno[2,3-b]pyridine-2-carboxamide 11a

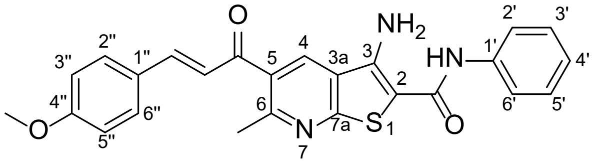

The reaction was carried out following General Procedure **A** using carbonitrile **9b** (40.0 mg, 0.13 mmol), chloride **4a** (22.0 mg, 0.13 mmol) and anhydrous sodium carbonate (28.0 mg, 0.26 mmol) in absolute ethanol (2.00 mL) for 48 h to give the *title compound* **11a** (26.0 mg, 45%)

as a brown solid. m.p. 220-222 °C. δ_H (400 MHz, $(CD_3)_2SO$) 2.69 (3H, s, 6-CH_3), 3.82 (3H, s, 4''-OCH_3), 7.03 (2H, d, J = 8.7 Hz, H-3'' and H-5''), 7.08 (1H, t, J = 7.5 Hz, H-4'), 7.32 (2H, t, J = 7.5 Hz, H-3' and H-5'), 7.40 (1H, d, J = 15.9 Hz, 5-COC$\underline{H}$CH), 7.46 (2H, br s, NH_2), 7.60 (1H, d, J = 15.9 Hz, 5-COCHC$\underline{H}$), 7.70 (2H, d, J = 7.5 Hz, H-2' and H-6'), 7.80 (2H, d, J = 8.7 Hz, H-2'' and H-6''), 8.83 (1H, s, H-4), 9.48 (1H, br s, NH). δ_C (100 MHz, $(CD_3)_2SO$) 23.9 (6-CH_3), 55.4 (4''-OCH_3), 114.5 (C-3'' and C-5''), 121.1 (C-2' and C-6'), 123.3 (5-CO$\underline{C}$HCH and C-4'), 123.6 (C-3a), 126.9 (C-1''), 128.4 (C-3' and C-5'), 130.2 (C-5), 130.8 (C-2'' and C-6''), 131.2 (C-4), 139.1 (C-1'), 145.8 (5-COCH$\underline{C}$H), 147.0 (C-3), 157.7 (C-6), 159.4 (C-7a), 161.6 (C-4''), 163.8 (2-CONH), 193.0 (5-CO). C-2 not observed. ν_{max} (ATR)/cm^{-1} 3406 (N-H amide), 3278 (N-H amine), 3053 (C-H aromatic), 2923 (C-H alkane), 1737 (C=O carbonyl), 1586 (C=O amide), 1509 (C=C aromatic), 1436 (-C-H bending), 1246 (C-N aromatic), 1171 (C-O ether), 1069 (C-N aliphatic). *m/z* (ESI^+): 466 (MNa^+, 100%), 227 (20%), 159 (10%), 101 (20%). HRMS (ESI^+) found (MNa^+): 466.1181 $C_{25}H_{21}N_3NaO_3S$ requires 466.1196.

(*E*)-3-Amino-5-(3-(4''-methoxyphenyl)acryloyl)-6-methyl-*N*-(*o*-tolyl)thieno[2,3-*b*]pyridine-2-carboxamide 11b

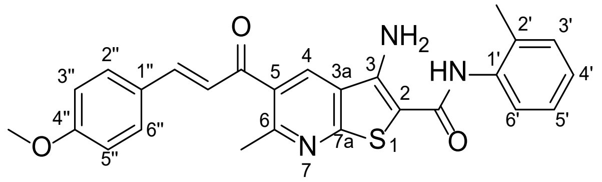

The reaction was carried out following General Procedure **A** using carbonitrile **9b** (0.10 g, 0.32 mmol), chloride **4b** (59.0 mg, 0.32 mmol) and anhydrous sodium carbonate (68.0 mg, 0.64 mmol) in absolute ethanol (3.00 mL) for 72 h to give the *title compound* **11b** (62.0 mg, 41%) as a mustard yellow solid. m.p. decomp. δ_H (400 MHz, $(CD_3)_2SO$) 2.24 (3H, s, 2'-CH_3), 2.69 (3H, s, 6-CH_3), 3.82 (3H, s, 4''-OCH_3), 7.03 (2H, d, J = 8.7 Hz, H-3'' and H-5''), 7.14 (1H, t, J = 7.2 Hz, H-4'), 7.20 (1H, t, J = 7.2 Hz, H-5'), 7.26 (1H, d J = 7.2 Hz, H-3'), 7.34 (1H, d, J = 7.2 Hz, H-6'), 7.36 (2H, br s, NH_2), 7.39 (1H, d, J = 15.9 Hz, 5-COC$\underline{H}$CH), 7.59 (1H, d, J = 15.9 Hz, 5-COCHC$\underline{H}$), 7.79 (2H, d, J = 8.7 Hz, H-2'' and H-6''), 8.81 (1H, s, H-4), 9.25 (1H, br s, NH). δ_C (100 MHz, $(CD_3)_2SO$) 18.0 (2'-CH_3), 23.9 (6-CH_3), 55.4 (4''-OCH_3), 114.5 (C-3'' and C-5''), 123.3 (5-CO$\underline{C}$HCH), 123.7 (C-3a), 125.7 and 125.9 (C-4' and C-5'), 126.8 (C-6'), 126.9 (C-1''), 130.1 and 130.2 (C-3' and C-5), 130.8 (C-2'' and C-6''), 131.1 (C-4), 133.9 (C-2'), 136.6 (C-1'), 145.8 (5-COCH$\underline{C}$H), 146.3 (C-3), 157.5 (C-6), 159.3 (C-7a), 161.6 (C-4''), 163.7 (2-CONH), 193.0 (5-CO). C-2 not observed. ν_{max} (ATR)/cm^{-1} 3418 (N-H amide), 3314

(N-H amine), 2970 (C-H aromatic), 2835 (C-H alkane), 1738 (C=O carbonyl), 1585 (C=O amide), 1509 (C=C aromatic), 1424 (-C-H bending), 1252 (C-N aromatic), 1171 (C-O ether), 1063 (C-N aliphatic). *m/z* (ESI^+): 480 (MNa^+, 100%). HRMS (ESI^+) found (MNa^+): 480.1339 $C_{26}H_{23}N_3NaO_3S$ requires 480.1352.

(*E*)-3-Amino-*N*-(3'-chloro-2'-methylphenyl)-5-(3-(4''-methoxyphenyl)acryloyl)-6-methylthieno[2,3-*b*]pyridine-2-carboxamide 11c

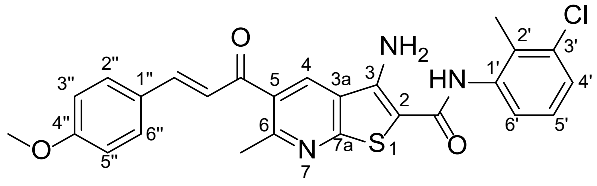

The reaction was carried out following General Procedure **A** using carbonitrile **9b** (0.10 g, 0.32 mmol), chloride **4c** (70.0 mg, 0.32 mmol) and anhydrous sodium carbonate (68.0 mg, 0.64 mmol) in absolute ethanol (3.00 mL) for 72 h to give the *title compound* **11c** (75.0 mg, 47%) as a yellow solid. m.p. 207-209 °C. δ_H (400 MHz, $(CD_3)_2SO$) 2.24 (3H, s, 2'-CH_3), 2.70 (3H, s, 6-CH_3), 3.82 (3H, s, 4''-OCH_3), 7.02 (2H, d, J = 8.6 Hz, H-3'' and H-5''), 7.23 (1H, t, J = 7.5 Hz, H-5'), 7.30 (1H, d, J = 7.5 Hz, H-6'), 7.34 (1H, d, J = 7.5 Hz, H-4'), 7.46-7.50 (3H, m, 5-CO<u>CH</u>CH and NH_2), 7.60 (1H, d, J = 15.8 Hz, 5-COCHC<u>H</u>), 7.83 (2H, d, J = 8.6 Hz, H-2'' and H-6''), 8.98 (1H, s, H-4), 9.49 (1H, br s, NH). δ_C (100 MHz, $(CD_3)_2SO$) 15.5 (2'-CH_2), 24.0 (6-CH_3), 55.4 (4''-OCH_3), 114.5 (C-3'' and C-5''), 123.2 (5-CO<u>C</u>HCH), 123.7 (C-3a), 126.2 (C-6'), 126.5 (C-1''), 126.7 (C-4'), 127.0 (C-5'), 130.0 (C-5), 130.9 (C-2'' and C-6''), 131.6 (C-4), 132.5 (C-2'), 133.6 (C-3'), 138.2 (C-1'), 145.7 (5-COCH<u>C</u>H), 147.0 (C-3), 157.8 (C-6), 159.5 (C-7a), 161.6 (C-4''), 163.9 (2-CONH), 192.7 (5-CO). C-2 not observed. ν_{max} (ATR)/cm^{-1} 3429 (N-H amide), 3311 (N-H amine), 2971 (C-H aromatic), 2894 (C-H alkane), 1739 (C=O carbonyl), 1656 (C=O amide), 1508 (C=C aromatic), 1425 (-C-H bending), 1260 (C-N aromatic), 1167 (C-O ether), 1062 (C-N aliphatic). *m/z* (ESI^+): 516 ($^{37}ClMNa^+$, 40%), 514 ($^{35}ClMNa^+$, 100%). HRMS (ESI^+) found ($^{37}ClMNa^+$): 516.0923 $C_{26}H_{22}{}^{37}ClN_3NaO_3S$ requires 516.0942. Found ($^{35}ClMNa^+$): 514.0946 $C_{26}H_{22}{}^{35}ClN_3NaO_3S$ requires 514.0963.

(*E*)-3-Amino-5-(3-(4''-methoxyphenyl)acryloyl)-6-methyl-*N*-(naphthalen-1'-yl)thieno[2,3-*b*]pyridine-2-carboxamide 11d

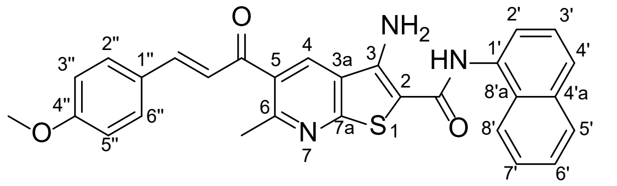

The reaction was carried out following General Procedure **A** using carbonitrile **9b** (50.0 mg, 0.16 mmol), chloride **4d** (35.0 mg, 0.16 mmol) and anhydrous sodium carbonate (34.0 mg, 0.32 mmol) in absolute ethanol (2.00 mL) for 48 h to give the *title compound* **11d** (45.0 mg, 57%) as a brown solid. m.p. > 230 °C. δ_H (400 MHz, $(CD_3)_2SO$) 2.71 (3H, s, 6-CH_3), 3.82 (3H, s, 4''-OCH_3), 7.03 (2H, d, *J* = 8.7 Hz, H-3'' and H-5''), 7.43-7.47 (3H, m, 5-COCHCH and NH_2), 7.53-7.55 (4H, m, 4 × Ar-CH), 7.61 (1H, d, *J* = 15.9 Hz, 5-COCHCH), 7.81-7.84 (3H, m, H-2'', H-6'' and Ar-CH), 7.94-7.98 (2H, m, 2 × Ar-CH), 8.92 (1H, s, H-4), 9.77 (1H, br s, NH). δ_C (100 MHz, $(CD_3)_2SO$) 24.0 (6-CH_3), 55.4 (4''-OCH_3), 114.5 (C-3'' and C-5''), 123.3 (5-COCHCH), 123.5 (Ar-CH), 123.7 (C-3a), 124.3 (Ar-CH), 125.5 (Ar-CH), 126.0 (2 × Ar-CH), 126.2 (Ar-CH), 127.0 (C-1''), 128.0 (Ar-CH), 129.7 (C-5), 130.1 (Ar-C), 130.9 (C-2'' and C-6''), 131.4 (C-4), 133.7 (Ar-C), 135.0 (C-1'), 145.7 (5-COCHCH), 147.5 (C-3), 157.7 (C-6), 159.7 (C-7a), 161.6 (C-4''), 164.7 (2-CONH), 192.9 (5-CO). C-2 not observed. ν_{max} (ATR)/cm^{-1} 3425 (N-H amide), 3285 (N-H amine), 2971 (C-H aromatic), 2855 (C-H alkane), 1740 (C=O carbonyl), 1592 (C=O amide), 1508 (C=C aromatic), 1429 (-C-H bending), 1255 (C-N aromatic), 1172 (C-O ether), 1069 (C-N aliphatic), 864 (C-S). *m/z* (ESI$^+$): 516 (MNa$^+$, 100%), 101 (25%). HRMS (ESI$^+$) found (MNa$^+$): 516.1343 $C_{29}H_{23}N_3NaO_3S$ requires 516.1352.

(*E*)-3-Amino-*N*-(4'-methoxyphenyl)-5-(3-(4''-methoxyphenyl)acryloyl)-6-methylthieno[2,3-*b*]pyridine-2-carboxamide 11e

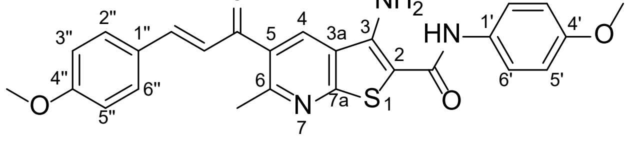

The reaction was carried out following General Procedure **A** using carbonitrile **9b** (50.0 mg, 0.16 mmol), chloride **4e** (32.0 mg, 0.16 mmol) and anhydrous sodium carbonate (34.0 mg, 0.32

mmol) in absolute ethanol (2.00 mL) for 48 h to give the *title compound* **11e** (45.0 mg, 59%) as a brown solid. m.p. > 230 °C. δ_H (400 MHz, $(CD_3)_2SO$) 2.69 (3H, s, 6-CH_3), 3.75 (3H, s, 4'-OCH_3), 3.82 (3H, s, 4''-OCH_3), 6.90 (2H, d, J = 8.7 Hz, H-3' and H-5'), 7.03 (2H, d, J = 8.7 Hz, H-3'' and H-5''), 7.37-7.41 (3H, m, 5-COC<u>H</u>CH and NH_2), 7.57-7.61 (3H, m, H-2', H-6' and 5-COCHC<u>H</u>), 7.80 (2H, d, J = 8.7 Hz, H-2'' and H-6''), 8.81 (1H, s, H-4), 9.37 (1H, br s, NH). δ_C (100 MHz, $(CD_3)_2SO$) 23.9 (6-CH_3), 55.1 (4'-OCH_3), 55.4 (4''-OCH_3), 113.6 (C-3' and C-5'), 114.5 (C-3'' and C-5''), 122.9 (C-2' and C-6'), 123.3 (5-CO<u>C</u>HCH), 124.0 (C-3a), 127.0 (C-1''), 130.2 (C-5), 130.8 (C-2'' and C-6''), 131.1 (C-4), 131.8 (C-1'), 145.8 (5-COCH<u>C</u>H and C-3), 155.5 (C-4'), 157.7 (C-6), 159.3 (C-7a), 161.6 (C-4''), 163.5 (2-CONH), 193.0 (5-CO). C-2 not observed. ν_{max} (ATR)/cm^{-1} 3425 (N-H amide), 3321 (N-H amine), 2954 (C-H aromatic), 2836 (C-H alkane), 1746 (C=O carbonyl), 1591 (C=O amide), 1508 (C=C aromatic), 1439 (-C-H bending), 1240 (C-N aromatic), 1171 (C-O ether), 1028 (C-N aliphatic). *m/z* (ESI^+): 496 (MNa^+, 100%), 227 (40%), 159 (20%), 101 (25%). HRMS (ESI^+) found (MNa^+): 496.1289 $C_{26}H_{23}N_3NaO_4S$ requires 496.1301.

(*E*)-3-Amino-6-methyl-5-(3-(4''-morpholinophenyl)acryloyl)-*N*-phenylthieno[2,3-*b*]pyridine-2-carboxamide 12a

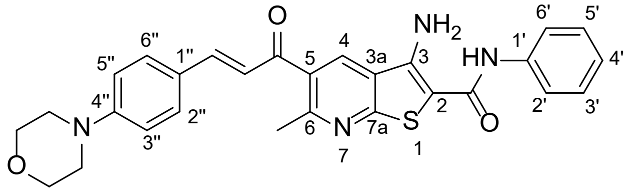

The reaction was carried out following General Procedure **A** using carbonitrile **9c** (40.0 mg, 0.11 mmol), chloride **4a** (18.0 mg, 0.11 mmol) and anhydrous sodium carbonate (23.0 mg, 0.22 mmol) in absolute ethanol (2.00 mL) for 48 h to give the *title compound* **12a** (44.0 mg, 80%) as a yellow solid. m.p. > 230 °C. δ_H (400 MHz, $(CD_3)_2SO$) 2.68 (3H, s, 6-CH_3), 3.26 (4H, t, J = 4.7 Hz, 2 × *N*-CH_2), 3.73 (4H, t, J = 4.7 Hz, 2 × O-CH_2), 6.99 (2H, d, J = 8.9 Hz, H-3'' and H-5''), 7.07 (1H, t, J = 7.4 Hz, H-4'), 7.27-7.34 (3H, m, 5-COC<u>H</u>CH, H-3' and H-5'), 7.46 (2H, br s, NH_2), 7.54 (1H, d, J = 15.8 Hz, 5-COCHC<u>H</u>), 7.69 (4H, t, J = 8.9 Hz, H-2' and H-6', H-2'' and H-6''), 8.80 (1H, s, H-4), 9.48 (1H, br s, NH). δ_C (100 MHz, $(CD_3)_2SO$) 23.8 (6-CH_3), 47.0 (2 × *N*-CH_2), 65.8 (2 × *O*-CH_2), 96.4 (C-2), 114.0 (C-3'' and C-5''), 121.1 (C-2' and C-6'), 121.5 (5-CO<u>C</u>HCH), 123.4 (C-4'), 123.5 (C-3a), 124.2 (C-1''), 128.4 (C-3' and C-5'), 130.6 (C-5, C-2'' and C-6''), 131.0 (C-4), 139.0 (C-1'), 146.4 (5-COCH<u>C</u>H), 147.1 (C-3), 152.8 (C-4''), 157.6 (C-6), 159.2 (C-7a), 163.8 (2-CONH), 192.9 (5-CO). ν_{max} (ATR)/cm^{-1} 3439 (N-H

amide), 3324 (N-H amine), 3039 (C-H aromatic), 2849 (C-H alkane), 1739 (C=O carbonyl), 1637 (C=O amide), 1582 (C=C aromatic), 1437 (-C-H bending), 1254 (C-N aromatic), 1187 (C-O ether), 1067 (C-N aliphatic). *m/z* (ESI^+): 521 (MNa^+, 100%). HRMS (ESI^+) found (MNa^+): 521.1608 $C_{28}H_{26}N_4NaO_3S$ requires 521.1618.

(*E*)-3-Amino-6-methyl-5-(3-(4''-morpholinophenyl)acryloyl)-*N*-(*o*-tolyl)thieno[2,3-*b*]pyridine-2-carboxamide 12b

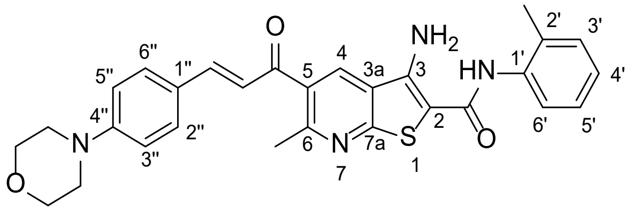

The reaction was carried out following General Procedure **A** using carbonitrile **9c** (60.0 mg, 0.16 mmol), chloride **4b** (30.0 mg, 0.16 mmol) and anhydrous sodium carbonate (35.0 mg, 0.33 mmol) in absolute ethanol (3.00 mL) for 48 h to give the *title compound* **12b** (31.0 mg, 37%) as a yellow solid. m.p. 212-214 °C. δ_H (400 MHz, $(CD_3)_2SO$) 2.24 (3H, s, 2'-CH_3), 2.68 (3H, s, 6-CH_3), 3.26 (4H, t, J = 4.7 Hz, 2 × *N*-CH_2), 3.73 (4H, t, J = 4.7 Hz, 2 × *O*-CH_2), 7.00 (2H, d, J = 8.8 Hz, H-3'' and H-5''), 7.15 (1H, t, J = 7.5 Hz, H-4'), 7.20 (1H, t, J = 7.5 Hz, H-5'), 7.25-7.33 (5H, m, H-3', H-6', 5-COC<u>H</u>CH and NH_2), 7.53 (1H, d, J = 15.9 Hz, 5-COCHC<u>H</u>), 7.68 (2H, d, J = 8.8 Hz, H-2'' and H-6''), 8.77 (1H, s, H-4), 9.20 (1H, br s, NH). δ_C (100 MHz, $(CD_3)_2SO$) 18.0 (2'-CH_3), 23.8 (6-CH_3), 47.0 (2 × *N*-CH_2), 65.8 (2 × *O*-CH_2), 114.0 (C-3'' and C-5''), 121.6 (5-CO<u>C</u>HCH), 123.7 (C-3a), 124.2 (C-1''), 125.6 and 125.9 (C-4' and C-5'), 126.8 (C-6'), 130.1 (C-3'), 130.6 (C-5, C-2'' and C-6''), 130.8 (C-4), 133.9 (C-2'), 137.1 (C-1'), 146.4 (C-3 and 5-COCH<u>C</u>H), 152.8 (C-4''), 157.3 (C-6), 159.2 (C-7a), 163.8 (2-CONH), 193.0 (5-CO). C-2 not observed. ν_{max} (ATR)/cm^{-1} 3454 (N-H amide), 3344 (N-H amine), 2968 (C-H aromatic), 2853 (C-H alkane), 1653 (C=O carbonyl), 1618 (C=O amide), 1587 (C=C aromatic), 1488 (-C-H bending), 1267 (C-N aromatic), 1183 (C-O ether), 1066 (C-N aliphatic). *m/z* (ESI^+): 535 (MNa^+, 100%). HRMS (ESI^+) found (MNa^+): 535.1783 $C_{29}H_{28}N_4NaO_3S$ requires 535.1774.

(*E*)-3-Amino-*N*-(3'-chloro-2'-methylphenyl)-6-methyl-5-(3-(4''-morpholinophenyl)acryloyl)thieno[2,3-*b*]pyridine-2-carboxamide 12c

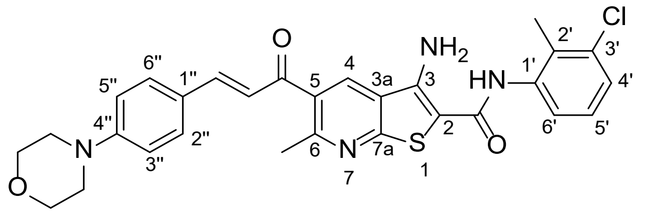

The reaction was carried out following General Procedure **A** using carbonitrile **9c** (72.0 mg, 0.20 mmol), chloride **4c** (43.0 mg, 0.20 mmol) and anhydrous sodium carbonate (42.0 mg, 0.40 mmol) in absolute ethanol (3.00 mL) for 72 h to give the *title compound* **12c** (57.0 mg, 52%) as a yellow solid. m.p. > 230 °C. δ_H (400 MHz, $(CD_3)_2SO$) 2.25 (3H, s, 2'-CH_3), 2.68 (3H, s, 6-CH_3), 3.26 (4H, t, *J* = 4.7 Hz, 2 × *N*-CH_2), 3.73 (4H, t, *J* = 4.7 Hz, 2 × *O*-CH_2), 6.99 (2H, d, *J* = 8.7 Hz, H-3'' and H-5''), 7.22 (1H, t, *J* = 7.8 Hz, H-5'), 7.28-7.32 (3H, m, 5-COC$\underline{H}$CH, H-4' and H-6'), 7.33 (2H, br s, NH_2), 7.52 (1H, d, *J* = 15.8 Hz, 5-COCHC$\underline{H}$), 7.68 (2H, d, *J* = 8.7 Hz, H-2'' and H-6''), 8.77 (1H, s, H-4), 9.44 (1H, br s, NH). δ_C (100 MHz, $(CD_3)_2SO$) 15.5 (2'-CH_3), 23.8 (6-CH_3), 47.0 (2 × *N*-CH_2), 65.9 (2 × *O*-CH_2), 96.7 (C-2), 114.1 (C-3'' and C-5''), 121.6 (C-4' or C-6' and 5-CO$\underline{C}$HCH), 123.7 (C-3a), 124.2 (C-1''), 126.0 (C-4' or C-6'), 126.7 (C-5'), 130.9 (C-5, C-2'' and C-6''), 130.9 (C-4), 132.3 (C-2'), 133.6 (C-3'), 139.2 (C-1'), 146.4 (C-3 and 5-COCH$\underline{C}$H), 152.8 (C-4''), 157.4 (C-6), 159.2 (C-7a), 164.0 (2-CONH), 193.0 (5-CO). ν_{max} (ATR)/cm^{-1} 3454 (N-H amide), 3311 (N-H amine), 2971 (C-H aromatic), 1739 (C=O carbonyl), 1650 (C=O amide), 1589 (C=C aromatic), 1433 (-C-H bending), 1293 (C-N aromatic), 1127 (C-O ether), 1070 (C-N aliphatic), 761 (C-Cl). *m/z* (ESI^+): 571 ($^{37}ClMNa^+$, 40%), 569 ($^{35}ClMNa^+$, 100%), 252 (20%). HRMS (ESI^+) found ($^{37}ClMNa^+$): 571.1353 $C_{29}H_{27}{}^{37}ClN_4NaO_3S$ requires 571.1366. Found ($^{35}ClMNa^+$): 569.1369 $C_{29}H_{27}{}^{35}ClN_4NaO_3S$ requires 569.1385.

(*E*)-3-Amino-6-methyl-5-(3-(4''-morpholinophenyl)acryloyl)-*N*-(naphthalen-1'-yl)thieno[2,3-*b*]pyridine-2-carboxamide 12d

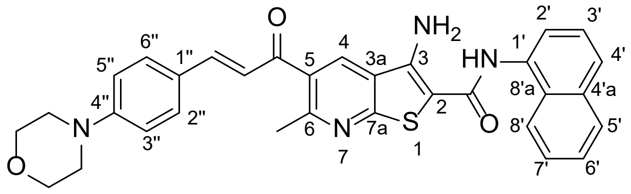

The reaction was carried out following General Procedure **A** using carbonitrile **9c** (56.0 mg, 0.15 mmol), chloride **4d** (34.0 mg, 0.15 mmol) and anhydrous sodium carbonate (32.0 mg, 0.31 mmol) in absolute ethanol (2.00 mL) for 48 h to give the *title compound* **12d** (79.0 mg, 94%) as an orange solid. m.p. > 230 °C. δ_H (400 MHz, $(CD_3)_2SO$) 2.70 (3H, s, 6-CH_3), 3.26 (4H, t, J = 4.7 Hz, 2 × *N*-CH_2), 3.73 (4H, t, J = 4.7 Hz, 2 × *O*-CH_2), 7.00 (2H, d, J = 8.8 Hz, H-3'' and H-5''), 7.29-7.33 (3H, m, 5-COC<u>H</u>CH and NH_2), 7.49-7.57 (4H, m, 3 × Ar-CH and 5-COCHC<u>H</u>), 7.62-7.64 (1H, m, Ar-CH), 7.69 (2H, d, J = 8.8 Hz, H-2'' and H-6''), 7.76-7.79 (1H, m, Ar-CH), 7.93-7.95 (1H, m, Ar-CH), 8.01-8.04 (1H, m, Ar-CH), 8.76 (1H, s, H-4), 9.74 (1H, br s, NH). δ_C (100 MHz, $(CD_3)_2SO$) 23.8 (6-CH_3), 47.0 (2 × *N*-CH_2), 65.8 (2 × *O*-CH_2), 96.8 (C-2), 114.1 (C-3'' and C-5''), 121.6 (5-CO<u>C</u>HCH), 123.6 (Ar-CH), 123.9 (C-3a), 124.2 (C-1''), 124.4 (Ar-CH), 125.5 (Ar-CH), 125.8 (Ar-CH), 125.9 (Ar-CH), 126.3 (Ar-CH), 127.9 (Ar-CH), 129.8 (Ar-C), 130.5 (C-5), 130.6 (C-2'' and C-6''), 130.8 (C-4), 133.8 (C-1'), 133.9 (Ar-C), 146.3 (C-3 and 5-COCH<u>C</u>H), 152.8 (C-4''), 157.3 (C-6), 159.3 (C-7a), 164.8 (2-CONH), 193.0 (5-CO). ν_{max} (ATR)/cm^{-1} 3422 (N-H amide), 3382 (N-H amine), 2957 (C-H aromatic), 2847 (C-H alkane), 1650 (C=O carbonyl), 1635 (C=O amide), 1558 (C=C aromatic), 1430 (-C-H bending), 1259 (C-N aromatic), 1181 (C-O ether), 1066 (C-N aliphatic). *m/z* (ESI^+): 571 (MNa^+, 100%), 252 (60%). HRMS (ESI^+) found (MNa^+): 571.1756 $C_{32}H_{28}N_4NaO_3S$ requires 571.1774.

(*E*)-3-Amino-*N*-(4'-methoxyphenyl)-6-methyl-5-(3-(4''-morpholinophenyl)acryloyl)thieno[2,3-*b*]pyridine-2-carboxamide 12e

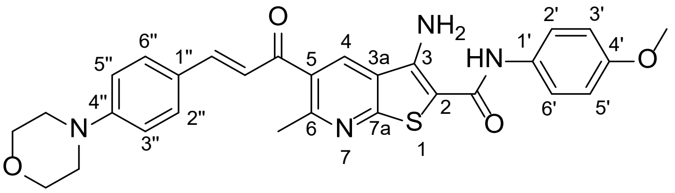

The reaction was carried out following General Procedure **A** using carbonitrile **9c** (50.0 mg, 0.14 mmol), chloride **4e** (27.0 mg, 0.14 mmol) and anhydrous sodium carbonate (29.0 mg, 0.27 mmol) in absolute ethanol (2.00 mL) for 48 h to give the *title compound* **12e** (78.0 mg, quant.) as a yellow solid. m.p. > 230 °C. δ_H (400 MHz, $(CD_3)_2SO$) 2.68 (3H, s, 6-CH_3), 3.26 (4H, t, *J* = 4.7 Hz, 2 × *N*-CH_2), 3.73 (4H, t, *J* = 4.7 Hz, 2 × *O*-CH_2), 3.75 (3H, s, 4'-OCH_3), 6.91 (2H, d, *J* = 9.0 Hz, H-3' and H-5'), 6.99 (2H, d, *J* = 8.9 Hz, H-3'' and H-5''), 7.29 (1H, d, *J* = 15.8 Hz, 5-COC<u>H</u>CH), 7.40 (2H, br s, NH_2), 7.54 (1H, d, *J* = 15.8 Hz, 5-COCHC<u>H</u>), 7.59 (2H, d, *J* = 9.0 Hz, H-2' and H-6'), 7.69 (2H, d, *J* = 9.0 Hz, H-2'' and H-6''), 8.78 (1H, s, H-4), 9.37 (1H, br s, NH). δ_C (100 MHz, $(CD_3)_2SO$) 23.8 (6-CH_3), 47.1 (2 × *N*-CH_2), 65.9 (2 × *O*-CH_2), 96.6 (C-2), 113.6 (C-3' and C-5'), 113.9 (C-3'' and C-5''), 121.6 (5-CO<u>C</u>HCH), 122.9 (C-5, C-2' and C-6'), 123.6 (C-3a), 124.2 (C-1''), 130.6 (C-2'' and C-6''), 130.9 (C-4), 131.9 (C-1'), 146.4 (5-COCH<u>C</u>H), 146.7 (C-3), 152.9 (C-4''), 155.5 (C-4'), 157.5 (C-6), 159.2 (C-7a), 163.6 (2-CONH), 192.9 (5-CO). ν_{max} (ATR)/cm^{-1} 3403 (N-H amide), 3308 (N-H amine), 2963 (C-H aromatic), 2838 (C-H alkane), 1662 (C=O carbonyl), 1619 (C=O amide), 1587 (C=C aromatic), 1510 (-C-H bending), 1236 (C-N aromatic), 1186 (C-O ether), 1040 (C-N aliphatic). *m/z* (ESI^+): 551 (MNa^+, 100%). HRMS (ESI^+) found (MNa^+): 551.1717 $C_{29}H_{28}N_4NaO_4S$ requires 551.1723.

3-Amino-5-cinnamoyl-6-methyl-*N*-phenylthieno[2,3-*b*]pyridine-2-carboxamide 13a

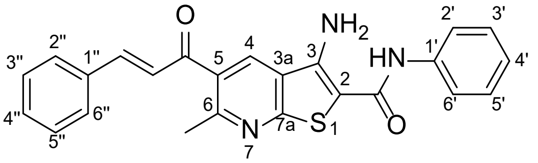

The reaction was carried out following General Procedure **A** using carbonitrile **9d** (0.15 g, 0.54 mmol), chloride **4a** (91.0 mg, 0.54 mmol) and anhydrous sodium carbonate (0.11 g, 1.07 mmol) in absolute ethanol (3.00 mL) for 48 h to give the *title compound* **13a** (0.14 g, 61%) as a brown

solid. m.p. decomp. 116 °C. δ_H (400 MHz, $(CD_3)_2SO$) 2.72 (3H, s, 6-CH_3), 7.08 (1H, t, *J* = 7.4 Hz, H-4'), 7.33 (2H, t, *J* = 7.4 Hz, H-3' and H-5'), 7.46-7.48 (5H, m, H-3", H-4", H-5" and NH_2), 7.56 (1H, d, *J* = 15.9 Hz, 5-COC<u>H</u>CH), 7.66 (1H, d, *J* = 15.9 Hz, 5-COCHC<u>H</u>), 7.71 (2H, d, *J* = 7.4 Hz, H-2' and H-6'), 7.82-7.85 (2H, m, H-2" and H-6"), 8.89 (1H, s, H-4), 9.49 (1H, br s, NH). δ_C (100 MHz, $(CD_3)_2SO$) 24.1 (6-CH_3), 96.4 (C-2), 121.1 (C-2' and C-6'), 123.4 (C-4'), 123.6 (C-3a), 125.6 (CO<u>C</u>HCH), 128.4 (C-3' and C-5'), 128.9 and 129.0 (C-3" and C-5", C-2" and C-6"), 129.8 (C-5), 130.9 (C-4"), 131.5 (C-4), 134.4 (C-1"), 138.9 (C-1'), 145.6 (5-COCH<u>C</u>H), 147.1 (C-3), 158.0 (C-6), 159.6 (C-7a), 163.7 (2-CONH), 192.8 (5-CO). ν_{max} (ATR)/cm^{-1} 3455 (N-H amide), 3354 (N-H amine), 3034 (C-H aromatic), 2835 (C-H alkane), 1665 (C=O carbonyl), 1630 (C=O amide), 1586 (C=C aromatic), 1438 (-C-H bending), 1258 (C-N aromatic), 1066 (C-N aliphatic). *m/z* (ESI^+): 436 (MNa^+, 100%), 295 (10%). HRMS (ESI^+) found (MNa^+): 436.1078 $C_{24}H_{19}N_3NaO_2S$ requires 436.1090.

3-Amino-5-cinnamoyl-6-methyl-*N*-(*o*-tolyl)thieno[2,3-*b*]pyridine-2-carboxamide 13b

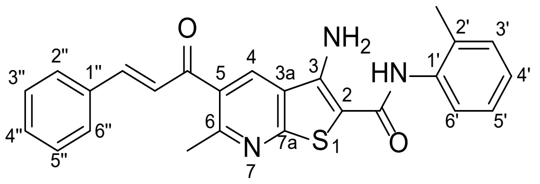

The reaction was carried out following General Procedure **A** using carbonitrile **9d** (50.0 mg, 0.18 mmol), chloride **4b** (33.0 mg, 0.18 mmol) and anhydrous sodium carbonate (38.0 mg, 0.36 mmol) in absolute ethanol (2.00 mL) for 48 h to give the *title compound* **13b** (34.0 mg, 44%) as a yellow solid. m.p. 183-185 °C. δ_H (400 MHz, $(CD_3)_2SO$) 2.24 (3H, s, 2'-CH_3), 2.72 (3H, s, 6-CH_3), 7.14-7.22 (2H, m, H-4' and H-5'), 7.26 (1H, dd, *J* = 7.5, 1.4 Hz, H-3'), 7.32 (1H, dd, *J* = 7.5, 1.4 Hz, H-6'), 7.36 (2H, br s, NH_2), 7.47-7.49 (3H, m, H-3", H-4" and H-5"), 7.56 (1H, d, *J* = 15.9 Hz, 5-COC<u>H</u>CH), 7.65 (1H, d, *J* = 15.9 Hz, 5-COCHC<u>H</u>), 7.82-7.85 (2H, m, H-2" and H-6"), 8.89 (1H, s, H-4), 9.20 (1H, br s, NH). δ_C (100 MHz, $(CD_3)_2SO$) 17.9 (2'-CH_3), 24.1 (6-CH_3), 96.8 (C-2), 123.7 (C-3a), 125.6 (5-CO<u>C</u>HCH), 125.9 (C-4' and C-5'), 126.9 (C-6'), 128.9 and 129.0 (C-2" and C-6", C-3" and C-5"), 129.8 (C-5), 130.2 (C-3'), 130.9 (C-4"), 131.5 (C-4), 134.0 (C-2'), 134.4 (C-1"), 136.4 (C-1'), 145.6 (5-COCH<u>C</u>H), 146.5 (C-3), 157.8 (C-6), 159.6 (C-7a), 163.7 (2-CONH), 192.9 (5-CO). ν_{max} (ATR)/cm^{-1} 3405 (N-H amide), 3282 (N-H amine), 3034 (C-H aromatic), 2920 (C-H alkane), 1662 (C=O carbonyl), 1634 (C=O amide), 1583 (C=C aromatic), 1446 (-C-H bending), 1256 (C-N aromatic), 1071

(C-N aliphatic). *m/z* (ESI$^+$): 450 (MNa$^+$, 100%). HRMS (ESI$^+$) found (MNa$^+$): 450.1229 $C_{25}H_{21}N_3NaO_2S$ requires 450.1247.

3-Amino-*N*-(3'-chloro-2'-methylphenyl)-5-cinnamoyl-6-methylthieno[2,3-*b*]pyridine-2-carboxamide 13c

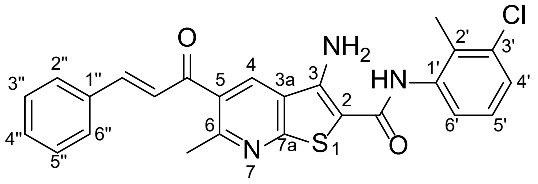

The reaction was carried out following General Procedure **A** using carbonitrile **9d** (50.0 mg, 0.18 mmol), chloride **4c** (39.0 mg, 0.18 mmol) and anhydrous sodium carbonate (38.0 mg, 0.36 mmol) in absolute ethanol (2.00 mL) for 48 h to give the *title compound* **13c** (60.0 mg, 72%) as a yellow solid. m.p. 219-221 °C. δ_H (400 MHz, $(CD_3)_2SO$) 2.25 (3H, s, 2'-CH_3), 2.72 (3H, s, 6-CH_3), 7.22 (1H, t, *J* = 7.9 Hz, H-5'), 7.31-7.33 (2H, m, H-4' and H-6'), 7.47-7.48 (5H, m, H-3", H-4", H-5" and NH_2), 7.57 (1H, d, *J* = 16.0 Hz, 5-COC<u>H</u>CH), 7.65 (1H, d, *J* = 16.0 Hz, 5-COCHC<u>H</u>), 7.83-7.85 (2H, m, H-2" and H-6"), 8.90 (1H, s, H-4), 9.42 (1H, br s, NH). δ_C (100 MHz, $(CD_3)_2SO$) 15.5 (2'-CH_2), 24.1 (6-CH_3), 96.8 (C-2), 123.7 (C-3a), 125.6 (5-CO<u>C</u>HCH), 126.0 (C-6'), 126.3 (C-4'), 126.7 (C-5'), 128.9 and 129.0 (C-2" and C-6", C-3" and C-5"), 129.7 (C-5), 130.9 (C-4"), 131.6 (C-4), 132.4 (C-2'), 133.6 (C-3'), 134.4 (C-1"), 138.7 (C-1'), 145.6 (5-COCH<u>C</u>H), 146.6 (C-3), 157.8 (C-6), 159.7 (C-7a), 163.9 (2-CONH), 192.8 (5-CO). ν_{max} (ATR)/cm^{-1} 3414 (N-H amide), 3371 (N-H amine), 3130 (C-H aromatic), 2938 (C-H alkane), 1662 (C=O carbonyl), 1631 (C=O amide), 1584 (C=C aromatic), 1429 (-C-H bending), 1259 (C-N aromatic), 1068 (C-N aliphatic), 764 (C-Cl). *m/z* (ESI$^+$): 486 (^{37}ClMNa$^+$, 40%), 484 (^{35}ClMNa$^+$, 100%). HRMS (ESI$^+$) found (^{37}ClMNa$^+$): 486.0825 $C_{25}H_{20}{}^{37}ClN_3NaO_2S$ requires 486.0836. Found (^{35}ClMNa$^+$): 484.0846 $C_{25}H_{20}{}^{35}ClN_3NaO_2S$ requires 484.0857.

3-Amino-5-cinnamoyl-6-methyl-*N*-(naphthalen-1'-yl)thieno[2,3-*b*]pyridine-2-carboxamide 13d

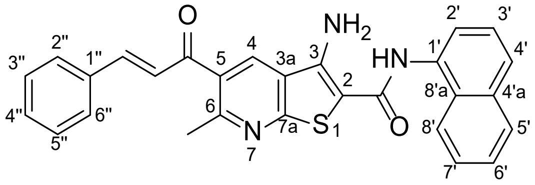

The reaction was carried out following General Procedure **A** using carbonitrile **9d** (0.10 g, 0.36 mmol), chloride **4d** (78.0 mg, 0.36 mmol) and anhydrous sodium carbonate (76.0 mg, 0.71 mmol) in absolute ethanol (3.00 mL) for 48 h to give the *title compound* **13d** (17.0 mg, 10%) as a yellow solid. m.p. > 230 °C. δ_H (400 MHz, $(CD_3)_2SO$) 2.74 (3H, s, 6-CH_3), 7.40 (2H, br s, NH_2), 7.48-7.49 (3H, m, H-3", H-4" and H-5"), 7.54-7.59 (5H, m, 4 × Ar-CH, 5-COC<u>H</u>CH), 7.66 (1H, d, *J* = 16.0 Hz, 5-COCHC<u>H</u>), 7.83-7.86 (3H, m, H-2", H-6" and Ar-CH), 7.94-7.99 (2H, m, 2 × Ar-CH), 8.89 (1H, s, H-4), 9.77 (1H, br s, NH). δ_C (100 MHz, $(CD_3)_2SO$) 24.1 (6-CH_3), 96.8 (C-2), 123.5 (Ar-CH), 123.6 (C-3a), 124.4 (Ar-CH), 125.5 (Ar-CH), 125.6 (Ar-CH), 125.9 (Ar-CH), 126.0 (5-CO<u>C</u>HCH), 126.3 (Ar-CH), 128.0 (Ar-CH), 128.9 and 129.0 (C-2" and C-6", C-3" and C-5"), 129.7 (Ar-C), 129.8 (C-5), 130.9 (C-4"), 131.5 (C-4), 133.7 (C-1' and Ar-C), 134.4 (C-1"), 145.6 (5-COCH<u>C</u>H), 146.8 (C-3), 157.9 (C-6), 159.7 (C-7a), 164.6 (2-CONH), 192.9 (5-CO). ν_{max} (ATR)/cm^{-1} 3454 (N-H amide), 3348 (N-H amine), 2931 (C-H aromatic), 1707 (C=O carbonyl), 1622 (C=O amide), 1541 (C=C aromatic), 1430 (-C-H bending), 1264 (C-N aromatic), 1061 (C-N aliphatic). *m/z* (ESI^+): 486 (MNa^+, 100%). HRMS (ESI^+) found (MNa^+): 486.1228 $C_{28}H_{21}N_3NaO_2S$ requires 486.1247.

3-Amino-5-cinnamoyl-*N*-(4'-methoxyphenyl)-6-methylthieno[2,3-*b*]pyridine-2-carboxamide 13e

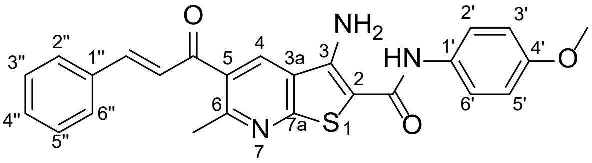

The reaction was carried out following General Procedure **A** using carbonitrile **9d** (50.0 mg, 0.18 mmol), chloride **4e** (36.0 mg, 0.18 mmol) and anhydrous sodium carbonate (38.0 mg, 0.36 mmol) in absolute ethanol (2.00 mL) for 48 h to give the *title compound* **13e** (25.0 mg, 32%) as a mustard yellow solid. m.p. 183-185 °C. δ_H (400 MHz, $(CD_3)_2SO$) 2.72 (3H, s, 6-CH_3),

3.74 (3H, s, 4'-OCH_3), 6.90 (2H, d, *J* = 9.0 Hz, H-3' and H-5'), 7.42 (2H, br s, NH_2), 7.47-7.49 (3H, m, H-3'', H-4'' and H-5''), 7.57 (1H, d, *J* = 15.9 Hz, 5-COCH̲CH), 7.57-7.59 (2H, d, *J* = 9.0 Hz, H-2' and H-6'), 7.66 (1H, d, *J* 15.9 Hz, 5-COCHCH̲), 7.82-7.85 (2H, m, H-2'' and H-6''), 8.87 (1H, s, H-4), 9.39 (1H, br s, NH). δ_C (100 MHz, $(CD_3)_2SO$) 24.0 (6-CH_3), 55.1 (4'-OCH_3), 96.6 (C-2), 113.5 (C-3' and C-5'), 122.9 (C-2' and C-6'), 123.6 (C-3a), 125.6 (5-COC̲HCH), 128.8 and 129.0 (C-2'' and C-6'', C-3'' and C-5''), 129.8 (C-5), 130.9 (C-4''), 131.4 (C-4), 131.8 (C-1'), 134.4 (C-1''), 145.5 (5-COCHC̲H), 146.6 (C-3), 155.5 (C-4'), 157.8 (C-6), 159.5 (C-7a), 163.5 (2-CONH), 192.8 (5-CO). ν_{max} (ATR)/cm^{-1} 3457 (N-H amide), 3336 (N-H amine), 3009 (C-H aromatic), 2836 (C-H alkane), 1655 (C=O carbonyl), 1589 (C=O amide), 1505 (C=C aromatic), 1448 (-C-H bending), 1296 (C-N aromatic), 1173 (C-O ether), 1083 (C-N aliphatic). *m/z* (ESI^+): 466 (MNa^+, 100%). HRMS (ESI^+) found (MNa^+): 466.1179 $C_{25}H_{21}N_3NaO_3S$ requires 466.1196.

(*E*)-3-Amino-5-(3-(3''-methoxyphenyl)acryloyl)-6-methyl-*N*-phenylthieno[2,3-*b*]pyridine-2-carboxamide 14a

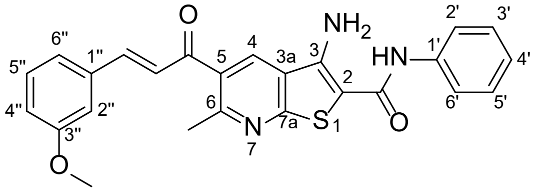

The reaction was carried out following General Procedure **A** using carbonitrile **9e** (60.0 mg, 0.19 mmol), chloride **4a** (33.0 mg, 0.19 mmol) and anhydrous sodium carbonate (41.0 mg, 0.39 mmol) in absolute ethanol (2.00 mL) for 48 h to give the *title compound* **14a** (69.0 mg, 80%) as a brown solid. m.p. 225-227 °C. δ_H (400 MHz, $(CD_3)_2SO$) 2.71 (3H, s, 6-CH_3), 3.81 (3H, s, 3''-OCH_3), 7.03-7.09 (2H, m, H-4' and H-4''), 7.33 (2H, t, *J* = 7.6 Hz, H-3' and H-5'), 7.36-7.43 (3H, m, H-2'', H-5'' and H-6''), 7.50 (2H, br s, NH_2), 7.58 (1H, d, *J* = 15.9 Hz, 5-COCH̲CH), 7.62 (1H, d, *J* = 15.9 Hz, 5-COCHCH̲), 7.71 (2H, d, *J* = 7.6 Hz, H-2' and H-6'), 8.82 (1H, s, H-4), 9.49 (1H, br s, NH). δ_C (100 MHz, $(CD_3)_2SO$) 24.0 (6-CH_3), 55.3 (3''-OCH_3), 96.4 (C-2), 113.7 (C-2''), 116.9 (C-4''), 121.1 (C-2' and C-6'), 121.4 (C-6''), 123.4 (C-4'), 123.6 (C-3a), 125.9 (COC̲HCH), 128.4 (C-3' and C-5'), 129.9 and 130.0 (C-5 and C-5''), 131.6 (C-4), 135.8 (C-1''), 138.9 (C-1'), 145.6 (5-COCHC̲H), 147.1 (C-3), 157.9 (C-6), 159.6 (C-7a), 159.7 (C-3''), 163.8 (2-CONH), 193.0 (5-CO). ν_{max} (ATR)/cm^{-1} 3455 (N-H amide), 3338 (N-H amine), 3064 (C-H aromatic), 2966 (C-H alkane), 1636 (C=O carbonyl), 1593 (C=O amide), 1542 (C=C aromatic), 1435 (-C-H bending), 1253 (C-N aromatic), 1103 (C-O ether), 1052 (C-N

aliphatic). *m/z* (ESI$^+$): 466 (MNa$^+$, 100%). HRMS (ESI$^+$) found (MNa$^+$): 466.1193 $C_{25}H_{21}N_3NaO_3S$ requires 466.1196.

(*E*)-3-Amino-5-(3-(3''-methoxyphenyl)acryloyl)-6-methyl-*N*-(*o*-tolyl)thieno[2,3-*b*]pyridine-2-carboxamide 14b

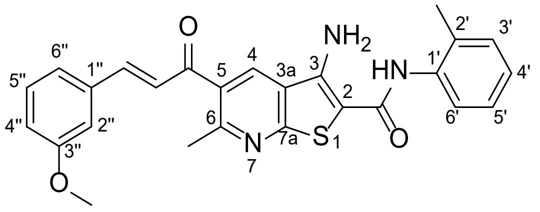

The reaction was carried out following General Procedure **A** using carbonitrile **9e** (70.0 mg, 0.23 mmol), chloride **4b** (42.0 mg, 0.23 mmol) and anhydrous sodium carbonate (48.0 mg, 0.45 mmol) in absolute ethanol (3.00 mL) for 48 h to give the *title compound* **14b** (0.10 g, quant.) as a crystalline brown solid. m.p. decomp 80 °C. δ_H (400 MHz, $(CD_3)_2SO$) 2.24 (3H, s, 2'-CH_3), 2.71 (3H, s, 6-CH_3), 3.81 (3H, s, *J* = 3''-OCH_3), 7.05 (1H, dt, *J* = 7.6, 1.8 Hz, H-4''), 7.16 (1H, td, *J* = 7.6, 1.8 Hz, H-4'), 7.21 (1H, td, *J* = 7.6, 1.8 Hz, H-5'), 7.27 (1H, dd, *J* = 7.6, 1.8 Hz, H-3'), 7.32 (1H, dd, *J* = 7.6, 1.8 Hz, H-6'), 7.35 (2H, br s, NH_2), 7.38-7.42 (3H, m, H-2'', H-5'' and H-6''), 7.54 (1H, d, *J* = 15.9 Hz, 5-COC$\underline{H}$CH), 7.61 (1H, d, *J* = 15.9 Hz, 5-COCHC$\underline{H}$), 8.84 (1H, s, H-4), 9.19 (1H, br s, NH). δ_C (100 MHz, $(CD_3)_2SO$) 17.9 (2'-CH_3), 24.0 (6-CH_3), 55.3 (3''-OCH_3), 96.8 (C-2), 113.7 (C-2''), 116.9 (C-4''), 121.3 (C-6''), 123.7 (C-3a), 125.9 (C-4' and C-5'), 126.0 (5-CO$\underline{C}$HCH), 126.9 (C-6'), 129.9 and 130.0 (C-5 and C-5''), 130.2 (C-3'), 131.4 (C-4), 134.0 (C-2'), 135.8 (C-1''), 136.3 (C-1'), 145.7 (5-COCH$\underline{C}$H), 146.5 (C-3), 157.6 (C-6), 159.5 and 159.6 (C-3'' and C-7a), 163.7 (2-CONH), 193.2 (5-CO). ν_{max} (ATR)/cm^{-1} 3432 (N-H amide), 3318 (N-H amine), 2926 (C-H aromatic), 2833 (C-H alkane), 1582 (C=O carbonyl and C=O amide), 1515 (C=C aromatic), 1450 (-C-H bending), 1251 (C-N aromatic), 1191 (C-O ether), 1063 (C-N aliphatic). *m/z* (ESI$^+$): 480 (MNa$^+$, 100%). HRMS (ESI$^+$) found (MNa$^+$): 480.1335 $C_{26}H_{23}N_3NaO_3S$ requires 480.1352.

(*E*)-3-Amino-*N*-(3'-chloro-2'-methylphenyl)-5-(3-(3''-methoxyphenyl)acryloyl)-6-methylthieno[2,3-*b*]pyridine-2-carboxamide 14c

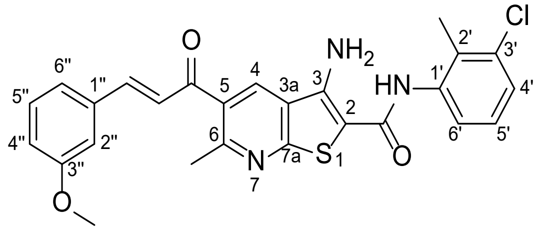

The reaction was carried out following General Procedure **A** using carbonitrile **9e** (50.0 mg, 0.16 mmol), chloride **4c** (35.0 mg, 0.16 mmol) and anhydrous sodium carbonate (34.0 mg, 0.32 mmol) in absolute ethanol (2.00 mL) for 48 h to give the *title compound* **14c** (35.0 mg, 44%) as a yellow solid. m.p. 211-213 °C. δ_H (400 MHz, $(CD_3)_2SO$) 2.25 (3H, s, 2'-CH_3), 2.71 (3H, s, 6-CH_3), 3.81 (3H, s, 3"-OCH_3), 7.05 (1H, dt, *J* = 7.5, 1.6 Hz, H-4"), 7.24 (1H, t, *J* = 7.5 Hz, H-5'), 7.29 (1H, dd, *J* = 7.5, 1.6 Hz, H-6'), 7.35 (1H, dd, *J* = 7.5, 1.6 Hz, H-4'), 7.38-7.42 (5H, m, H-2", H-5", H-6" and NH_2), 7.54 (1H, d, *J* = 16.0 Hz, 5-COC$\underline{H}$CH), 7.61 (1H, d, *J* = 16.0 Hz, 5-COCHC$\underline{H}$), 8.86 (1H, s, H-4), 9.46 (1H, br s, NH). δ_C (100 MHz, $(CD_3)_2SO$) 15.4 (2'-CH_2), 24.0 (6-CH_3), 55.3 (3"-OCH_3), 96.2 (C-2), 113.7 (C-2"), 116.9 (C-4"), 121.3 (C-6"), 123.6 (C-3a), 126.0 (5-CO$\underline{C}$HCH), 126.2 (C-6'), 126.6 and 126.7 (C-4' and C-5'), 129.9 and 130.0 (C-5 and C-5'), 131.5 (C-4), 132.6 (C-2'), 133.6 (C-3'), 135.8 (C-1"), 138.1 (C-1'), 145.7 (5-COCH$\underline{C}$H), 146.9 (C-3), 157.8 (C-6), 159.59 and 159.64 (C-3" and C-7a), 163.9 (2-CONH), 193.1 (5-CO). ν_{max} (ATR)/cm^{-1} 3424 (N-H amide), 3383 (N-H amine), 2971 (C-H aromatic), 2837 (C-H alkane), 1664 (C=O carbonyl), 1634 (C=O amide), 1683 (C=C aromatic), 1431 (-C-H bending), 1258 (C-N aromatic), 1159 (C-O ether), 1052 (C-N aliphatic), 774 (C-Cl). *m/z* (ESI$^+$): 516 (^{37}ClMNa$^+$, 40%), 514 (^{35}ClMNa$^+$, 100%), 431 (10%), 363 (30%). HRMS (ESI$^+$) found (^{37}ClMNa$^+$): 516.0931 $C_{26}H_{22}{}^{37}ClN_3NaO_3S$ requires 516.0942. Found (^{35}ClMNa$^+$): 514.0950 $C_{26}H_{22}{}^{35}ClN_3NaO_3S$ requires 514.0963.

(*E*)-3-Amino-5-(3-(3''-methoxyphenyl)acryloyl)-6-methyl-*N*-(naphthalen-1'-yl)thieno[2,3-*b*]pyridine-2-carboxamide 14d

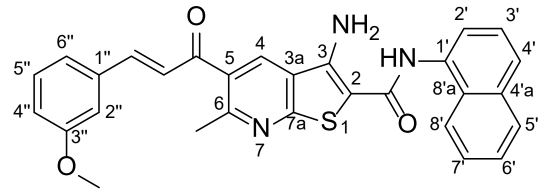

The reaction was carried out following General Procedure **A** using carbonitrile **9e** (0.15 g, 0.48 mmol), chloride **4d** (0.11 g, 0.48 mmol) and anhydrous sodium carbonate (0.10 g, 0.97 mmol) in absolute ethanol (4.00 mL) for 72 h to give the *title compound* **14d** (0.17 g, 70%) as a mustard solid. m.p. 211-213 °C. δ_H (400 MHz, $(CD_3)_2SO$) 2.73 (3H, s, 6-CH_3), 3.81 (3H, s, 3''-OCH_3), 7.05 (1H, dt, *J* = 7.5, 1.9 Hz, H-4''), 7.37-7.43 (5H, m, H-2'', H-5'', H-6'' and NH_2), 7.53-7.58 (5H, m, 4 × Ar-CH, 5-COC<u>H</u>CH), 7.63 (1H, d, *J* = 15.9 Hz, 5-COCHC<u>H</u>), 7.85-7.88 (1H, m, Ar-CH), 7.94-7.98 (2H, m, 2 × Ar-CH), 8.88 (1H, s, H-4), 9.77 (1H, br s, NH). δ_C (100 MHz, $(CD_3)_2SO$) 24.0 (6-CH_3), 55.3 (3''-OCH_3), 96.6 (C-2), 113.8 (C-2''), 116.9 (C-4''), 121.4 (C-6''), 123.5 (Ar-CH), 123.6 (C-3a), 124.4 (Ar-CH), 125.5 (Ar-CH), 125.9 (Ar-CH), 125.97 (Ar-CH), 126.01 (5-CO<u>C</u>HCH), 126.3 (Ar-CH), 128.0 (Ar-CH), 129.7 (Ar-C), 129.9 (C-5''), 130.0 (C-5), 131.5 (C-4), 133.7 (C-1'), 133.9 (Ar-C), 135.8 (C-1''), 145.7 (5-COCH<u>C</u>H), 146.9 (C-3), 157.8 (C-6), 159.6 and 159.7 (C-3'' and C-7a), 164.6 (2-CONH), 193.1 (5-CO). ν_{max} (ATR)/cm^{-1} 3416 (N-H amide), 3311 (N-H amine), 2925 (C-H aromatic), 2845 (C-H alkane), 1661 (C=O carbonyl), 1631 (C=O amide), 1583 (C=C aromatic), 1487 (-C-H bending), 1258 (C-N aromatic), 1170 (C-O ether), 1040 (C-N aliphatic). *m/z* (ESI^+): 516 (MNa^+, 100%), 398 (20%), 355 (23%). HRMS (ESI^+) found (MNa^+): 516.1347 $C_{29}H_{23}N_3NaO_3S$ requires 516.1352.

(*E*)-3-Amino-*N*-(4'-methoxyphenyl)-5-(3-(3''-methoxyphenyl)acryloyl)-6-methylthieno[2,3-*b*]pyridine-2-carboxamide 14e

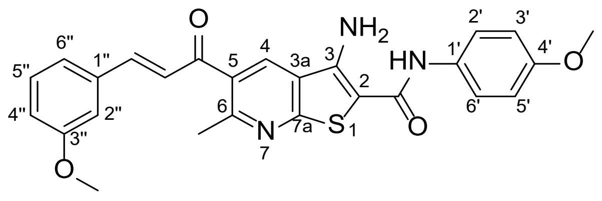

The reaction was carried out following General Procedure **A** using carbonitrile **9e** (0.15 g, 0.48 mmol), chloride **4e** (96.0 mg, 0.48 mmol) and anhydrous sodium carbonate (0.10 g, 0.97 mmol) in absolute ethanol (4.00 mL) for 72 h to give the *title compound* **14e** (0.14 g, 62%) as a yellow

solid. m.p. 113-115 °C. δ_H (400 MHz, $(CD_3)_2SO$) 2.71 (3H, s, 6-CH_3), 3.75 (3H, s, 4'-OCH_3), 3.81 (3H, s, 3''-OCH_3), 6.90 (2H, d, J = 9.0 Hz, H-3' and H-5'), 7.05 (1H, dt, J = 7.5, 1.8 Hz, H-4''), 7.36-7.42 (5H, m, H-2'', H-5'', H-6'' and NH_2), 7.53-7.64 (4H, m, 5-COC$\underline{H}$CH, 5-COCHC$\underline{H}$, H-2' and H-6'), 8.85 (1H, s, H-4), 9.38 (1H, br s, NH). δ_C (100 MHz, $(CD_3)_2SO$) 24.0 (6-CH_3), 55.1 and 55.3 (3''-OCH_3 and 4'-OCH_3), 96.6 (C-2), 113.6 (C-3' and C-5'), 113.7 (C-2''), 116.9 (C-4''), 121.4 (C-6''), 122.9 (C-2' and C-6'), 123.6 (C-3a), 126.0 (5-CO$\underline{C}$HCH), 129.9 and 130.0 (C-5 and C-5''), 131.4 (C-4), 131.9 (C-1'), 135.8 (C-1''), 145.6 (5-COCH$\underline{C}$H), 146.7 (C-3), 155.5 (C-4'), 157.7 (C-6), 159.5 and 159.6 (C-3'' and C-7a), 163.5 (2-CONH), 193.1 (5-CO). ν_{max} (ATR)/cm^{-1} 3399 (N-H amide), 3200 (N-H amine), 3062 (C-H aromatic), 2836 (C-H alkane), 1661 (C=O carbonyl), 1583 (C=O amide), 1511 (C=C aromatic), 1429 (-C-H bending), 1244 (C-N aromatic), 1170 (C-O ether), 1066 (C-N aliphatic). *m/z* (ESI^+): 496 (MNa^+, 100%). HRMS (ESI^+) found (MNa^+): 496.1298 $C_{26}H_{23}N_3NaO_4S$ requires 496.1301.

(*E*)-3-Amino-5-(3-(3'',4''-dihydroxyphenyl)acryloyl)-6-methyl-*N*-phenylthieno[2,3-*b*]pyridine-2-carboxamide 15a

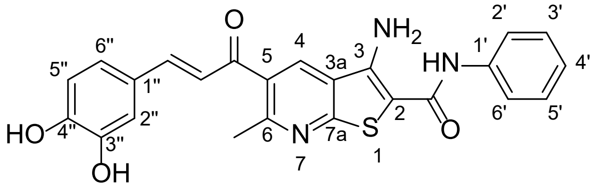

The reaction was carried out following General Procedure **D** using MOM-protected thienopyridine **10a** (50.0 mg, 0.09 mmol) and 6 M HCl (1.5 mL) in methanol (1.5 mL) for 24 h to give the *title compound* **15a** (20.0 mg, 48%) as a yellow solid. m.p. decomp. δ_H (400 MHz, $(CD_3)_2SO$) 2.68 (3H, s, 6-CH_3), 6.81 (1H, d, J = 8.0 Hz, H-5''), 7.08 (1H, t, J = 7.0 Hz, H-4'), 7.11 (1H, dd, J = 8.0, 2.0 Hz, H-6''), 7.20 (1H, d, J = 2.0 Hz, H-2''), 7.20 (1H, d, J = 16.0 Hz, 5-COC$\underline{H}$CH), 7.33 (2H, t, J = 7.0 Hz, H-3' and H-5'), 7.47 (2H, br s, NH_2), 7.47 (1H, d, J = 16.0 Hz, 5-COCHC$\underline{H}$), 7.70 (2H, d, J = 7.0 Hz, H-2' and H-6'), 8.82 (1H, s, H-4), 9.19 (1H, br s, 3''-OH), 9.47 (1H, br s, NH), 9.76 (1H, br s, 4''-OH). δ_C (100 MHz, $(CD_3)_2SO$) 23.9 (6-CH_3), 115.2 (C-2''), 115.8 (C-5''), 121.1 (C-2' and C-6'), 122.1 (5-CO$\underline{C}$HCH), 122.5 (C-6''), 123.4 (C-4'), 123.6 (C-3a), 125.9 (C-1''), 128.4 (C-3' and C-5'), 130.4 (C-5), 131.1 (C-4), 138.9 (C-1'), 145.7 (C-3''), 146.7 (5-COCH$\underline{C}$H), 147.1 (C-3), 149.1 (C-4''), 157.7 (C-6), 159.5 (C-7a), 163.8 (2-CONH), 192.8 (5-CO). C-2 not observed. ν_{max} (ATR)/cm^{-1} 3315 (very broad O-H alcohol, N-H amide and N-H amine), 2930 (C-H aromatic), 1652 (C=O carbonyl), 1582 (C=O amide), 1521 (C=C aromatic), 1439 (-C-H bending), 1252 (C-N aromatic), 1112 (C-O alcohol), 1059

(C-N aliphatic). *m/z* (ESI$^+$): 468 (MNa$^+$, 100%), 381 (10%), 252 (35%). HRMS (ESI$^+$) found (MNa$^+$): 468.0983 $C_{24}H_{19}N_3NaO_4S$ requires 468.0988.

(*E*)-3-Amino-5-(3-(3'',4''-dihydroxyphenyl)acryloyl)-6-methyl-*N*-(*o*-tolyl)thieno[2,3-*b*]pyridine-2-carboxamide 15b

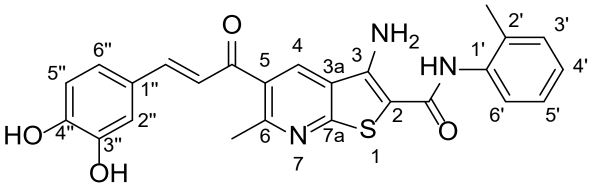

The reaction was carried out following General Procedure **D** using MOM-protected thienopyridine **10b** (50.0 mg, 0.09 mmol) and 6 M HCl (1.4 mL) in methanol (1.4 mL) for 24 h to give the *title compound* **15b** (16.0 mg, 38%) as a yellow solid. m.p. 174-176 °C. δ_H (400 MHz, $(CD_3)_2SO$) 2.24 (3H, s, 2'-CH_3), 2.68 (3H, s, 6-CH_3), 6.81 (1H, d, J = 8.0 Hz, H-5''), 7.12 (1H, dd, J = 8.0, 1.9 Hz, H-6''), 7.16-7.21 (4H, m, H-2'', H-4', H-5' and 5-COC<u>H</u>CH), 7.26 (1H, dd, J = 7.2, 1.5 Hz, H-3'), 7.32 (1H, dd, J = 7.2, 1.5 Hz, H-6'), 7.34 (2H, br s, NH_2), 7.46 (1H, d, J = 15.9 Hz, 5-COCHC<u>H</u>), 8.80 (1H, s, H-4), 9.18 (1H, br s, NH), 9.20 (1H, br s, 3''-OH), 9.76 (1H, br s, 4''-OH). δ_C (100 MHz, $(CD_3)_2SO$) 17.9 (2'-CH_3), 23.9 (6-CH_3), 96.7 (C-2), 115.2 (C-2''), 115.8 (C-5''), 122.1 (5-CO<u>C</u>HCH), 122.5 (C-6''), 123.6 (C-3a), 125.9 (C-4', C-5' and C-1''), 126.9 (C-6'), 130.2 (C-3'), 130.4 (C-5), 131.0 (C-4), 134.0 (C-2'), 136.3 (C-1'), 144.4 (C-3''), 145.7 (5-COCH<u>C</u>H), 146.5 (C-3), 149.1 (C-4''), 157.5 (C-6), 159.2 (C-7a), 163.7 (2-CONH), 192.8 (5-CO). ν_{max} (ATR)/cm^{-1} 3324 (O-H alcohol, N-H amide and N-H amine), 2925 (C-H aromatic), 1738 (C=O carbonyl), 1651 (C=O amide), 1584 (C=C aromatic), 1451 (-C-H bending), 1257 (C-N aromatic), 1114 (C-O ether), 1068 (C-N aliphatic). *m/z* (ESI$^+$): 482 (MNa$^+$, 100%), 398 (15%). HRMS (ESI$^+$) found (MNa$^+$): 482.1140 $C_{25}H_{21}N_3NaO_4S$ requires 482.1145.

(*E*)-3-Amino-*N*-(3'-chloro-2'-methylphenyl)-5-(3-(3'',4''-dihydroxyphenyl)acryloyl)-6-methylthieno[2,3-*b*]pyridine-2-carboxamide 15c

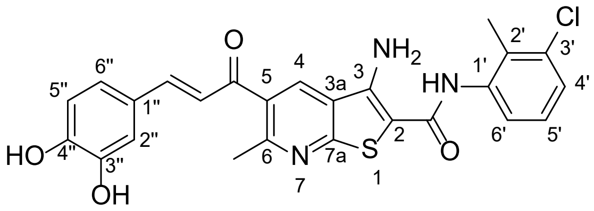

The reaction was carried out following General Procedure **D** using MOM-protected thienopyridine **10c** (50.0 mg, 0.09 mmol) and 6 M HCl (1.4 mL) in methanol (1.4 mL) for 24 h to give the *title compound* **15c** (25.0 mg, 60%) as a yellow solid. m.p. > 230 °C. δ_H (400 MHz, $(CD_3)_2SO$) 2.24 (3H, s, 2'-CH_3), 2.68 (3H, s, 6-CH_3), 6.80 (1H, d, *J* = 8.0 Hz, H-5''), 7.12 (1H, dd, *J* = 8.0, 1.9 Hz, H-6''), 7.18 (1H, d, *J* = 15.9 Hz, 5-COC$\underline{H}$CH), 7.19 (1H, d, *J* = 1.7 Hz, H-2''), 7.24 (1H, t, *J* = 7.9 Hz, H-5'), 7.29 (1H, dd, *J* = 7.9, 1.5 Hz, H-4' or H-6'), 7.36 (1H, dd, *J* = 7.9, 1.5 Hz, H-4' or H-6'), 7.39 (2H, br s, NH_2), 7.46 (1H, d, *J* = 15.9 Hz, 5-COCHC$\underline{H}$), 8.81 (1H, s, H-4), 9.45 (1H, br s, NH). 2 × OH not observed. δ_C (100 MHz, $(CD_3)_2SO$) 15.4 (2'-CH_3), 23.8 (6-CH_3), 96.1 (C-2), 115.1 (C-2''), 115.8 (C-5''), 121.9 (5-CO$\underline{C}$HCH), 122.6 (C-6''), 123.5 (C-3a), 125.7 (C-4' or C-6'), 126.2 (C-1''), 126.6 (C-4' or C-6'), 126.7 (C-5'), 130.5 (C-5), 131.1 (C-4), 132.6 (C-2'), 133.6 (C-3'), 138.1 (C-1'), 145.7 (C-3''), 146.8 (5-COCH$\underline{C}$H), 146.9 (C-3), 149.4 (C-4''), 157.7 (C-6), 159.3 (C-7a), 163.9 (2-CONH), 192.8 (5-CO). ν_{max} (ATR)/cm^{-1} 3330 (O-H alcohol, N-H amide and N-H amine), 2925 (C-H aromatic), 2845 (C-H alkane), 1737 (C=O carbonyl), 1652 (C=O amide), 1575 (C=C aromatic), 1431 (-C-H bending), 1252 (C-N aromatic), 1058 (C-N aliphatic), 737 (C-Cl). *m/z* (ESI^+): 518 ($^{37}ClMNa^+$, 40%), 516 ($^{35}ClMNa^+$, 100%), 381 (75%). HRMS (ESI^+) found ($^{37}ClMNa^+$): 518.0738 $C_{25}H_{20}{}^{37}ClN_3NaO_4S$ requires 518.0735. Found ($^{35}ClMNa^+$): 516.0748 $C_{25}H_{20}{}^{35}ClN_3NaO_4S$ requires 516.0755.

(*E*)-3-Amino-5-(3-(3'',4''-dihydroxyphenyl)acryloyl)-6-methyl-*N*-(naphthalen-1'-yl)thieno[2,3-*b*]pyridine-2-carboxamide 15d

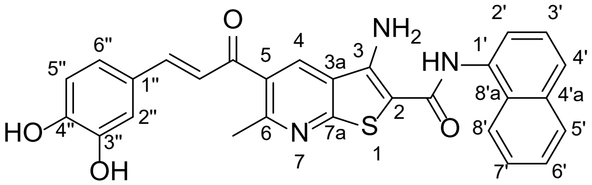

The reaction was carried out following General Procedure **D** using MOM-protected thienopyridine **10d** (50.0 mg, 0.09 mmol) and 6 M HCl (1.4 mL) in methanol (1.4 mL) for 24 h to give the *title compound* **15d** (25.0 mg, 60%) as a yellow solid. m.p. decomp. δ_H (400 MHz, $(CD_3)_2SO$) 2.70 (3H, s, 6-CH_3), 6.82 (1H, d, J = 8.2 Hz, H-5''), 7.13 (1H, dd, J = 8.2, 1.9 Hz, H-6''), 7.21 (1H, d, J = 2.0 Hz, H-2''), 7.21 (1H, d, J = 15.8 Hz, 5-COC<u>H</u>CH), 7.40 (2H, br s, NH_2), 7.48 (1H, d, J = 15.8 Hz, 5-COCHC<u>H</u>), 7.53-7.56 (4H, m, 4 × Ar-CH), 7.85-7.88 (1H, m, Ar-CH), 7.93-7.98 (2H, m, 2 × Ar-CH), 8.83 (1H, s, H-4), 9.21 (1H, br s, 3''-OH), 9.76 (1H, br s, 4''-OH), 9.77 (1H, br s, NH). δ_C (100 MHz, $(CD_3)_2SO$) 23.9 (6-CH_3), 96.5 (C-2), 115.2 (C-2''), 115.8 (C-5''), 122.1 (5-CO<u>C</u>HCH), 122.5 (C-6''), 123.5 (Ar-CH), 123.6 (C-3a), 124.4 (Ar-CH), 125.5 (Ar-CH), 125.86 and 125.92 (C-1'' and Ar-CH), 126.0 (Ar-CH), 126.3 (Ar-CH), 128.0 (Ar-CH), 129.7 (C-8'a), 130.4 (C-5), 131.1 (C-4), 136.7 (C-1'), 133.9 (C-4'a), 145.7 (C-3''), 146.8 and 146.9 (C-3 and 5-COCH<u>C</u>H), 149.1 (C-4''), 157.7 (C-6), 159.4 (C-7a), 163.6 (2-CONH), 192.8 (5-CO). ν_{max} (ATR)/cm^{-1} 3307 (O-H alcohol, N-H amide and N-H amine), 2918 (C-H aromatic), 2856 (C-H alkane), 1716 (C=O carbonyl), 1656 (C=O amide), 1584 (C=C aromatic), 1489 (-C-H bending), 1258 (C-N aromatic), 1059 (C-N aliphatic). *m/z* (ESI^+): 518 (MNa^+, 100%), 398 (10%). HRMS (ESI^+) found (MNa^+): 518.1133 $C_{28}H_{21}N_3NaO_4S$ requires 518.1145.

(*E*)-3-Amino-5-(3-(3'',4''-dihydroxyphenyl)acryloyl)-*N*-(4'-methoxyphenyl)-6-methylthieno[2,3-*b*]pyridine-2-carboxamide 15e

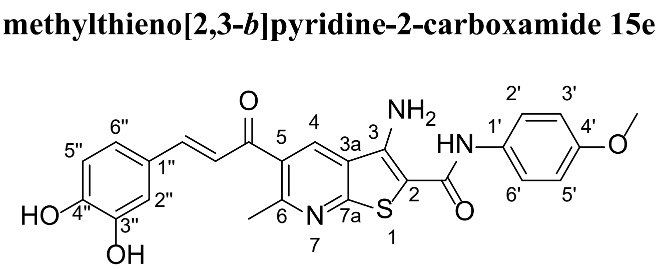

The reaction was carried out following General Procedure **D** using MOM-protected thienopyridine **10e** (50.0 mg, 0.09 mmol) and 6 M HCl (1.4 mL) in methanol (1.4 mL) for 24

h to give the *title compound* **15e** (17.0 mg, 40%) as a yellow solid. m.p. decomp. δ_H (400 MHz, $(CD_3)_2SO$) 2.68 (3H, s, 6-CH_3), 3.75 (3H, s, 4'-OCH_3), 6.81 (1H, d, J = 8.0 Hz, H-5''), 6.91 (2H, d, J = 9.0 Hz, H-3' and H-5'), 7.12 (1H, dd, J = 8.0, 2.0 Hz, H-6''), 7.20 (1H, d, J = 2.0 Hz, H-2''), 7.20 (1H, d, J = 16.0 Hz, 5-COC$\underline{H}$CH), 7.41 (2H, br s, NH_2), 7.47 (1H, d, J = 16.0 Hz, 5-COCHC$\underline{H}$), 7.58 (2H, d, J = 9.0 Hz, H-2' and H-6'), 8.80 (1H, s, H-4), 9.19 (1H, br s, 3''-OH), 9.37 (1H, br s, NH), 9.75 (1H, br s, 4''-OH). δ_C (100 MHz, $(CD_3)_2SO$) 23.8 (6-CH_3), 55.2 (4'-OCH_3), 96.5 (C-2), 113.6 (C-3' and C-5'), 115.2 (C-2''), 115.8 (C-5''), 122.1 (5-CO$\underline{C}$HCH), 122.5 (C-6''), 122.9 (C-2' and C-6'), 123.6 (C-3a), 125.9 (C-1''), 130.4 (C-5), 131.0 (C-4), 131.8 (C-1'), 145.7 (C-3''), 146.7 (C-3 and 5-COCH$\underline{C}$H), 149.1 (C-4''), 155.2 (C-4'), 157.5 (C-6), 159.2 (C-7a), 163.2 (2-CONH), 192.8 (5-CO). ν_{max} (ATR)/cm^{-1} 3315 (O-H alcohol, N-H amide and N-H amine), 2970 (C-H aromatic), 1740 (C=O carbonyl), 1585 (C=O amide), 1509 (C=C aromatic), 1442 (-C-H bending), 1232 (C-N aromatic), 1107 (C-O ether), 1067 (C-N aliphatic). *m/z* (ESI^+): 498 (MNa^+, 100%). HRMS (ESI^+) found (MNa^+): 498.1094 $C_{25}H_{21}N_3NaO_5S$ requires 498.1094.

(*E*)-3-Amino-*N*-(3'-chloro-2'-methylphenyl)-5-(3-(2''-methoxyphenyl)acryloyl)-6-methylthieno[2,3-*b*]pyridine-2-carboxamide 17f

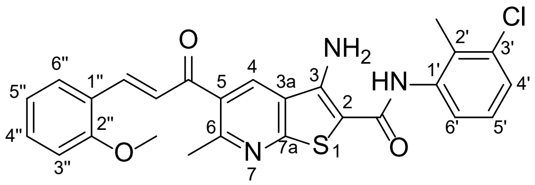

The reaction was carried out following General Procedure **A** using carbonitrile **9f** (0.10 g, 0.32 mmol), chloride **4c** (70.0 mg, 0.32 mmol) and anhydrous sodium carbonate (68.0 mg, 0.64 mmol) in absolute ethanol (5.00 mL) for 48 h to give the *title compound* **17f** (0.14 g, 89%) as a yellow solid. m.p. > 230 °C. δ_H (400 MHz, $(CD_3)_2SO$) 2.25 (3H, s, 2'-CH_3), 2.72 (3H, s, 6-CH_3), 3.88 (3H, s, 2''-OCH_3), 7.05 (1H, t, J = 7.5 Hz, H-5''), 7.13 (1H, d, J = 7.5 Hz, H-3''), 7.22 (1H, t, J = 7.9 Hz, H-5'), 7.31 (1H, d, J = 7.9 Hz, H-6'), 7.33 (1H, d, J = 7.9 Hz, H-4'), 7.42 (2H, br s, NH_2), 7.48 (1H, td, J = 7.5, 1.8 Hz, H-4''), 7.56 (1H, d, J = 15.9 Hz, 5-COC$\underline{H}$CH), 7.89 (1H, dd, 7.5, 1.8 Hz, H-6''), 7.91 (1H, d, J = 15.9 Hz, 5-COCHC$\underline{H}$), 8.90 (1H, s, H-4), 9.48 (1H, br s, NH). δ_C (100 MHz, $(CD_3)_2SO$) 15.5 (2'-CH_3), 24.1 (6-CH_3), 55.8 (2''-OCH_3), 111.9 (C-3''), 120.8 (C-5''), 122.6 (C-1''), 123.6 (C-3a), 125.5 (5-CO$\underline{C}$HCH), 126.1 (C-4' and C-6'), 126.7 (C-5'), 128.7 (C-6''), 130.0 (C-5), 131.5 (C-4), 132.4 (C-2'), 132.7 (C-4''), 133.6 (C-3'), 138.6 (C-1'), 139.9 (5-COCH$\underline{C}$H), 146.6 (C-3), 157.8 (C-6), 158.3 (C-2''), 159.6

(C-7a), 163.9 (2-CONH), 192.8 (5-CO). C-2 not observed. ν_{max} (ATR)/cm^{-1} 3434 (N-H amide), 3366 (N-H amine), 2954 (C-H aromatic), 2856 (C-H alkane), 1648 (C=O carbonyl), 1602 (C=O amide), 1573 (C=C aromatic), 1451 (-C-H bending), 1248 (C-N aromatic), 1180 (C-O ether), 1068 (C-N aliphatic), 748 (C-Cl). *m/z* (ESI$^+$): 516 (^{37}ClMNa$^+$, 35%), 514 (^{35}ClMNa$^+$, 100%). HRMS (ESI$^+$) found (^{37}ClMNa$^+$): 516.0927 $C_{26}H_{22}{}^{37}ClN_3NaO_3S$ requires 516.0942. Found (^{35}ClMNa$^+$): 514.0943 $C_{26}H_{22}{}^{35}ClN_3NaO_3S$ requires 514.0963.

(*E*)-3-Amino-*N*-(3'-chloro-2'-methylphenyl)-5-(3-(3''-(methoxymethoxy)phenyl)acryloyl)-6-methylthieno[2,3-*b*]pyridine-2-carboxamide 17g

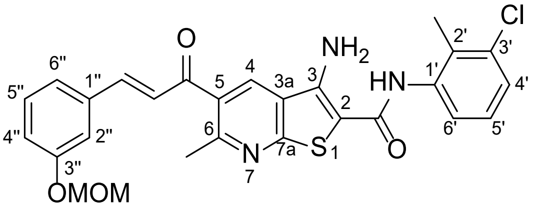

The reaction was carried out following General Procedure **A** using carbonitrile **9g** (0.16 g, 0.47 mmol), chloride **4c** (0.10 g, 0.47 mmol) and anhydrous sodium carbonate (0.10 g, 0.94 mmol) in absolute ethanol (6.00 mL) for 48 h to give the *title compound* **17g** (0.15 g, 61%) as a yellow solid. m.p. 145-147 °C. δ_H (400 MHz, $(CD_3)_2SO$) 2.25 (3H, s, 2'-CH_3), 2.70 (3H, s, 6-CH_3), 3.39 (3H, s, MOMCH_3), 5.25 (2H, s, MOMCH_2), 7.14 (1H, ddd, *J* = 8.0, 2.4, 1.0 Hz, H-4''), 7.17 (1H, d, *J* = 8.0 Hz, H-5'), 7.23 (1H, d, *J* = 8.0 Hz, H-6'), 7.38-7.42 (2H, m, H-4' and H-5''), 7.46-7.48 (2H, m, H-2'' and H-6''), 7.53 (1H, d, *J* = 15.9 Hz, 5-COC<u>H</u>CH), 7.59 (1H, d, *J* = 15.9 Hz, 5-COCHC<u>H</u>), 8.79 (1H, s, H-4). NH and NH_2 not observed. δ_C (100 MHz, $(CD_3)_2SO$) 15.5 (2'-CH_3), 24.0 (6-CH_3), 55.7 (MOMCH_3), 93.8 (MOMCH_2), 116.2 (C-2''), 118.7 (C-4''), 122.4 (C-6''), 123.9 (C-3a), 125.5 (C-4' and C-6'), 126.1 (5-CO<u>C</u>HCH), 126.5 (C-5'), 129.6 (C-5), 130.1 (C-5''), 131.2 (C-4), 132.3 (C-2'), 133.5 (C-3'), 135.8 (C-1''), 138.7 (C-1'), 145.4 (C-3 and 5-COCH<u>C</u>H), 157.1 (C-3''), 157.4 (C-6), 159.7 (C-7a), 164.2 (2-CONH), 193.1 (5-CO). C-2 not observed. ν_{max} (ATR)/cm^{-1} 3415 (N-H amide), 3311 (N-H amine), 2923 (C-H aromatic), 2844 (C-H alkane), 1661 (C=O carbonyl), 1620 (C=O amide), 1595 (C=C aromatic), 1435 (-C-H bending), 1232 (C-N aromatic), 1147 (C-O ether), 1065 (C-N aliphatic), 776 (C-Cl). *m/z* (ESI$^+$): 546 (^{37}ClMNa$^+$, 40%), 544 (^{35}ClMNa$^+$, 100%). HRMS (ESI$^+$) found (^{37}ClMNa$^+$): 546.1045 $C_{27}H_{24}{}^{37}ClN_3NaO_4S$ requires 546.1049. Found (^{35}ClMNa$^+$): 544.1056 $C_{27}H_{24}{}^{35}ClN_3NaO_4S$ requires 544.1068.

(*E*)-3-Amino-*N*-(3'-chloro-2'-methylphenyl)-6-methyl-5-(3-(2''-(trifluoromethyl)phenyl)acryloyl)thieno[2,3-*b*]pyridine-2-carboxamide 17h

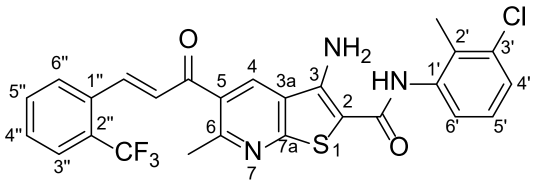

The reaction was carried out following General Procedure **A** using carbonitrile **9h** (0.13 g, 0.37 mmol), chloride **4c** (81.0 mg, 0.37 mmol) and anhydrous sodium carbonate (79.0 mg, 0.75 mmol) in absolute ethanol (5.00 mL) for 48 h to give the *title compound* **17h** (0.12 g, 62%) as an orange solid. m.p. decomp. δ_H (400 MHz, $(CD_3)_2SO$) 2.25 (3H, s, 2'-CH_3), 2.77 (3H, s, 6-CH_3), 7.19 (1H, t, J = 7.8 Hz, H-5'), 7.27 (1H, d, J = 7.8 Hz, H-6'), 7.30 (2H, br s, NH_2), 7.36 (1H, d, J = 7.8 Hz, H-4'), 7.68-7.93 (5H, m, H-3'', H-4'', H-5'', 5-COC<u>H</u>CH and 5-COCHC<u>H</u>), 8.24 (1H, d, J = 7.5 Hz, H-6''), 8.98 (1H, s, H-4), 9.47 (1H, br s, NH). δ_C (100 MHz, $(CD_3)_2SO$) 15.5 (2'-CH_3), 24.4 (6-CH_3), 123.7 (C-3a), 126.1 (C-6'), 126.6 (C-4'), 126.7 (C-5'), 127.6 (q, $^3J_{F/C}$ = 5.6 Hz, C-3''), 127.6 (q, $^2J_{F/C}$ = 30.1 Hz, C-2''), 127.9 (q, $^1J_{F/C}$ = 266.5 Hz, CF_3), 128.7 (C-5), 129.1 (C-6''), 129.3 (5-CO<u>C</u>HCH), 130.8 (C-4''), 132.2 (C-4), 132.5 (C-2'), 133.1 (C-5''), 133.5 (C-3'), 135.4 (q, $^3J_{F/C}$ = 5.1 Hz, C-1''), 138.5 (5-COCH<u>C</u>H), 139.7 (C-1'), 146.1 (C-3), 158.7 (C-6), 160.1 (C-7a), 164.0 (2-CONH), 191.3 (5-CO). C-2 not observed. ν_{max} (ATR)/cm^{-1} 3420 (N-H amide), 3339 (N-H amine), 2925 (C-H aromatic), 2857 (C-H alkane), 1651 (C=O carbonyl), 1608 (C=O amide), 1586 (C=C aromatic), 1428 (-C-H bending), 1289 (C-N aromatic), 1262 (C-F), 1062 (C-N aliphatic), 760 (C-Cl). *m/z* (ESI^+): 554 ($^{37}ClMNa^+$, 40%), 552 ($^{35}ClMNa^+$, 100%). HRMS (ESI^+) found ($^{37}ClMNa^+$): 554.0705 $C_{26}H_{19}{}^{37}ClF_3N_3NaO_2S$ requires 554.0710. Found ($^{35}ClMNa^+$): 552.0732 $C_{26}H_{19}{}^{35}ClF_3N_3NaO_2S$ requires 552.0731.

(*E*)-3-Amino-*N*-(3'-chloro-2'-methylphenyl)-5-(3-(2''-fluorophenyl)acryloyl)-6-methylthieno[2,3-*b*]pyridine-2-carboxamide 17i

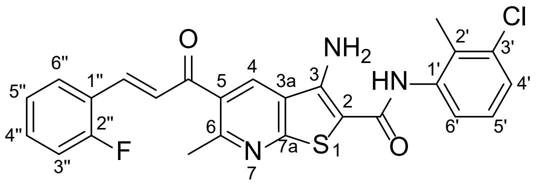

The reaction was carried out following General Procedure **A** using carbonitrile **9i** (80.0 mg g, 0.27 mmol), chloride **4c** (58.0 mg, 0.27 mmol) and anhydrous sodium carbonate (57.0 mg, 0.54 mmol) in absolute ethanol (4.00 mL) for 48 h to give the *title compound* **17i** (0.11 g, 86%) as a yellow solid. m.p. 200-202 °C. δ_H (400 MHz, $(CD_3)_2SO$) 2.25 (3H, s, 2'-CH_3), 2.74 (3H, s, 6-CH_3), 7.20 (1H, t, *J* = 7.9 Hz, H-5'), 7.29 (1H, d, *J* = 7.9 Hz, H-6'), 7.32-7.37 (3H, m, H-3'', H-4' and H-5''), 7.39 (2H, br s, NH_2), 7.52-7.58 (1H, m, H-4''), 7.64 (1H, d, *J* = 16.0 Hz, 5-COC$\underline{H}$CH), 7.73 (1H, d, *J* = 16.0 Hz, 5-COCHC$\underline{H}$), 8.01 (1H, td, *J* = 7.6, 1.8 Hz, H-6''), 8.89 (1H, s, H-4), 9.45 (1H, br s, NH). δ_C (100 MHz, $(CD_3)_2SO$) 15.5 (2'-CH_3), 24.2 (6-CH_3), 116.2 (d, ${}^2J_{F/C}$ = 21.3 Hz, C-3''), 122.1 (${}^2J_{F/C}$ = 11.0 Hz, C-1''), 123.8 (C-3a), 125.1 (d, ${}^4J_{F/C}$ = 3.0 Hz, C-5''), 125.8 (C-4' and C-6'), 126.6 (C-5'), 127.7 (5-CO$\underline{C}$HCH), 129.3 (C-5 and C-6''), 131.6 (C-4), 132.5 (C-2'), 133.0 (d, ${}^3J_{F/C}$ = 8.9 Hz, C-4''), 133.5 (C-3'), 136.6 (5-COCH$\underline{C}$H), 139.4 (C-1'), 146.4 (C-3), 157.8 (C-6), 159.9 (C-7a), 160.4 (d, ${}^1J_{F/C}$ = 242.0 Hz, C-2''), 164.0 (2-CONH), 192.4 (5-CO). C-2 not observed. ν_{max} (ATR)/cm^{-1} 3474 (N-H amide), 3364 (N-H amine), 2922 (C-H aromatic), 2844 (C-H alkane), 1662 (C=O carbonyl), 1609 (C=O amide), 1575 (C=C aromatic), 1481 (-C-H bending), 1267 (C-N aromatic), 1227 (C-F), 1060 (C-N aliphatic), 760 (C-Cl). *m/z* (ESI^+): 504 ($^{37}ClMNa^+$, 38%), 502 ($^{35}ClMNa^+$, 100%). HRMS (ESI^+) found ($^{37}ClMNa^+$): 504.0741 $C_{25}H_{19}{}^{37}ClFN_3NaO_2S$ requires 504.0741. Found ($^{35}ClMNa^+$): 502.0760 $C_{25}H_{19}{}^{35}ClFN_3NaO_2S$ requires 502.0763.

(*E*)-3-Amino-*N*-(3'-chloro-2'-methylphenyl)-5-(3-(3''-fluorophenyl)acryloyl)-6-methylthieno[2,3-*b*]pyridine-2-carboxamide 17j

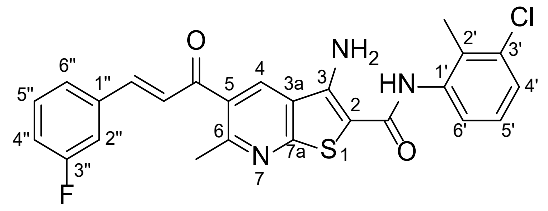

The reaction was carried out following General Procedure **A** using carbonitrile **9j** (0.10 g, 0.34 mmol), chloride **4c** (73.0 mg, 0.34 mmol) and anhydrous sodium carbonate (71.0 mg, 0.67 mmol) in absolute ethanol (5.00 mL) for 48 h to give the *title compound* **17j** (0.12 g, 72%) as a yellow solid. m.p. 196-198 °C. δ_H (400 MHz, $(CD_3)_2SO$) 2.25 (3H, s, 2'-CH_3), 2.72 (3H, s, 6-CH_3), 7.19 (1H, t, J = 7.9 Hz, H-5'), 7.27 (1H, d, J = 7.9 Hz, H-6'), 7.32 (1H, td, J = 7.9, 2.1 Hz, H-4''), 7.33 (2H, br s, NH_2), 7.36 (1H, d, J = 7.9 Hz, H-4'), 7.52 (1H, dt, J = 7.9, 6.1 Hz, H-5''), 7.60-7.67 (2H, m, 5-CO$C\underline{H}$CH and 5-COCH$C\underline{H}$), 7.67 (1H, dt, J = 7.9, 1.9 Hz, H-6''), 7.75 (1H, dt, J = 7.9, 1.9 Hz, H-2''), 8.84 (1H, s, H-4), 9.42 (1H, br s, NH). δ_C (100 MHz, $(CD_3)_2SO$) 15.5 (2'-CH_3), 24.1 (6-CH_3), 114.8 (d, $^2J_{F/C}$ = 20.2 Hz, C-2''), 117.5 (d, $^2J_{F/C}$ = 20.2 Hz, C-4''), 123.8 (C-3a), 125.3 (d, $^4J_{F/C}$ = 3.0 Hz, C-6''), 125.7 (C-4' and C-6'), 126.6 (C-5'), 127.0 (5-CO$\underline{C}$HCH), 129.5 (C-5), 131.0 (d, $^3J_{F/C}$ = 8.3 Hz, C-5''), 131.6 (C-4), 132.5 (C-2'), 133.5 (C-3'), 137.0 (C-1''), 139.7 (C-1'), 144.0 (5-COCH$\underline{C}$H), 146.6 (C-3), 157.9 (C-6), 159.8 (C-7a), 162.7 (d, $^1J_{F/C}$ = 243.0 Hz, C-3''), 163.9 (2-CONH), 192.8 (5-CO). C-2 not observed. ν_{max} (ATR)/cm^{-1} 3406 (N-H amide), 3377 (N-H amine), 2923 (C-H aromatic), 2855 (C-H alkane), 1661 (C=O carbonyl), 1632 (C=O amide), 1583 (C=C aromatic), 1432 (-C-H bending), 1257 (C-F), 1243 (C-N aromatic), 1068 (C-N aliphatic), 758 (C-Cl). *m/z* (ESI$^+$): 504 (^{37}ClMNa$^+$, 40%), 502 (^{35}ClMNa$^+$, 100%). HRMS (ESI$^+$) found (^{37}ClMNa$^+$): 504.0720 $C_{25}H_{19}{}^{37}ClFN_3NaO_2S$ requires 504.0741. Found (^{35}ClMNa$^+$): 502.0745 $C_{25}H_{19}{}^{35}ClFN_3NaO_2S$ requires 502.0763.

(*E*)-3-Amino-*N*-(3'-chloro-2'-methylphenyl)-5-(3-(4''-fluorophenyl)acryloyl)-6-methylthieno[2,3-*b*]pyridine-2-carboxamide 17k

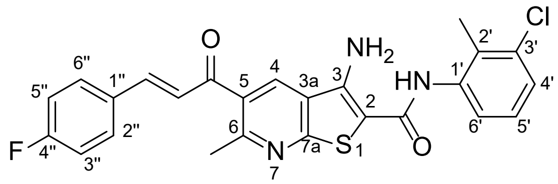

The reaction was carried out following General Procedure **A** using carbonitrile **9k** (0.10 g, 0.29 mmol), chloride **4c** (63.0 mg, 0.29 mmol) and anhydrous sodium carbonate (61.0 mg, 0.57 mmol) in absolute ethanol (5.00 mL) for 48 h to give the *title compound* **17k** (0.14 g, 99%) as a yellow solid. m.p. > 230 °C. δ_H (400 MHz, $(CD_3)_2SO$) 2.25 (3H, s, 2'-CH_3), 2.71 (3H, s, 6-CH_3), 7.22 (1H, t, J = 7.9 Hz, H-5'), 7.30-7.34 (4H, m, H-4', H-6', H-3'' and H-5''), 7.41 (2H, br s, NH_2), 7.51 (1H, d, J = 15.9 Hz, 5-COC$\underline{H}$CH), 7.65 (1H, d, J = 15.9 Hz, 5-COCHC$\underline{H}$), 7.90-7.93 (2H, m, H-2'' and H-6''), 8.85 (1H, s, H-4), 9.39 (1H, br s, NH). δ_C (100 MHz, $(CD_3)_2SO$) 15.5 (2'-CH_3), 24.0 (6-CH_3), 116.1 (d, $^2J_{F/C}$ = 21.3 Hz, C-3'' and C-5''), 123.7 (C-3a), 125.6 (5-CO$\underline{C}$HCH), 126.0 (C-4' or C-6'), 126.2 (C-4' or C-6'), 126.7 (C-5'), 129.8 (C-5), 131.1 ($^4J_{F/C}$ = 3.1 Hz, C-1''), 131.3 (d, $^3J_{F/C}$ = 8.7 Hz, C-2'' and C-6''), 131.5 (C-4), 132.3 (C-2'), 133.6 (C-3'), 138.7 (C-1'), 144.4 (5-COCH$\underline{C}$H), 146.5 (C-3), 157.7 (C-6), 159.6 (C-7a), 163.6 (d, $^1J_{F/C}$ = 249.2 Hz, C-4''), 164.8 (2-CONH), 192.9 (5-CO). C-2 not observed. ν_{max} (ATR)/cm^{-1} 3411 (N-H amide), 3378 (N-H amine), 2924 (C-H aromatic), 2855 (C-H alkane), 1660 (C=O carbonyl), 1633 (C=O amide), 1583 (C=C aromatic), 1464 (-C-H bending), 1259 (C-N aromatic), 1230 (C-F), 1067 (C-N aliphatic), 755 (C-Cl). *m/z* (ESI$^+$): 504 (^{37}ClMNa$^+$, 41%), 502 (^{35}ClMNa$^+$, 100%). HRMS (ESI$^+$) found (^{37}ClMNa$^+$): 504.0731 $C_{25}H_{19}{}^{37}ClFN_3NaO_2S$ requires 504.0741. Found (^{35}ClMNa$^+$): 502.0749 $C_{25}H_{19}{}^{35}ClFN_3NaO_2S$ requires 502.0763.

(*E*)-3-Amino-*N*-(3'-chloro-2'-methylphenyl)-5-(3-(2''-chlorophenyl)acryloyl)-6-methylthieno[2,3-*b*]pyridine-2-carboxamide 17l

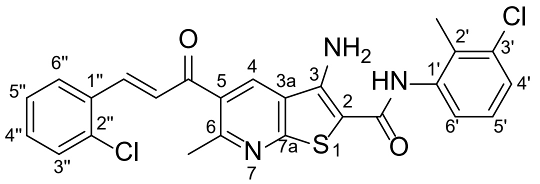

The reaction was carried out following General Procedure **A** using carbonitrile **9l** (0.10 g, 0.32 mmol), chloride **4c** (69.0 mg, 0.32 mmol) and anhydrous sodium carbonate (67.0 mg, 0.64 mmol) in absolute ethanol (5.00 mL) for 48 h to give the *title compound* **17l** (83.0 mg, 52%) as a yellow solid. m.p. > 230 °C. δ_H (400 MHz, $(CD_3)_2SO$) 2.25 (3H, s, 2'-CH_3), 2.76 (3H, s, 6-CH_3), 7.23 (1H, t, *J* = 7.9 Hz, H-5'), 7.31 (1H, d, *J* = 7.9 Hz, H-6'), 7.34 (1H, d, *J* = 7.9 Hz, H-4'), 7.39 (2H, br s, NH_2), 7.47-7.53 (2H, m, H-4'' and H-5''), 7.60 (1H, dd, *J* = 6.9, 1.8 Hz, H-3''), 7.69 (1H, d, *J* = 15.9 Hz, 5-COC$\underline{H}$CH), 7.95 (1H, d, *J* = 15.9 Hz, 5-COCHC$\underline{H}$), 8.11 (1H, dd, *J* = 6.9, 1.8 Hz, H-6''), 8.97 (1H, s, H-4), 9.47 (1H, br s, NH). δ_C (100 MHz, $(CD_3)_2SO$) 15.5 (2'-CH_3), 24.3 (6-CH_3), 123.7 (C-3a), 126.0 (C-4' and C-6'), 126.7 (C-5'), 127.0 (5-CO$\underline{C}$HCH), 127.9 (C-5''), 128.5 (C-6''), 129.2 (C-5), 130.1 (C-3''), 132.0 (C-4), 132.1 (C-4''), 132.3 (C-1''), 132.4 (C-2'), 133.6 (C-3'), 134.4 (C-2''), 138.7 (C-1'), 139.5 (5-COCH$\underline{C}$H), 146.6 (C-3), 158.2 (C-6), 160.0 (C-7a), 163.9 (2-CONH), 191.9 (5-CO). C-2 not observed. ν_{max} (ATR)/cm^{-1} 3464 (N-H amide), 3341 (N-H amine), 2923 (C-H aromatic), 2854 (C-H alkane), 1656 (C=O carbonyl), 1644 (C=O amide), 1576 (C=C aromatic), 1432 (-C-H bending), 1246 (C-N aromatic), 1051 (C-N aliphatic), 775 (C-Cl), 760 (C-Cl). *m/z* (ESI^+): 522 ($^{37}Cl_2MNa^+$, 12%), 520 ($^{37}Cl^{35}ClMNa^+$, 75%), 518 ($^{35}Cl_2MNa^+$, 100%). HRMS (ESI^+) found ($^{37}Cl_2MNa^+$): 522.0412 $C_{25}H_{19}{}^{37}Cl_2N_3NaO_2S$ requires 522.0422. Found ($^{35}Cl^{37}ClMNa^+$): 520.0435 $C_{25}H_{19}{}^{35}Cl^{37}ClN_3NaO_2S$ requires 520.0442. Found ($^{35}Cl_2MNa^+$): 518.0465 $C_{25}H_{19}{}^{35}Cl_2N_3NaO_2S$ requires 518.0467.

(*E*)-3-Amino-*N*-(3'-chloro-2'-methylphenyl)-5-(3-(3''-chlorophenyl)acryloyl)-6-methylthieno[2,3-*b*]pyridine-2-carboxamide 17m

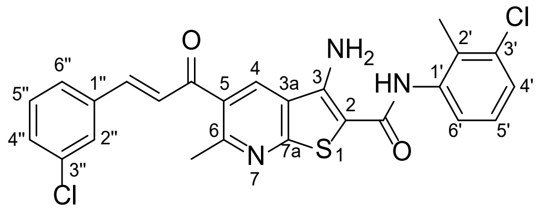

The reaction was carried out following General Procedure **A** using carbonitrile **9m** (0.10 g, 0.32 mmol), chloride **4c** (69.0 mg, 0.32 mmol) and anhydrous sodium carbonate (67.0 mg, 0.64 mmol) in absolute ethanol (5.00 mL) for 48 h to give the *title compound* **17m** (0.12 g, 77%) as a yellow solid. m.p. 214-216 °C. δ_H (400 MHz, $(CD_3)_2SO$) 2.25 (3H, s, 2'-CH_3), 2.72 (3H, s, 6-CH_3), 7.20 (1H, t, *J* = 7.9 Hz, H-5'), 7.29 (1H, d, *J* = 7.9 Hz, H-6'), 7.34 (1H, d, *J* = 7.9 Hz, H-4'), 7.38 (2H, br s, NH_2), 7.50 (1H, t, *J* = 7.5 Hz, H-5''), 7.53 (1H, dd, *J* = 7.5, 1.6 Hz, H-4''), 7.62 (1H, d, *J* = 15.9 Hz, 5-COC$\underline{H}$CH), 7.67 (1H, d, *J* = 15.9 Hz, 5-COCHC$\underline{H}$), 7.80 (1H, dt, *J* = 7.5, 1.6 Hz, H-6''), 7.80 (1H, t, *J* = 1.6 Hz, H-2''), 8.87 (1H, s, H-4), 9.43 (1H, br s, NH). δ_C (100 MHz, $(CD_3)_2SO$) 15.5 (2'-CH_3), 24.2 (6-CH_3), 123.8 (C-3a), 125.8 (C-4' and C-6'), 126.6 (C-5'), 127.0 (5-CO$\underline{C}$HCH), 127.6 (C-6''), 128.2 (C-2''), 129.5 (C-5), 130.4 (C-4''), 130.8 (C-5''), 131.7 (C-4), 132.0 (C-2'), 133.6 (C-3'), 133.8 (C-3''), 136.7 (C-1''), 138.5 (C-1'), 143.7 (5-COCH$\underline{C}$H), 145.8 (C-3), 158.1 (C-6), 159.8 (C-7a), 164.0 (2-CONH), 192.7 (5-CO). C-2 not observed. ν_{max} (ATR)/cm^{-1} 3436 (N-H amide), 3368 (N-H amine), 2922 (C-H aromatic), 2853 (C-H alkane), 1652 (C=O carbonyl), 1640 (C=O amide), 1594 (C=C aromatic), 1432 (-C-H bending), 1250 (C-N aromatic), 1075 (C-N aliphatic), 773 (C-Cl), 758 (C-Cl). *m/z* (ESI$^+$): 500 ($^{37}Cl_2MH^+$, 12%), 498 ($^{37}Cl^{35}ClMH^+$, 65%), 496 ($^{35}Cl_2MH^+$, 100%). HRMS (ESI$^+$) found ($^{37}Cl_2MH^+$): 500.0583 $C_{25}H_{20}{}^{37}Cl_2N_3O_2S$ requires 500.0603. Found ($^{35}Cl^{37}ClMH^+$): 498.0620 $C_{25}H_{20}{}^{35}Cl^{37}ClN_3O_2S$ requires 498.0623. Found ($^{35}Cl_2MH^+$): 496.0640 $C_{25}H_{20}{}^{35}Cl_2N_3O_2S$ requires 496.0648.

(*E*)-3-Amino-*N*-(3'-chloro-2'-methylphenyl)-5-(3-(4''-chlorophenyl)acryloyl)-6-methylthieno[2,3-*b*]pyridine-2-carboxamide 17n

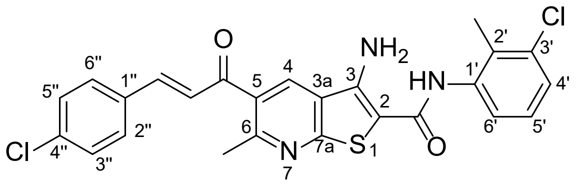

The reaction was carried out following General Procedure **A** using carbonitrile **9n** (0.10 g, 0.32 mmol), chloride **4c** (69.0 mg, 0.32 mmol) and anhydrous sodium carbonate (67.0 mg, 0.64 mmol) in absolute ethanol (5.00 mL) for 48 h to give the *title compound* **17n** (0.14 g, 89%) as a yellow solid. m.p. > 230 °C. δ_H (400 MHz, $(CD_3)_2SO$) 2.25 (3H, s, 2'-CH_3), 2.71 (3H, s, 6-CH_3), 7.20 (1H, t, *J* = 7.8 Hz, H-5'), 7.29 (1H, d, *J* = 7.8 Hz, H-6'), 7.34 (1H, d, *J* = 7.8 Hz, H-4'), 7.38 (2H, br s, NH_2), 7.55 (2H, d, *J* = 8.5 Hz, H-3" and H-5"), 7.58 (1H, d, *J* = 15.9 Hz, 5-COC$\underline{H}$CH), 7.65 (1H, d, *J* = 15.9 Hz, 5-COCHC$\underline{H}$), 7.88 (2H, d, *J* = 8.5 Hz, H-2" and H-6"), 8.86 (1H, s, H-4), 9.44 (1H, br s, NH). δ_C (100 MHz, $(CD_3)_2SO$) 15.4 (2'-CH_3), 24.1 (6-CH_3), 123.8 (C-3a), 125.8 (C-4' and C-6'), 126.3 (C-5'), 126.6 (5-CO$\underline{C}$HCH), 129.0 (C-3" and C-5"), 129.6 (C-5), 130.5 (C-2" and C-6"), 131.6 (C-4), 132.4 (C-2'), 133.4 (C-1"), 133.5 (C-3'), 135.4 (C-4"), 139.2 (C-1'), 144.0 (5-COCH$\underline{C}$H), 146.5 (C-3), 157.6 (C-6), 159.9 (C-7a), 164.0 (2-CONH), 192.8 (5-CO). C-2 not observed. ν_{max} (ATR)/cm^{-1} 3411 (N-H amide), 3377 (N-H amine), 2924 (C-H aromatic), 2853 (C-H alkane), 1660 (C=O carbonyl), 1632 (C=O amide), 1583 (C=C aromatic), 1465 (-C-H bending), 1258 (C-N aromatic), 1066 (C-N aliphatic), 775 (C-Cl), 758 (C-Cl). *m/z* (ESI$^+$): 522 (37Cl_2MNa^+, 13%), 520 ($^{37}Cl^{35}ClMNa^+$, 60%), 518 ($^{35}Cl_2MNa^+$, 100%). HRMS (ESI$^+$) found ($^{37}Cl_2MNa^+$): 522.0398 $C_{25}H_{19}{}^{37}Cl_2N_3NaO_2S$ requires 522.0422. Found ($^{35}Cl^{37}ClMNa^+$): 520.0430 $C_{25}H_{19}{}^{35}Cl^{37}ClN_3NaO_2S$ requires 520.0442. Found ($^{35}Cl_2MNa^+$): 518.0450 $C_{25}H_{19}{}^{35}Cl_2N_3NaO_2S$ requires 518.0467.

(*E*)-3-Amino-5-(3-(2''-bromophenyl)acryloyl)-*N*-(3'-chloro-2'-methylphenyl)-6-methylthieno[2,3-*b*]pyridine-2-carboxamide 17o

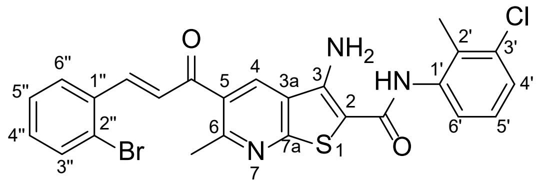

The reaction was carried out following General Procedure **A** using carbonitrile **9o** (0.10 g, 0.28 mmol), chloride **4c** (61.0 mg, 0.28 mmol) and anhydrous sodium carbonate (59.0 mg, 0.56 mmol) in absolute ethanol (5.00 mL) for 48 h to give the *title compound* **17o** (0.10 g, 67%) as a yellow solid. m.p. > 230 °C. δ_H (400 MHz, $(CD_3)_2SO$) 2.25 (3H, s, 2'-CH_3), 2.76 (3H, s, 6-CH_3), 7.16 (1H, t, *J* = 7.5 Hz, H-5'), 7.21 (1H, d, *J* = 7.5 Hz, H-6'), 7.40-7.44 (2H, m, H-4' and H-4''), 7.52 (1H, t, *J* = 7.8 Hz, H-5''), 7.65 (1H, d, *J* = 15.9 Hz, 5-COC$\underline{\text{H}}$CH), 7.77 (1H, dd, *J* = 7.8, 1.1 Hz, H-3''), 7.91 (1H, d, *J* = 15.9 Hz, 5-COCHC$\underline{\text{H}}$), 8.10 (1H, dd, *J* = 7.8, 1.1 Hz, H-6''), 8.91 (1H, s, H-4). NH and NH_2 not observed. δ_C (100 MHz, $(CD_3)_2SO$) 15.5 (2'-CH_3), 24.3 (6-CH_3), 124.0 (C-3a), 125.4 (C-2'', C-4' and C-6'), 126.5 (C-5'), 128.0 (5-CO$\underline{\text{C}}$HCH), 128.3 (C-5''), 128.6 (C-6''), 129.0 (C-5), 131.7 (C-4), 132.4 (C-4''), 132.5 (C-2'), 133.4 (C-3''), 133.5 (C-3'), 133.8 (C-1''), 140.9 (C-1'), 142.2 (5-COCH$\underline{\text{C}}$H), 145.7 (C-3), 157.8 (C-6), 160.0 (C-7a), 164.1 (2-CONH), 191.9 (5-CO). C-2 not observed. ν_{max} (ATR)/cm^{-1} 3474 (N-H amide), 3396 (N-H amine), 2921 (C-H aromatic), 2853 (C-H alkane), 1658 (C=O carbonyl), 1643 (C=O amide), 1577 (C=C aromatic), 1432 (-C-H bending), 1264 (C-N aromatic), 1064 (C-N aliphatic), 740 (C-Cl), 669 (C-Br). *m/z* (ESI^+): 544 ($^{81}Br^{37}ClMH^+$, 30%), 542 ($^{79}Br^{37}ClMH^+$ and $^{81}Br^{35}ClMHa^+$, 100%), 540 ($^{79}Br^{35}ClMH^+$, 65%). HRMS (ESI^+) found ($^{81}Br^{37}ClMH^+$): 544.0067 $C_{25}H_{20}{}^{81}Br^{37}ClN_3O_2S$ requires 544.0102. Found ($^{79}Br^{37}ClMH^+$ and $^{81}Br^{35}ClMH^+$): 542.0083 $C_{25}H_{20}{}^{79}Br^{37}ClN_3O_2S$ and $C_{25}H_{20}{}^{81}Br^{35}ClN_3O_2S$ requires 542.0122. Found (^{79}Br $^{35}ClMH^+$): 540.0127 $C_{25}H_{20}{}^{79}Br^{35}ClN_3O_2S$ requires 540.0143.

(*E*)-3-Amino-5-(3-(3''-bromophenyl)acryloyl)-*N*-(3'-chloro-2'-methylphenyl)-6-methylthieno[2,3-*b*]pyridine-2-carboxamide 17p

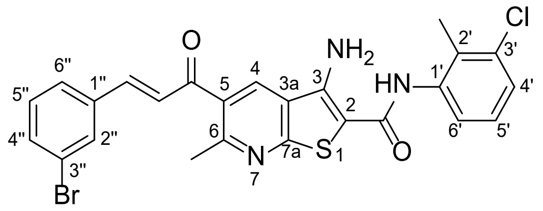

The reaction was carried out following General Procedure **A** using carbonitrile **9p** (0.10 g, 0.28 mmol), chloride **4c** (61.0 mg, 0.28 mmol) and anhydrous sodium carbonate (59.0 mg, 0.56 mmol) in absolute ethanol (5.00 mL) for 48 h to give the *title compound* **17p** (0.14 g, 93%) as a yellow solid. m.p. > 230 °C. δ_H (400 MHz, $(CD_3)_2SO$) 2.25 (3H, s, 2'-CH_3), 2.72 (3H, s, 6-CH_3), 7.22 (1H, t, *J* = 7.6 Hz, H-5'), 7.32 (2H, d, *J* = 7.6 Hz, H-4' and H-6'), 7.39 (2H, br s, NH_2), 7.43 (1H, t, *J* = 7.9 Hz, H-5''), 7.59-7.67 (3H, m, H-4'', 5-COC$\underline{H}$CH and 5-COCHC$\underline{H}$), 7.84 (1H, d, *J* = 7.9 Hz, H-6''), 8.09 (1H, s, H-2''), 8.87 (1H, s, H-4), 9.47 (1H, br s, NH). δ_C (100 MHz, $(CD_3)_2SO$) 15.5 (2'-CH_3), 24.1 (6-CH_3), 97.5 (C-2), 122.4 (C-3''), 123.7 (C-3a), 126.0 (C-4' and C-6'), 126.7 (C-5'), 126.9 (5-CO$\underline{C}$HCH), 127.9 (C-6''), 129.5 (C-5), 131.0 (C-5''), 131.1 (C-2''), 131.7 (C-4), 132.3 (C-2'), 133.3 (C-4''), 133.6 (C-3'), 136.9 (C-1''), 138.7 (C-1'), 143.7 (5-COCH$\underline{C}$H), 146.6 (C-3), 157.9 (C-6), 159.8 (C-7a), 163.9 (2-CONH), 192.7 (5-CO). ν_{max} (ATR)/cm^{-1} 3436 (N-H amide), 3366 (N-H amine), 2923 (C-H aromatic), 2855 (C-H alkane), 1733 (C=O carbonyl), 1640 (C=O amide), 1575 (C=C aromatic), 1432 (-C-H bending), 1269 (C-N aromatic), 1152 (C-O ether), 1069 (C-N aliphatic), 750 (C-Cl), 670 (C-Br). *m/z* (ESI^+): 566 ($^{81}Br^{37}ClMNa^+$, 28%), 564 ($^{79}Br^{37}ClMNa^+$ and $^{81}Br^{35}ClMNa^+$, 100%), 562 ($^{79}Br^{35}ClMNa^+$, 60%). HRMS (ESI^+) found ($^{81}Br^{37}ClMNa^+$): 565.9903 $C_{25}H_{19}{}^{81}Br^{37}ClN_3NaO_2S$ requires 565.9922. Found ($^{79}Br^{37}ClMNa^+$ and $^{81}Br^{35}ClMNa^+$): 563.9935 $C_{25}H_{19}{}^{79}Br^{37}ClN_3NaO_2S$ and $C_{25}H_{19}{}^{81}Br^{35}ClN_3NaO_2S$ requires 563.9941. Found ($^{79}Br\,^{35}ClMNa^+$): 561.9955 $C_{25}H_{19}{}^{79}Br^{35}ClN_3NaO_2S$ requires 561.9962.

(*E*)-3-Amino-5-(3-(4''-bromophenyl)acryloyl)-*N*-(3'-chloro-2'-methylphenyl)-6-methylthieno[2,3-*b*]pyridine-2-carboxamide 17q

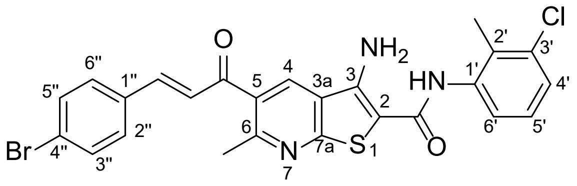

The reaction was carried out following General Procedure **A** using carbonitrile **9q** (0.10 g, 0.28 mmol), chloride **4c** (61.0 mg, 0.28 mmol) and anhydrous sodium carbonate (59.0 mg, 0.56 mmol) in absolute ethanol (5.00 mL) for 48 h to give the *title compound* **17q** (0.12 g, 81%) as a yellow solid. m.p. > 230 °C. δ_H (400 MHz, $(CD_3)_2SO$) 2.25 (3H, s, 2'-CH_3), 2.71 (3H, s, 6-CH_3), 7.18 (1H, t, *J* = 7.8 Hz, H-5'), 7.24 (1H, d, *J* = 7.8 Hz, H-6'), 7.37 (2H, br s, NH_2), 7.38 (1H, d, *J* = 7.8 Hz, H-4'), 7.59 (1H, d, *J* = 15.9 Hz, 5-COC$\underline{H}$CH), 7.63 (1H, d, *J* = 15.9 Hz, 5-COCHC$\underline{H}$), 7.68 (2H, d, *J* = 8.4 Hz, H-3'' and H-5''), 7.80 (2H, d, *J* = 8.4 Hz, H-2'' and H-6''), 8.83 (1H, s, H-4), 9.40 (1H, br s, NH). δ_C (100 MHz, $(CD_3)_2SO$) 15.5 (2'-CH_3), 24.1 (6-CH_3), 123.9 (C-3a), 124.3 (C-4''), 125.5 (C-4' and C-6'), 126.3 (5-CO$\underline{C}$HCH), 126.5 (C-5'), 129.5 (C-5), 130.7 (C-2'' and C-6''), 131.4 (C-4), 132.0 (C-2', C-3'' and C-5''), 133.5 (C-3'), 133.7 (C-1''), 139.1 (C-1'), 144.0 (5-COCH$\underline{C}$H), 145.5 (C-3), 157.4 (C-6), 159.7 (C-7a), 164.2 (2-CONH), 192.8 (5-CO). C-2 not observed. ν_{max} (ATR)/cm^{-1} 3410 (N-H amide), 3274 (N-H amine), 2924 (C-H aromatic), 2856 (C-H alkane), 1735 (C=O carbonyl), 1661 (C=O amide), 1583 (C=C aromatic), 1430 (-C-H bending), 1258 (C-N aromatic), 1065 (C-N aliphatic), 758 (C-Cl), 656 (C-Br). *m/z* (ESI^+): 566 ($^{81}Br^{37}ClMNa^+$, 30%), 564 ($^{79}Br^{37}ClMNa^+$ and $^{81}Br^{35}ClMNa^+$, 100%), 562 ($^{79}Br^{35}ClMNa^+$, 65%). HRMS (ESI^+) found ($^{81}Br^{37}ClMNa^+$): 565.9889 $C_{25}H_{19}{}^{81}Br^{37}ClN_3NaO_2S$ requires 565.9922. Found ($^{79}Br^{37}ClMNa^+$ and $^{81}Br^{35}ClMNa^+$): 563.9915 $C_{25}H_{19}{}^{79}Br^{37}ClN_3NaO_2S$ and $C_{25}H_{19}{}^{81}Br^{35}ClN_3NaO_2S$ requires 563.9941. Found ($^{79}Br^{35}ClMNa^+$): 561.9932 $C_{25}H_{19}{}^{79}Br^{35}ClN_3NaO_2S$ requires 561.9962.

(*E*)-3-Amino-*N*-(3'-chloro-2'-methylphenyl)-5-(3-(3'',4''-dimethoxyphenyl)acryloyl)-6-methylthieno[2,3-*b*]pyridine-2-carboxamide 17r

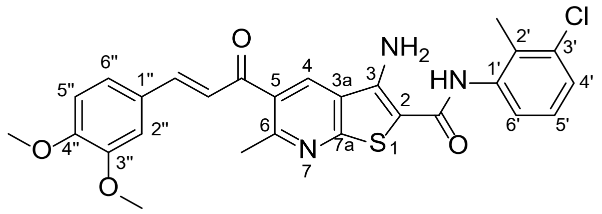

The reaction was carried out following General Procedure **A** using carbonitrile **9r** (0.10 g, 0.29 mmol), chloride **4c** (64.0 mg, 0.29 mmol) and anhydrous sodium carbonate (62.0 mg, 0.59 mmol) in absolute ethanol (5.00 mL) for 48 h to give the *title compound* **17r** (0.12 g, 79%) as a yellow solid. m.p. 160-162 °C. δ_H (400 MHz, $(CD_3)_2SO$) 2.24 (3H, s, 2'-CH_3), 2.67 (3H, s, 6-CH_3), 3.817 (3H, s, 4''-OCH_3), 3.819 (3H, s, 3''-OCH_3), 7.03 (1H, d, *J* = 8.4 Hz, H-5''), 7.22 (1H, t, *J* = 7.5 Hz, H-5'), 7.31 (1H, d, *J* = 7.5 Hz, H-6'), 7.33 (2H, br s, NH_2), 7.34 (1H, d, *J* = 7.5 Hz, H-4'), 7.38 (1H, dd, *J* = 8.4, 2.0 Hz, H-6''), 7.39 (1H, d, *J* = 16.0 Hz, 5-COC<u>H</u>CH), 7.45 (1H, d, *J* = 2.0 Hz, H-2''), 7.54 (1H, d, *J* = 16.0 Hz, 5-COCHC<u>H</u>), 8.76 (1H, s, H-4), 9.45 (1H, br s, NH). δ_C (100 MHz, $(CD_3)_2SO$) 15.5 (2'-CH_3), 23.7 (6-CH_3), 55.6 and 55.7 (4''-OCH_3 and 3''-OCH_3), 111.2 (C-2''), 111.6 (C-5''), 123.7 (C-3a), 123.8 (C-6'' and 5-CO<u>C</u>HCH), 125.9 (C-4' and C-6'), 126.6 (C-5'), 127.1 (C-1''), 130.4 (C-5), 130.9 (C-4), 132.5 (C-2'), 133.6 (C-3'), 138.9 (C-1'), 146.6 (C-3 and 5-COCH<u>C</u>H), 149.0 (C-3''), 151.5 (C-4''), 157.4 (C-6), 159.3 (C-7a), 164.0 (2-CONH), 193.7 (5-CO). C-2 not observed. ν_{max} (ATR)/cm^{-1} 3402 (N-H amide), 3286 (N-H amine), 2924 (C-H aromatic), 2854 (C-H alkane), 1732 (C=O carbonyl), 1650 (C=O amide), 1586 (C=C aromatic), 1424 (-C-H bending), 1263 (C-N aromatic), 1161 (C-O ether), 1068 (C-N aliphatic), 760 (C-Cl). *m/z* (ESI$^+$): 546 (^{37}ClMNa$^+$, 42%), 544 (^{35}ClMNa$^+$, 100%). HRMS (ESI$^+$) found (^{37}ClMNa$^+$): 546.1034 $C_{27}H_{24}{}^{37}ClN_3NaO_4S$ requires 546.1049. Found (^{35}ClMNa$^+$): 544.1057 $C_{27}H_{24}{}^{35}ClN_3NaO_4S$ requires 544.1068.

(*E*)-3-Amino-*N*-(3'-chloro-2'-methylphenyl)-5-(3-(3''-hydroxyphenyl)acryloyl)-6-methylthieno[2,3-*b*]pyridine-2-carboxamide 17s

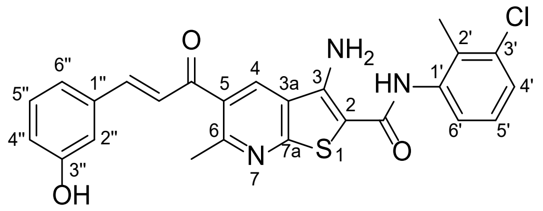

The reaction was carried out following General Procedure **D** using MOM-protected thienopyridine **17g** (20.0 mg, 0.04 mmol) and 6 M HCl (1.0 mL) in methanol (1.0 mL) for 24 h to give the *title compound* **17s** (18.0 mg, quant.) as a yellow solid. m.p. decomp. δ_H (400 MHz, $(CD_3)_2SO$) 2.24 (3H, s, 2'-CH_3), 2.72 (3H, s, 6-CH_3), 7.14 (1H, dt, *J* = 7.2, 1.9 Hz, H-4''), 7.17 (1H, d, *J* = 1.9 Hz, H-2''), 7.22-7.30 (4H, m, H-5', H-5'', H-6' and H-6''), 7.36 (1H, dd, *J* = 7.5, 1.2 Hz, H-4'), 7.40 (2H, br s, NH_2), 7.44 (1H, d, *J* = 15.9 Hz, 5-COC<u>H</u>CH), 7.56 (1H, d, *J* = 15.9 Hz, 5-COCHC<u>H</u>), 8.88 (1H, s, H-4), 9.46 (1H, br s, NH), 9.67 (1H, br s, OH). δ_C (100 MHz, $(CD_3)_2SO$) 15.5 (2'-CH_3), 24.1 (6-CH_3), 96.1 (C-2), 115.1 (C-2''), 118.2 (C-4''), 120.0 (C-6''), 123.6 (C-3a), 125.3 (5-CO<u>C</u>HCH), 126.2 (C-6'), 126.6 (C-4'), 126.7 (C-5'), 129.8 (C-5), 130.0 (C-5''), 131.6 (C-4), 132.6 (C-2'), 133.6 (C-3'), 135.6 (C-1''), 138.1 (C-1'), 145.9 (5-COCH<u>C</u>H), 147.0 (C-3), 157.8 (C-3''), 158.0 (C-6), 159.6 (C-7a), 163.9 (2-CONH), 192.8 (5-CO). ν_{max} (ATR)/cm^{-1} 3440 (N-H amide), 3220 (O-H alcohol, N-H amine), 2925 (C-H aromatic), 2855 (C-H alkane), 1735 (C=O amide), 1519 (C=C aromatic), 1426 (-C-H bending), 1254 (C-N aromatic), 1076 (C-N aliphatic), 1012 (C-O alcohol), 783 (C-Cl). *m/z* (ESI^+): 502 ($^{37}ClMNa^+$, 40%), 500 ($^{35}ClMNa^+$, 100%). HRMS (ESI^+) found ($^{37}ClMNa^+$): 502.0758 $C_{25}H_{20}{}^{37}ClN_3NaO_3S$ requires 502.0785. Found ($^{35}ClMNa^+$): 500.0787 $C_{25}H_{20}{}^{35}ClN_3NaO_3S$ requires 500.0806.

3-Amino-5-(3-(3-methoxyphenyl)propanoyl)-6-methyl-*N*-phenylthieno[2,3-*b*]pyridine-2-carboxamide 18a

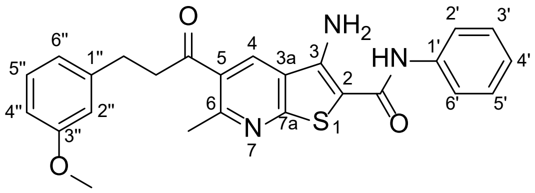

The reaction was carried out following General Procedure **E** using ketone **14a** (0.10 g, 0.23 mmol), 10% palladium on carbon (10.0 mg) in dry methanol (5 mL) for 48 h to give the *title compound* **18a** (0.10 g, quant.) as a yellow solid. m.p. 191-193 °C. δ_H (400 MHz, $(CD_3)_2SO$) 2.70 (3H, s, 6-CH_3), 2.94 (2H, t, *J* = 7.6 Hz, 5-$COCH_2C\underline{H}_2$), 3.40 (2H, t, *J* = 7.6 Hz, 5-$CO\underline{CH_2}$ → COC$\underline{H}_2CH_2$), 3.73 (3H, s, 3"-OCH_3), 6.75-6.78 (1H, m, H-4"), 6.86-6.88 (2H, m, H-2" and H-6"), 7.93 (1H, t, *J* = 7.5 Hz, H-4'), 7.19-7.25 (3H, m, H-3', H-5' and H-5"), 7.62 (2H, d, *J* = 7.5 Hz, H-2' and H-6'), 8.94 (1H, s, H-4). NH and NH_2 not observed. δ_C (100 MHz, $(CD_3)_2SO$) 24.9 (6-CH_3), 29.7 (5-$COCH_2\underline{C}H_2$), 42.0 (5-$CO\underline{C}H_2CH_2$), 54.9 (3"-OCH_3), 96.5 (C-2), 111.4 (C-4"), 114.1 (C-2"), 120.6 (C-6"), 121.1 (C-2' and C-6'), 123.4 (C-4'), 123.7 (C-3a), 128.4 (C-5, C-3' and C-5'), 129.3 (C-5"), 132.1 (C-4), 139.0 (C-1'), 142.7 (C-1"), 147.0 (C-3), 158.4 (C-6), 159.3 (C-3"), 159.9 (C-7a), 163.7 (2-CONH), 201.1 (5-CO). ν_{max} (ATR)/cm^{-1} 3402 (N-H amide), 3266 (N-H amine), 2973 (C-H aromatic), 2833 (C-H alkane), 1685 (C=O carbonyl), 1635 (C=O amide), 1583 (C=C aromatic), 1435 (-C-H bending), 1251 (C-N aromatic), 1153 (C-O ether), 1050 (C-N aliphatic). *m/z* (ESI^+): 468 (MNa^+, 100%). HRMS (ESI^+) found (MNa^+): 468.1347 $C_{25}H_{23}N_3NaO_3S$ requires 468.1352.

3-Amino-5-(3-(3"-methoxyphenyl)propanoyl)-6-methyl-*N*-(*o*-tolyl)thieno[2,3-*b*]pyridine-2-carboxamide 18b

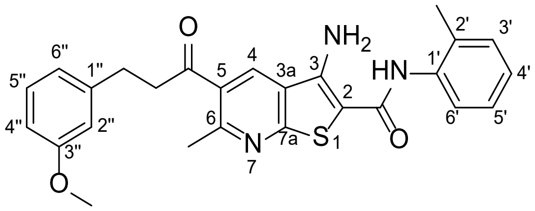

The reaction was carried out following General Procedure **E** using ketone **14b** (50.0 mg, 0.11 mmol), 10% palladium on carbon (10.0 mg) in dry methanol (5 mL) for 48 h to give the crude product, which was purified using flash chromatography (4:1 petroleum ether : ethyl acetate) to give the *title compound* **18b** (39.0 mg, 33%) as a yellow solid. m.p. 164-166 °C. δ_H (400

MHz, $(CD_3)_2SO$) 2.23 (3H, s, 2'-CH_3), 2.71 (3H, s, 6-CH_3), 2.94 (2H, t, *J* = 7.6 Hz, 5-$COCH_2C\underline{H}_2$), 3.41 (2H, t, *J* = 7.6 Hz, 5-$COC\underline{H}_2CH_2$), 3.73 (3H, s, 3"-OCH_3), 6.75-6.78 (1H, m, H-4"), 6.86-6.88 (2H, m, H-2" and H-6"), 7.13-7.23 (3H, m, H-4', H-5' and H-5"), 7.26 (1H, dd, *J* = 7.3, 1.5 Hz, H-3'), 7.31 (1H, dd, *J* = 7.3, 1.5 Hz, H-6'), 7.35 (2H, br s, NH_2), 9.03 (1H, s, H-4), 9.17 (1H, br s, NH). δ_C (100 MHz, $(CD_3)_2SO$) 17.9 (2'-CH_3), 24.9 (6-CH_3), 29.7 (5-$COCH_2\underline{C}H_2$), 42.0 (5-$CO\underline{C}H_2CH_2$), 54.9 (3"-OCH_3), 96.7 (C-2), 111.3 (C-4"), 114.1 (C-2"), 120.6 (C-6"), 123.8 (C-3a), 125.9 (C-4' and C-5'), 126.9 (C-6'), 128.3 (C-5), 129.3 (C-5"), 130.2 (C-3'), 132.1 (C-4), 134.0 (C-2'), 136.3 (C-1'), 142.6 (C-1"), 146.5 (C-3), 158.2 (C-6), 159.3 (C-3"), 159.8 (C-7a), 163.6 (2-CONH), 201.1 (5-CO). ν_{max} (ATR)/cm^{-1} 3405 (N-H amide), 3293 (N-H amine), 2926 (C-H aromatic), 2837 (C-H alkane), 1684 (C=O carbonyl), 1638 (C=O amide), 1582 (C=C aromatic), 1451 (-C-H bending), 1251 (C-N aromatic), 1154 (C-O ether), 1032 (C-N aliphatic). *m/z* (ESI^+): 482 (MNa^+, 100%). HRMS (ESI^+) found (MNa^+): 482.1495 $C_{26}H_{25}N_3NaO_3S$ requires 482.1509.

3-Amino-*N*-(3'-chloro-2'-methylphenyl)-5-(3-(3''-methoxyphenyl)propanoyl)-6-methylthieno[2,3-*b*]pyridine-2-carboxamide 18c

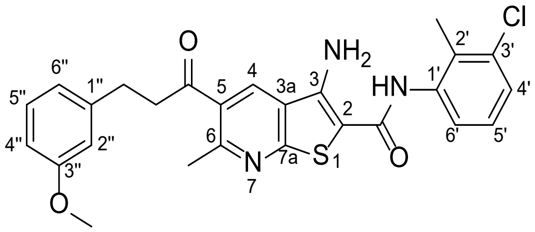

The reaction was carried out following General Procedure **E** using ketone **14c** (0.20 g, 0.41 mmol), 10% palladium on carbon (20.0 mg) in dry methanol (10 mL) for 72 h to give the *title compound* **18c** (68.0 mg, 34%) as a grey solid. m.p. 180-182 °C. δ_H (400 MHz, $(CD_3)_2SO$) 2.24 (3H, s, 2'-CH_3), 2.71 (3H, s, 6-CH_3), 2.94 (2H, t, *J* = 7.6 Hz, 5-$COCH_2C\underline{H}_2$), 3.40 (2H, t, *J* = 7.6 Hz, 5-$COC\underline{H}_2CH_2$), 3.73 (3H, s, 3"-OCH_3), 6.76 (1H, dd, *J* = 7.9, 2.0 Hz, H-4"), 6.85-6.87 (2H, m, H-2" and H-6"), 7.18-7.23 (2H, m, H-5' and H-5"), 7.31 (2H, t, *J* = 8.3 Hz, H-4' and H-6'), 7.37 (2H, br s, NH_2), 9.02 (1H, s, H-4), 9.38 (1H, br s, NH). δ_C (100 MHz, $(CD_3)_2SO$) 15.4 (2'-CH_3), 24.9 (6-CH_3), 29.7 (5-$COCH_2\underline{C}H_2$), 42.0 (5-$CO\underline{C}H_2CH_2$), 54.9 (3"-OCH_3), 111.4 (C-4"), 114.1 (C-2"), 120.6 (C-6"), 123.9 (C-3a), 125.9 (C-4' and C-6'), 126.6 (C-5'), 128.3 (C-5), 129.3 (C-5"), 132.0 (C-2'), 132.2 (C-4), 133.6 (C-3'), 139.3 (C-1'), 142.7 (C-1"), 146.3 (C-3), 158.1 (C-6), 159.3 (C-3"), 159.9 (C-7a), 164.0 (2-CONH), 201.1 (5-CO). C-2 not observed. ν_{max} (ATR)/cm^{-1} 3430 (N-H amide), 3346 (N-H amine), 2922 (C-H aromatic), 2845

(C-H alkane), 1689 (C=O carbonyl), 1632 (C=O amide), 1588 (C=C aromatic), 1477 (-C-H bending), 1262 (C-N aromatic), 1162 (C-O ether), 1037 (C-N aliphatic), 774 (C-Cl). *m/z* (ESI^+): 518 ($^{37}ClMNa^+$, 40%), 516 ($^{35}ClMNa^+$, 100%), 381 (20%). HRMS (ESI^+) found ($^{37}ClMNa^+$): 518.1097 $C_{26}H_{24}{}^{37}ClN_3NaO_3S$ requires 518.1099. Found ($^{35}ClMNa^+$): 516.1113 $C_{26}H_{24}{}^{35}ClN_3NaO_3S$ requires 516.1119.

3-Amino-*N*-(4'-methoxyphenyl)-5-(3-(3''-methoxyphenyl)propanoyl)-6-methylthieno[2,3-*b*]pyridine-2-carboxamide 18e

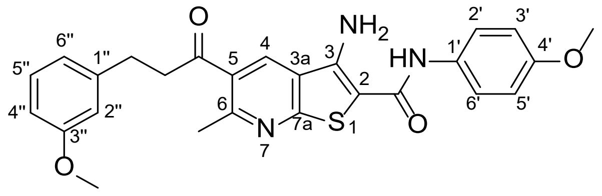

The reaction was carried out following General Procedure **E** using ketone **14e** (0.10 g, 0.21 mmol), 10% palladium on carbon (20.0 mg) in dry methanol (5 mL) for 48 h to give the *title compound* **18e** (89.0 g, 89%) as a yellow solid. m.p. 185-187 °C. δ_H (400 MHz, $(CD_3)_2SO$) 2.71 (3H, s, 6-CH_3), 2.94 (2H, t, *J* = 7.6 Hz, 5-$COCH_2C\underline{H}_2$), 3.40 (2H, t, *J* = 7.6 Hz, 5-$CO C\underline{H}_2CH_2$), 3.73 (3H, s, 3''-OCH_3), 3.74 (3H, s, 4'-OCH_3), 6.76 (1H, dd, *J* = 7.9, 2.0 Hz, H-4''), 6.85-6.91 (4H, m, H-3' and H-5', H-2'' and H-6''), 7.21 (1H, t, *J* = 8.1 Hz, H-5''), 7.40 (2H, br s, NH_2), 7.57 (2H, d, *J* = 9.0 Hz, H-2' and H-6'), 9.02 (1H, s, H-4), 9.36 (1H, br s, NH). δ_C (100 MHz, $(CD_3)_2SO$) 24.9 (6-CH_3), 29.8 (5-$COCH_2\underline{C}H_2$), 42.0 (5-$CO\underline{C}H_2CH_2$), 54.9 (3''-OCH_3), 55.2 (4'-OCH_3), 96.5 (C-2), 111.4 (C-4''), 113.6 (C-3' and C-5'), 114.2 (C-2''), 120.7 (C-6''), 123.0 (C-2' and C-6'), 123.8 (C-3a), 128.4 (C-5), 129.4 (C-5''), 131.9 (C-1'), 132.1 (C-4), 142.7 (C-1''), 146.7 (C-3), 155.6 (C-4'), 158.3 (C-6), 159.4 (C-3''), 159.9 (C-7a), 163.5 (2-CONH), 201.2 (5-CO). ν_{max} (ATR)/cm^{-1} 3428 (N-H amide), 3345 (N-H amine), 2974 (C-H aromatic), 2833 (C-H alkane), 1673 (C=O carbonyl), 1635 (C=O amide), 1581 (C=C aromatic), 1457 (-C-H bending), 1246 (C-N aromatic), 1158 (C-O ether), 1049 (C-N aliphatic). *m/z* (ESI^+): 498 (MNa^+, 100%). HRMS (ESI^+) found (MNa^+): 498.1456 $C_{26}H_{25}N_3NaO_4S$ requires 498.1458.

3-Amino-*N*-(3'-chloro-2'-methylphenyl)-5-(3-(2''-methoxyphenyl)propanoyl)-6-methylthieno[2,3-*b*]pyridine-2-carboxamide 19f

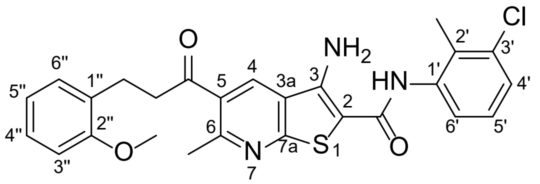

The reaction was carried out following General Procedure **E** using ketone **17f** (35.0 mg, 0.07 mmol), 10% palladium on carbon (7.0 mg) in dry methanol (5 mL) for 72 h to give the *title compound* **19f** (35.0 mg, quant.) as a yellow solid. m.p. > 230 °C. δ_H (400 MHz, $(CD_3)_2SO$) 2.23 (3H, s, 2'-CH_3), 2.71 (3H, s, 6-CH_3), 2.93 (2H, t, J = 7.5 Hz, 5-$COCH_2C\underline{H}_2$), 3.33 (2H, t, J = 7.5 Hz, 5-$COC\underline{H}_2CH_2$), 3.78 (3H, s, 2''-OCH_3), 6.88 (1H, td, J = 7.4, 1.0 Hz, H-5''), 6.96 (1H, d, J = 7.4 Hz, H-3''), 7.20 (1H, t, J = 7.4 Hz, H-4''), 7.21 (1H, d, J = 7.4 Hz, H-6''), 7.22 (1H, t, J = 7.8 Hz, H-5'), 7.28 (1H, dd, J = 7.8, 1.6 Hz, H-6'), 7.35 (1H, dd, J = 7.8, 1.6 Hz, H-4'), 7.40 (2H, br s, NH_2), 9.02 (1H, s, H-4), 9.43 (1H, br s, NH). δ_C (100 MHz, $(CD_3)_2SO$) 15.5 (2'-CH_3), 24.5 (5-$COCH_2\underline{C}H_2$), 24.9 (6-CH_3), 40.5 (5-$CO\underline{C}H_2CH_2$), 55.2 (2''-OCH_3), 96.0 (C-2), 110.6 (C-3''), 120.3 (C-5''), 123.7 (C-3a), 126.2 (C-6'), 126.6 (C-4'), 126.7 (C-5'), 127.5 (C-6''), 128.3 (C-5), 128.6 (C-1''), 129.6 (C-4''), 132.2 (C-4), 132.6 (C-2'), 133.6 (C-3'), 138.0 (C-1'), 147.0 (C-3), 157.1 (C-2''), 158.4 (C-6), 159.9 (C-7a), 163.8 (2-CONH), 201.4 (5-CO). ν_{max} (ATR)/cm^{-1} 3434 (N-H amide), 3319 (N-H amine), 2924 (C-H aromatic), 2853 (C-H alkane), 1690 (C=O carbonyl), 1644 (C=O amide), 1577 (C=C aromatic), 1459 (-C-H bending), 1286 (C-N aromatic), 1177 (C-O ether), 1072 (C-N aliphatic), 750 (C-Cl). *m/z* (ESI^+): 518 ($^{37}ClMNa^+$, 38%), 516 ($^{35}ClMNa^+$, 100%). HRMS (ESI^+) found ($^{37}ClMNa^+$): 518.1087 $C_{26}H_{24}{}^{37}ClN_3NaO_3S$ requires 518.1099. Found ($^{35}ClMNa^+$): 516.1126 $C_{25}H_{24}{}^{35}ClN_3NaO_3S$ requires 516.1119.

3-Amino-*N*-(3'-chloro-2'-methylphenyl)-5-(1-hydroxy-3-(3''-(methoxymethoxy)phenyl)propyl)-6-methylthieno[2,3-*b*]pyridine-2-carboxamide 19g

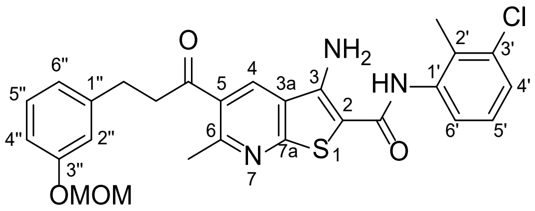

The reaction was carried out following General Procedure **E** using ketone **17g** (40.0 mg, 0.08 mmol), 10% palladium on carbon (8.0 mg) in THF (5 mL) for 72 h and purified with flash chromatography (9:1 petroleum ether, ethyl acetate) to give the *title compound* **19g** (28.0 mg, 70%) as a yellow solid. m.p. 147-149 °C. δ_H (400 MHz, $(CD_3)_2SO$) 2.23 (3H, s, 2'-CH_3), 2.71 (3H, s, 6-CH_3), 2.94 (2H, t, *J* = 7.6 Hz, 5-$COCH_2C\underline{H}_2$), 3.36 (3H, s, $MOMCH_3$), 3.40 (2H, t, *J* = 7.6 Hz, 5-$COC\underline{H}_2CH_2$), 5.16 (2H, s, $MOMCH_2$), 6.86 (1H, ddd, *J* = 8.1, 2.3, 1.0 Hz, H-4''), 6.92-6.95 (2H, m, H-2'' and H-6''), 7.22 (1H, t, *J* = 7.9 Hz, H-5''), 7.23 (1H, t, *J* = 7.7 Hz, H-5'), 7.28 (1H, dd, *J* = 7.7, 1.4 Hz, H-6'), 7.35 (1H, dd, *J* = 7.7, 1.4 Hz, H-4'), 7.39 (2H, br s, NH_2), 9.04 (1H, s, H-4), 9.44 (1H, br s, NH). δ_C (100 MHz, $(CD_3)_2SO$) 15.4 (2'-CH_3), 24.9 (6-CH_3), 29.6 (5-$COCH_2\underline{C}H_2$), 41.9 (5-$CO\underline{C}H_2CH_2$), 55.5 ($MOMCH_3$), 93.8 ($MOMCH_2$), 96.0 (C-2), 113.6 (C-4''), 116.4 (C-2''), 121.8 (C-6''), 123.7 (C-3a), 126.2 (C-6'), 126.6 (C-4'), 126.7 (C-5'), 128.4 (C-5), 129.3 (C-5''), 132.2 (C-4), 132.5 (C-2'), 133.6 (C-3'), 138.0 (C-1'), 142.7 (C-1''), 146.9 (C-3), 156.9 (C-3''), 158.4 (C-6), 159.9 (C-7a), 163.8 (2-CONH), 201.1 (5-CO). ν_{max} (ATR)/cm^{-1} 3429 (N-H amide), 3346 (N-H amine), 2921 (C-H aromatic), 2851 (C-H alkane), 1689 (C=O carbonyl), 1632 (C=O amide), 1587 (C=C aromatic), 1443 (-C-H bending), 1255 (C-N aromatic), 1149 (C-O ether), 1087 (C-N aliphatic), 775 (C-Cl). *m/z* (ESI^+): 548 ($^{37}ClMNa^+$, 40%), 546 ($^{35}ClMNa^+$, 100%). HRMS (ESI^+) found ($^{37}ClMNa^+$): 548.1210 $C_{27}H_{26}{}^{37}ClN_3NaO_4S$ requires 548.1205. Found ($^{35}ClMNa^+$): 546.1229 $C_{27}H_{26}{}^{35}ClN_3NaO_4S$ requires 546.1225.

3-Amino-*N*-(3'-chloro-2'-methylphenyl)-6-methyl-5-(3-(2''-(trifluoromethyl)phenyl)propanoyl)thieno[2,3-*b*]pyridine-2-carboxamide 19h

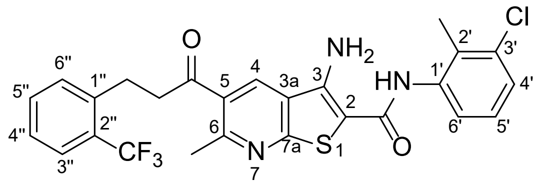

The reaction was carried out following General Procedure **E** using ketone **17h** (0.19 g, 0.60 mmol), 10% palladium on carbon (38.0 mg) in dry THF (15 mL) for 72 h to give the *title compound* **19h** (30.0 mg, 79%) as a yellow solid. m.p. 218-220 °C. δ_H (400 MHz, $(CD_3)_2SO$) 2.23 (3H, s, 2'-CH_3), 2.76 (3H, s, 6-CH_3), 3.15 (2H, t, J = 7.5 Hz, 5-$COCH_2C\underline{H}_2$), 3.44 (2H, t, J = 7.5 Hz, 5-$COC\underline{H}_2CH_2$), 7.23 (1H, t, J = 7.8 Hz, H-5'), 7.28 (1H, d, J = 7.8 Hz, H-6'), 7.35 (1H, d, J = 7.8 Hz, H-4'), 7.39 (2H, br s, NH_2), 7.45 (1H, t, J = 7.5 Hz, H-4''), 7.62 (1H, d, J = 7.5 Hz, H-6''), 7.66 (1H, t, J = 7.5 Hz, H-5''), 7.72 (1H, d, J = 7.5 Hz, H-3''), 9.05 (1H, s, H-4), 9.44 (1H, br s, NH). δ_C (100 MHz, $(CD_3)_2SO$) 15.4 (2'-CH_3), 25.1 (6-CH_3), 26.1 (5-$COCH_2\underline{C}H_2$), 41.8 (5-$CO\underline{C}H_2CH_2$), 96.1 (C-2), 123.7 (C-3a), 124.6 (q, $^1J_{F/C}$ = 273.5 Hz, CF_3), 125.8 (q, $^3J_{F/C}$ = 5.7 Hz, C-3''), 126.2 (C-6'), 126.6 (C-4'), 126.7 (C-4'' and C-5'), 127.1 (q, $^2J_{F/C}$ = 29.4 Hz, C-2''), 127.9 (C-5), 131.3 (C-6''), 132.3 (C-4), 132.5 (C-2'), 132.6 (C-5''), 133.6 (C-3'), 138.0 (C-1'), 139.8 (C-1''), 146.9 (C-3), 158.6 (C-6), 160.1 (C-7a), 163.8 (2-CONH), 200.1 (5-CO). ν_{max} (ATR)/cm^{-1} 3424 (N-H amide), 3324 (N-H amine), 2923 (C-H aromatic), 2850 (C-H alkane), 1691 (C=O carbonyl), 1643 (C=O amide), 1577 (C=C aromatic), 1459 (-C-H bending), 1308 (C-F), 1249 (C-N aromatic), 1101 (C-N aliphatic), 766 (C-Cl). *m/z* (ESI^+): 534 ($^{37}ClMH^+$, 40%), 532 ($^{35}ClMH^+$, 100%). HRMS (ESI^+) found ($^{37}ClMH^+$): 534.1058 $C_{26}H_{22}{}^{37}ClF_3N_3O_2S$ requires 534.1047. Found ($^{35}ClMH^+$): 532.1067 $C_{26}H_{22}{}^{35}ClF_3N_3O_2S$ requires 532.1068.

3-Amino-*N*-(3'-chloro-2'-methylphenyl)-5-(3-(2''-fluorophenyl)propanoyl)-6-methylthieno[2,3-*b*]pyridine-2-carboxamide 19i

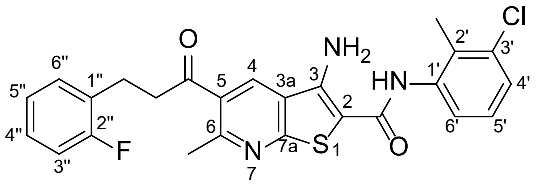

The reaction was carried out following General Procedure **E** using ketone **17i** (31.0 mg, 0.06 mmol), 10% palladium on carbon (6.2 mg) in dry methanol (3 mL) for 72 h to give the *title compound* **19i** (30.0 mg, 97%) as a yellow solid. m.p. 210-212 °C. δ_H (400 MHz, $(CD_3)_2SO$) 2.23 (3H, s, 2'-CH_3), 2.72 (3H, s, 6-CH_3), 3.00 (2H, t, J = 7.5 Hz, 5-$COCH_2C\underline{H}_2$), 3.41 (2H, t, J = 7.5 Hz, 5-$COC\underline{H}_2CH_2$), 7.13-7.19 (2H, m, H-3'' and H-5''), 7.23 (1H, t, J = 7.6 Hz, H-5'), 7.26-7.30 (2H, m, H-6' and H-4''), 7.35 (1H, dd, J = 7.6, 1.4 Hz, H-4'), 7.37-7.41 (3H, m, H-6'' and NH_2), 9.05 (1H, s, H-4), 9.44 (1H, br s, NH). δ_C (100 MHz, $(CD_3)_2SO$) 15.5 (2'-CH_3), 22.8 (5-$COCH_2\underline{C}H_2$), 25.0 (6-CH_3), 40.4 (5-$CO\underline{C}H_2CH_2$), 96.0 (C-2), 115.1 (d, $^2J_{F/C}$ = 22.1 Hz, C-3''), 123.7 (C-3a), 124.4 (d, $^4J_{F/C}$ = 2.9 Hz, C-5''), 126.2 (C-6'), 126.6 (C-4'), 126.7 (C-5'), 127.6 (d, $^2J_{F/C}$ = 15.4 Hz, C-1''), 128.1 (C-5), 128.2 (d, $^3J_{F/C}$ = 8.0 Hz, C-4''), 130.9 (d, $^3J_{F/C}$ = 5.1 Hz, C-6''), 132.3 (C-4), 132.5 (C-2'), 133.6 (C-3'), 138.0 (C-1'), 146.9 (C-3), 158.5 (C-6), 160.0 (C-7a), 160.5 (d, $^1J_{F/C}$ = 243.2 Hz, C-2''), 163.8 (2-CONH), 200.6 (5-CO). ν_{max} (ATR)/cm^{-1} 3420 (N-H amide), 3316 (N-H amine), 2925 (C-H aromatic), 2851 (C-H alkane), 1684 (C=O carbonyl), 1646 (C=O amide), 1578 (C=C aromatic), 1460 (-C-H bending), 1249 (C-N aromatic), 1226 (C-F), 1068 (C-N aliphatic), 756 (C-Cl). *m/z* (ESI^+): 506 ($^{37}ClNa^+$, 40%), 504 ($^{35}ClNa^+$, 100%). HRMS (ESI^+) found ($^{37}ClNa^+$): 506.0884 $C_{25}H_{21}{}^{37}ClFN_3NaO_2S$ requires 506.0898. Found ($^{35}ClNa^+$): 504.0909 $C_{25}H_{21}{}^{35}ClFN_3NaO_2S$ requires 504.0919.

3-Amino-*N*-(3'-chloro-2'-methylphenyl)-5-(3-(3''-fluorophenyl)propanoyl)-6-methylthieno[2,3-*b*]pyridine-2-carboxamide 19j

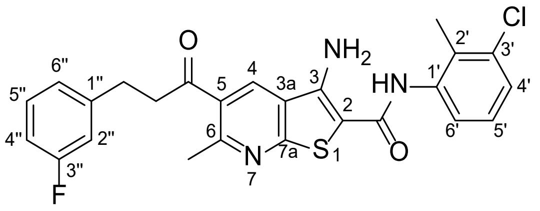

The reaction was carried out following General Procedure **E** using ketone **17j** (35.0 mg, 0.07 mmol), 10% palladium on carbon (7.0 mg) in dry methanol (5 mL) for 48 h to give the *title compound* **19j** (27.0 mg, 77%) as a yellow solid. m.p. 200-202 °C. δ_H (400 MHz, $(CD_3)_2SO$) 2.24 (3H, s, 2'-CH_3), 2.72 (3H, s, 6-CH_3), 2.99 (2H, t, *J* = 7.5 Hz, 5-$COCH_2C\underline{H}_2$), 3.43 (2H, t, *J* = 7.5 Hz, 5-$COC\underline{H}_2CH_2$), 7.02 (1H, td, *J* = 8.2, 2.7 Hz, H-4''), 7.14-7.18 (2H, m, H-2'' and H-6''), 7.23 (1H, t, *J* = 7.8 Hz, H-5'), 7.28 (1H, d, *J* = 7.8 Hz, H-6'), 7.32-7.37 (2H, m, H-4' and H-5''), 7.39 (2H, br s, NH_2), 9.04 (1H, s, H-4), 9.44 (1H, br s, NH). δ_C (100 MHz, $(CD_3)_2SO$) 15.5 (2'-CH_3), 24.9 (6-CH_3), 29.3 (5-$COCH_2\underline{C}H_2$), 41.6 (5-$CO\underline{C}H_2CH_2$), 112.7 (d, $^2J_{F/C}$ = 20.6 Hz, C-4''), 115.2 (d, $^2J_{F/C}$ = 20.6 Hz, C-2''), 123.8 (C-3a), 124.6 (d, $^4J_{F/C}$ = 2.3 Hz, C-6''), 126.2 (C-6'), 126.6 (C-4'), 126.7 (C-5'), 128.2 (C-5), 130.2 (d, $^3J_{F/C}$ = 8.9 Hz, C-5''), 132.2 (C-4), 132.5 (C-2'), 133.6 (C-3'), 138.1 (C-1'), 144.1 (d, $^3J_{F/C}$ = 7.9 Hz, C-1''), 146.8 (C-3), 158.4 (C-6), 159.9 (C-7a), 162.3 (d, $^1J_{F/C}$ = 243.3 Hz, C-3''), 163.8 (2-CONH), 200.8 (5-CO). C-2 not observed. ν_{max} (ATR)/cm^{-1} 3425 (N-H amide), 3305 (N-H amine), 2923 (C-H aromatic), 2852 (C-H alkane), 1692 (C=O carbonyl), 1644 (C=O amide), 1576 (C=C aromatic), 1461 (-C-H bending), 1249 (C-N aromatic), 1230 (C-F), 1028 (C-N aliphatic), 783 (C-Cl). *m/z* (ESI^+): 506 ($^{37}ClMNa^+$, 35%), 504 ($^{35}ClMNa^+$, 100%). HRMS (ESI^+) found ($^{37}ClMNa^+$): 506.0884 $C_{25}H_{21}{}^{37}ClFN_3NaO_2S$ requires 506.0898. Found ($^{35}ClMNa^+$): 504.0904 $C_{25}H_{21}{}^{35}ClFN_3NaO_2S$ requires 504.0919.

3-Amino-*N*-(3'-chloro-2'-methylphenyl)-5-(3-(4''-fluorophenyl)propanoyl)-6-methylthieno[2,3-*b*]pyridine-2-carboxamide 19k

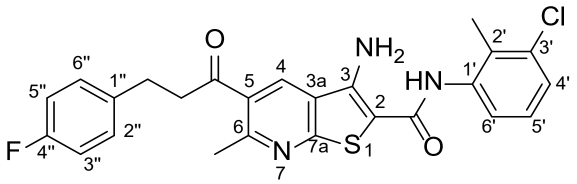

The reaction was carried out following General Procedure **E** using ketone **17k** (32.0 mg, 0.07 mmol), 10% palladium on carbon (6.4 mg) in dry THF (5 mL) for 72 h to give the *title compound* **19k** (20.0 mg, 63%) as a yellow solid. m.p. 200-202 °C. δ_H (400 MHz, $(CD_3)_2SO$) 2.25 (3H, s, 2'-CH_3), 2.73 (3H, s, 6-CH_3), 2.98 (2H, t, J = 7.5 Hz, 5-$COCH_2C\underline{H}_2$), 3.42 (2H, t, J = 7.5 Hz, 5-$COC\underline{H}_2CH_2$), 7.14 (2H, t, J = 8.9 Hz, H-3'' and H-5''), 7.25 (1H, t, J = 7.7 Hz, H-5'), 7.29 (1H, dd, J = 7.7, 1.4 Hz, H-6'), 7.33-7.38 (3H, m, H-4', H-2'' and H-6''), 7.41 (2H, br s, NH_2), 9.05 (1H, s, H-4), 9.45 (1H, br s, NH). δ_C (100 MHz, $(CD_3)_2SO$) 15.4 (2'-CH_3), 24.9 (6-CH_3), 28.8 (5-$COCH_2\underline{C}H_2$), 42.1 (5-$CO\underline{C}H_2CH_2$), 96.1 (C-2), 115.0 (d, $^2J_{F/C}$ = 20.8 Hz, C-3'' and C-5''), 123.7 (C-3a), 126.2 (C-6'), 126.6 (C-4'), 126.7 (C-5'), 128.3 (C-5), 130.2 (d, $^3J_{F/C}$ = 8.0 Hz, C-2'' and C-6''), 132.2 (C-4), 132.5 (C-2'), 133.6 (C-3'), 137.2 (d, $^4J_{F/C}$ = 2.7 Hz, C-1''), 138.0 (C-1'), 146.9 (C-3), 158.4 (C-6), 159.9 (C-7a), 160.7 (d, $^1J_{F/C}$ = 243.2 Hz, C-4''), 163.8 (2-CONH), 201.0 (5-CO). ν_{max} (ATR)/cm^{-1} 3427 (N-H amide), 3328 (N-H amine), 2921 (C-H aromatic), 2855 (C-H alkane), 1688 (C=O carbonyl), 1644 (C=O amide), 1578 (C=C aromatic), 1430 (-C-H bending), 1250 (C-N aromatic), 1213 (C-F), 1066 (C-N aliphatic), 781 (C-Cl). *m/z* (ESI$^+$): 506 (^{37}ClMNa$^+$, 40%), 504 (^{35}ClMNa$^+$, 100%). HRMS (ESI$^+$) found (^{37}ClMNa$^+$): 506.0893 $C_{25}H_{21}{}^{37}ClFN_3NaO_2S$ requires 506.0898. Found (^{35}ClMNa$^+$): 504.0923 $C_{25}H_{21}{}^{35}ClFN_3NaO_2S$ requires 504.0919.

3-Amino-*N*-(3'-chloro-2'-methylphenyl)-5-(3-(2''-chlorophenyl)propanoyl)-6-methylthieno[2,3-*b*]pyridine-2-carboxamide 19l

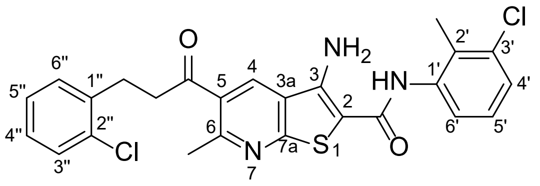

The reaction was carried out following General Procedure **E** using ketone **17l** (29.0 mg, 0.06 mmol), 10% palladium on carbon (5.8 mg) in dry methanol (5 mL) for 72 h to give the *title compound* **19l** (12.0 mg, 41%) as a yellow solid. m.p. > 230 °C. δ_H (400 MHz, $(CD_3)_2SO$) 2.23 (3H, s, 2'-CH_3), 2.74 (3H, s, 6-CH_3), 3.08 (2H, t, *J* = 7.6 Hz, 5-$COCH_2C\underline{H}_2$), 3.41 (2H, t, *J* = 7.6 Hz, 5-$COC\underline{H}_2CH_2$), 7.23 (1H, t, *J* = 7.8 Hz, H-5'), 7.26-7.36 (4H, m, H-4', H-4'', H-5'' and H-6'), 7.40 (2H, br s, NH_2), 7.45 (2H, dd, *J* = 7.7, 1.5 Hz, H-3'' and H-6''), 9.06 (1H, s, H-4), 9.44 (1H, br s, NH). δ_C (100 MHz, $(CD_3)_2SO$) 15.5 (2'-CH_3), 25.0 (6-CH_3), 27.4 (5-$COCH_2\underline{C}H_2$), 40.1 (5-$CO\underline{C}H_2CH_2$), 96.0 (C-2), 123.7 (C-3a), 126.2 (C-6'), 126.6 (C-4'), 126.7 (C-5'), 127.4 (C-5''), 128.1 (C-4''), 128.4 (C-5), 129.2 (C-3''), 130.7 (C-6''), 132.3 (C-4), 132.6 (C-2'), 133.0 (C-2''), 133.6 (C-3'), 138.0 (C-1'), 138.4 (C-1''), 147.0 (C-3), 158.5 (C-6), 160.0 (C-7a), 163.8 (2-CONH), 200.5 (5-CO). ν_{max} (ATR)/cm^{-1} 3424 (N-H amide), 3320 (N-H amine), 2919 (C-H aromatic), 2850 (C-H alkane), 1691 (C=O carbonyl), 1643 (C=O amide), 1575 (C=C aromatic), 1459 (-C-H bending), 1248 (C-N aromatic), 1070 (C-N aliphatic), 783 (C-Cl), 745 (C-Cl). *m/z* (ESI^+): 524 ($^{37}Cl_2MNa^+$, 13%), 522 ($^{37}Cl^{35}ClMNa^+$, 65%), 520 ($^{35}Cl_2MNa^+$, 100%). HRMS (ESI^+) found ($^{37}Cl_2MNa^+$): 524.0559 $C_{25}H_{21}{}^{37}Cl_2N_3NaO_2S$ requires 524.0579. Found ($^{35}Cl^{37}ClMNa^+$): 522.0576 $C_{25}H_{21}{}^{35}Cl^{37}ClN_3NaO_2S$ requires 522.0599. Found ($^{35}Cl_2MNa^+$): 520.0616 $C_{25}H_{21}{}^{35}Cl_2N_3NaO_2S$ requires 520.0624.

3-Amino-*N*-(3'-chloro-2'-methylphenyl)-5-(3-(3''-chlorophenyl)propanoyl)-6-methylthieno[2,3-*b*]pyridine-2-carboxamide 19m

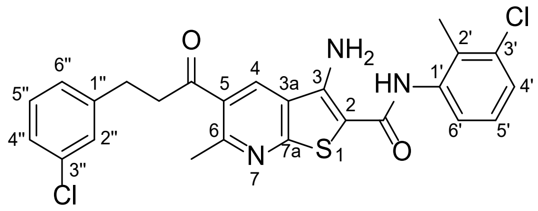

The reaction was carried out following General Procedure **E** using ketone **17m** (36.0 mg, 0.08 mmol), 10% palladium on carbon (8.0 mg) in dry methanol (4 mL) and THF (1 mL) for 48 h to give the *title compound* **19m** (36.0 mg, quant.) as a yellow solid. m.p. 161-163 °C. δ_H (400 MHz, $(CD_3)_2SO$) 2.24 (3H, s, 2'-CH_3), 2.71 (3H, s, 6-CH_3), 2.98 (2H, t, J = 7.5 Hz, 5-$COCH_2C\underline{H}_2$), 3.43 (2H, t, J = 7.5 Hz, 5-$COC\underline{H}_2CH_2$), 7.16 (1H, t, J = 7.3 Hz, H-5'), 7.21 (1H, d, J = 7.3 Hz, H-6'), 7.26 (1H, d, J = 7.3 Hz, H-4'), 7.27-7.29 (1H, m, H-4''), 7.28 (2H, br s, NH_2), 7.33 (1H, t, J = 7.5 Hz, H-5''), 7.38-7.40 (2H, m, H-2'' and H-6''), 8.98 (1H, s, H-4), 9.45 (1H, br s, NH). δ_C (100 MHz, $(CD_3)_2SO$) 15.4 (2'-CH_3), 24.9 (6-CH_3), 29.3 (5-$COCH_2\underline{C}H_2$), 41.7 (5-$CO\underline{C}H_2CH_2$), 124.1 (C-3a), 125.9 (C-4', C-6' and C-6''), 126.5 (C-5'), 127.2 (C-4''), 128.0 (C-5), 128.3 (C-2''), 130.1 (C-5''), 131.9 (C-4), 132.7 (C-3''), 133.0 (C-2'), 133.4 (C-3'), 138.2 (C-1'), 143.8 (C-1''), 145.2 (C-3), 157.6 (C-6), 160.0 (C-7a), 164.2 (2-CONH), 200.9 (5-CO). C-2 not observed. ν_{max} (ATR)/cm^{-1} 3424 (N-H amide), 3318 (N-H amine), 2924 (C-H aromatic), 2853 (C-H alkane), 1684 (C=O carbonyl), 1649 (C=O amide), 1575 (C=C aromatic), 1460 (-C-H bending), 1249 (C-N aromatic), 1063 (C-N aliphatic), 784 (C-Cl), 699 (C-Cl). *m/z* (ESI^+): 524 ($^{37}Cl_2MNa^+$, 10%), 522 ($^{37}Cl^{35}ClMNa^+$, 60%), 520 ($^{35}Cl_2MNa^+$, 100%). HRMS (ESI^+) found ($^{37}Cl_2MNa^+$): 524.0568 $C_{25}H_{21}{}^{37}Cl_2N_3NaO_2S$ requires 524.0579. Found ($^{35}Cl^{37}ClMNa^+$): 522.0591 $C_{25}H_{21}{}^{35}Cl^{37}ClN_3NaO_2S$ requires 522.0599. Found ($^{35}Cl_2MNa^+$): 520.0614 $C_{25}H_{21}{}^{35}Cl_2N_3NaO_2S$ requires 520.0624.

3-Amino-*N*-(3'-chloro-2'-methylphenyl)-5-(3-(4''-chlorophenyl)propanoyl)-6-methylthieno[2,3-*b*]pyridine-2-carboxamide 19n

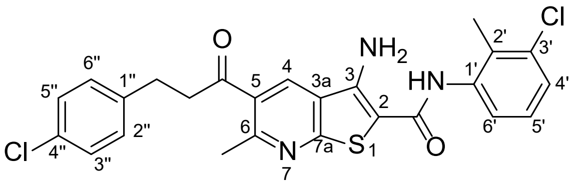

The reaction was carried out following General Procedure **E** using ketone **17n** (38.0 mg, 0.08 mmol), 10% palladium on carbon (7.6 mg) in dry methanol (5 mL) for 72 h to give the *title compound* **19n** (38.0 mg, quant.) as a yellow solid. m.p. 224-226 °C. δ_H (400 MHz, $(CD_3)_2SO$) 2.23 (3H, s, 2'-CH_3), 2.71 (3H, s, 6-CH_3), 2.97 (2H, t, *J* = 7.5 Hz, 5-$COCH_2C\underline{H}_2$), 3.41 (2H, t, *J* = 7.5 Hz, 5-$COC\underline{H}_2CH_2$), 7.23 (1H, t, *J* = 7.8 Hz, H-5'), 7.28 (1H, dd, *J* = 7.8, 1.4 Hz, H-6'), 7.32-7.37 (5H, m, H-4', H-3'' and H-5'', H-2'' and H-6''), 7.39 (2H, br s, NH_2), 9.03 (1H, s, H-4), 9.44 (1H, br s, NH). δ_C (100 MHz, $(CD_3)_2SO$) 15.4 (2'-CH_3), 24.9 (6-CH_3), 28.9 (5-$COCH_2\underline{C}H_2$), 41.8 (5-$CO\underline{C}H_2CH_2$), 96.1 (C-2), 123.7 (C-3a), 126.2 (C-6'), 126.6 (C-4'), 126.7 (C-5'), 128.2 (C-3'' and C-5''), 128.3 (C-5), 130.3 (C-2'' and C-6''), 130.3 (C-4''), 132.2 (C-4), 132.5 (C-2'), 133.6 (C-3'), 138.0 (C-1'), 140.2 (C-1''), 146.9 (C-3), 158.4 (C-6), 159.9 (C-7a), 163.8 (2-CONH), 200.9 (5-CO). ν_{max} (ATR)/cm^{-1} 3431 (N-H amide), 3329 (N-H amine), 2927 (C-H aromatic), 2854 (C-H alkane), 1687 (C=O carbonyl), 1651 (C=O amide), 1579 (C=C aromatic), 1427 (-C-H bending), 1250 (C-N aromatic), 1067 (C-N aliphatic), 782 (C-Cl), 753 (C-Cl). *m/z* (ESI^+): 524 ($^{37}Cl_2MNa^+$, 5%), 522 ($^{37}Cl^{35}ClMNa^+$, 20%), 520 ($^{35}Cl_2MNa^+$, 33%), 480 (100%). HRMS (ESI^+) found ($^{37}Cl_2MNa^+$): 524.0574 $C_{25}H_{21}{}^{37}Cl_2N_3NaO_2S$ requires 524.0579. Found ($^{35}Cl^{37}ClMNa^+$): 522.0587 $C_{25}H_{21}{}^{35}Cl^{37}ClN_3NaO_2S$ requires 522.0599. Found ($^{35}Cl_2MNa^+$): 520.0610 $C_{25}H_{21}{}^{35}Cl_2N_3NaO_2S$ requires 520.0624.

3-Amino-*N*-(3'-chloro-2'-methylphenyl)-5-(1-hydroxy-3-(3''-hydroxyphenyl)propyl)-6-methylthieno[2,3-*b*]pyridine-2-carboxamide 19o

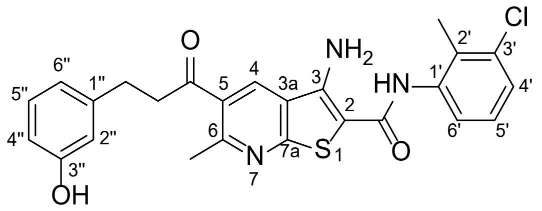

The reaction was carried out following General Procedure **D** using MOM-protected thienopyridine **19g** (11.0 mg, 0.02 mmol) and 6 M HCl (1.0 mL) in methanol (1.0 mL) for 24 h to give the *title compound* **19o** (10.0 mg, quant.) as a yellow solid. m.p. 180-182 °C. δ_H (400 MHz, $(CD_3)_2SO$) 2.23 (3H, s, 2'-CH_3), 2.71 (3H, s, 6-CH_3), 2.88 (2H, t, *J* = 7.6 Hz, 5-$COCH_2C\underline{H}_2$), 3.37 (2H, t, *J* = 7.6 Hz, 5-$COC\underline{H}_2CH_2$), 6.60 (1H, dd, *J* = 7.6, 1.8 Hz, H-4''), 6.68 (1H, d, *J* = 1.8 Hz, H-2''), 6.70 (1H, d, *J* = 7.6 Hz, H-6''), 7.08 (1H, t, *J* = 7.6 Hz, H-5''), 7.23 (1H, t, *J* = 7.8 Hz, H-5'), 7.28 (1H, dd, *J* = 7.8, 1.5 Hz, H-6'), 7.35 (1H, dd, *J* = 7.8, 1.5 Hz, H-4'), 7.40 (2H, br s, NH_2), 9.04 (1H, s, H-4), 9.26 (1H, br s, OH), 9.44 (1H, br s, NH). δ_C (100 MHz, $(CD_3)_2SO$) 15.5 (2'-CH_3), 24.9 (6-CH_3), 29.7 (5-$COCH_2\underline{C}H_2$), 42.0 (5-$CO\underline{C}H_2CH_2$), 96.0 (C-2), 112.9 (C-4''), 115.3 (C-2''), 119.0 (C-6''), 123.7 (C-3a), 126.2 (C-6'), 126.6 (C-4'), 126.7 (C-5'), 128.4 (C-5), 129.3 (C-5''), 132.1 (C-4), 132.6 (C-2'), 133.6 (C-3'), 138.1 (C-1'), 142.4 (C-1''), 146.9 (C-3), 157.4 (C-3''), 158.5 (C-6), 159.9 (C-7a), 163.8 (2-CONH), 201.1 (5-CO). ν_{max} (ATR)/cm^{-1} 3320 (very broad N-H amide, O-H alcohol, and N-H amine), 2971 (C-H aromatic), 2845 (C-H alkane), 1739 (C=O carbonyl), 1683 (C=O amide), 1579 (C=C aromatic), 1462 (-C-H bending), 1231 (C-N aromatic), 1071 (C-N aliphatic), 781 (C-Cl). *m/z* (ESI$^+$): 504 (^{37}ClMNa$^+$, 40%), 502 (^{35}ClMNa$^+$, 100%). HRMS (ESI$^+$) found (^{37}ClMNa$^+$): 504.0914 $C_{25}H_{22}{}^{37}ClN_3NaO_3S$ requires 504.0942. Found (^{35}ClMNa$^+$): 502.0963 $C_{25}H_{22}{}^{35}ClN_3NaO_3S$ requires 502.0963.

(*E*)-3-Amino-5-(1-hydroxy-3-(3''-methoxyphenyl)allyl)-6-methyl-*N*-phenylthieno[2,3-*b*]pyridine-2-carboxamide 20a

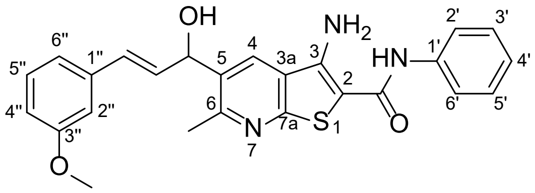

The reaction was carried out following General Procedure **F** using ketone **14a** (20.0 mg, 0.04 mmol), $NaBH_4$ (1.7 mg, 0.04 mmol), and anhydrous $CeCl_3$ (10.8 mg, 0.04 mmol) in dry 2:1 THF:MeOH (5 mL) for 10 min to give the *title compound* **20a** (10.0 mg, 50%) as a yellow solid. m.p. decomp. δ_H (400 MHz, $(CD_3)_2SO$) 2.66 (3H, s, 6-CH_3), 3.75 (3H, s, 3"-OCH_3), 5.51-5.54 (1H, m, C<u>H</u>OH), 5.87 (1H, d, *J* = 3.9 Hz, CHO<u>H</u>), 6.45 (1H, dd, *J* = 15.8, 6.0 Hz, 5-CHOHC<u>H</u>CH), 6.66 (1H, d, *J* = 15.8 Hz, 5-CHOHCHC<u>H</u>), 6.81 (1H, dd, *J* = 8.0, 2.6 Hz, H-4"), 7.01-7.08 (3H, m, H-4', H-2" and H-6"), 7.23 (1H, t, *J* = 7.6 Hz, H-5"), 7.31 (2H, t, *J* = 7.6 Hz, H-3' and H-5'), 7.42 (2H, br s, NH_2), 7.68 (2H, d, *J* = 7.6 Hz, H-2' and H-6'), 8.55 (1H, s, H-4), 9.36 (1H, br s, NH). δ_C (100 MHz, $(CD_3)_2SO$) 22.6 (6-CH_3), 55.0 (3"-OCH_3), 69.6 (5-CHOH), 95.8 (C-2), 111.5 (C-2"), 113.5 (C-4"), 119.0 (C-6"), 121.1 (C-4'), 124.5 (C-3a), 128.3 (C-2' and C-6'), 128.4 (C-3' and C-5'), 128.8 (C-4), 129.4 (5-CHOHCH<u>C</u>H), 129.6 (C-5"), 131.8 (5-CHOH<u>C</u>HCH), 134.1 (C-5), 137.9 (C-1"), 139.0 (C-1'), 147.4 (C-3), 156.6 (C-7a), 157.3 (C-6), 159.5 (C-3"), 164.1 (2-CONH). ν_{max} (ATR)/cm^{-1} 3424 (N-H amide), 3328 (O-H alcohol, N-H amine), 2921 (C-H aromatic), 2853 (C-H alkane), 1590 (C=O amide), 1525 (C=C aromatic), 1437 (-C-H bending), 1254 (C-N aromatic), 1154 (C-O ether), 1037 (C-N aliphatic). *m/z* (ESI^+): 468 (MNa^+, 100%). HRMS (ESI^+) found (MNa^+): 468.1338 $C_{25}H_{23}N_3NaO_3S$ requires 468.1352.

(*E*)-3-Amino-5-(1-hydroxy-3-(3''-methoxyphenyl)allyl)-6-methyl-*N*-(*o*-tolyl)thieno[2,3-*b*]pyridine-2-carboxamide 20b

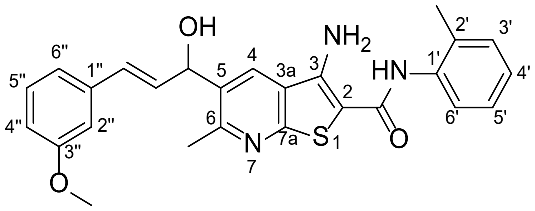

The reaction was carried out following General Procedure **F** using ketone **14b** (34.0 mg, 0.07 mmol), $NaBH_4$ (2.8 mg, 0.07 mmol), and anhydrous $CeCl_3$ (20.0 mg, 0.08 mmol) in dry 2:1

THF:MeOH (3 mL) for 10 min to give the *title compound* **20b** (24.0 mg, 71%) as a yellow solid. m.p. decomp. δ_H (400 MHz, $(CD_3)_2SO$) 2.22 (3H, s, 2'-CH_3), 2.66 (3H, s, 6-CH_3), 3.75 (3H, s, 3''-OCH_3), 5.52-5.54 (1H, m, C<u>H</u>OH), 5.87 (1H, d, *J* = 3.9 Hz, CHO<u>H</u>), 6.45 (1H, dd, *J* = 15.8, 6.0 Hz, 5-CHOHC<u>H</u>CH), 6.65 (1H, d, *J* = 15.8 Hz, 5-CHOHCHC<u>H</u>), 6.81 (1H, dd, *J* = 8.0, 2.6 Hz, H-4''), 7.01-7.04 (2H, m, H-2'' and H-6''), 7.14-7.32 (7H, m, H-3', H-4', H-5', H-5'', H-6' and NH_2), 8.53 (1H, s, H-4), 9.05 (1H, br s, NH). δ_C (100 MHz, $(CD_3)_2SO$) 17.9 (2'-CH_2), 22.6 (6-CH_3), 55.0 (3''-OCH_3), 69.6 (5-CHOH), 96.2 (C-2), 111.5 (C-2''), 113.5 (C-4''), 119.0 (C-6''), 124.6 (C-3a), 125.8 and 125.9 (C-4' and C-5'), 126.9 (C-6'), 128.7 (C-4), 129.4 (5-CHOHCH<u>C</u>H), 129.6 (C-5''), 130.1 (C-3'), 131.8 (5-CHOH<u>C</u>HCH), 133.9 (C-2'), 134.1 (C-5), 136.5 (C-1'), 137.9 (C-1''), 146.7 (C-3), 156.6 (C-7a), 157.1 (C-6), 159.5 (C-3''), 164.0 (2-CONH). ν_{max} (ATR)/cm^{-1} 3447 (N-H amide), 3319 (O-H alcohol, N-H amine), 2919 (C-H aromatic), 2834 (C-H alkane), 1592 (C=O amide), 1510 (C=C aromatic), 1413 (-C-H bending), 1245 (C-N aromatic), 1153 (C-O ether), 1035 (C-N aliphatic). *m/z* (ESI^+): 482 (MNa^+, 100%). HRMS (ESI^+) found (MNa^+): 482.1499 $C_{26}H_{25}N_3NaO_3S$ requires 482.1509.

(*E*)-3-Amino-*N*-(3'-chloro-2'-methylphenyl)-5-(1-hydroxy-3-(3''-methoxyphenyl)allyl)-6-methylthieno[2,3-*b*]pyridine-2-carboxamide 20c

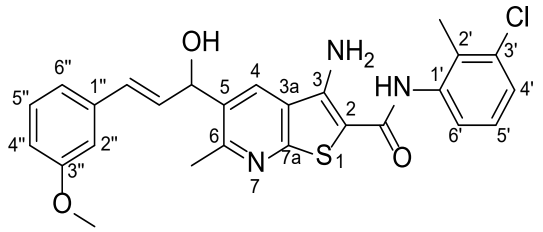

The reaction was carried out following General Procedure **F** using ketone **14c** (20.0 mg, 0.04 mmol), $NaBH_4$ (1.5 mg, 0.04 mmol), and anhydrous $CeCl_3$ (12.3 mg, 0.05 mmol) in dry 2:1 THF:MeOH (5 mL) for 10 min to give the *title compound* **20c** (18.0 mg, 90%) as a yellow solid. m.p. decomp. δ_H (400 MHz, $(CD_3)_2SO$) 2.23 (3H, s, 2'-CH_3), 2.66 (3H, s, 6-CH_3), 3.75 (3H, s, 3''-OCH_3), 5.52-5.54 (1H, m, C<u>H</u>OH), 5.88 (1H, d, *J* = 3.9 Hz, CHO<u>H</u>), 6.44 (1H, dd, *J* = 15.8, 6.0 Hz, 5-CHOHC<u>H</u>CH), 6.65 (1H, d, *J* = 15.8 Hz, 5-CHOHCHC<u>H</u>), 6.81 (1H, dd, *J* = 8.0, 2.6 Hz, H-4''), 7.01-7.04 (2H, m, H-2'' and H-6''), 7.20-7.29 (3H, m, H-5', H-5'' and H-6'), 7.33-7.35 (3H, m, H-4' and NH_2), 8.54 (1H, s, H-4), 9.33 (1H, br s, NH). δ_C (100 MHz, $(CD_3)_2SO$) 15.5 (2'-CH_2), 22.6 (6-CH_3), 55.0 (3''-OCH_3), 69.6 (5-CHOH), 95.6 (C-2), 111.5 (C-2''), 113.5 (C-4''), 119.0 (C-6''), 124.5 (C-3a), 126.2 (C-6'), 126.6 (C-4'), 126.7 (C-5'), 128.8 (C-4), 129.4 (5-CHOHCH<u>C</u>H), 129.6 (C-5''), 131.8 (5-CHOH<u>C</u>HCH), 132.5 (C-2'), 133.6 (C-

3'), 134.2 (C-5), 137.9 (C-1''), 138.2 (C-1'), 147.2 (C-3), 156.7 (C-7a), 157.3 (C-6), 159.5 (C-3''), 164.2 (2-CONH). ν_{max} (ATR)/cm^{-1} 3413 (N-H amide), 3291 (O-H alcohol, N-H amine), 2924 (C-H aromatic), 2861 (C-H alkane), 1577 (C=O amide), 1508 (C=C aromatic), 1431 (-C-H bending), 1256 (C-N aromatic), 1154 (C-O ether), 1038 (C-N aliphatic), 775 (C-Cl). *m/z* (ESI$^+$): 518 (^{37}ClMNa$^+$, 40%), 516 (^{35}ClMNa$^+$, 100%). HRMS (ESI$^+$) found (^{37}ClMNa$^+$): 518.1125 $C_{26}H_{24}{}^{37}ClN_3NaO_3S$ requires 518.1099. Found (^{35}ClMNa$^+$): 516.1118 $C_{26}H_{24}{}^{35}ClN_3NaO_3S$ requires 514.1119.

(*E*)-3-Amino-5-(1-hydroxy-3-(3''-methoxyphenyl)allyl)-6-methyl-*N*-(naphthalen-1'-yl)thieno[2,3-*b*]pyridine-2-carboxamide 20d

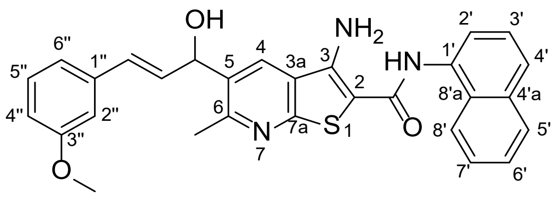

The reaction was carried out following General Procedure **F** using ketone **14d** (50.0 mg, 0.10 mmol), $NaBH_4$ (4.0 mg, 0.10 mmol), and anhydrous $CeCl_3$ (27.0 mg, 0.10 mmol) in dry 2:1 THF:MeOH (5 mL) for 10 min to give the *title compound* **20d** (20.0 mg, 40%) as a yellow solid. m.p. decomp. δ_H (400 MHz, $(CD_3)_2SO$) 2.67 (3H, s, 6-CH_3), 3.75 (3H, s, 3''-OCH_3), 5.51-5.54 (1H, m, C<u>H</u>OH), 5.88 (1H, d, *J* = 3.9 Hz, CHO<u>H</u>), 6.46 (1H, dd, *J* = 15.8, 6.0 Hz, 5-CHOHC<u>H</u>CH), 6.67 (1H, d, *J* = 15.8 Hz, 5-CHOHCHC<u>H</u>), 6.82 (1H, dd, *J* = 8.0, 2.6 Hz, H-4''), 7.02-7.05 (2H, m, H-2'' and H-6''), 7.24 (1H, t, *J* = 7.7 Hz, H-5''), 7.34 (2H, br s, NH_2), 7.52-7.56 (4H, m, 4 × Ar-CH), 7.84-7.86 (1H, m, Ar-CH), 7.91-7.98 (2H, m, 2 × Ar-CH), 8.55 (1H, s, H-4), 9.64 (1H, br s, NH). δ_C (100 MHz, $(CD_3)_2SO$) 22.6 (6-CH_3), 55.0 (3''-OCH_3), 69.6 (5-CHOH), 96.0 (C-2), 111.5 (C-2''), 113.6 (C-4''), 119.0 (C-6''), 123.5 (Ar-CH), 123.9 (C-3a), 124.3 (Ar-CH), 125.5 (Ar-CH), 125.85 (Ar-CH), 125.94 (Ar-CH), 126.2 (Ar-CH), 128.0 (Ar-CH), 128.8 (C-4), 129.4 (5-CHOHCH<u>C</u>H), 129.6 (C-5''), 129.7 (Ar-C), 131.8 (5-CHOH<u>C</u>HCH), 133.7 (C-1'), 134.0 (Ar-C), 134.1 (C-5), 137.9 (C-1''), 147.1 (C-3), 156.7 (C-7a), 157.2 (C-6), 159.5 (C-3''), 164.9 (2-CONH). ν_{max} (ATR)/cm^{-1} 3332 (O-H alcohol, N-H amine, N-H amide), 2964 (C-H aromatic), 2853 (C-H alkane), 1591 (C=O amide), 1524 (C=C aromatic), 1437 (-C-H bending), 1255 (C-N aromatic), 1155 (C-O ether), 1038 (C-N aliphatic). *m/z* (ESI$^+$): 518 (MNa$^+$, 100%). HRMS (ESI$^+$) found (MNa$^+$): 518.1498 $C_{29}H_{25}N_3NaO_3S$ requires 518.1498.

(*E*)-3-Amino-5-(1-hydroxy-3-(3''-methoxyphenyl)allyl)-*N*-(4'-methoxyphenyl)-6-methylthieno[2,3-*b*]pyridine-2-carboxamide 20e

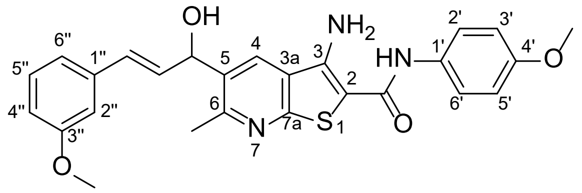

The reaction was carried out following General Procedure **F** using ketone **14e** (20.0 mg, 0.04 mmol), $NaBH_4$ (1.6 mg, 0.04 mmol), and anhydrous $CeCl_3$ (11.0 mg, 0.05 mmol) in dry 2:1 THF:MeOH (2 mL) for 10 min to give the *title compound* **20e** (4.0 mg, 20%) as a yellow solid. m.p. decomp. δ_H (400 MHz, $(CD_3)_2SO$) 2.65 (3H, s, 6-CH_3), 3.74 (3H, s, 4'-OCH_3), 3.75 (3H, s, 3''-OCH_3), 5.51-5.53 (1H, m, C<u>H</u>OH), 5.86 (1H, d, *J* = 3.8 Hz, CHO<u>H</u>), 6.45 (1H, dd, *J* = 15.8, 6.0 Hz, 5-CHOHC<u>H</u>CH), 6.66 (1H, d, *J* = 15.8 Hz, 5-CHOHCHC<u>H</u>), 6.81 (1H, dd, *J* = 8.0, 2.6 Hz, H-4''), 6.89 (2H, d, *J* = 9.0 Hz, H-3' and H-5'), 7.01-7.04 (2H, m, H-2'' and H-6''), 7.23 (1H, t, *J* = 7.6 Hz, H-5''), 7.36 (2H, br s, NH_2), 7.56 (2H, d, *J* = 9.0 Hz, H-2' and H-6'), 8.53 (1H, s, H-4), 9.25 (1H, br s, NH). δ_C (100 MHz, $(CD_3)_2SO$) 22.6 (6-CH_3), 55.0 (3''-OCH_3), 55.1 (4'-OCH_3), 69.6 (5-CHOH), 95.9 (C-2), 111.5 (C-2''), 113.46 and 113.52 (C-4'', C-3' and C-5'), 119.0 (C-6''), 122.9 (C-2' and C-6'), 124.4 (C-3a), 128.6 (C-4), 129.4 (5-CHOHCH<u>C</u>H), 129.6 (C-5''), 131.8 (5-CHOH<u>C</u>HCH), 131.9 (C-1'), 134.1 (C-5), 137.9 (C-1''), 146.9 (C-3), 155.4 (C-4'), 156.5 (C-7a), 157.2 (C-6), 159.5 (C-3''), 163.9 (2-CONH). ν_{max} (ATR)/cm^{-1} 3449 (N-H amide), 3319 (O-H alcohol, N-H amine), 2922 (C-H aromatic), 2851 (C-H alkane), 1592 (C=O amide), 1509 (C=C aromatic), 1413 (-C-H bending), 1245 (C-N aromatic), 1156 (C-O ether), 1035 (C-N aliphatic). *m/z* (ESI^+): 498 (MNa^+, 100%). HRMS (ESI^+) found (MNa^+): 498.1448 $C_{26}H_{25}N_3NaO_4S$ requires 498.1458.

(*E*)-3-Amino-*N*-(3'-chloro-2'-methylphenyl)-5-(1-hydroxy-3-(2''-methoxyphenyl)allyl)-6-methylthieno[2,3-*b*]pyridine-2-carboxamide 21f

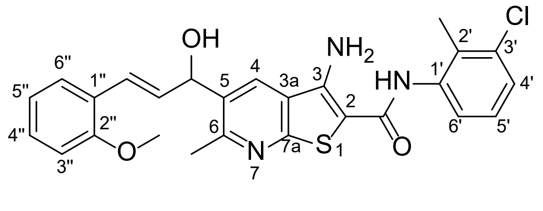

The reaction was carried out following General Procedure **F** using ketone **17f** (30.0 mg, 0.06 mmol), $NaBH_4$ (2.3 mg, 0.06 mmol), and anhydrous $CeCl_3$ (16.5 mg, 0.07 mmol) in dry 2:1

THF:MeOH (2 mL) for 15 min to give the *title compound* **21f** (30.0 mg, quant.) as a yellow solid. m.p. 170-172 °C. δ_H (400 MHz, $(CD_3)_2SO$) 2.23 (3H, s, 2'-CH_3), 2.65 (3H, s, 6-CH_3), 3.80 (3H, s, 2"-OCH_3), 5.52 (1H, t, *J* = 3.8 Hz, 5-CHOH), 5.86 (1H, d, *J* = 3.8 Hz, 5-CHOH), 6.39 (1H, dd, *J* = 16.0, 6.0 Hz, 5-CHOHCHCH), 6.90 (1H, t, *J* = 7.7 Hz, H-5"), 6.95 (1H, d, *J* = 16.0 Hz, 5-CHOHCHCH), 7.00 (1H, d, *J* = 7.7 Hz, H-3"), 7.20-7.29 (3H, m, H-4", H-5' and H-6'), 7.34 (1H, dd, *J* = 7.9, 1.6 Hz, H-4'), 7.35 (2H, br s, NH_2), 7.49 (1H, t, *J* = 1.6 Hz, H-6"), 8.54 (1H, s, H-4), 9.32 (1H, br s, NH). δ_C (100 MHz, $(CD_3)_2SO$) 15.4 (2'-CH_3), 22.6 (6-CH_3), 55.4 (2"-OCH_3), 70.1 (5-CHOH), 95.5 (C-2), 111.3 (C-3"), 120.5 (C-5"), 124.1 (5-CHOHCHCH), 124.5 (C-3a), 125.0 (C-1"), 126.2 (C-6'), 126.6 (C-4' and C-6"), 126.7 (C-5'), 128.7 (C-4"), 128.8 (C-4), 131.7 (5-CHOHCHCH), 132.5 (C-2'), 133.6 (C-3'), 134.3 (C-5), 138.2 (C-1'), 147.2 (C-3), 156.3 (C-2"), 156.7 (C-7a), 157.3 (C-6), 164.2 (2-CONH). ν_{max} (ATR)/cm^{-1} 3425 (N-H amide), 3361 (O-H alcohol, N-H amine), 2923 (C-H aromatic), 2854 (C-H alkane), 1731 (C=O amide), 1574 (C=C aromatic), 1461 (-C-H bending), 1243 (C-N aromatic), 1125 (C-O ether), 1024 (C-N aliphatic), 748 (C-Cl). *m/z* (ESI^+): 518 ($^{37}ClMNa^+$, 40%), 516 ($^{35}ClMNa^+$, 100%). HRMS (ESI^+) found ($^{37}ClMNa^+$): 518.1097 $C_{26}H_{24}{}^{37}ClN_3NaO_3S$ requires 518.1099. Found ($^{35}ClMNa^+$): 516.1125 $C_{25}H_{24}{}^{35}ClN_3NaO_3S$ requires 516.1119.

(*E*)-3-Amino-*N*-(3'-chloro-2'-methylphenyl)-5-(1-hydroxy-3-(3"-(methoxymethoxy)phenyl)allyl)-6-methylthieno[2,3-*b*]pyridine-2-carboxamide 21g

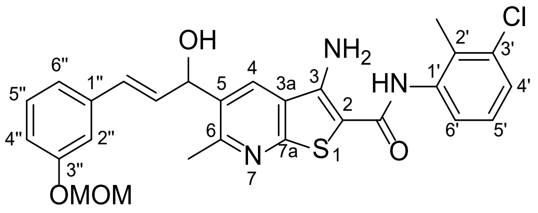

The reaction was carried out following General Procedure **F** using ketone **17g** (50.0 mg, 0.10 mmol), $NaBH_4$ (3.6 mg, 0.10 mmol), and anhydrous $CeCl_3$ (26.0 mg, 0.10 mmol) in dry 2:1 THF:MeOH (4 mL) for 15 min to give the *title compound* **21g** (45.0 mg, 90%) as a yellow solid. m.p. 142-144 °C. δ_H (400 MHz, $(CD_3)_2SO$) 2.23 (3H, s, 2'-CH_3), 2.66 (3H, s, 6-CH_3), 3.36 (3H, s, $MOMCH_3$), 5.19 (2H, s, $MOMCH_2$), 5.53 (1H, t, *J* = 4.4 Hz, 5-CHOH), 5.88 (1H, d, *J* = 4.4 Hz, 5-CHOH), 6.42 (1H, dd, *J* = 16.0, 6.0 Hz, 5-CHOHCHCH), 6.65 (1H, d, *J* = 16.0 Hz, 5-CHOHCHCH), 6.90 (1H, dd, *J* = 8.1, 1.6 Hz, H-4"), 7.08-7.10 (2H, m, H-2" and H-6"), 7.22 (1H, t, *J* = 7.8 Hz, H-5'), 7.25 (1H, t, *J* = 8.1 Hz, H-5"), 7.28 (1H, d, *J* = 7.8 Hz, H-

6'), 7.33-7.35 (3H, m, H-4' and NH_2), 8.52 (1H, s, H-4), 9.33 (1H, br s, NH). δ_C (100 MHz, $(CD_3)_2SO$) 15.4 (2'-CH_3), 22.6 (6-CH_3), 55.5 (MOMCH_3), 69.6 (5-CHOH), 93.8 (MOMCH_2), 95.6 (C-2), 113.8 (C-2''), 115.5 (C-4''), 120.1 (C-6''), 124.5 (C-3a), 126.2 (C-6'), 126.5 (C-4'), 126.7 (C-5'), 128.8 (C-4), 129.2 (5-CHOHCH$\underline{C}$H), 129.6 (C-5''), 131.9 (5-CHOH$\underline{C}$HCH), 132.5 (C-2'), 133.6 (C-3'), 134.1 (C-5), 137.9 (C-1''), 138.2 (C-1'), 147.2 (C-3), 156.7 (C-7a), 157.0 (C-3''), 157.3 (C-6), 164.2 (2-CONH). ν_{max} (ATR)/cm^{-1} 3430 (N-H amide), 3319 (O-H alcohol, N-H amine), 2923 (C-H aromatic), 2853 (C-H alkane), 1737 (C=O amide), 1589 (C=C aromatic), 1506 (-C-H bending), 1253 (C-N aromatic), 1150 (C-O ether), 1079 (C-N aliphatic), 787 (C-Cl). *m/z* (ESI^+): 548 ($^{37}ClMNa^+$, 43%), 546 ($^{35}ClMNa^+$, 100%). HRMS (ESI^+) found ($^{37}ClMNa^+$): 548.1185 $C_{27}H_{26}{}^{37}ClN_3NaO_4S$ requires 548.1205. Found ($^{35}ClMNa^+$): 546.1215 $C_{27}H_{26}{}^{35}ClN_3NaO_4S$ requires 546.1225.

(*E*)-3-Amino-*N*-(3'-chloro-2'-methylphenyl)-5-(1-hydroxy-3-(2''-(trifluoromethyl)phenyl)allyl)-6-methylthieno[2,3-*b*]pyridine-2-carboxamide 21h

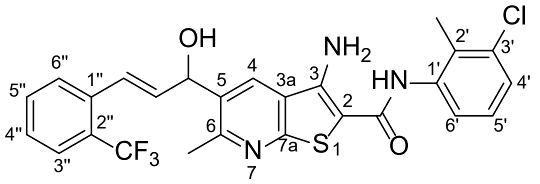

The reaction was carried out following General Procedure **F** using ketone **17h** (31.0 mg, 0.06 mmol), $NaBH_4$ (2.2 mg, 0.06 mmol), and anhydrous $CeCl_3$ (16.0 mg, 0.06 mmol) in dry 2:1 THF:MeOH (3 mL) for 15 min to give the *title compound* **21h** (24.0 mg, 77%) as a yellow solid. m.p. 200-202 °C. δ_H (400 MHz, $(CD_3)_2SO$) 2.23 (3H, s, 2'-CH_3), 2.67 (3H, s, 6-CH_3), 5.60 (1H, t, J = 4.5 Hz, 5-C$\underline{H}$OH), 6.03 (1H, d, J = 4.5 Hz, 5-CHO$\underline{H}$), 6.53 (1H, dd, J = 16.0, 5.9 Hz, 5-CHOHC$\underline{H}$CH), 7.00 (1H, d, J = 16.0 Hz, 5-CHOHCHC$\underline{H}$), 7.22 (1H, t, J = 7.8 Hz, H-5'), 7.28 (1H, dd, J = 7.8, 1.4 Hz, H-6'), 7.33-7.35 (3H, m, H-4' and NH_2), 7.46 (1H, t, J = 7.5 Hz, H-4''), 7.63 (1H, t, J = 7.5 Hz, H-5''), 7.71 (1H, d, J = 7.5 Hz, H-3''), 7.84 (1H, d, J = 7.5 Hz, H-6''), 8.53 (1H, s, H-4), 9.33 (1H, br s, NH). δ_C (100 MHz, $(CD_3)_2SO$) 15.4 (2'-CH_3), 22.4 (6-CH_3), 69.5 (5-CHOH), 95.6 (C-2), 124.3 (C-3a), 124.4 (5-CHOHCH$\underline{C}$H), 125.7 (q, $^3J_{F/C}$ = 5.2 Hz, C-3''), 126.2 (C-6'), 126.3 (q, $^2J_{F/C}$ = 33.1 Hz, C-2''), 126.5 (C-4'), 126.7 (C-5'), 127.7 (C-4''), 127.8 (C-6''), 128.9 (C-4), 132.5 (C-2'), 132.7 (C-5''), 133.6 (C-3'), 133.7 (C-5), 135.2 (q, $^3J_{F/C}$ = 5.2 Hz, C-1''), 136.2 (5-CHOH$\underline{C}$HCH), 138.2 (C-1'), 147.0 (q, $^1J_{F/C}$ = 279.2 Hz, CF_3), 147.2 (C-3), 156.8 (C-7a), 157.4 (C-6), 164.2 (2-CONH). ν_{max} (ATR)/cm^{-1} 3445 (N-H amide), 3343 (O-H alcohol, N-H amine), 2925 (C-H aromatic), 2856 (C-H alkane), 1714

(C=O amide), 1605 (C=C aromatic), 1462 (-C-H bending), 1312 (C-F), 1260 (C-N aromatic), 1060 (C-N aliphatic), 763 (C-Cl). *m/z* (ESI$^+$): 556 (^{37}ClMNa$^+$, 40%), 554 (^{35}ClMNa$^+$, 100%). HRMS (ESI$^+$) found (^{37}ClMNa$^+$): 556.0872 $C_{26}H_{21}{}^{37}ClF_3N_3NaO_2S$ requires 556.0867. Found (^{35}ClMNa$^+$): 554.0880 $C_{26}H_{21}{}^{35}ClF_3N_3NaO_2S$ requires 554.0887.

(*E*)-3-Amino-*N*-(3'-chloro-2'-methylphenyl)-5-(3-(2''-fluorophenyl)-1-hydroxyallyl)-6-methylthieno[2,3-*b*]pyridine-2-carboxamide 21i

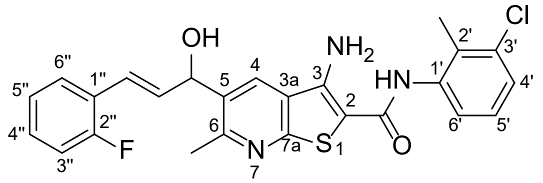

The reaction was carried out following General Procedure **F** using ketone **17i** (35.0 mg, 0.07 mmol), $NaBH_4$ (2.8 mg, 0.07 mmol), and anhydrous $CeCl_3$ (20.0 mg, 0.08 mmol) in dry 2:1 THF:MeOH (4 mL) for 15 min to give the *title compound* **21i** (34.0 mg, 97%) as a yellow solid. m.p. 207-209 °C. δ_H (400 MHz, $(CD_3)_2SO$) 2.23 (3H, s, 2'-CH_3), 2.67 (3H, s, 6-CH_3), 5.58 (1H, t, *J* = 4.5 Hz, 5-C<u>H</u>OH), 5.95 (1H, d, *J* = 4.5 Hz, 5-CHO<u>H</u>), 6.55 (1H, dd, *J* = 16.0, 5.9 Hz, 5-CHOHC<u>H</u>CH), 6.85 (1H, d, *J* = 16.0 Hz, 5-CHOHCHC<u>H</u>), 7.17 (1H, t, *J* = 7.3 Hz, H-5''), 7.19-7.21 (1H, m, H-3''), 7.22 (1H, t, *J* = 7.8 Hz, H-5'), 7.27-7.33 (3H, m, H-4', H-4'', H-6'), 7.35 (2H, br s, NH_2), 7.65 (1H, td, *J* = 7.7, 1.6 Hz, H-6''), 8.53 (1H, s, H-4), 9.33 (1H, br s, NH). δ_C (100 MHz, $(CD_3)_2SO$) 15.5 (2'-CH_3), 22.6 (6-CH_3), 69.6 (5-CHOH), 95.6 (C-2), 115.7 (d, $^2J_{F/C}$ = 21.9 Hz, C-3''), 121.1 (d, $^3J_{F/C}$ = 3.7 Hz, 5-CHOHCH<u>C</u>H), 124.0 (d, $^2J_{F/C}$ = 12.1 Hz, C-1''), 124.5 (C-3a), 124.6 (d, $^4J_{F/C}$ = 3.1 Hz, C-5''), 126.2 (C-6'), 126.5 (C-4'), 126.7 (C-5'), 127.7 (d, $^3J_{F/C}$ = 3.7 Hz, C-6''), 128.9 (C-4), 129.3 (d, $^3J_{F/C}$ = 8.7 Hz, C-4''), 132.5 (C-2'), 133.6 (C-3'), 133.9 (C-5), 134.3 (5-CHOH<u>C</u>HCH), 138.2 (C-1'), 147.2 (C-3), 156.7 (C-7a), 157.3 (C-6), 159.6 (d, $^1J_{F/C}$ = 247.1 Hz, C-2''), 164.2 (2-CONH). ν_{max} (ATR)/cm^{-1} 3421 (N-H amide), 3314 (O-H alcohol, N-H amine), 2924 (C-H aromatic), 2855 (C-H alkane), 1737 (C=O amide), 1573 (C=C aromatic), 1486 (-C-H bending), 1253 (C-N aromatic), 1230 (C-F), 1030 (C-N aliphatic), 754 (C-Cl). *m/z* (ESI$^+$): 506 (^{37}ClMNa$^+$, 40%), 504 (^{35}ClMNa$^+$, 100%). HRMS (ESI$^+$) found (^{37}ClMNa$^+$): 506.0881 $C_{25}H_{21}{}^{37}ClFN_3NaO_2S$ requires 506.0898. Found (^{35}ClMNa$^+$): 504.0910 $C_{25}H_{21}{}^{35}ClFN_3NaO_2S$ requires 504.0919.

(*E*)-3-Amino-*N*-(3'-chloro-2'-methylphenyl)-5-(3-(3''-fluorophenyl)-1-hydroxyallyl)-6-methylthieno[2,3-*b*]pyridine-2-carboxamide 21j

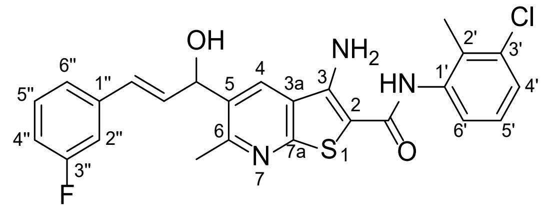

The reaction was carried out following General Procedure **F** using ketone **17j** (30.0 mg, 0.06 mmol), $NaBH_4$ (2.4 mg, 0.06 mmol), and anhydrous $CeCl_3$ (17.0 mg, 0.07 mmol) in dry 2:1 THF:MeOH (2 mL) for 15 min to give the *title compound* **21j** (25.0 mg, quant.) as a yellow solid. m.p. 96-98 °C. δ_H (400 MHz, $(CD_3)_2SO$) 2.23 (3H, s, 2'-CH_3), 2.67 (3H, s, 6-CH_3), 5.54 (1H, t, J = 4.0 Hz, 5-C<u>H</u>OH), 5.92 (1H, d, J = 4.0 Hz, 5-CHO<u>H</u>), 6.54 (1H, dd, J = 16.0, 5.7 Hz, 5-CHOHC<u>H</u>CH), 6.71 (1H, d, J = 16.0 Hz, 5-CHOHCHC<u>H</u>), 7.06 (1H, td, J = 8.3, 2.2 Hz, H-4''), 7.22 (1H, t, J = 7.8 Hz, H-5'), 7.28 (1H, d, J = 7.8 Hz, H-6'), 7.31-7.37 (6H, m, H-2'', H-4', H-5'', H-6'' and NH_2), 8.52 (1H, s, H-4), 9.33 (1H, br s, NH). δ_C (100 MHz, $(CD_3)_2SO$) 15.5 (2'-CH_3), 22.6 (6-CH_3), 69.5 (5-CHOH), 95.6 (C-2), 112.7 (d, $^2J_{F/C}$ = 20.2 Hz, C-2''), 114.3 (d, $^2J_{F/C}$ = 20.2 Hz, C-4''), 122.8 (d, $^4J_{F/C}$ = 3.0 Hz, C-6''), 124.5 (C-3a), 126.2 (C-6'), 126.5 (C-4'), 126.7 (C-5'), 128.2 (5-CHOHCH<u>C</u>H), 128.9 (C-4), 130.5 (d, $^3J_{F/C}$ = 8.3 Hz, C-5''), 132.5 (C-2'), 133.2 (5-CHOH<u>C</u>HCH), 133.6 (C-3'), 133.9 (C-5), 138.2 (C-1'), 139.3 (C-1''), 147.2 (C-3), 156.7 (C-7a), 157.3 (C-6), 162.5 (d, $^1J_{F/C}$ = 245.0 Hz, C-3''), 164.2 (2-CONH). ν_{max} (ATR)/cm^{-1} 3426 (N-H amide), 3318 (O-H alcohol, N-H amine), 2923 (C-H aromatic), 2853 (C-H alkane), 1731 (C=O amide), 1583 (C=C aromatic), 1461 (-C-H bending), 1252 (C-N aromatic), 1230 (C-F), 1143 (C-O ether), 1071 (C-N aliphatic), 778 (C-Cl). *m/z* (ESI$^+$): 506 (^{37}ClMNa$^+$, 40%), 504 (^{35}ClMNa$^+$, 100%). HRMS (ESI$^+$) found (^{37}ClMNa$^+$): 506.0893 $C_{25}H_{21}{}^{37}ClFN_3NaO_2S$ requires 506.0898. Found (^{35}ClMNa$^+$): 504.0911 $C_{25}H_{21}{}^{35}ClFN_3NaO_2S$ requires 504.0919.

(*E*)-3-Amino-*N*-(3'-chloro-2'-methylphenyl)-5-(3-(4''-fluorophenyl)-1-hydroxyallyl)-6-methylthieno[2,3-*b*]pyridine-2-carboxamide 21k

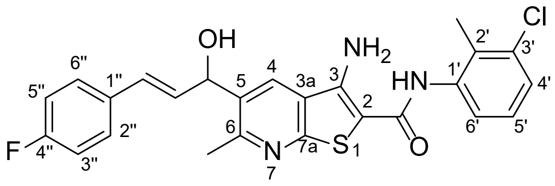

The reaction was carried out following General Procedure **F** using ketone **17k** (31.0 mg, 0.06 mmol), $NaBH_4$ (2.4 mg, 0.06 mmol), and anhydrous $CeCl_3$ (17.5 mg, 0.07 mmol) in dry 2:1 THF:MeOH (3 mL) for 15 min to give the *title compound* **21k** (31.0 mg, quant.) as a yellow solid. m.p. 190-192 °C. δ_H (400 MHz, $(CD_3)_2SO$) 2.23 (3H, s, 2'-CH_3), 2.66 (3H, s, 6-CH_3), 5.53 (1H, t, *J* = 4.8 Hz, 5-C<u>H</u>OH), 5.88 (1H, d, *J* = 4.8 Hz, 5-CHO<u>H</u>), 6.40 (1H, dd, *J* = 16.0, 6.0 Hz, 5-CHOHC<u>H</u>CH), 6.69 (1H, d, *J* = 16.0 Hz, 5-CHOHCHC<u>H</u>), 7.15 (2H, t, *J* = 8.8 Hz, H-3'' and H-5''), 7.22 (1H, t, *J* = 7.8 Hz, H-5'), 7.28 (1H, dd, *J* = 7.8, 1.4 Hz, H-6'), 7.33-7.35 (3H, m, H-4' and NH_2), 7.51 (2H, dd, *J* = 8.8, 5.8 Hz, H-2'' and H-6''), 8.54 (1H, s, H-4), 9.33 (1H, br s, NH). δ_C (100 MHz, $(CD_3)_2SO$) 15.4 (2'-CH_3), 22.6 (6-CH_3), 69.6 (5-CHOH), 95.5 (C-2), 115.4 (d, $^2J_{F/C}$ = 21.5 Hz, C-3'' and C-5''), 124.5 (C-3a), 126.2 (C-6'), 126.5 (C-4'), 126.7 (C-5'), 128.3 (5-CHOHCH<u>C</u>H), 128.4 (d, $^3J_{F/C}$ = 8.2 Hz, C-2'' and C-6''), 128.8 (C-4), 131.4 (5-CHOH<u>C</u>HCH), 132.5 (C-2'), 133.1 (d, $^4J_{F/C}$ = 2.9 Hz, C-1''), 133.6 (C-3'), 134.1 (C-5), 138.2 (C-1'), 147.2 (C-3), 156.7 (C-7a), 157.3 (C-6), 161.6 (d, $^1J_{F/C}$ = 244.8 Hz, C-4''), 164.2 (2-CONH). ν_{max} (ATR)/cm^{-1} 3444 (N-H amide), 3332 (O-H alcohol, N-H amine), 2925 (C-H aromatic), 2856 (C-H alkane), 1737 (C=O amide), 1592 (C=C aromatic), 1460 (-C-H bending), 1261 (C-N aromatic), 1228 (C-F), 1059 (C-N aliphatic), 760 (C-Cl). *m/z* (ESI^+): 506 ($^{37}ClMNa^+$, 40%), 504 ($^{35}ClMNa^+$, 100%). HRMS (ESI^+) found ($^{37}ClMNa^+$): 506.0881 $C_{25}H_{21}{}^{37}ClFN_3NaO_2S$ requires 506.0898. Found ($^{35}ClMNa^+$): 504.0908 $C_{25}H_{21}{}^{35}ClFN_3NaO_2S$ requires 504.0919.

(*E*)-3-Amino-*N*-(3'-chloro-2'-methylphenyl)-5-(3-(2''-chlorophenyl)-1-hydroxyallyl)-6-methylthieno[2,3-*b*]pyridine-2-carboxamide 21l

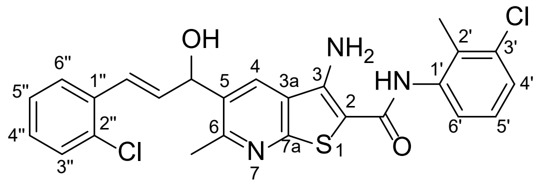

The reaction was carried out following General Procedure **F** using ketone **17l** (35.0 mg, 0.07 mmol), $NaBH_4$ (2.7 mg, 0.07 mmol), and anhydrous $CeCl_3$ (19.0 mg, 0.08 mmol) in dry 2:1 THF:MeOH (3 mL) for 15 min to give the *title compound* **21l** (30.0 mg, 85%) as a yellow solid. m.p. 159-161 °C. δ_H (400 MHz, $(CD_3)_2SO$) 2.23 (3H, s, 2'-CH_3), 2.69 (3H, s, 6-CH_3), 5.62 (1H, t, *J* = 4.3 Hz, 5-C<u>H</u>OH), 6.01 (1H, d, *J* = 4.3 Hz, 5-CHO<u>H</u>), 6.53 (1H, dd, *J* = 16.0, 5.8 Hz, 5-CHOHC<u>H</u>CH), 7.07 (1H, d, *J* = 16.0 Hz, 5-CHOHCHC<u>H</u>), 7.24 (1H, t, *J* = 7.7 Hz, H-5'), 7.28-7.35 (4H, m, H-4', H-4'', H-5'' and H-6'), 7.37 (2H, br s, NH_2), 7.47 (1H, dd, *J* = 7.4, 1.8 Hz, H-3''), 7.74 (1H, d, *J* = 7.4, 1.8 Hz, H-6''), 8.54 (1H, s, H-4), 9.33 (1H, br s, NH). δ_C (100 MHz, $(CD_3)_2SO$) 15.5 (2'-CH_3), 22.6 (6-CH_3), 69.5 (5-CHOH), 95.6 (C-2), 124.5 (C-3a), 124.8 (5-CHOHCH<u>C</u>H), 126.2 (C-6'), 126.5 (C-4'), 126.7 (C-5'), 127.2 (C-6''), 127.4 (C-5''), 128.9 (C-4), 129.1 (C-4''), 129.6 (C-3''), 132.0 (C-2''), 132.5 (C-2'), 133.6 (C-1''), 133.8 (C-3'), 134.2 (C-5), 134.8 (5-CHOH<u>C</u>HCH), 138.2 (C-1'), 147.2 (C-3), 156.8 (C-7a), 157.4 (C-6), 164.2 (2-CONH). ν_{max} (ATR)/cm^{-1} 3419 (N-H amide), 3319 (O-H alcohol, N-H amine), 2924 (C-H aromatic), 2853 (C-H alkane), 1730 (C=O amide), 1592 (C=C aromatic), 1462 (-C-H bending), 1279 (C-N aromatic), 1039 (C-N aliphatic), 754 (C-Cl), 698 (C-Cl). *m/z* (ESI$^+$): 524 ($^{37}Cl_2MNa^+$, 13%), 522 ($^{37}Cl^{35}ClMNa^+$, 67%), 520 ($^{35}Cl_2MNa^+$, 100%). HRMS (ESI$^+$) found ($^{37}Cl_2MNa^+$): 524.0564 $C_{25}H_{21}{}^{37}Cl_2N_3NaO_2S$ requires 524.0579. Found ($^{35}Cl^{37}ClMNa^+$): 522.0589 $C_{25}H_{21}{}^{35}Cl^{37}ClN_3NaO_2S$ requires 522.0599. Found ($^{35}Cl_2MNa^+$): 520.0610 $C_{25}H_{21}{}^{35}Cl_2N_3NaO_2S$ requires 520.0624.

(*E*)-3-Amino-*N*-(3'-chloro-2'-methylphenyl)-5-(3-(3''-chlorophenyl)-1-hydroxyallyl)-6-methylthieno[2,3-*b*]pyridine-2-carboxamide 21m

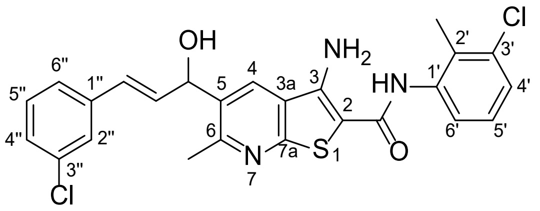

The reaction was carried out following General Procedure **F** using ketone **17m** (30.0 mg, 0.06 mmol), $NaBH_4$ (2.3 mg, 0.06 mmol), and anhydrous $CeCl_3$ (16.0 mg, 0.07 mmol) in dry 2:1 THF:MeOH (2 mL) for 15 min to give the *title compound* **21m** (30.0 mg, quant.) as a yellow semi-solid. m.p. decomp. δ_H (400 MHz, $(CD_3)_2SO$) 2.23 (3H, s, 2'-CH_3), 2.67 (3H, s, 6-CH_3), 5.53 (1H, t, *J* = 3.8 Hz, 5-C$\underline{H}$OH), 5.92 (1H, d, *J* = 3.8 Hz, 5-CHO$\underline{H}$), 6.55 (1H, dd, *J* = 16.0, 5.9 Hz, 5-CHOHC$\underline{H}$CH), 6.70 (1H, d, *J* = 16.0 Hz, 5-CHOHCHC$\underline{H}$), 7.22 (1H, t, *J* = 7.9 Hz, H-5'), 7.27-7.30 (2H, m, H-4' and H-6'), 7.33-7.37 (4H, m, H-4'', H-5'' and NH_2), 7.44 (1H, d, *J* = 7.9 Hz, H-6''), 7.57 (1H, t, *J* = 1.9 Hz, H-2''), 8.52 (1H, s, H-4), 9.33 (1H, br s, NH). δ_C (100 MHz, $(CD_3)_2SO$) 15.5 (2'-CH_3), 22.6 (6-CH_3), 69.4 (5-CHOH), 95.6 (C-2), 124.5 (C-3a), 125.2 (C-6''), 126.0 (C-2''), 126.2 (C-6'), 126.5 (C-4'), 126.7 (C-5'), 127.3 (C-4''), 127.9 (5-CHOHCH$\underline{C}$H), 128.9 (C-4), 130.4 (C-5''), 132.5 (C-2'), 133.3 (5-CHOH$\underline{C}$HCH), 133.5 (C-3''), 133.6 (C-3'), 133.9 (C-5), 138.2 (C-1'), 138.9 (C-1''), 147.1 (C-3), 156.7 (C-7a), 157.4 (C-6), 164.2 (2-CONH). ν_{max} (ATR)/cm^{-1} 3440 (N-H amide), 3210 (O-H alcohol, N-H amine), 2924 (C-H aromatic), 2854 (C-H alkane), 1735 (C=O amide), 1509 (C=C aromatic), 1431 (-C-H bending), 1258 (C-N aromatic), 1199 (C-O ether), 1076 (C-N aliphatic), 776 (C-Cl), 760 (C-Cl). *m/z* (ESI^+): 524 ($^{37}Cl_2MNa^+$, 12%), 522 ($^{37}Cl^{35}ClMNa^+$, 55%), 520 ($^{35}Cl_2MNa^+$, 100%). HRMS (ESI^+) found ($^{37}Cl_2MNa^+$): 524.0560 $C_{25}H_{21}{}^{37}Cl_2N_3NaO_2S$ requires 524.0579. Found ($^{35}Cl^{37}ClMNa^+$): 522.0579 $C_{25}H_{21}{}^{35}Cl^{37}ClN_3NaO_2S$ requires 522.0599. Found ($^{35}Cl_2MNa^+$): 520.0608 $C_{25}H_{21}{}^{35}Cl_2N_3NaO_2S$ requires 520.0624.

(*E*)-3-Amino-*N*-(3'-chloro-2'-methylphenyl)-5-(3-(4''-chlorophenyl)-1-hydroxyallyl)-6-methylthieno[2,3-*b*]pyridine-2-carboxamide 21n

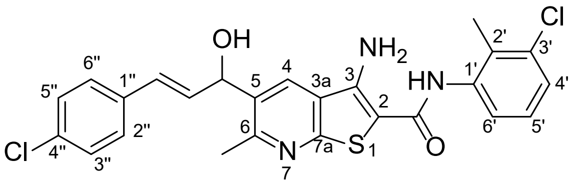

The reaction was carried out following General Procedure **F** using ketone **17n** (40.0 mg, 0.08 mmol), $NaBH_4$ (3.0 mg, 0.08 mmol), and anhydrous $CeCl_3$ (22.0 mg, 0.09 mmol) in dry 2:1 THF:MeOH (4 mL) for 15 min to give the *title compound* **21n** (40.0 mg, quant.) as a yellow solid. m.p. 202-204 °C. δ_H (400 MHz, $(CD_3)_2SO$) 2.23 (3H, s, 2'-CH_3), 2.66 (3H, s, 6-CH_3), 5.53 (1H, t, *J* = 4.5 Hz, 5-C<u>H</u>OH), 5.91 (1H, d, *J* = 4.5 Hz, 5-CHO<u>H</u>), 6.47 (1H, dd, *J* = 16.0, 6.0 Hz, 5-CHOHC<u>H</u>CH), 6.70 (1H, d, *J* = 16.0 Hz, 5-CHOHCHC<u>H</u>), 7.22 (1H, t, *J* = 7.8 Hz, H-5'), 7.28 (1H, d, *J* = 7.8 Hz, H-6'), 7.33-7.35 (3H, m, H-4' and NH_2), 7.37 (2H, d, *J* = 8.2 Hz, H-3'' and H-5''), 7.50 (2H, d, *J* = 8.2 Hz, H-2'' and H-6''), 8.53 (1H, s, H-4), 9.33 (1H, br s, NH). δ_C (100 MHz, $(CD_3)_2SO$) 15.4 (2'-CH_3), 22.6 (6-CH_3), 69.5 (5-CHOH), 95.6 (C-2), 124.5 (C-3a), 126.2 (C-6'), 126.5 (C-4'), 126.7 (C-5'), 128.1 (5-CHOHCH<u>C</u>H), 128.2 (C-2'' and C-6''), 128.6 (C-3'' and C-5''), 128.8 (C-4), 131.9 (C-4''), 132.47 (5-CHOH<u>C</u>HCH), 132.51 (C-2'), 133.6 (C-3'), 134.0 (C-5), 135.5 (C-1''), 138.2 (C-1'), 147.2 (C-3), 156.7 (C-7a), 157.3 (C-6), 164.2 (2-CONH). ν_{max} (ATR)/cm^{-1} 3430 (N-H amide), 3343 (O-H alcohol, N-H amine), 2924 (C-H aromatic), 2856 (C-H alkane), 1736 (C=O amide), 1591 (C=C aromatic), 1491 (-C-H bending), 1260 (C-N aromatic), 1037 (C-N aliphatic), 777 (C-Cl), 698 (C-Cl). *m/z* (ESI^+): 524 ($^{37}Cl_2MNa^+$, 13%), 522 ($^{37}Cl^{35}ClMNa^+$, 80%), 520 ($^{35}Cl_2MNa^+$, 100%). HRMS (ESI^+) found ($^{37}Cl_2MNa^+$): 524.0617 $C_{25}H_{21}{}^{37}Cl_2N_3NaO_2S$ requires 524.0579. Found ($^{35}Cl^{37}ClMNa^+$): 522.0598 $C_{25}H_{21}{}^{35}Cl^{37}ClN_3NaO_2S$ requires 522.0599. Found ($^{35}Cl_2MNa^+$): 520.0613 $C_{25}H_{21}{}^{35}Cl_2N_3NaO_2S$ requires 520.0624.

(*E*)-3-Amino-5-(3-(2''-bromophenyl)-1-hydroxyallyl)-*N*-(3'-chloro-2'-methylphenyl)-6-methylthieno[2,3-*b*]pyridine-2-carboxamide 21o

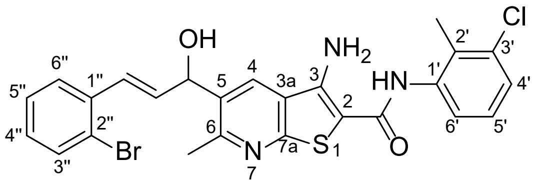

The reaction was carried out following General Procedure **F** using ketone **17o** (30.0 mg, 0.06 mmol), $NaBH_4$ (2.0 mg, 0.06 mmol), and anhydrous $CeCl_3$ (15.0 mg, 0.06 mmol) in dry 2:1 THF:MeOH (2 mL) for 15 min to give the *title compound* **21o** (24.0 mg, 80%) as a yellow solid. m.p. 173-175 °C. δ_H (400 MHz, $(CD_3)_2SO$) 2.23 (3H, s, 2'-CH_3), 2.69 (3H, s, 6-CH_3), 5.59 (1H, t, *J* = 4.5 Hz, 5-C<u>H</u>OH), 6.00 (1H, d, *J* = 4.5 Hz, 5-CHO<u>H</u>), 6.47 (1H, dd, *J* = 15.9, 5.9 Hz, 5-CHOHC<u>H</u>CH), 6.99 (1H, d, *J* = 15.9 Hz, 5-CHOHCHC<u>H</u>), 7.20 (1H, td, *J* = 7.9, 1.5 Hz, H-4''), 7.22 (1H, t, *J* = 7.8 Hz, H-5'), 7.28 (1H, dd, *J* = 7.8, 1.4 Hz, H-6'), 7.33-7.37 (4H, m, H-4', H-5'' and NH_2), 7.62 (1H, dd, *J* = 7.9, 1.5 Hz, H-3''), 7.70 (1H, d, *J* = 7.9, 1.5 Hz, H-6''), 8.54 (1H, s, H-4), 9.33 (1H, br s, NH). δ_C (100 MHz, $(CD_3)_2SO$) 15.5 (2'-CH_3), 22.6 (6-CH_3), 69.5 (5-CHOH), 95.6 (C-2), 122.9 (C-2''), 124.5 (C-3a), 126.2 (C-6'), 126.5 (C-4'), 126.7 (C-5'), 127.4 (C-5'' and 5-CHOHCH<u>C</u>H), 128.0 (C-6''), 128.9 (C-4), 129.4 (C-4''), 132.5 (C-2'), 132.8 (C-3''), 133.6 (C-3'), 133.8 (C-5), 134.8 (5-CHOH<u>C</u>HCH), 135.9 (C-1''), 138.2 (C-1'), 147.2 (C-3), 156.8 (C-7a), 157.4 (C-6), 164.2 (2-CONH). ν_{max} (ATR)/cm^{-1} 3437 (N-H amide), 3333 (O-H alcohol, N-H amine), 2923 (C-H aromatic), 2853 (C-H alkane), 1730 (C=O amide), 1592 (C=C aromatic), 1462 (-C-H bending), 1259 (C-N aromatic), 1023 (C-N aliphatic), 753 (C-Cl), 668 (C-Br). *m/z* (ESI^+): 568 ($^{81}Br^{37}ClMNa^+$, 28%), 566 ($^{79}Br^{37}ClMNa^+$ and $^{81}Br^{35}ClMNa^+$, 100%), 564 ($^{79}Br^{35}ClMNa^+$, 75%). HRMS (ESI^+) found ($^{81}Br^{37}ClMNa^+$): 568.0083 $C_{25}H_{21}{}^{81}Br^{37}ClN_3NaO_2S$ requires 568.0078. Found ($^{79}Br^{37}ClMNa^+$ and $^{81}Br^{35}ClMNa^+$): 566.0098 $C_{25}H_{21}{}^{79}Br^{37}ClN_3NaO_2S$ and $C_{25}H_{21}{}^{81}Br^{35}ClN_3NaO_2S$ requires 566.0098. Found ($^{79}Br\,^{35}ClMNa^+$): 564.0111 $C_{25}H_{21}{}^{79}Br^{35}ClN_3NaO_2S$ requires 564.0119.

(*E*)-3-Amino-5-(3-(3''-bromophenyl)-1-hydroxyallyl)-*N*-(3'-chloro-2'-methylphenyl)-6-methylthieno[2,3-*b*]pyridine-2-carboxamide 21p

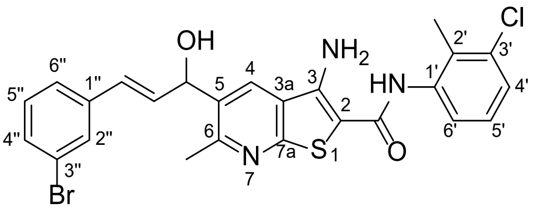

The reaction was carried out following General Procedure **F** using ketone **17p** (35.0 mg, 0.06 mmol), $NaBH_4$ (2.4 mg, 0.06 mmol), and anhydrous $CeCl_3$ (17.5 mg, 0.07 mmol) in dry 2:1 THF:MeOH (3 mL) for 15 min to give the *title compound* **21p** (35.0 mg, quant.) as a yellow solid. m.p. 179-181 °C. δ_H (400 MHz, $(CD_3)_2SO$) 2.23 (3H, s, 2'-CH_3), 2.66 (3H, s, 6-CH_3), 5.54 (1H, t, *J* = 4.3 Hz, 5-C<u>H</u>OH), 5.92 (1H, d, *J* = 4.3 Hz, 5-CHO<u>H</u>), 6.54 (1H, dd, *J* = 16.0, 6.0 Hz, 5-CHOHC<u>H</u>CH), 6.69 (1H, d, *J* = 16.0 Hz, 5-CHOHCHC<u>H</u>), 7.22 (1H, t, *J* = 7.7 Hz, H-5'), 7.28 (1H, d, *J* = 7.7 Hz, H-6'), 7.28 (1H, t, *J* = 7.9 Hz, H-5''), 7.33-7.35 (3H, m, H-4' and NH_2), 7.43 (1H, d, *J* = 7.9 Hz, H-4''), 7.48 (1H, d, *J* = 7.9 Hz, H-6''), 7.70 (1H, t, *J* = 1.7 Hz, H-2''), 8.52 (1H, s, H-4), 9.33 (1H, br s, NH). δ_C (100 MHz, $(CD_3)_2SO$) 15.5 (2'-CH_3), 22.6 (6-CH_3), 69.4 (5-CHOH), 95.5 (C-2), 122.1 (C-3''), 124.5 (C-3a), 125.5 (C-6''), 126.2 (C-6'), 126.5 (C-4'), 126.7 (C-5'), 127.8 (5-CHOHCH<u>C</u>H), 128.9 (C-2'' and C-4), 130.2 (C-4''), 130.7 (C-5''), 132.5 (C-2'), 133.3 (5-CHOH<u>C</u>HCH), 133.6 (C-3'), 133.9 (C-5), 138.2 (C-1'), 139.1 (C-1''), 147.2 (C-3), 156.7 (C-7a), 157.4 (C-6), 164.2 (2-CONH). ν_{max} (ATR)/cm^{-1} 3421 (N-H amide), 3312 (O-H alcohol, N-H amine), 2924 (C-H aromatic), 2853 (C-H alkane), 1729 (C=O amide), 1578 (C=C aromatic), 1461 (-C-H bending), 1257 (C-N aromatic), 1070 (C-N aliphatic), 776 (C-Cl), 669 (C-Br). *m/z* (ESI$^+$): 568 ($^{81}Br^{37}ClMNa^+$, 30%), 566 ($^{79}Br^{37}ClMNa^+$ and $^{81}Br^{35}ClMNa^+$, 100%), 564 ($^{79}Br^{35}ClMNa^+$, 75%). HRMS (ESI$^+$) found ($^{81}Br^{37}ClMNa^+$): 568.0065 $C_{25}H_{21}{}^{81}Br^{37}ClN_3NaO_2S$ requires 568.0078. Found ($^{79}Br^{37}ClMNa^+$ and $^{81}Br^{35}ClMNa^+$): 566.0089 $C_{25}H_{21}{}^{79}Br^{37}ClN_3NaO_2S$ and $C_{25}H_{21}{}^{81}Br^{35}ClN_3NaO_2S$ requires 566.0098. Found ($^{79}Br\,^{35}ClMNa^+$): 564.0103 $C_{25}H_{21}{}^{79}Br^{35}ClN_3NaO_2S$ requires 564.0119.

(*E*)-3-Amino-5-(3-(4''-bromophenyl)-1-hydroxyallyl)-*N*-(3'-chloro-2'-methylphenyl)-6-methylthieno[2,3-*b*]pyridine-2-carboxamide 21q

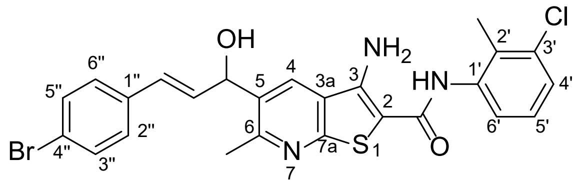

The reaction was carried out following General Procedure **F** using ketone **17q** (35.0 mg, 0.06 mmol), $NaBH_4$ (2.4 mg, 0.06 mmol), and anhydrous $CeCl_3$ (17.5 mg, 0.07 mmol) in dry 2:1 THF:MeOH (4 mL) for 15 min to give the *title compound* **21q** (34.0 mg, 97%) as a yellow solid. m.p. 215-217 °C. δ_H (400 MHz, $(CD_3)_2SO$) 2.23 (3H, s, 2'-CH_3), 2.66 (3H, s, 6-CH_3), 5.53 (1H, t, *J* = 4.5 Hz, 5-C<u>H</u>OH), 5.91 (1H, d, *J* = 4.5 Hz, 5-CHO<u>H</u>), 6.48 (1H, dd, *J* = 16.0, 6.0 Hz, 5-CHOHC<u>H</u>CH), 6.68 (1H, d, *J* = 16.0 Hz, 5-CHOHCHC<u>H</u>), 7.22 (1H, t, *J* = 7.8 Hz, H-5'), 7.28 (1H, dd, *J* = 7.8, 1.4 Hz, H-6'), 7.33-7.35 (3H, m, H-4' and NH_2), 7.44 (2H, d, *J* = 8.5 Hz, H-2'' and H-6''), 7.51 (2H, d, *J* = 8.5 Hz, H-3'' and H-5''), 8.52 (1H, s, H-4), 9.33 (1H, br s, NH). δ_C (100 MHz, $(CD_3)_2SO$) 15.4 (2'-CH_3), 22.6 (6-CH_3), 69.5 (5-CHOH), 95.6 (C-2), 120.5 (C-4''), 124.5 (C-3a), 126.2 (C-6'), 126.5 (C-4'), 126.7 (C-5'), 128.2 (5-CHOHCH<u>C</u>H), 128.5 (C-2'' and C-6''), 128.9 (C-4), 131.5 (C-3'' and C-5''), 132.5 (C-2'), 132.6 (5-CHOH<u>C</u>HCH), 133.6 (C-3'), 134.0 (C-5), 135.8 (C-1''), 138.2 (C-1'), 147.2 (C-3), 156.7 (C-7a), 157.3 (C-6), 164.2 (2-CONH). ν_{max} (ATR)/cm^{-1} 3447 (N-H amide), 3340 (O-H alcohol, N-H amine), 2924 (C-H aromatic), 2855 (C-H alkane), 1731 (C=O amide), 1591 (C=C aromatic), 1487 (-C-H bending), 1260 (C-N aromatic), 1072 (C-N aliphatic), 777 (C-Cl), 655 (C-Br). *m/z* (ESI^+): 568 ($^{81}Br^{37}ClMNa^+$, 28%), 566 ($^{79}Br^{37}ClMNa^+$ and $^{81}Br^{35}ClMNa^+$, 100%), 564 ($^{79}Br^{35}ClMNa^+$, 68%). HRMS (ESI^+) found ($^{81}Br^{37}ClMNa^+$): 568.0065 $C_{25}H_{21}{}^{81}Br^{37}ClN_3NaO_2S$ requires 568.0078. Found ($^{79}Br^{37}ClMNa^+$ and $^{81}Br^{35}ClMNa^+$): 566.0080 $C_{25}H_{21}{}^{79}Br^{37}ClN_3NaO_2S$ and $C_{25}H_{21}{}^{81}Br^{35}ClN_3NaO_2S$ requires 566.0098. Found ($^{79}Br^{35}ClMNa^+$): 564.0105 $C_{25}H_{21}{}^{79}Br^{35}ClN_3NaO_2S$ requires 564.0119.

(*E*)-3-Amino-*N*-(3'-chloro-2'-methylphenyl)-5-(1-hydroxy-3-(3''-hydroxyphenyl)allyl)-6-methylthieno[2,3-*b*]pyridine-2-carboxamide 21r

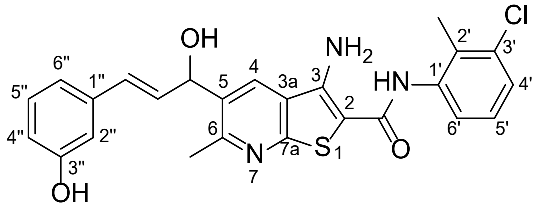

The reaction was carried out following General Procedure **F** using ketone **17r** (18.0 mg, 0.04 mmol), $NaBH_4$ (1.4 mg, 0.04 mmol), and anhydrous $CeCl_3$ (10.0 mg, 0.04 mmol) in dry 2:1 THF:MeOH (2 mL) for 15 min to give the *title compound* **21r** (18.0 mg, quant.) as a yellow solid. m.p. 135-137 °C. δ_H (400 MHz, $(CD_3)_2SO$) 2.23 (3H, s, 2'-CH_3), 2.65 (3H, s, 6-CH_3), 5.51 (1H, t, *J* = 4.3 Hz, 5-C<u>H</u>OH), 5.85 (1H, d, *J* = 4.3 Hz, 5-CHO<u>H</u>), 6.32 (1H, dd, *J* = 16.0, 6.0 Hz, 5-CHOHC<u>H</u>CH), 6.59 (1H, d, *J* = 16.0 Hz, 5-CHOHCHC<u>H</u>), 6.65 (1H, dd, *J* = 8.0, 2.1 Hz, H-4''), 6.82 (1H, d, *J* = 2.1 Hz, H-2''), 6.87 (1H, d, *J* = 8.0 Hz, H-6''), 7.11 (1H, t, *J* = 8.0 Hz, H-5''), 7.22 (1H, t, *J* = 7.7 Hz, H-5'), 7.28 (1H, d, *J* = 7.7 Hz, H-6'), 7.33-7.35 (3H, m, H-4' and NH_2), 8.53 (1H, s, H-4), 9.32 (1H, br s, NH), 9.35 (1H, br s, OH). δ_C (100 MHz, $(CD_3)_2SO$) 15.4 (2'-CH_3), 22.6 (6-CH_3), 69.6 (5-CHOH), 95.6 (C-2), 113.0 (C-2''), 114.7 (C-4''), 117.4 (C-6''), 124.5 (C-3a), 126.2 (C-6'), 126.5 (C-4'), 126.7 (C-5'), 128.8 (C-4), 129.5 (5-CHOHCH<u>C</u>H), 129.6 (C-5''), 131.2 (5-CHOH<u>C</u>HCH), 132.5 (C-2'), 133.6 (C-3'), 134.2 (C-5), 137.8 (C-1''), 138.2 (C-1'), 147.2 (C-3), 156.6 (C-7a), 157.3 (C-6), 157.6 (C-3''), 164.2 (2-CONH). ν_{max} (ATR)/cm^{-1} 3318 (very broad N-H amide, O-H alcohol, and N-H amine), 2922 (C-H aromatic), 2853 (C-H alkane), 1735 (C=O amide), 1591 (C=C aromatic), 1461 (-C-H bending), 1262 (C-N aromatic), 1072 (C-N aliphatic), 778 (C-Cl). *m/z* (ESI^+): 504 ($^{37}ClMNa^+$, 40%), 502 ($^{35}ClMNa^+$, 100%). HRMS (ESI^+) found ($^{37}ClMNa^+$): 504.1005 $C_{25}H_{22}{}^{37}ClN_3NaO_3S$ requires 504.0942. Found ($^{35}ClMNa^+$): 502.0944 $C_{25}H_{22}{}^{35}ClN_3NaO_3S$ requires 502.0963.

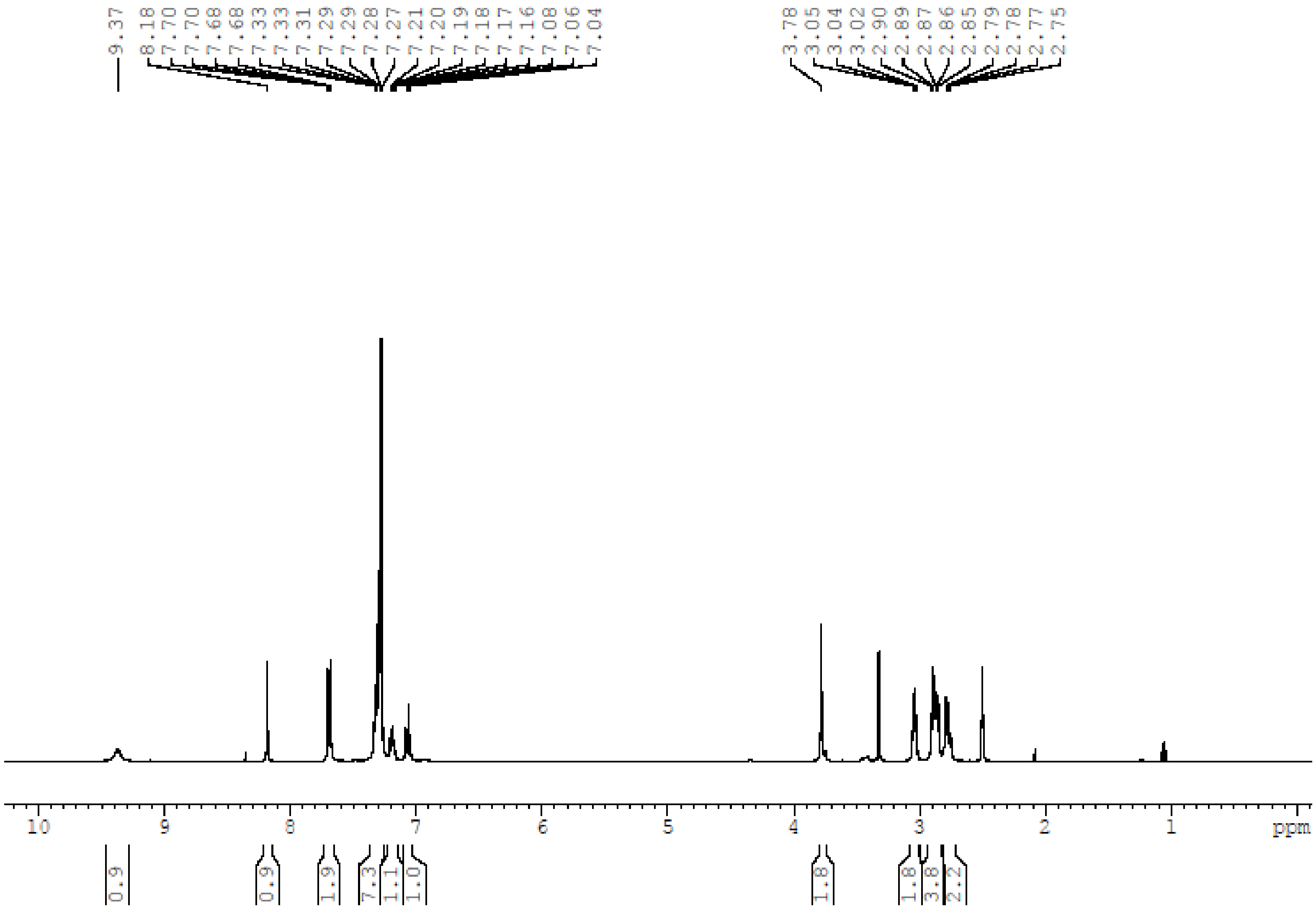

Figure S1: ^{1}H NMR spectrum of **5a** (400 MHz; DMSO-d_6).

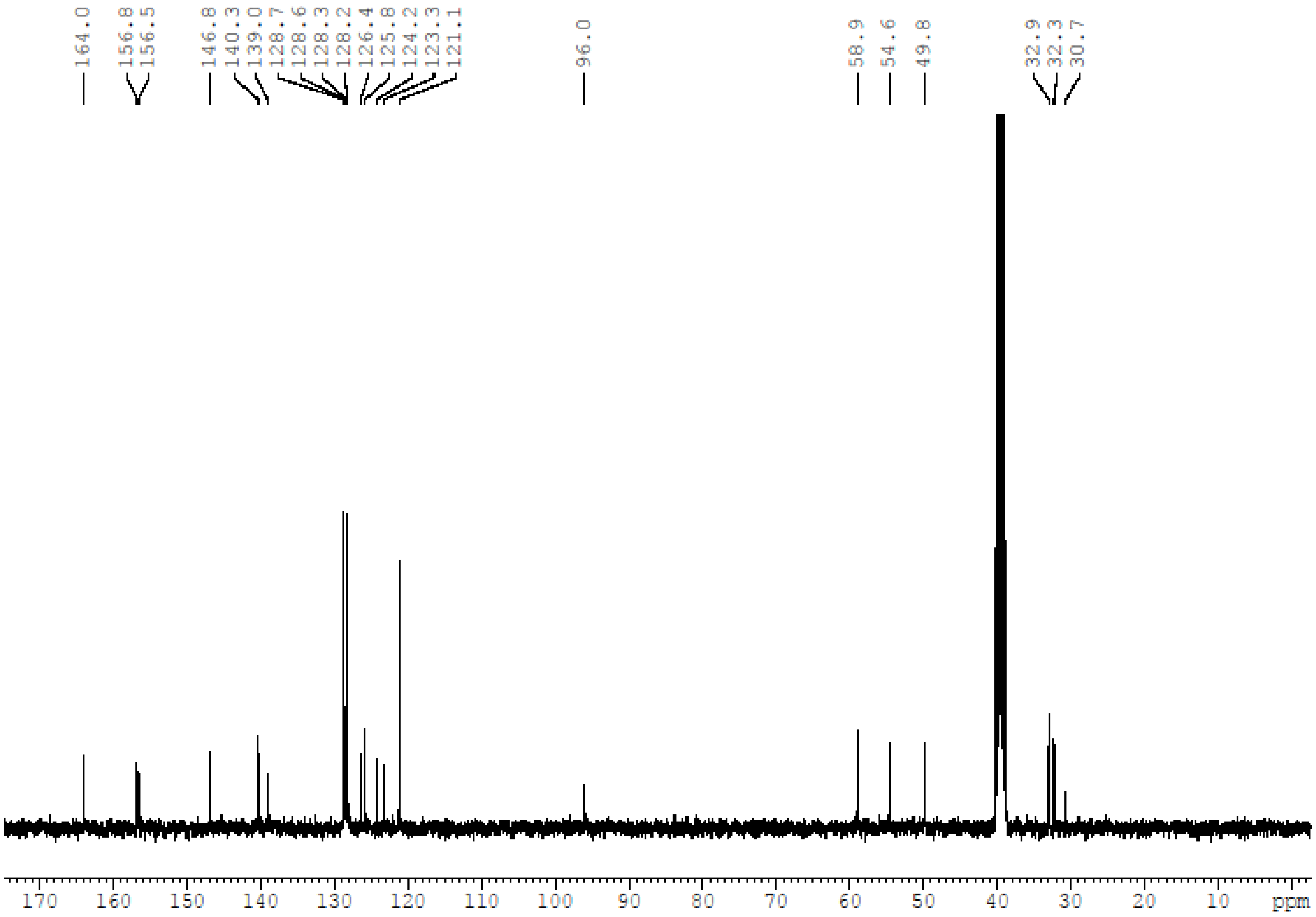

Figure S2: ^{13}C NMR spectrum of **5a** (100 MHz; DMSO-d_6).

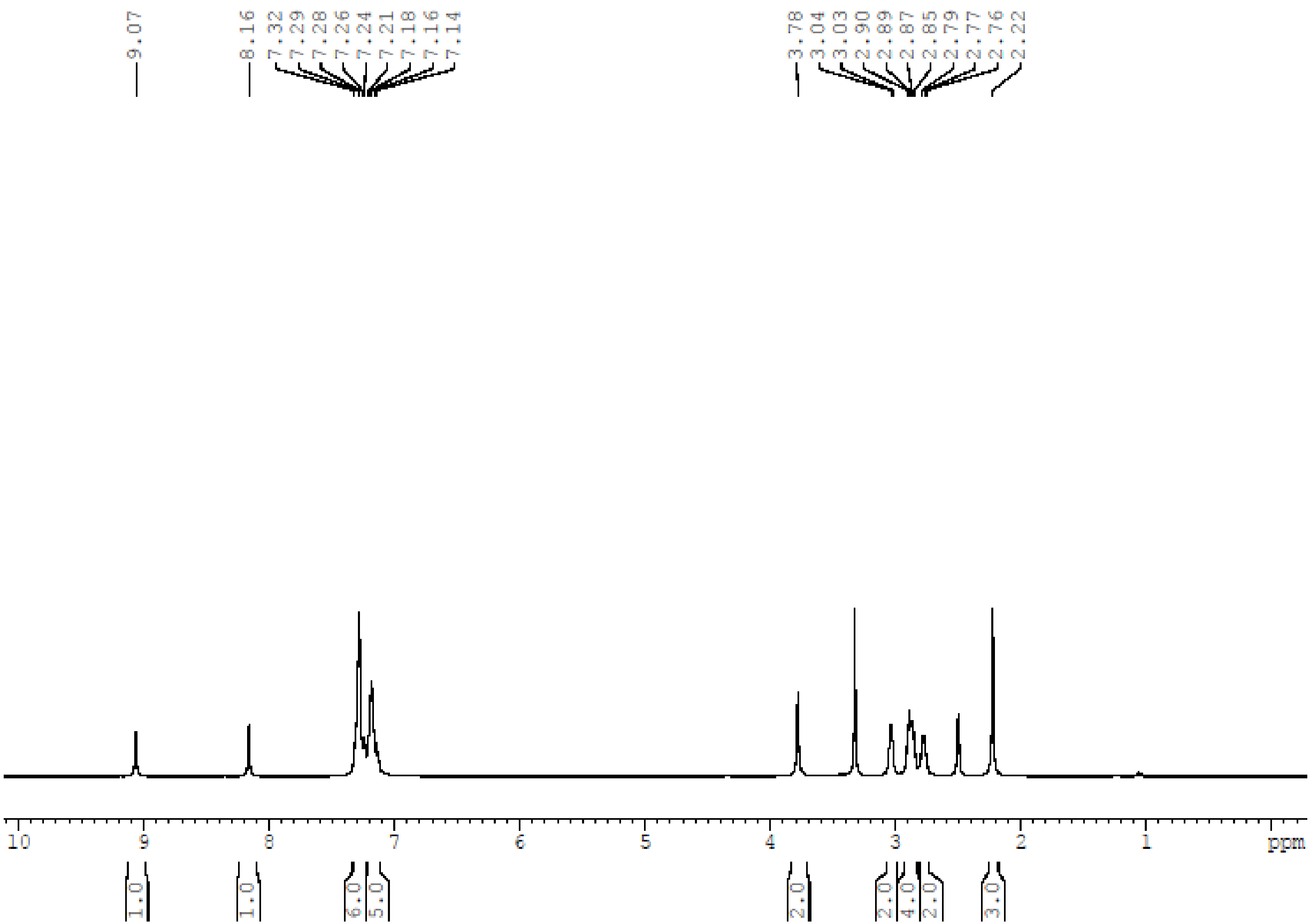

Figure S3: ^{1}H NMR spectrum of **5b** (400 MHz; DMSO-d_6).

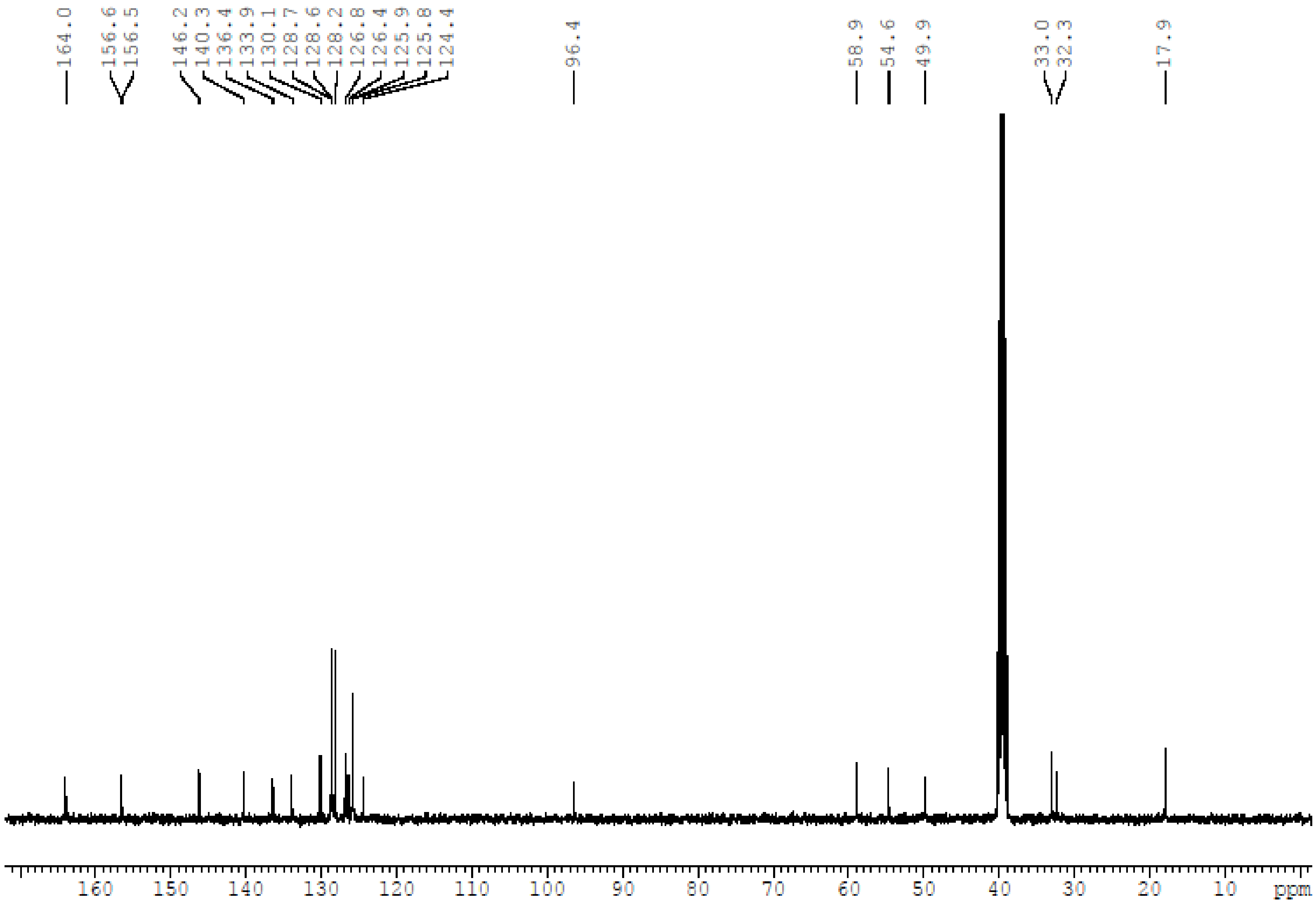

Figure S4: ^{13}C NMR spectrum of **5b** (100 MHz; DMSO-d_6).

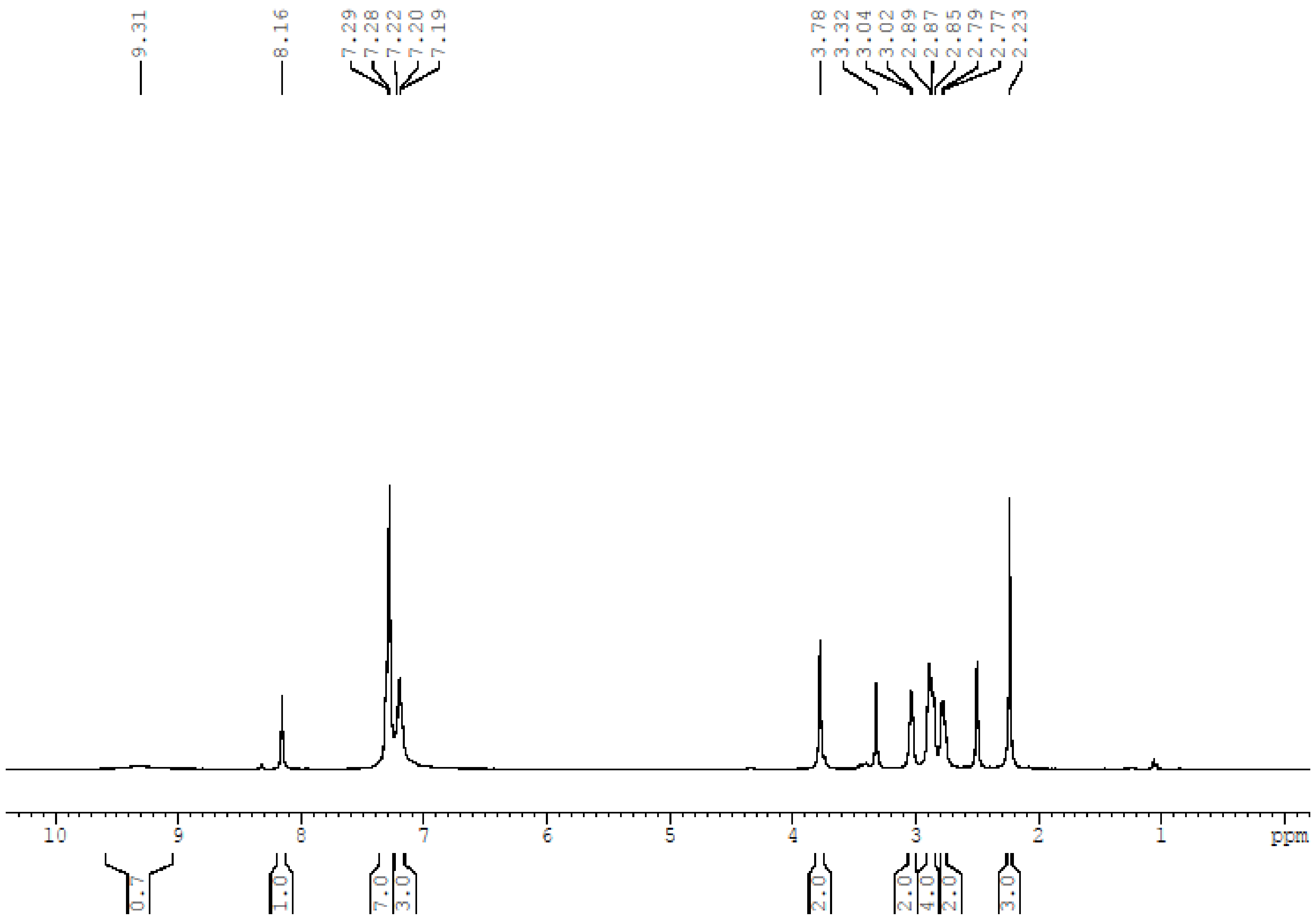

Figure S5: ^{1}H NMR spectrum of **5c** (400 MHz; DMSO-d_6).

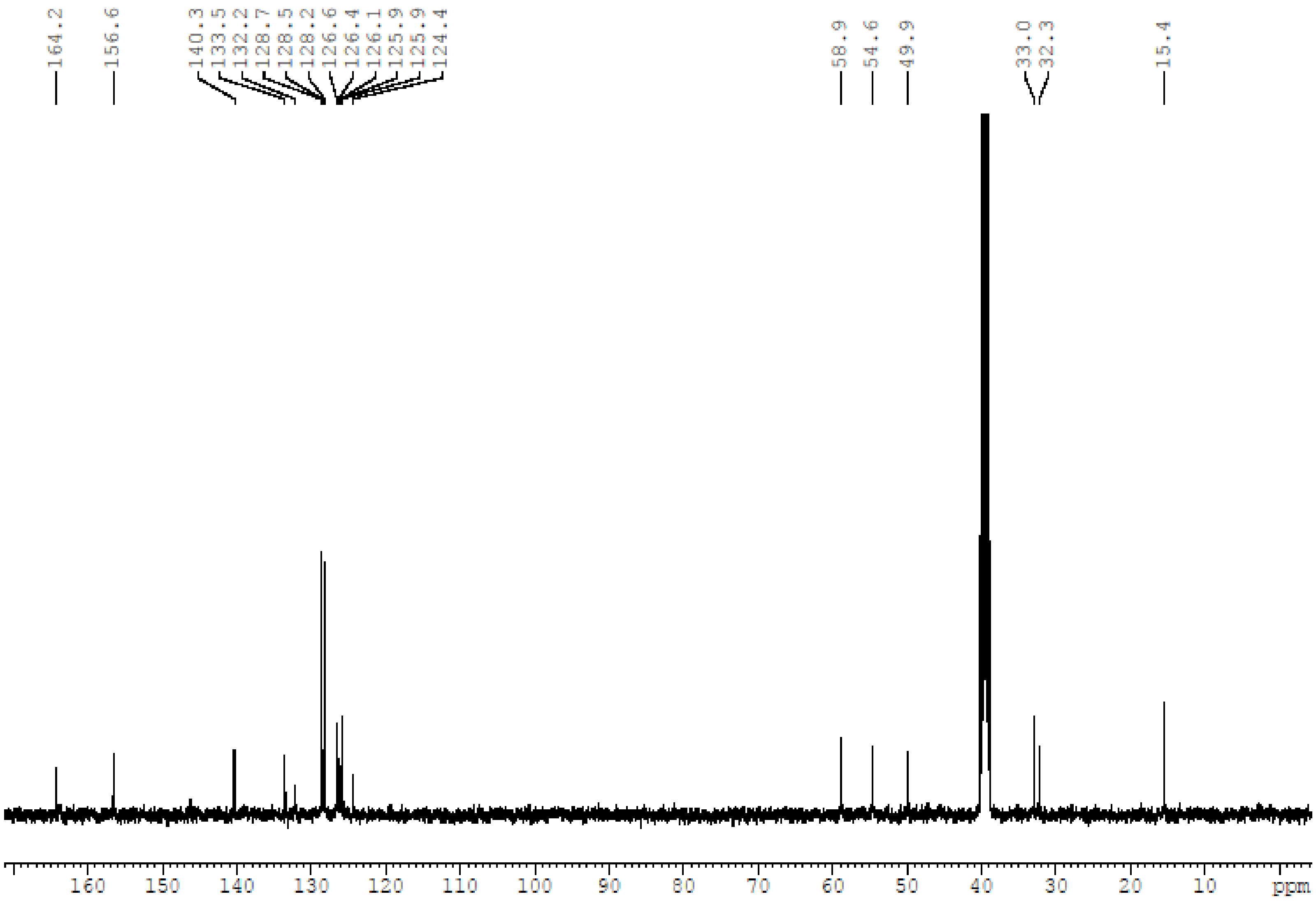

Figure S6: ^{13}C NMR spectrum of **5c** (100 MHz; DMSO-d_6).

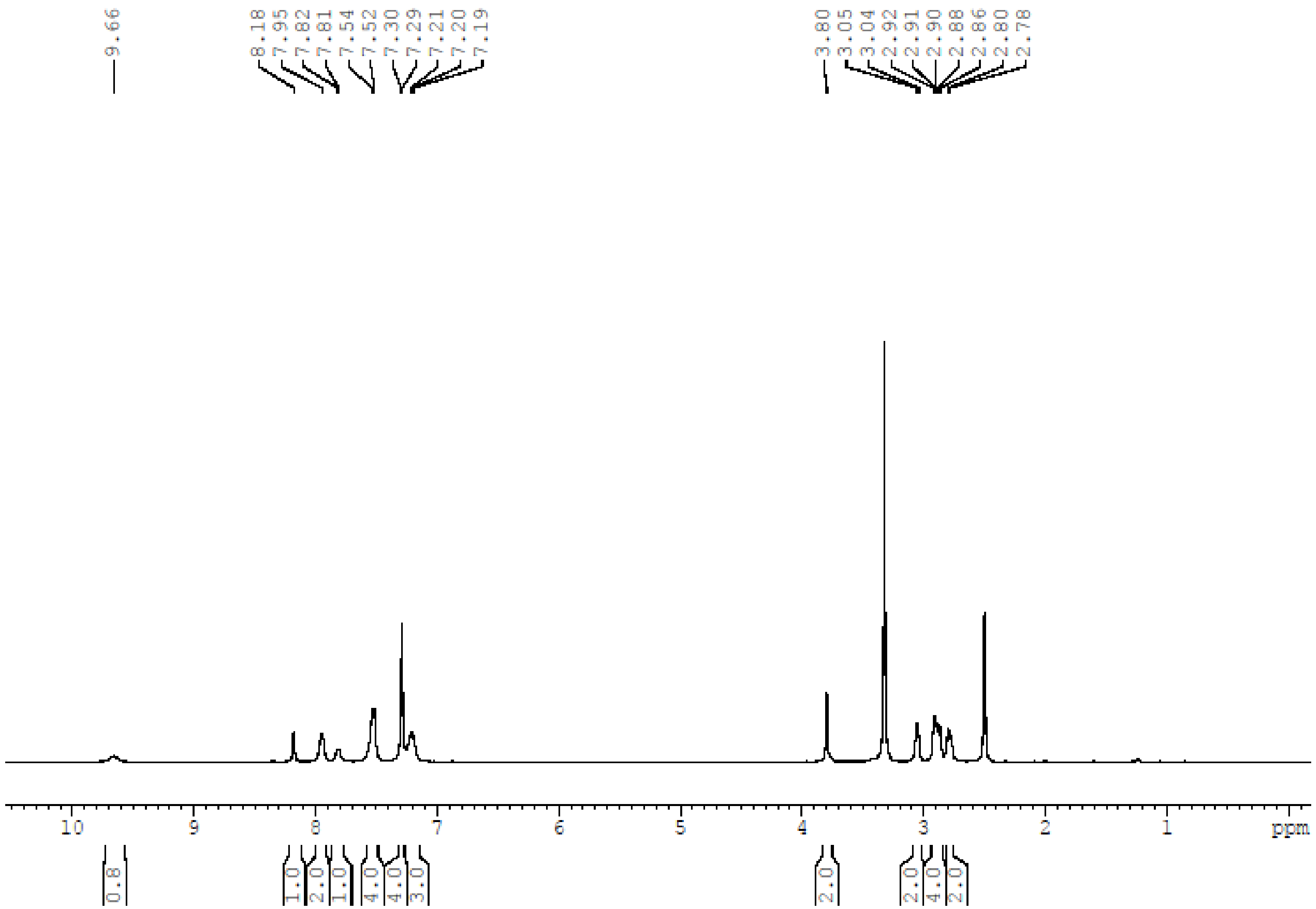

Figure S7: ^{1}H NMR spectrum of **5d** (400 MHz; DMSO-d_6).

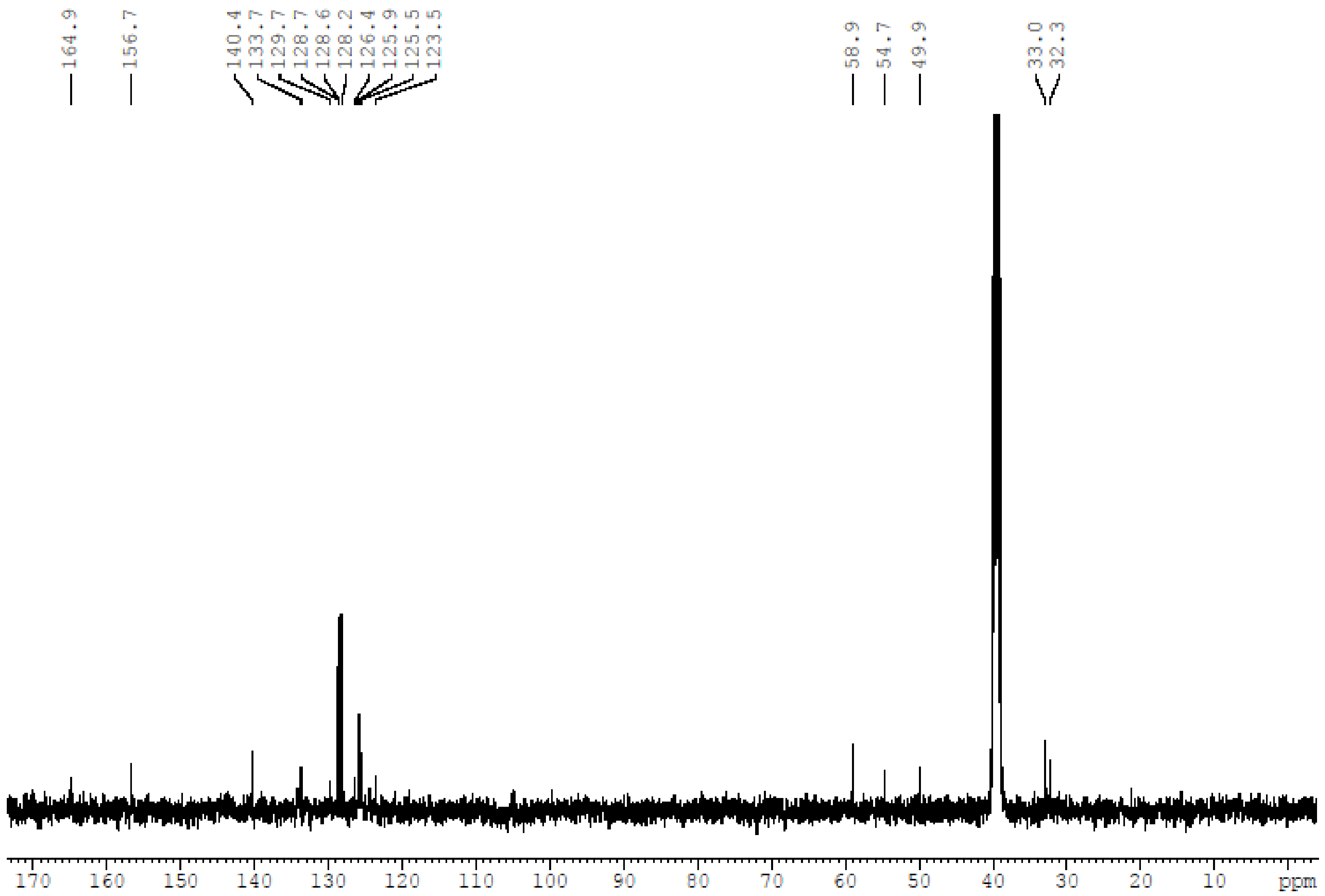

Figure S8: ^{13}C NMR spectrum of **5d** (100 MHz; DMSO-d_6).

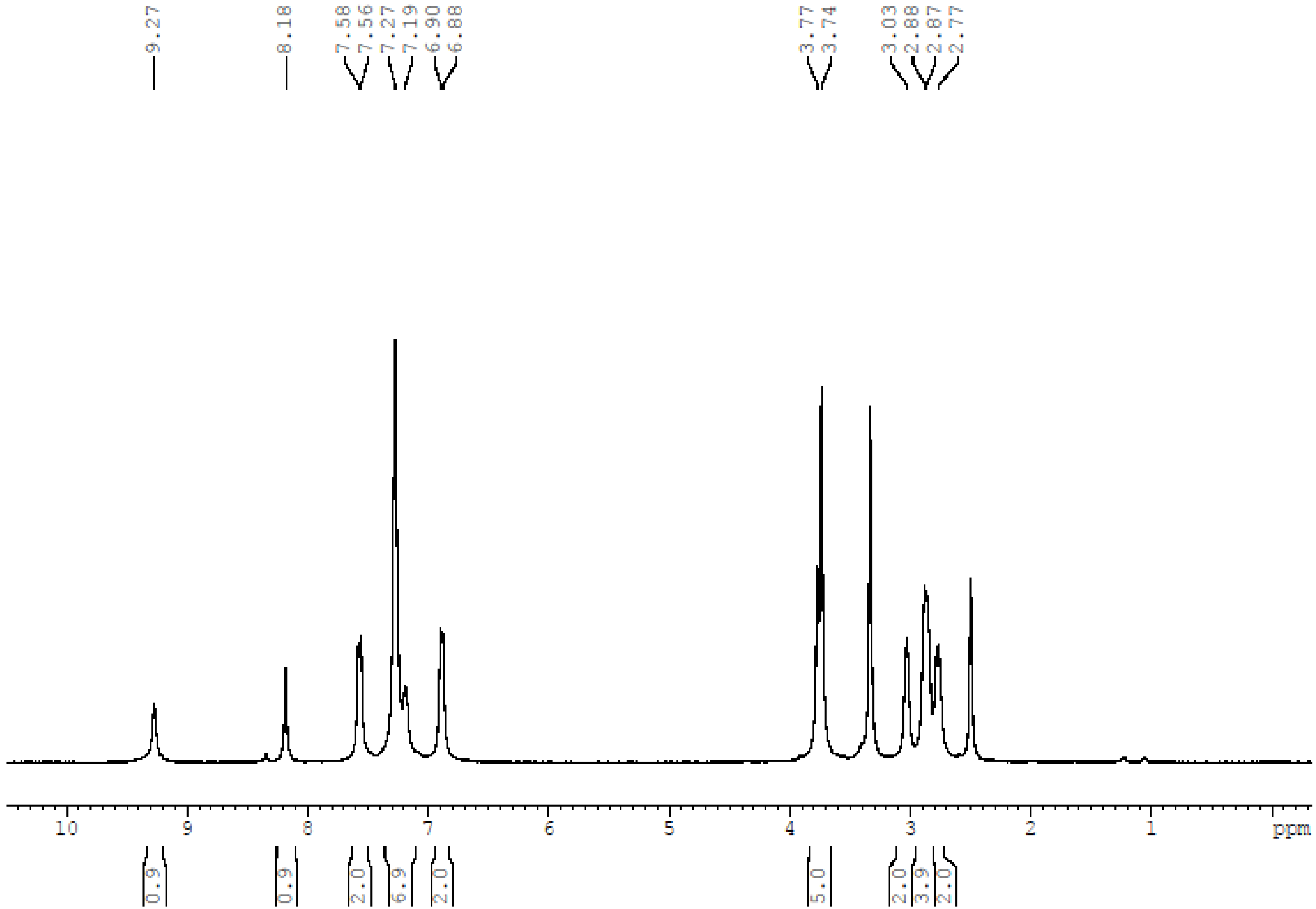

Figure S9: ^{1}H NMR spectrum of **5e** (400 MHz; DMSO-d_6).

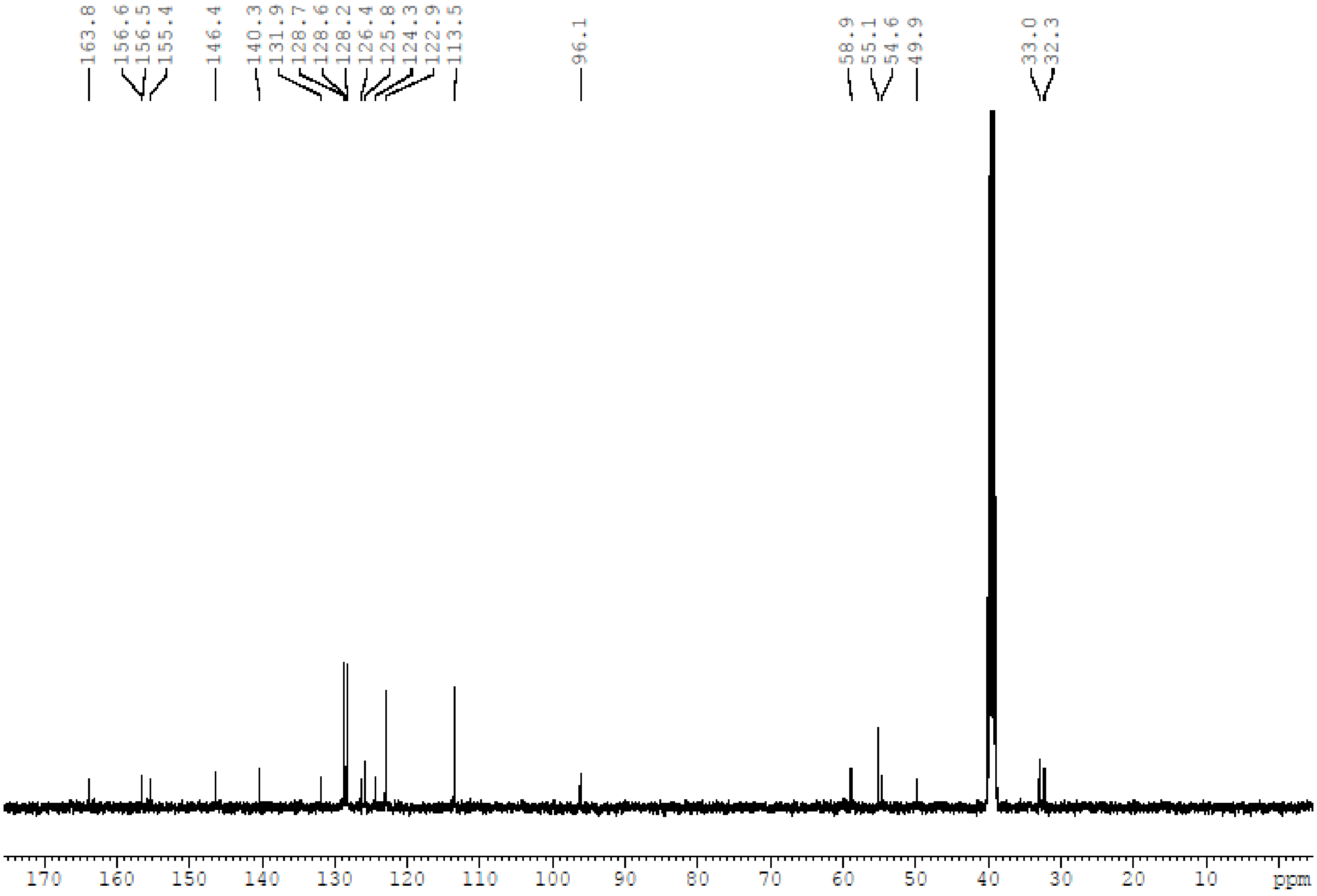

Figure S10: ^{13}C NMR spectrum of **5e** (100 MHz; DMSO-d_6).

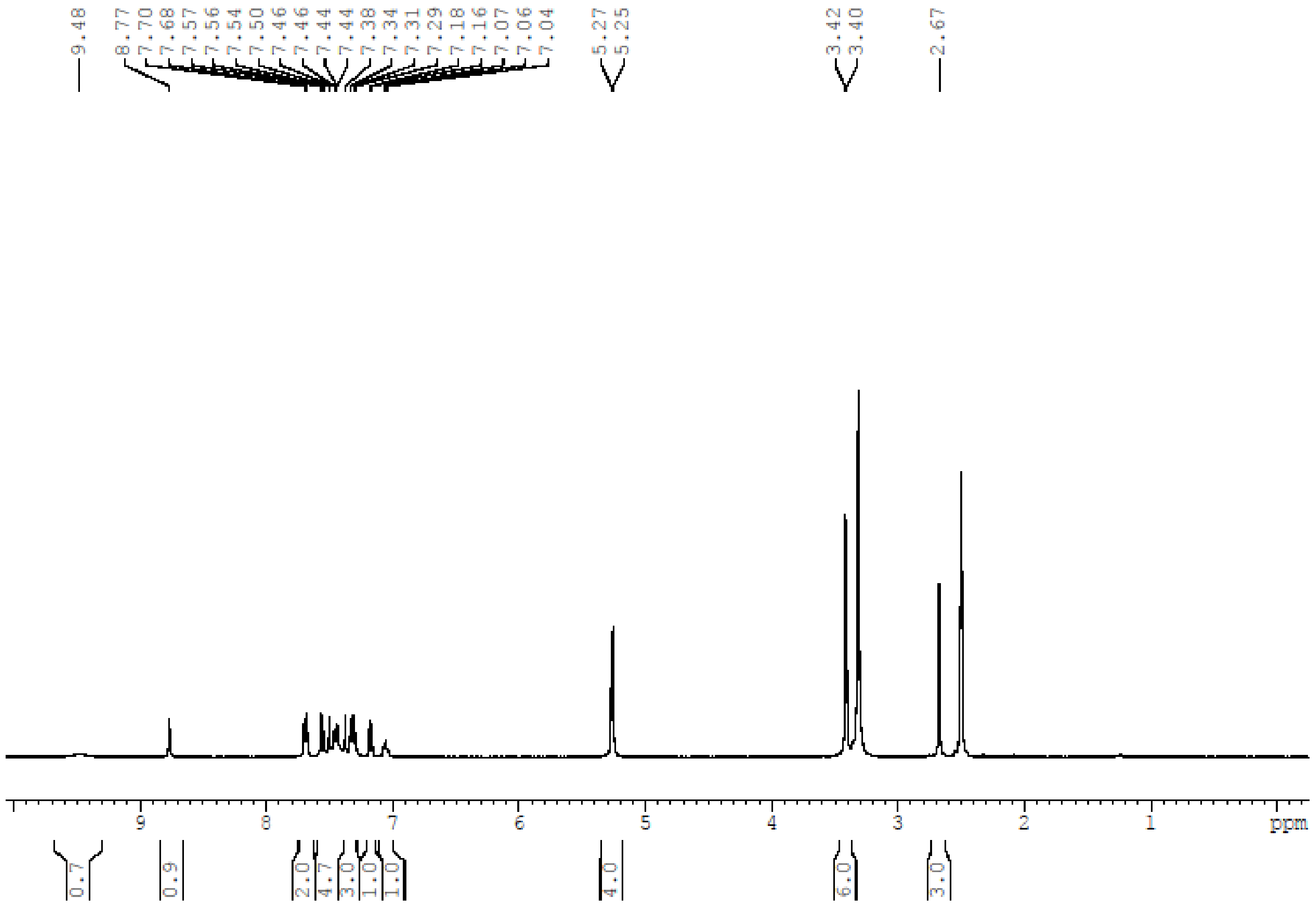

Figure S11: ^{1}H NMR spectrum of **10a** (400 MHz; DMSO-d_6).

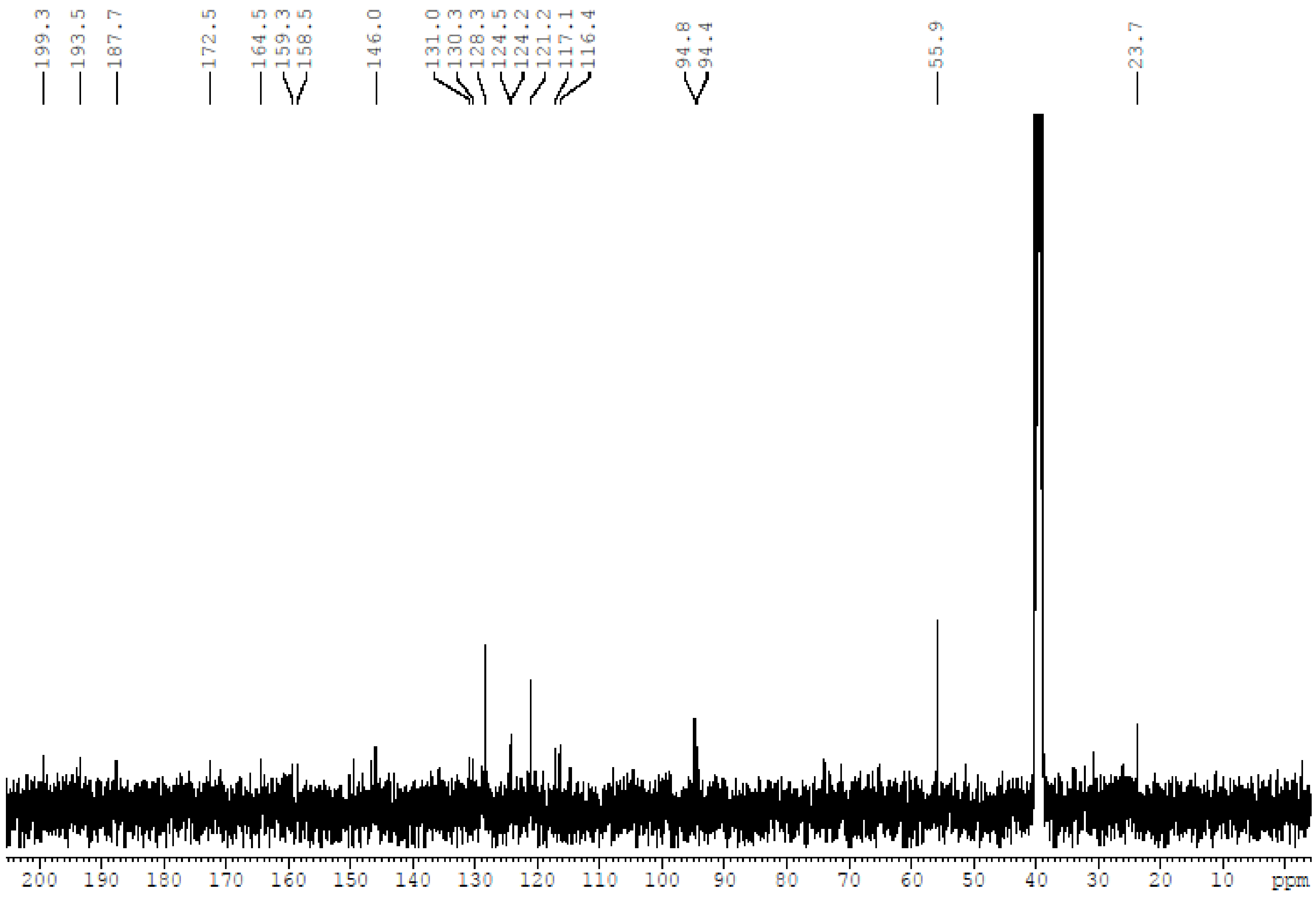

Figure S12: ^{13}C NMR spectrum of **10a** (100 MHz; DMSO-d_6).

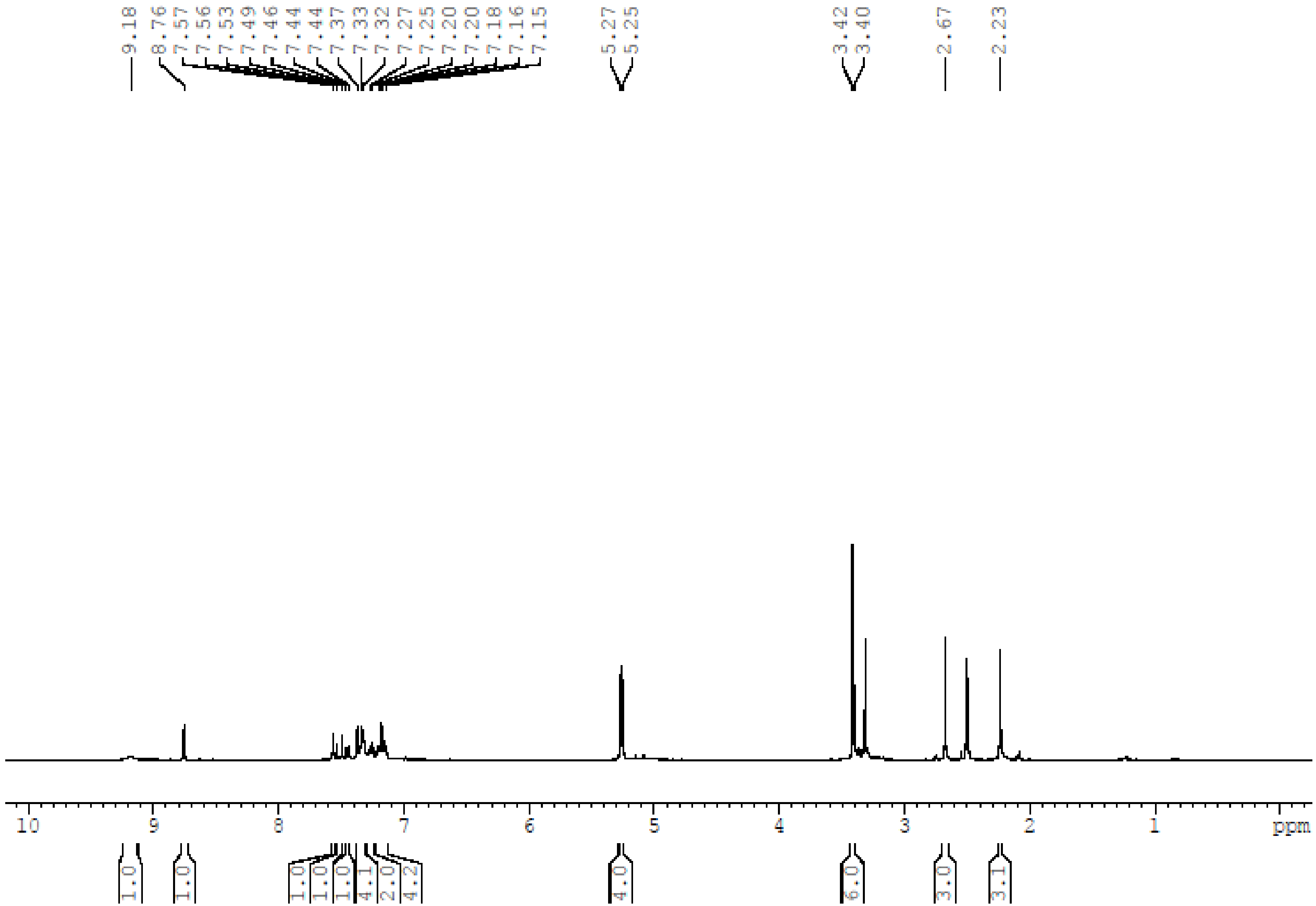

Figure S13: ^{1}H NMR spectrum of **10b** (400 MHz; DMSO-d_6).

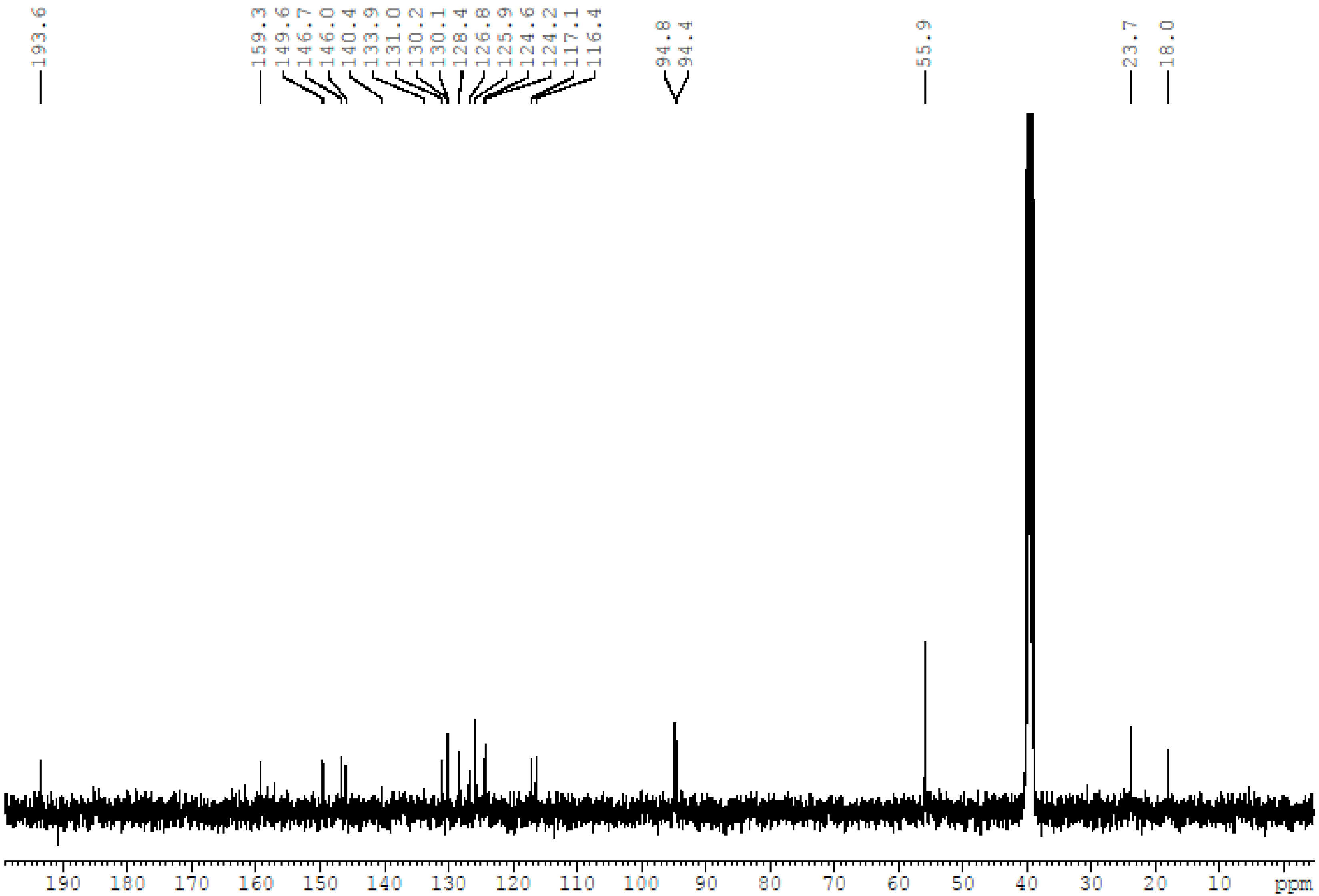

Figure S14: ^{13}C NMR spectrum of **10b** (100 MHz; DMSO-d_6).

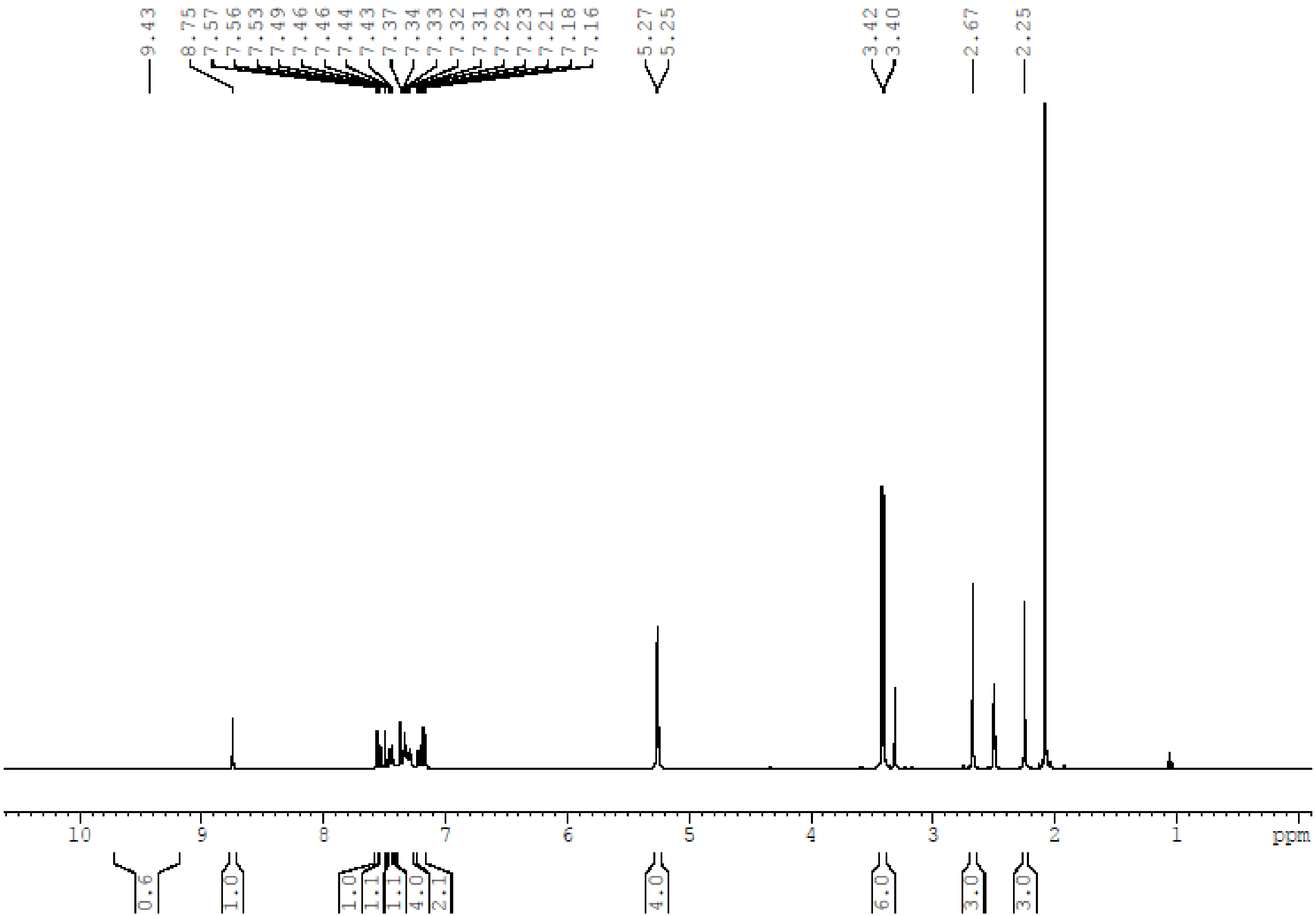

Figure 15: ^{1}H NMR spectrum of **10c** (400 MHz; DMSO-d_6).

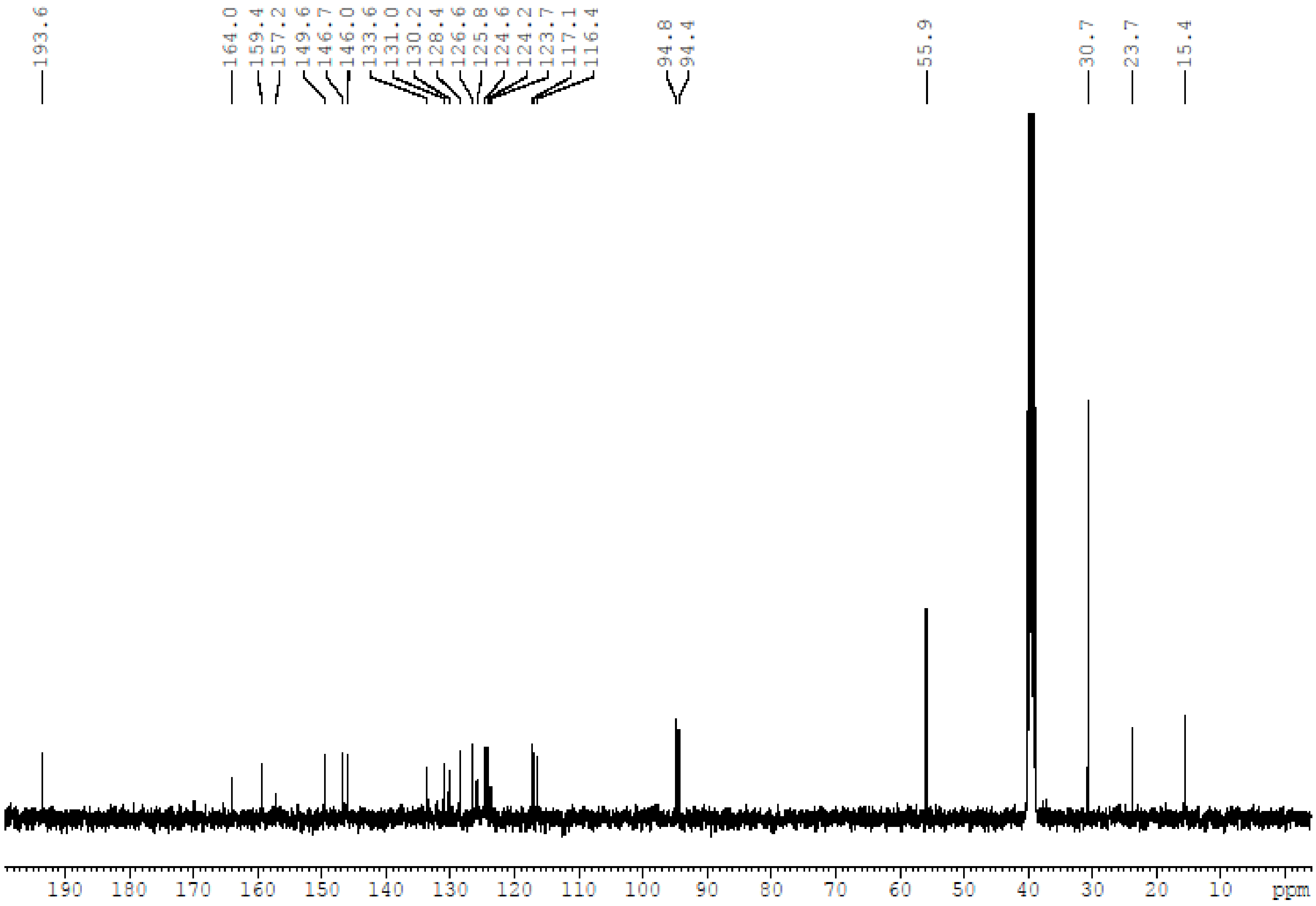

Figure S16: ^{13}C NMR spectrum of **10c** (100 MHz; DMSO-d_6).

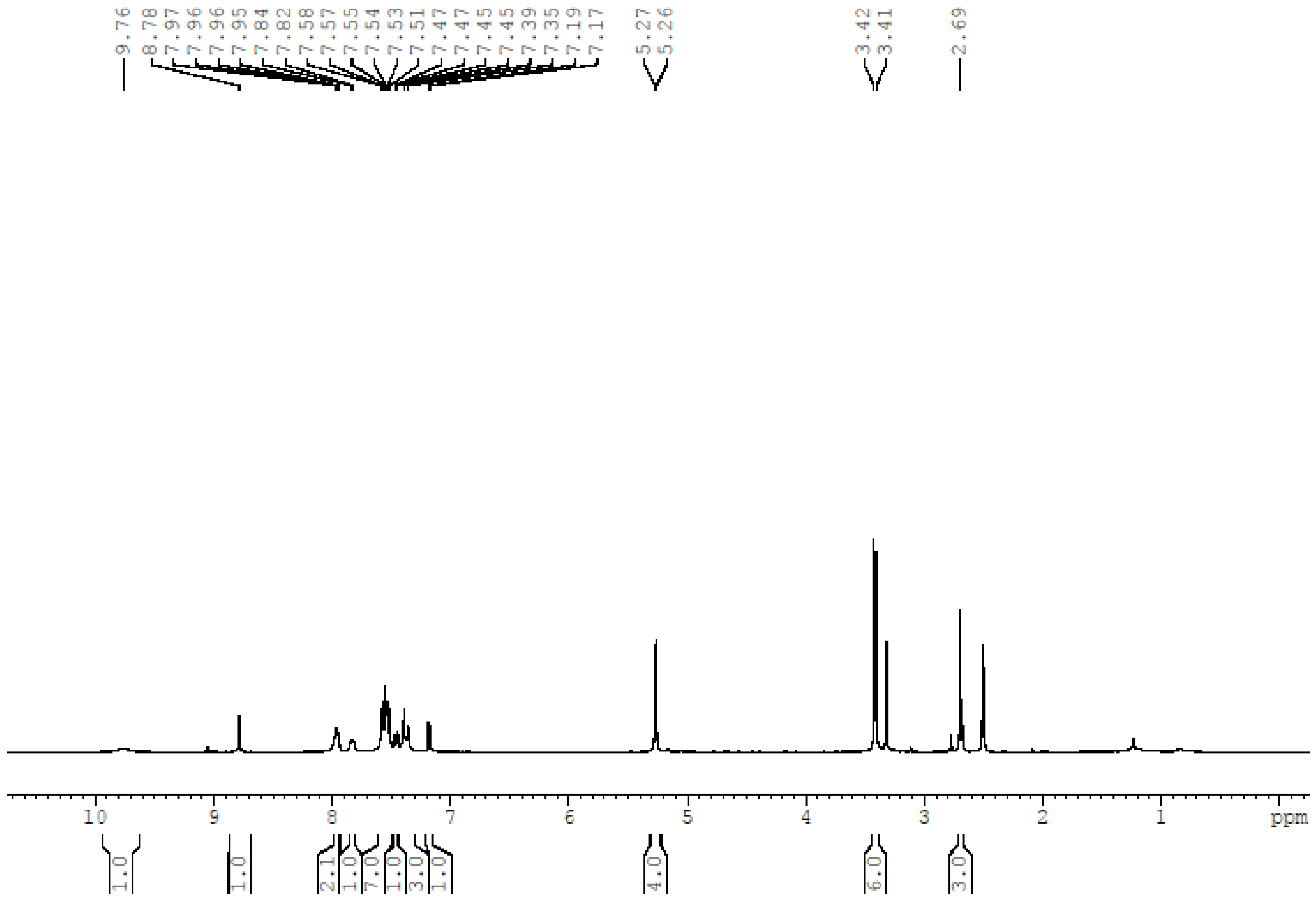

Figure 17: ^{1}H NMR spectrum of **10d** (400 MHz; DMSO-d_6).

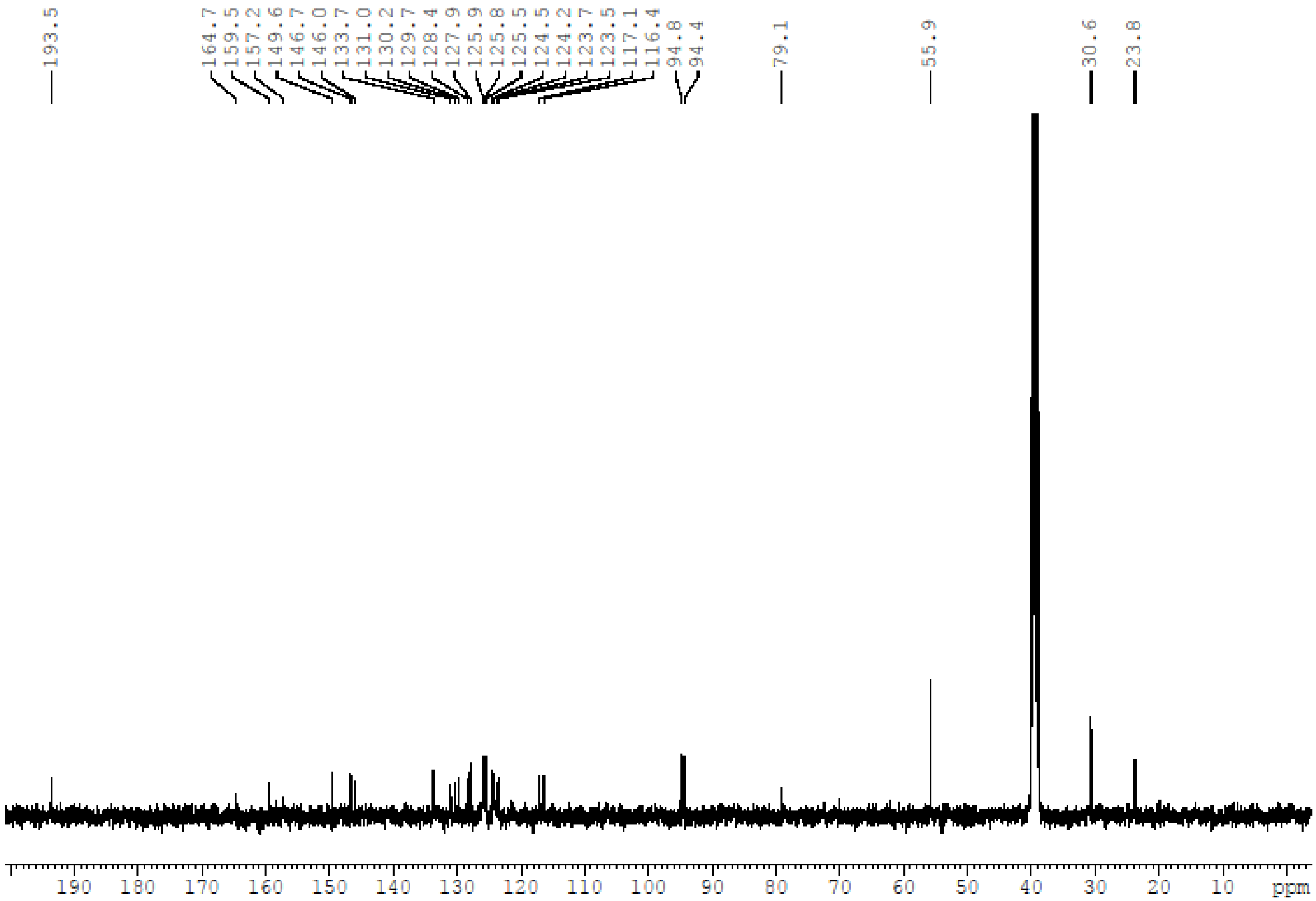

Figure S18: ^{13}C NMR spectrum of **10d** (100 MHz; DMSO-d_6).

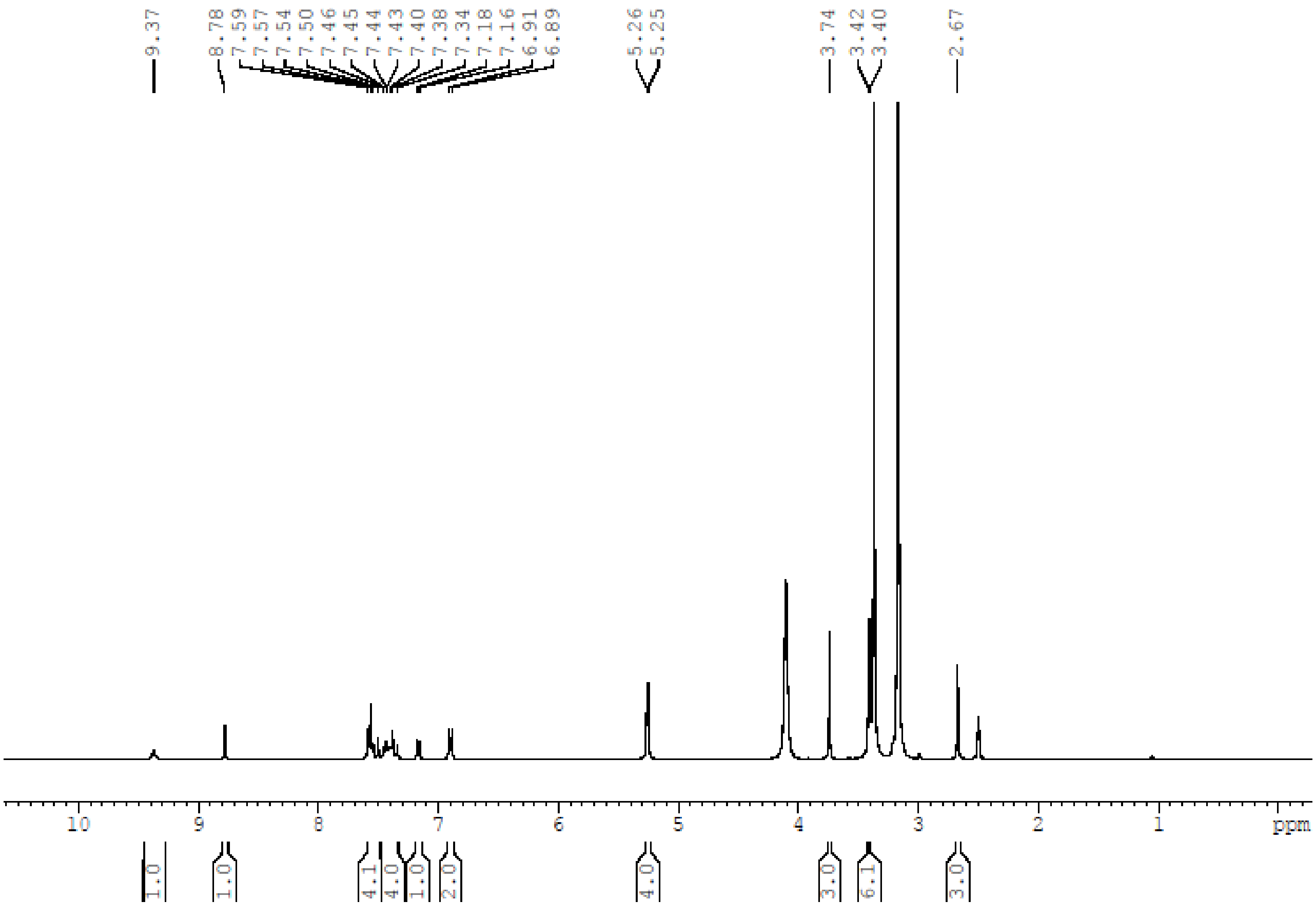

Figure S19: ^{1}H NMR spectrum of **10e** (400 MHz; DMSO-d_6).

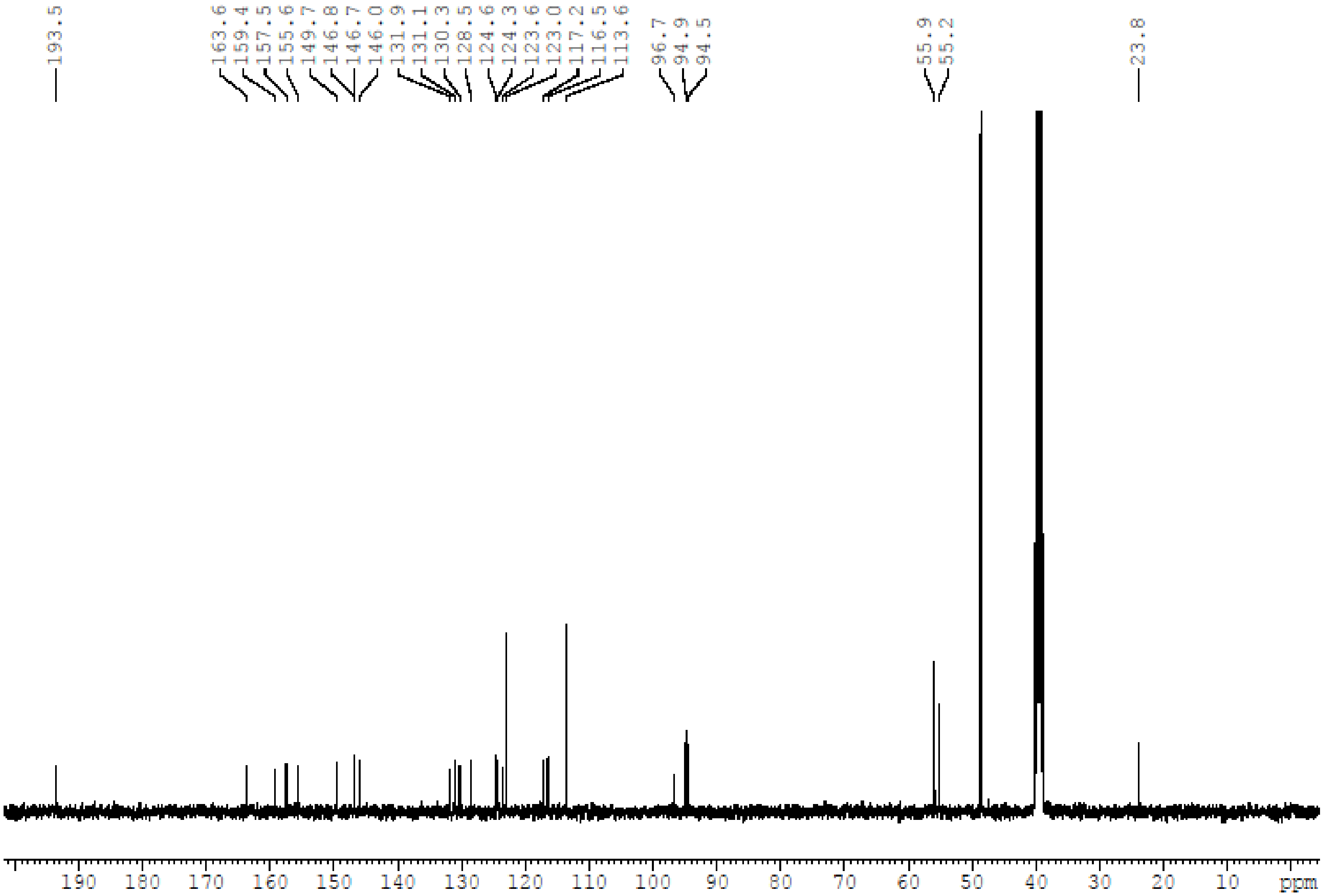

Figure S20: ^{13}C NMR spectrum of **10e** (100 MHz; DMSO-d_6).

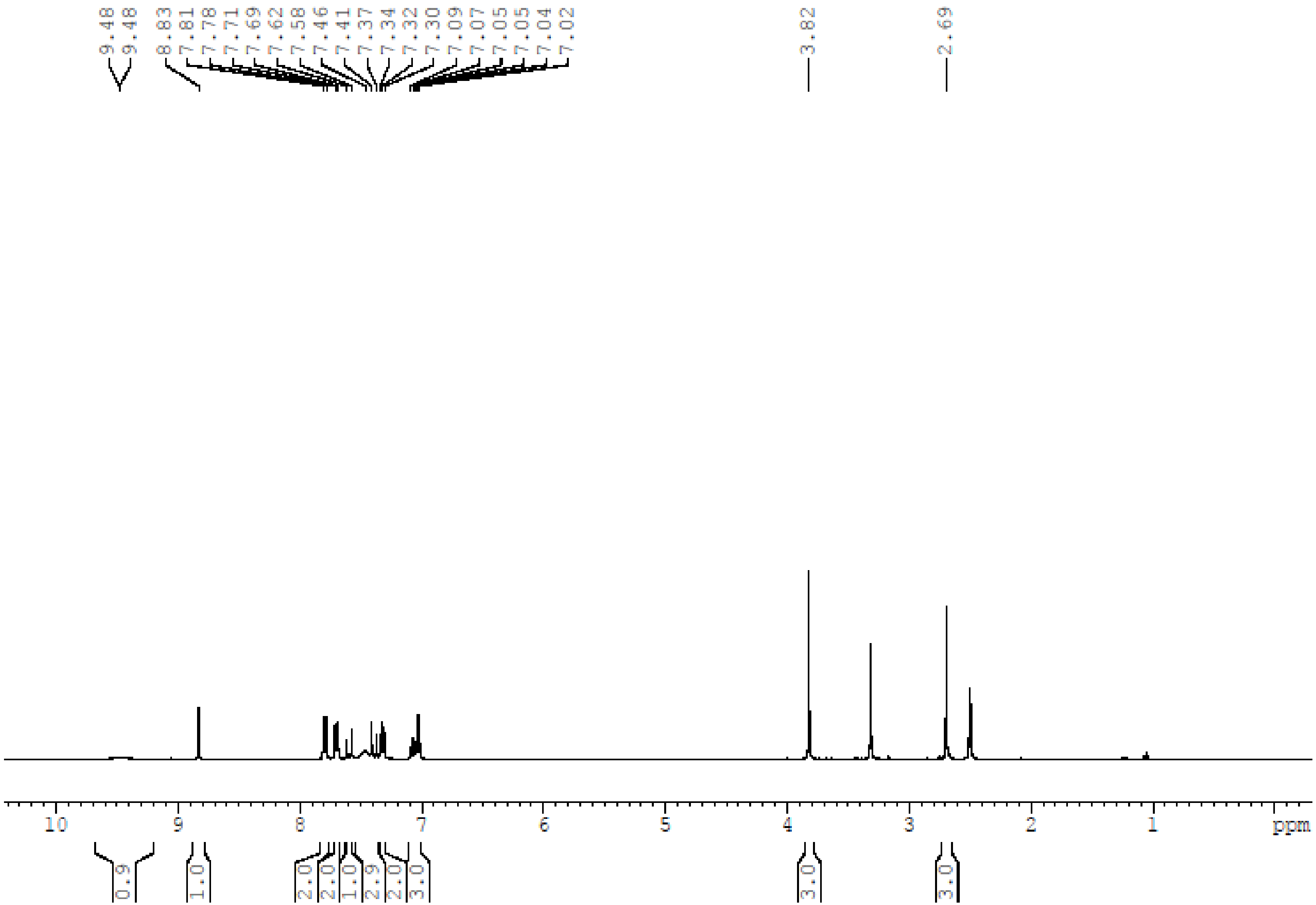

Figure S21: ^{1}H NMR spectrum of **11a** (400 MHz; DMSO-d_6).

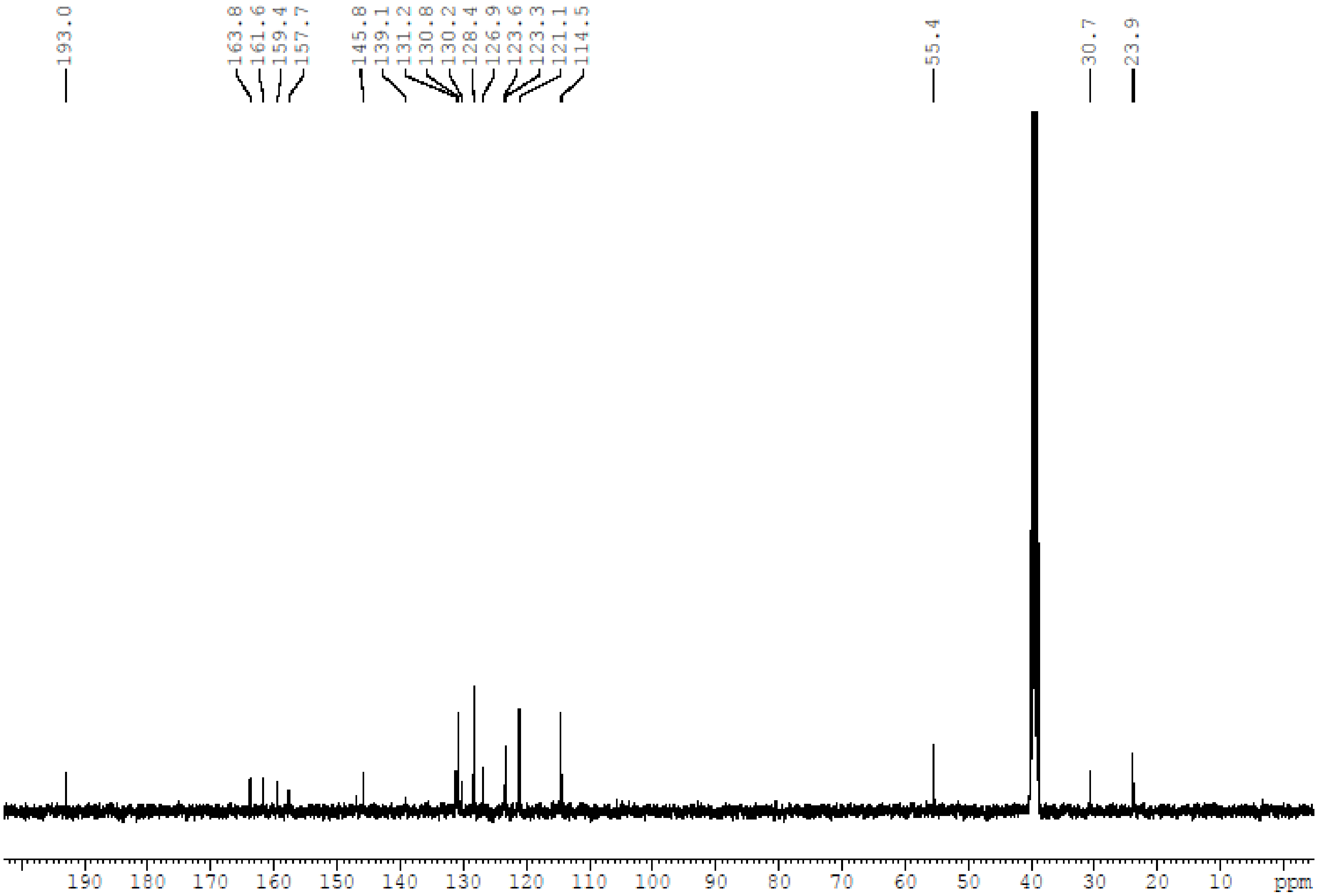

Figure S22: ^{13}C NMR spectrum of **11a** (100 MHz; DMSO-d_6).

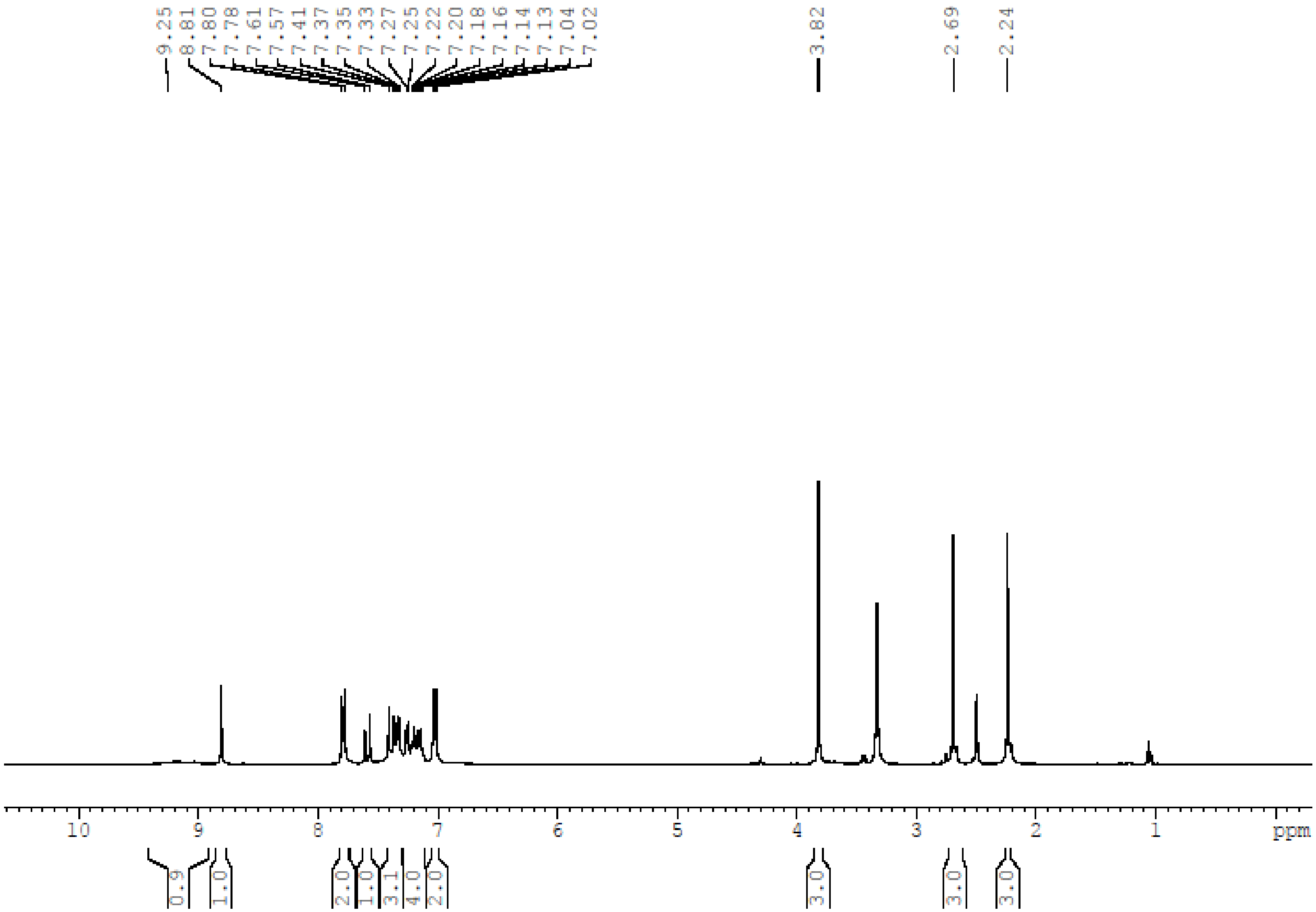

Figure S23: ^{1}H NMR spectrum of **11b** (400 MHz; DMSO-d_6).

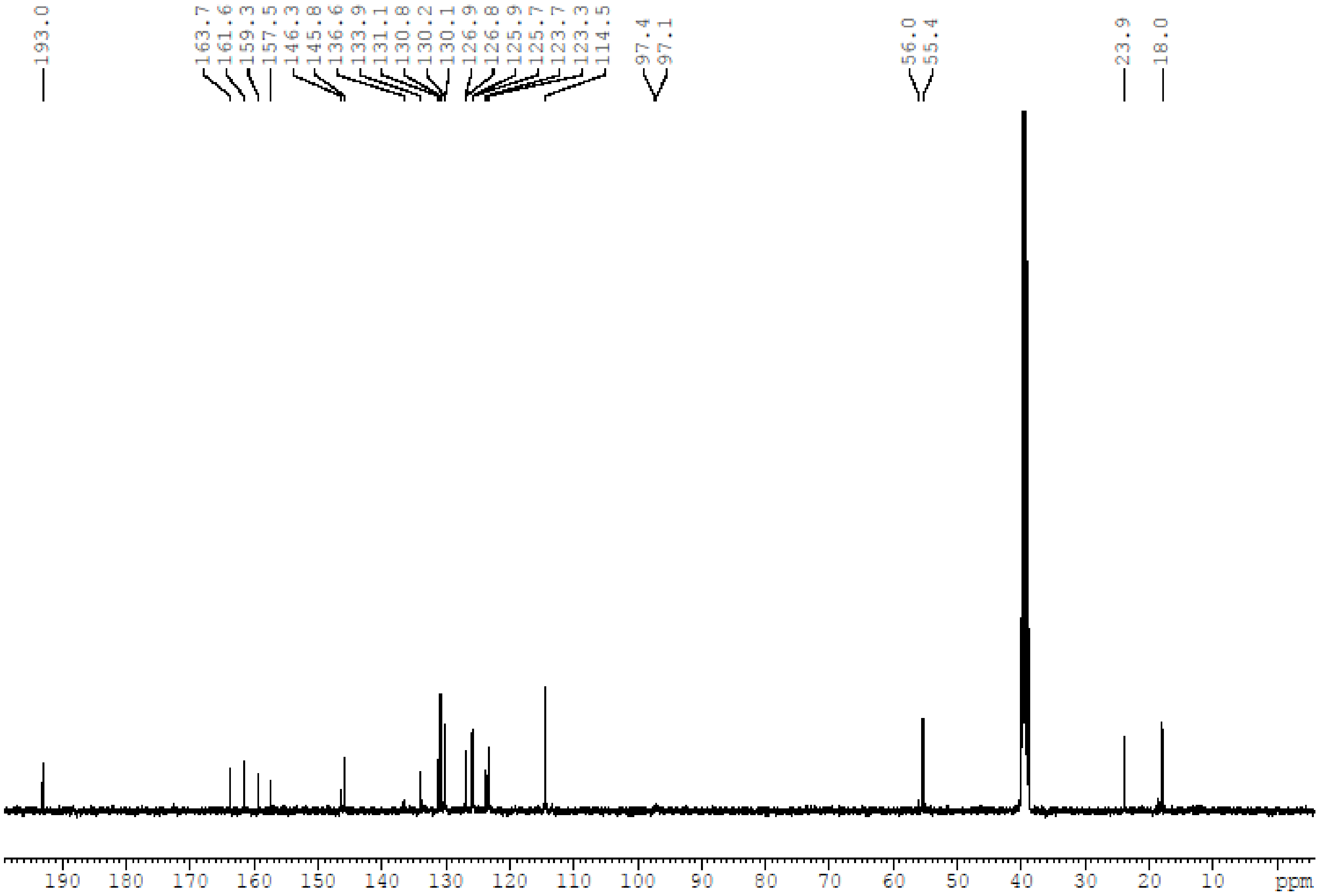

Figure S24: ^{13}C NMR spectrum of **11b** (100 MHz; DMSO-d_6).

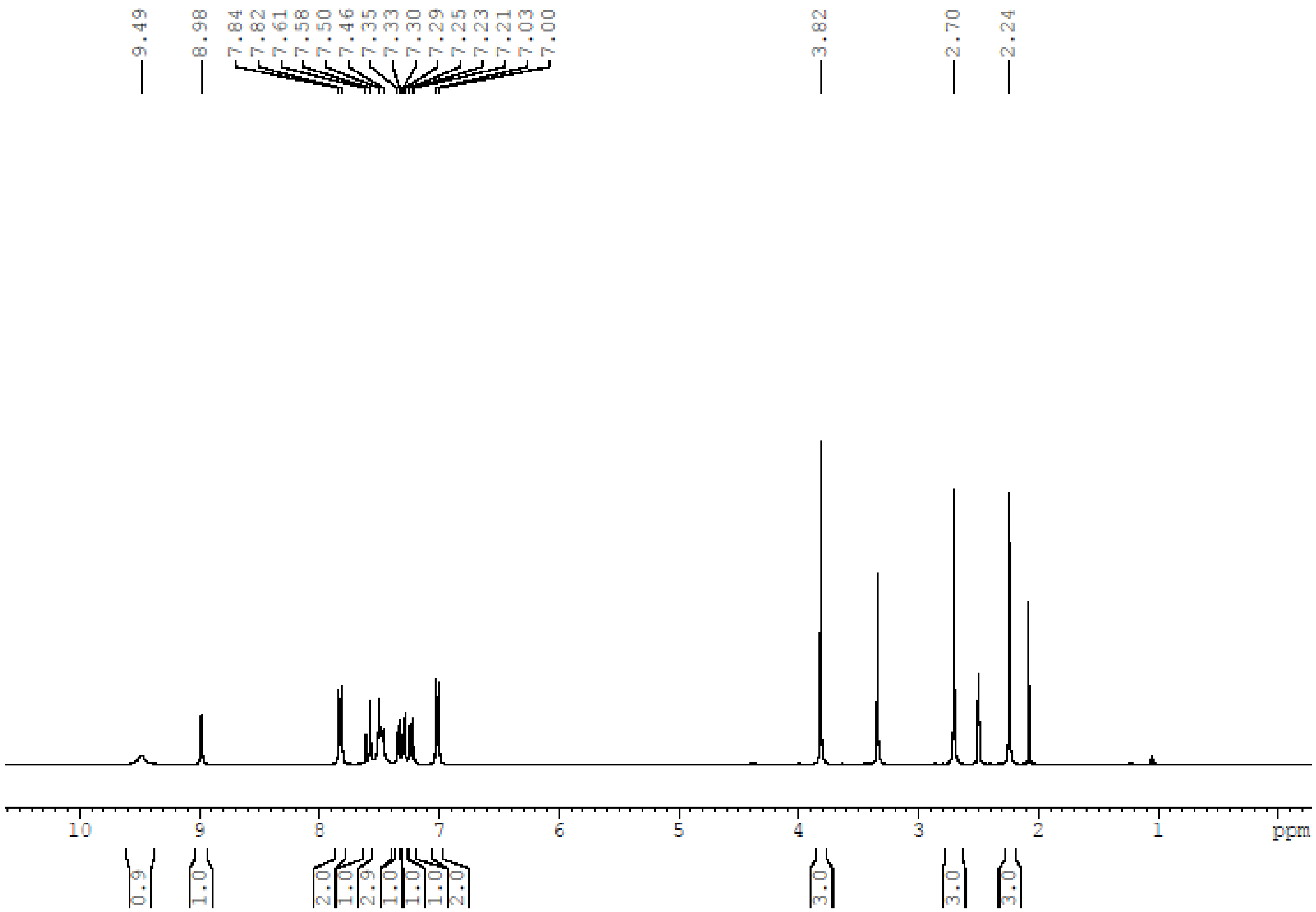

Figure S25: ^{1}H NMR spectrum of **11c** (400 MHz; DMSO-d_6).

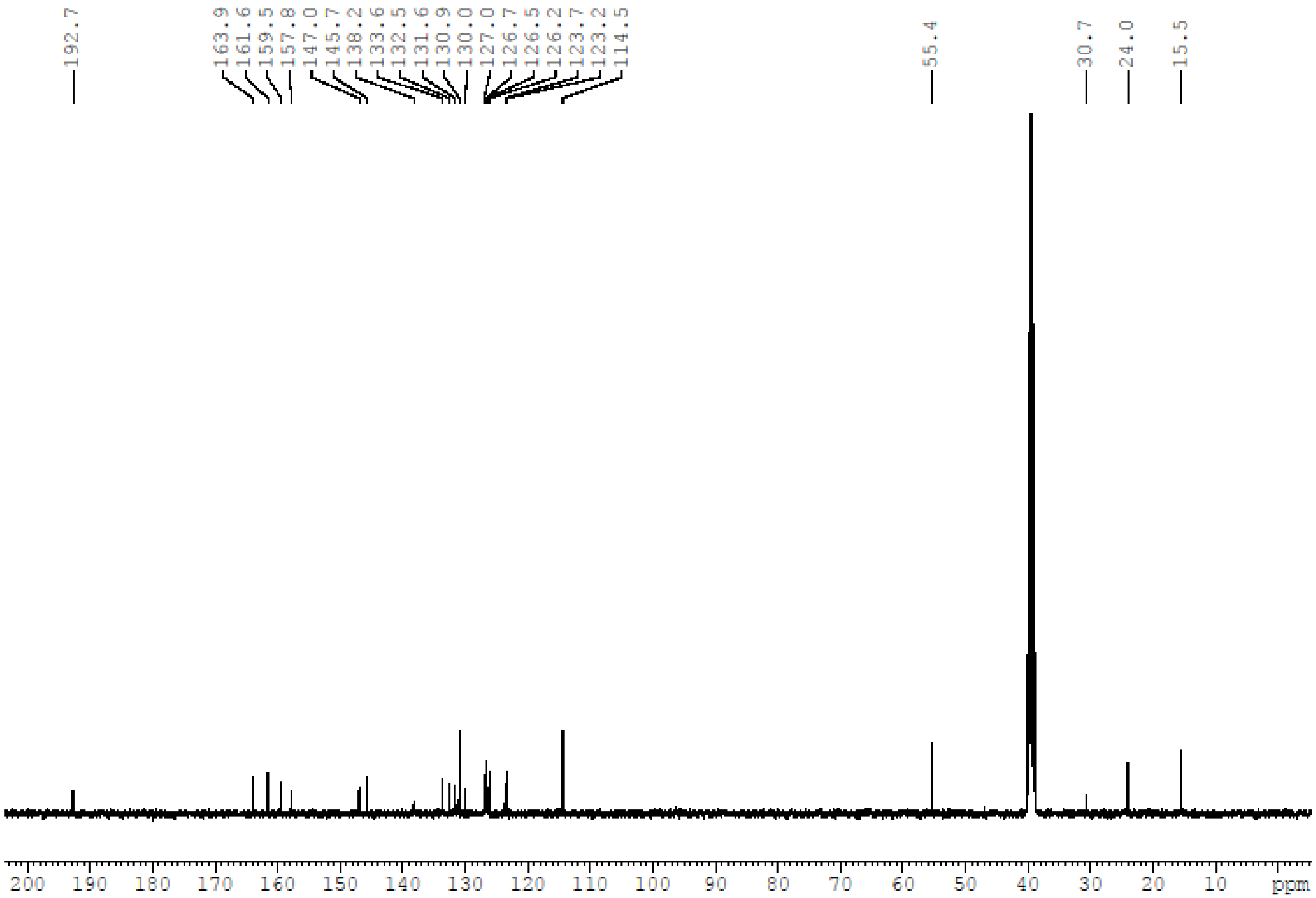

Figure S26: ^{13}C NMR spectrum of **11c** (100 MHz; DMSO-d_6).

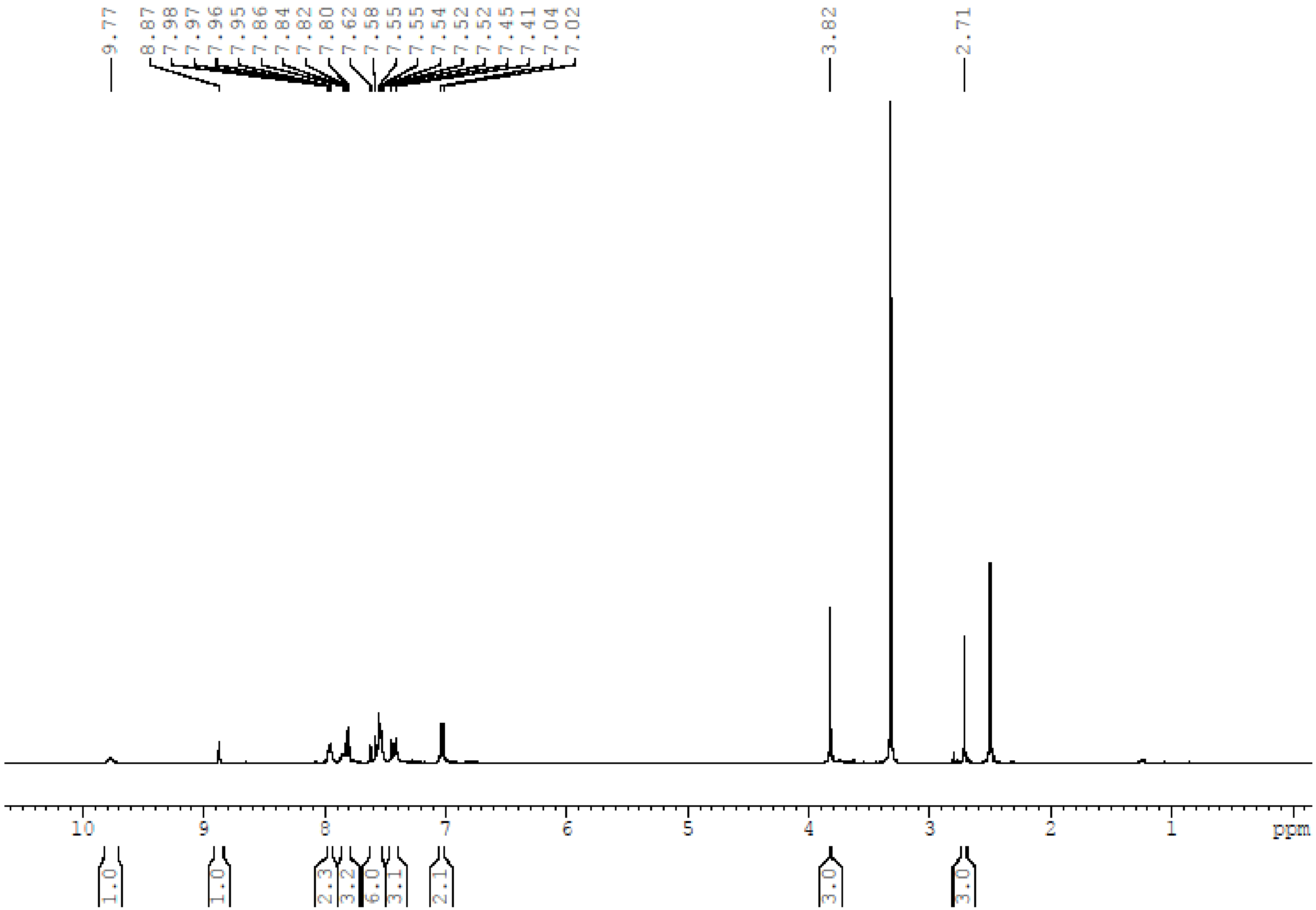

Figure S27: ^{1}H NMR spectrum of **11d** (400 MHz; DMSO-d_6).

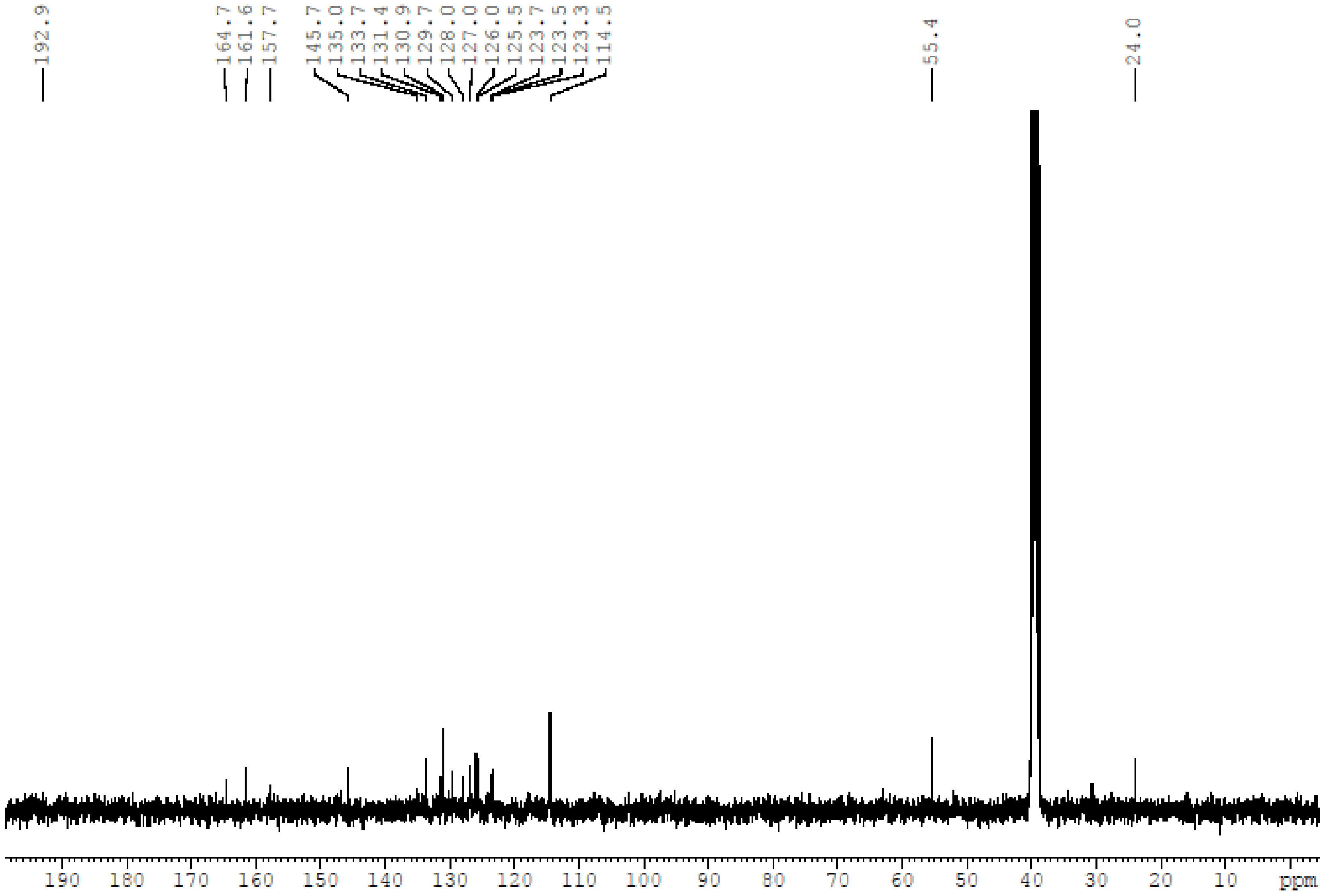

Figure S28: ^{13}C NMR spectrum of **11d** (100 MHz; DMSO-d_6).

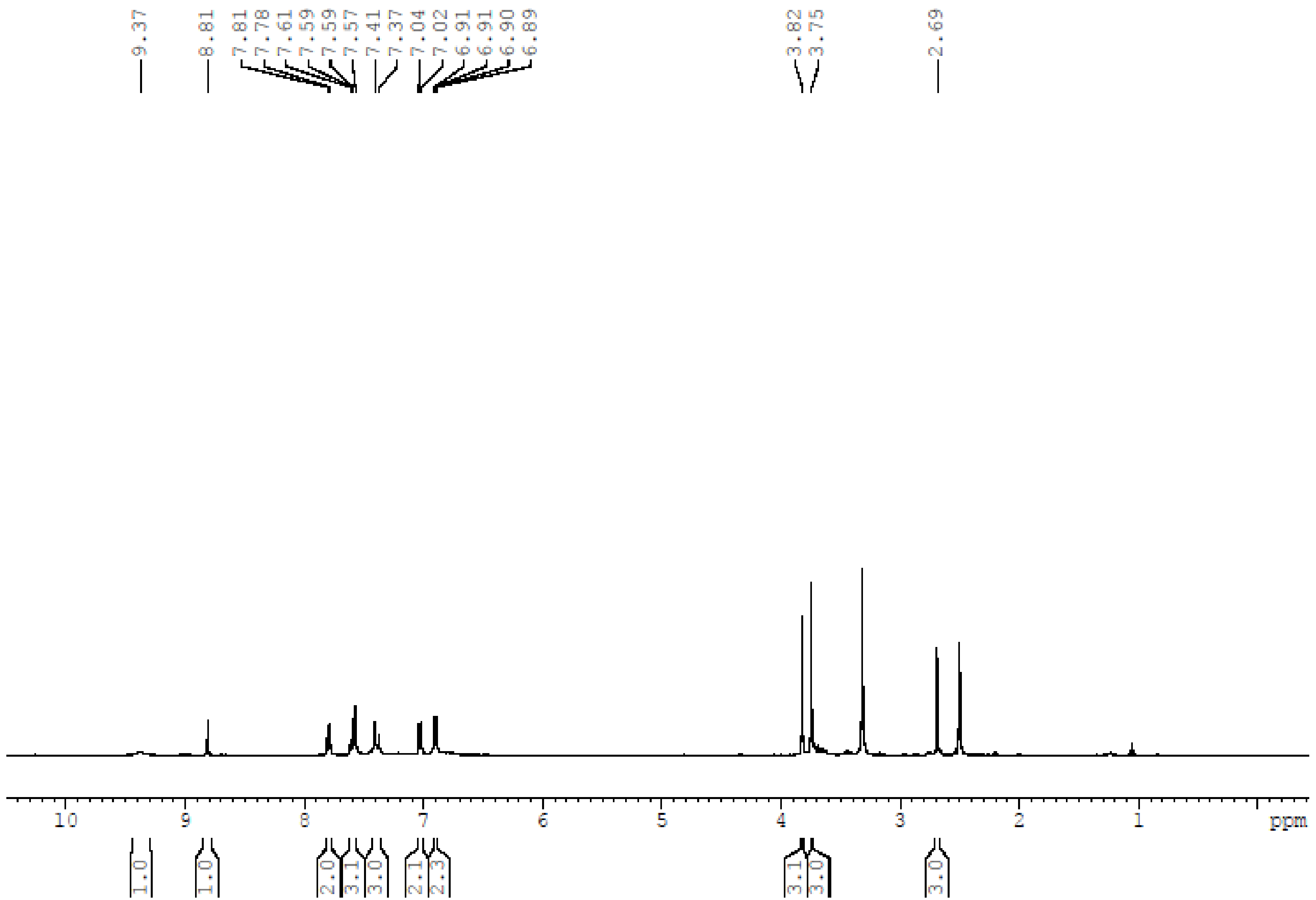

Figure S29: ^{1}H NMR spectrum of **11e** (400 MHz; DMSO-d_6).

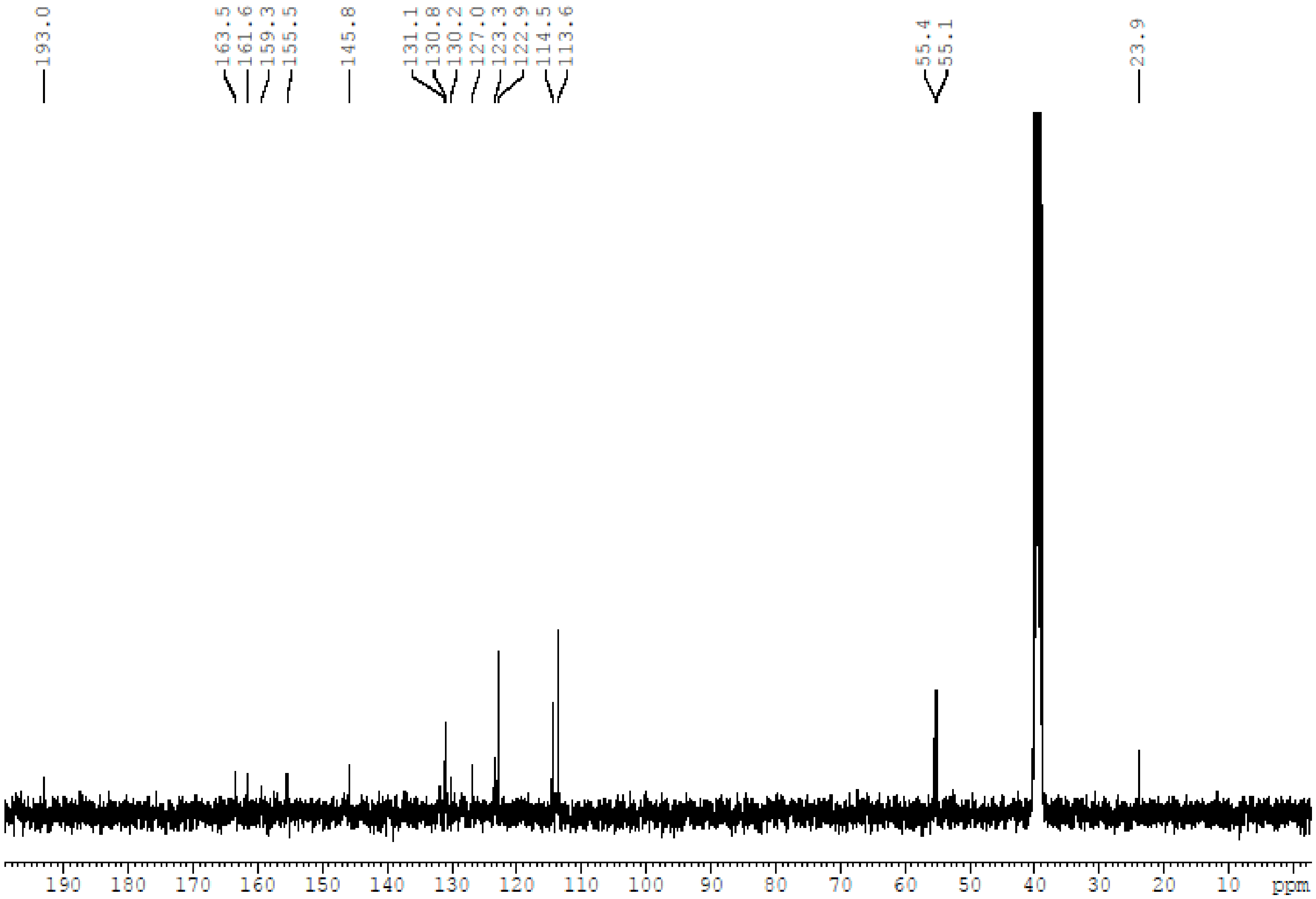

Figure S30: ^{13}C NMR spectrum of **11e** (100 MHz; DMSO-d_6).

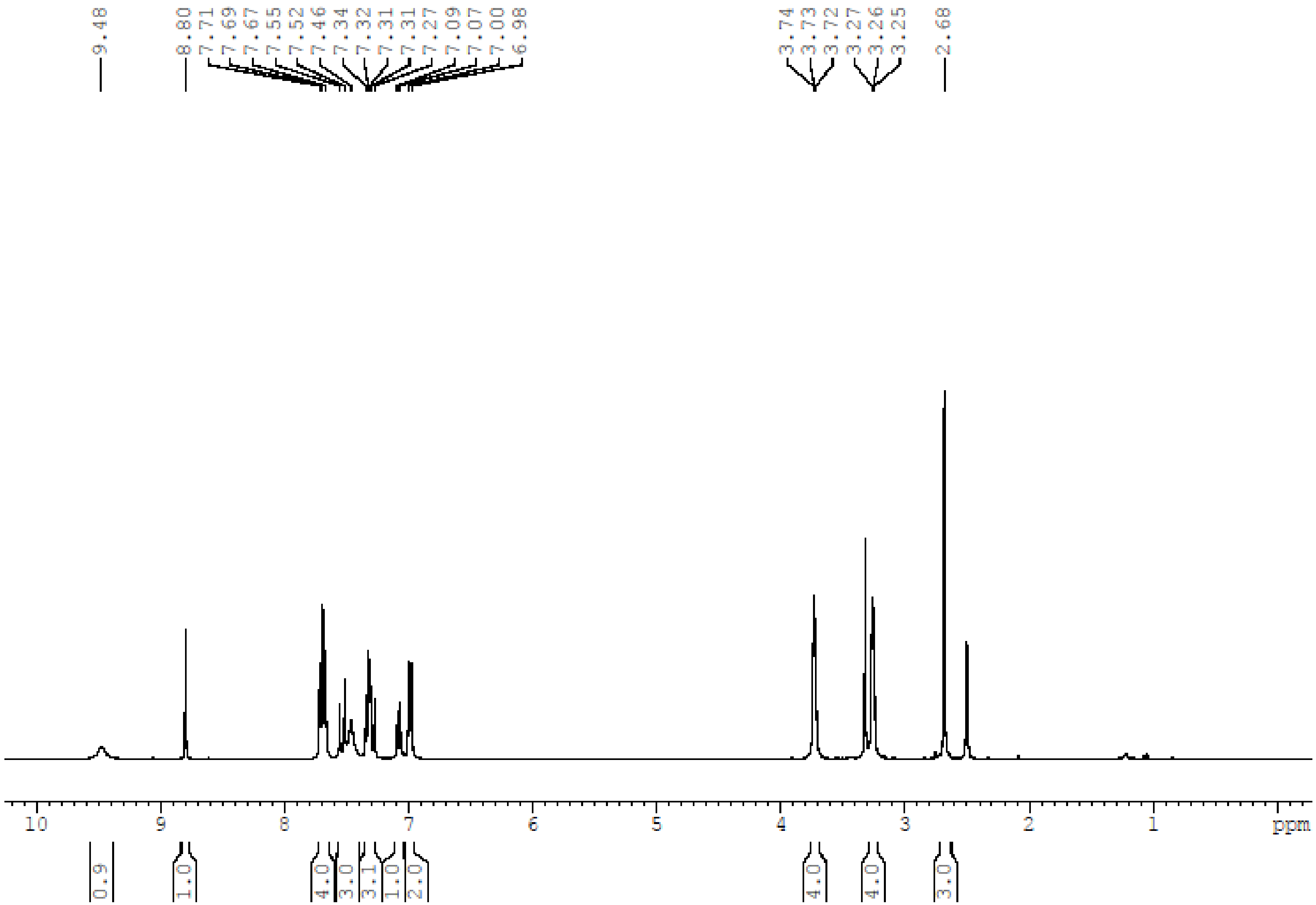

Figure S31: ^{1}H NMR spectrum of **12a** (400 MHz; DMSO-d_6).

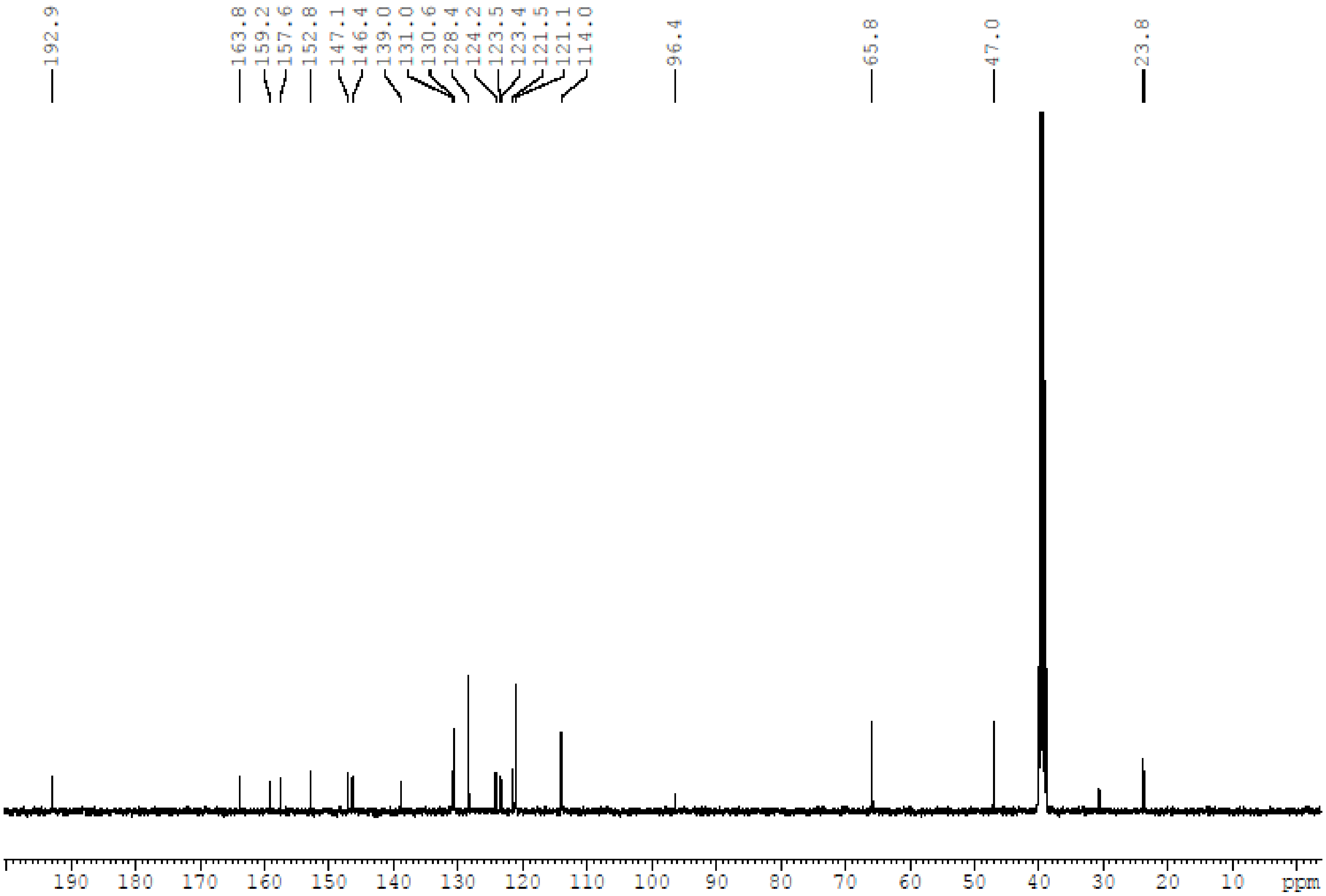

Figure S32: ^{13}C NMR spectrum of **12a** (100 MHz; DMSO-d_6).

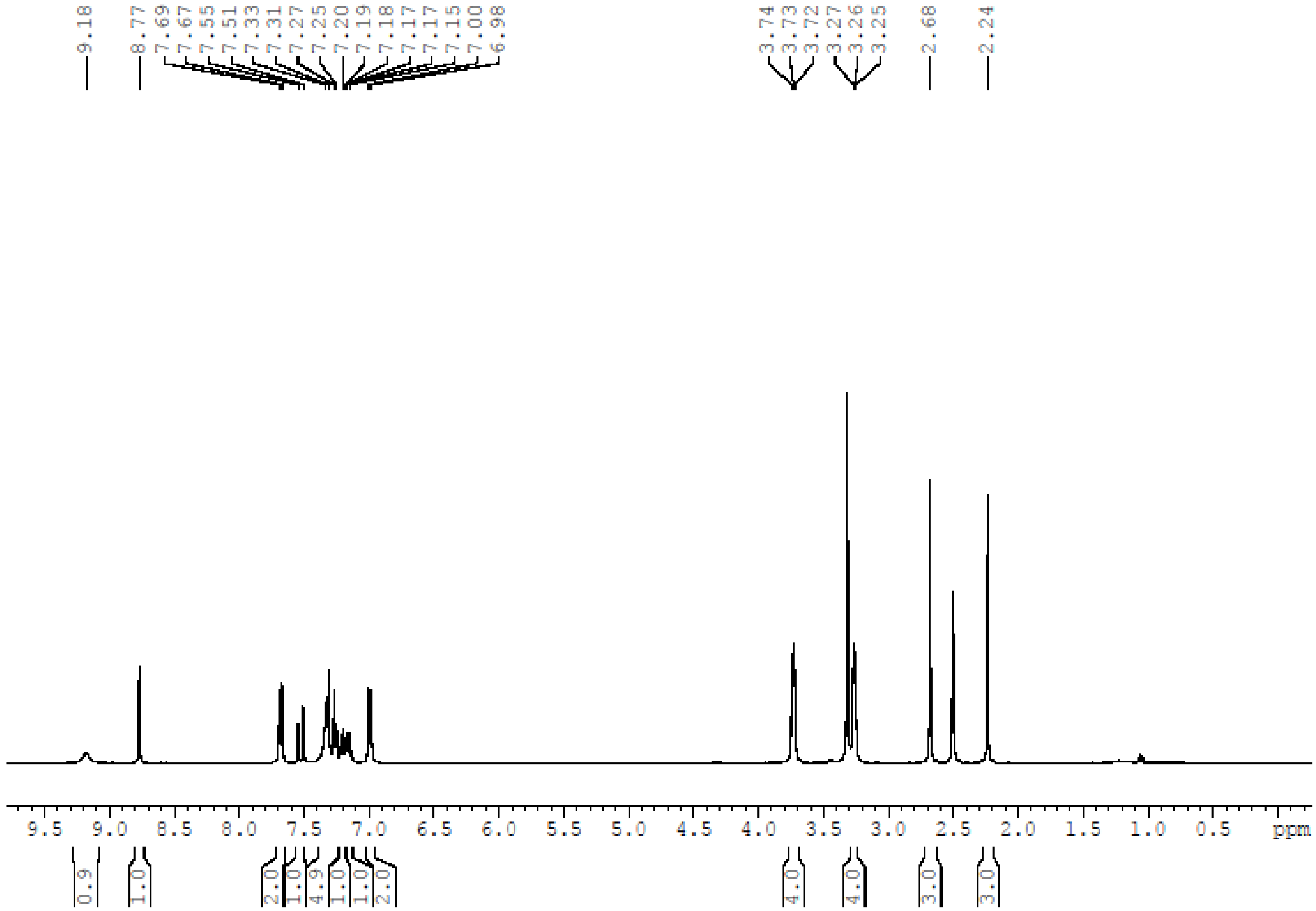

Figure S33: ^{1}H NMR spectrum of **12b** (400 MHz; DMSO-d_6).

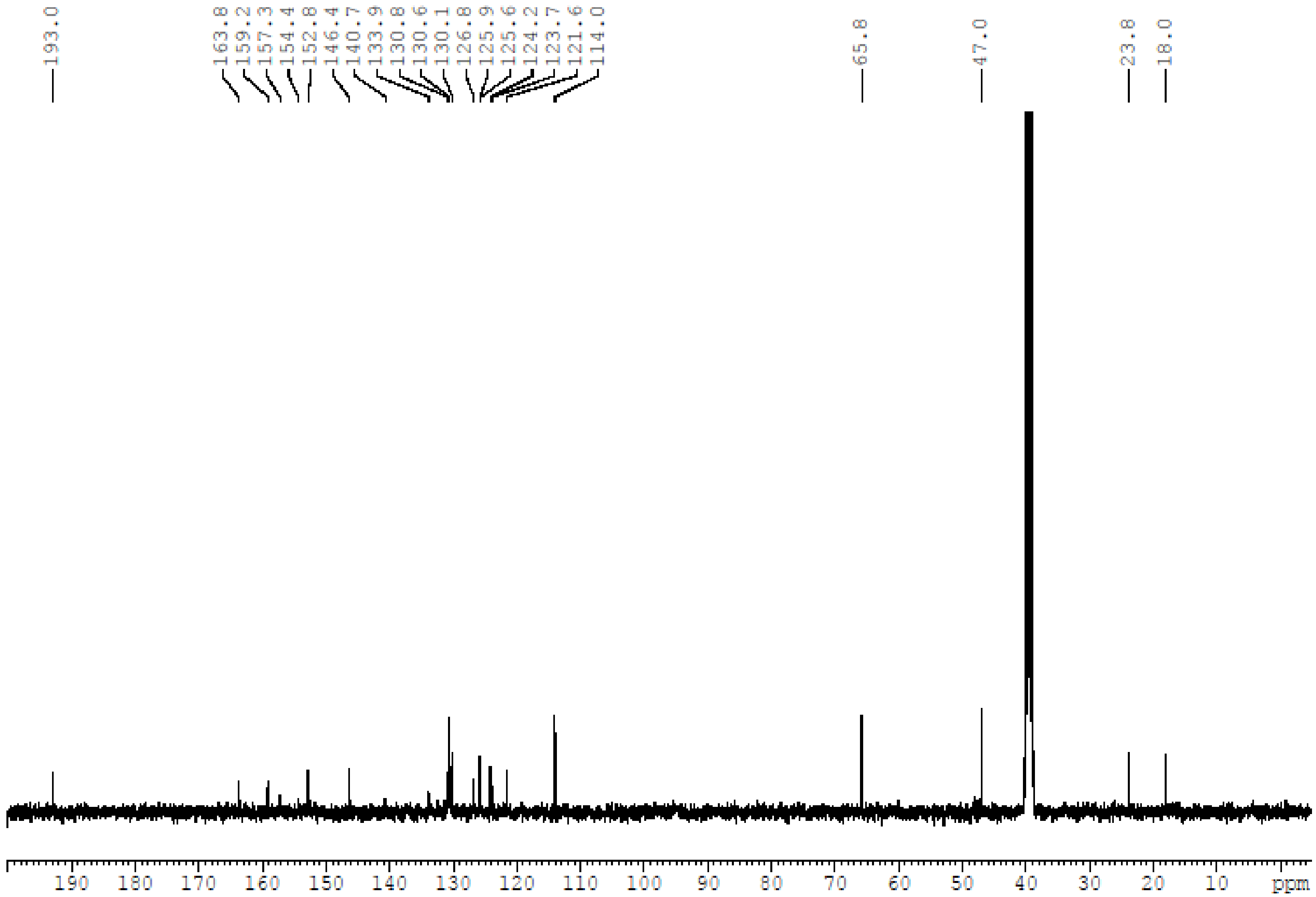

Figure S34: ^{13}C NMR spectrum of **12b** (100 MHz; DMSO-d_6).

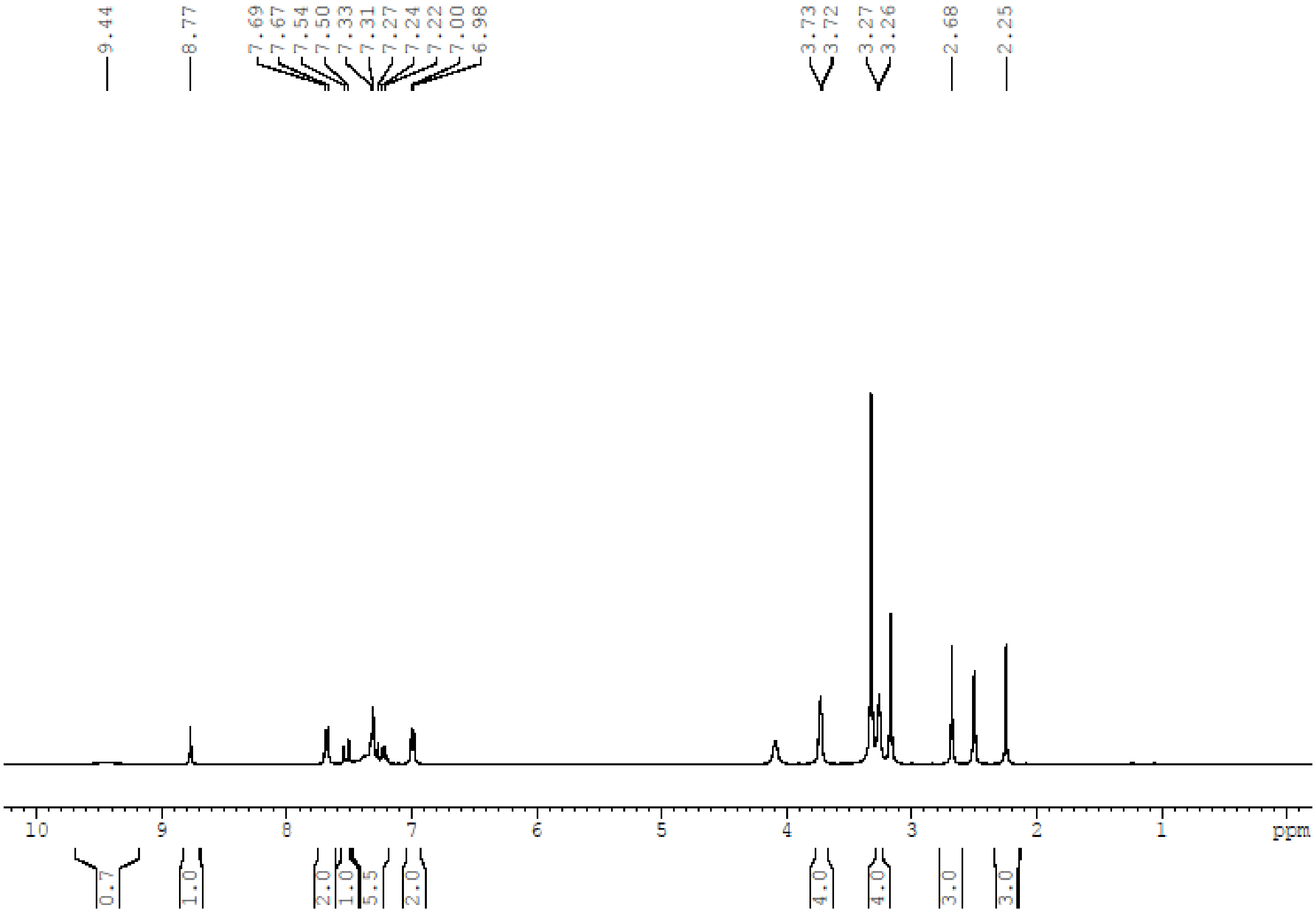

Figure S35: ^{1}H NMR spectrum of **12c** (400 MHz; DMSO-*d*$_6$).

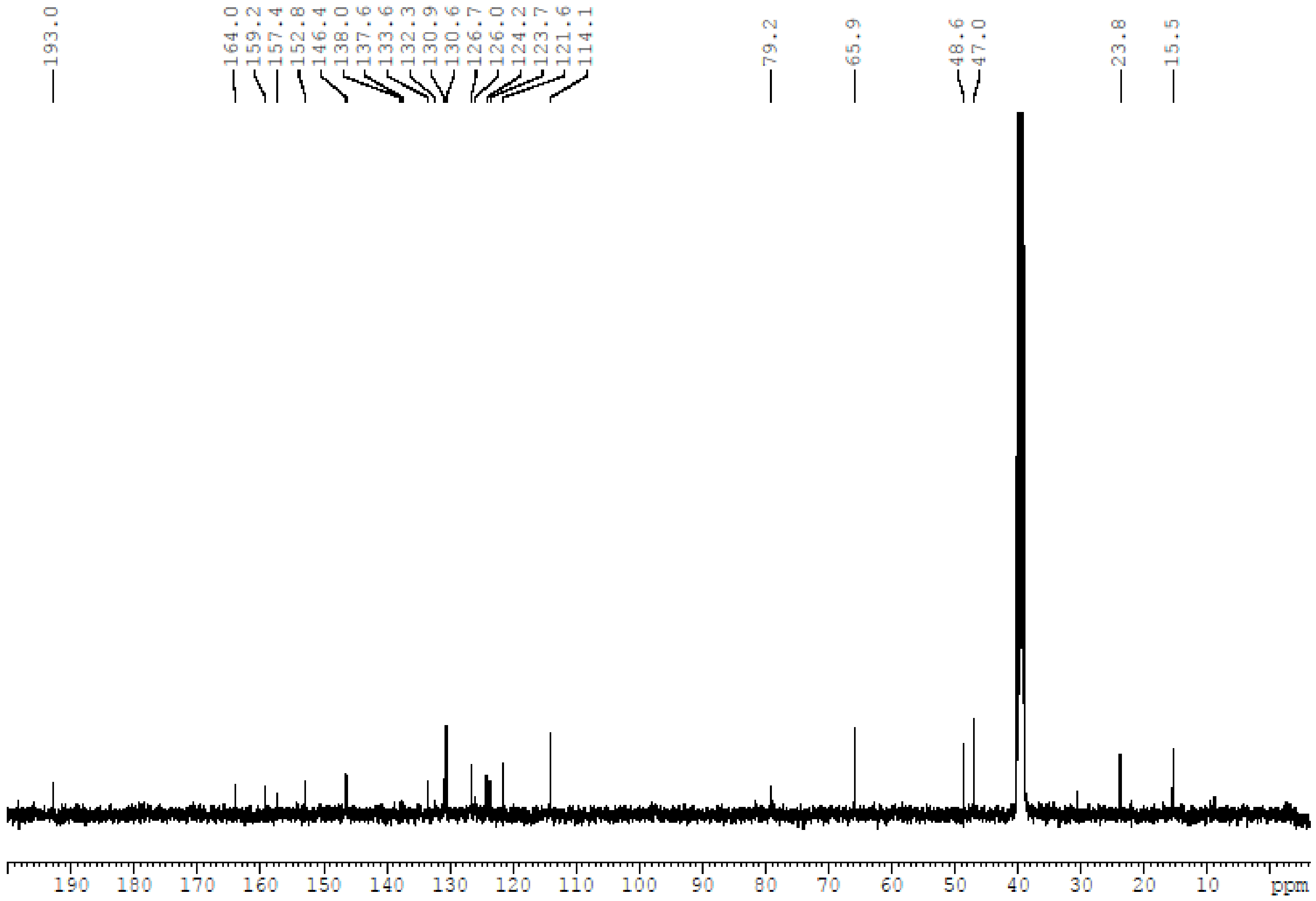

Figure S36: ^{13}C NMR spectrum of **12c** (100 MHz; DMSO-d_6).

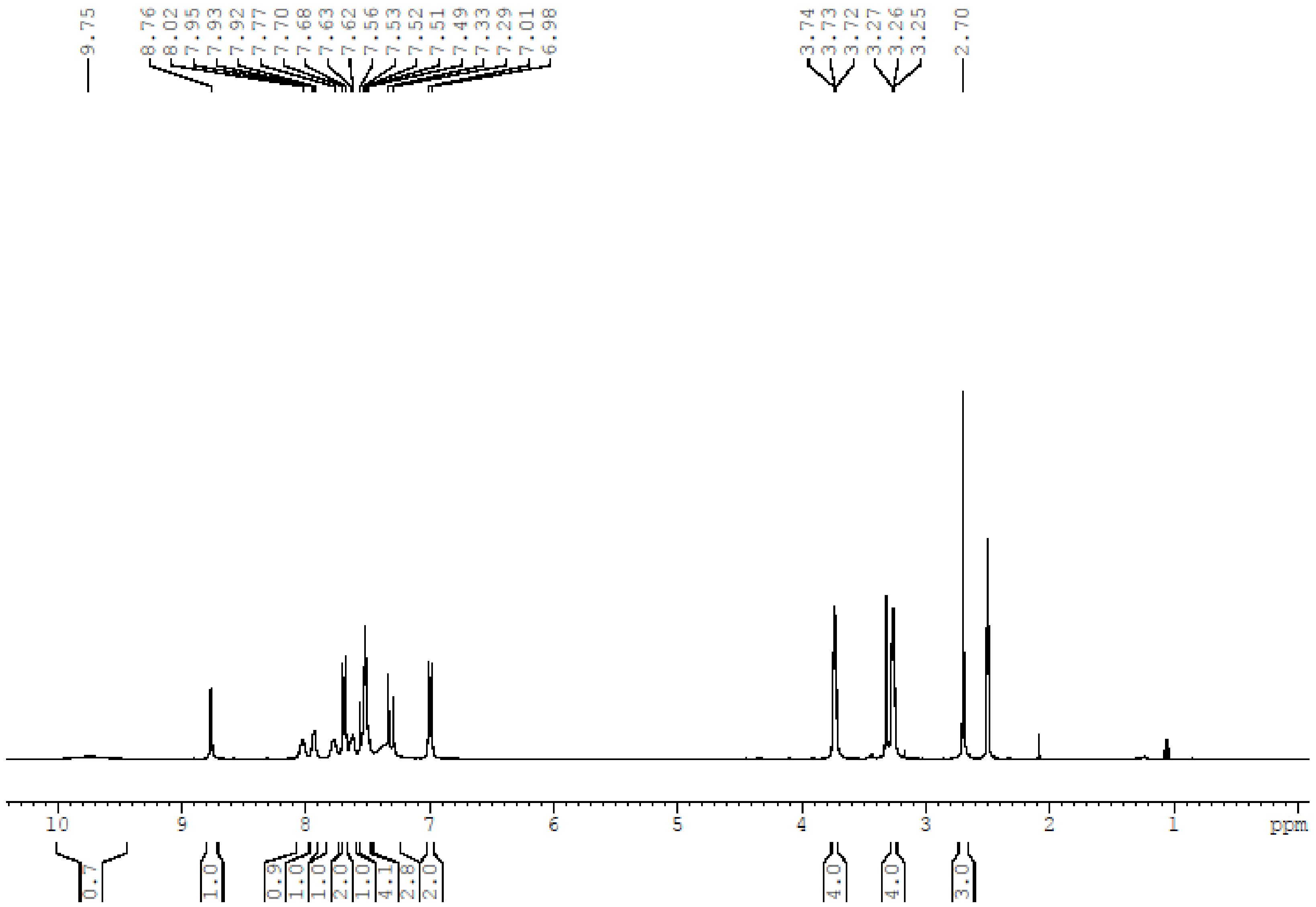

Figure S37: ^{1}H NMR spectrum of **12d** (400 MHz; DMSO-d_6).

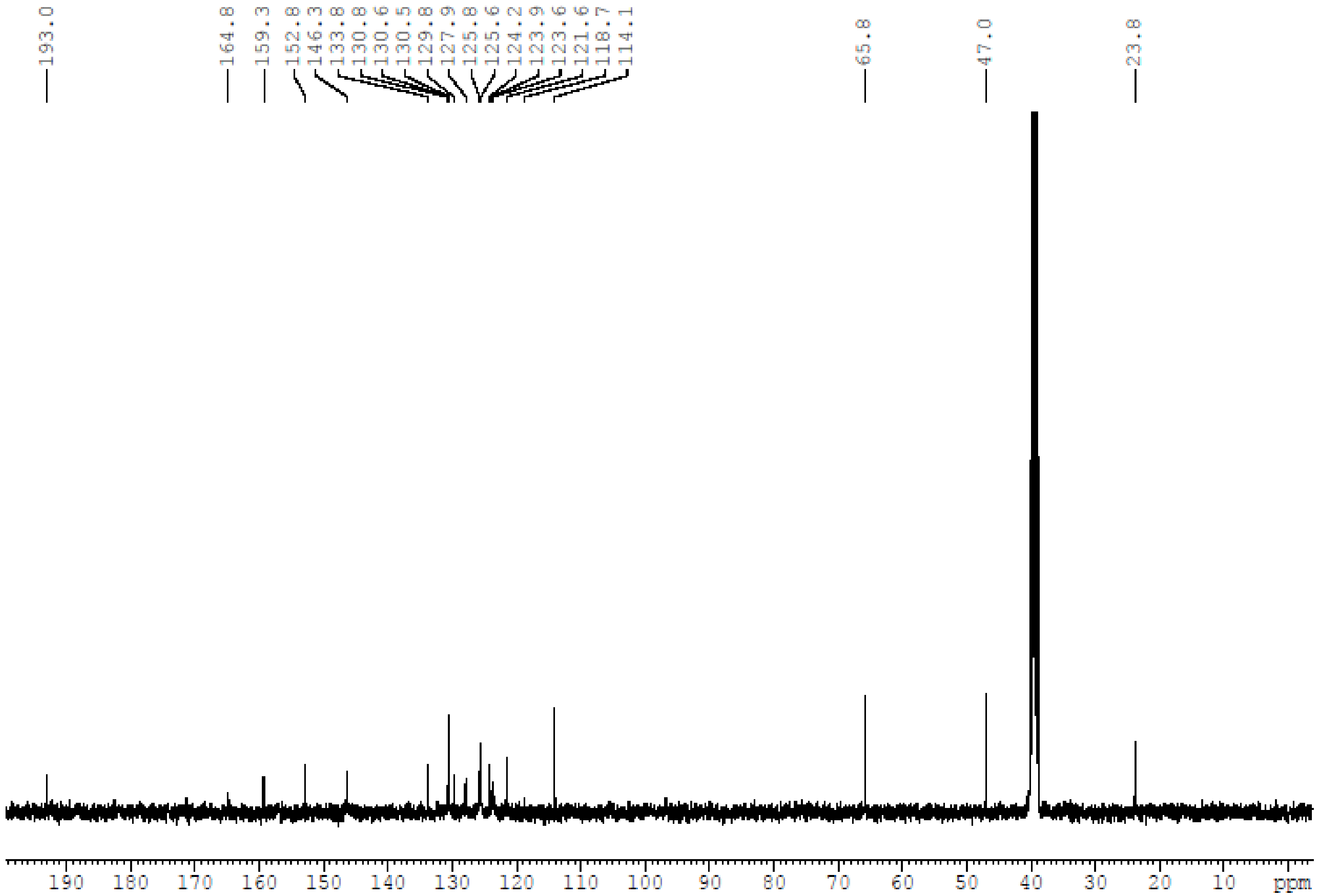

Figure S38: ^{13}C NMR spectrum of **12d** (100 MHz; DMSO-d_6).

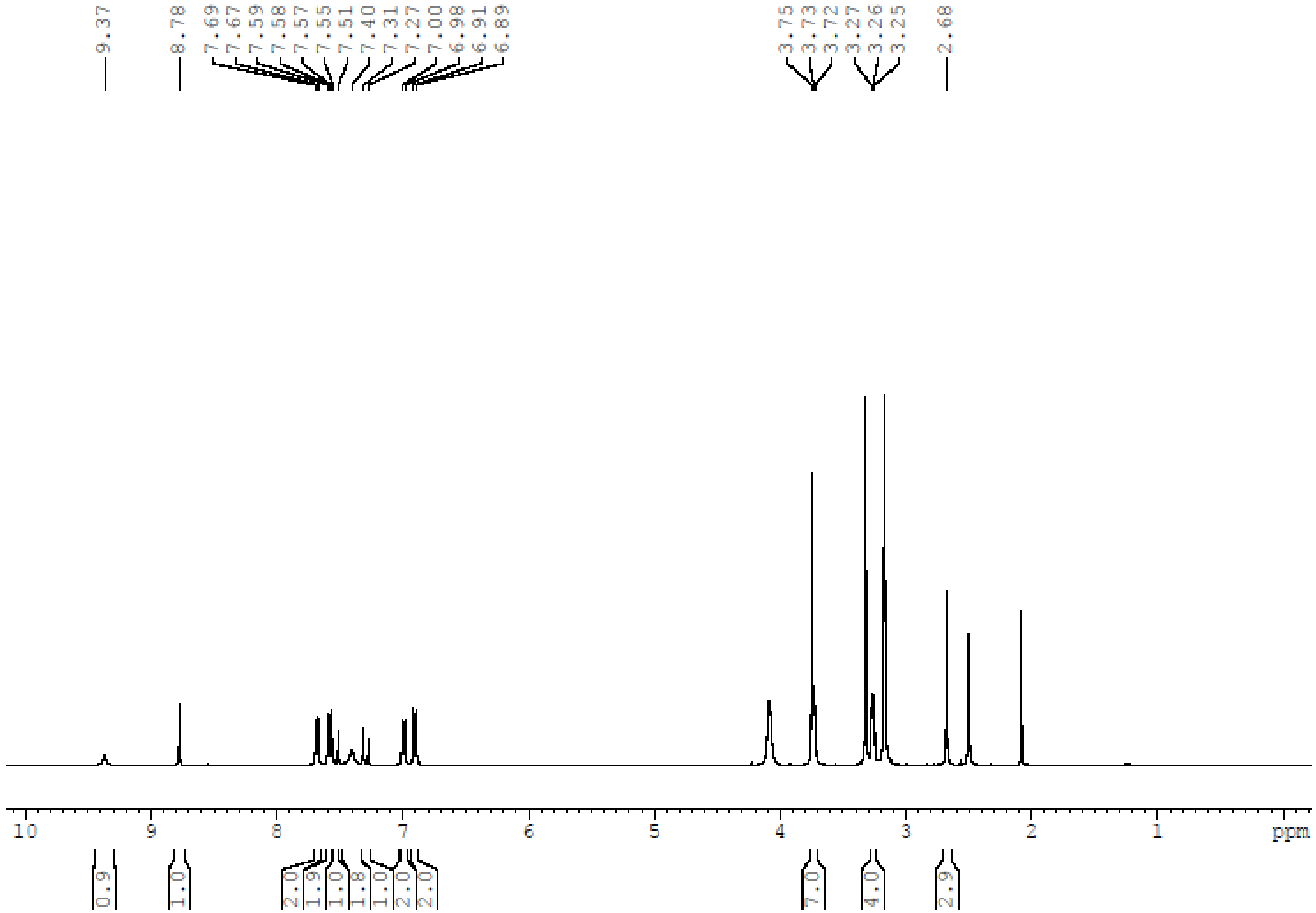

Figure S39: ^{1}H NMR spectrum of **12e** (400 MHz; DMSO-d_6).

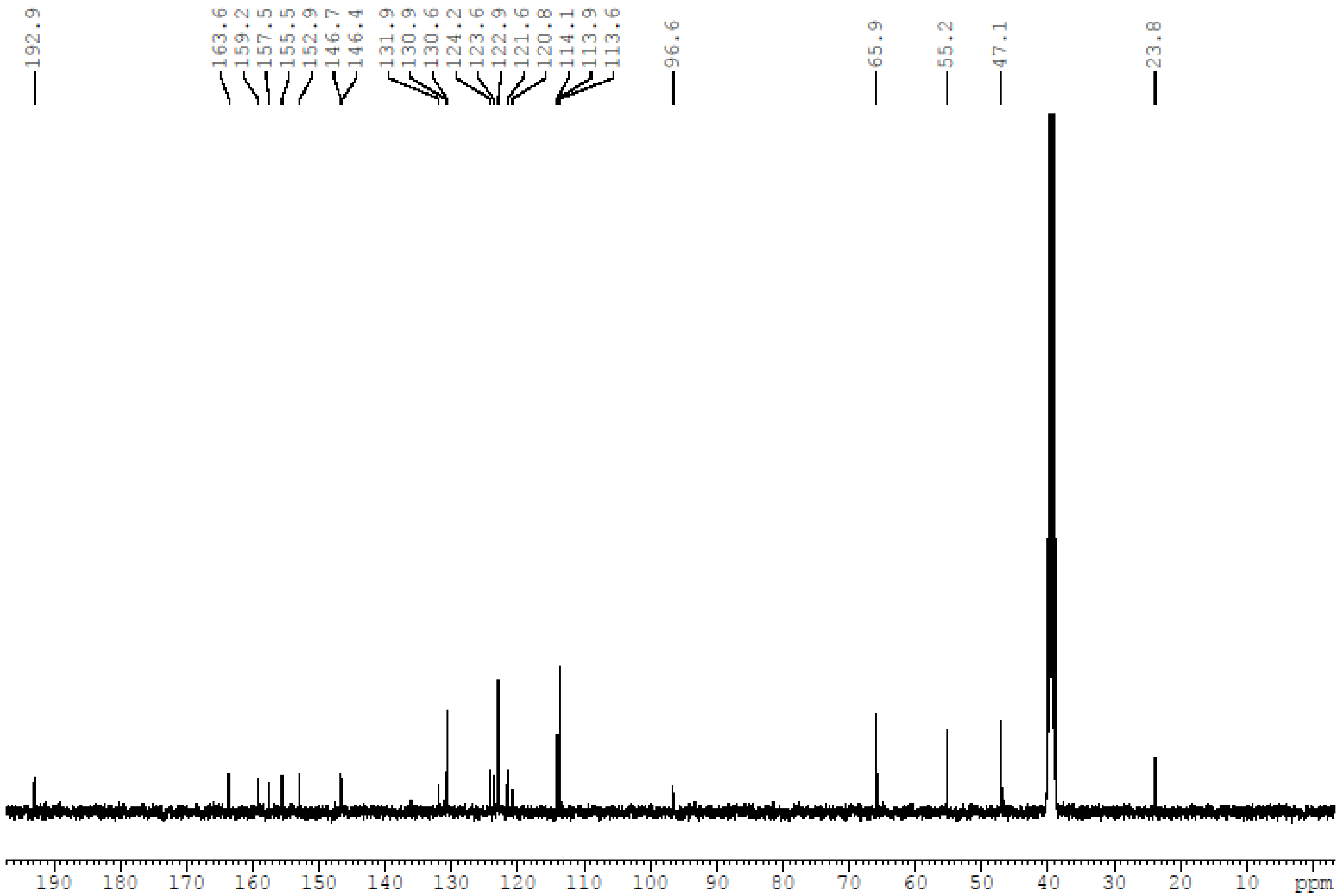

Figure S40: ^{13}C NMR spectrum of **12e** (100 MHz; DMSO-d_6).

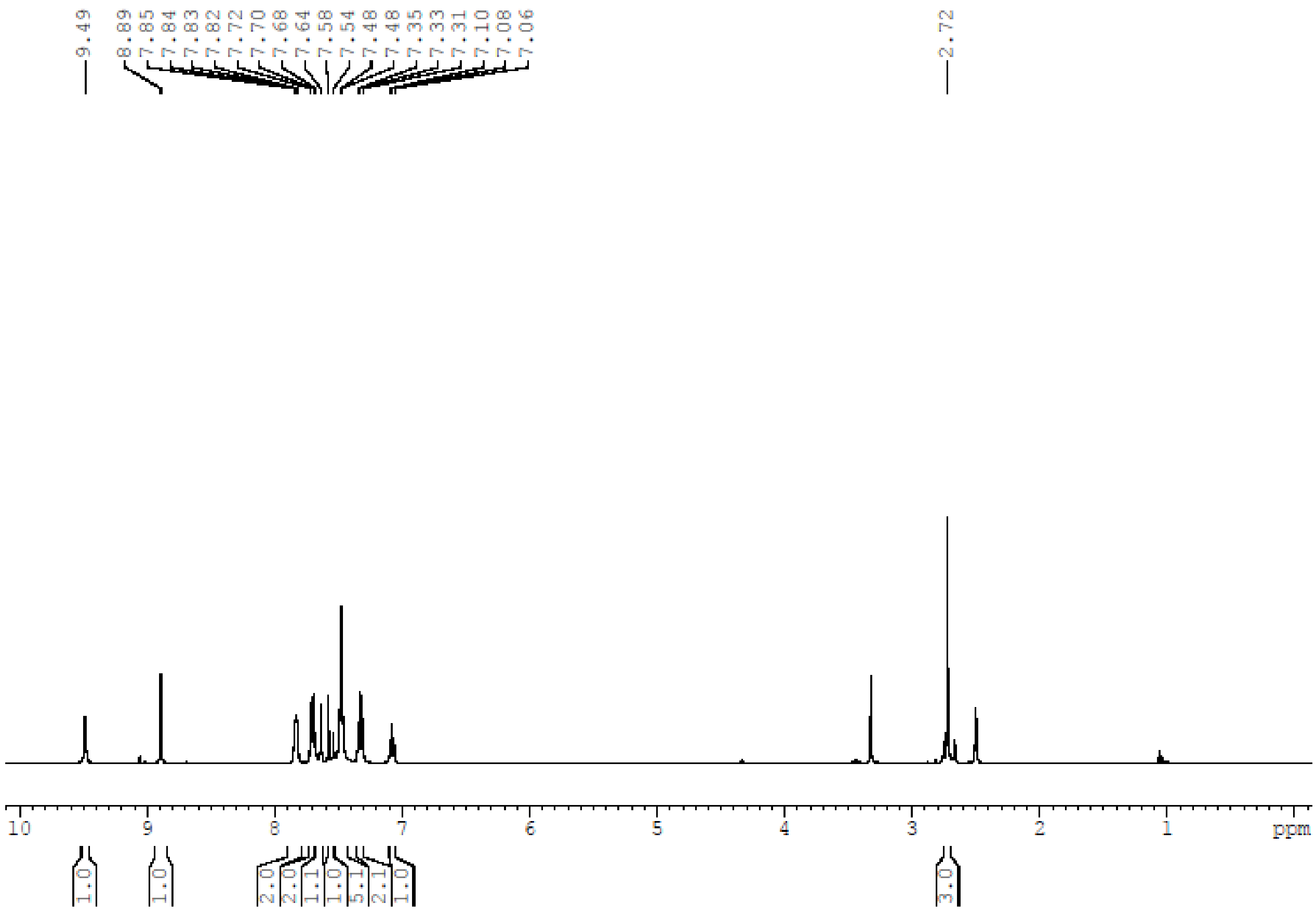

Figure S41: ^{1}H NMR spectrum of **13a** (400 MHz; DMSO-d_6).

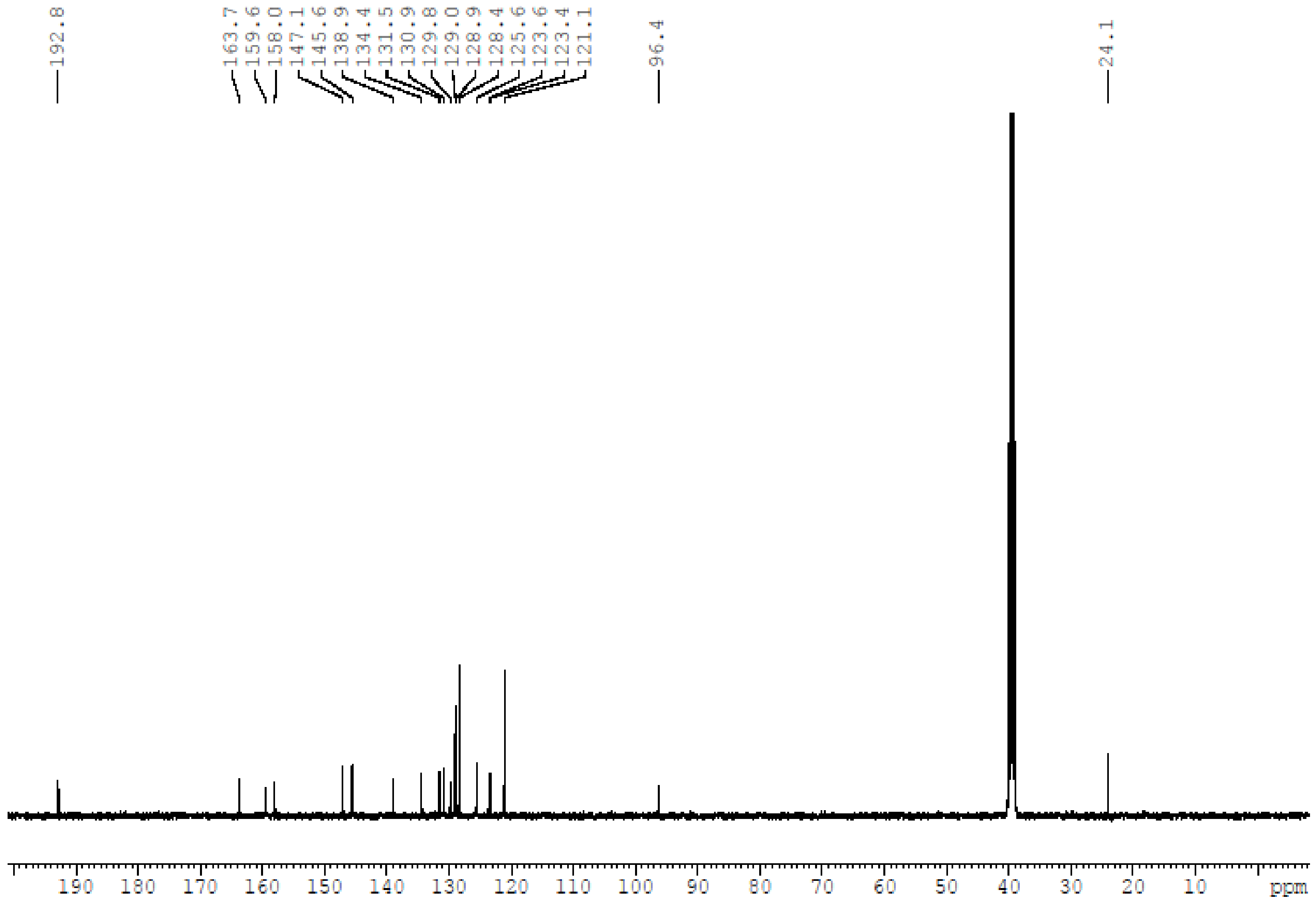

Figure S42: ^{13}C NMR spectrum of **13a** (100 MHz; DMSO-d_6).

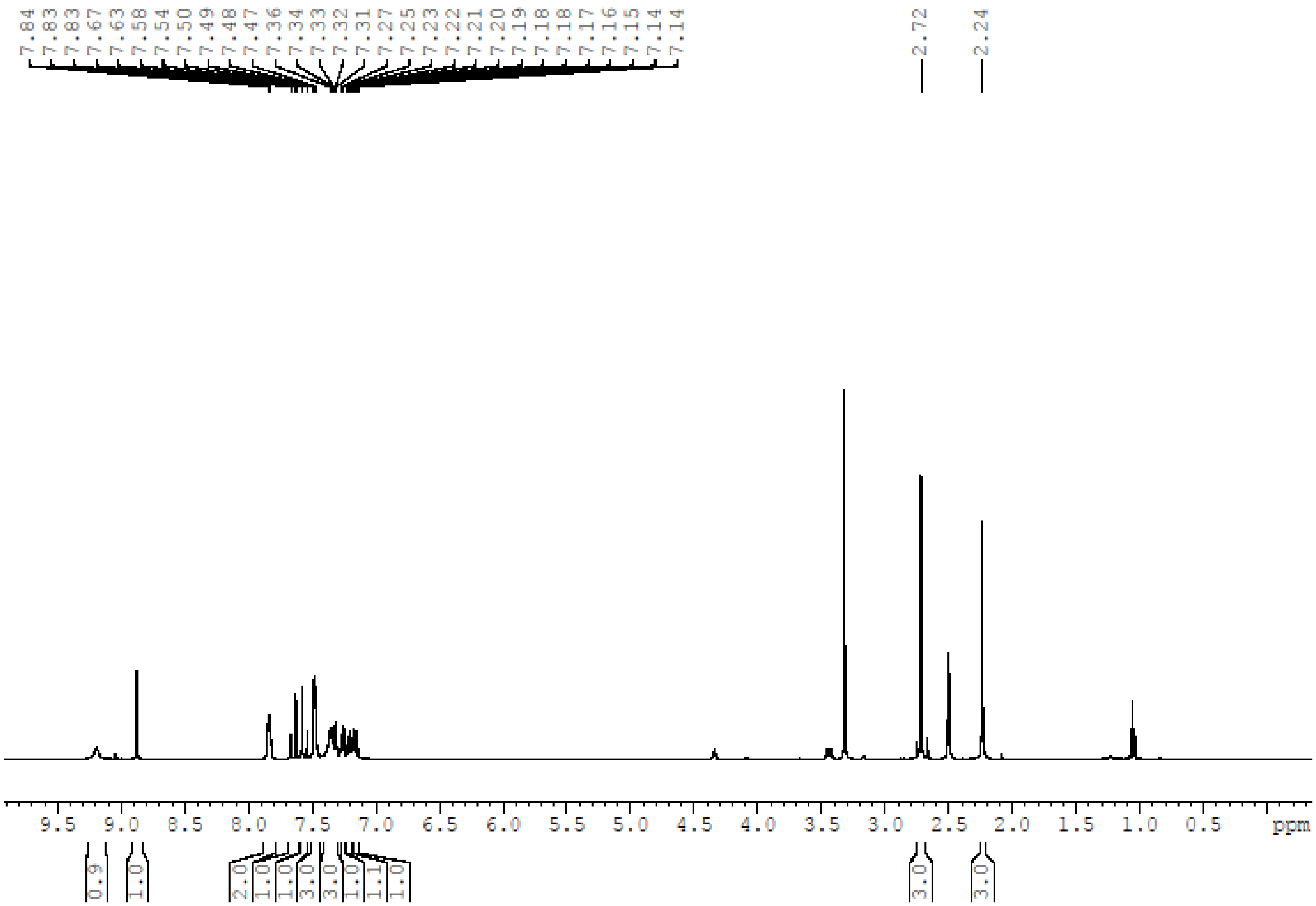

Figure S43: ^{1}H NMR spectrum of **13b** (400 MHz; DMSO-d_6).

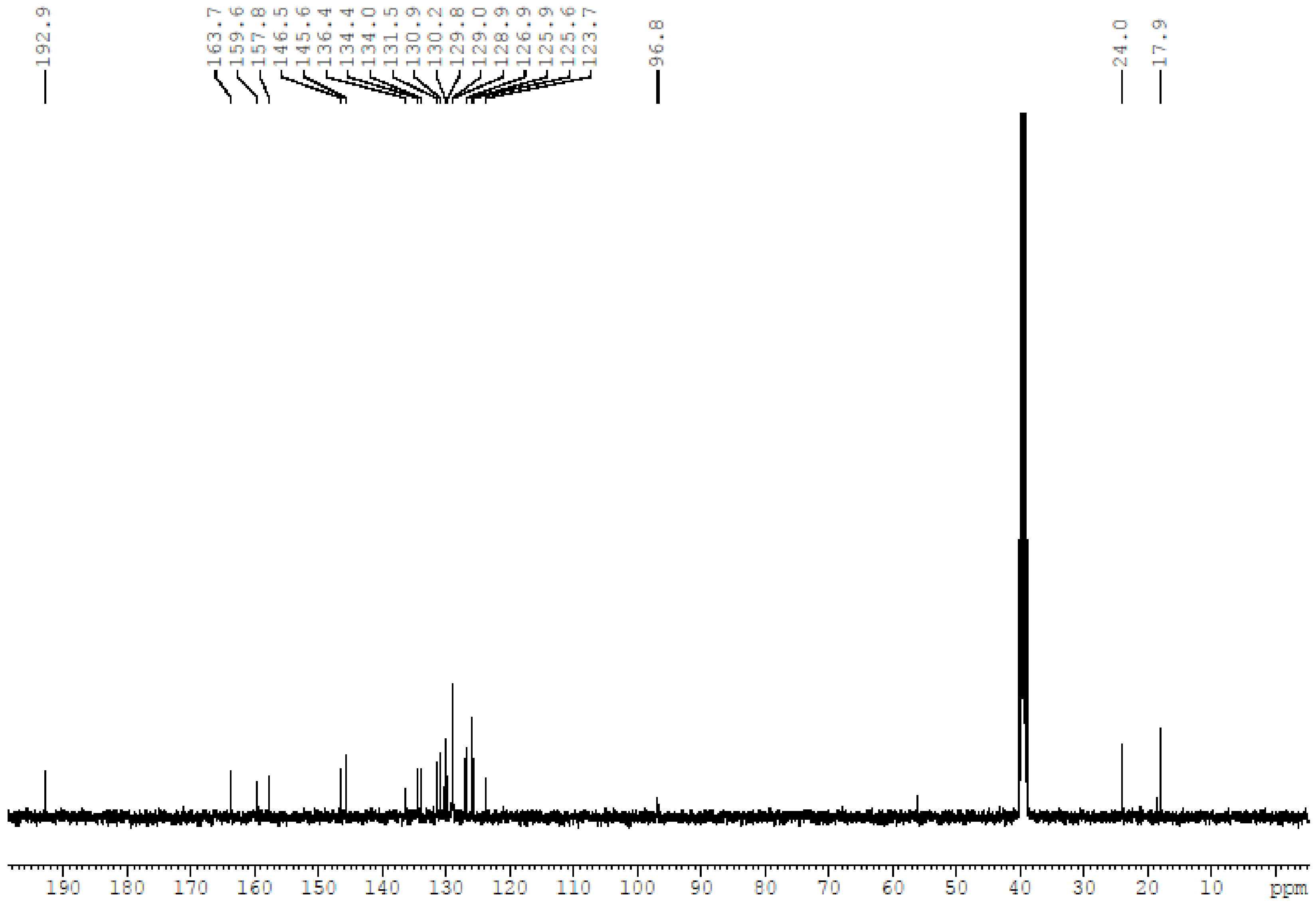

Figure S44: ^{13}C NMR spectrum of **13b** (100 MHz; DMSO-d_6).

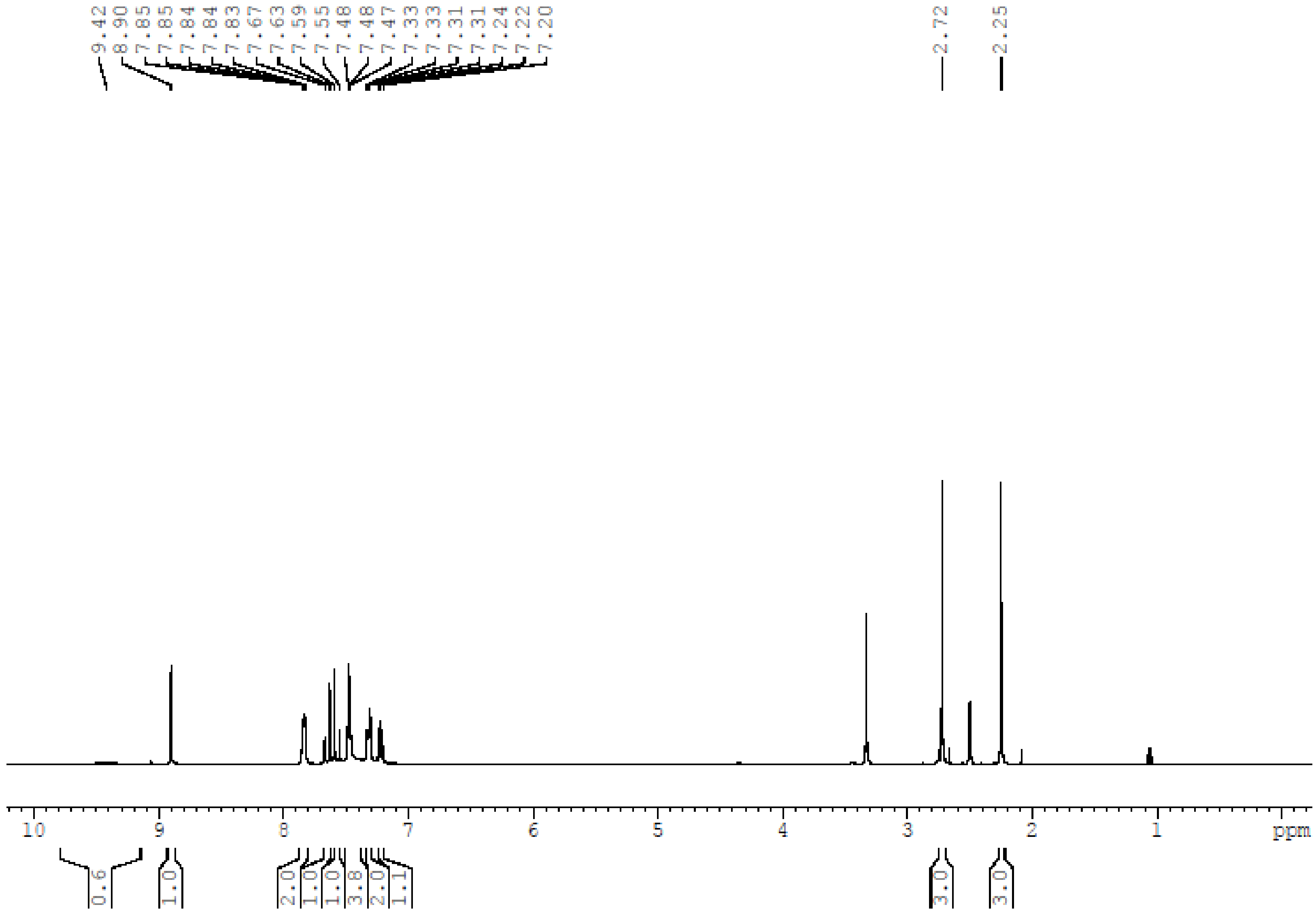

Figure S45: ^{1}H NMR spectrum of **13c** (400 MHz; DMSO-d_6).

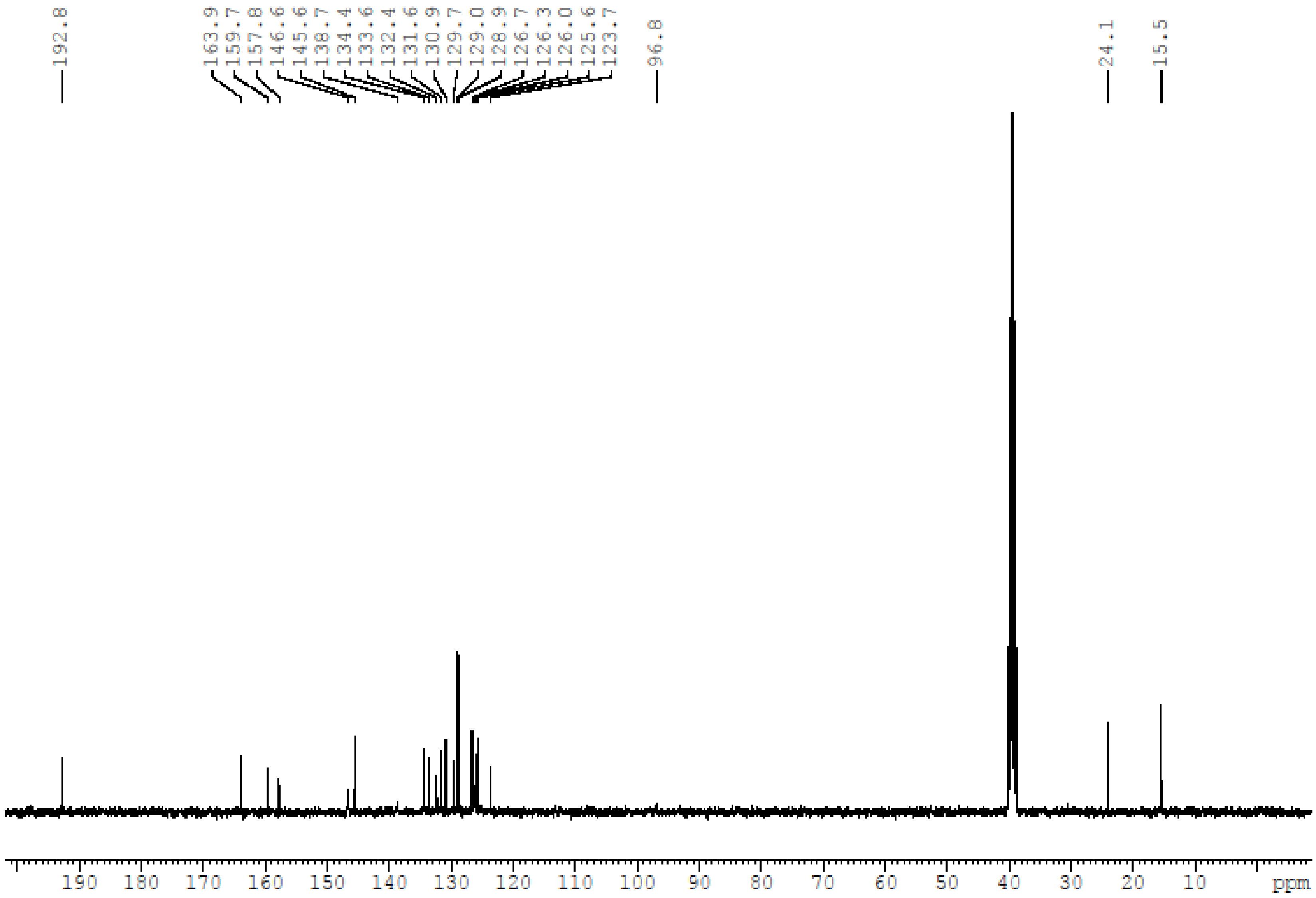

Figure S46: ^{13}C NMR spectrum of **13c** (100 MHz; DMSO-d_6).

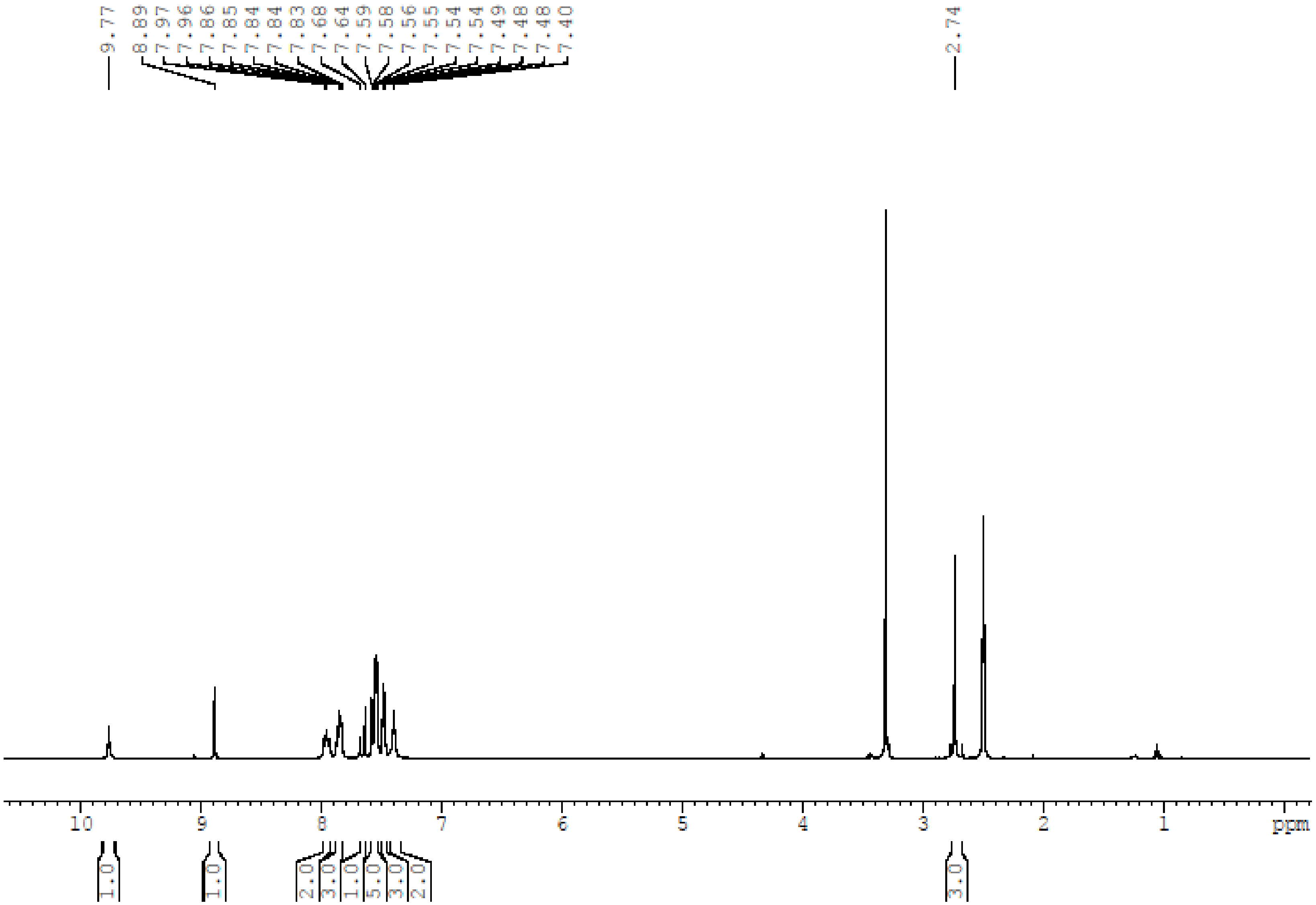

Figure S47: ^{1}H NMR spectrum of **13d** (400 MHz; DMSO-d_6).

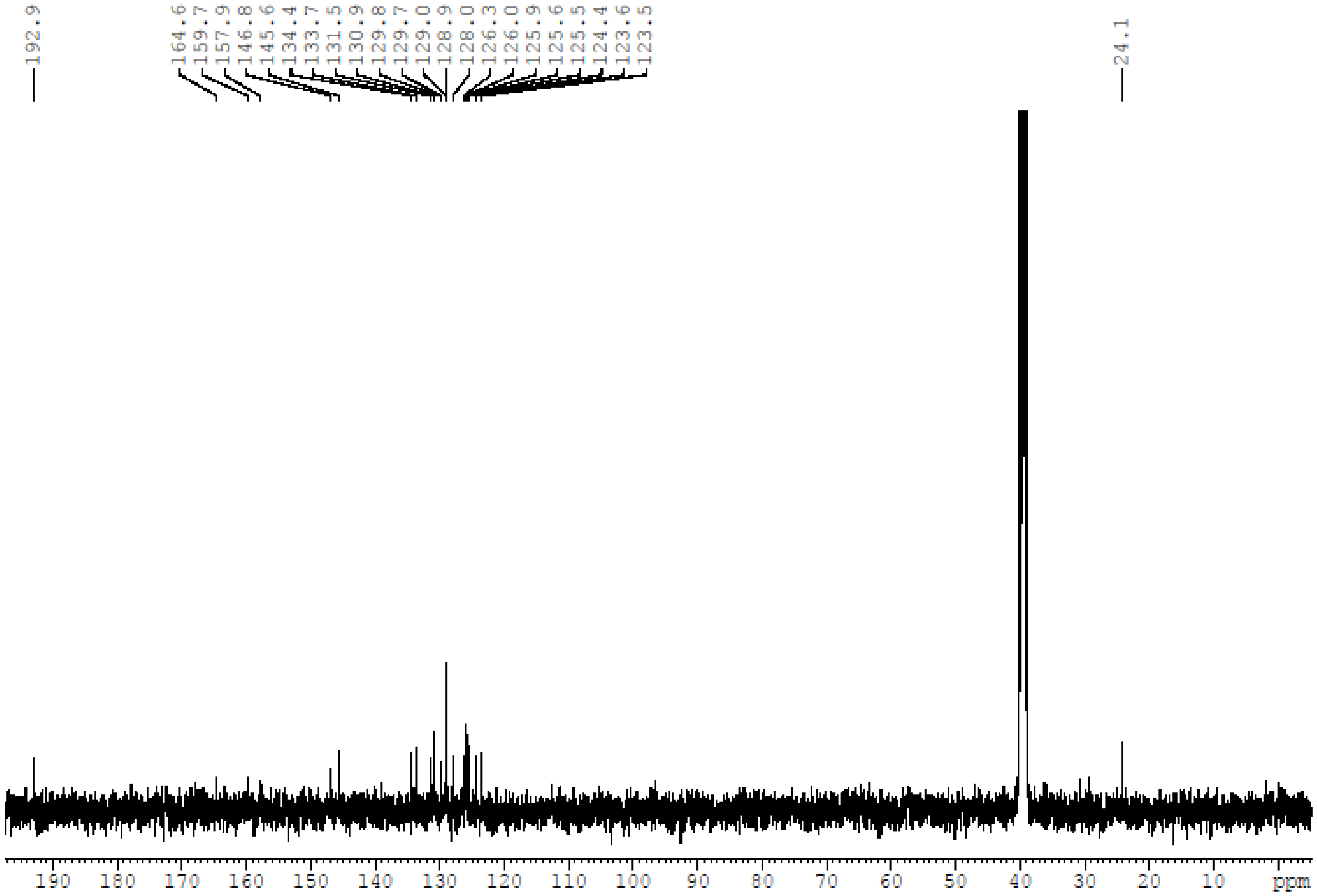

Figure S48: ^{13}C NMR spectrum of **13d** (100 MHz; DMSO-d_6).

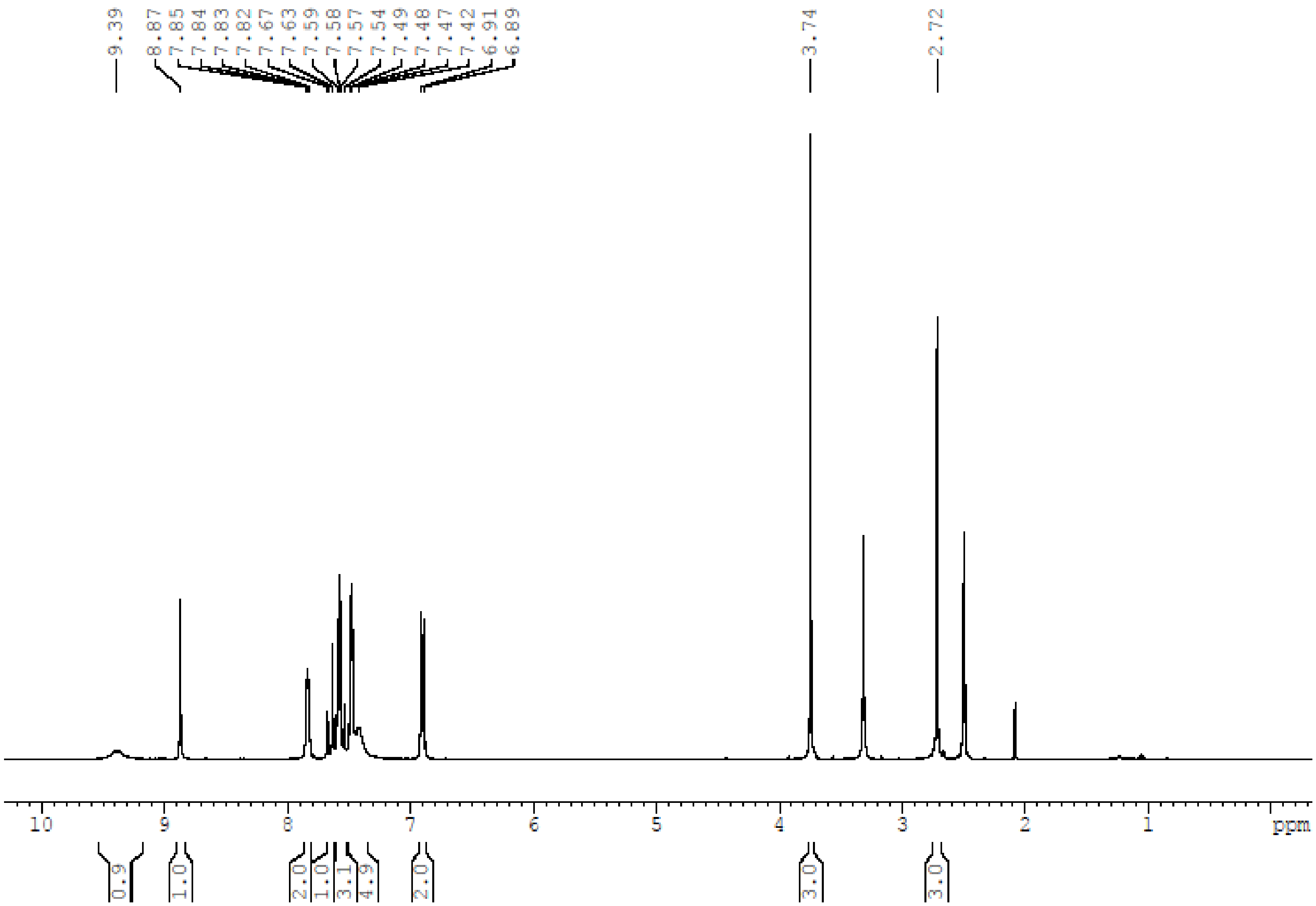

Figure S49: ^{1}H NMR spectrum of **13e** (400 MHz; DMSO-d_6).

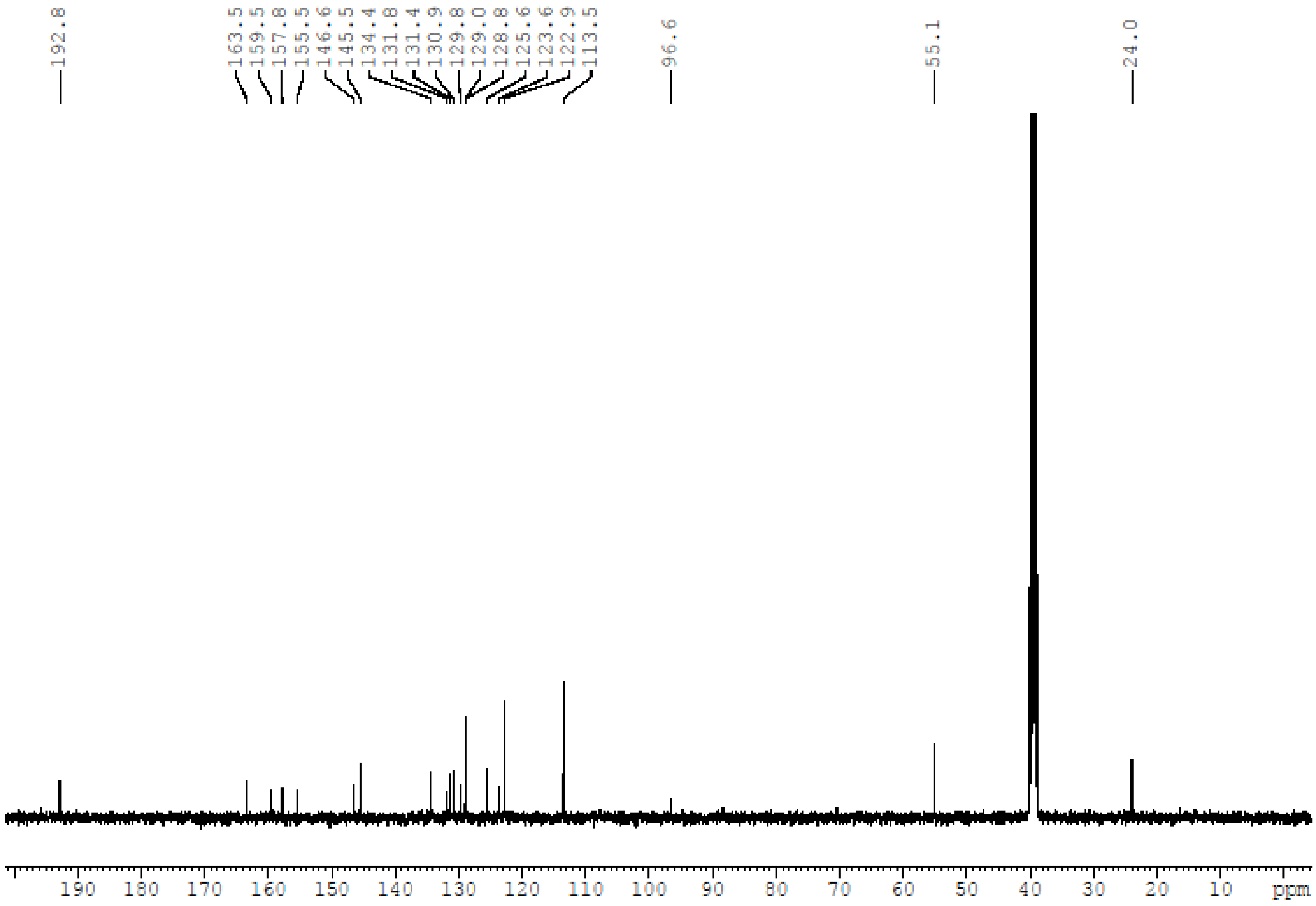

Figure S50: ^{13}C NMR spectrum of **13e** (100 MHz; DMSO-d_6).

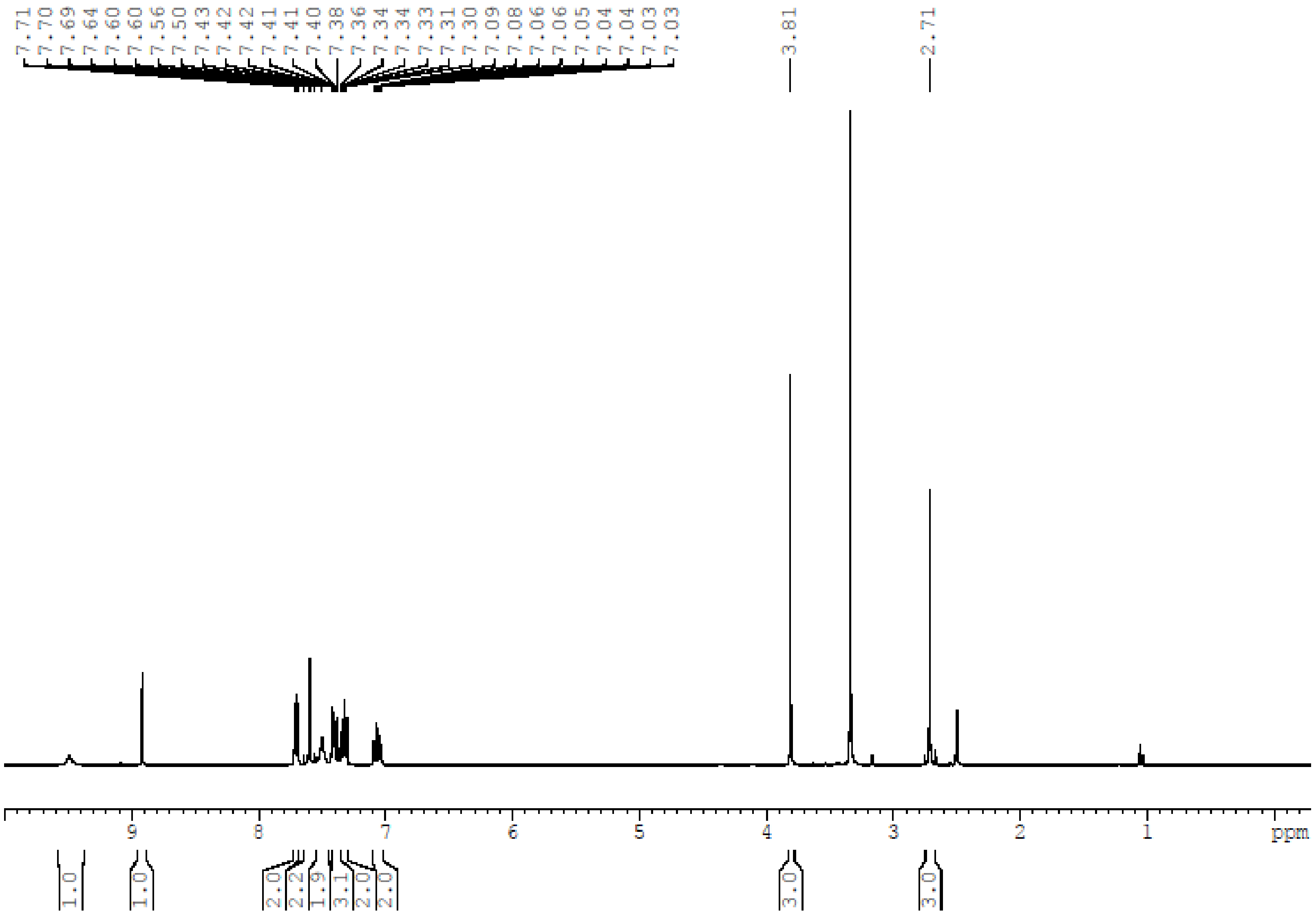

Figure S51: ^{1}H NMR spectrum of **14a** (400 MHz; DMSO-d_6).

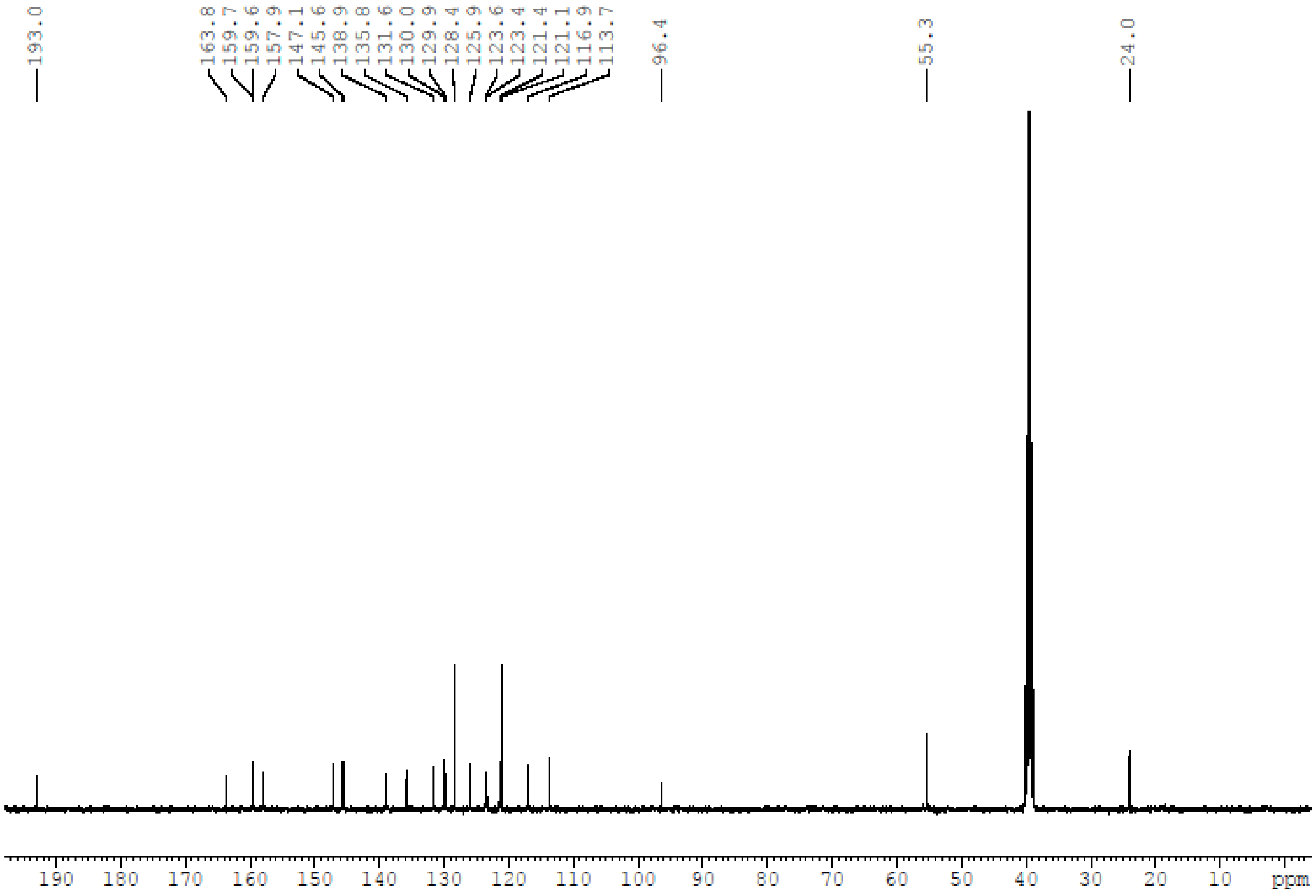

Figure S52: ^{13}C NMR spectrum of **14a** (100 MHz; DMSO-d_6).

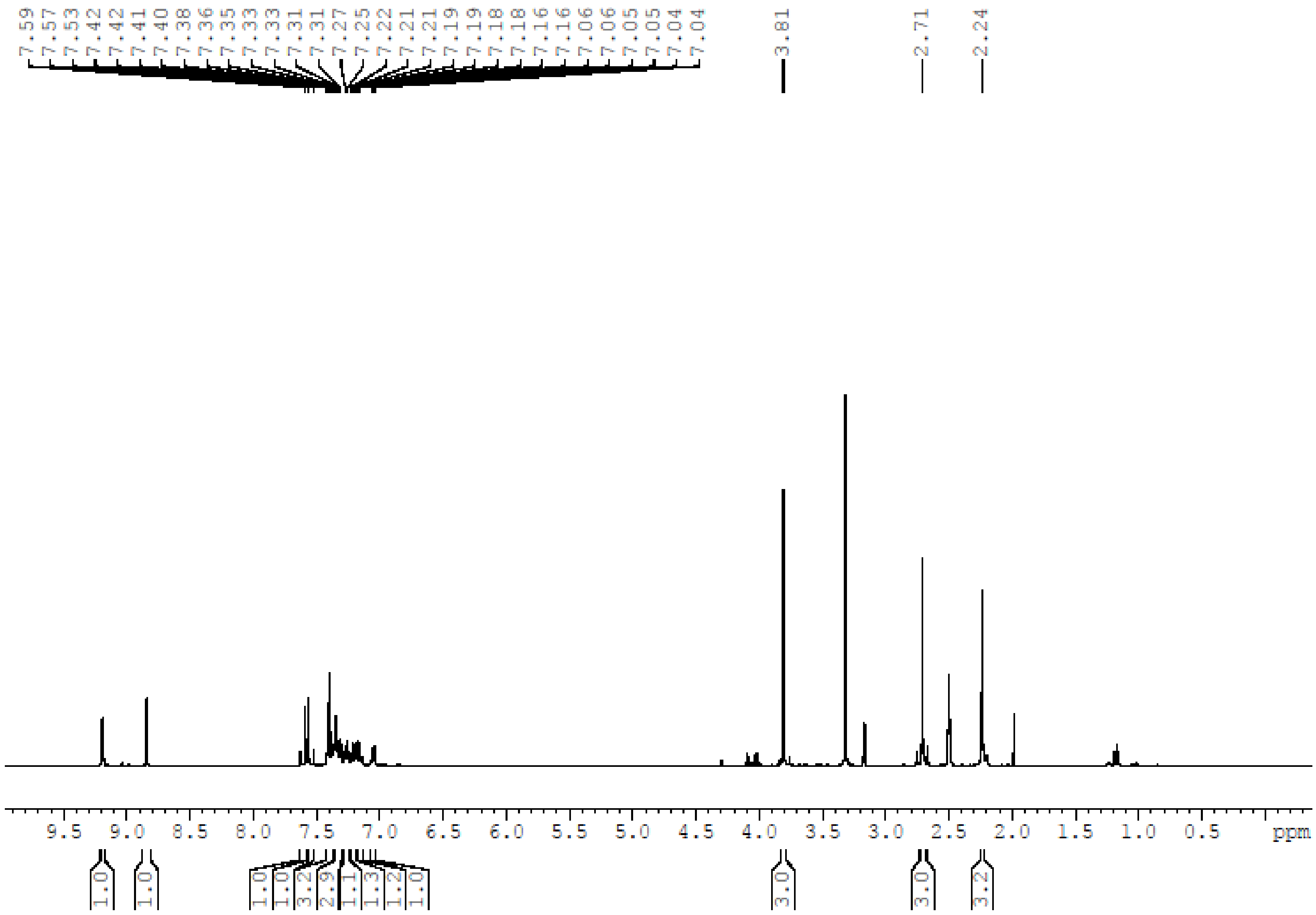

Figure S53: ^{1}H NMR spectrum of **14b** (400 MHz; DMSO-d_6).

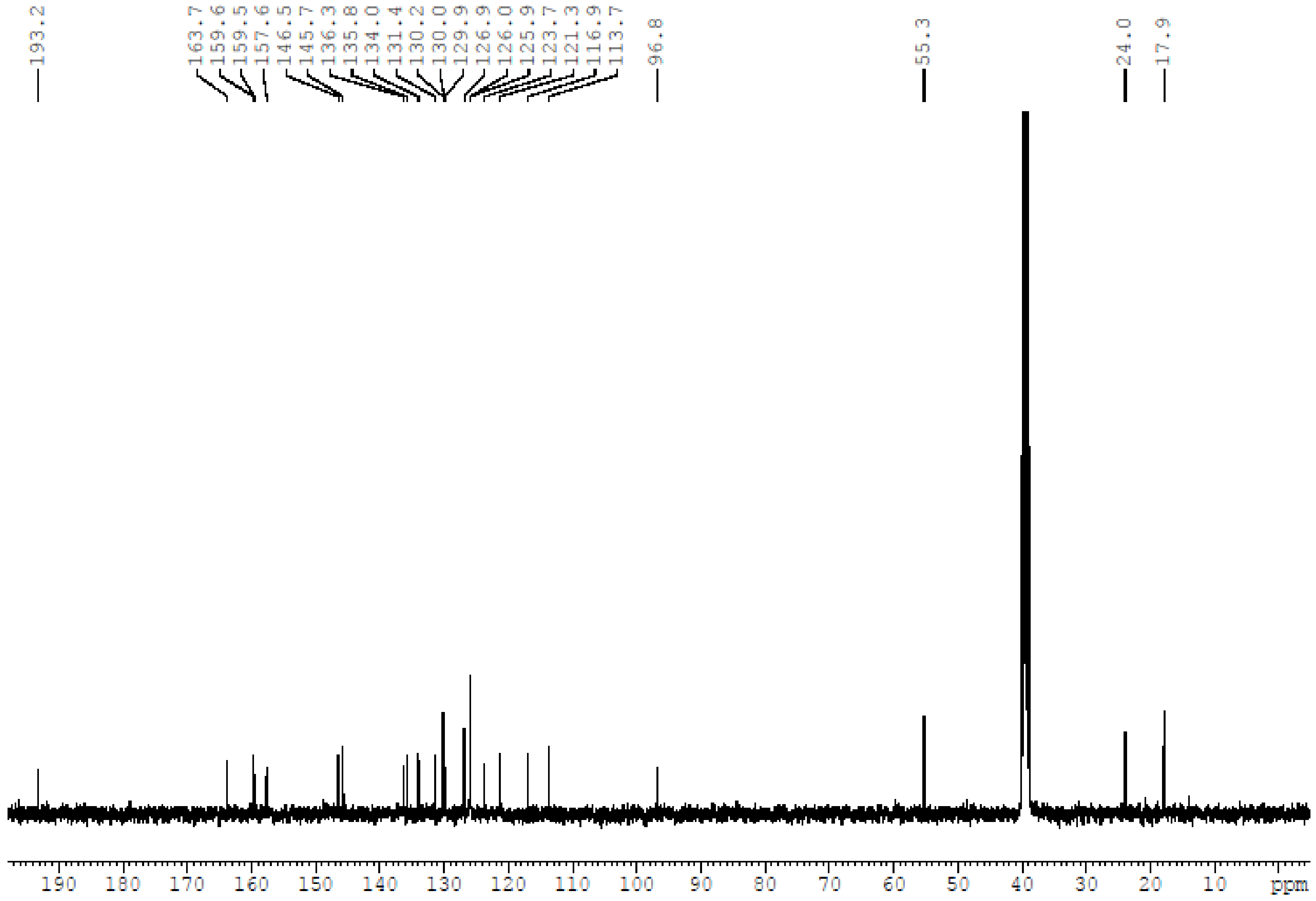

Figure S54: ^{13}C NMR spectrum of **14b** (100 MHz; DMSO-d_6).

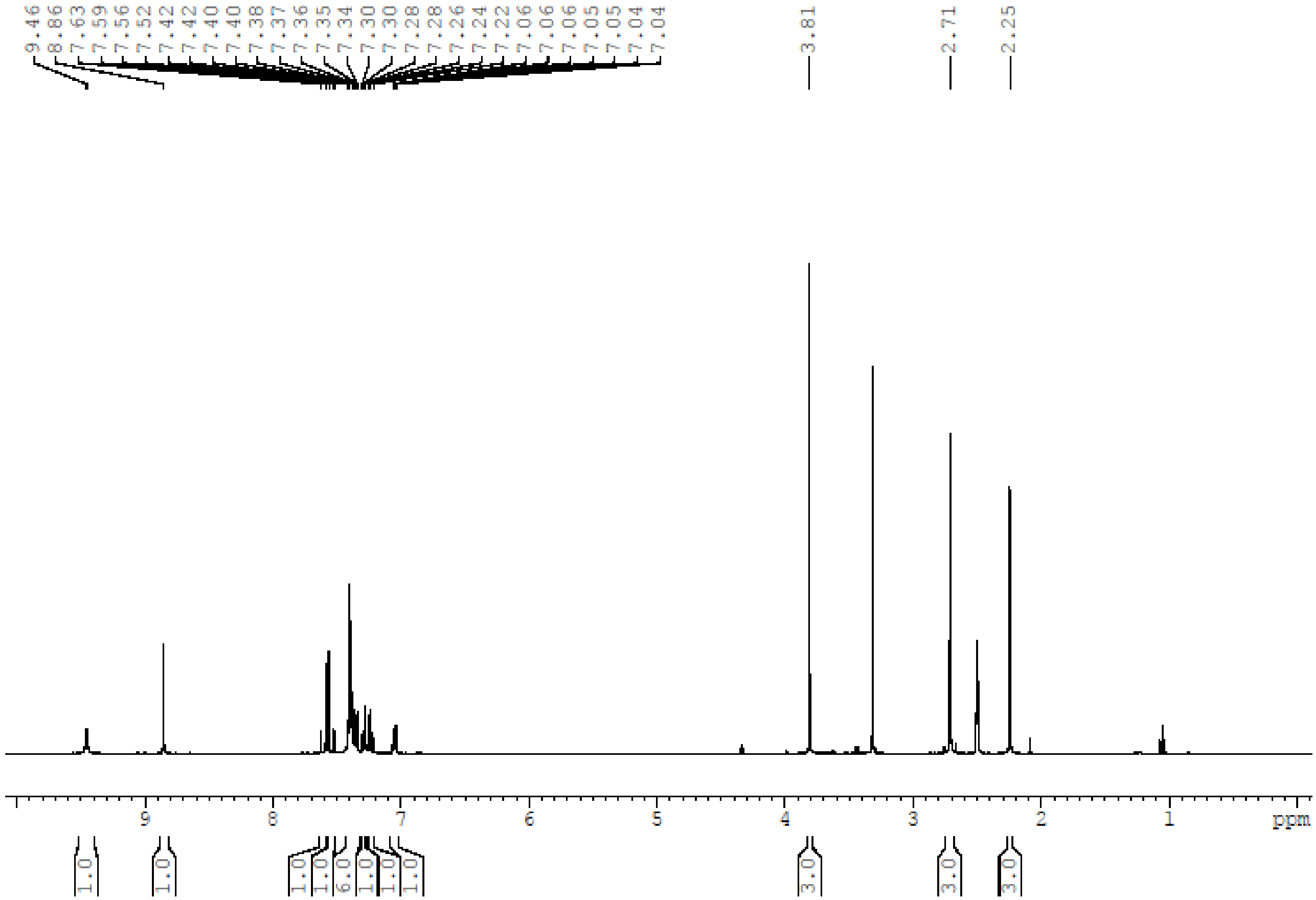

Figure S55: ^{1}H NMR spectrum of **14c** (400 MHz; DMSO-d_6).

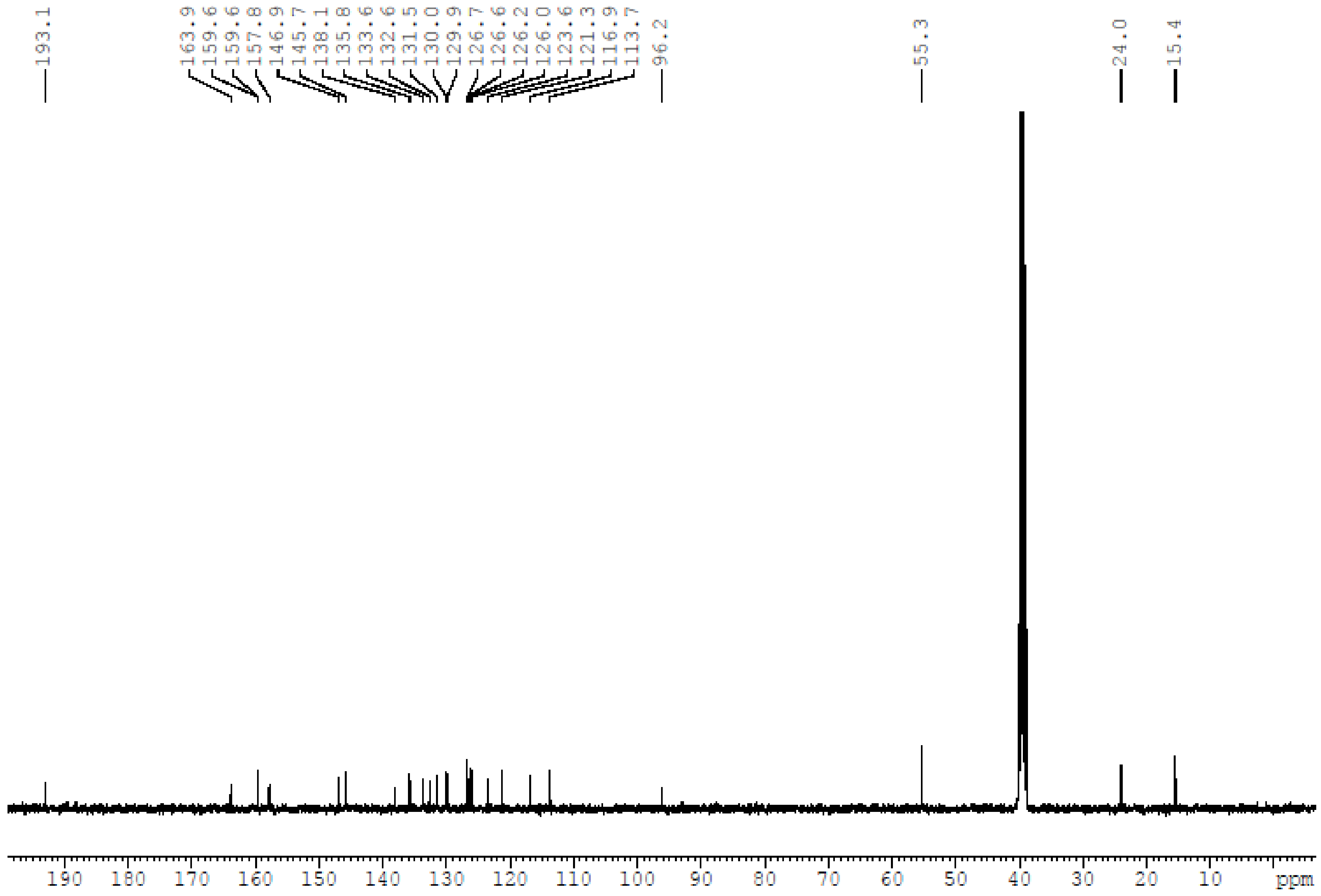

Figure S56: ^{13}C NMR spectrum of **14c** (100 MHz; DMSO-d_6).

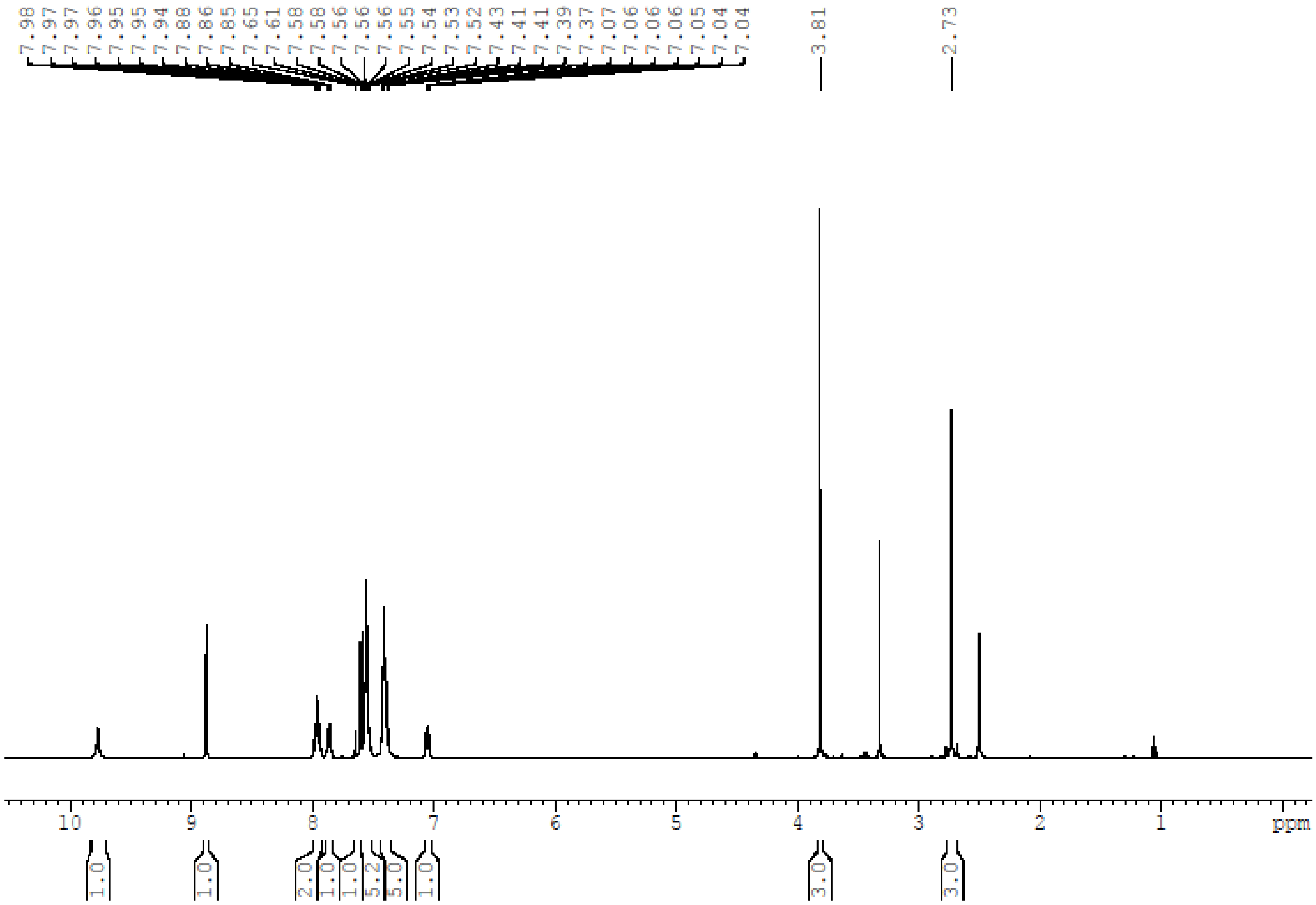

Figure S57: ^{1}H NMR spectrum of **14d** (400 MHz; DMSO-d_6).

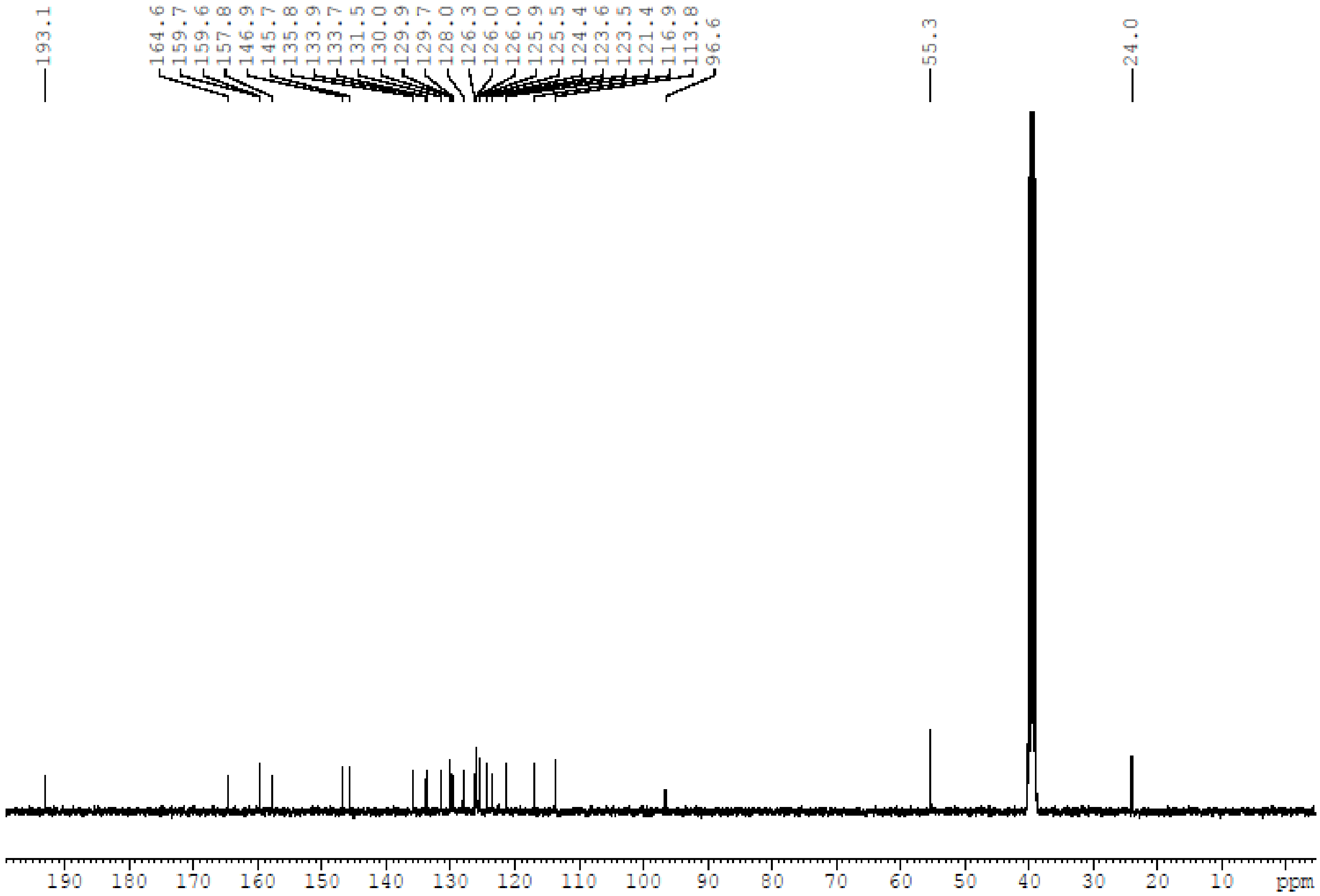

Figure S58: ^{13}C NMR spectrum of **14d** (100 MHz; DMSO-d_6).

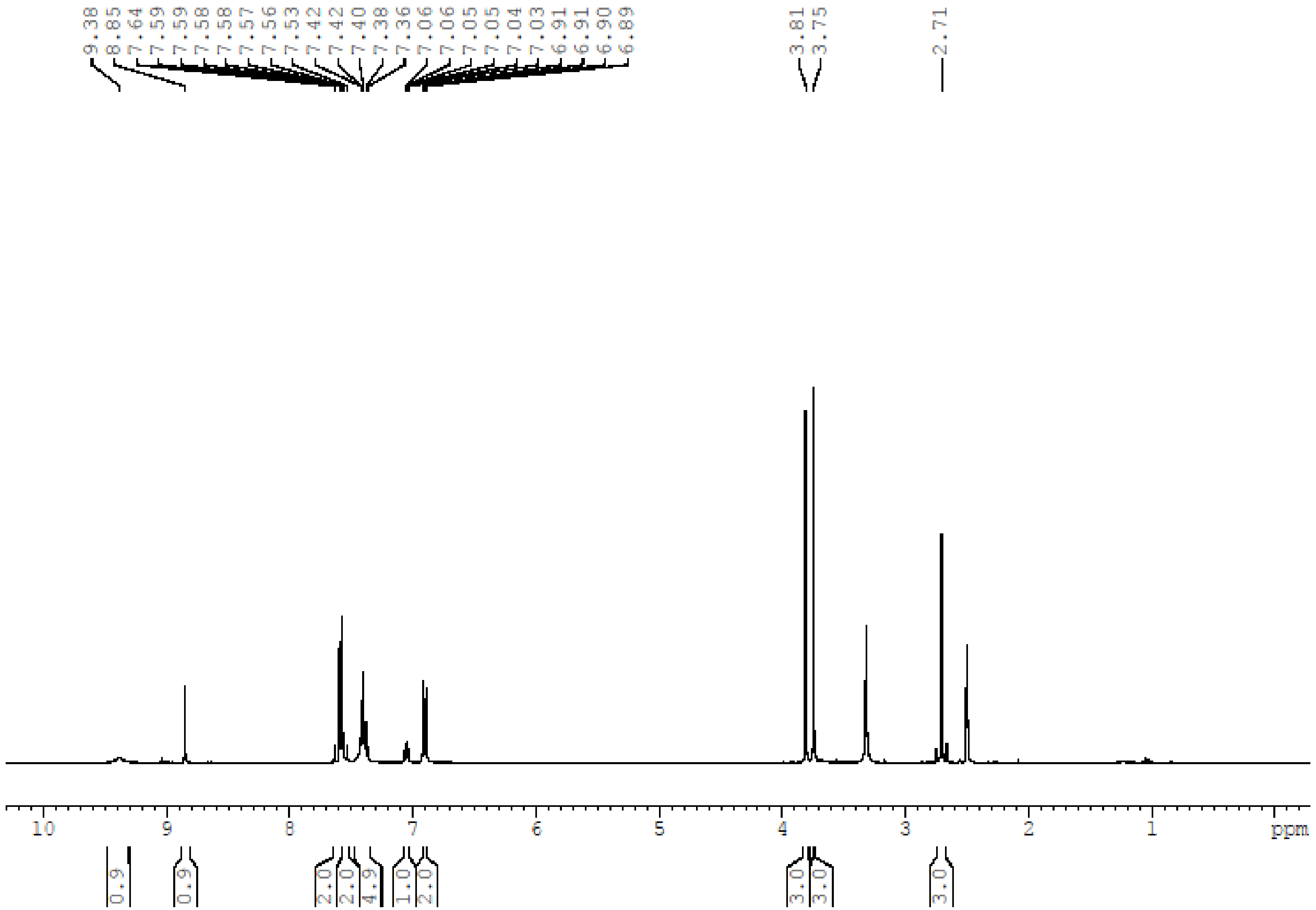

Figure S59: ^{1}H NMR spectrum of **14e** (400 MHz; DMSO-d_6).

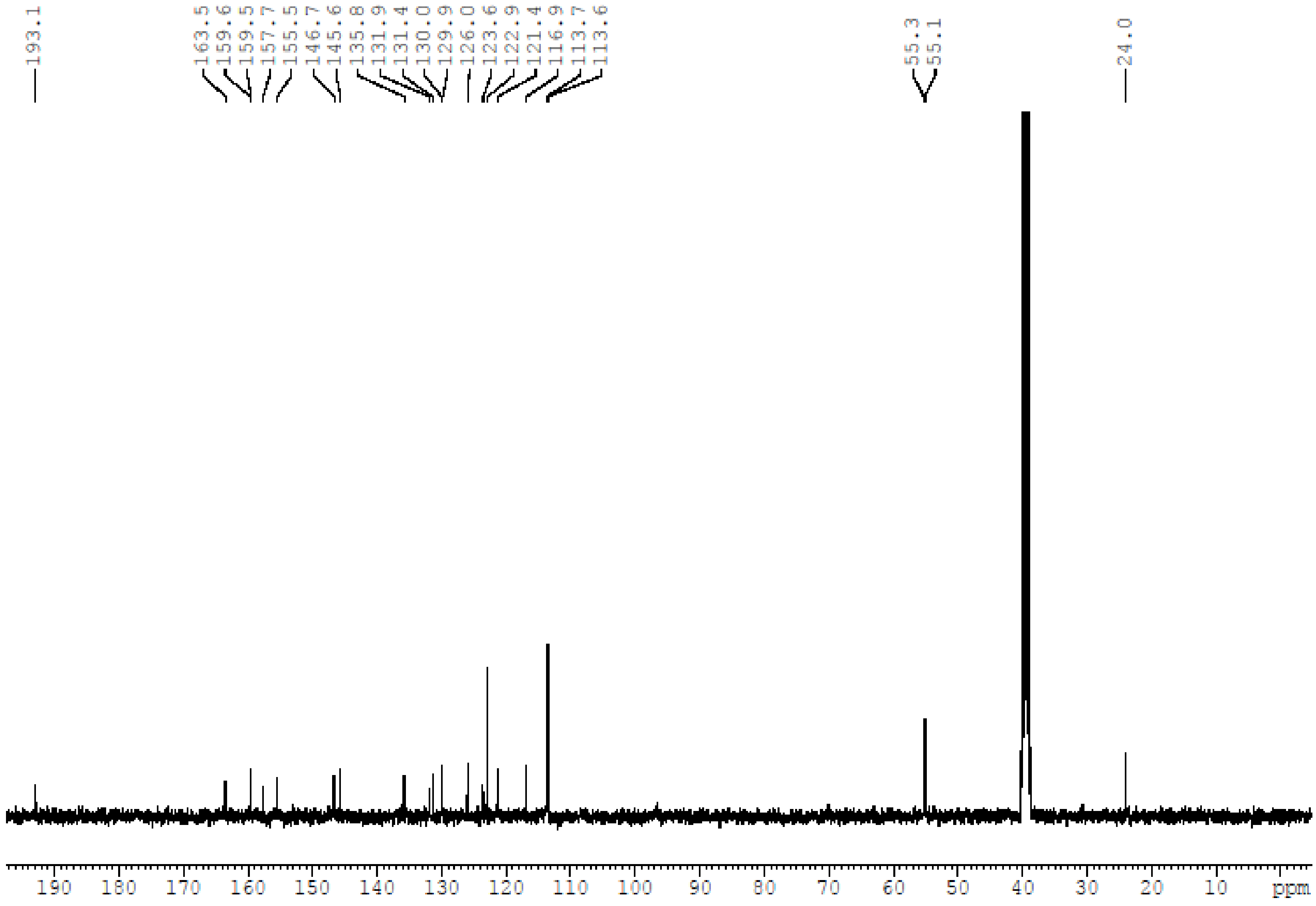

Figure S60: ^{13}C NMR spectrum of **14e** (100 MHz; DMSO-d_6).

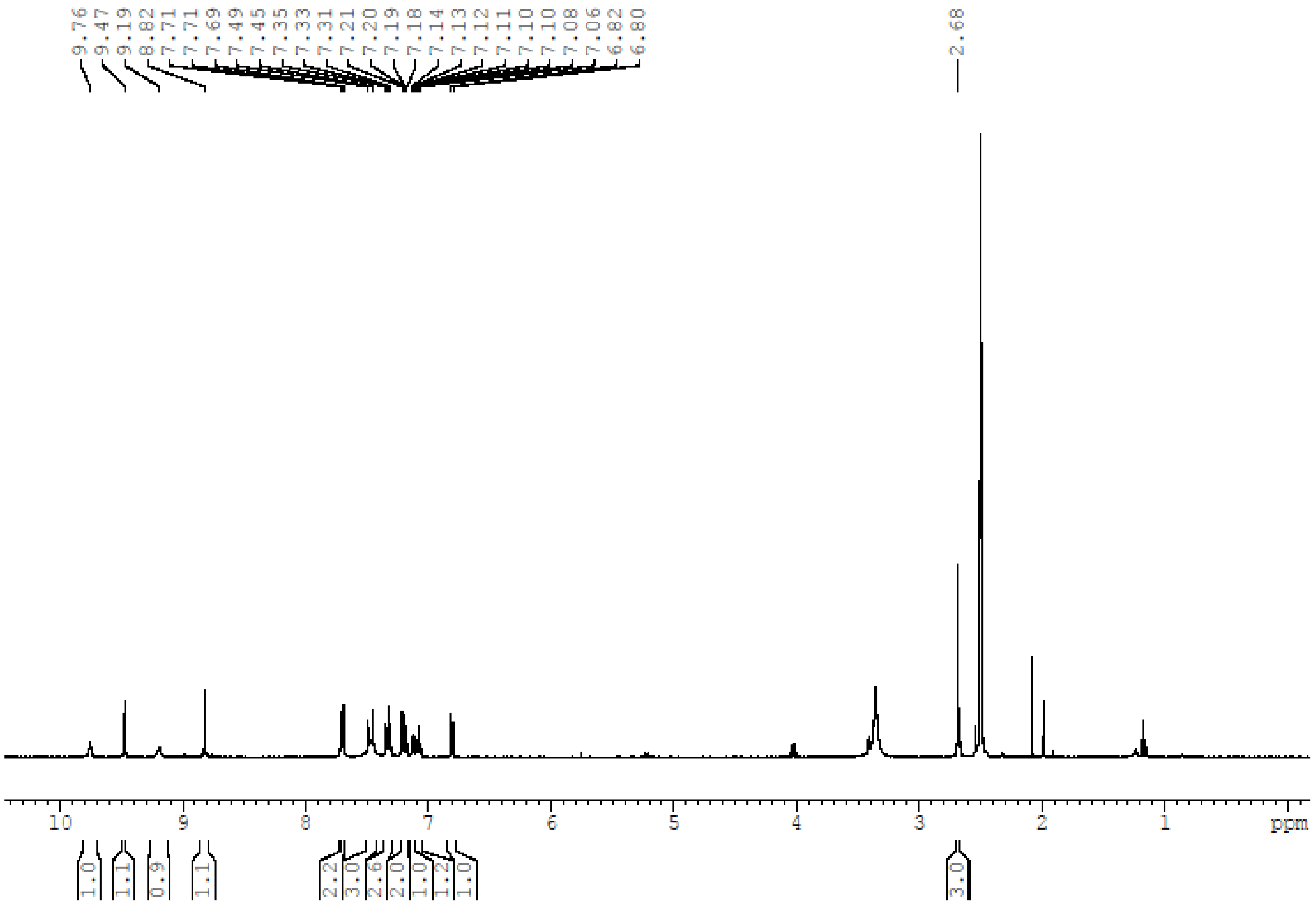

Figure S61: ^{1}H NMR spectrum of **15a** (400 MHz; DMSO-*d*$_6$).

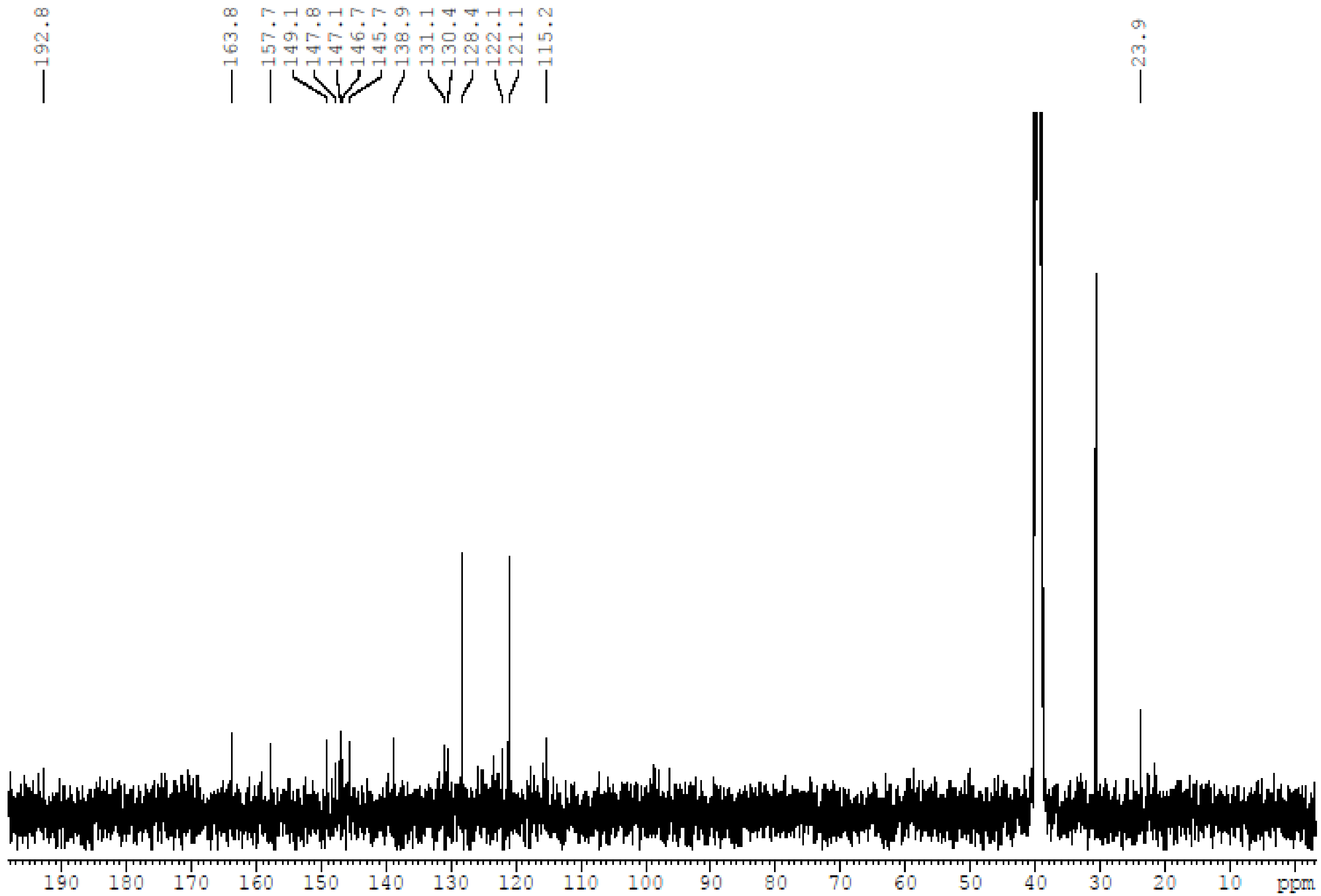

Figure S62: ^{13}C NMR spectrum of **15a** (100 MHz; DMSO-d_6).

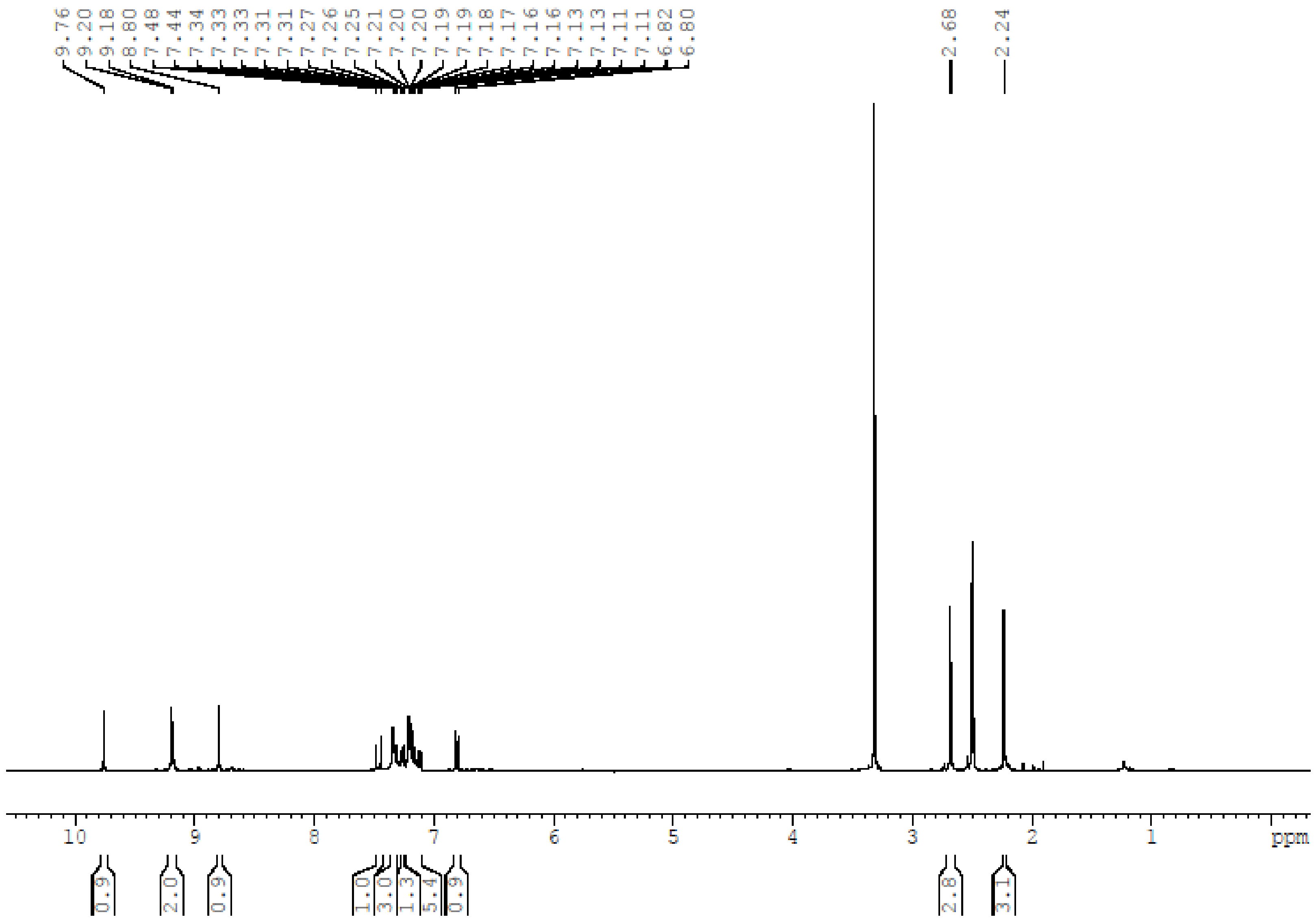

Figure S63: ^{1}H NMR spectrum of **15b** (400 MHz; DMSO-d_6).

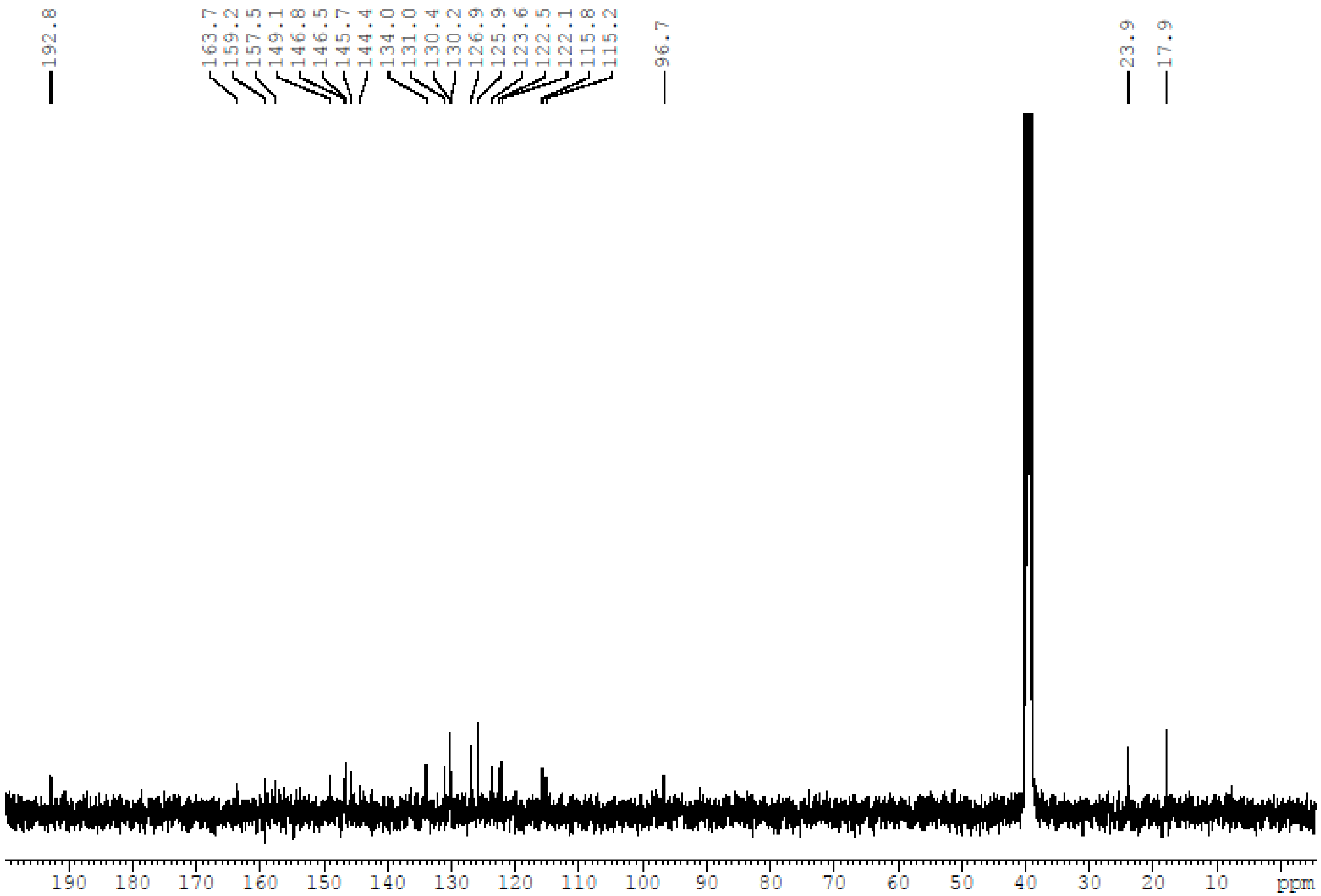

Figure S64: ^{13}C NMR spectrum of **15b** (100 MHz; DMSO-d_6).

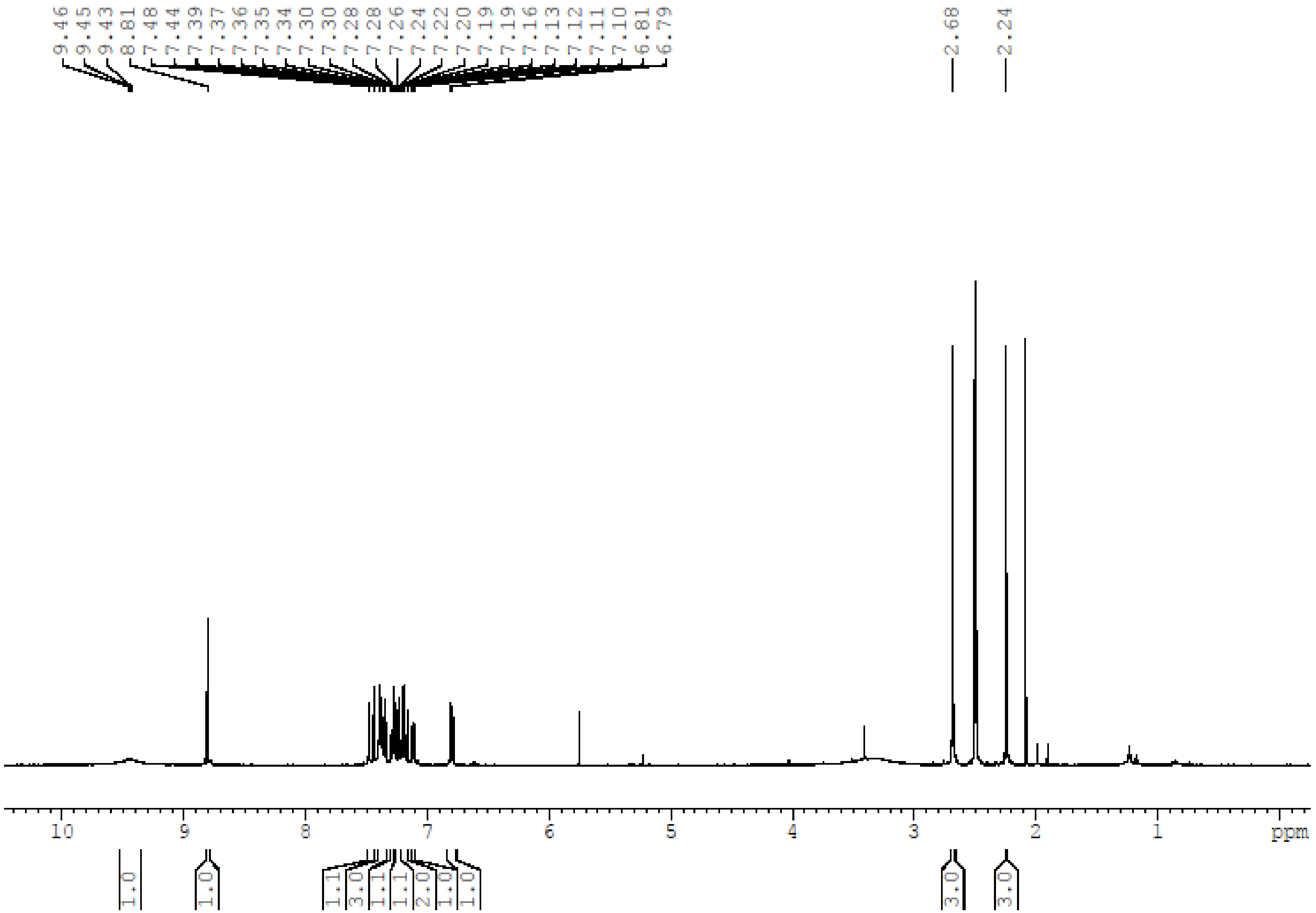

Figure S65: ^{1}H NMR spectrum of **15c** (400 MHz; DMSO-d_6).

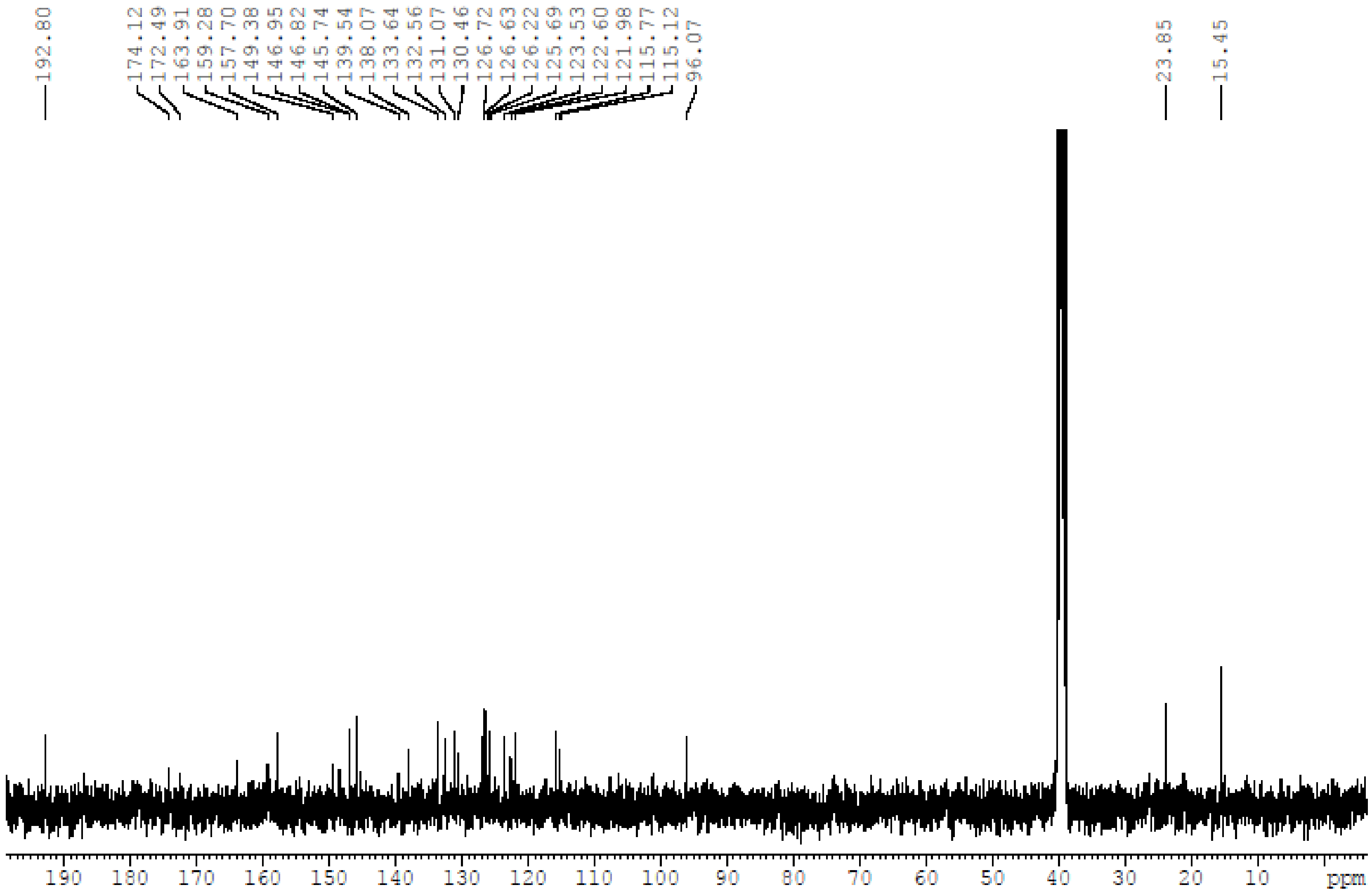

Figure S66: ^{13}C NMR spectrum of **15c** (100 MHz; DMSO-*d*$_6$).

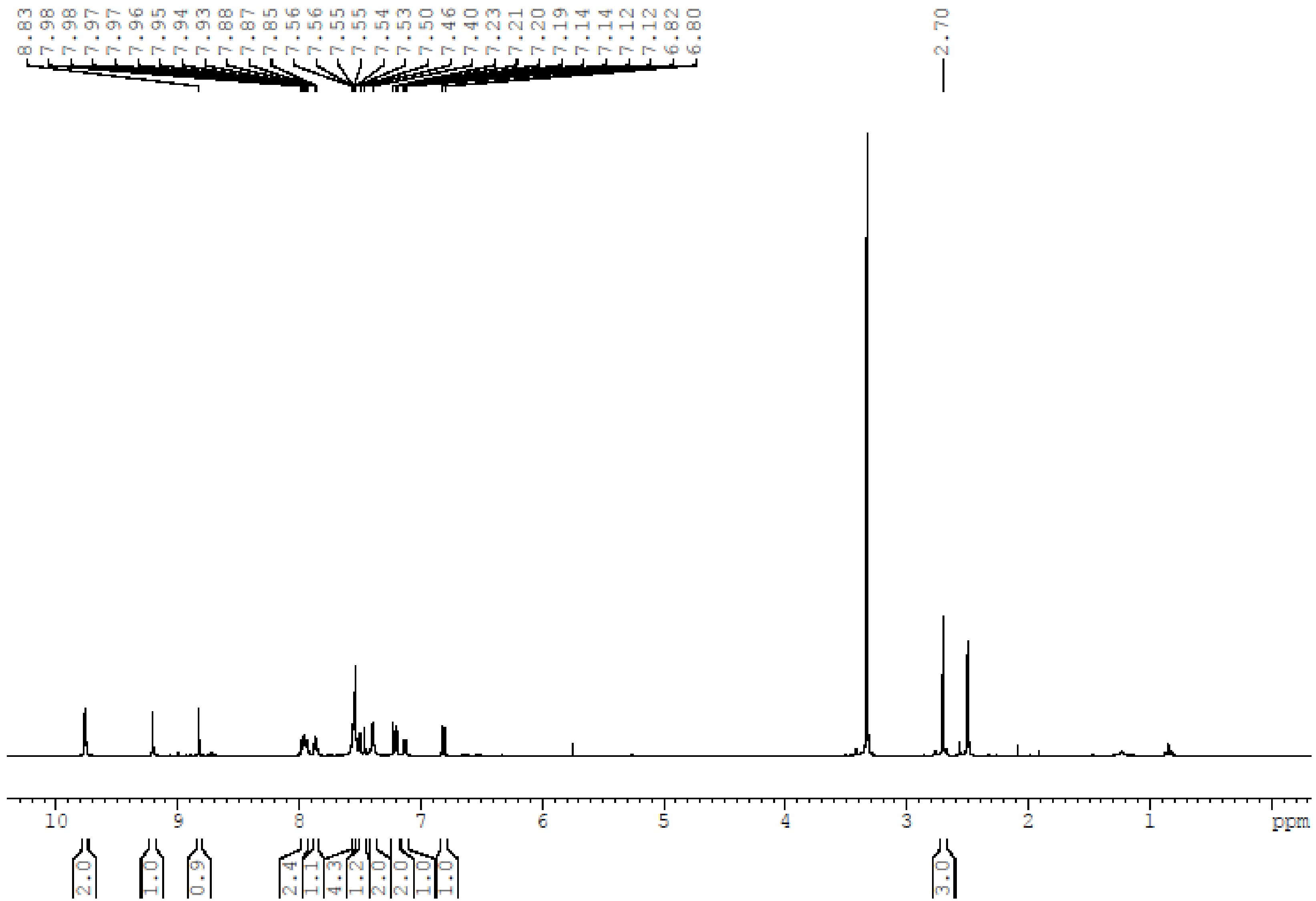

Figure S67: ^{1}H NMR spectrum of **15d** (400 MHz; DMSO-*d*$_6$).

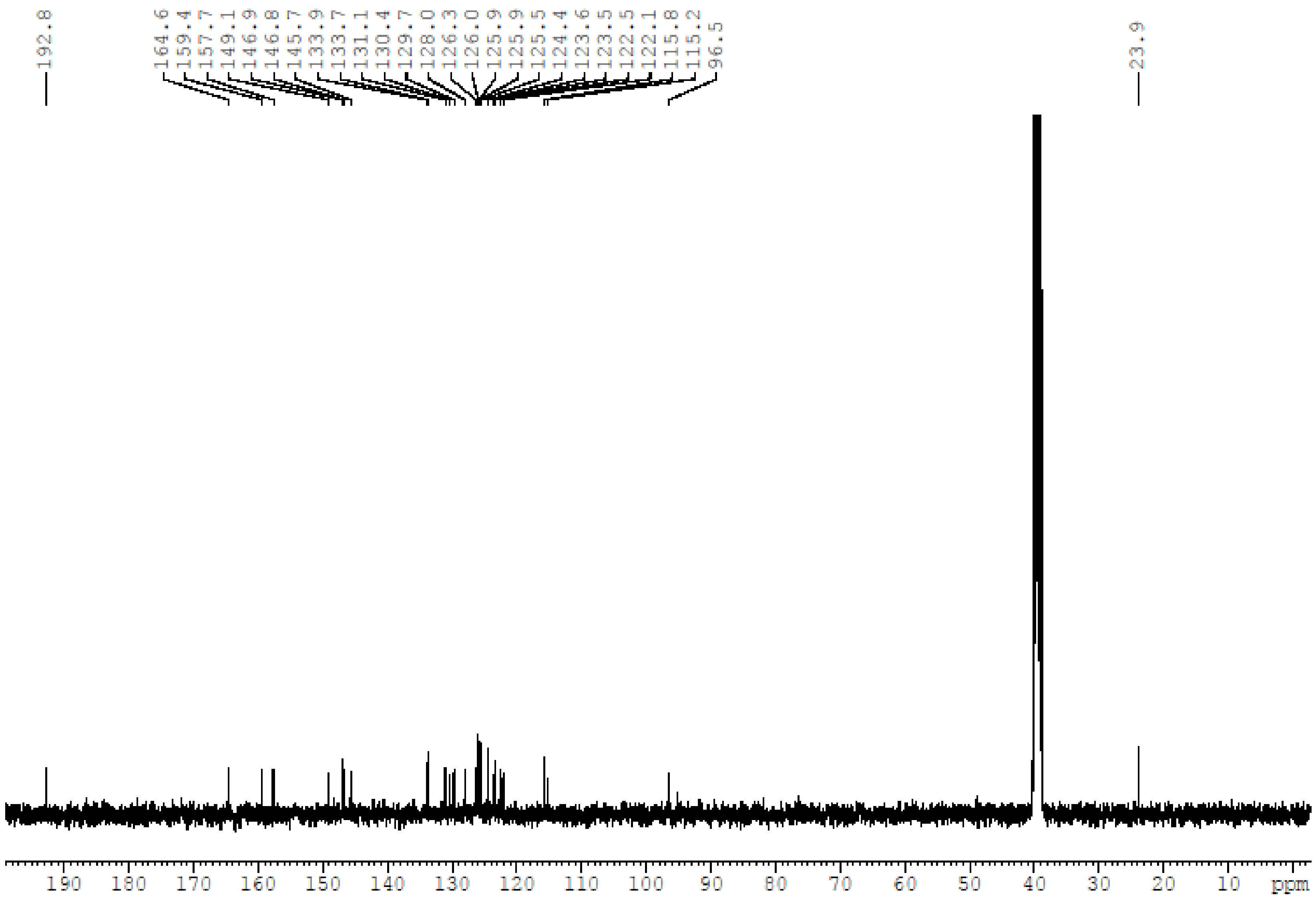

Figure S68: ^{13}C NMR spectrum of **15d** (100 MHz; DMSO-d_6).

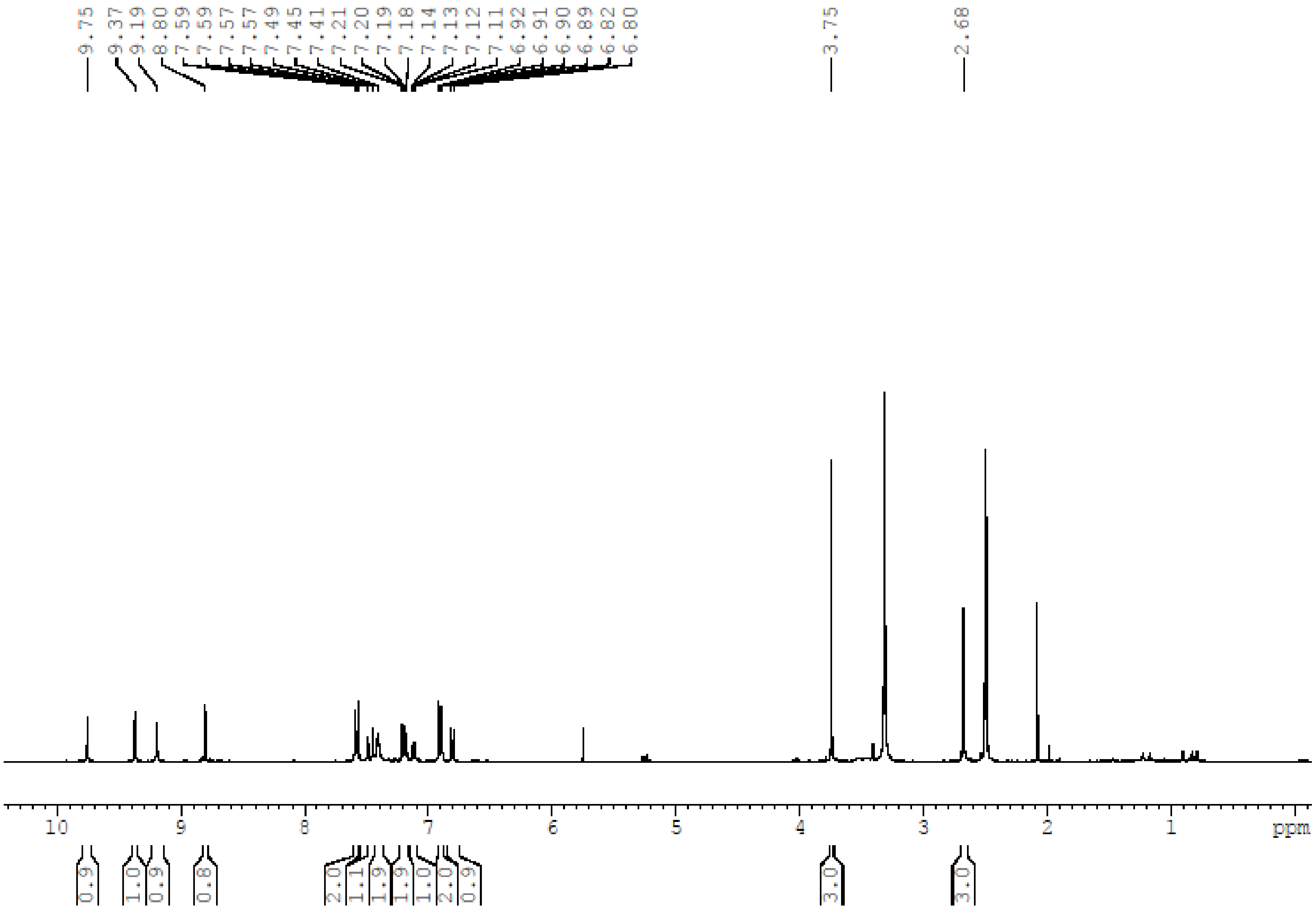

Figure S69: ^{1}H NMR spectrum of **15e** (400 MHz; DMSO-d_6).

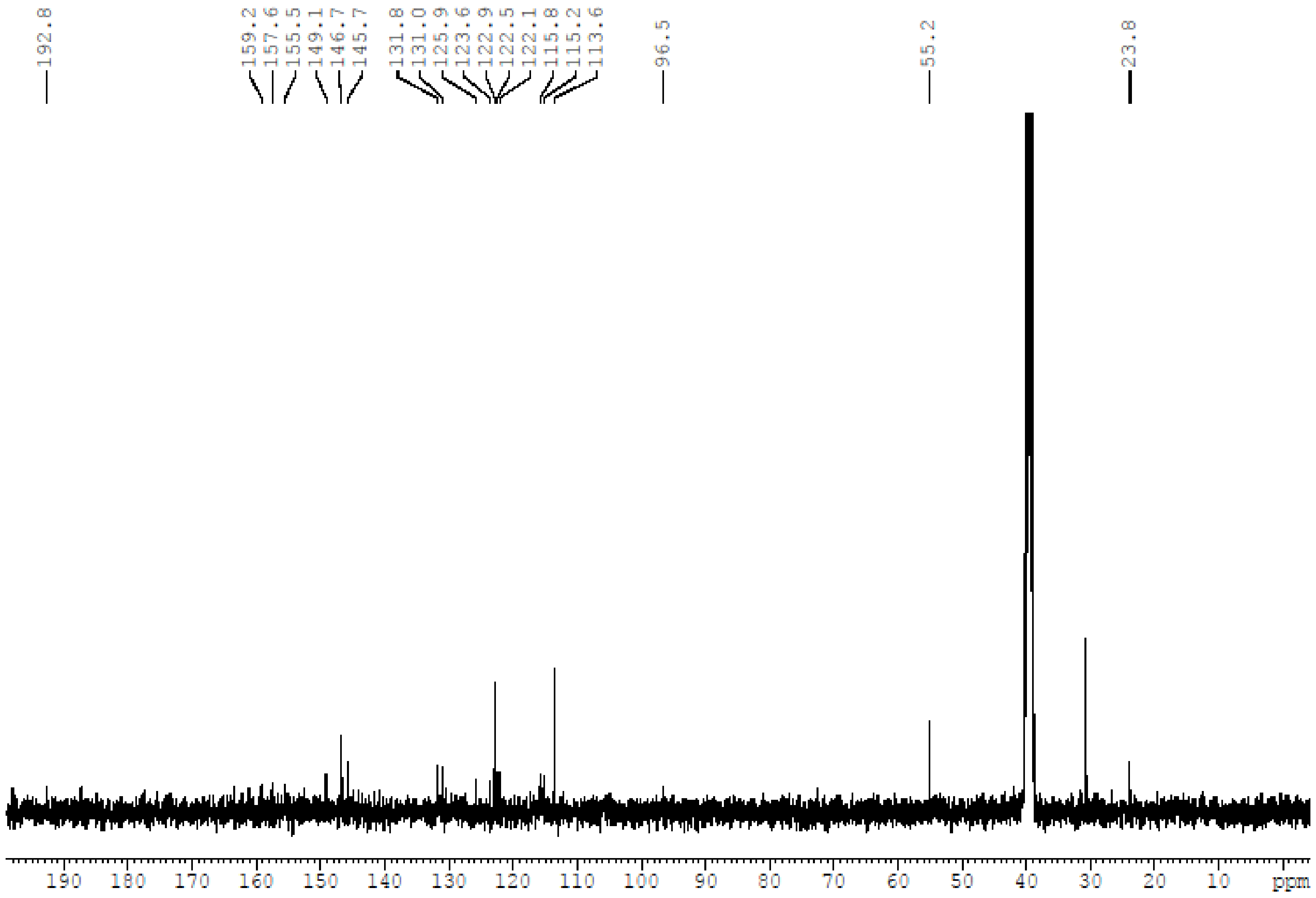

Figure S70: ^{13}C NMR spectrum of **15e** (100 MHz; DMSO-d_6).

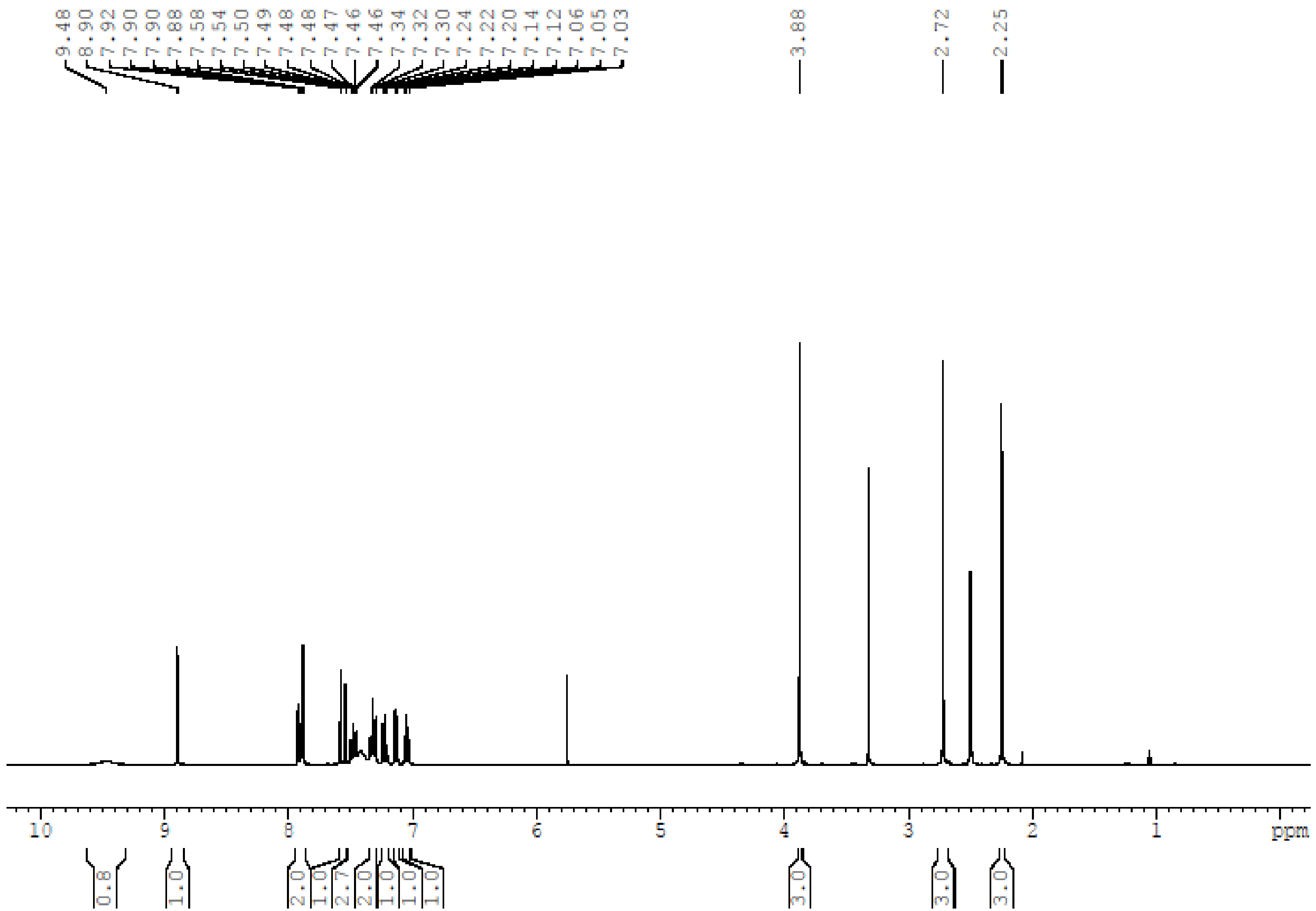

Figure S71: ^{1}H NMR spectrum of **17f** (400 MHz; DMSO-d_6).

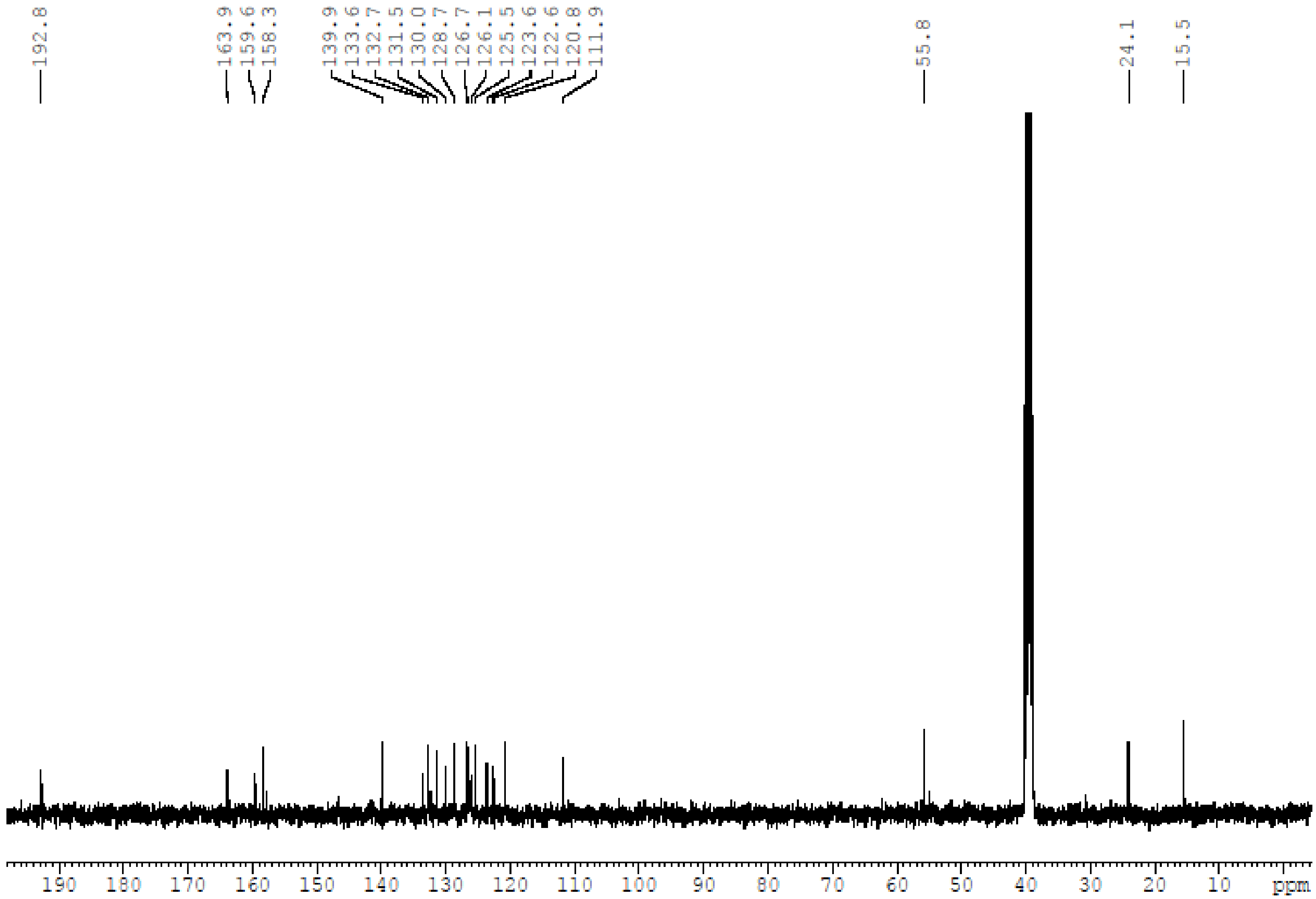

Figure S72: ^{13}C NMR spectrum of **17f** (100 MHz; DMSO-d_6).

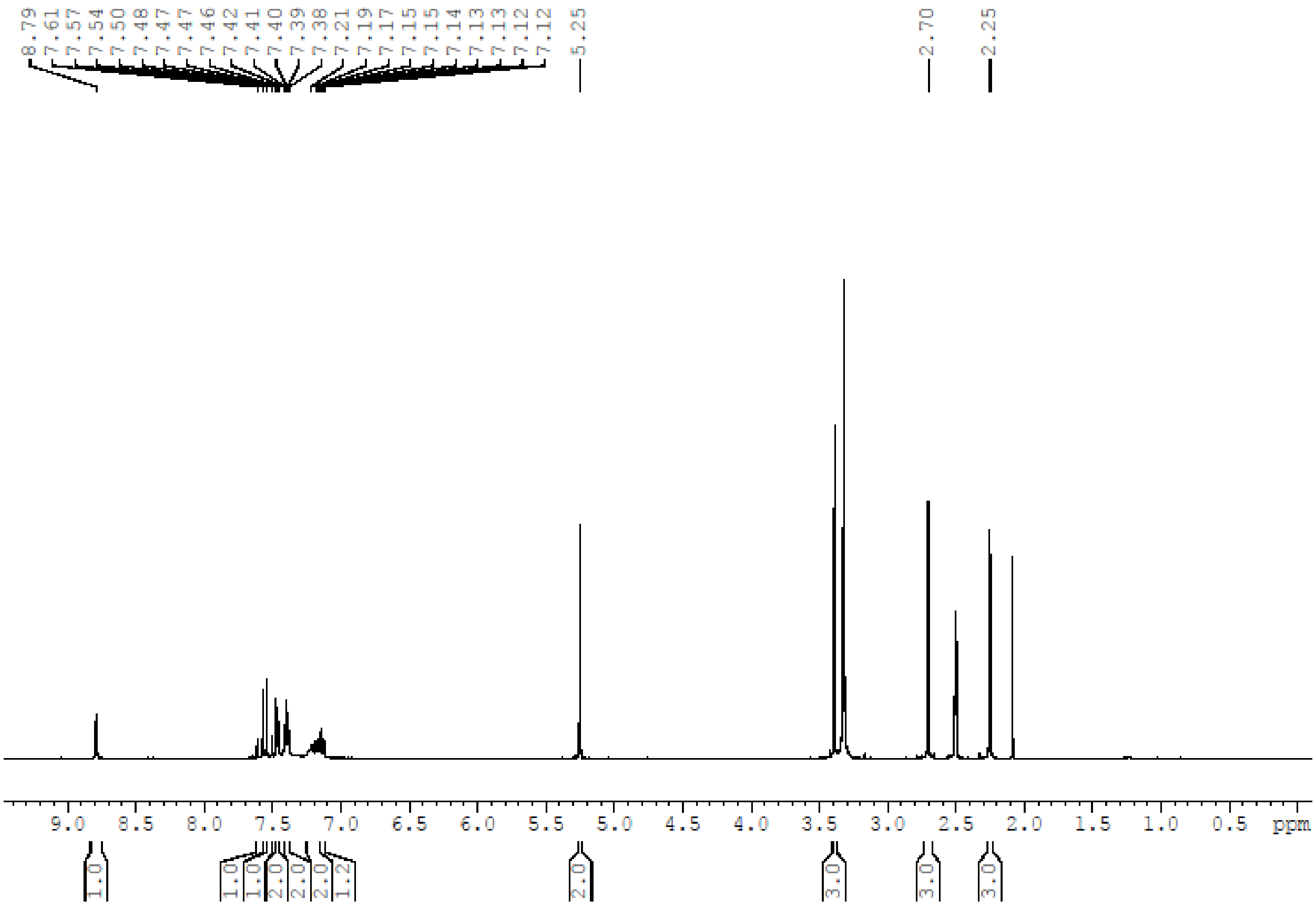

Figure S73: ^{1}H NMR spectrum of **17g** (400 MHz; DMSO-d_6).

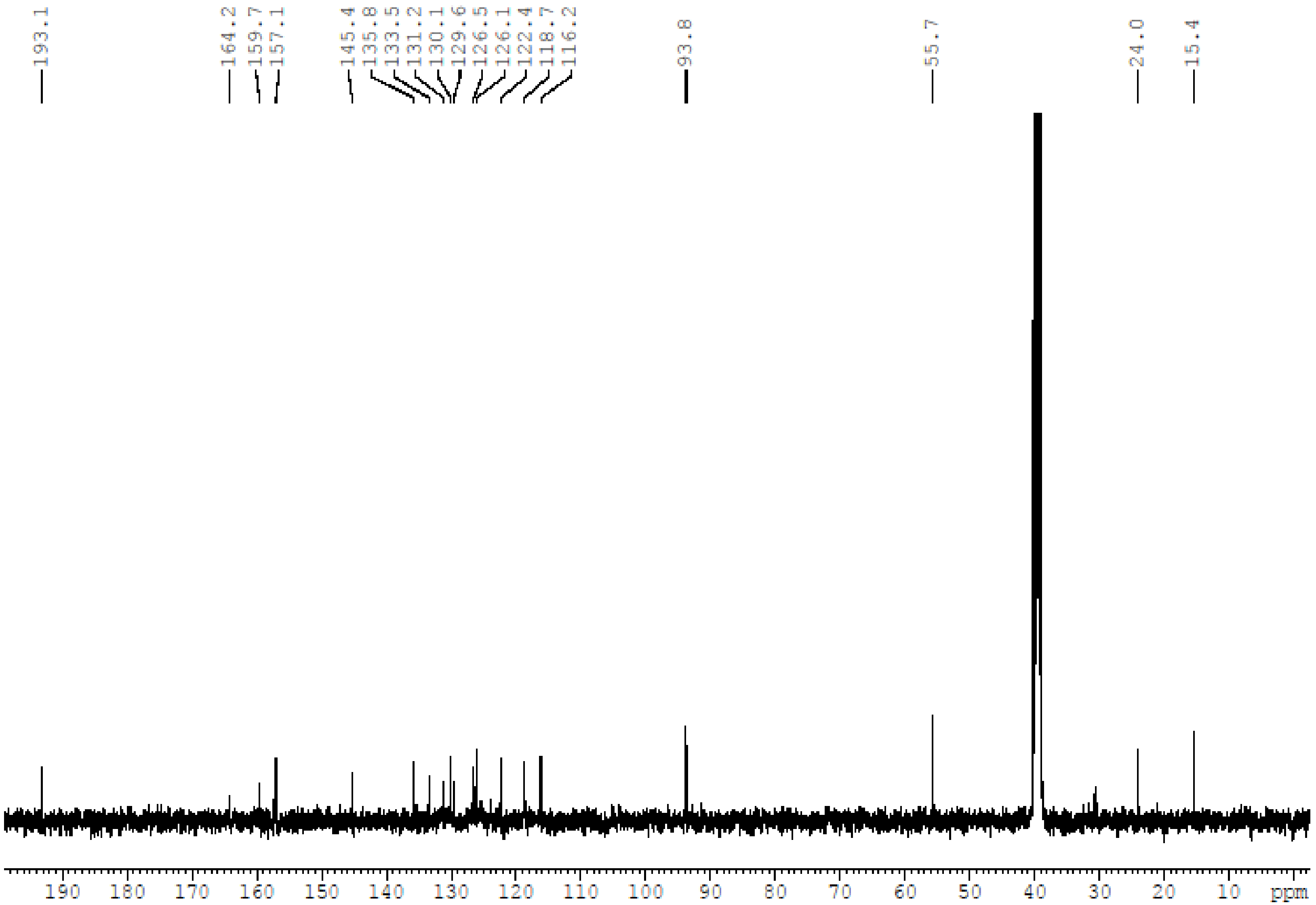

Figure S74: ^{13}C NMR spectrum of **17g** (100 MHz; DMSO-d_6).

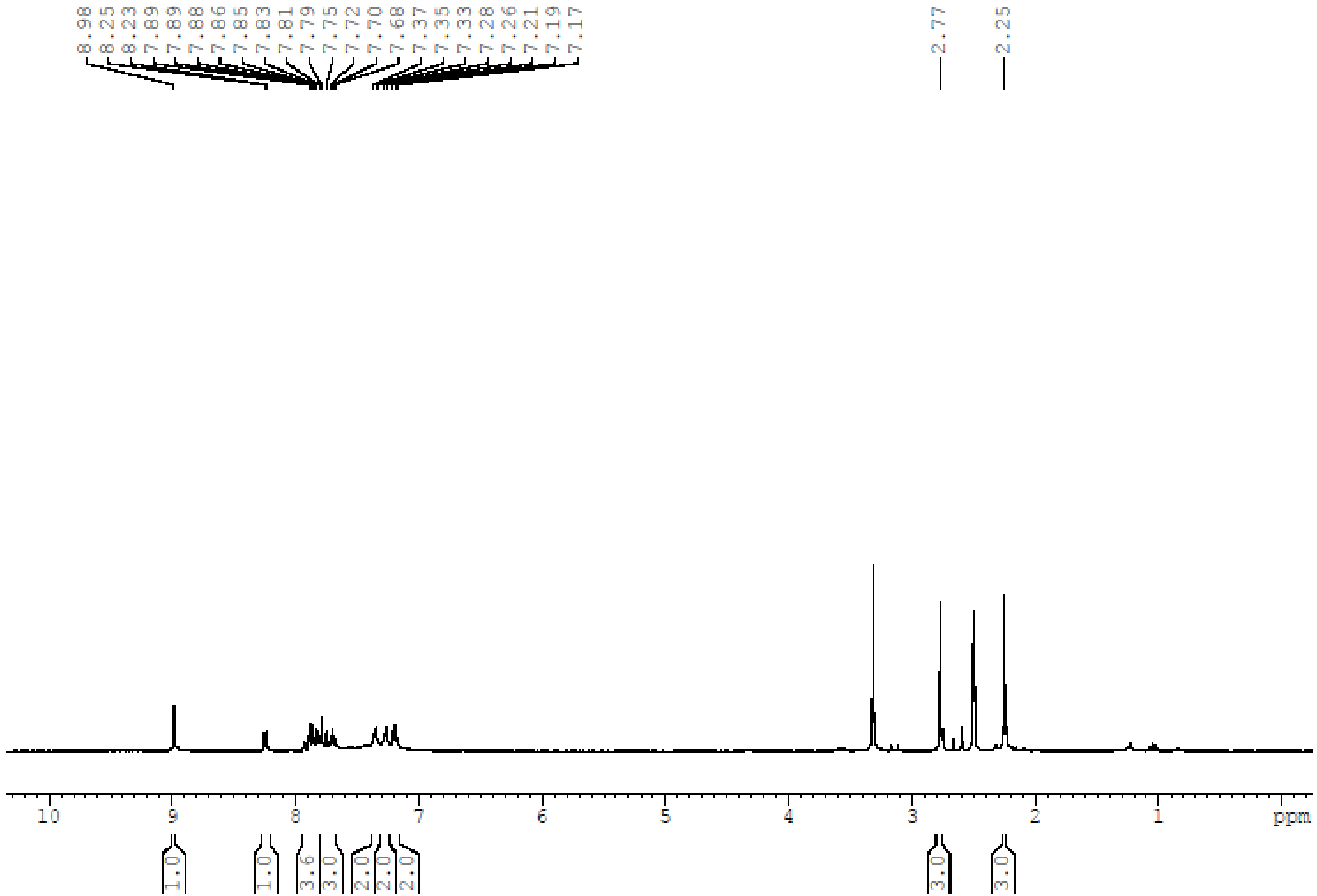

Figure S75: ^{1}H NMR spectrum of **17h** (400 MHz; DMSO-d_6).

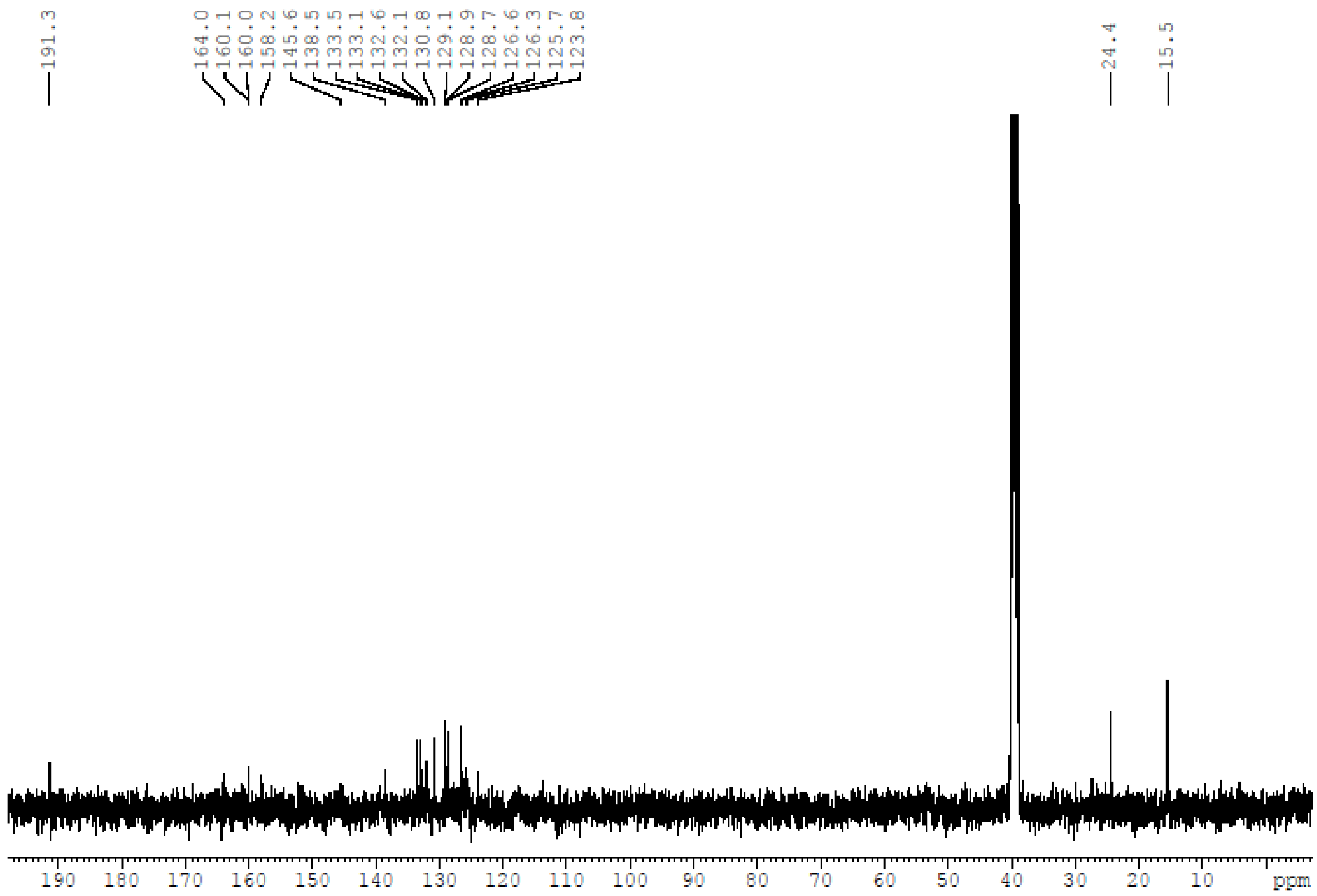

Figure S76: ^{13}C NMR spectrum of **17h** (100 MHz; DMSO-d_6).

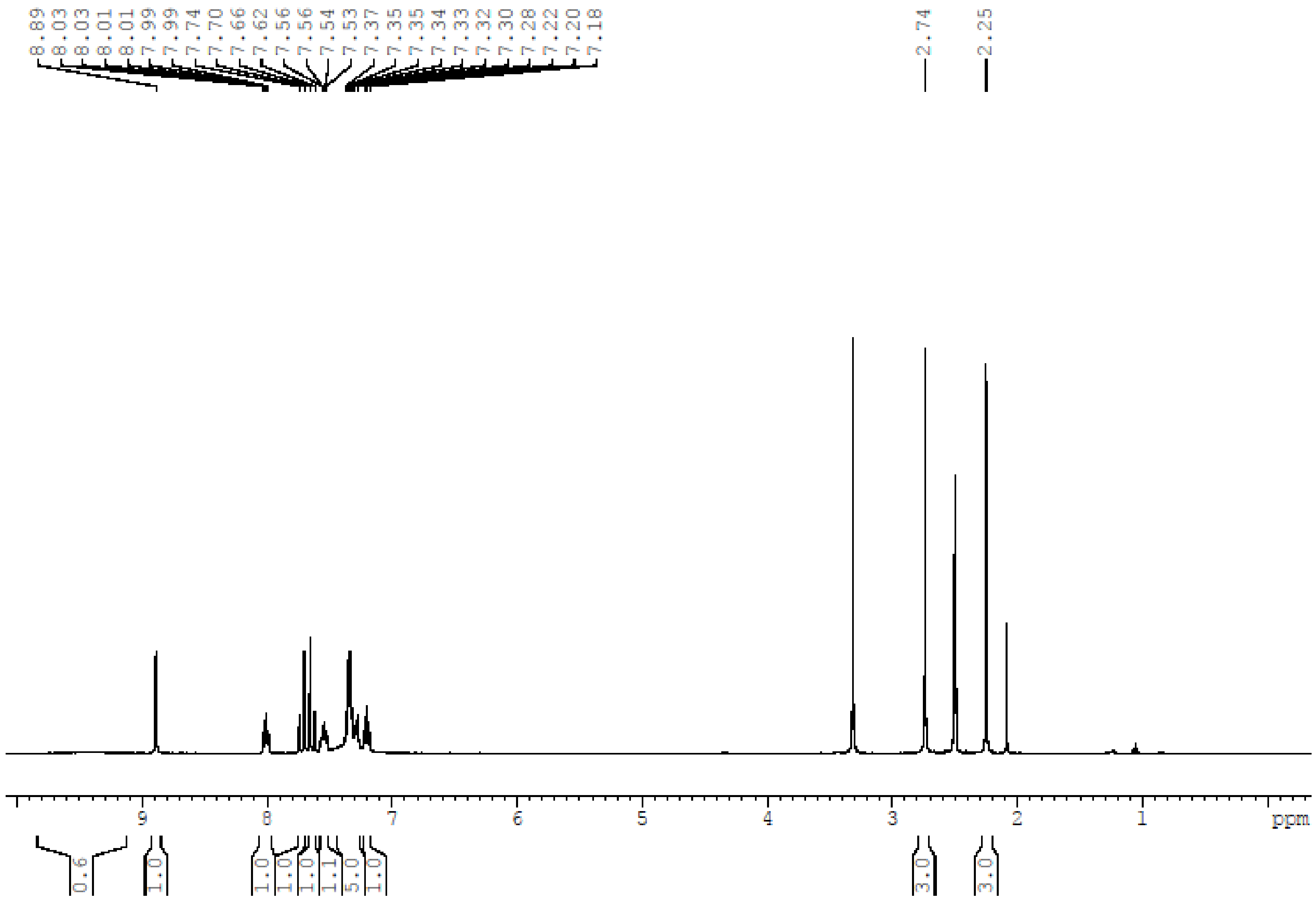

Figure S77: ^{1}H NMR spectrum of **17i** (400 MHz; DMSO-d_6).

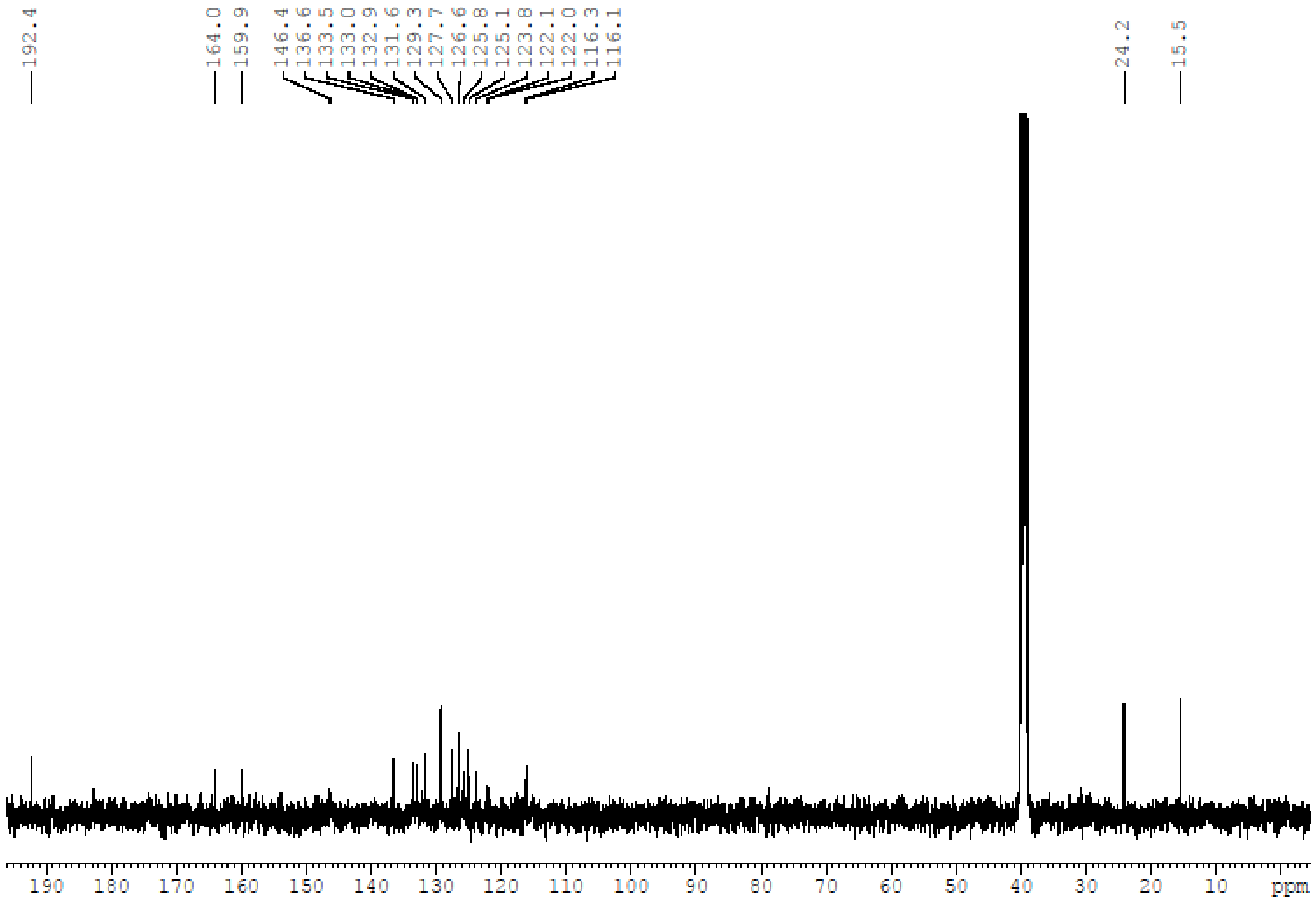

Figure S78: ^{13}C NMR spectrum of **17i** (100 MHz; DMSO-d_6).

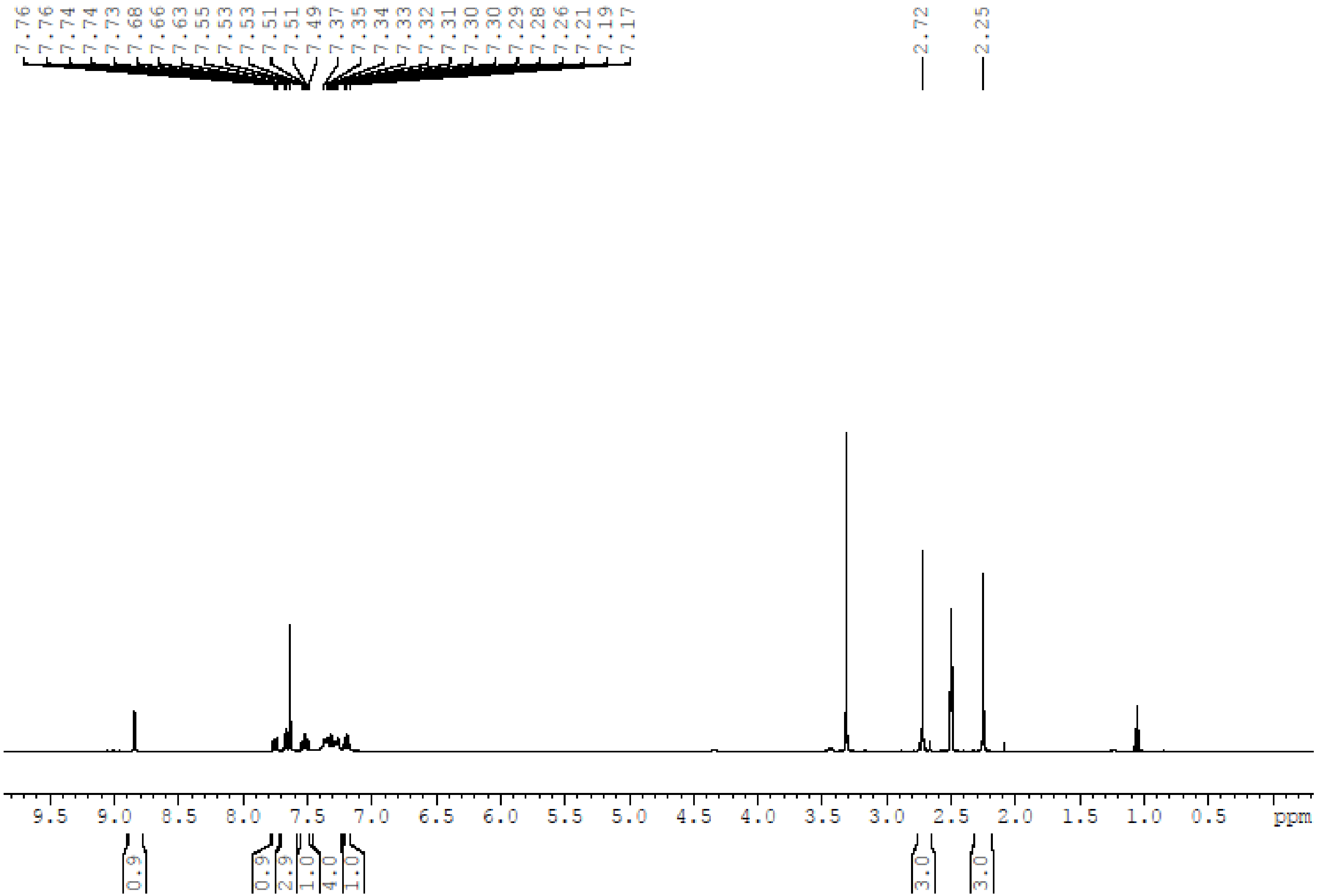

Figure S79: ^{1}H NMR spectrum of **17j** (400 MHz; DMSO-d_6).

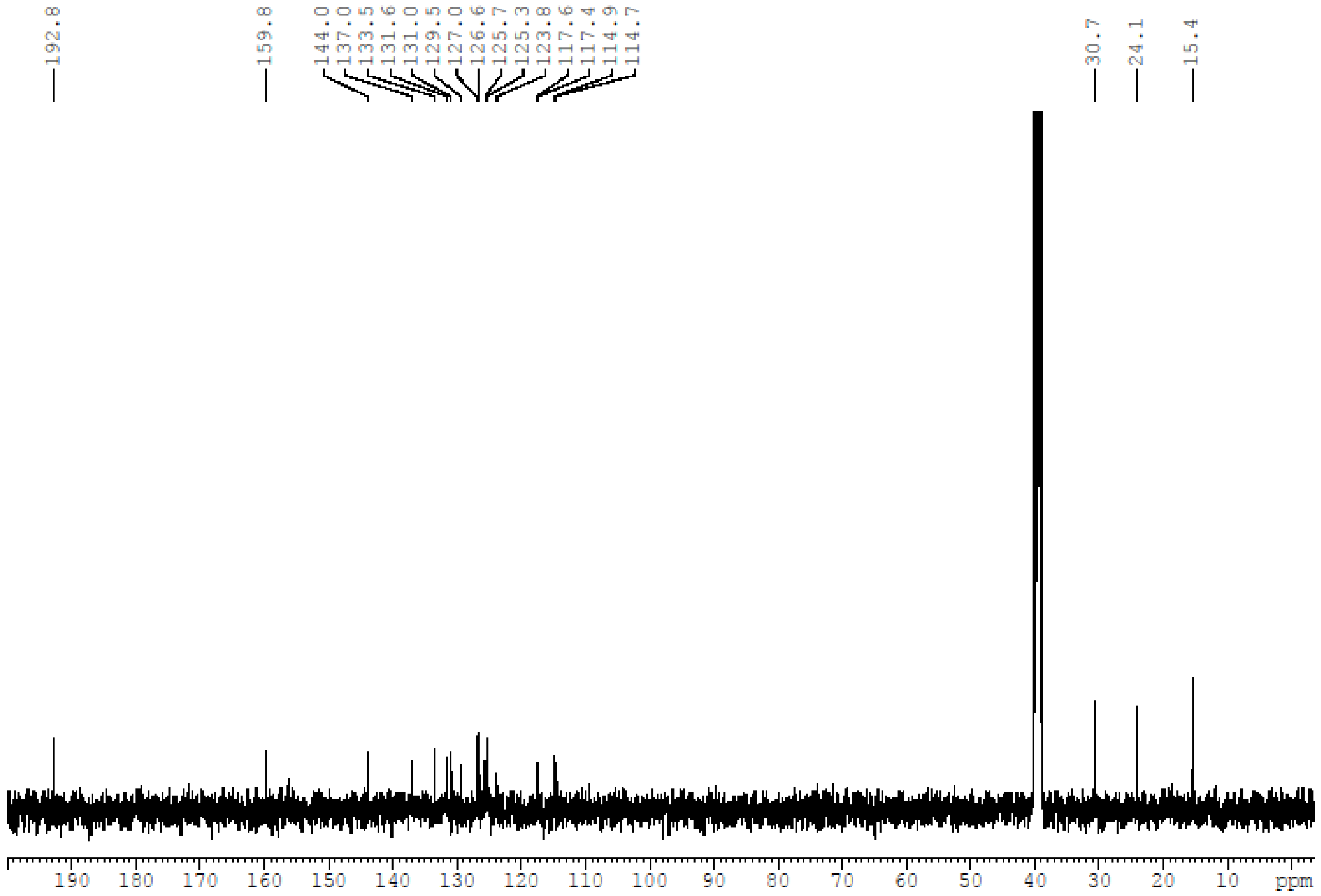

Figure S80: ^{13}C NMR spectrum of **17j** (100 MHz; DMSO-d_6).

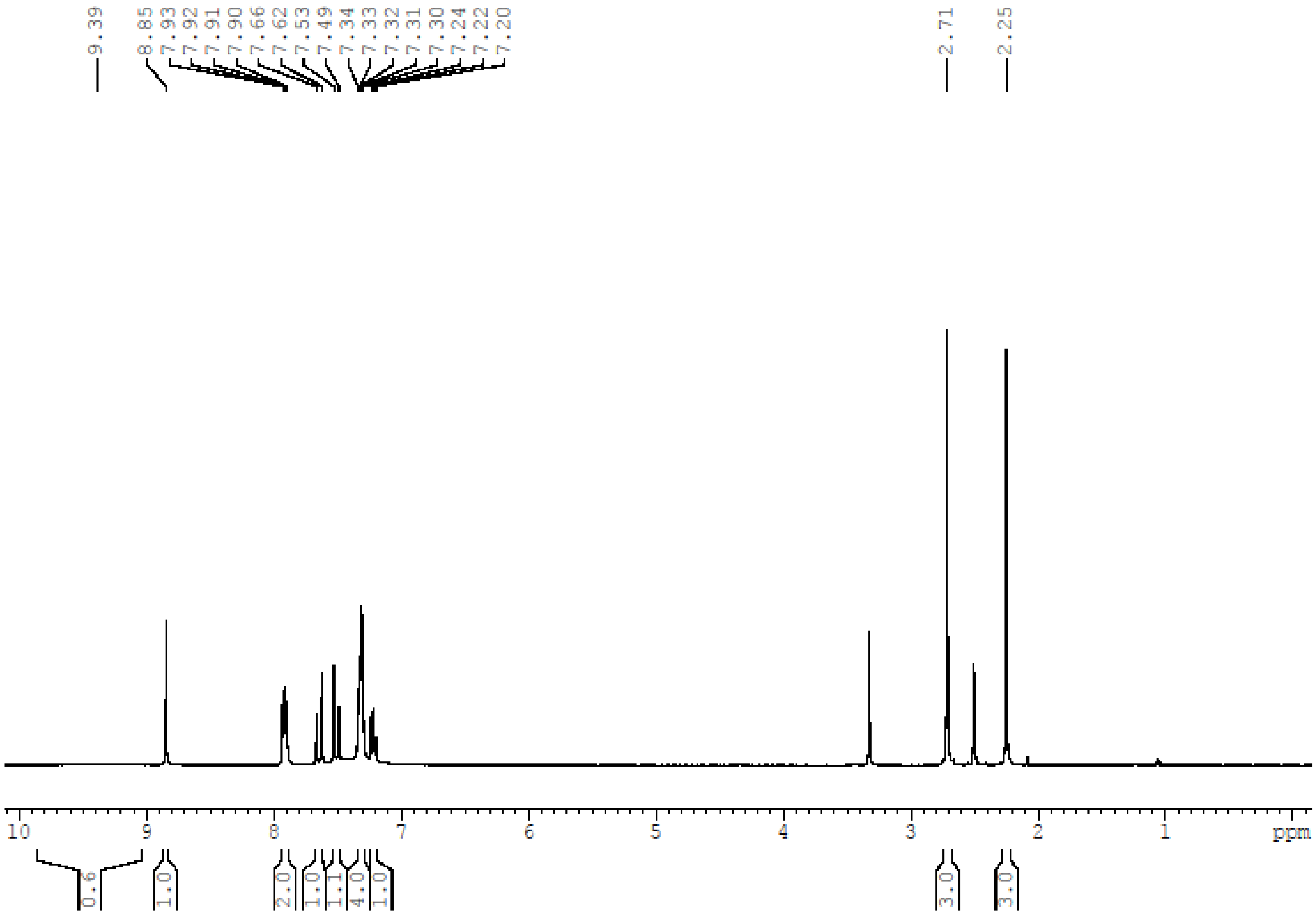

Figure S81: ^{1}H NMR spectrum of **17k** (400 MHz; DMSO-d_6).

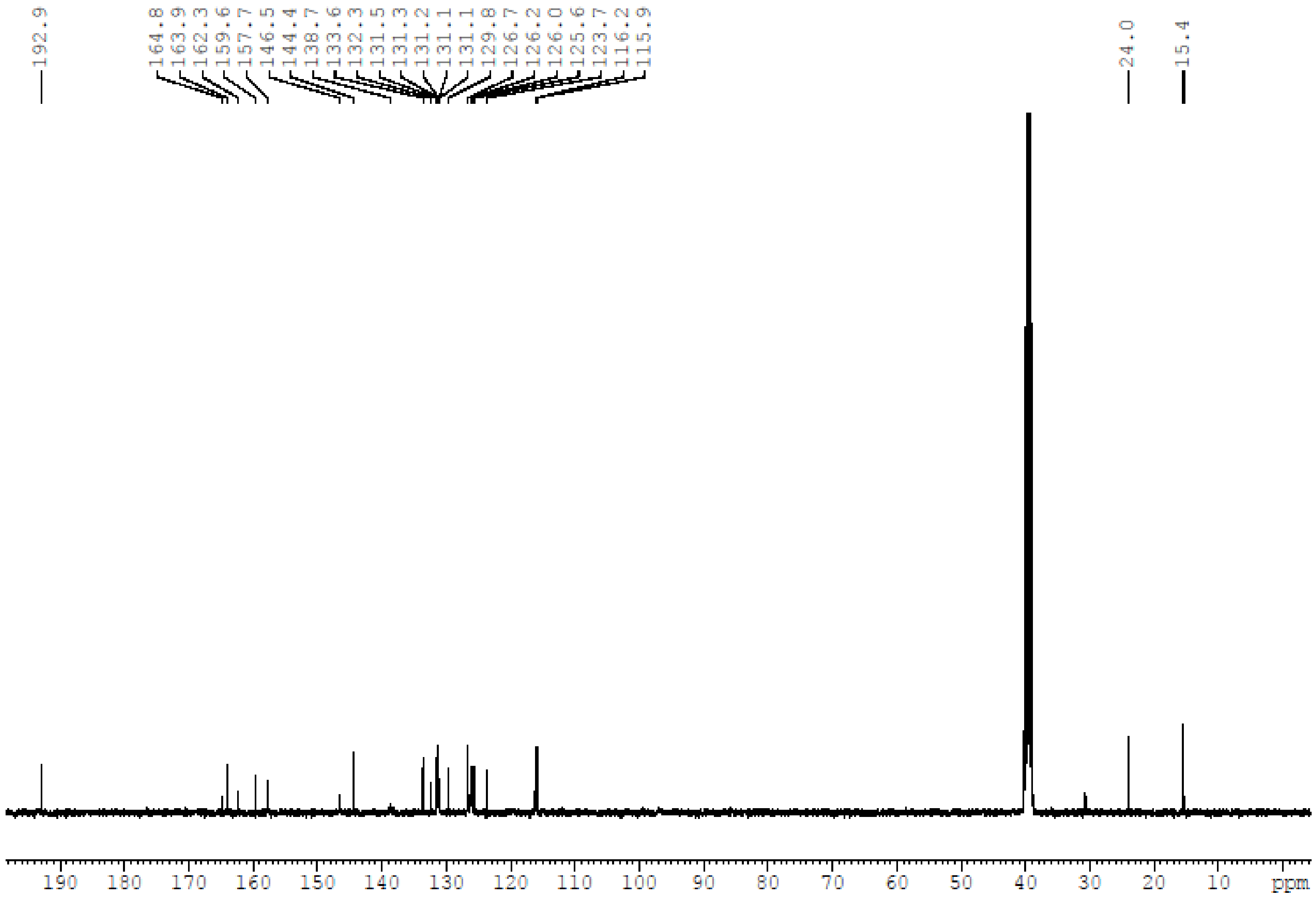

Figure S82: ^{13}C NMR spectrum of **17k** (100 MHz; DMSO-d_6).

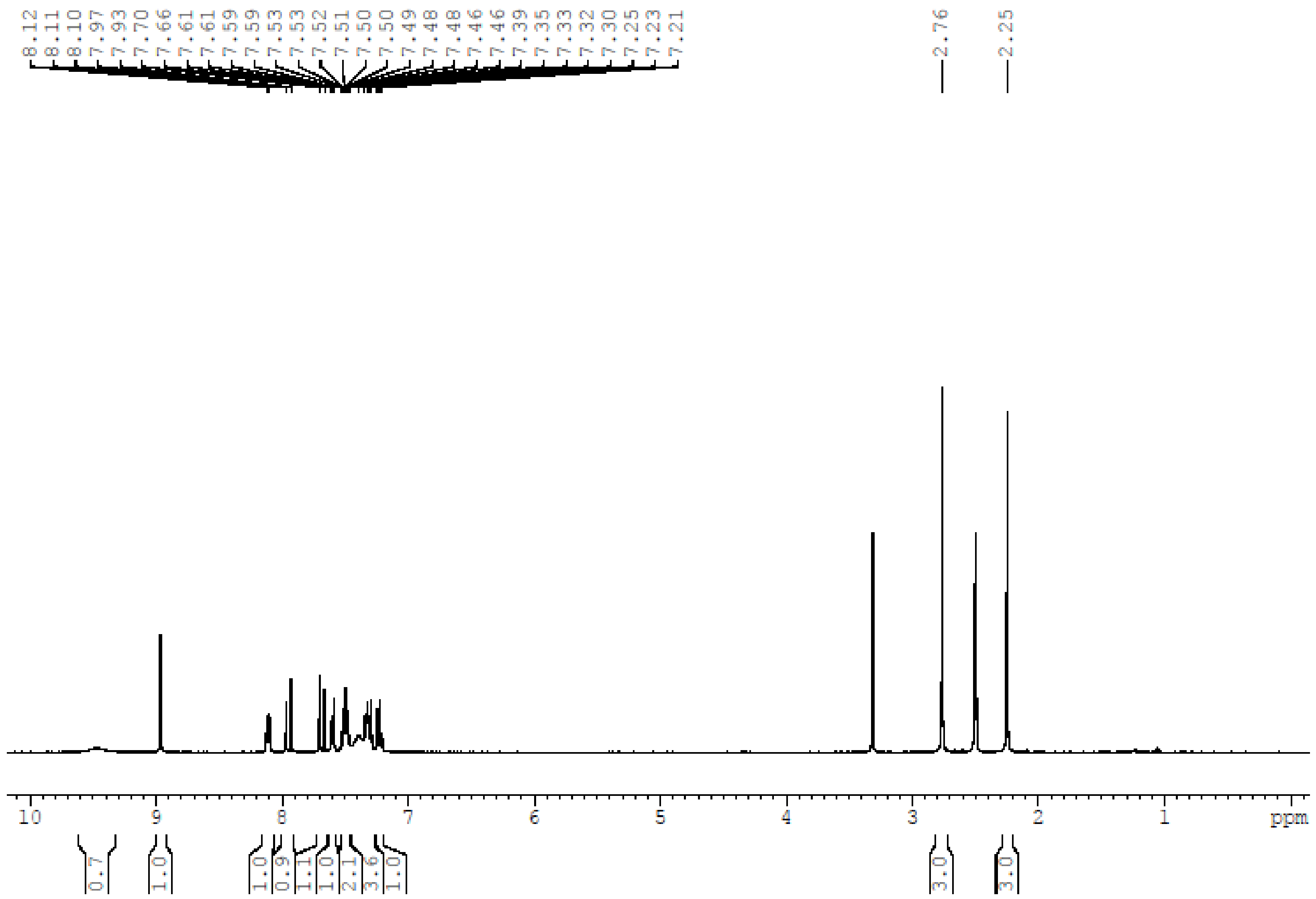

Figure S83: ^{1}H NMR spectrum of **17l** (400 MHz; DMSO-d_6).

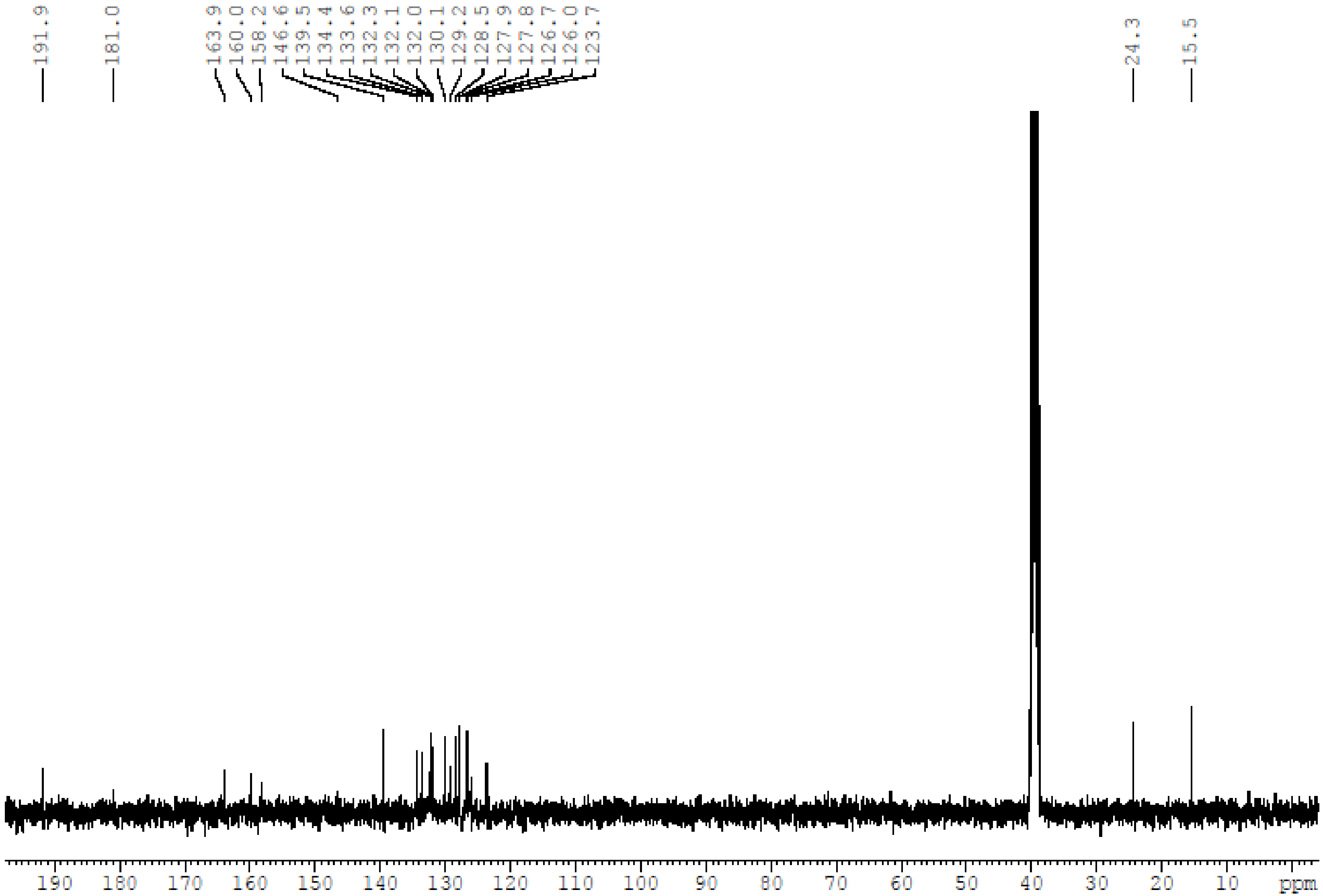

Figure S84: ^{13}C NMR spectrum of **17l** (100 MHz; DMSO-d_6).

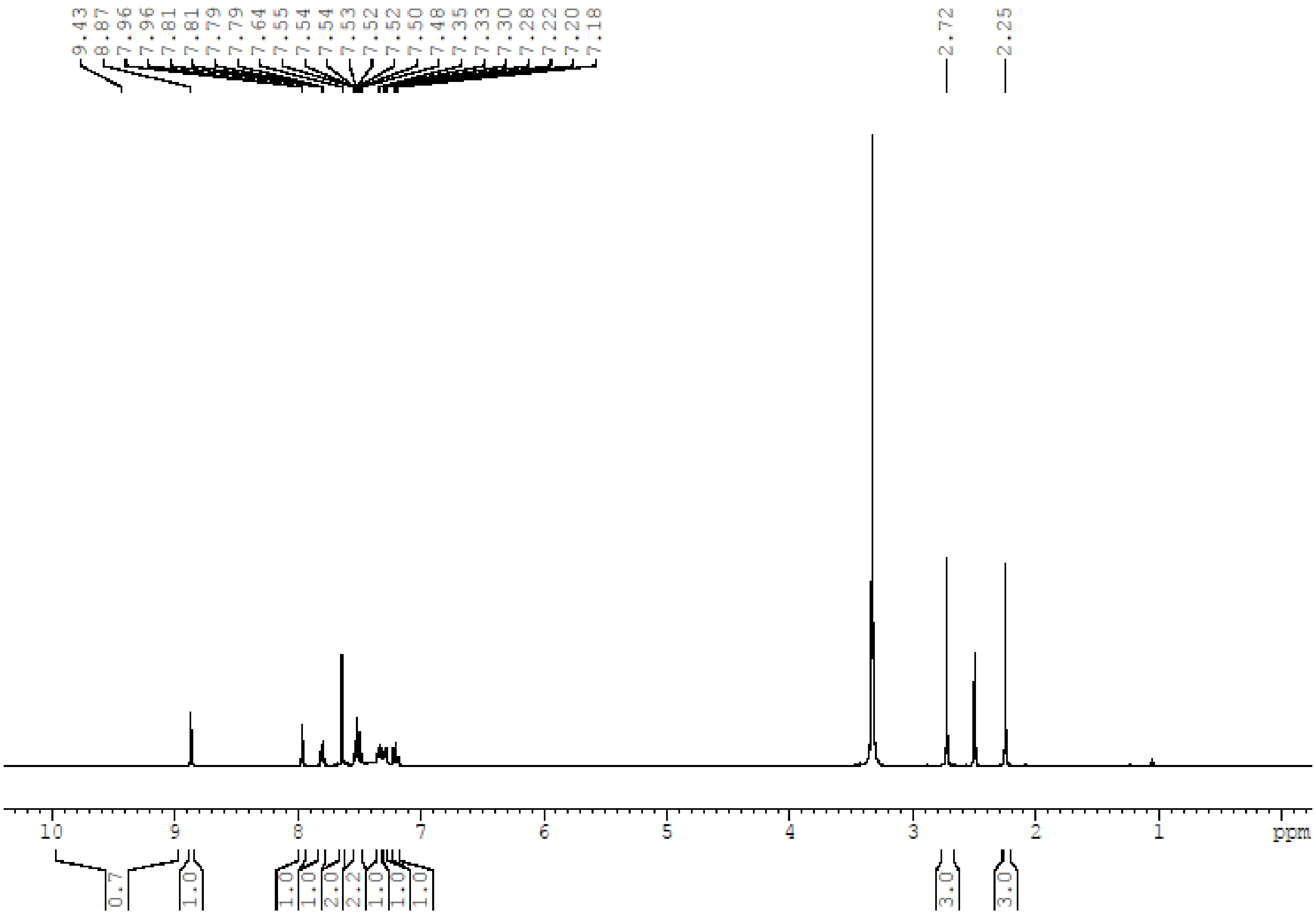

Figure S85: ^{1}H NMR spectrum of **17m** (400 MHz; DMSO-d_6).

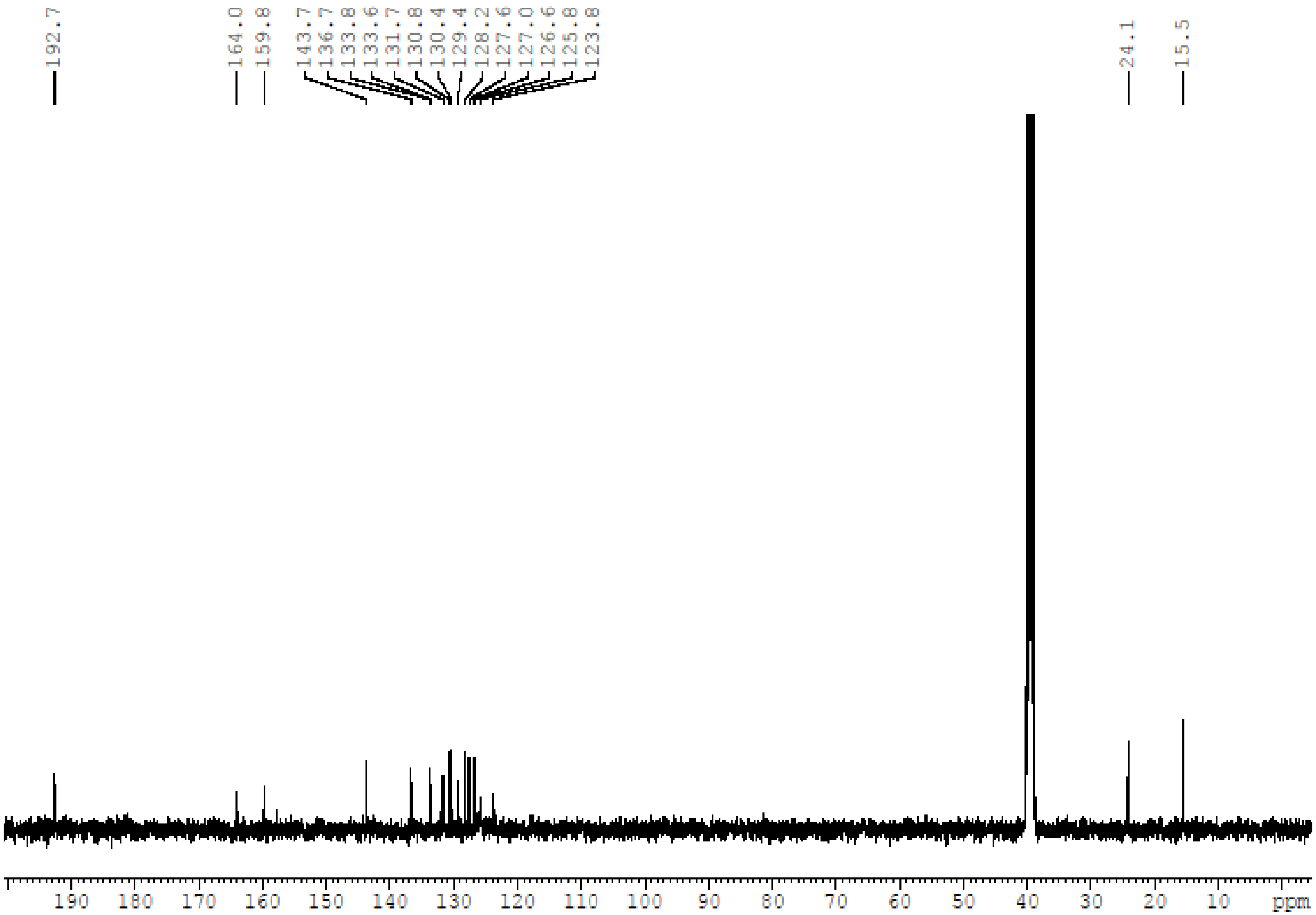

Figure S86: ^{13}C NMR spectrum of **17m** (100 MHz; DMSO-d_6).

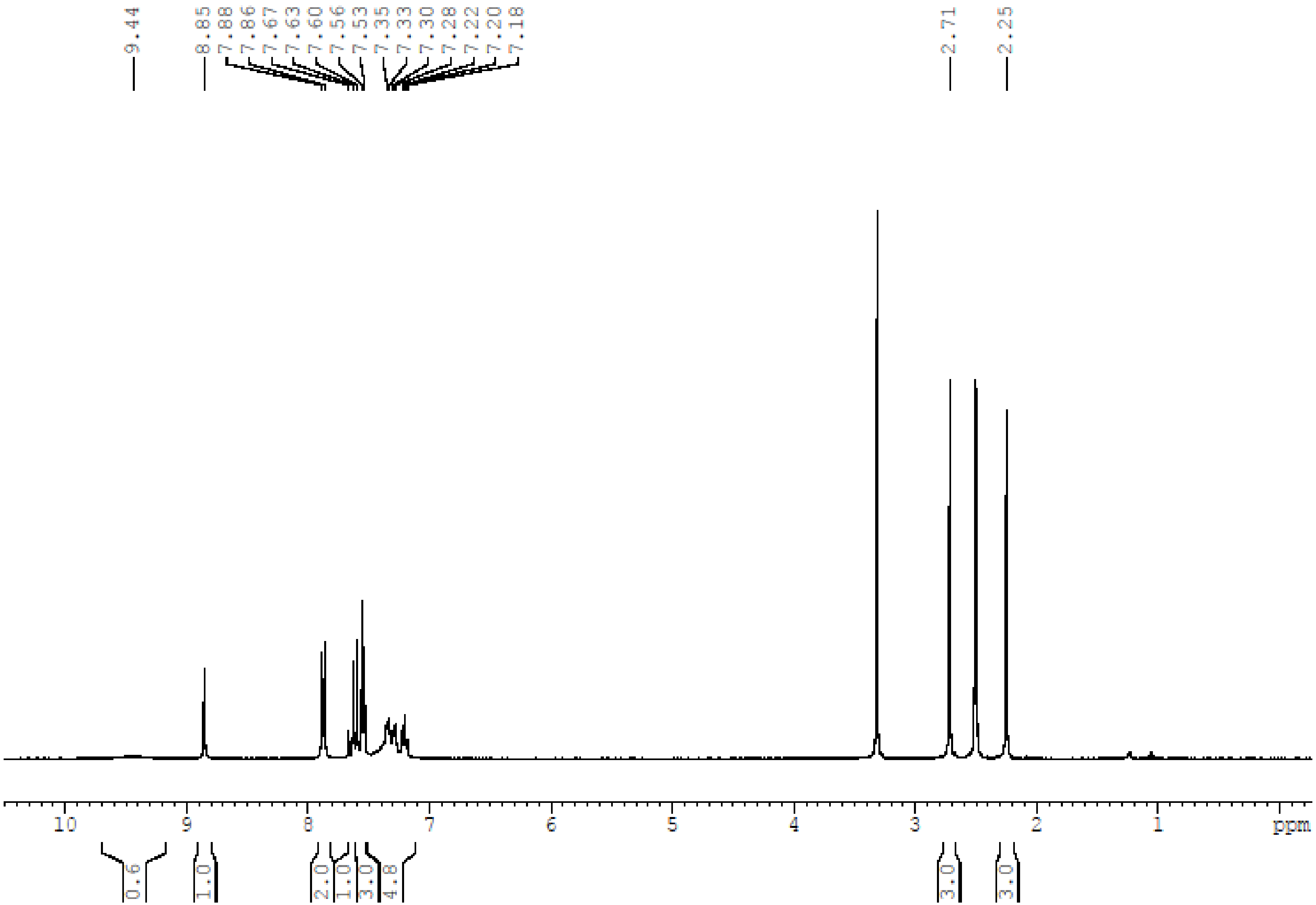

Figure S87: ^{1}H NMR spectrum of **17n** (400 MHz; DMSO-d_6).

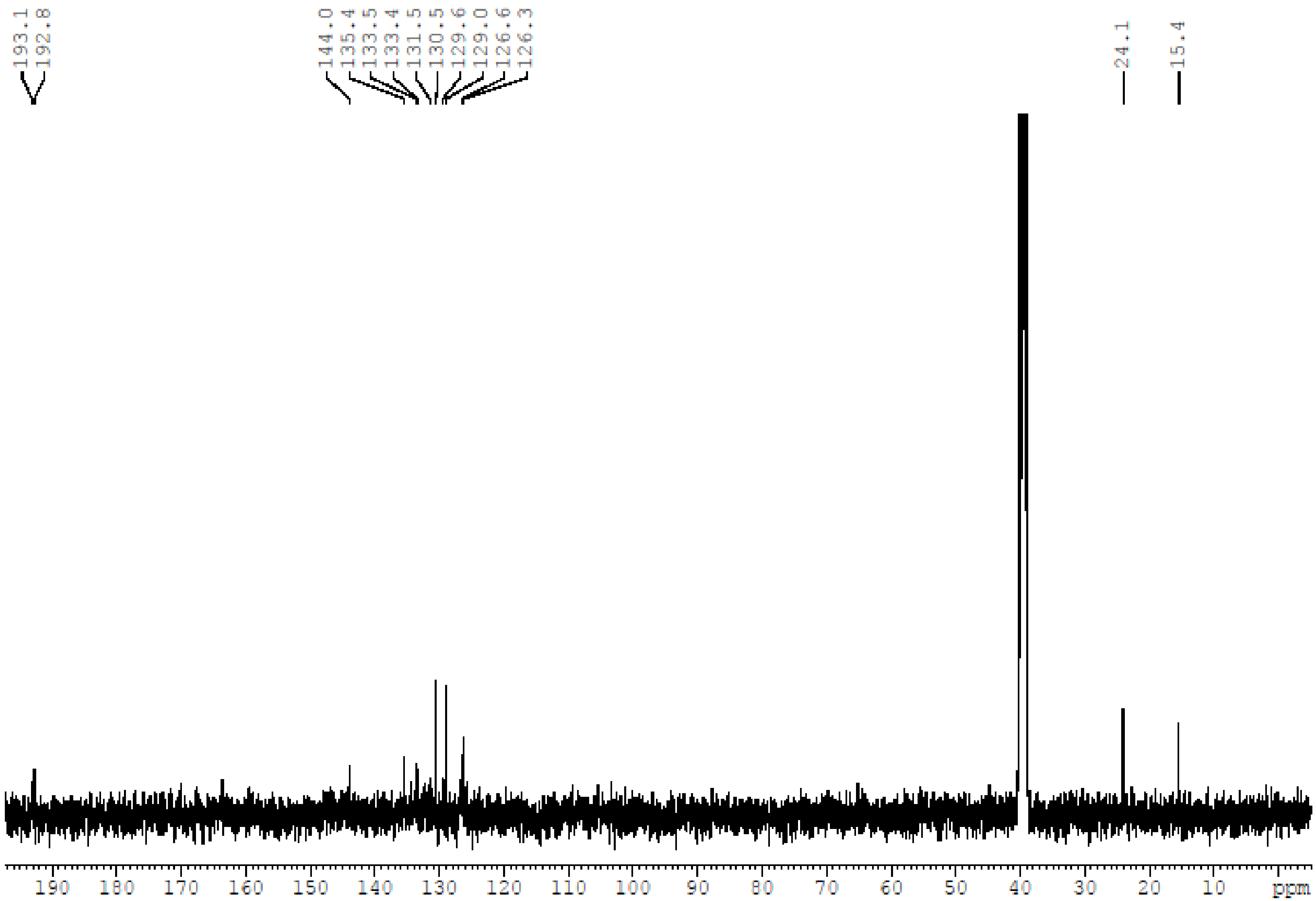

Figure S88: ^{13}C NMR spectrum of **17n** (100 MHz; DMSO-d_6).

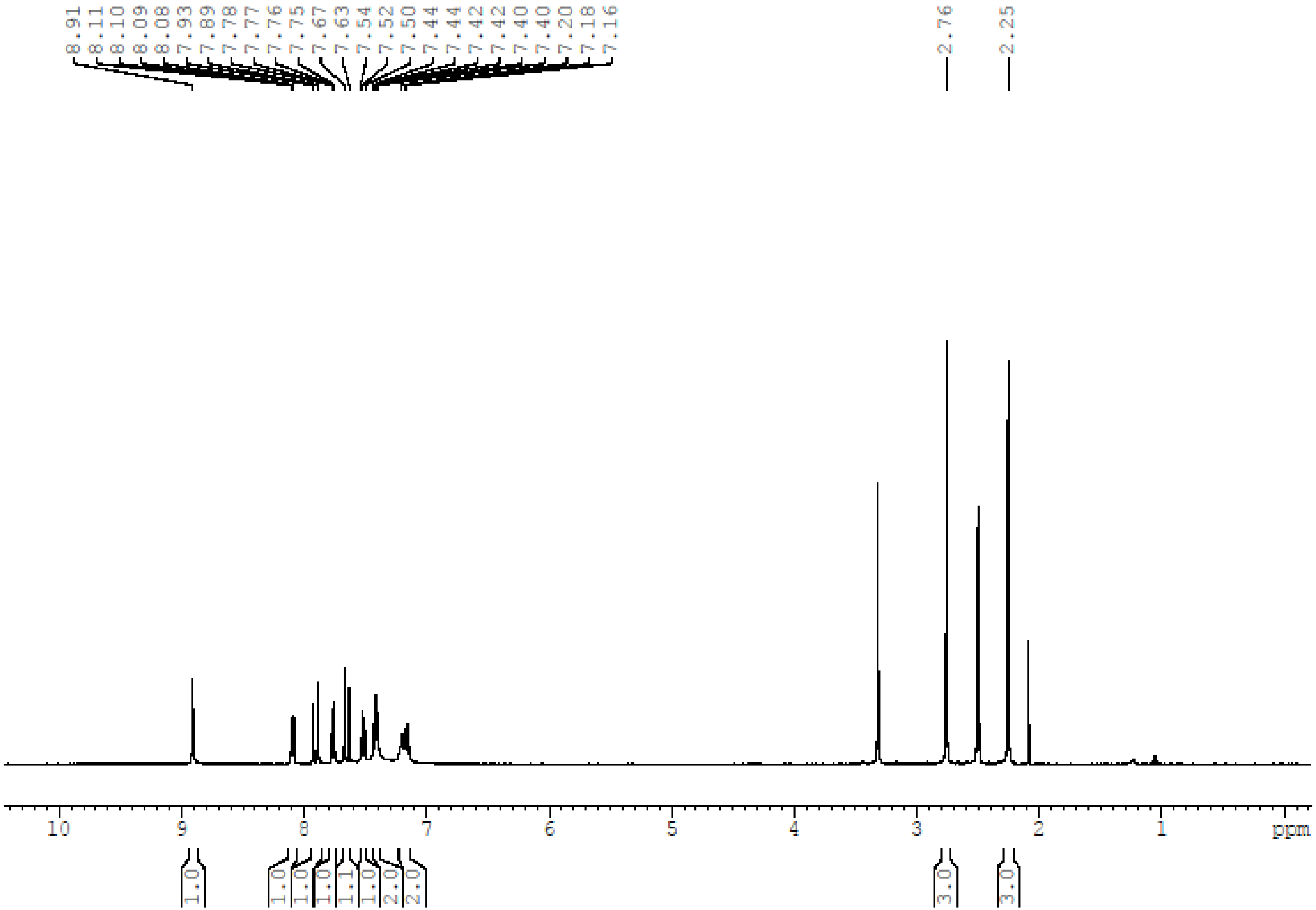

Figure S89: ^{1}H NMR spectrum of **17o** (400 MHz; DMSO-d_6).

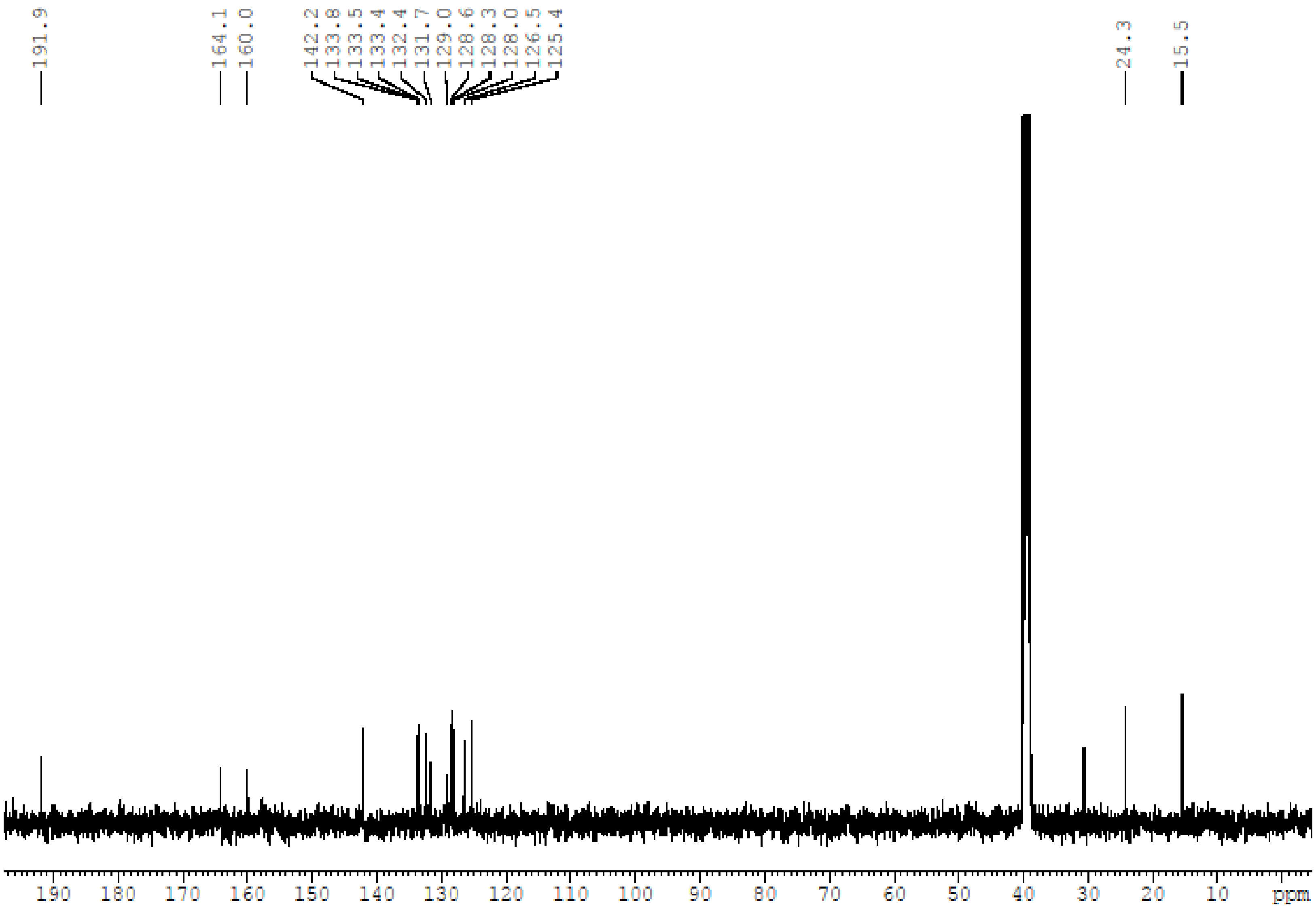

Figure S90: ^{13}C NMR spectrum of **17o** (100 MHz; DMSO-d_6).

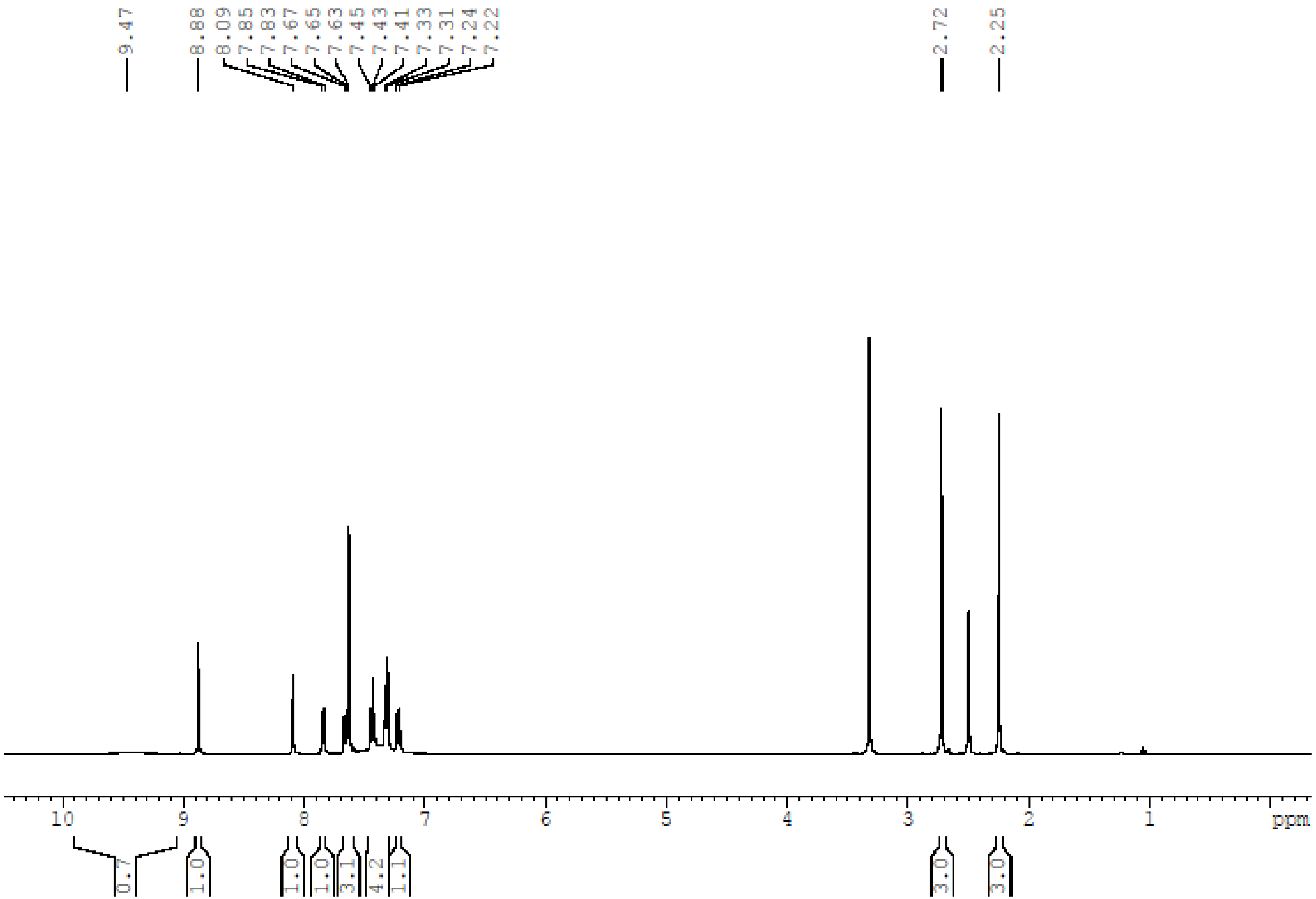

Figure S91: ^{1}H NMR spectrum of **17p** (400 MHz; DMSO-d_6).

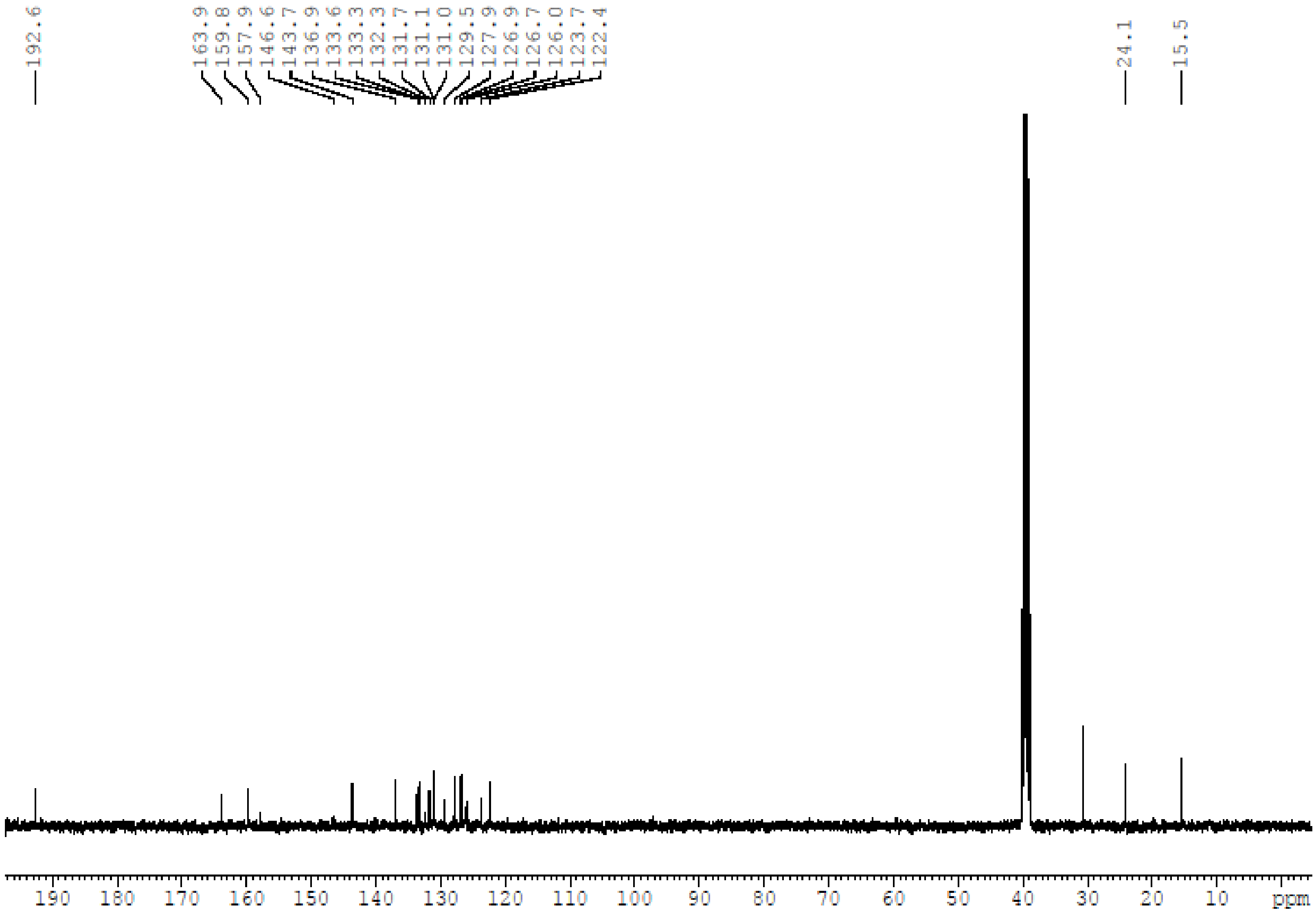

Figure S92: ^{13}C NMR spectrum of **17p** (100 MHz; DMSO-d_6).

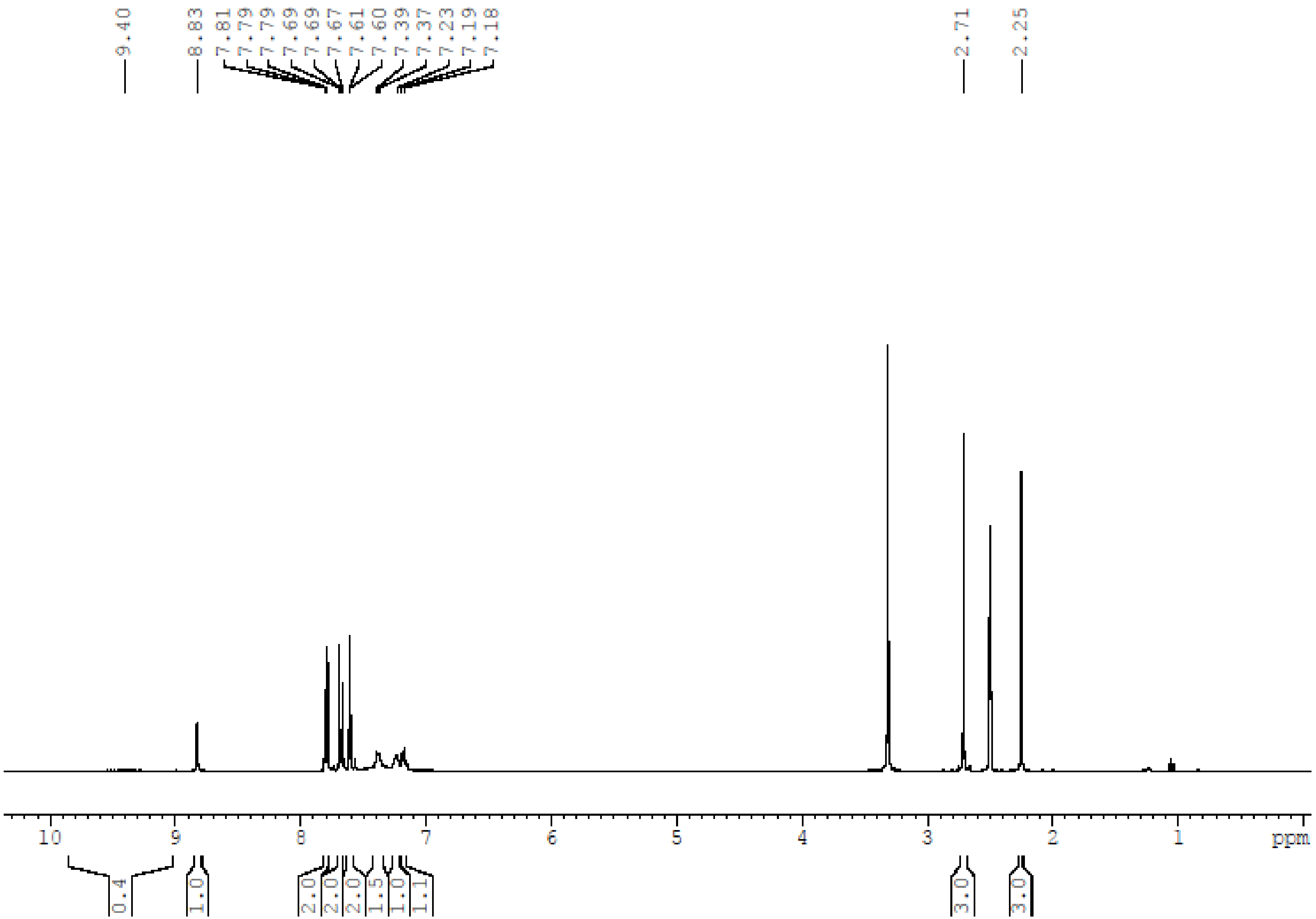

Figure S93: ^{1}H NMR spectrum of **17q** (400 MHz; DMSO-d_6).

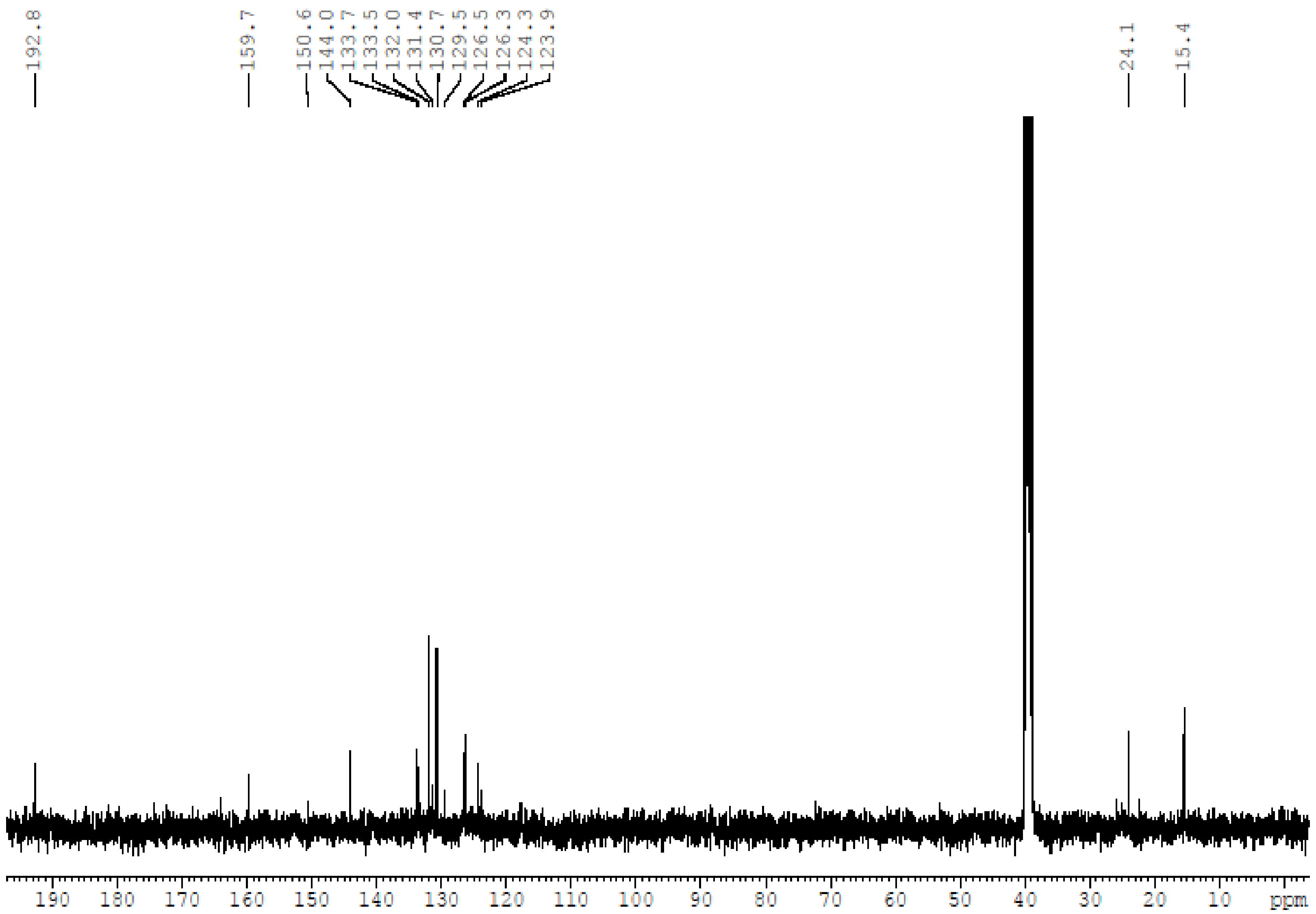

Figure S94: ^{13}C NMR spectrum of **17q** (100 MHz; DMSO-*d*$_6$).

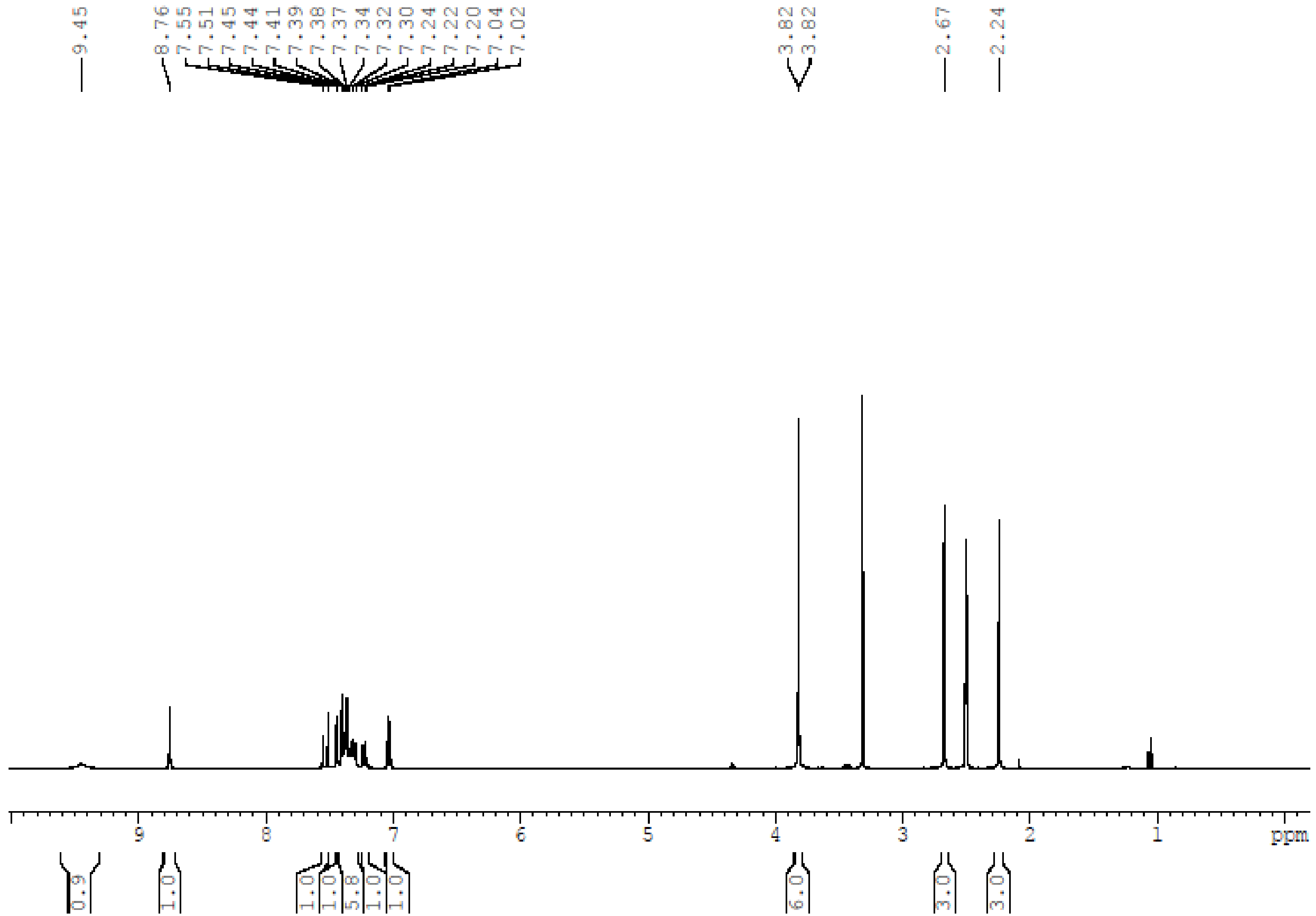

Figure S95: ^{1}H NMR spectrum of **17r** (400 MHz; DMSO-d_6).

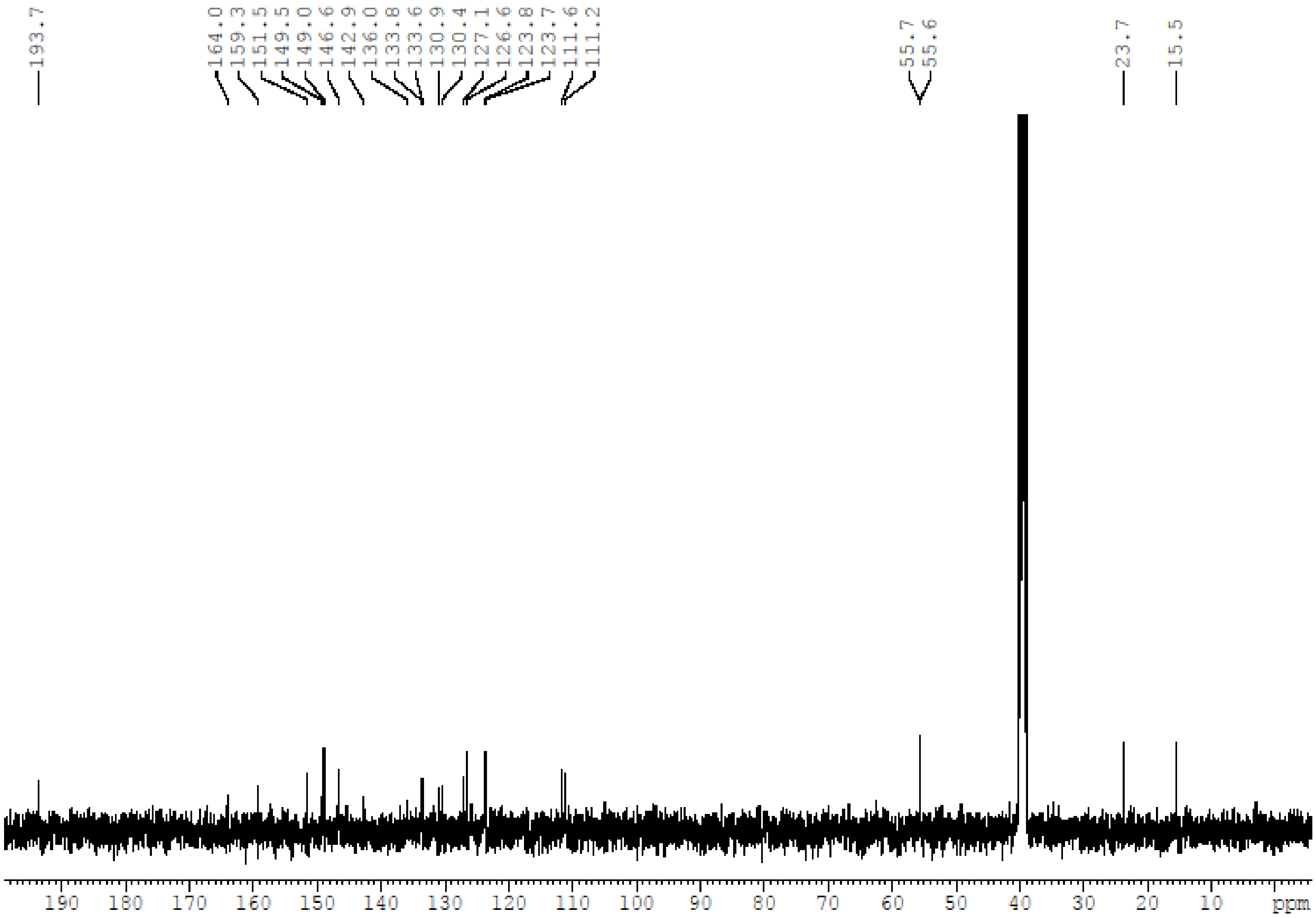

Figure S96: ^{13}C NMR spectrum of **17r** (100 MHz; DMSO-d_6).

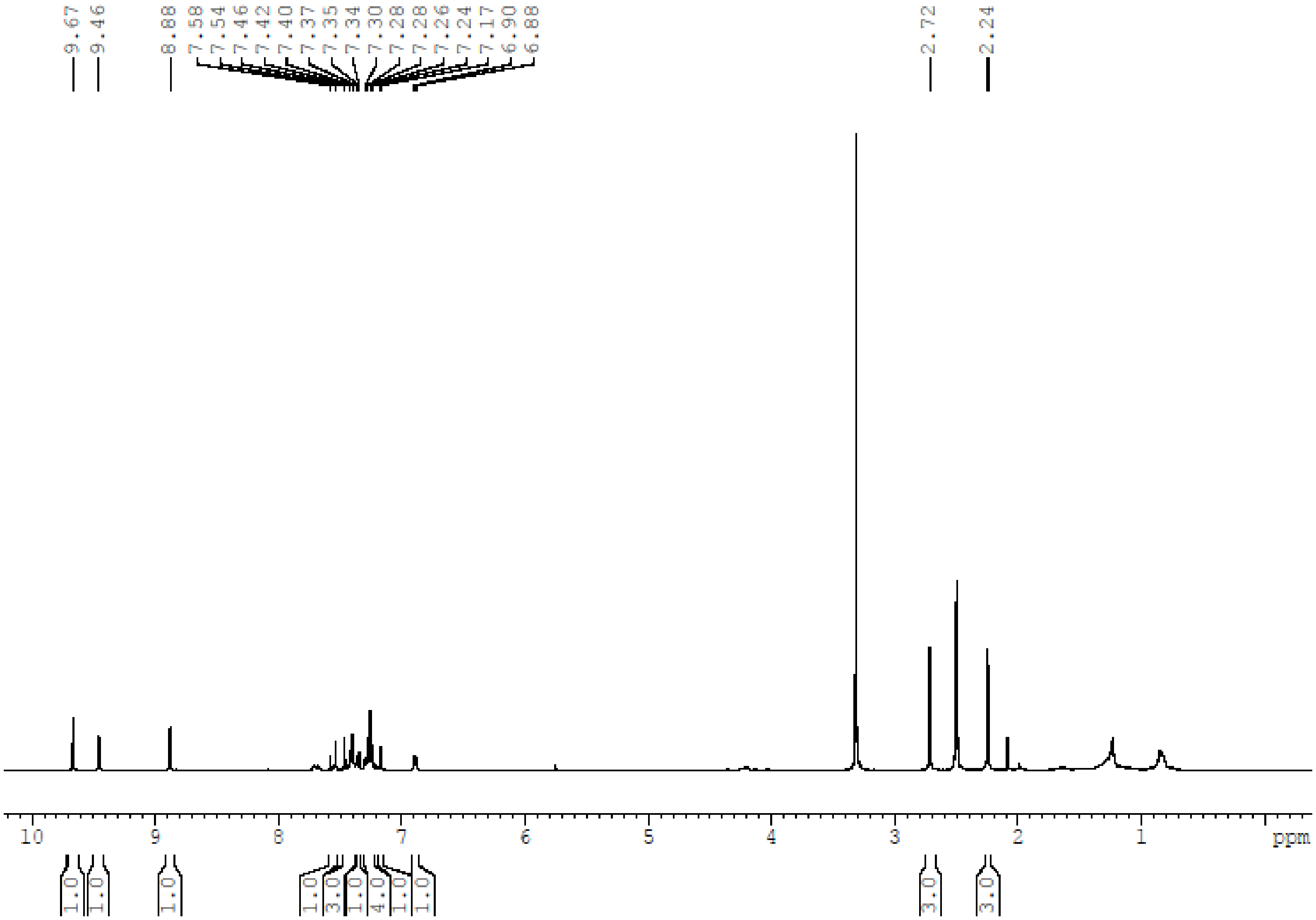

Figure S97: ^{1}H NMR spectrum of **17s** (400 MHz; DMSO-d_6).

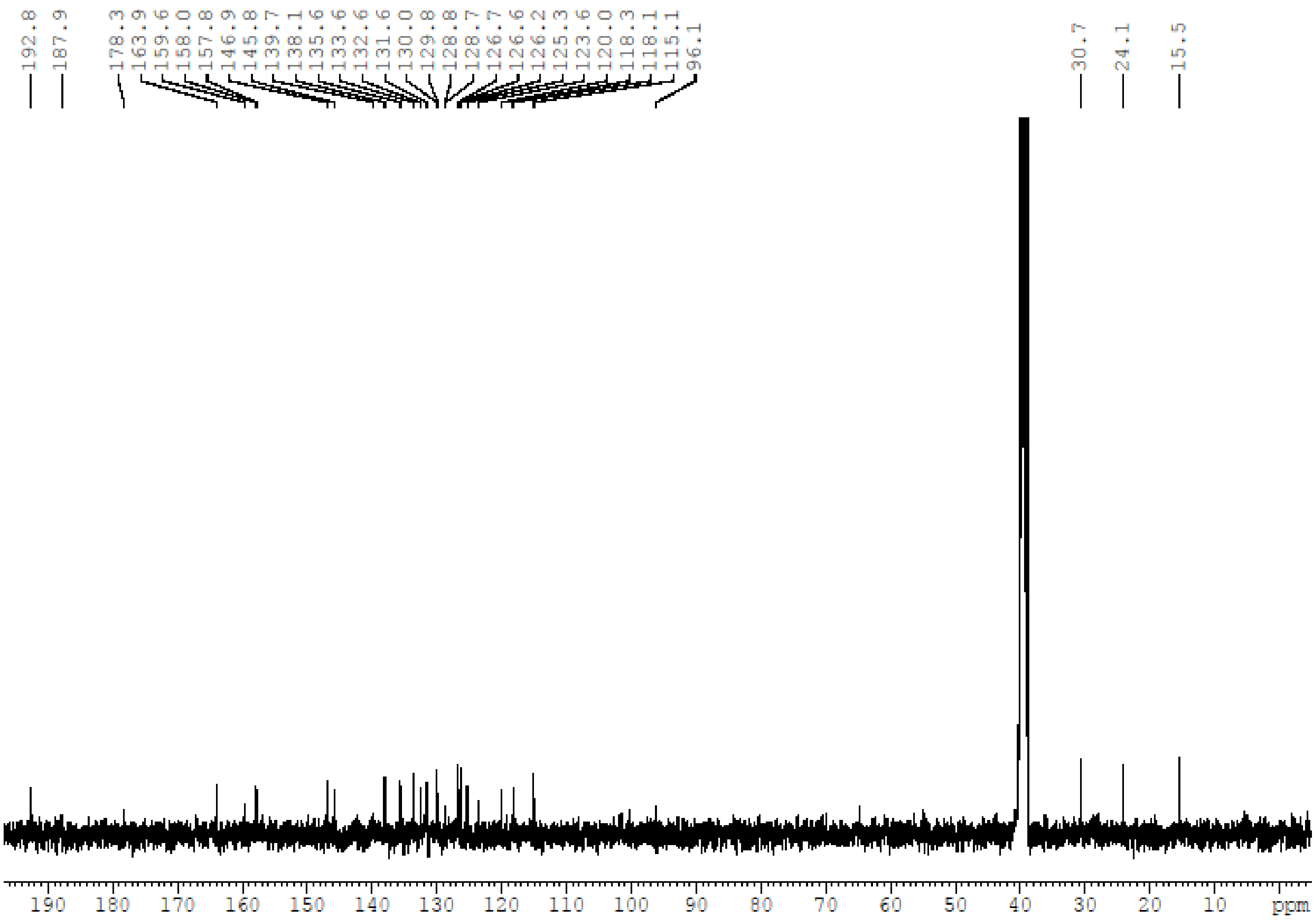

Figure S98: ^{13}C NMR spectrum of **17s** (100 MHz; DMSO-d_6).

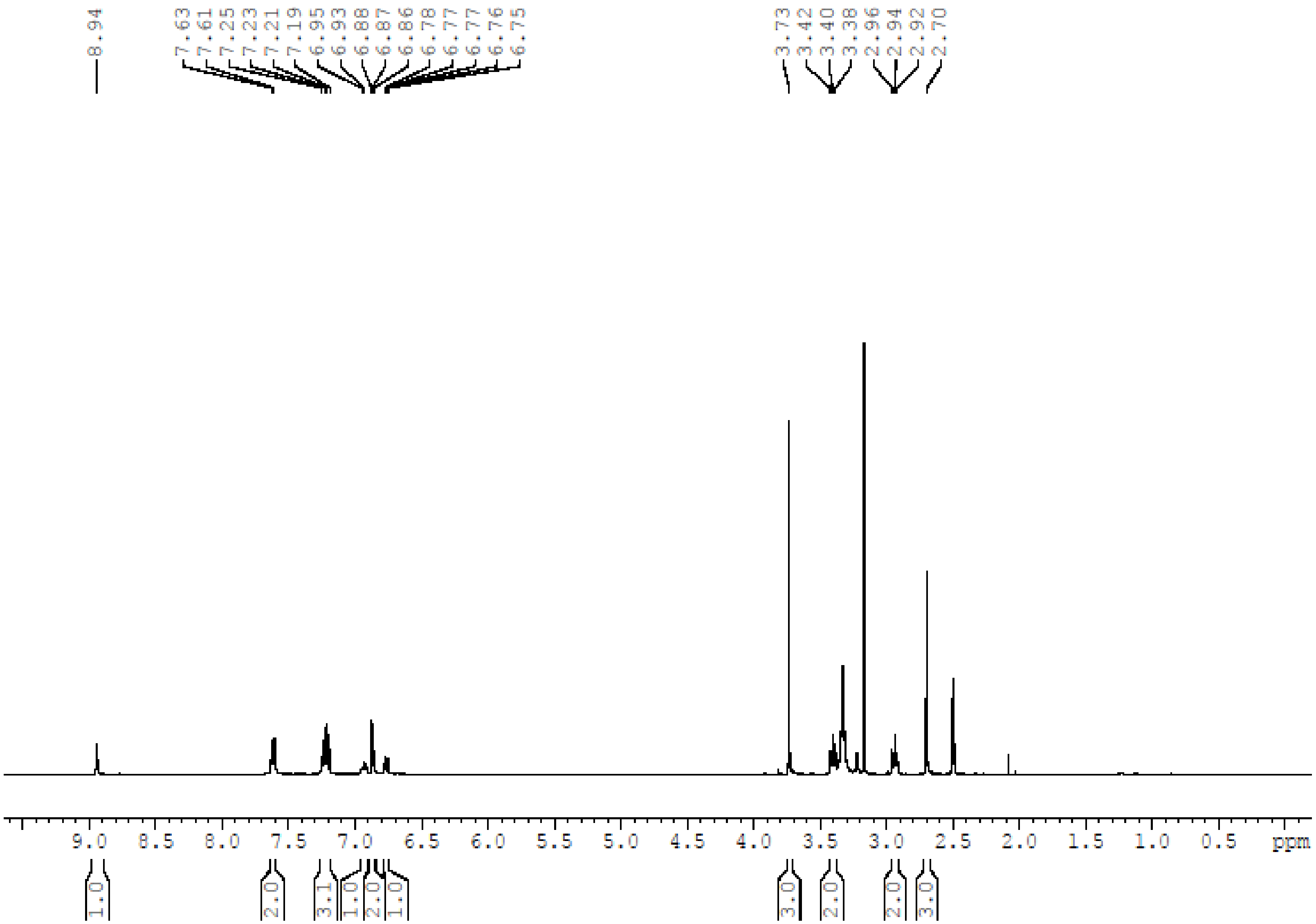

Figure S99: ^{1}H NMR spectrum of **18a** (400 MHz; DMSO-d_6).

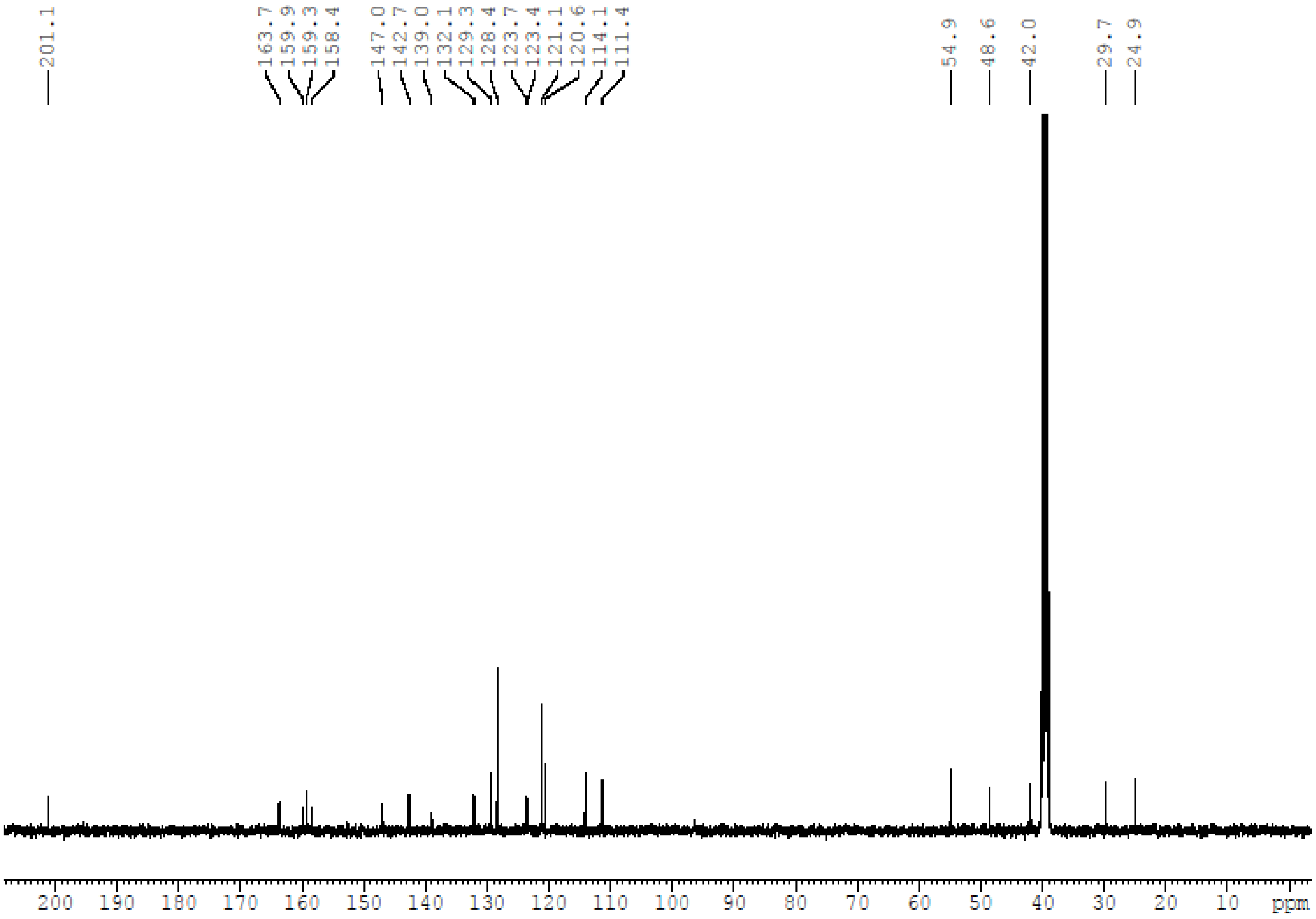

Figure S100: ^{13}C NMR spectrum of **18a** (100 MHz; DMSO-d_6).

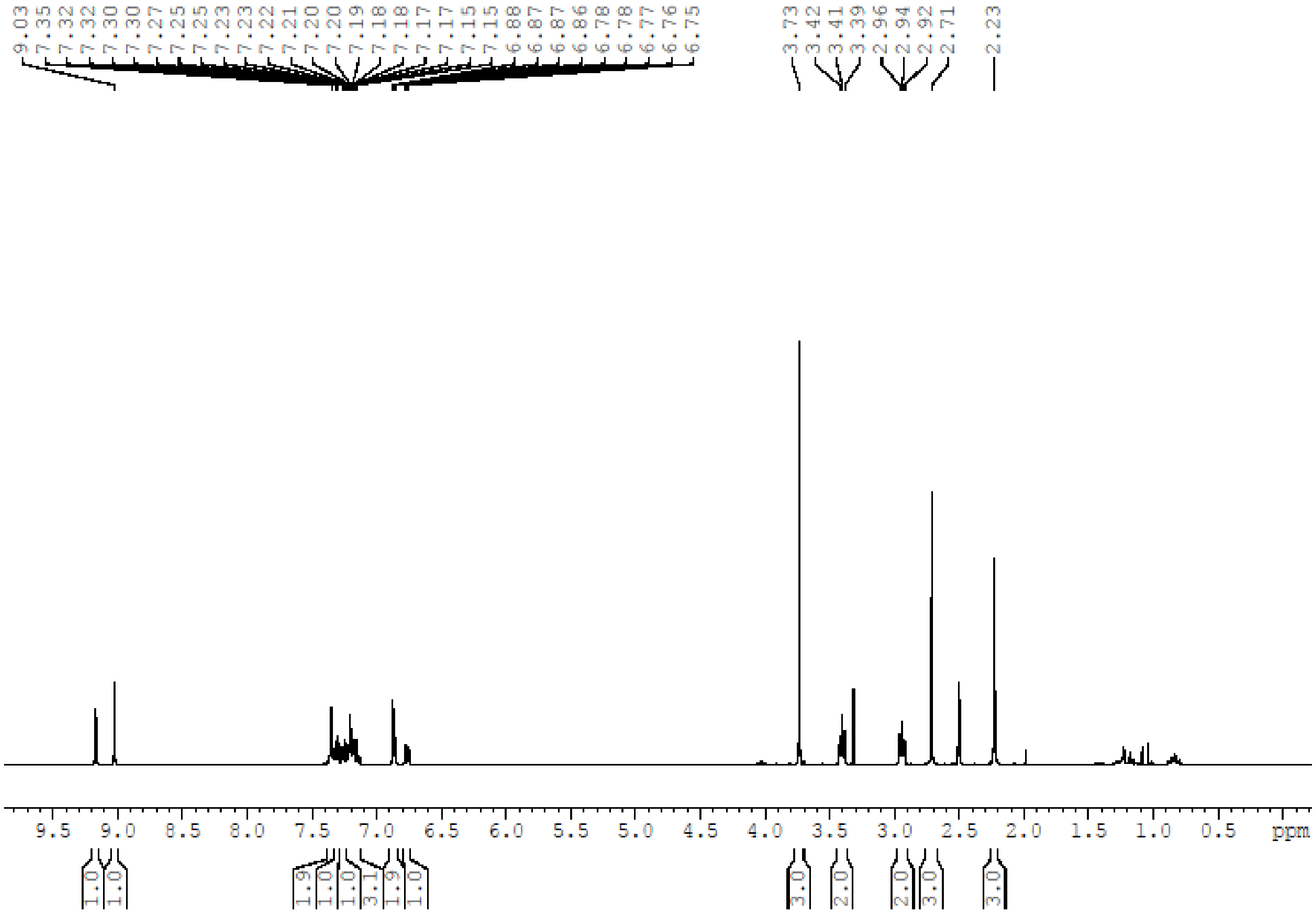

Figure S101: ^{1}H NMR spectrum of **18b** (400 MHz; DMSO-d_6).

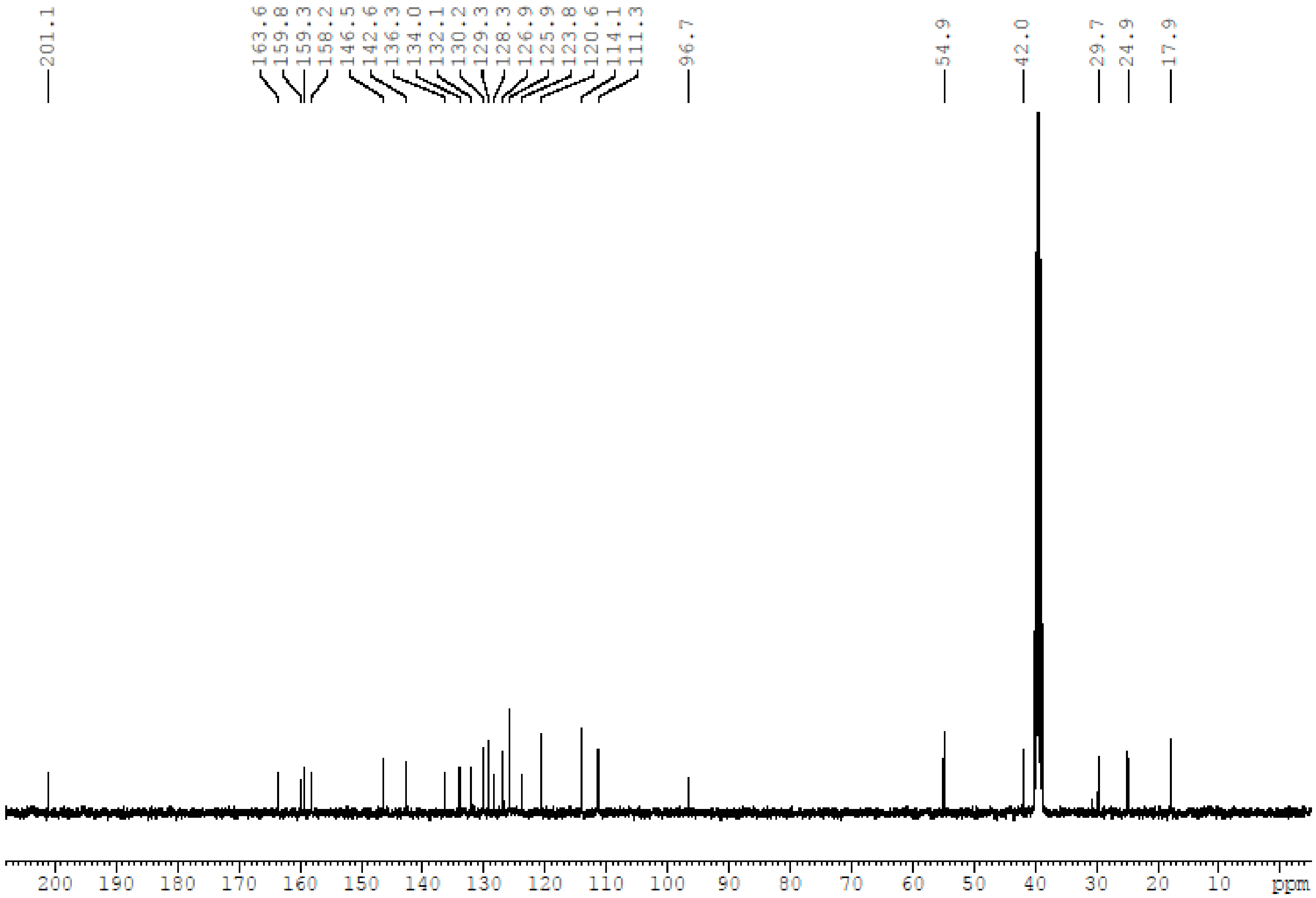

Figure S102: ^{13}C NMR spectrum of **18b** (100 MHz; DMSO-d_6).

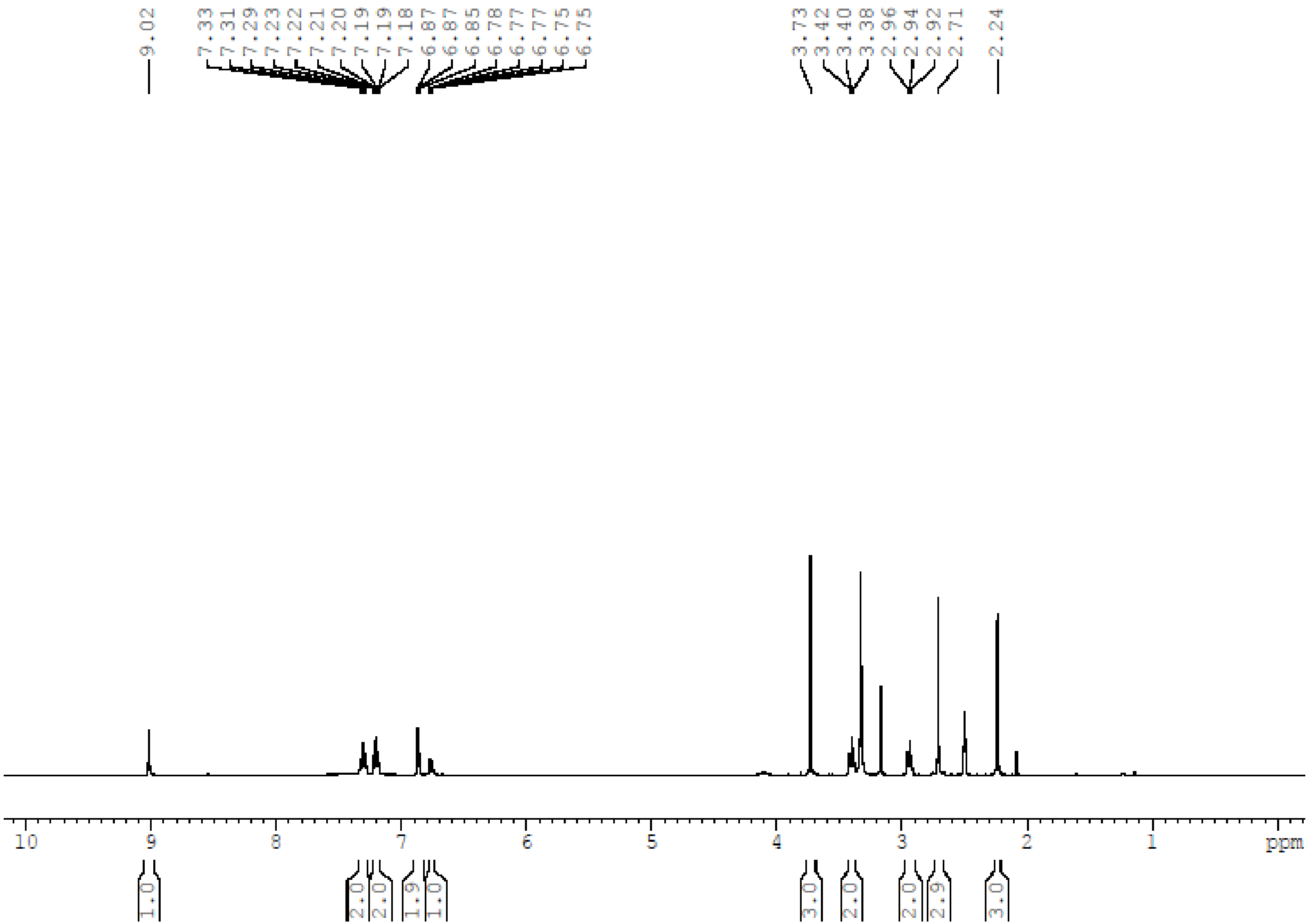

Figure S103: ^{1}H NMR spectrum of **18c** (400 MHz; DMSO-d_6).

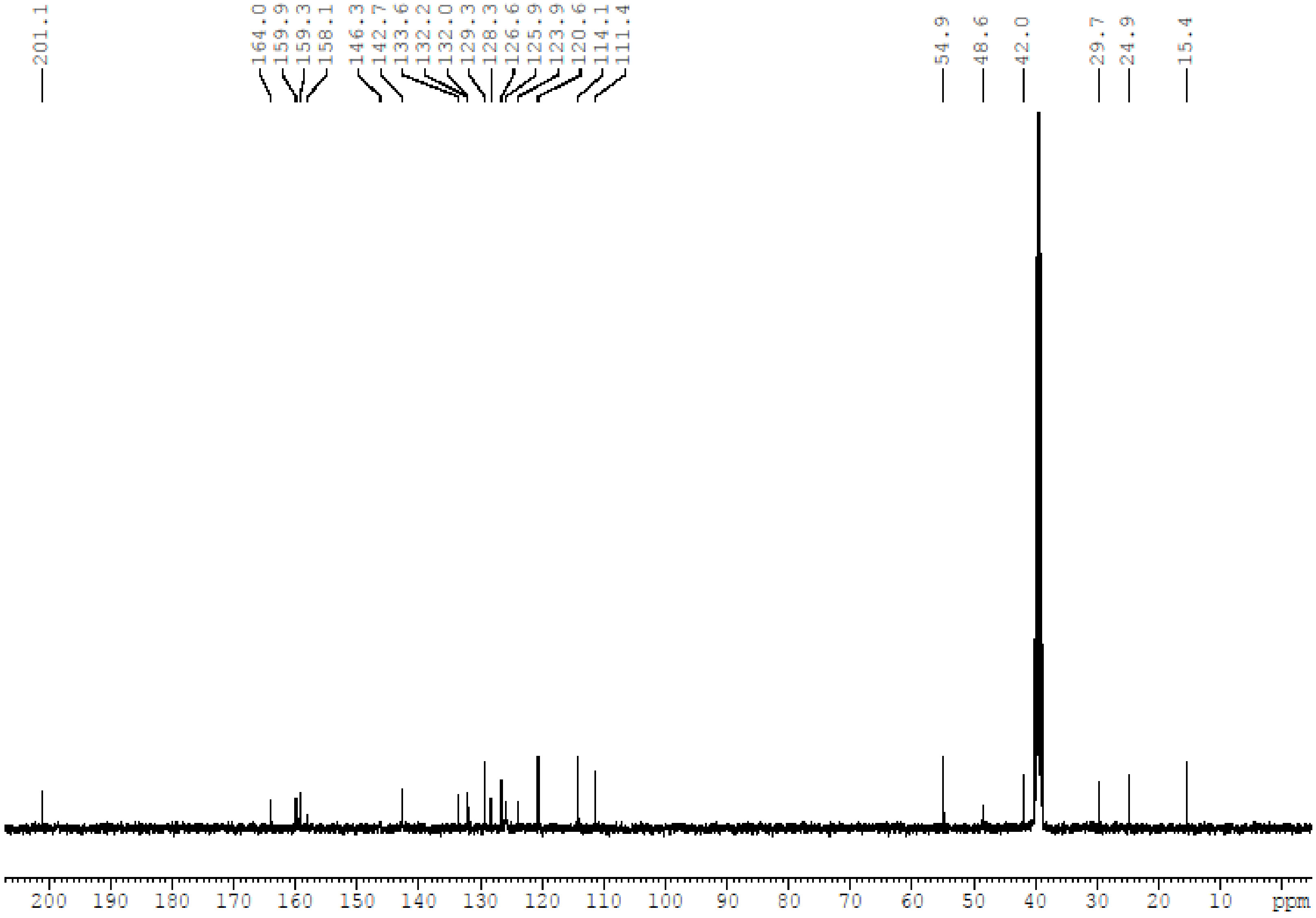

Figure S104: ^{13}C NMR spectrum of **18c** (100 MHz; DMSO-d_6).

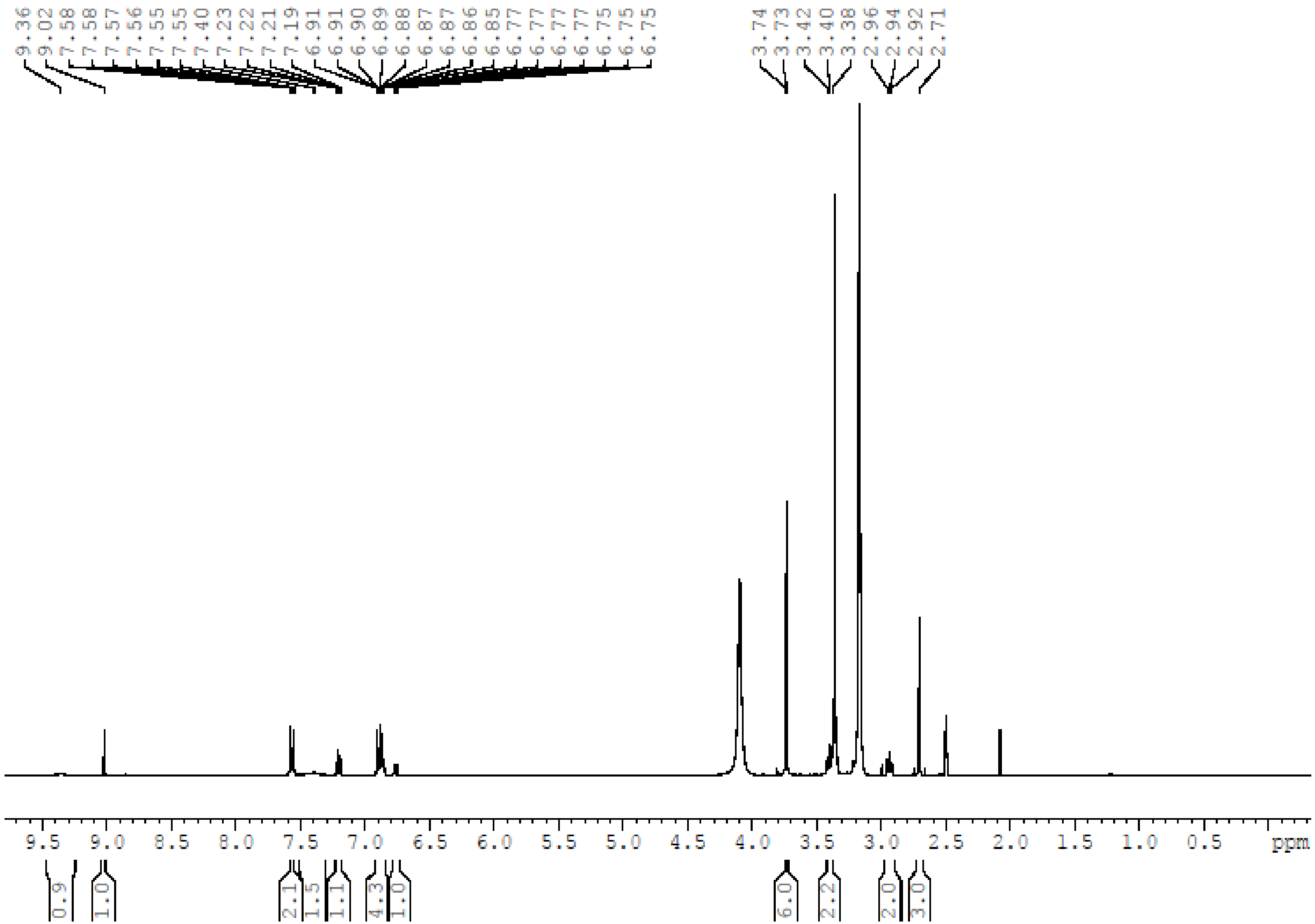

Figure S105: ^{1}H NMR spectrum of **18e** (400 MHz; DMSO-d_6).

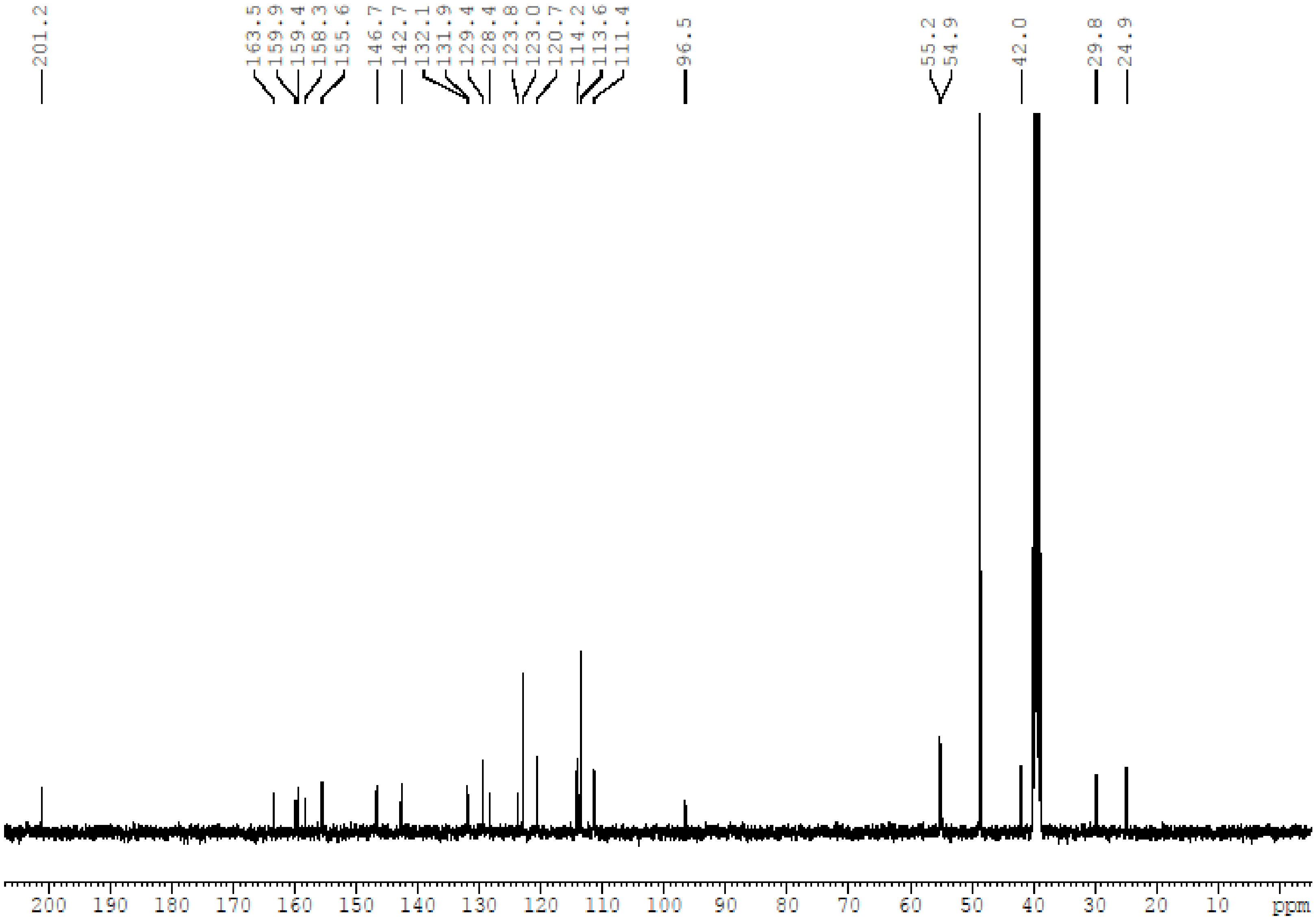

Figure S106: ^{13}C NMR spectrum of **18e** (100 MHz; DMSO-d_6).

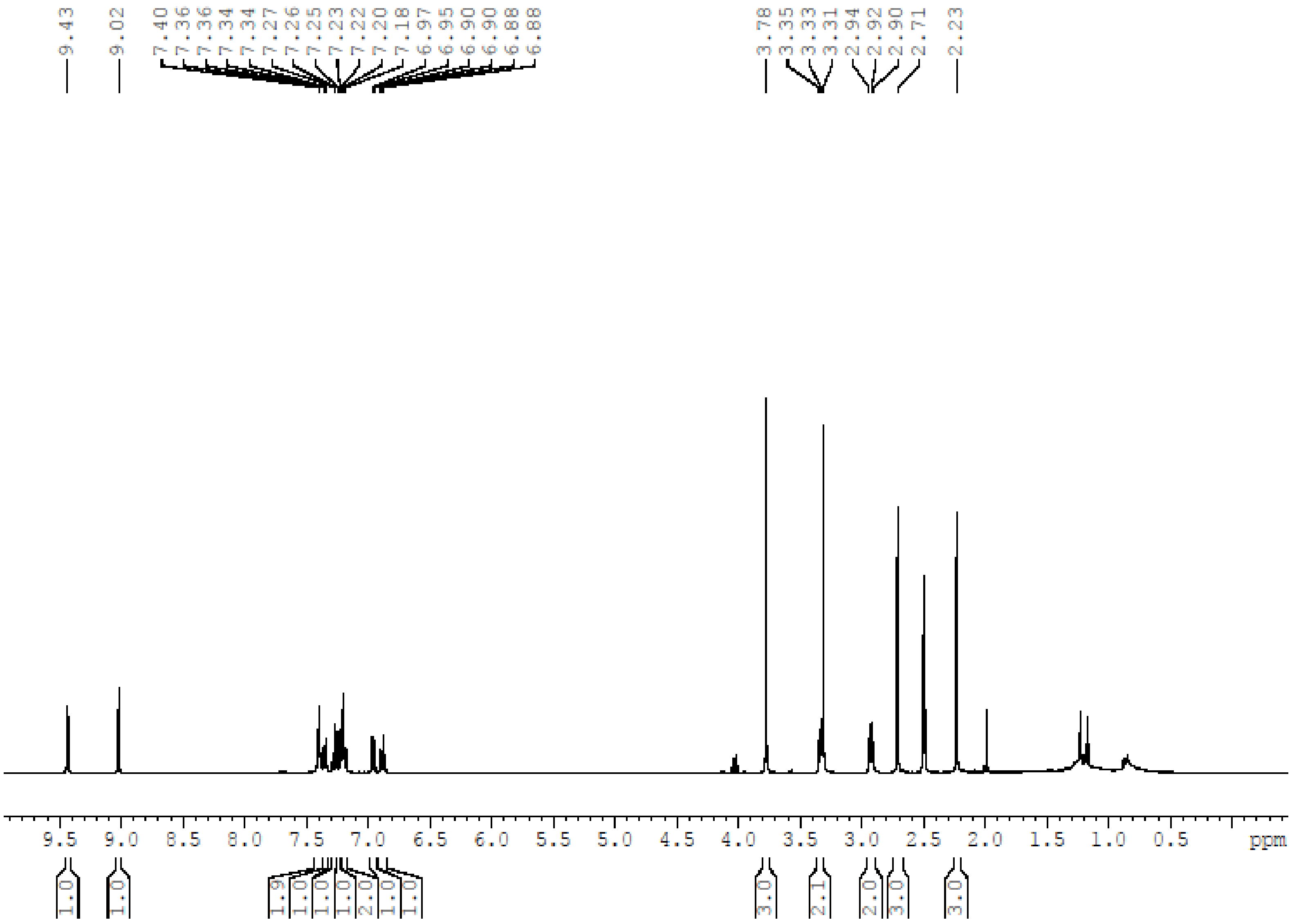

Figure S107: ^{1}H NMR spectrum of **19f** (400 MHz; DMSO-d_6).

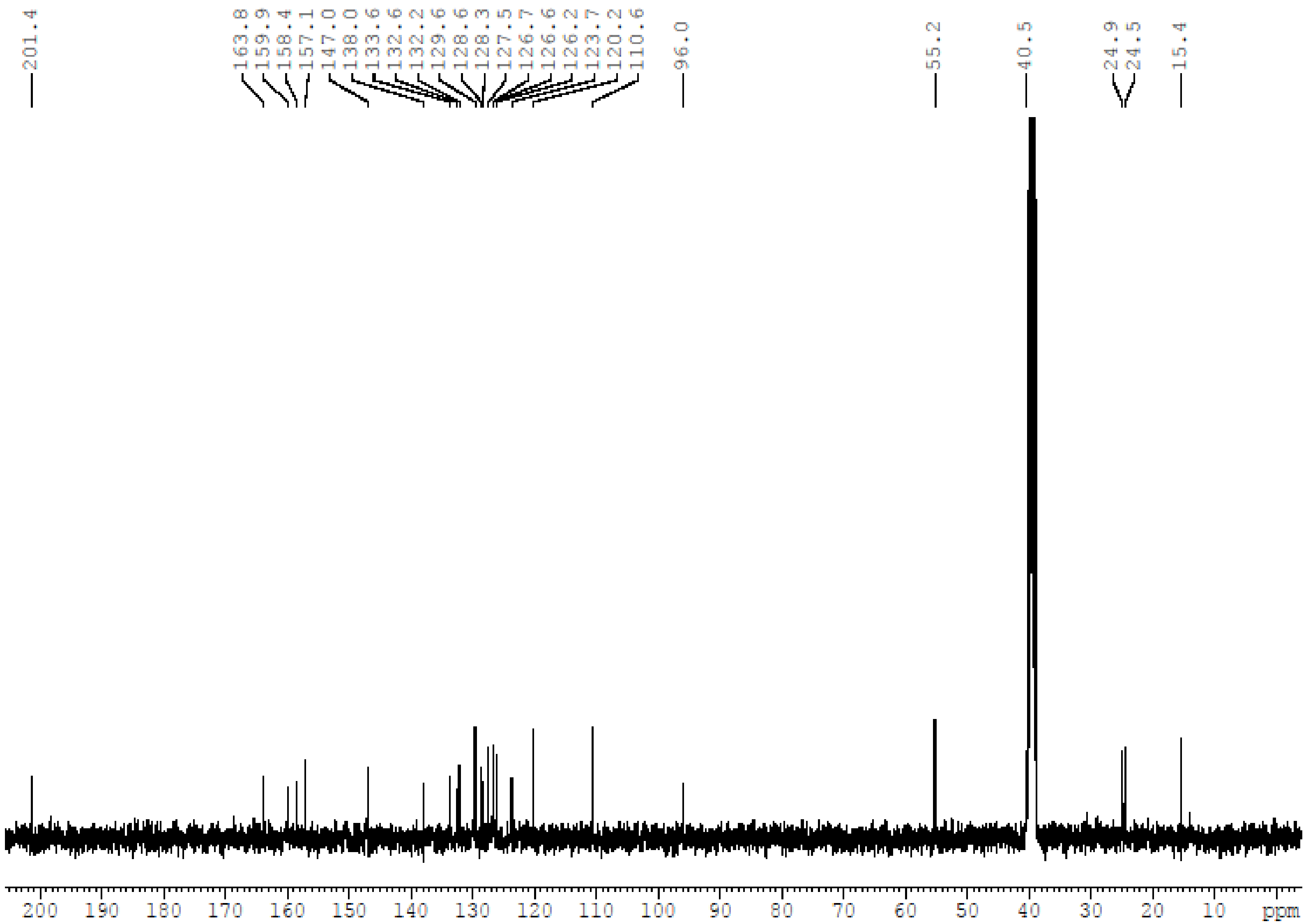

Figure S108: ^{13}C NMR spectrum of **19f** (100 MHz; DMSO-d_6).

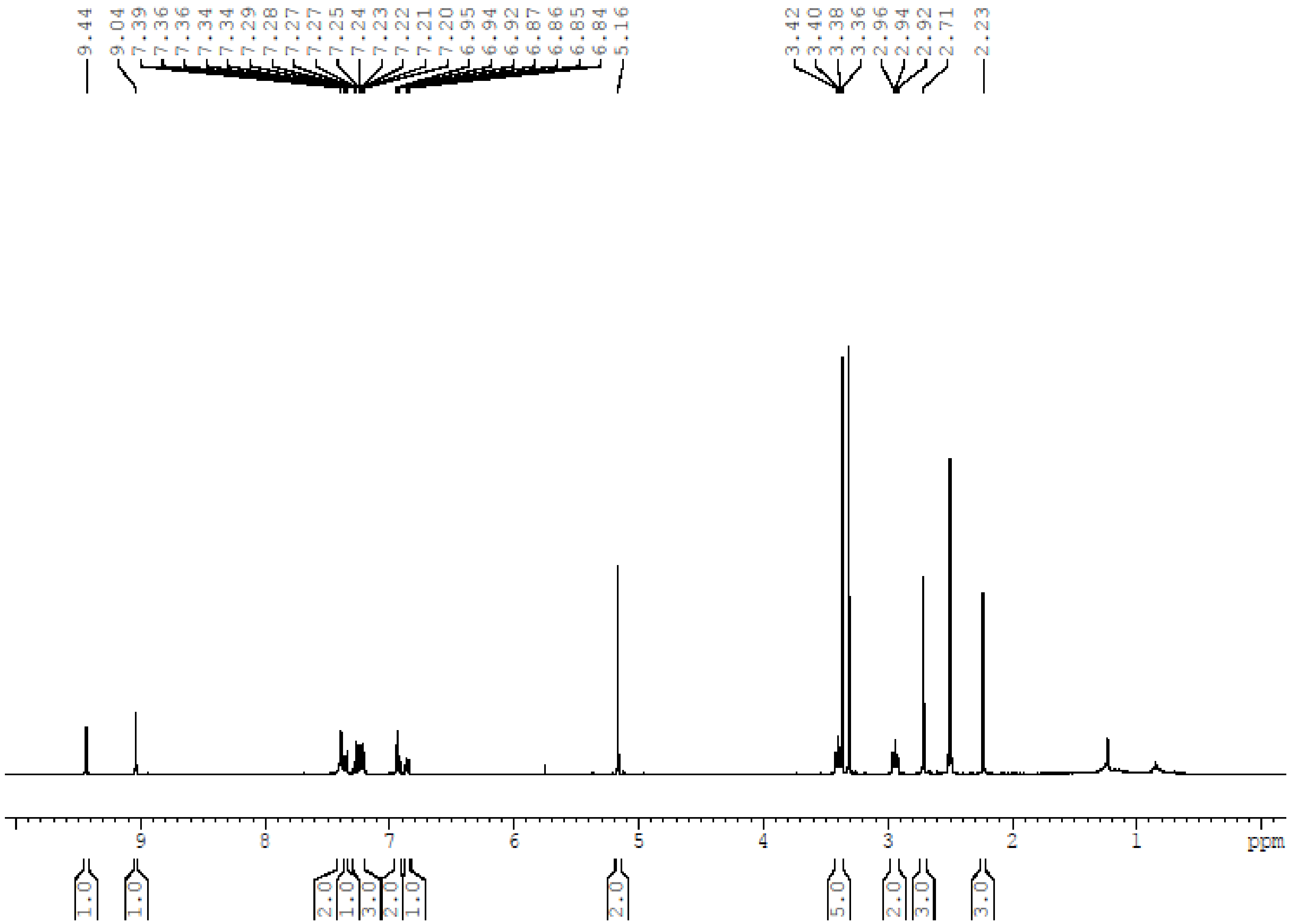

Figure S109: ^{1}H NMR spectrum of **19g** (400 MHz; DMSO-d_6).

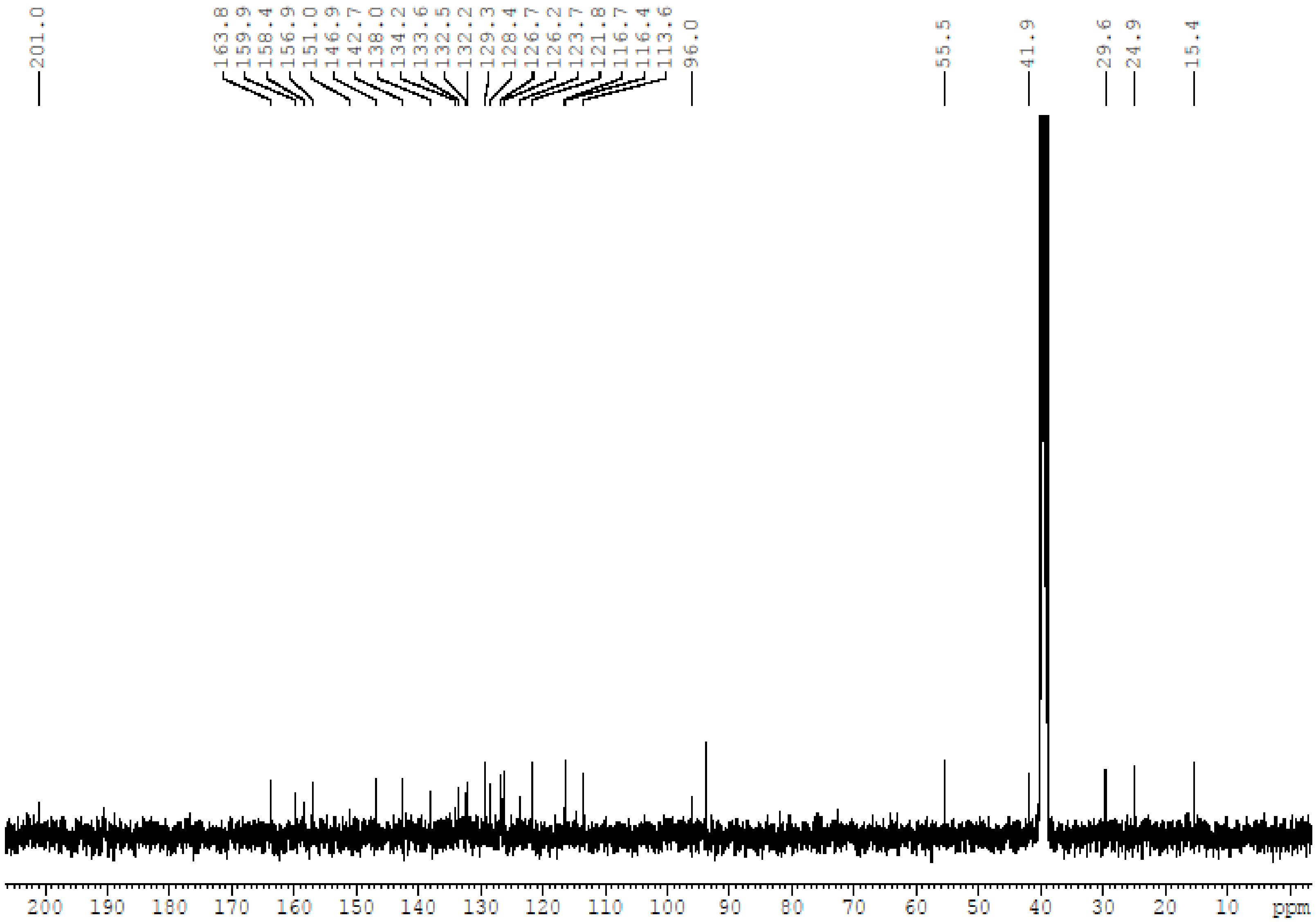

Figure S110: ^{13}C NMR spectrum of **19g** (100 MHz; DMSO-d_6).

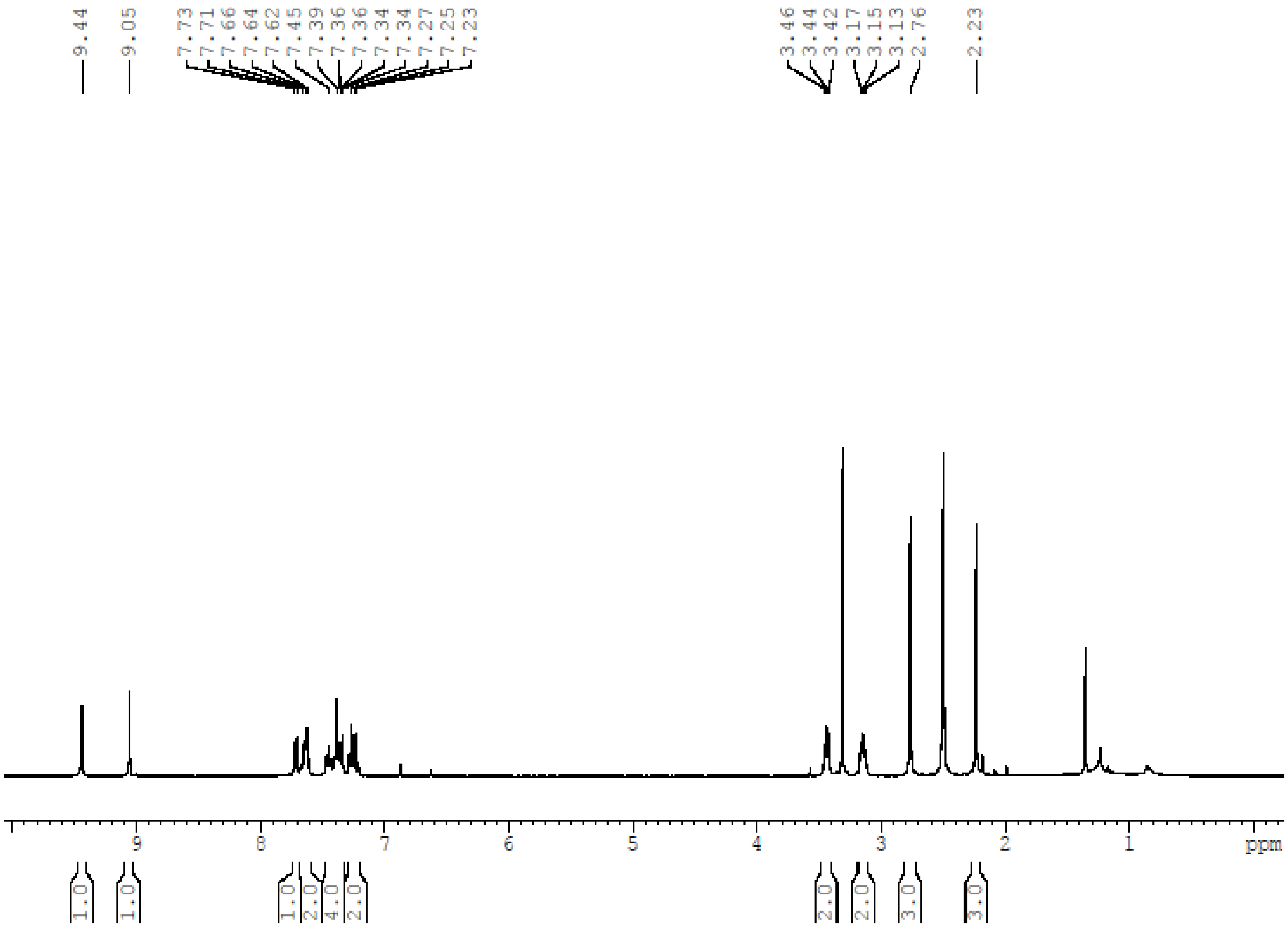

Figure S111: ^{1}H NMR spectrum of **19h** (400 MHz; DMSO-d_6).

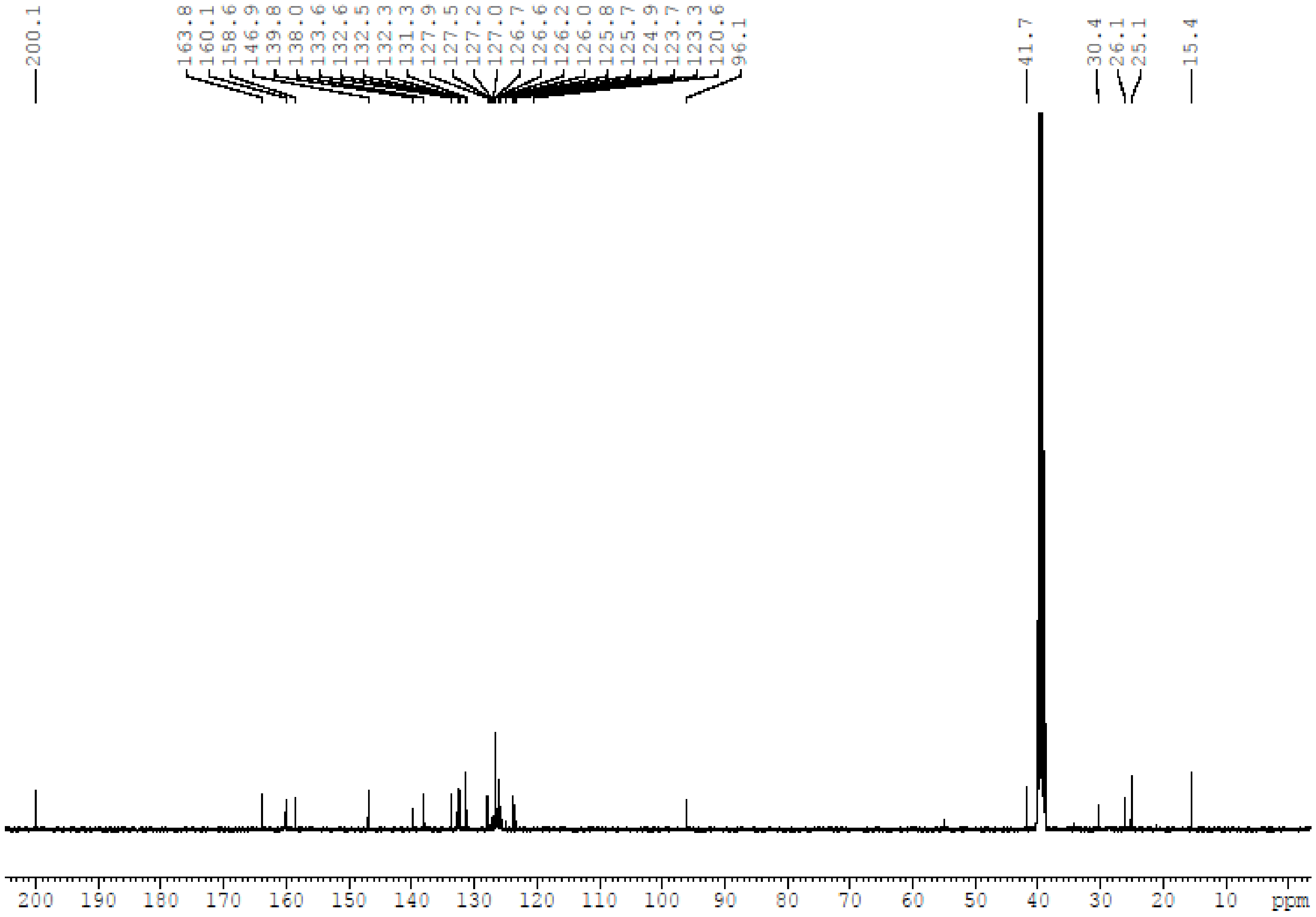

Figure S112: ^{13}C NMR spectrum of **19h** (100 MHz; DMSO-d_6).

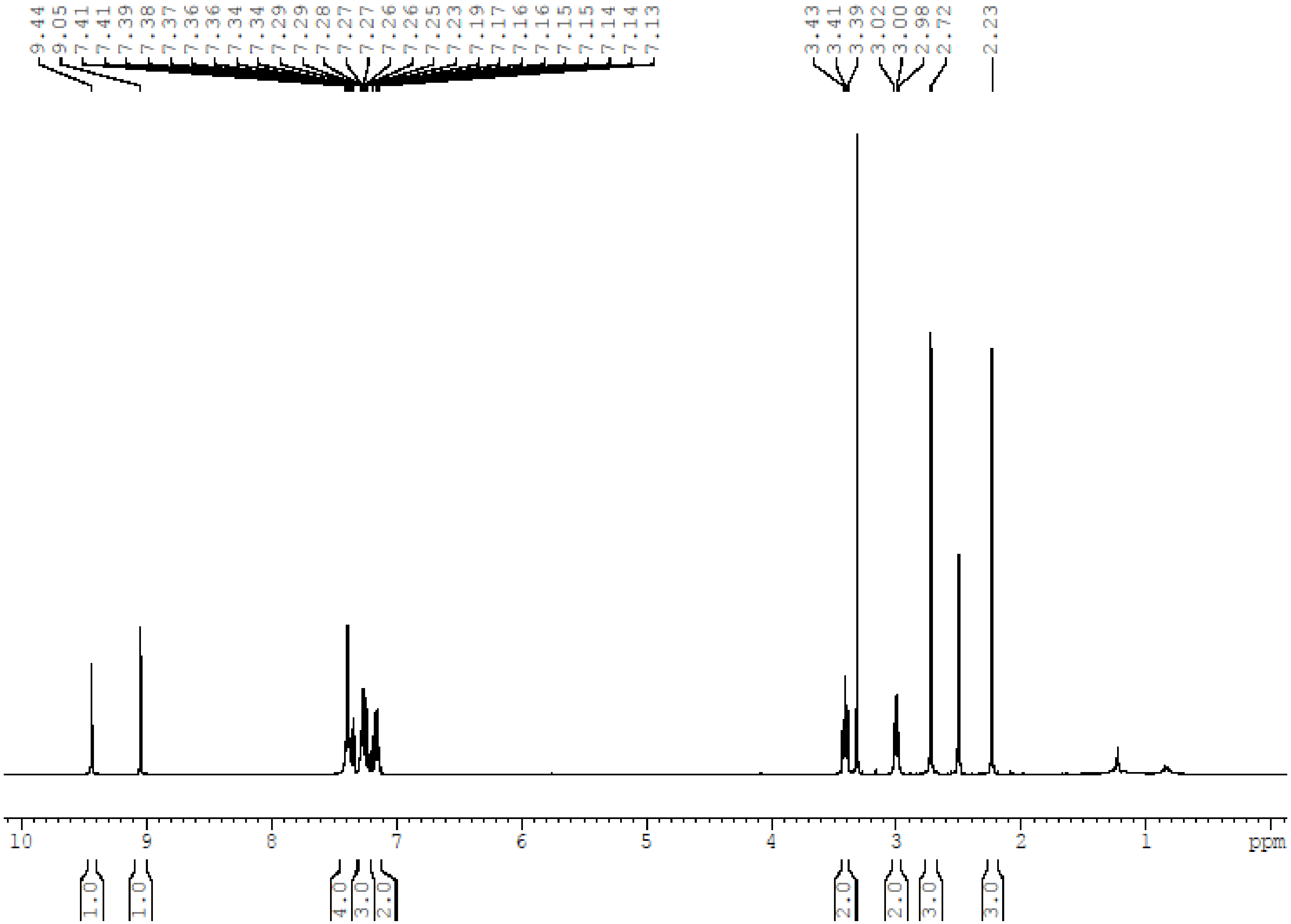

Figure S113: ^{1}H NMR spectrum of **19i** (400 MHz; DMSO-d_6).

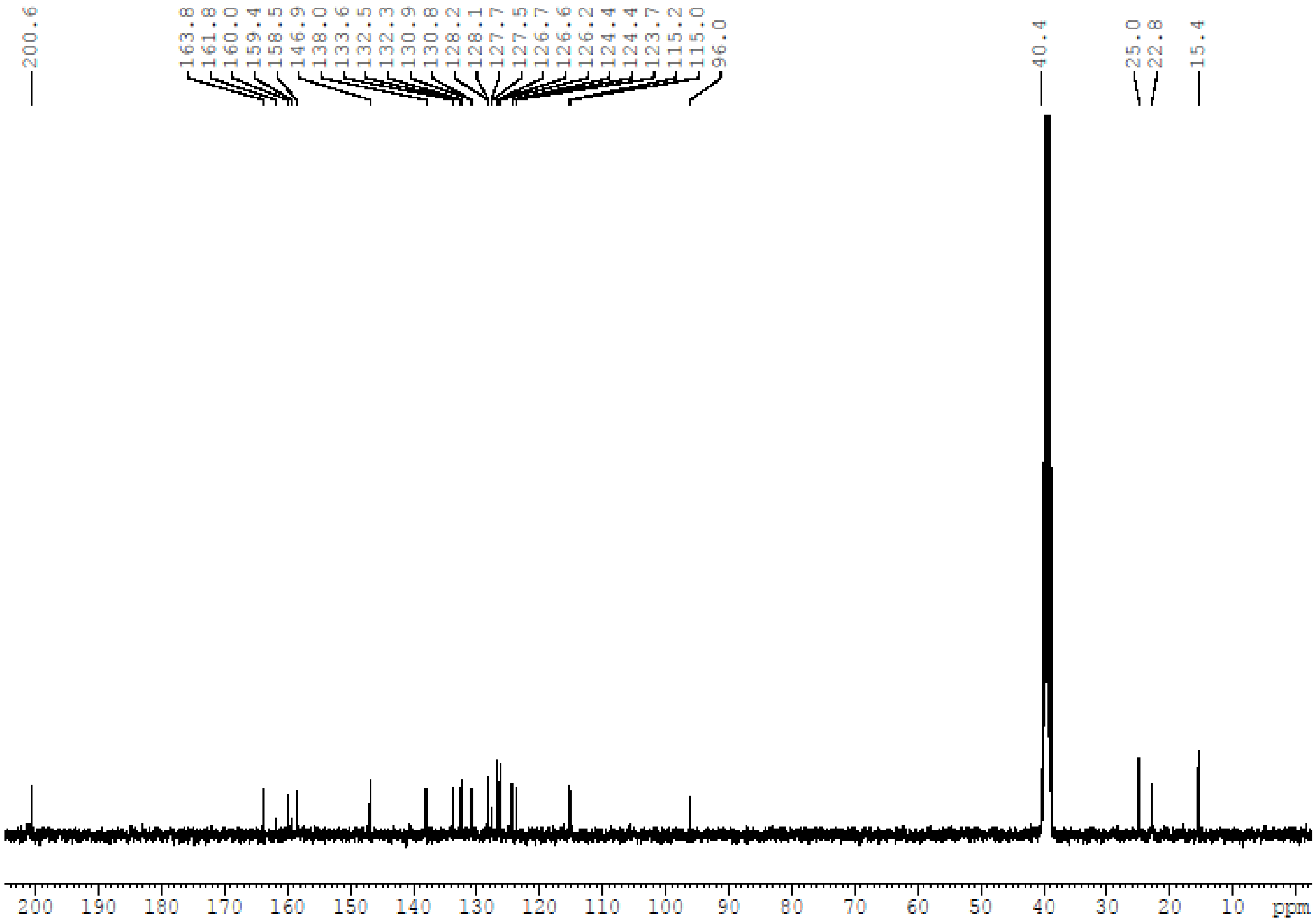

Figure S114: ^{13}C NMR spectrum of **19i** (100 MHz; DMSO-d_6).

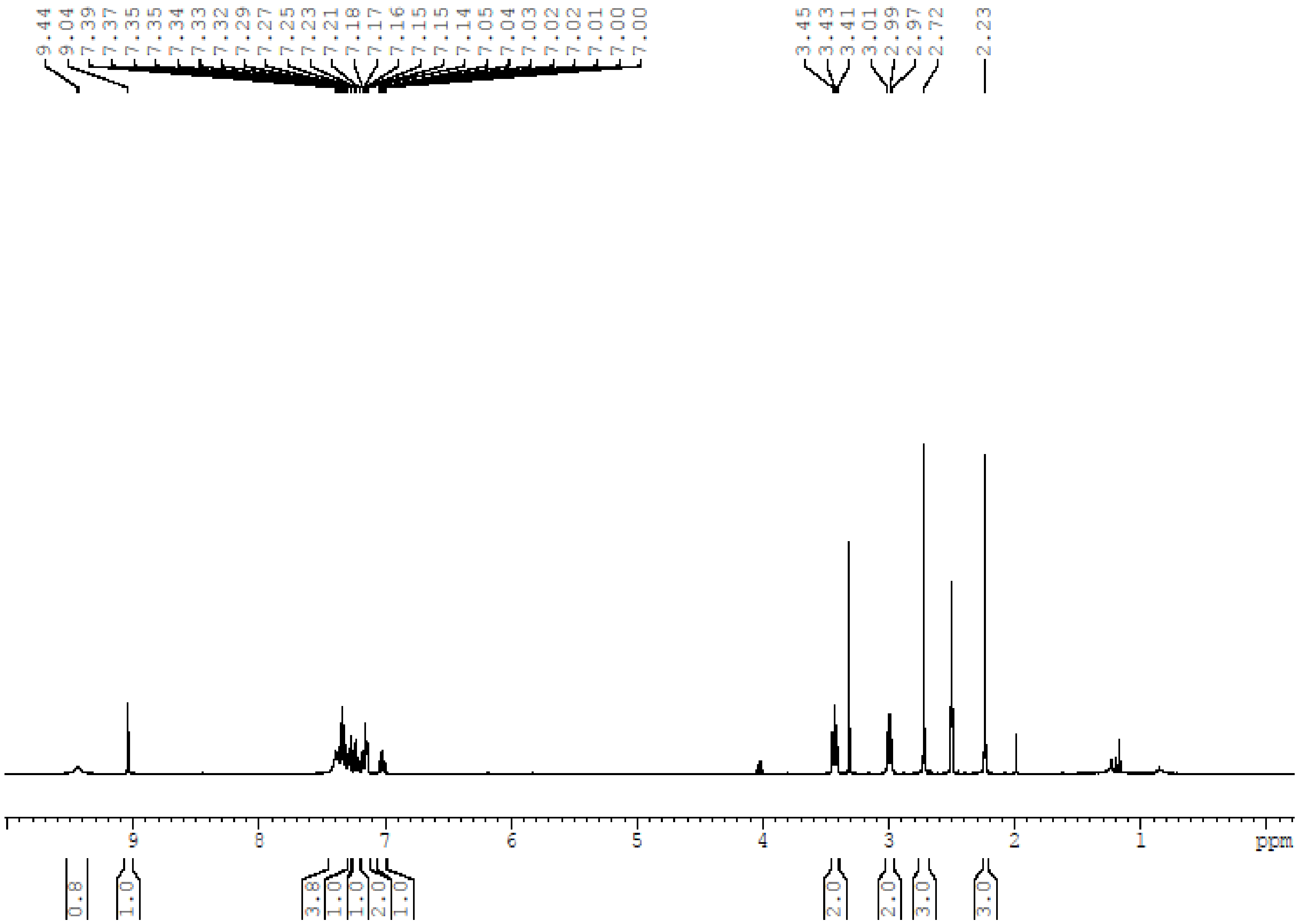

Figure S115: ^{1}H NMR spectrum of **19j** (400 MHz; DMSO-d_6).

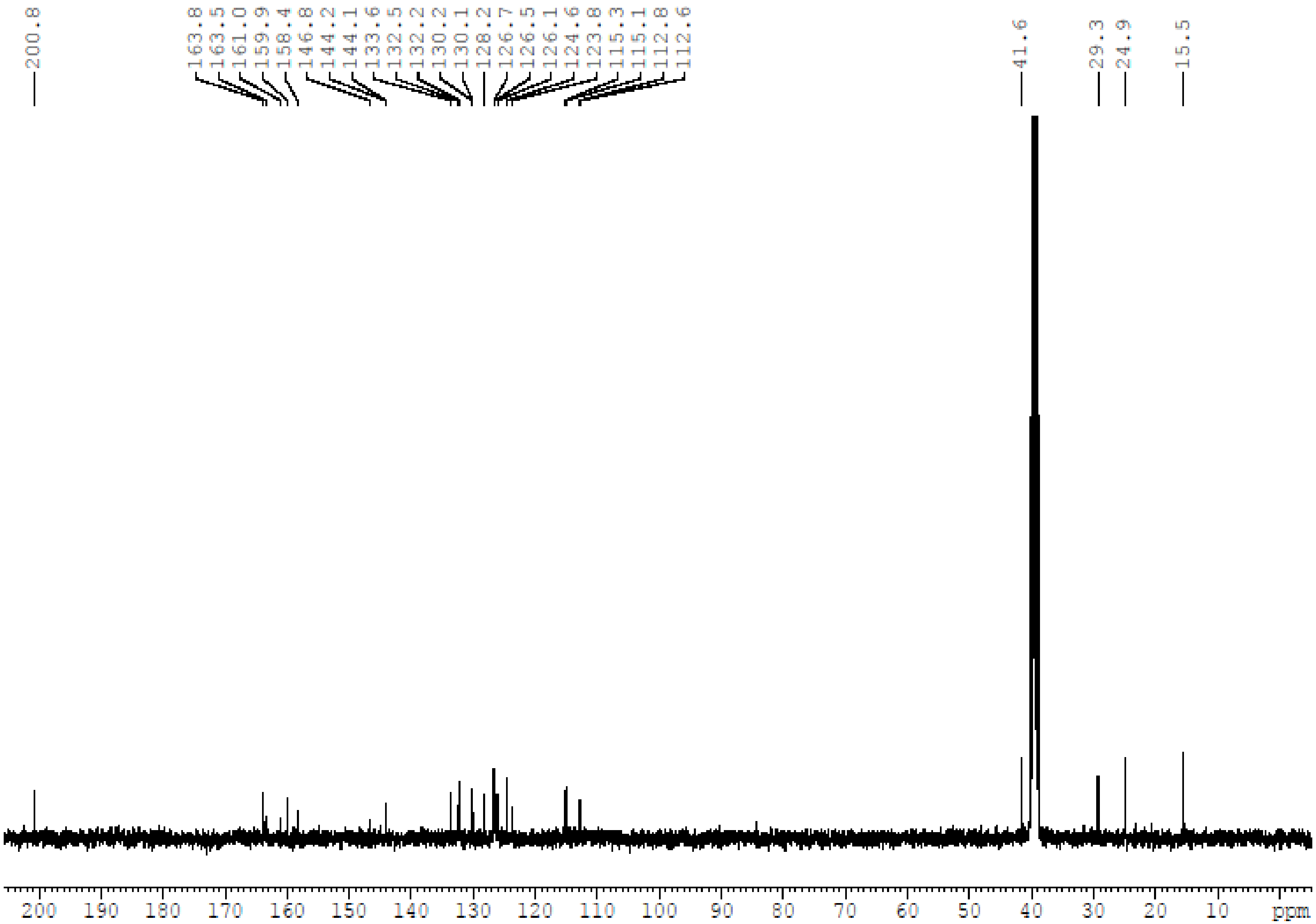

Figure S116: ^{13}C NMR spectrum of **19j** (100 MHz; DMSO-d_6).

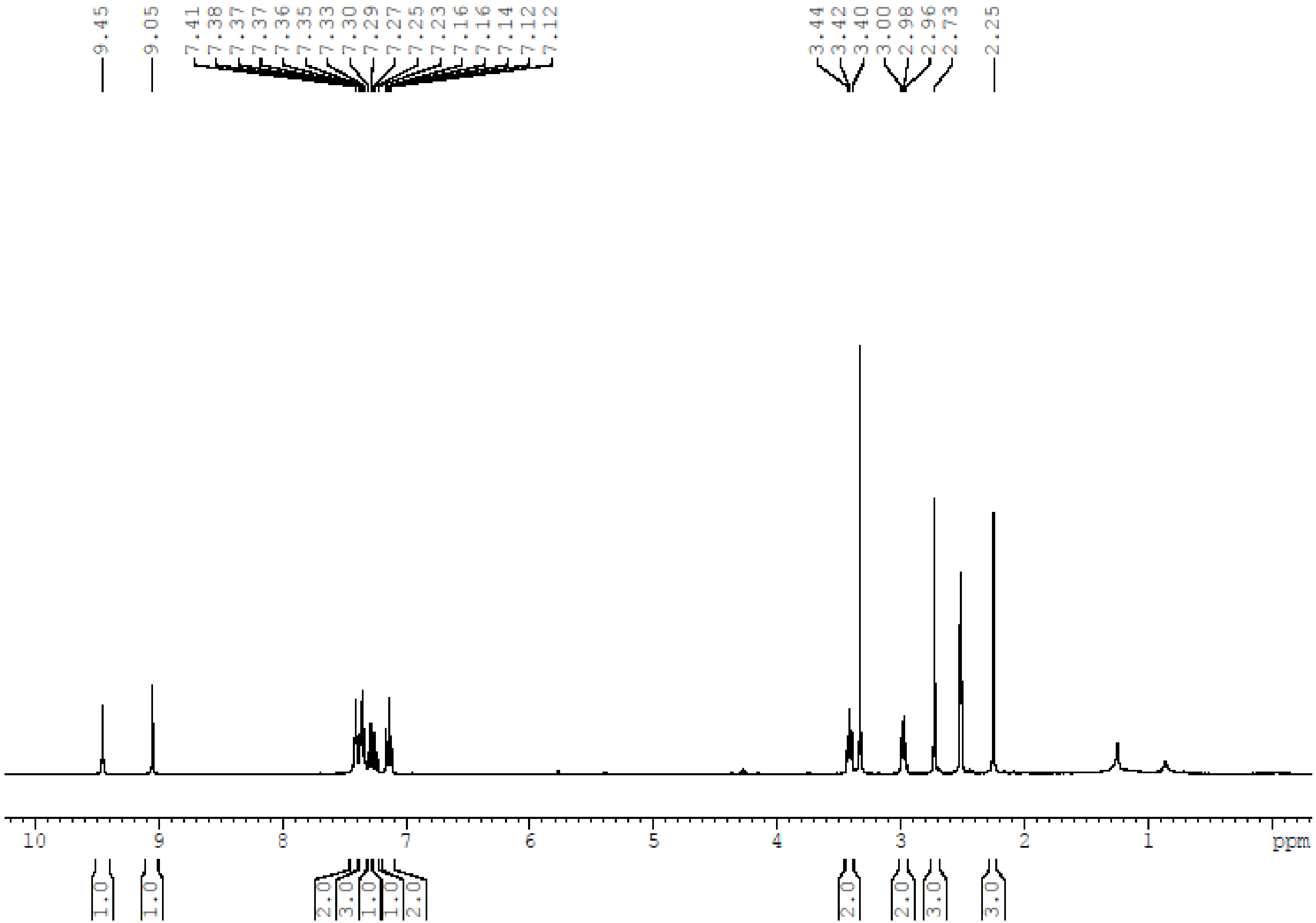

Figure S117: ^{1}H NMR spectrum of **19k** (400 MHz; DMSO-d_6).

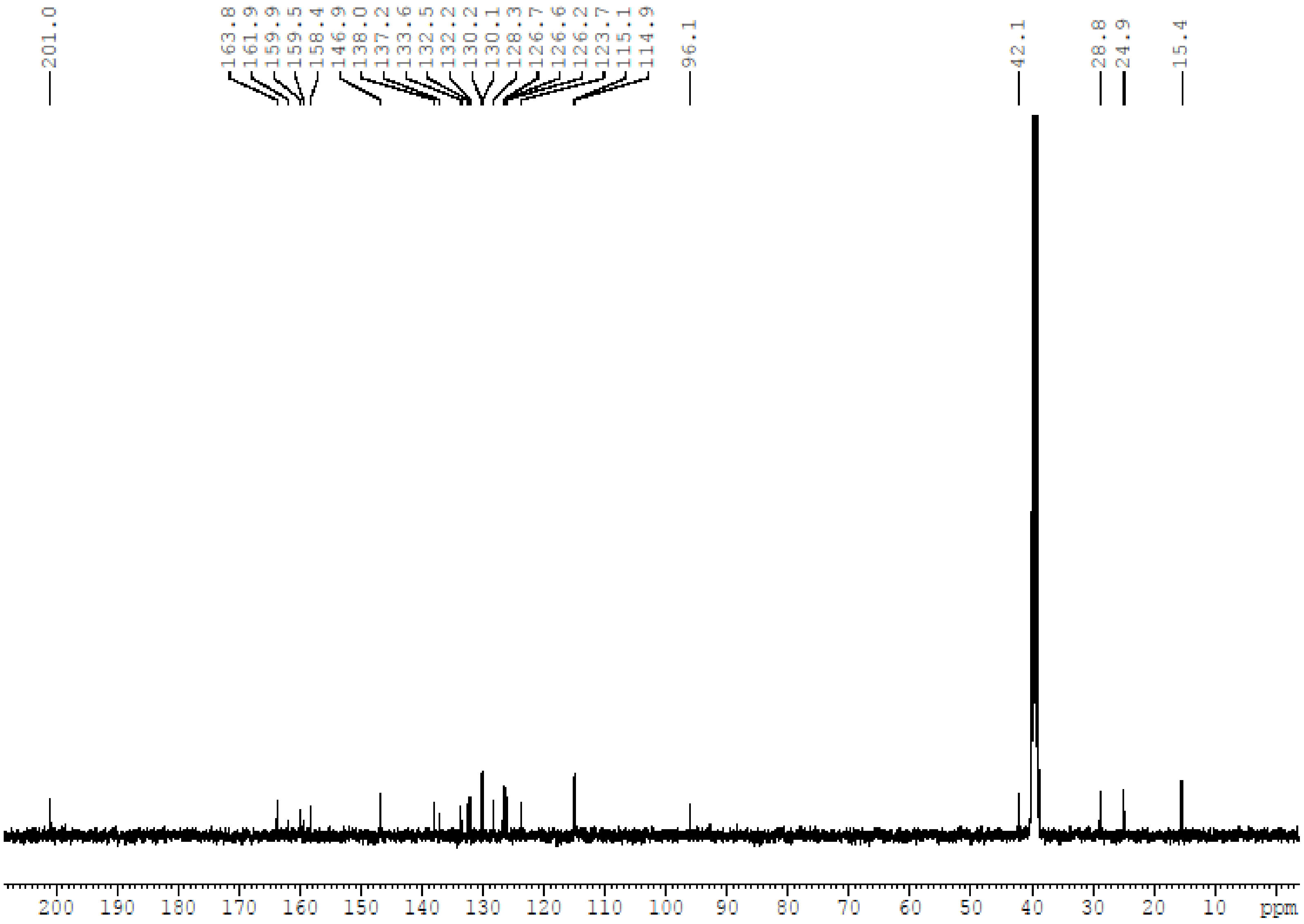

Figure S118: ^{13}C NMR spectrum of **19k** (100 MHz; DMSO-d_6).

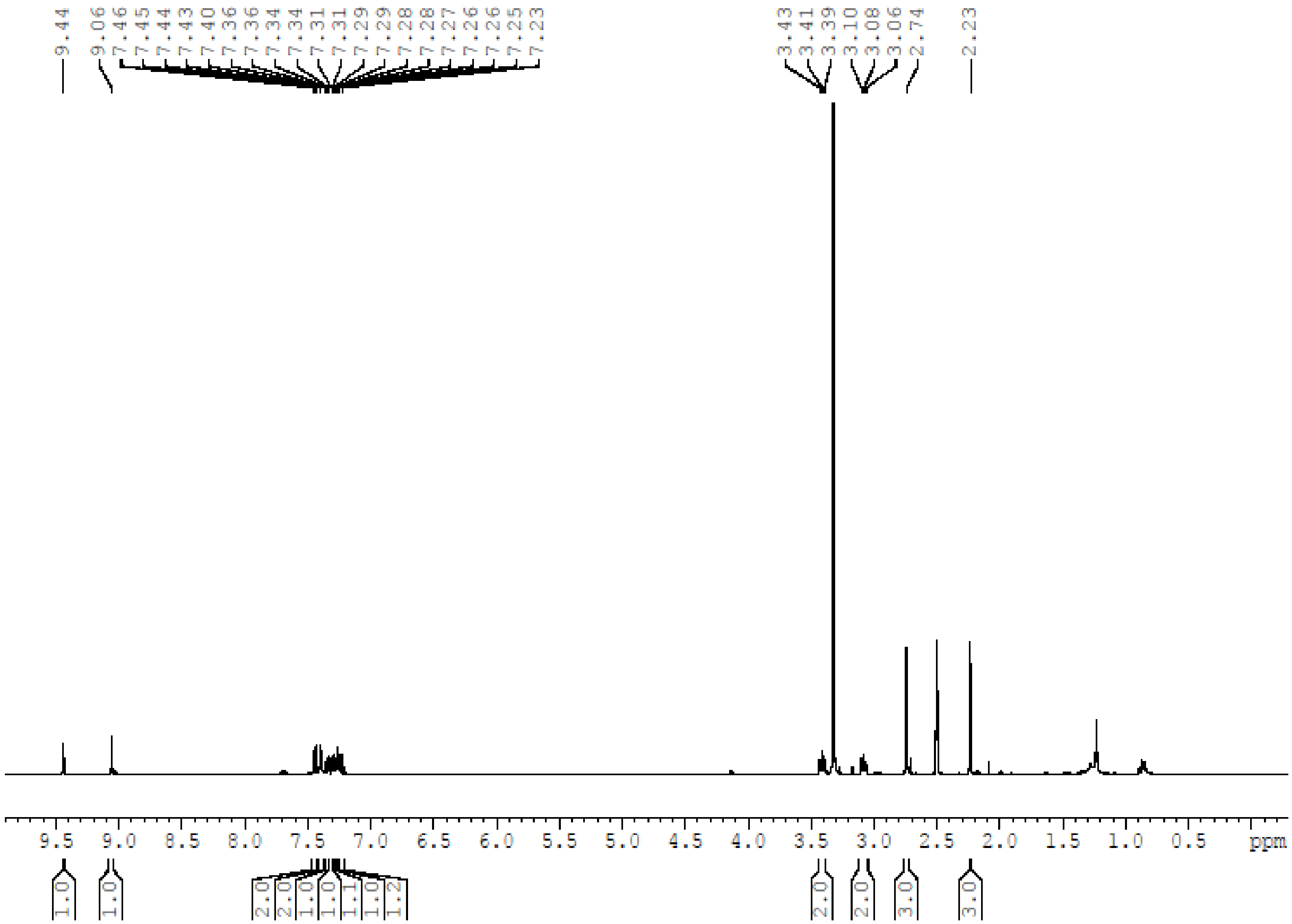

Figure S119: ^{1}H NMR spectrum of **19l** (400 MHz; DMSO-d_6).

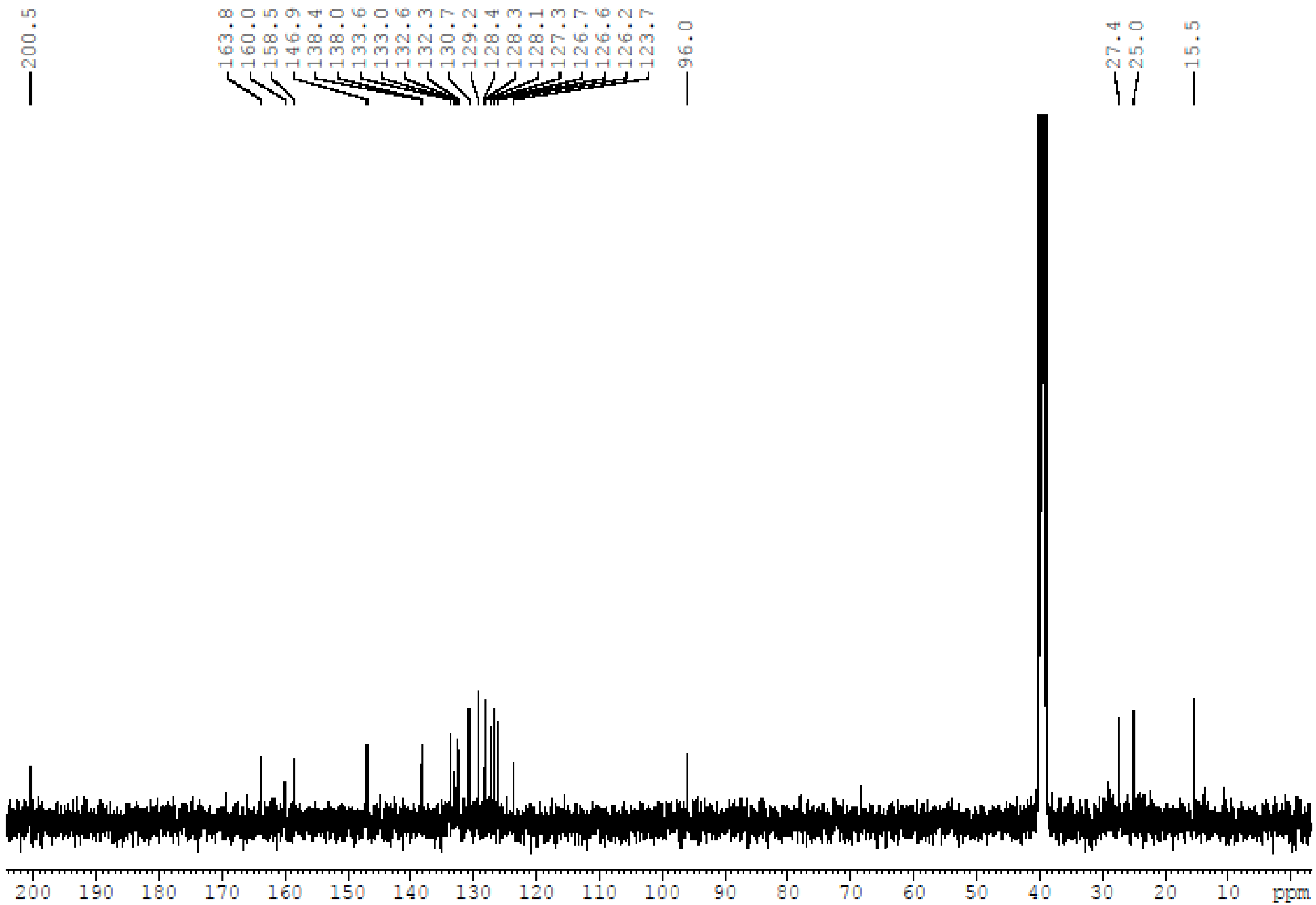

Figure S120: ^{13}C NMR spectrum of **19l** (100 MHz; DMSO-d_6).

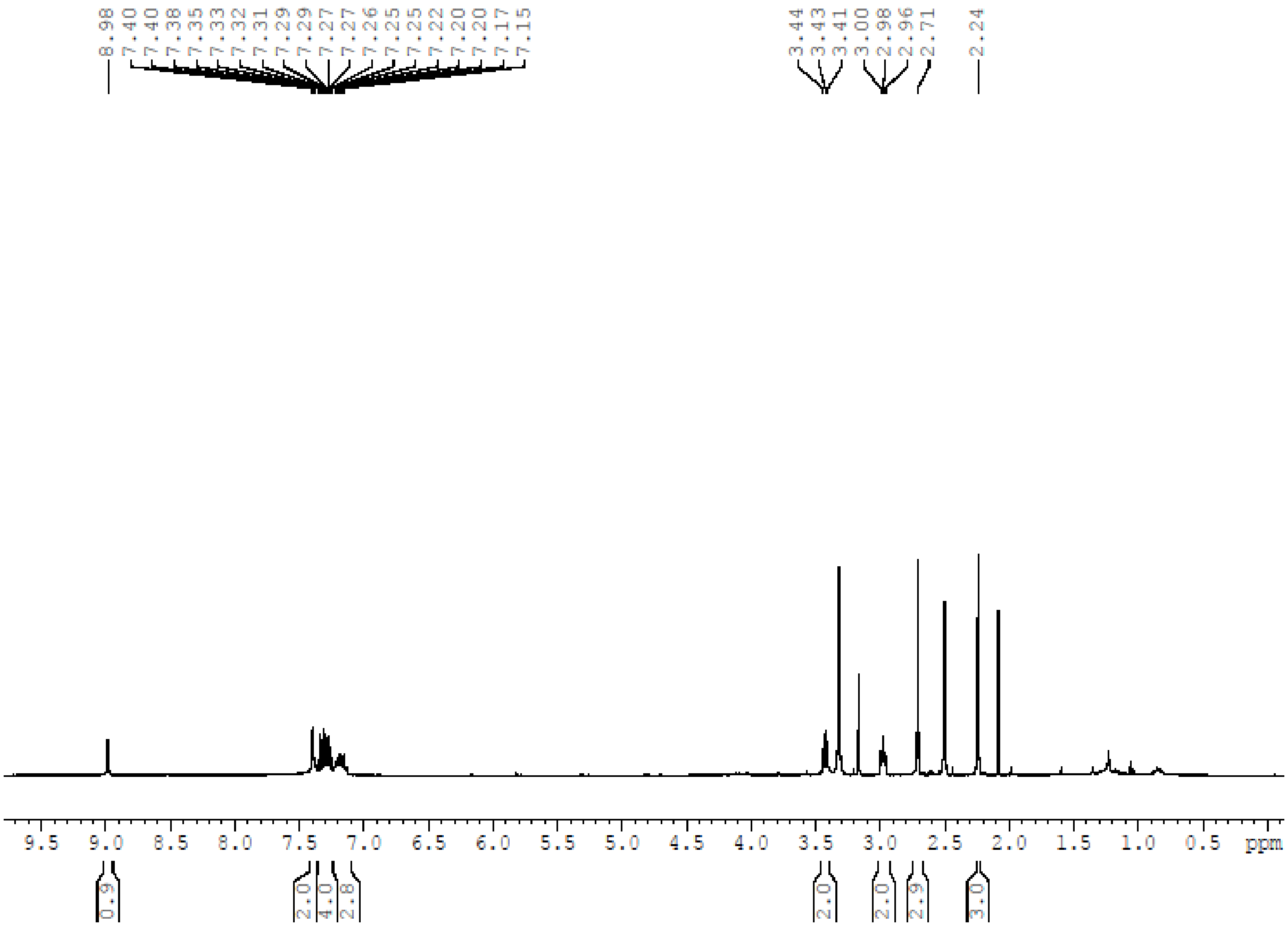

Figure S121: ^{1}H NMR spectrum of **19m** (400 MHz; DMSO-d_6).

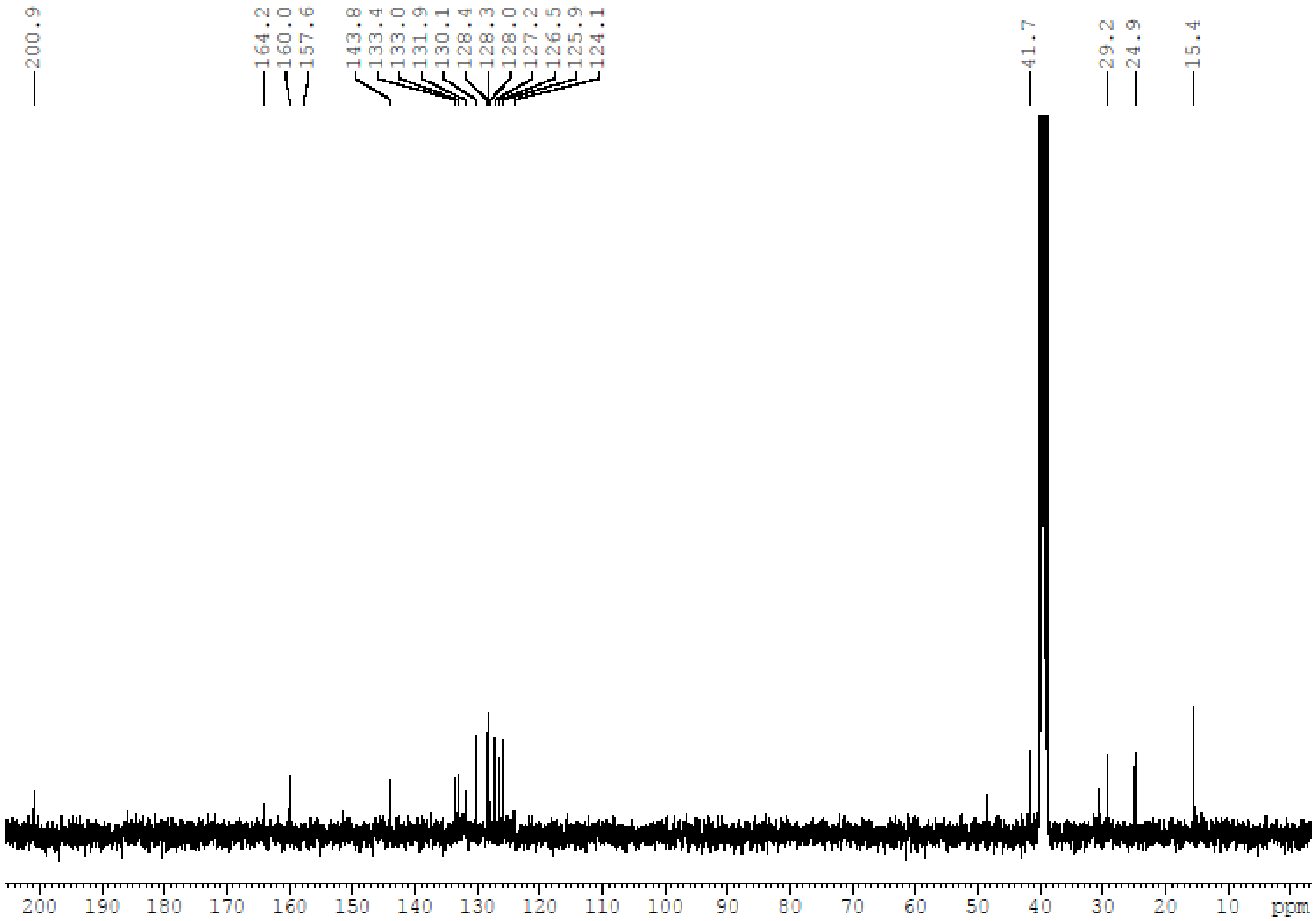

Figure S122: ^{13}C NMR spectrum of **19m** (100 MHz; DMSO-d_6).

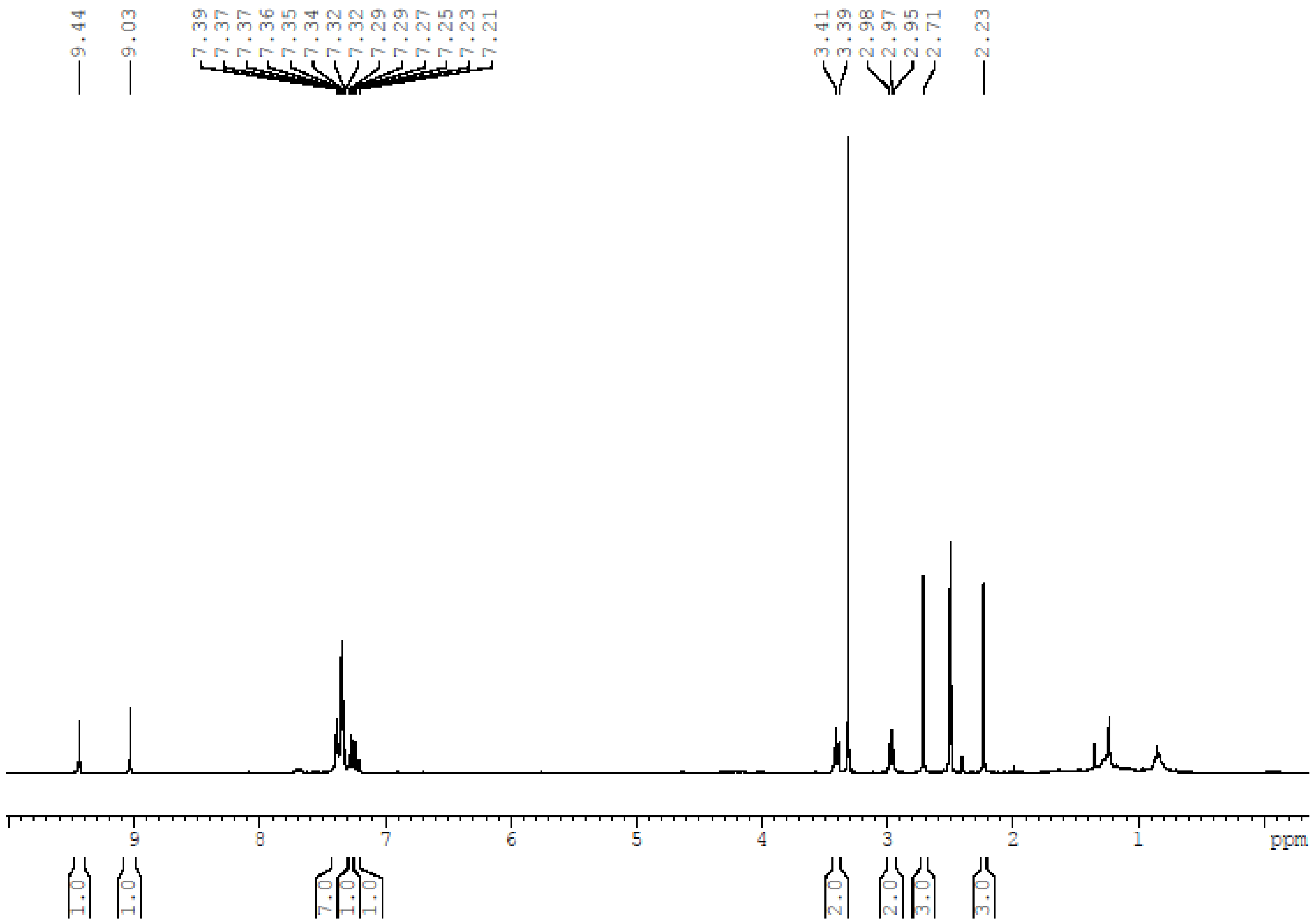

Figure S123: ^{1}H NMR spectrum of **19n** (400 MHz; DMSO-d_6).

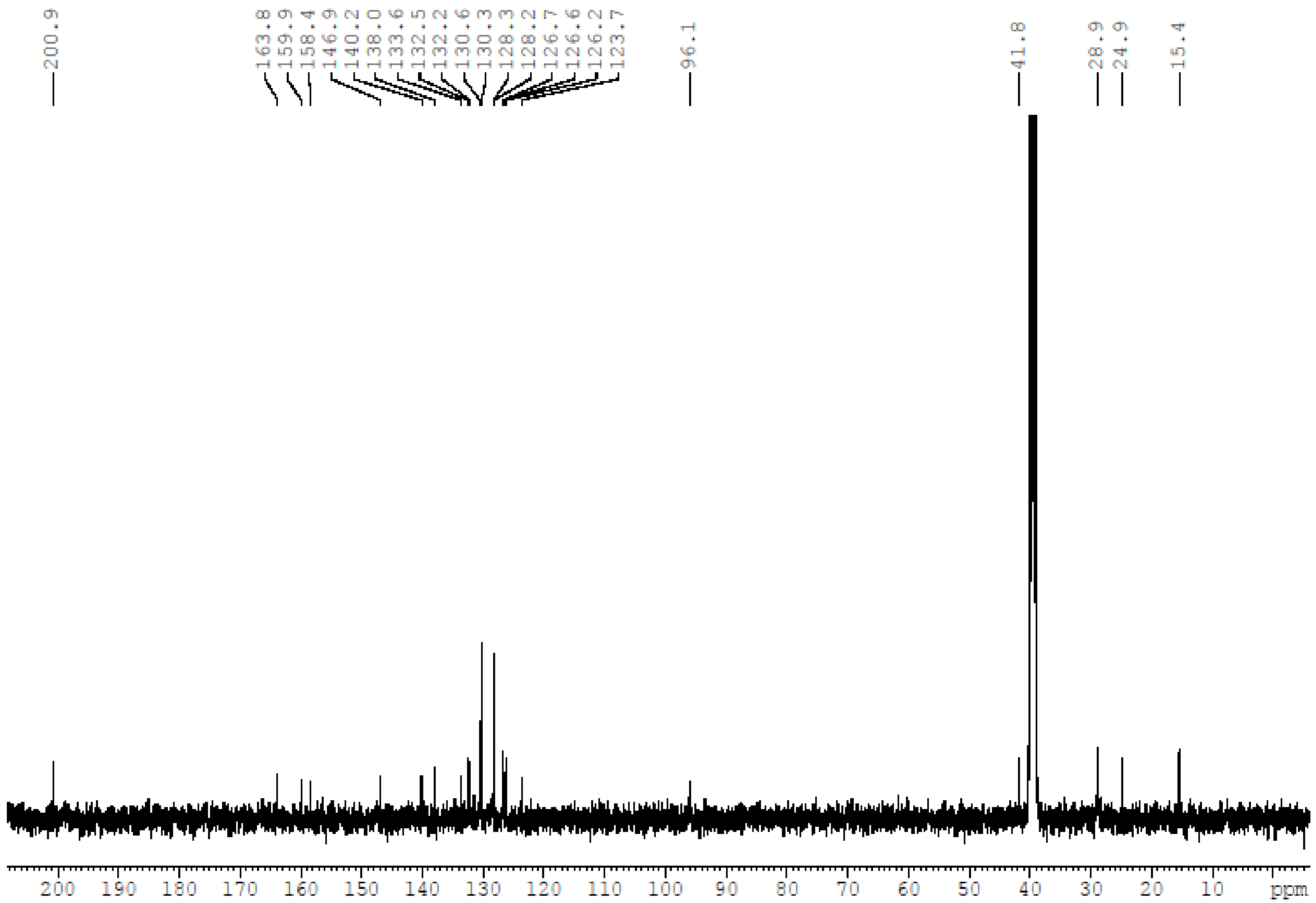

Figure S124: ^{13}C NMR spectrum of **19n** (100 MHz; DMSO-d_6).

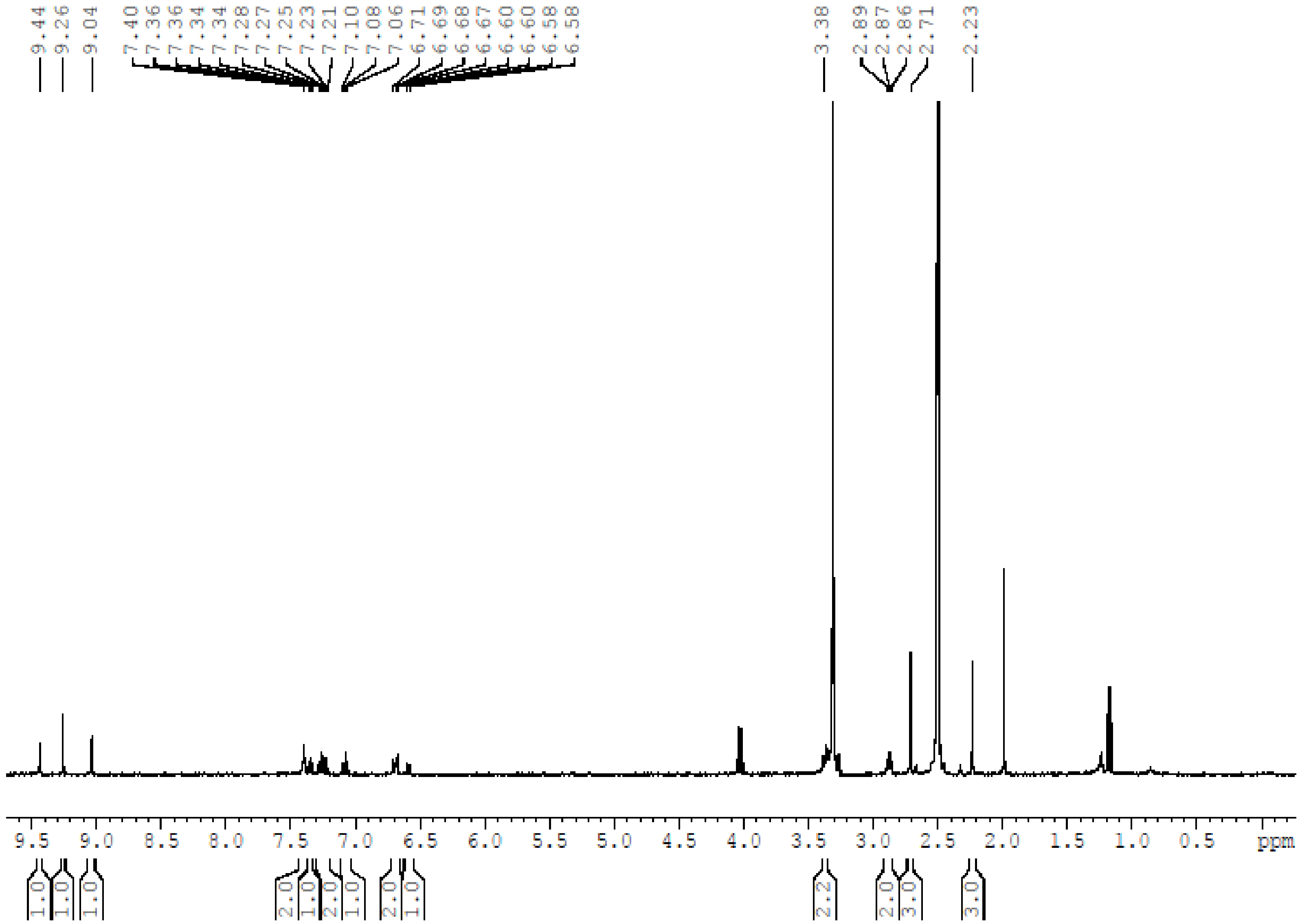

Figure S125: ^{1}H NMR spectrum of **19o** (400 MHz; DMSO-d_6).

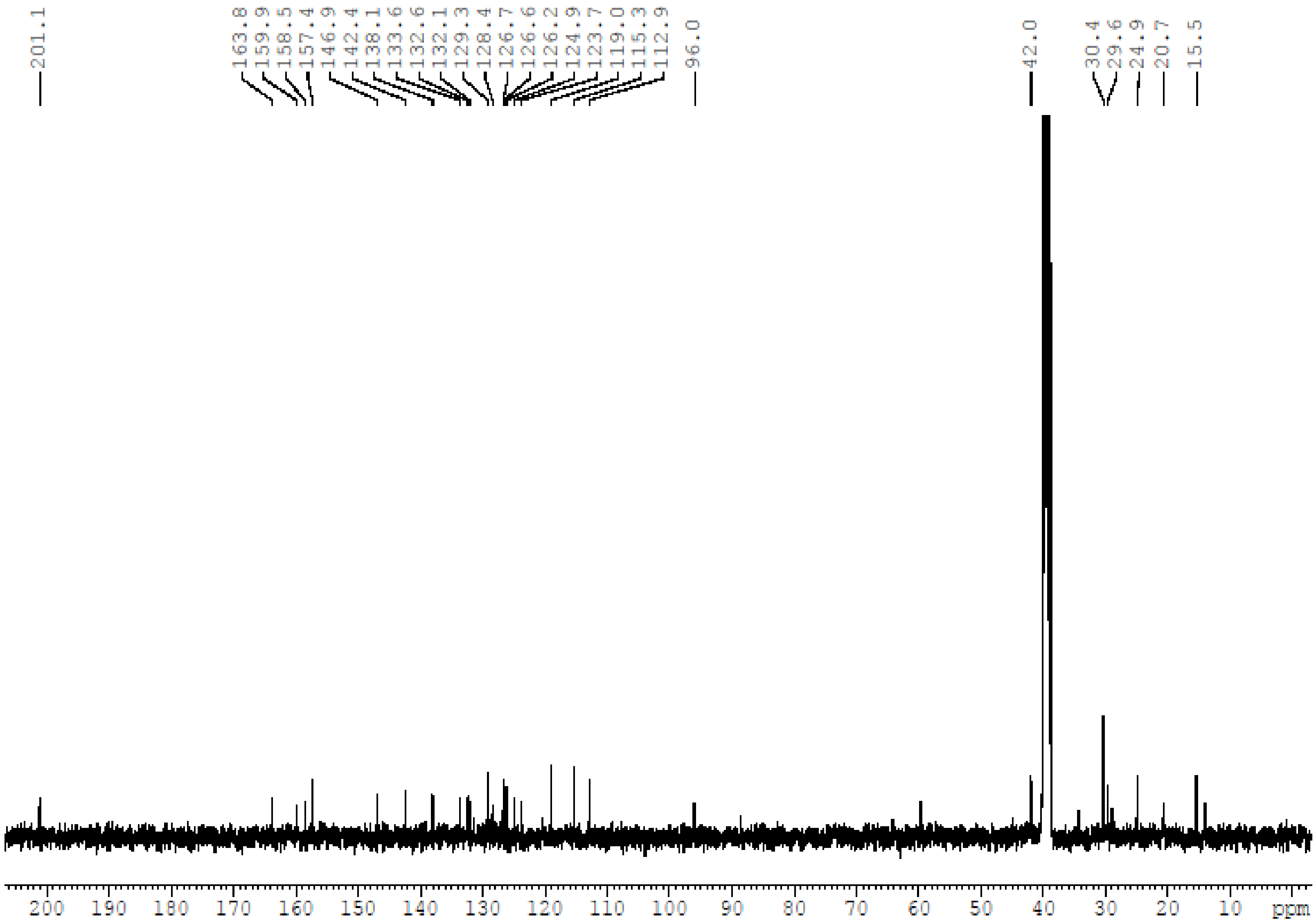

Figure S126: ^{13}C NMR spectrum of **19o** (100 MHz; DMSO-d_6).

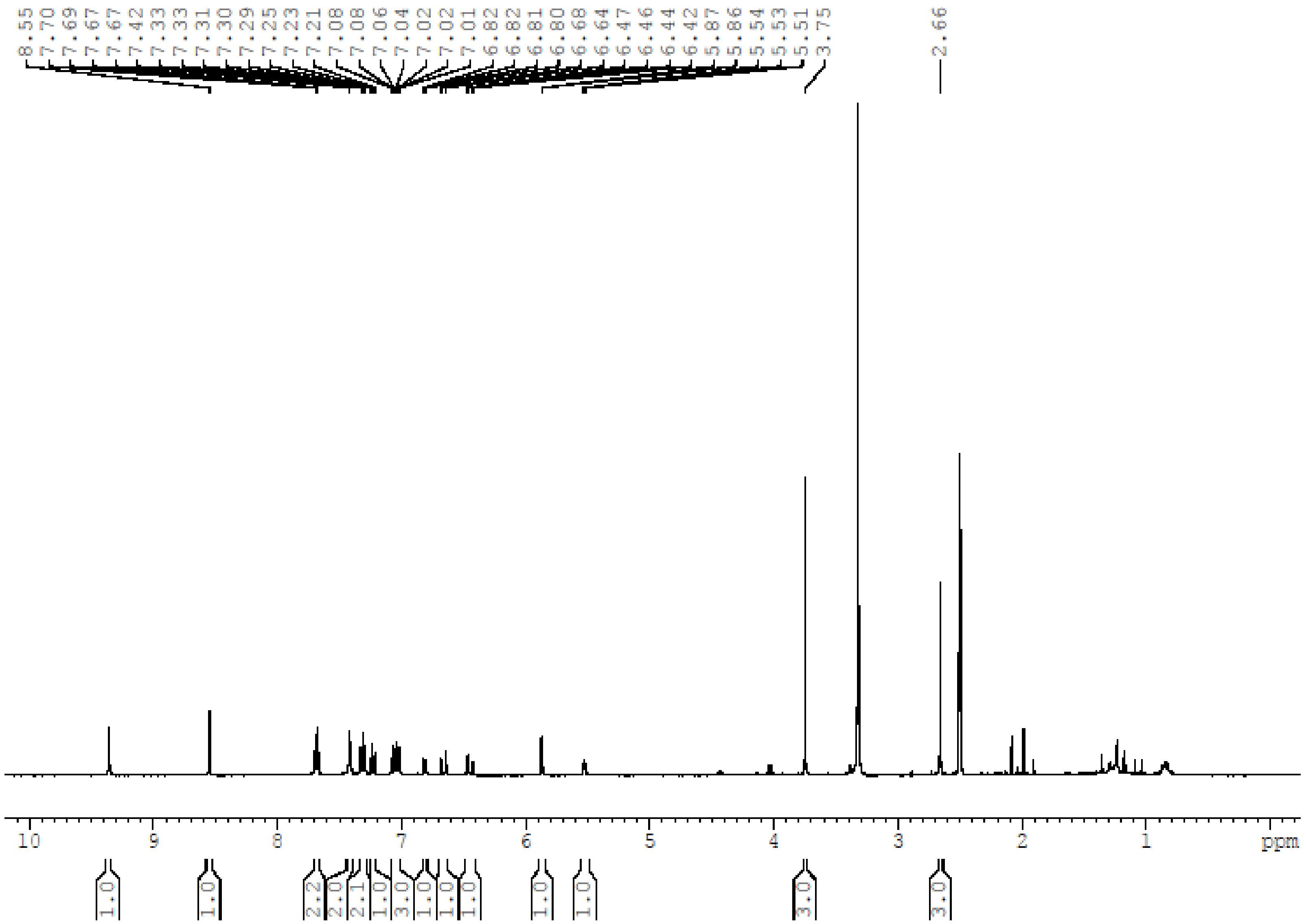

Figure S127: ^{1}H NMR spectrum of **20a** (400 MHz; DMSO-d_6).

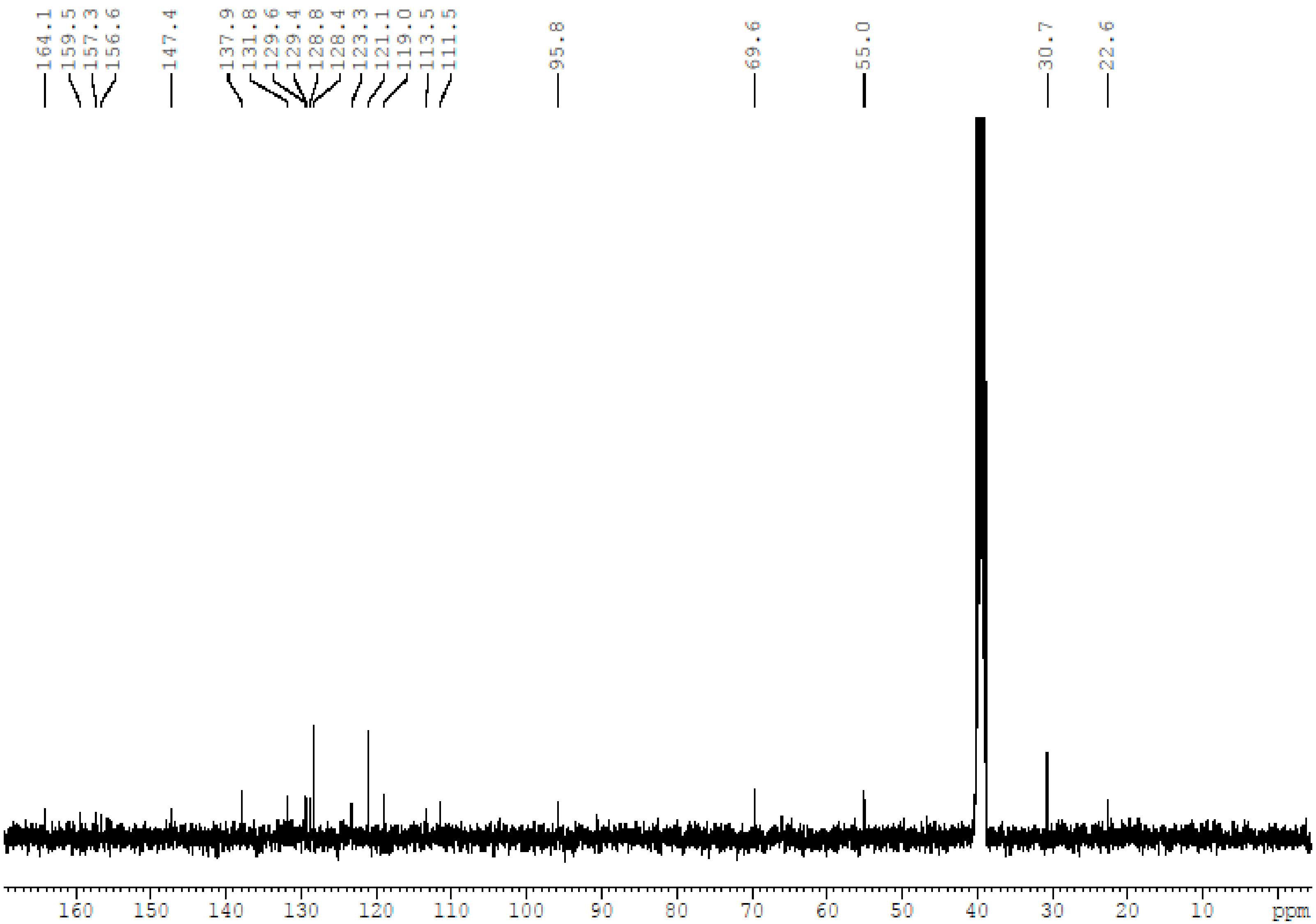

Figure S128: ^{13}C NMR spectrum of **20a** (100 MHz; DMSO-d_6).

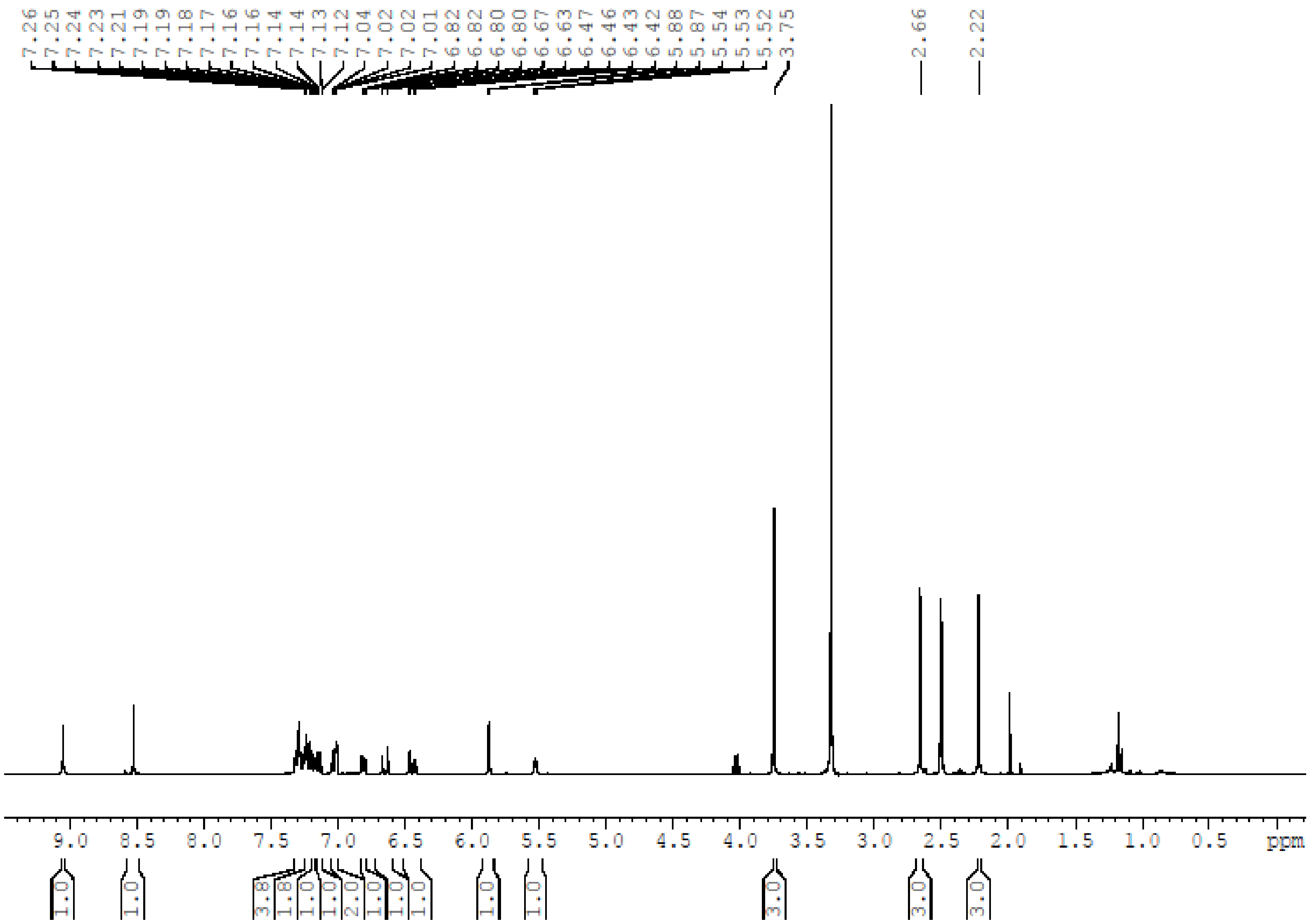

Figure S129: ^{1}H NMR spectrum of **20b** (400 MHz; DMSO-d_6).

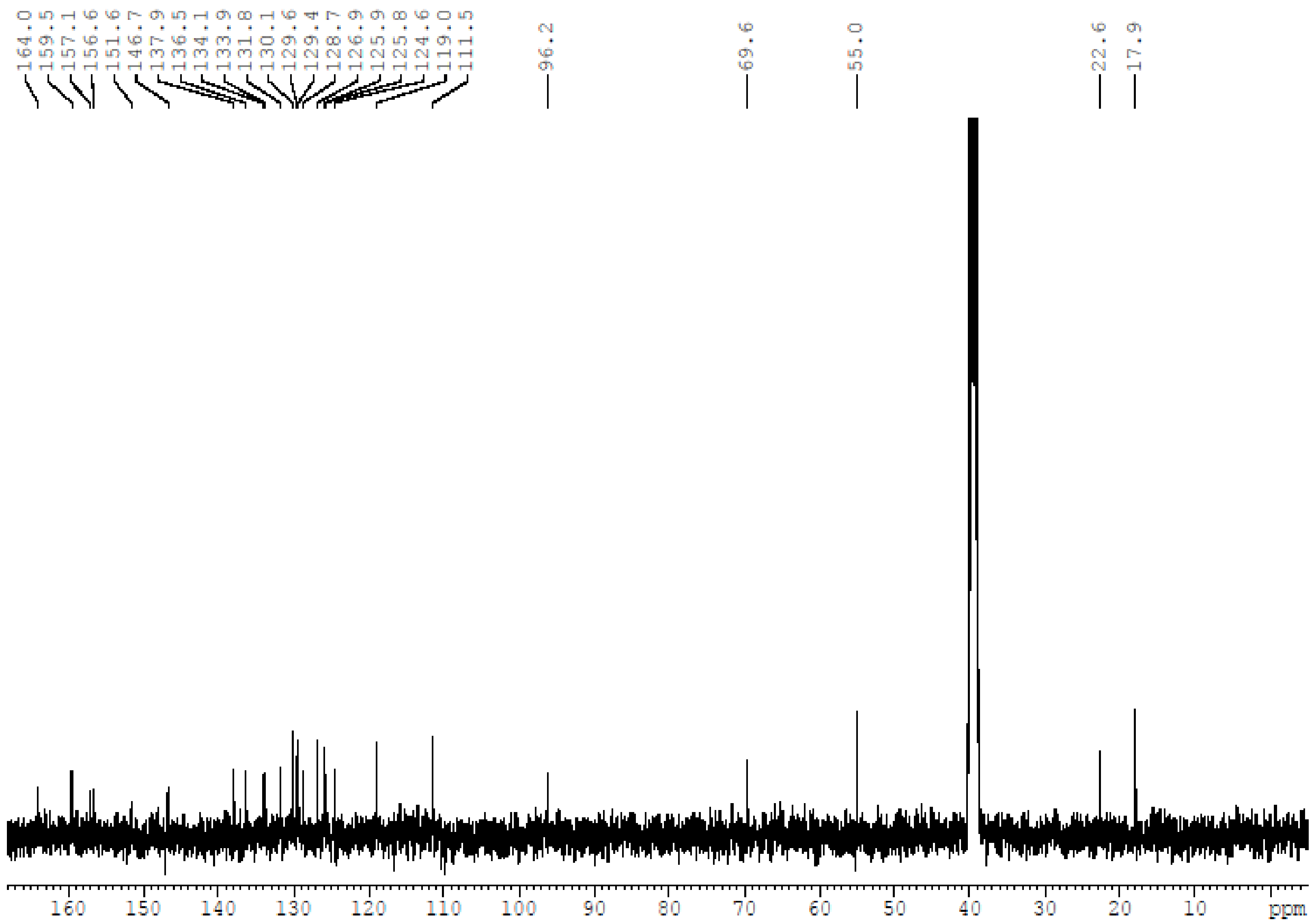

Figure S130: ^{13}C NMR spectrum of **20b** (100 MHz; DMSO-d_6).

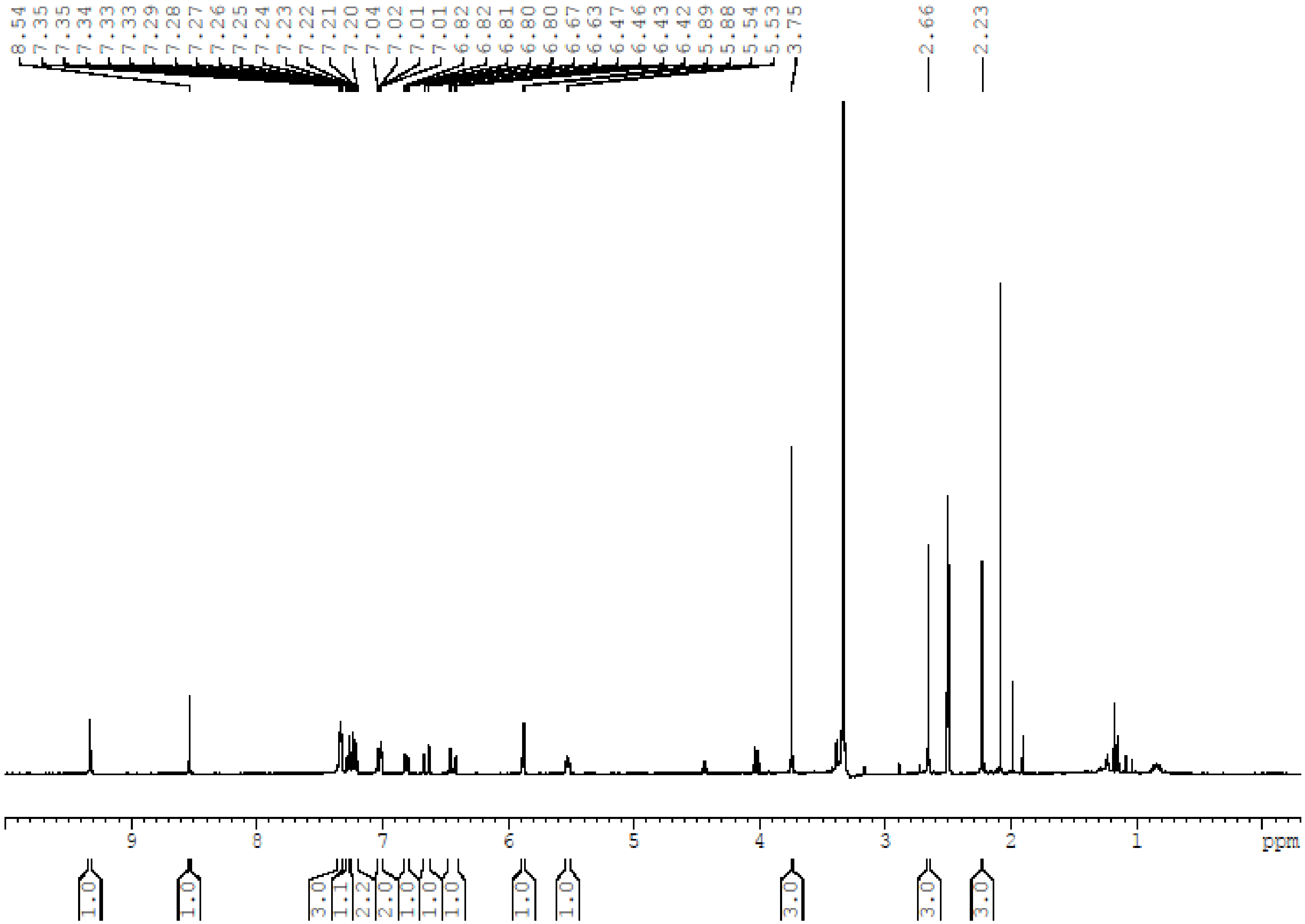

Figure S131: ^{1}H NMR spectrum of **20c** (400 MHz; DMSO-d_6).

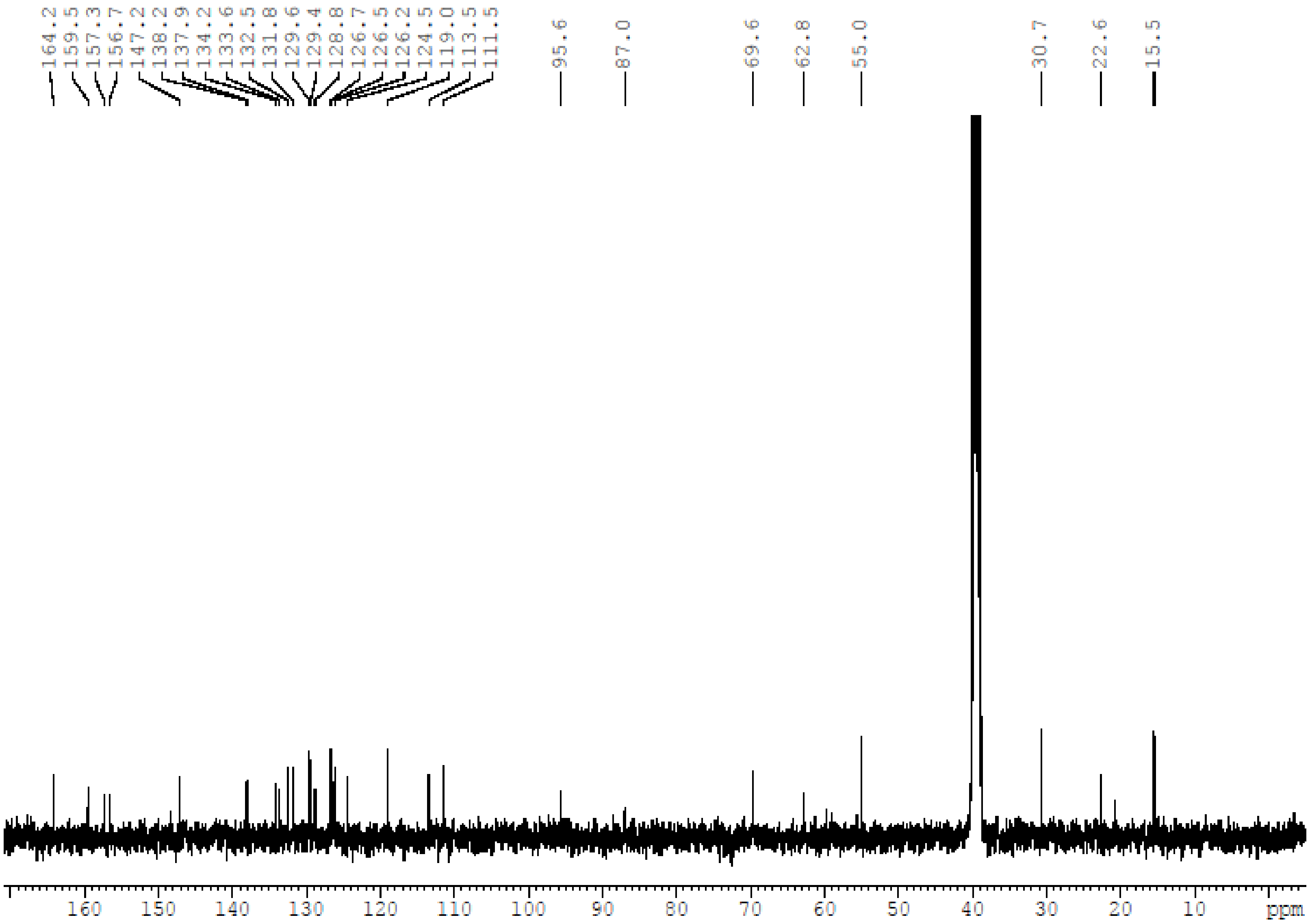

Figure S132: ^{13}C NMR spectrum of **20c** (100 MHz; DMSO-d_6).

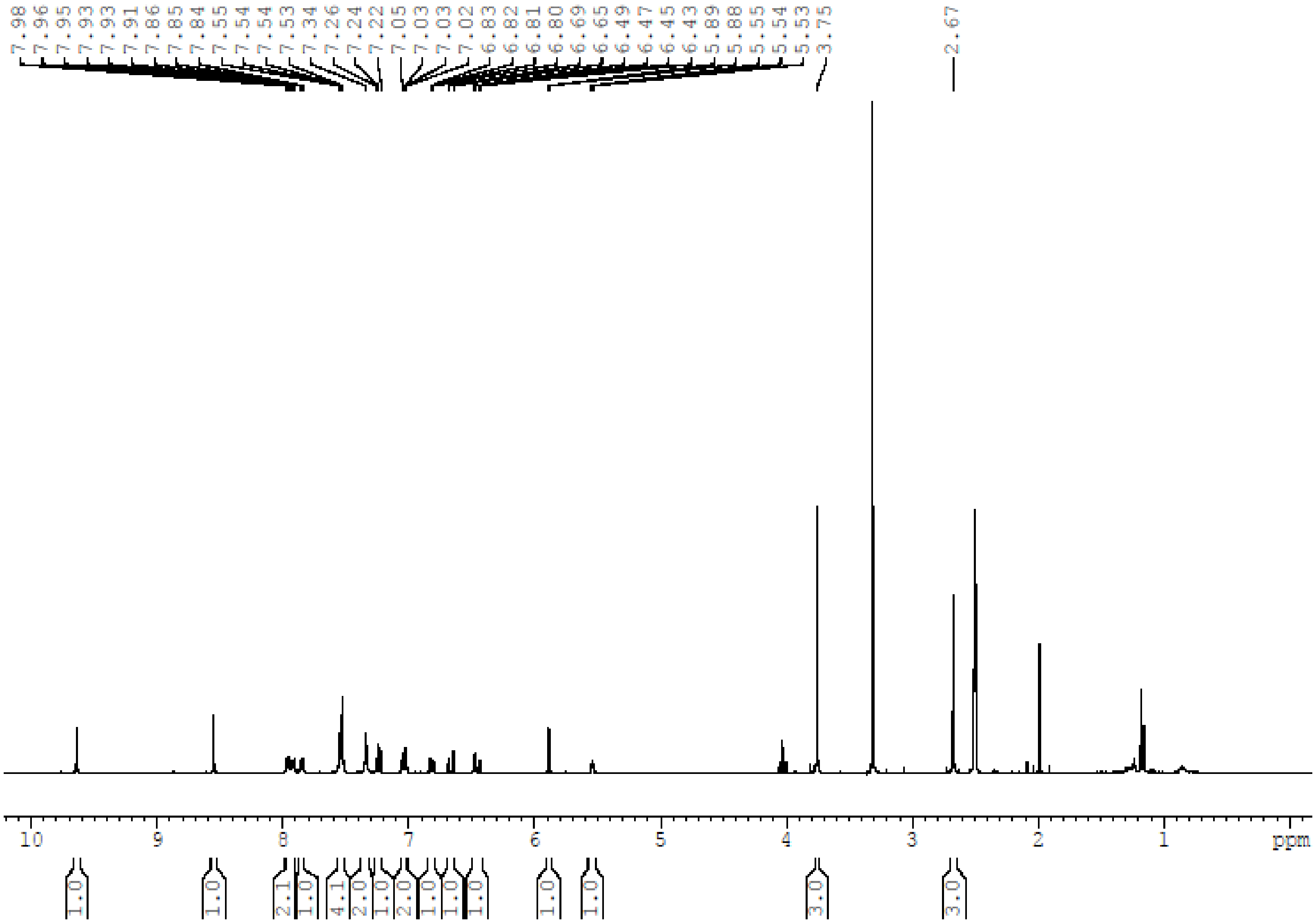

Figure S133: ^{1}H NMR spectrum of **20d** (400 MHz; DMSO-d_6).

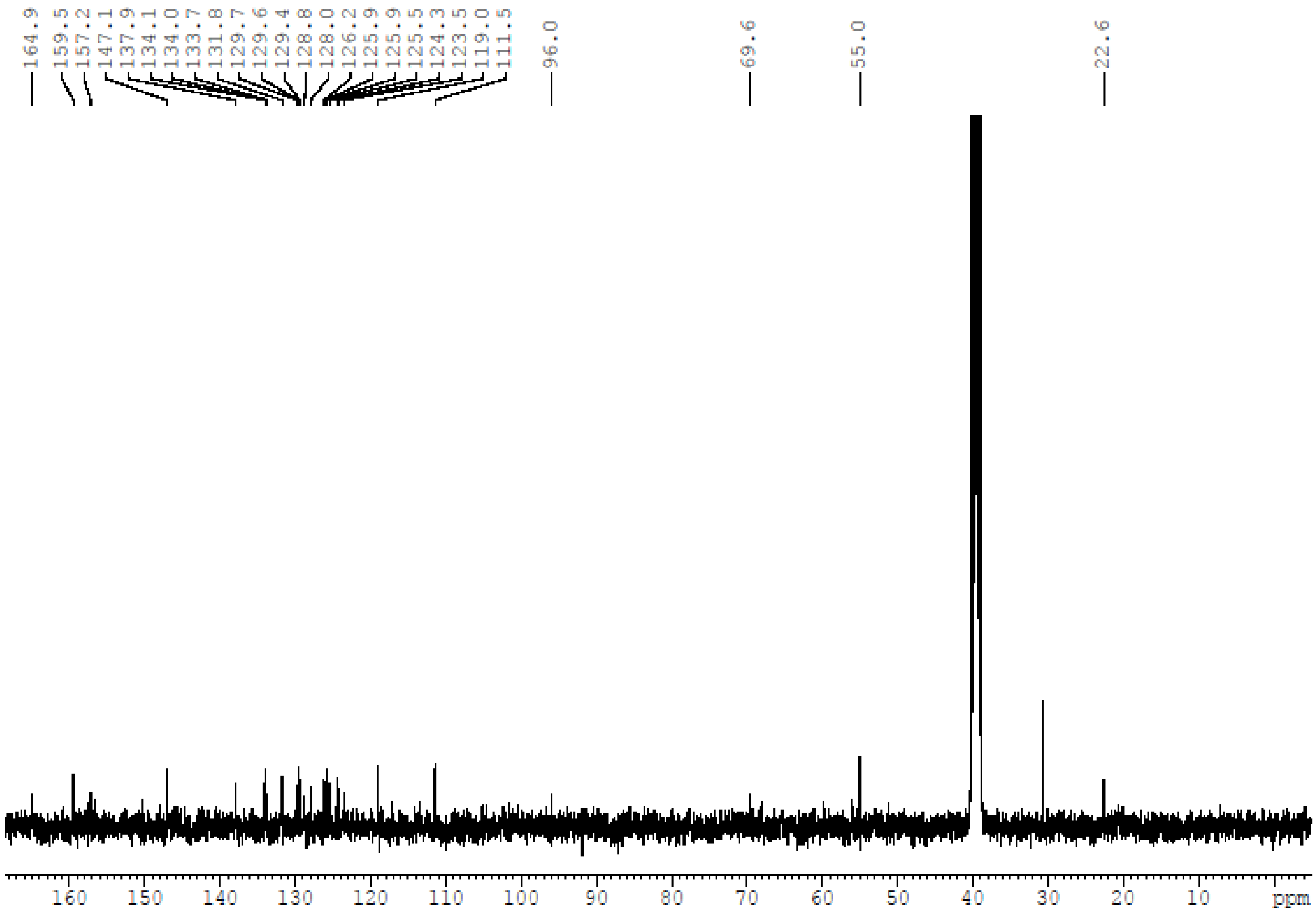

Figure S134: ^{13}C NMR spectrum of **20d** (100 MHz; DMSO-d_6).

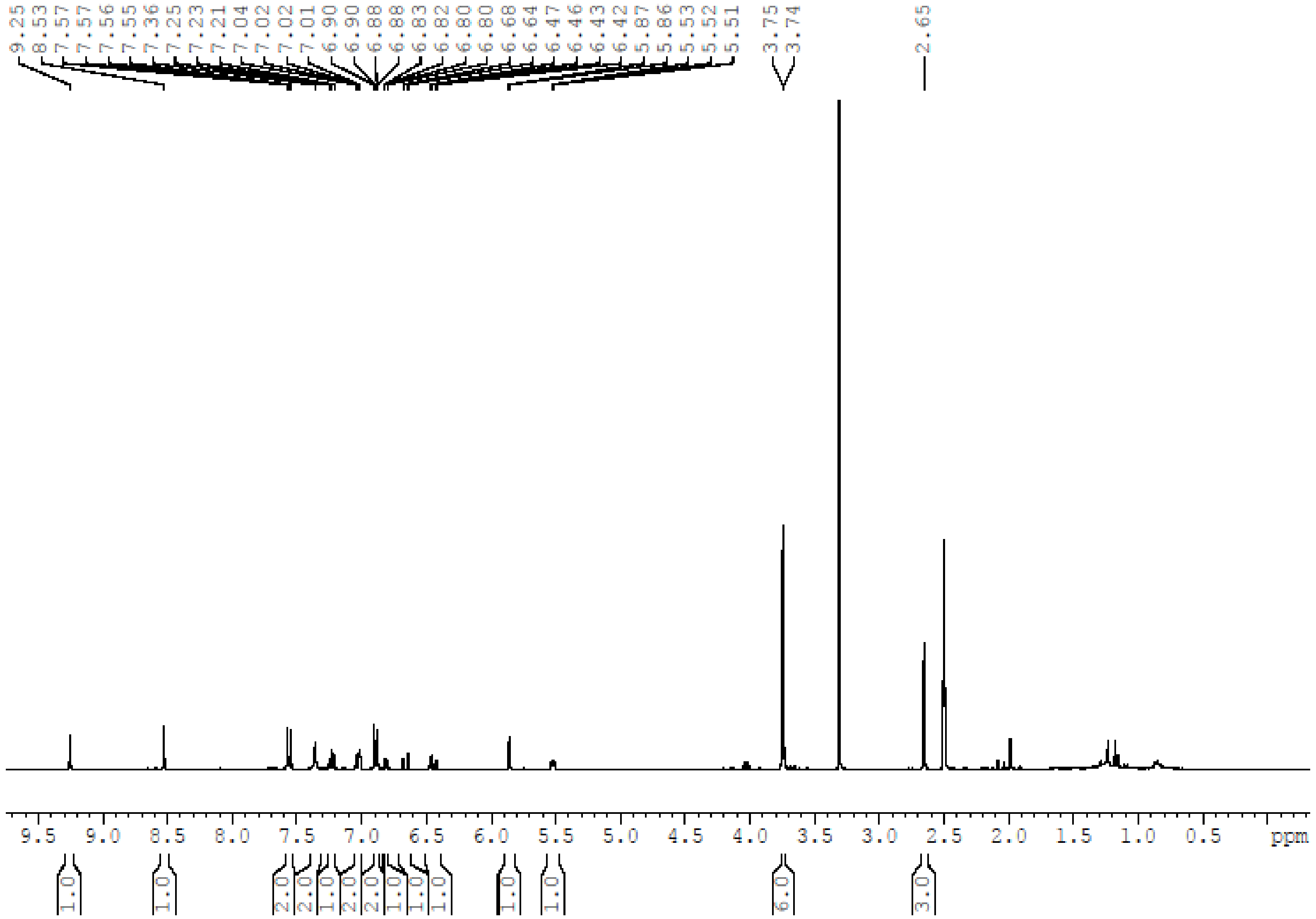

Figure S135: ^{1}H NMR spectrum of **20e** (400 MHz; DMSO-d_6).

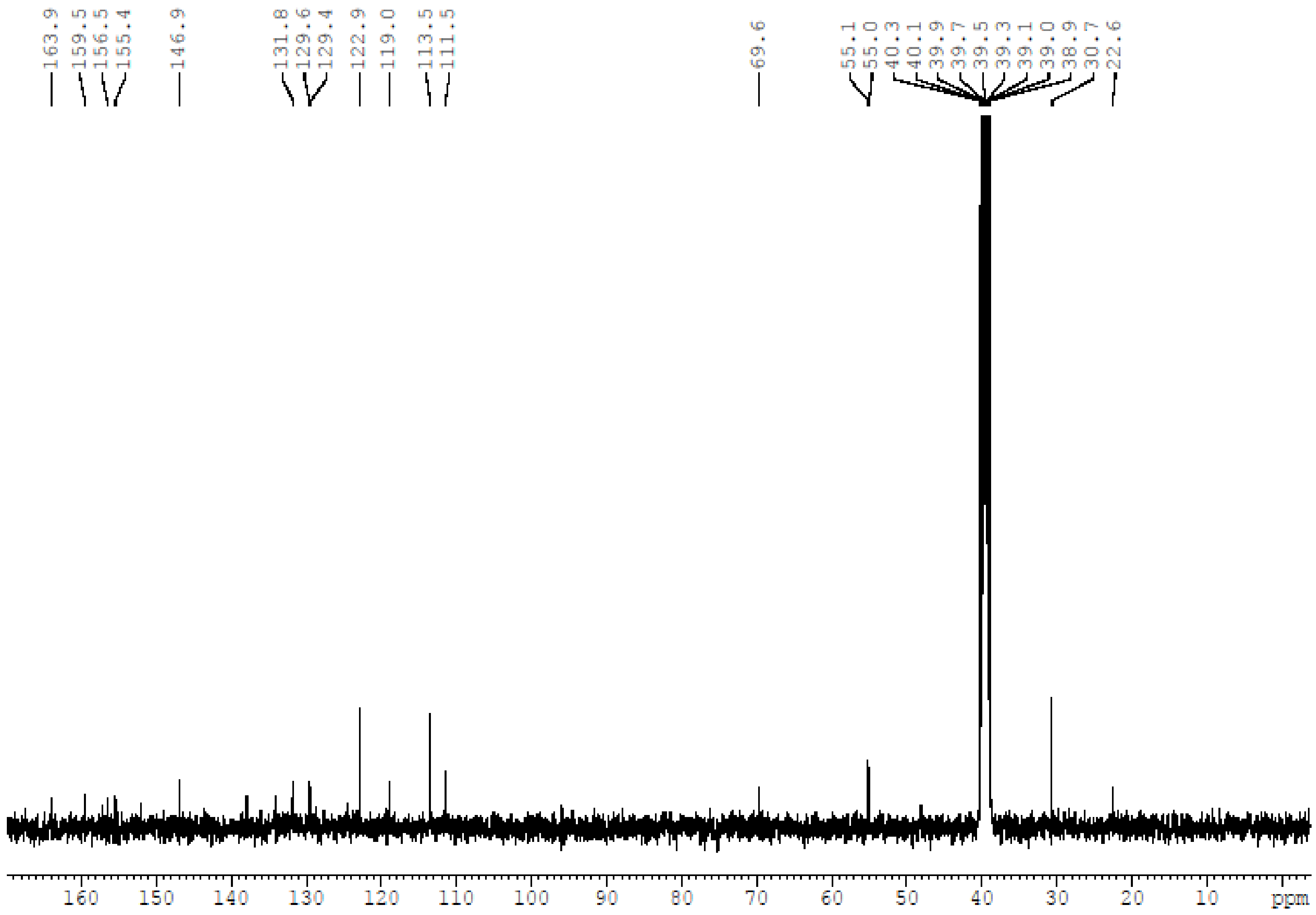

Figure S136: ^{13}C NMR spectrum of **20e** (100 MHz; DMSO-d_6).

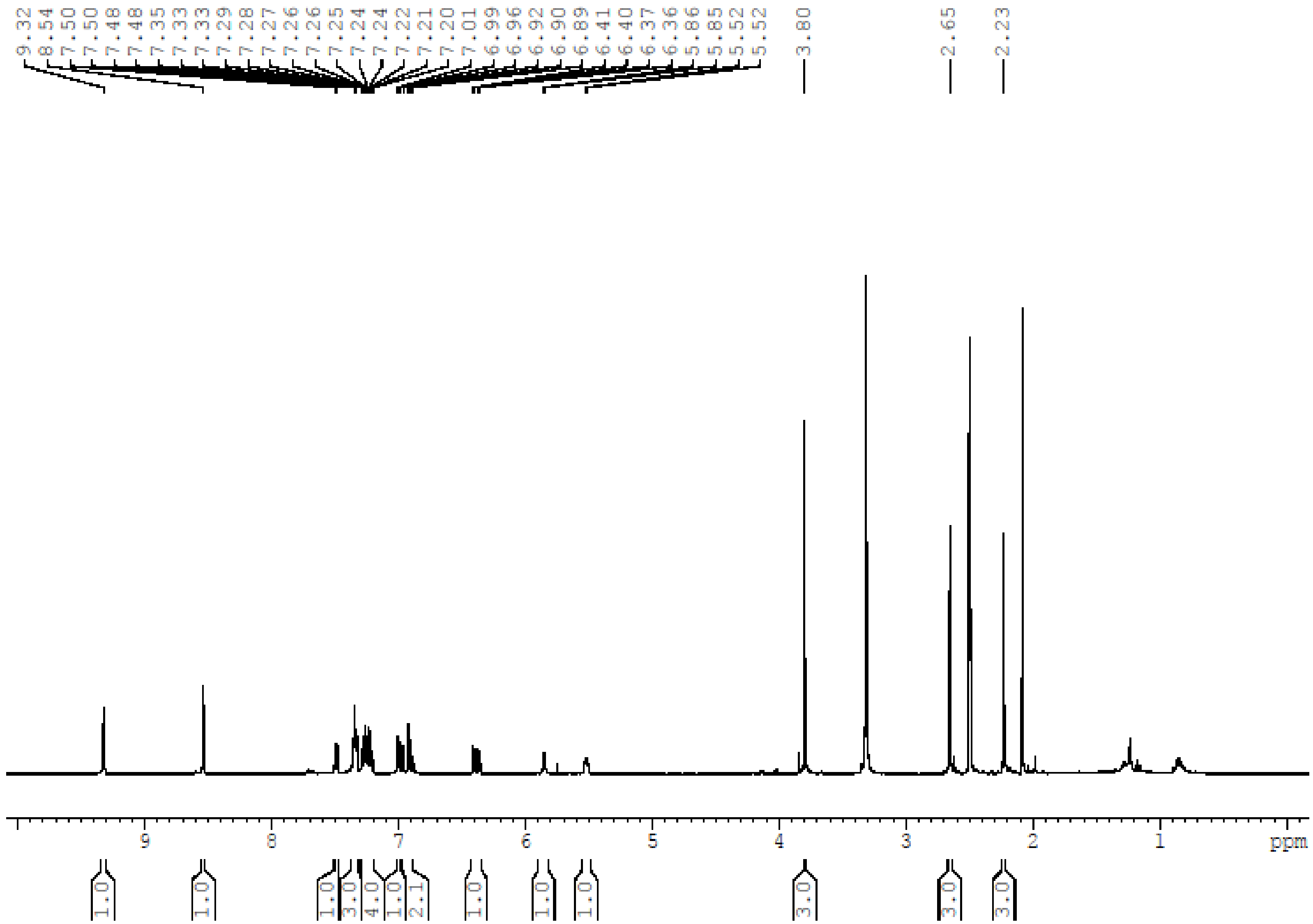

Figure S137: ^{1}H NMR spectrum of **21f** (400 MHz; DMSO-d_6).

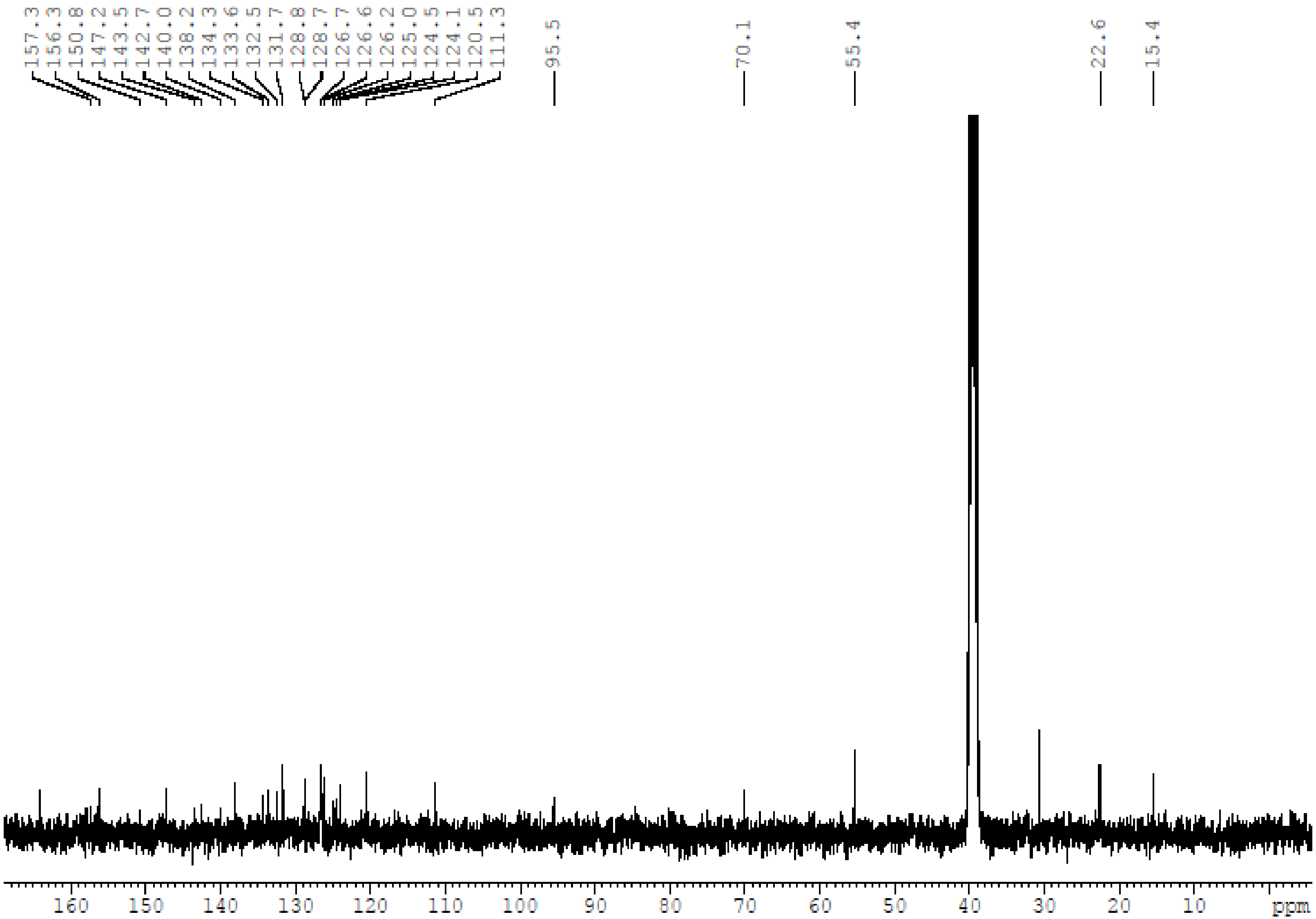

Figure S138: ^{13}C NMR spectrum of **21f** (100 MHz; DMSO-d_6).

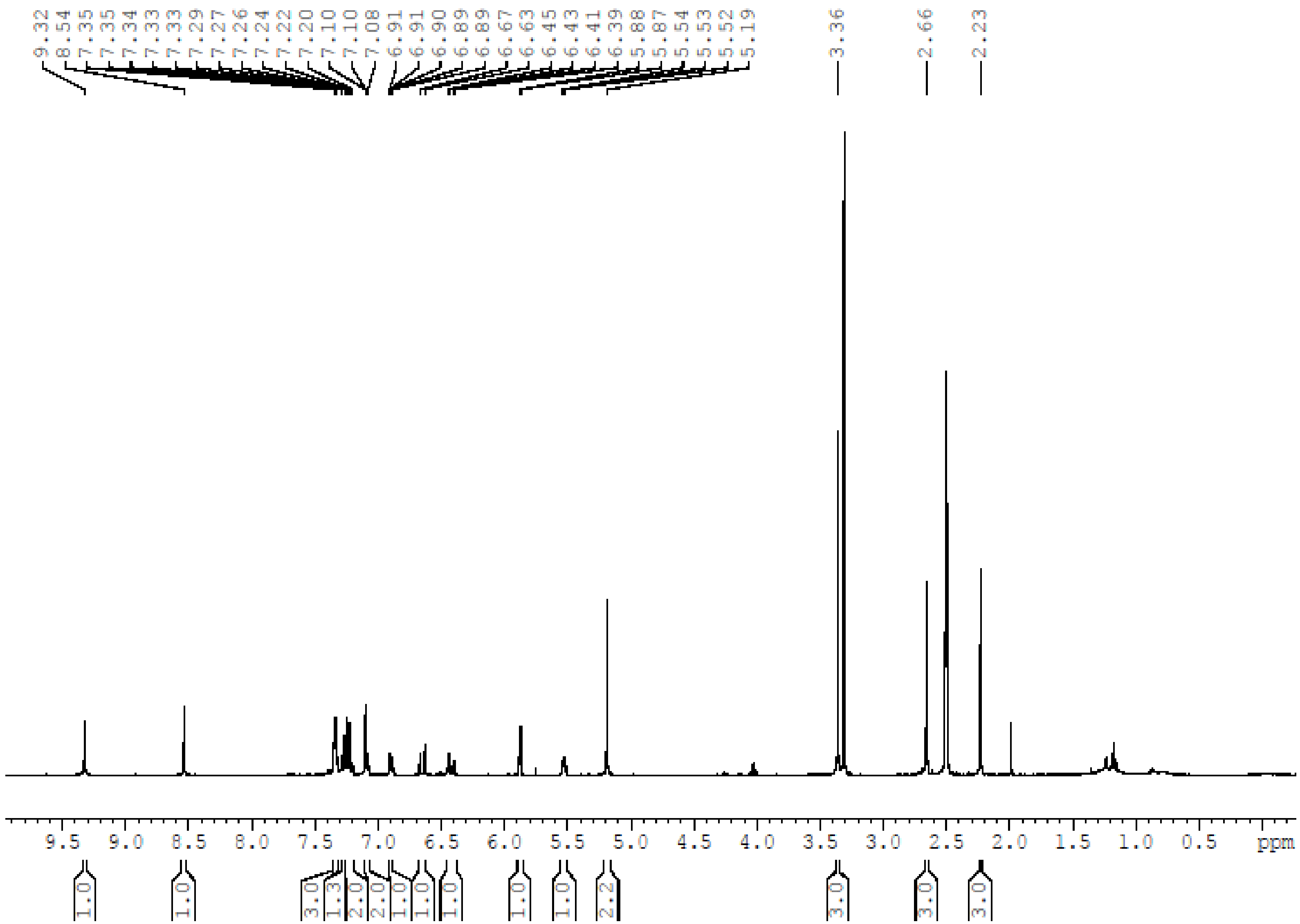

Figure S139: ^{1}H NMR spectrum of **21g** (400 MHz; DMSO-d_6).

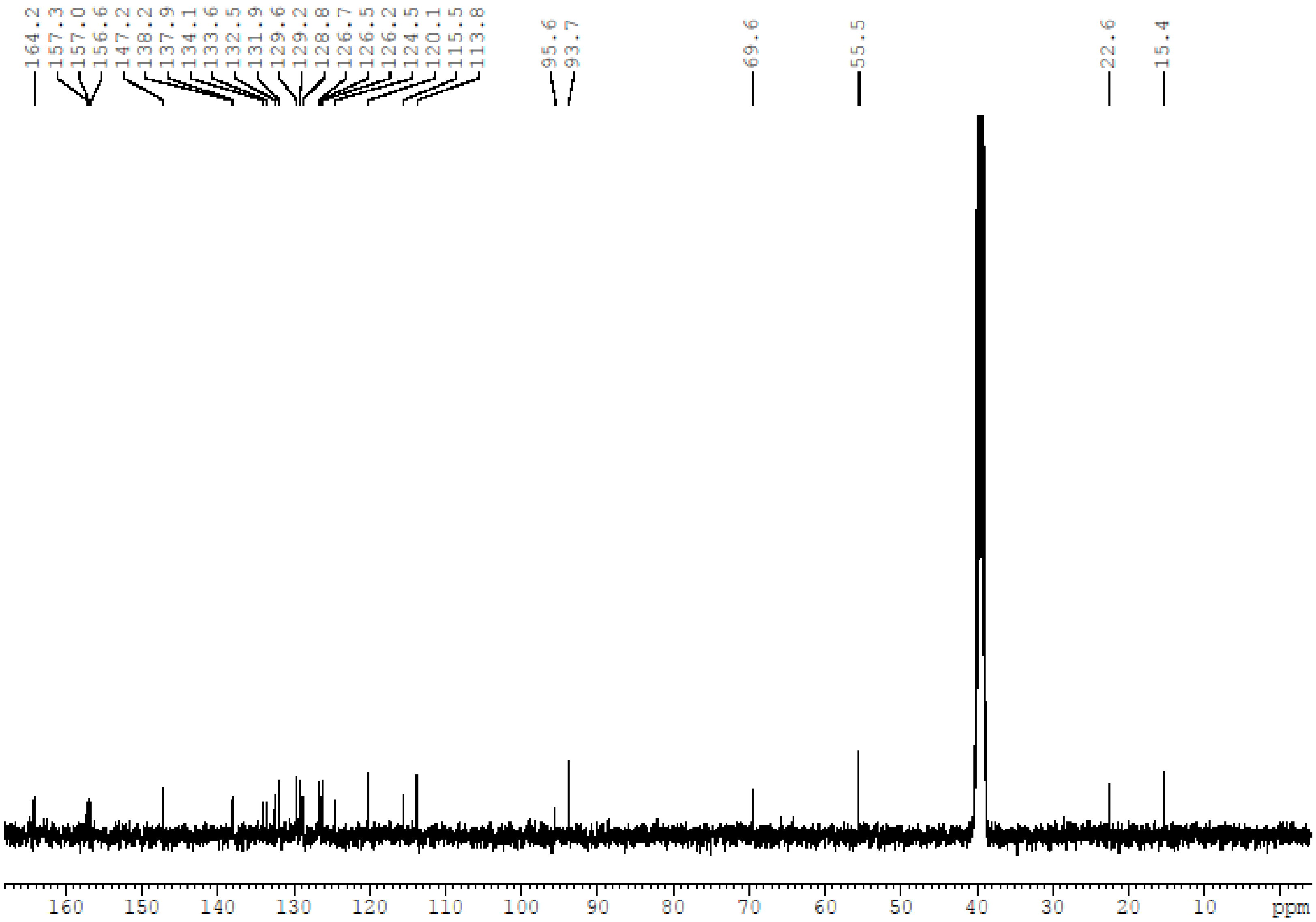

Figure S140: ^{13}C NMR spectrum of **21g** (100 MHz; DMSO-d_6).

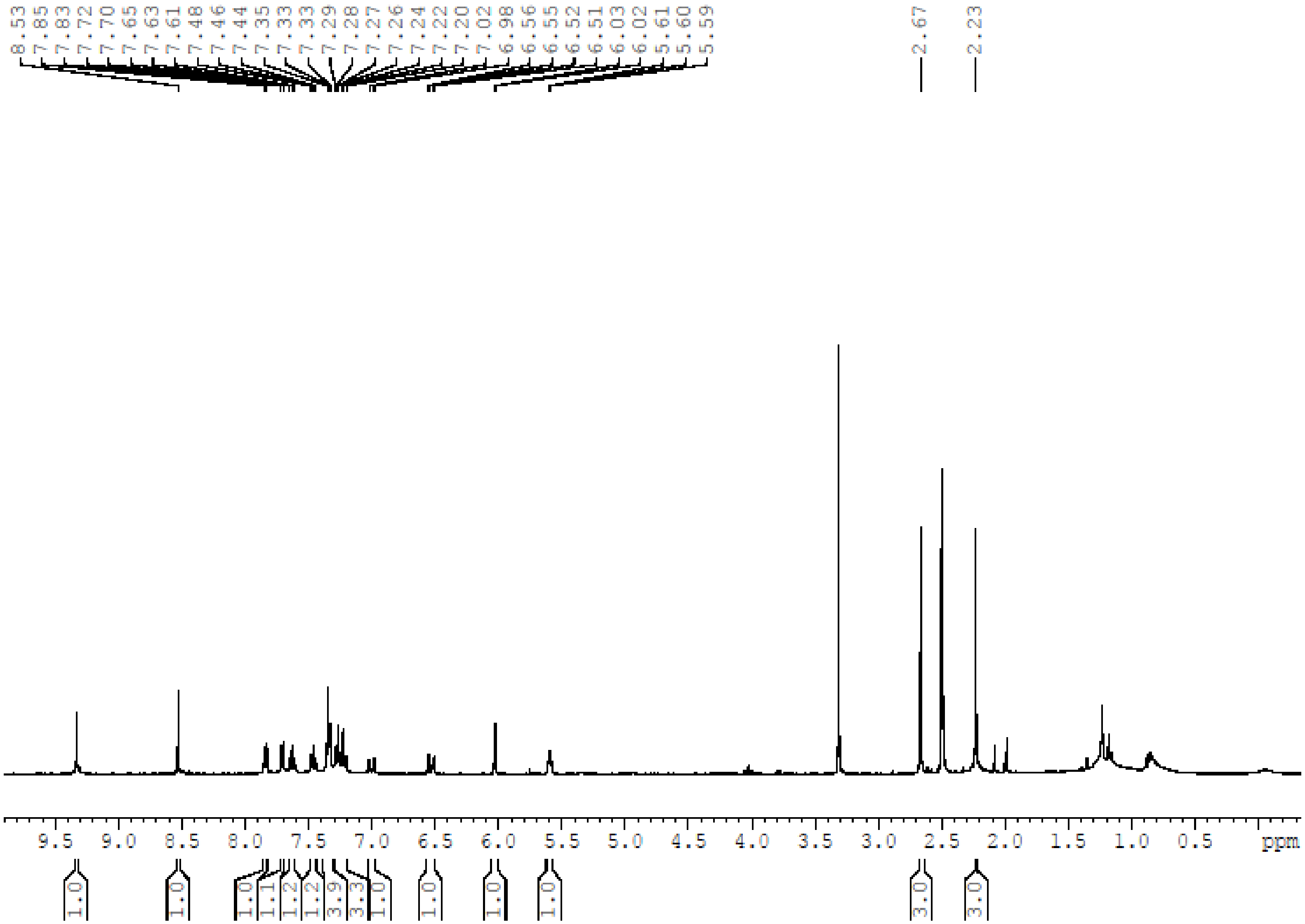

Figure S141: ^{1}H NMR spectrum of **21h** (400 MHz; DMSO-d_6).

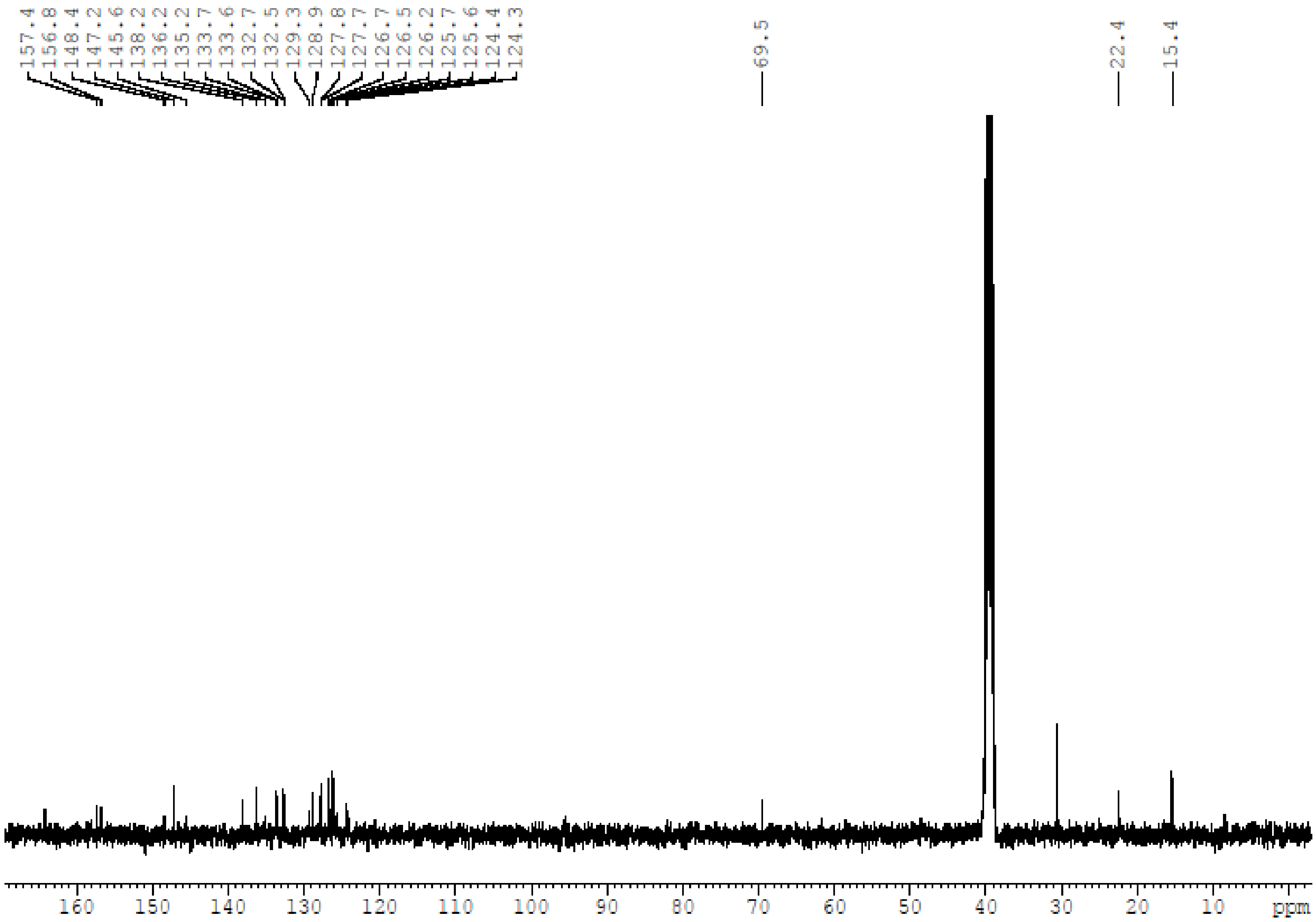

Figure S142: ^{13}C NMR spectrum of **21h** (100 MHz; DMSO-d_6).

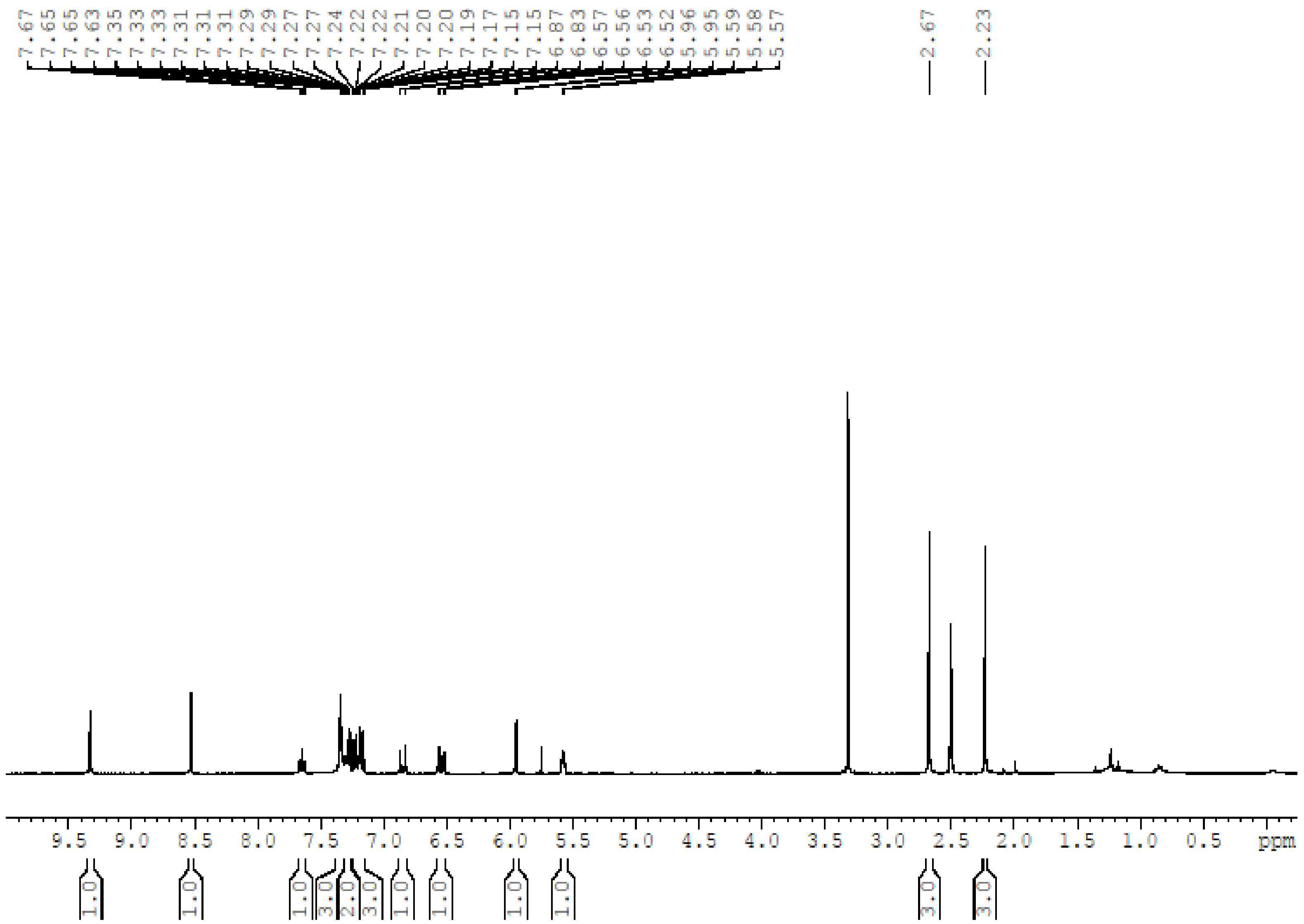

Figure S143: ^{1}H NMR spectrum of **21i** (400 MHz; DMSO-d_6).

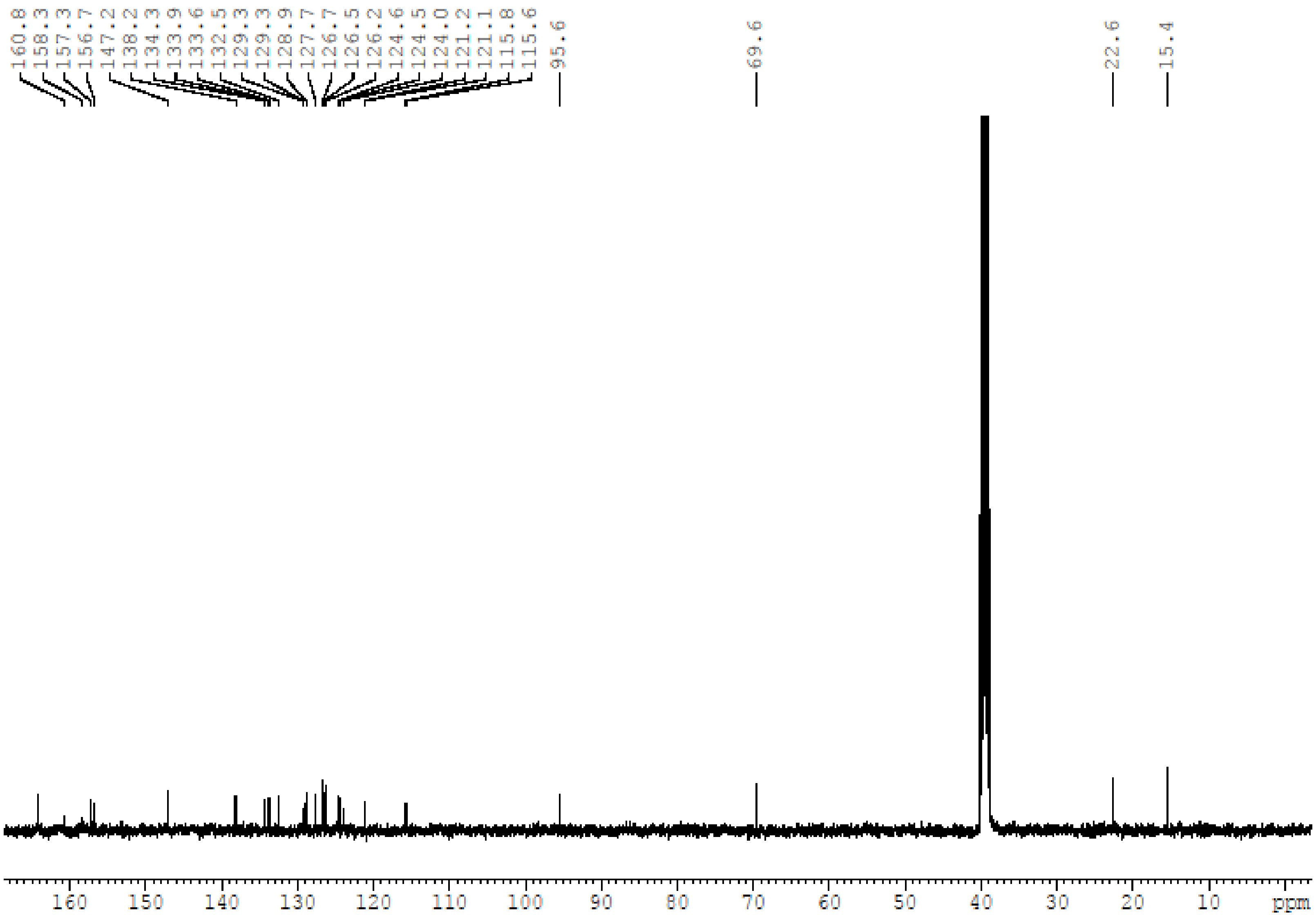

Figure S144: ^{13}C NMR spectrum of **21i** (100 MHz; DMSO-d_6).

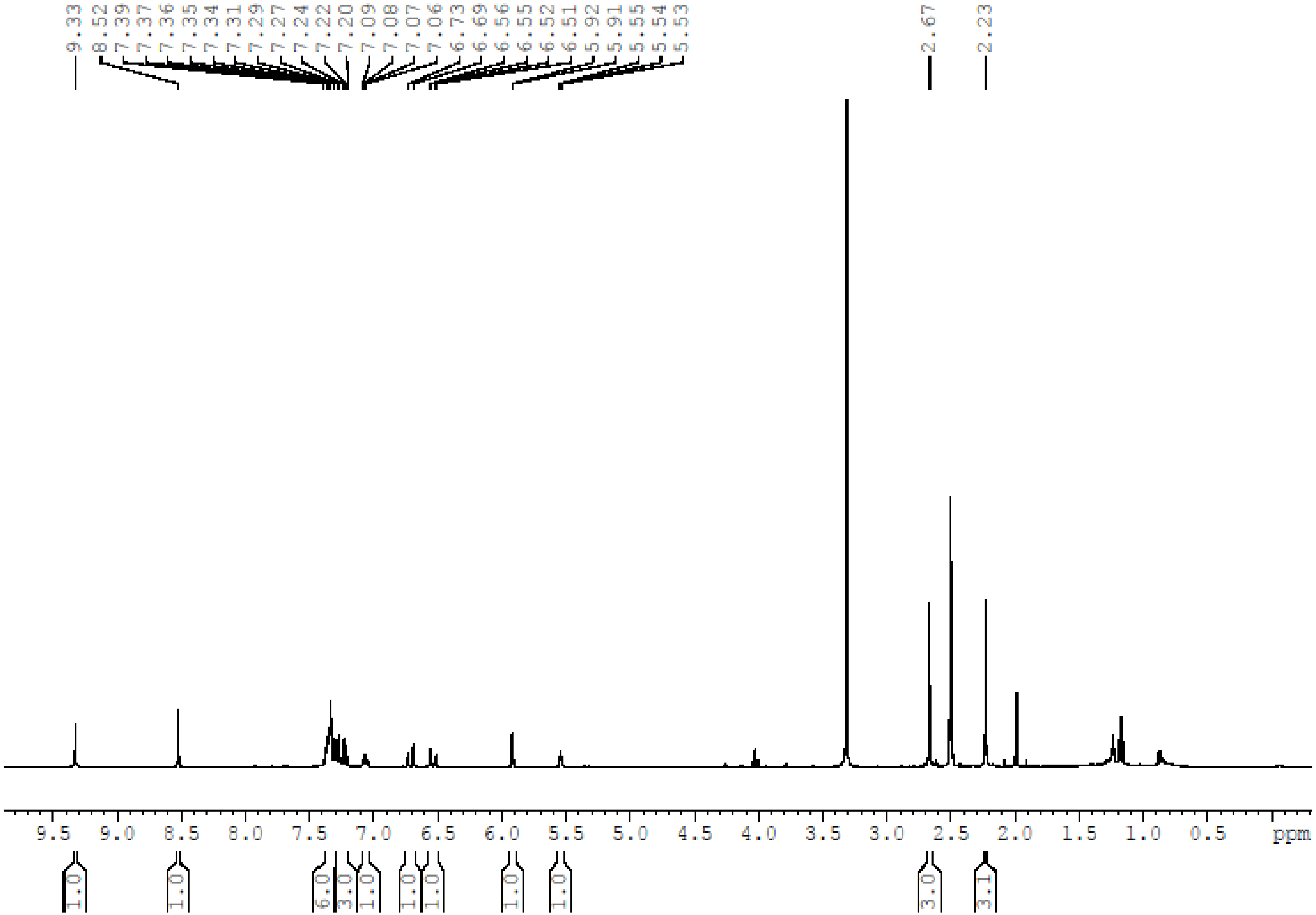

Figure S145: ^{1}H NMR spectrum of **21j** (400 MHz; DMSO-d_6).

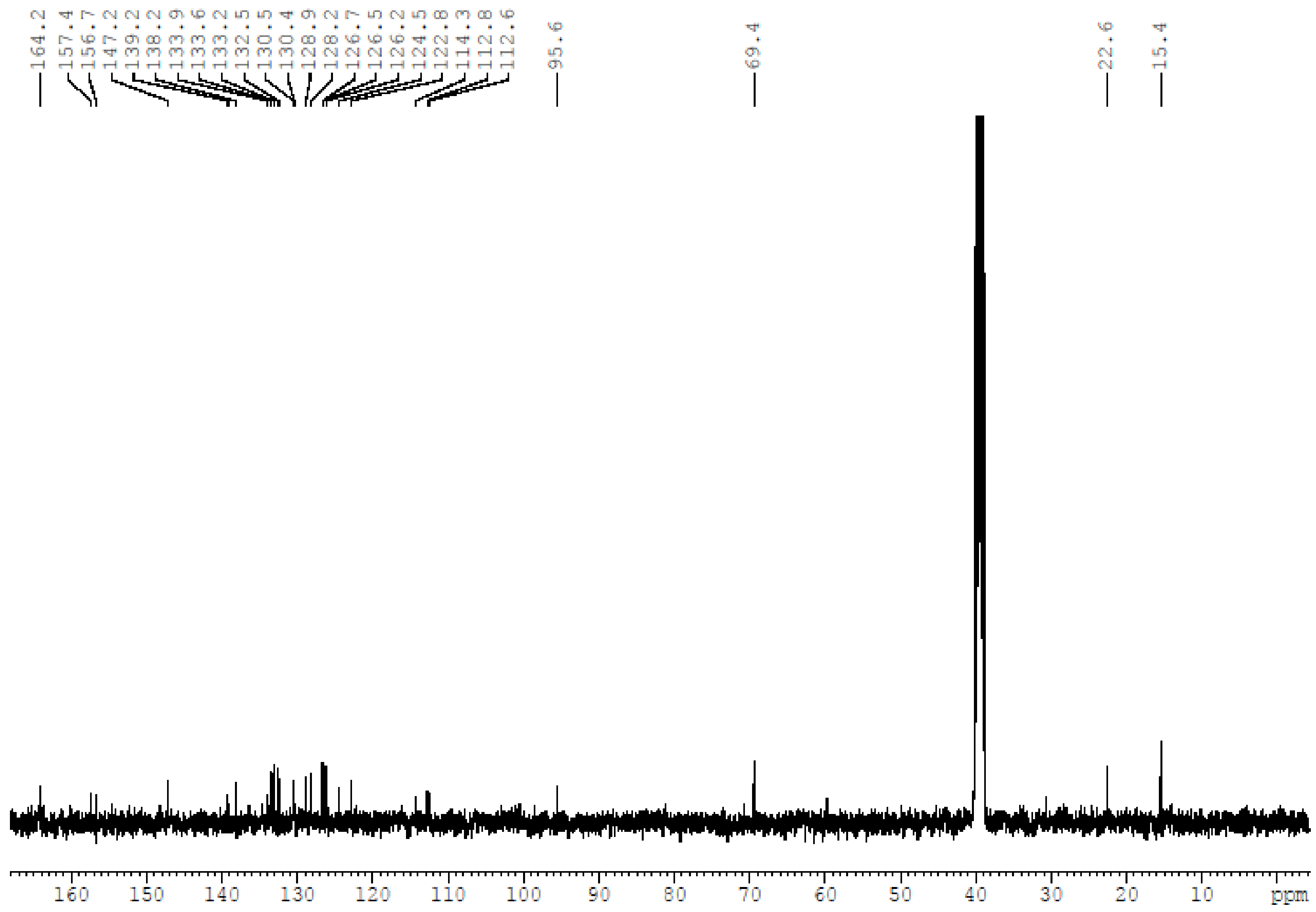

Figure S146: ^{13}C NMR spectrum of **21j** (100 MHz; DMSO-d_6).

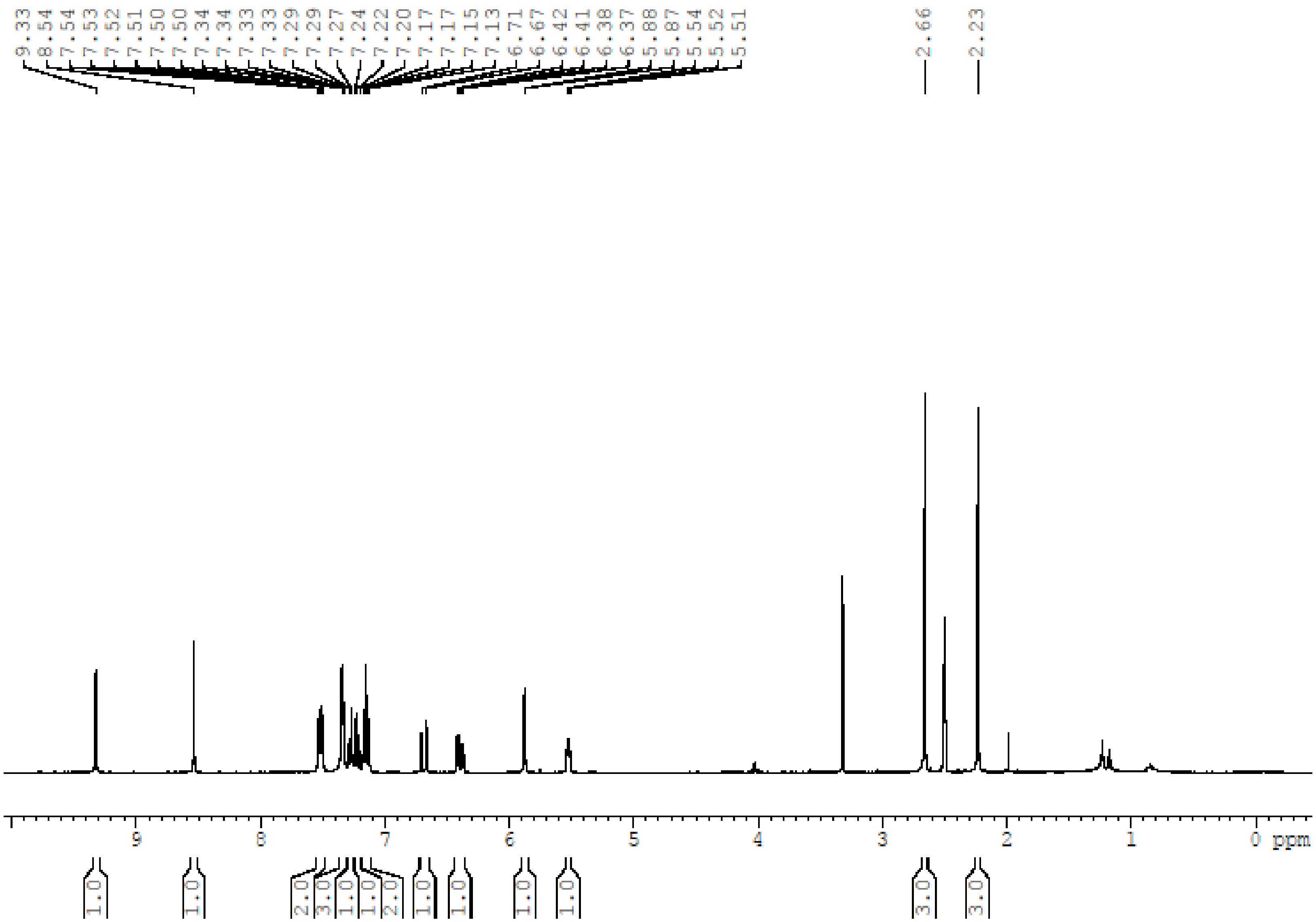

Figure S147: ^{1}H NMR spectrum of **21k** (400 MHz; DMSO-d_6).

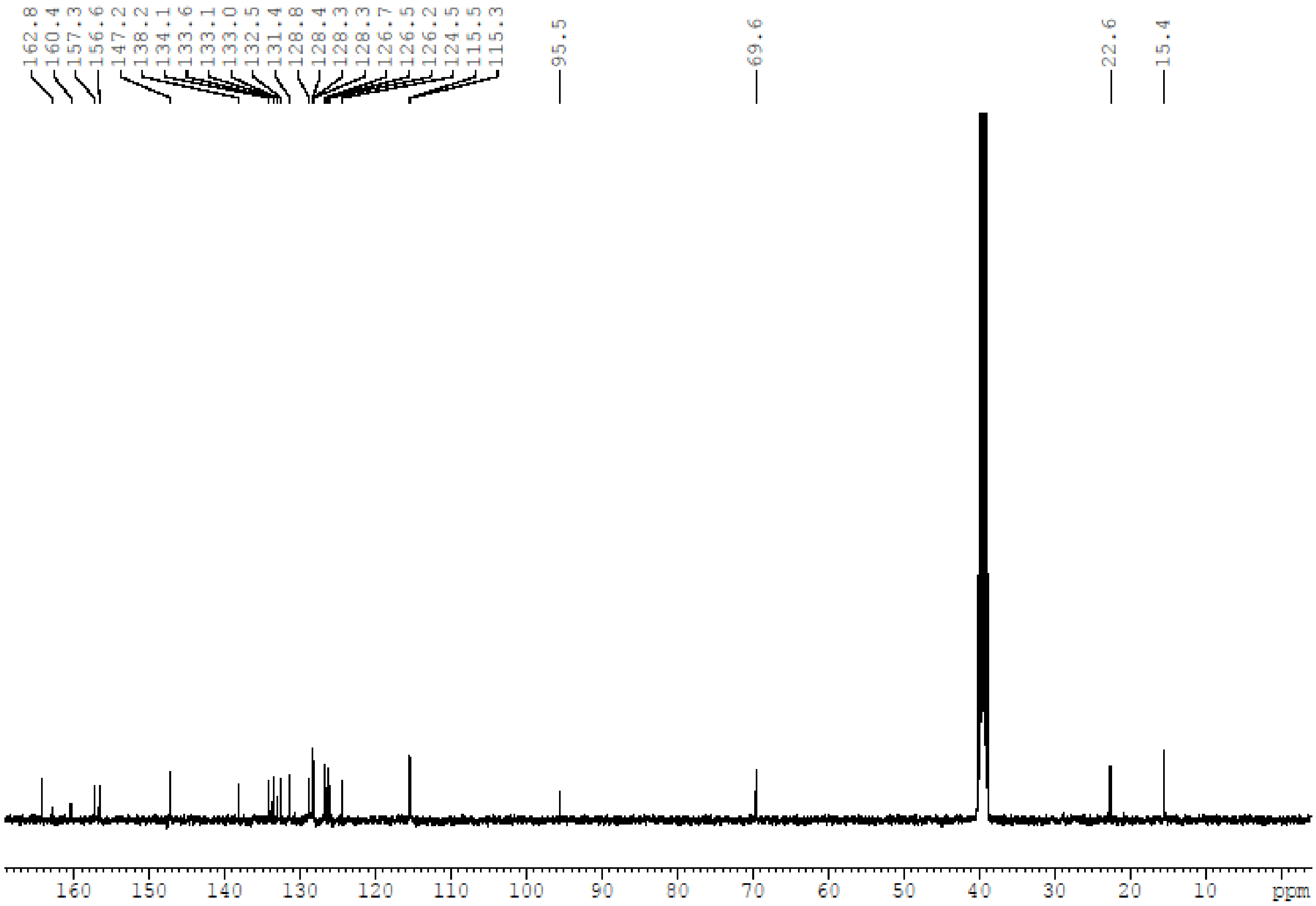

Figure S148: ^{13}C NMR spectrum of **21k** (100 MHz; DMSO-d_6).

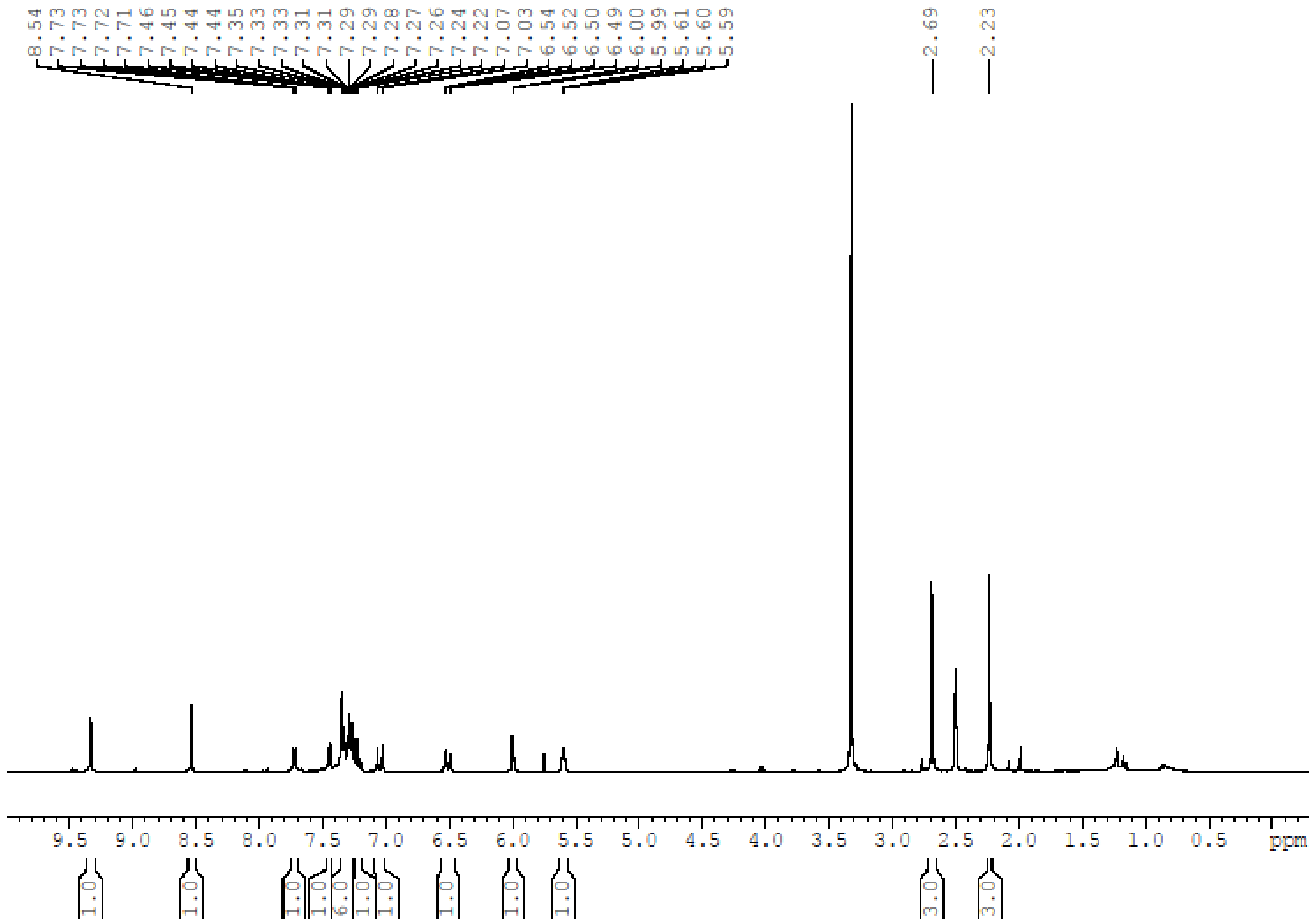

Figure S149: ^{1}H NMR spectrum of **21l** (400 MHz; DMSO-d_6).

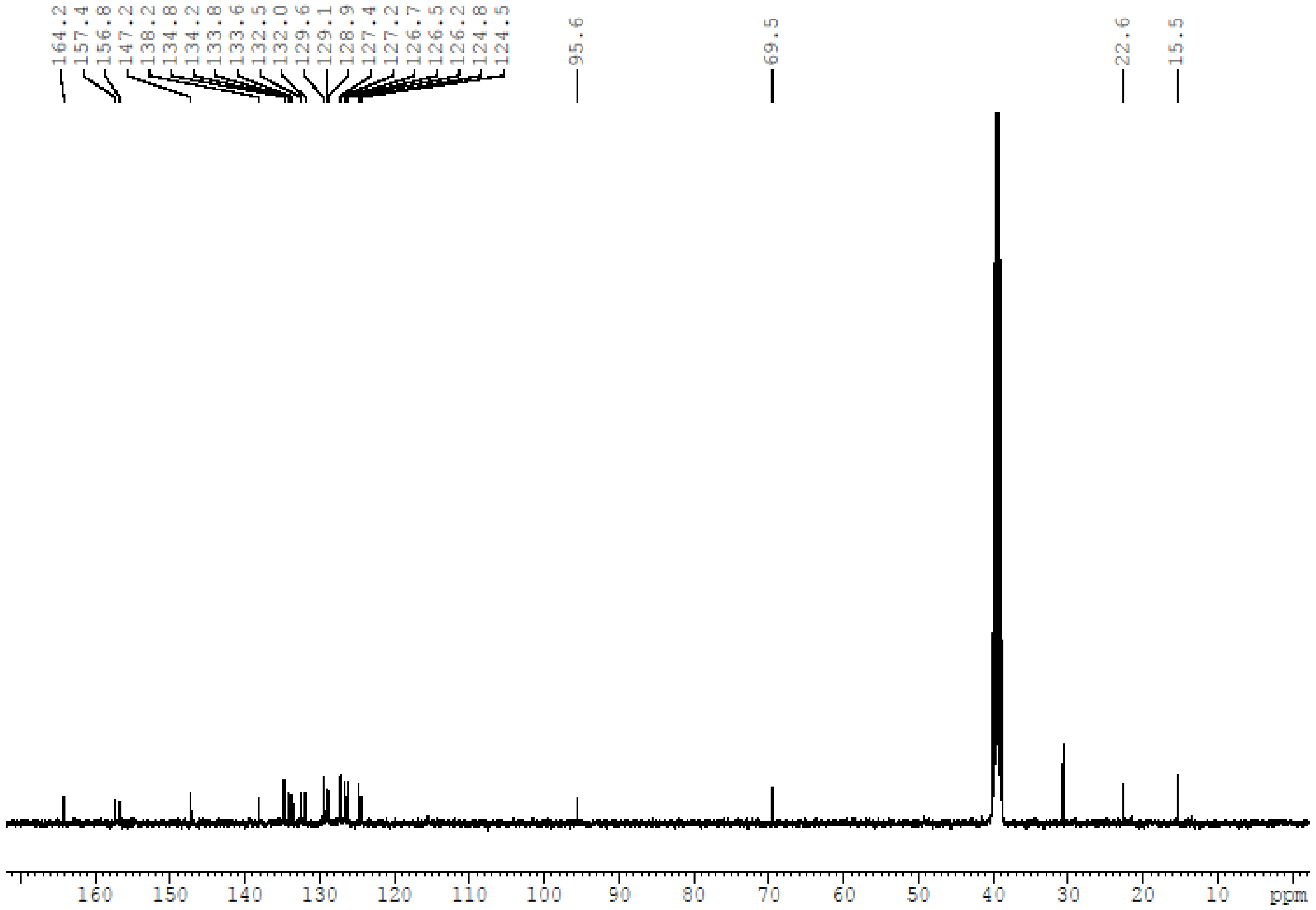

Figure S150: ^{13}C NMR spectrum of **21l** (100 MHz; DMSO-d_6).

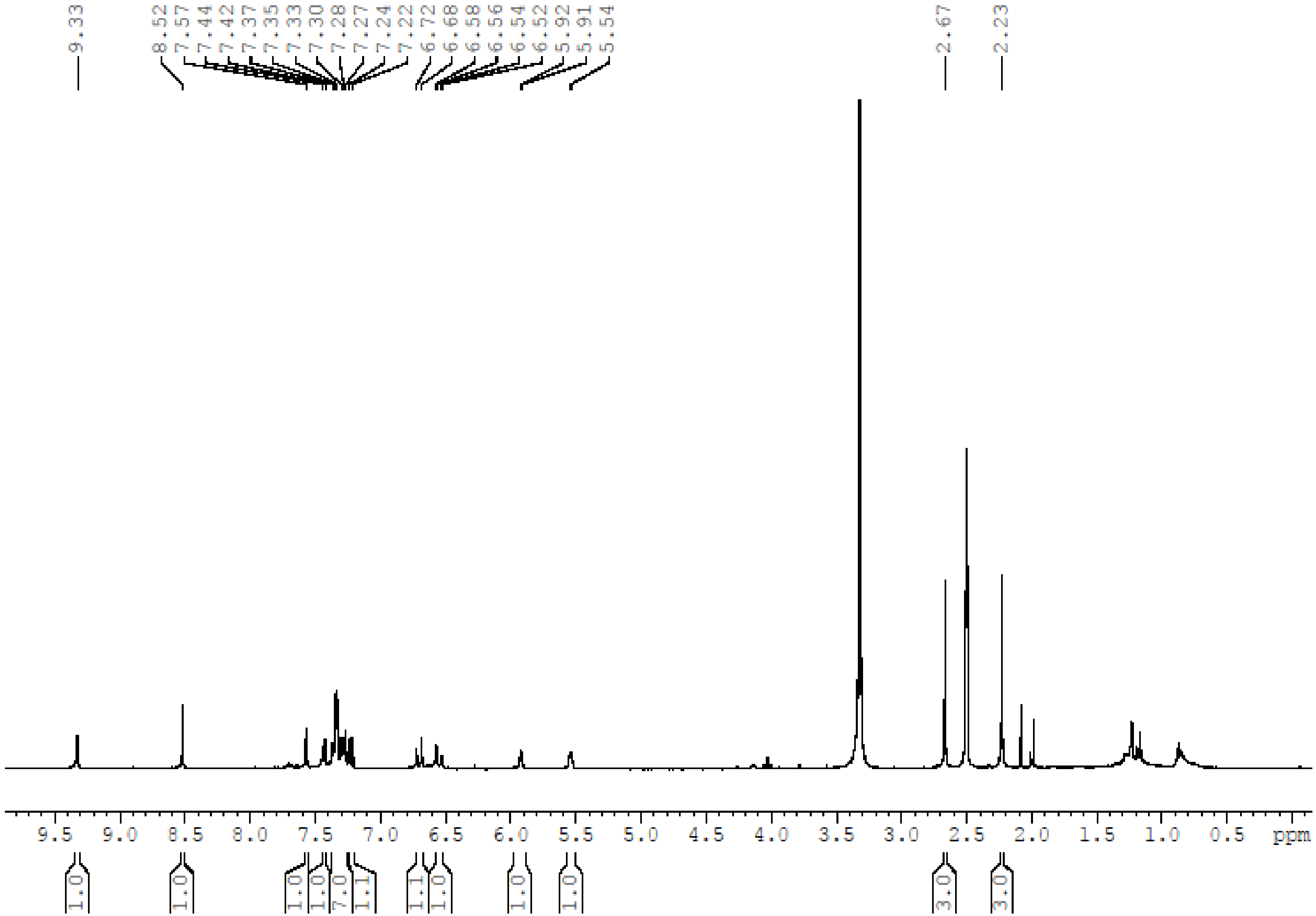

Figure S151: ^{1}H NMR spectrum of **21m** (400 MHz; DMSO-d_6).

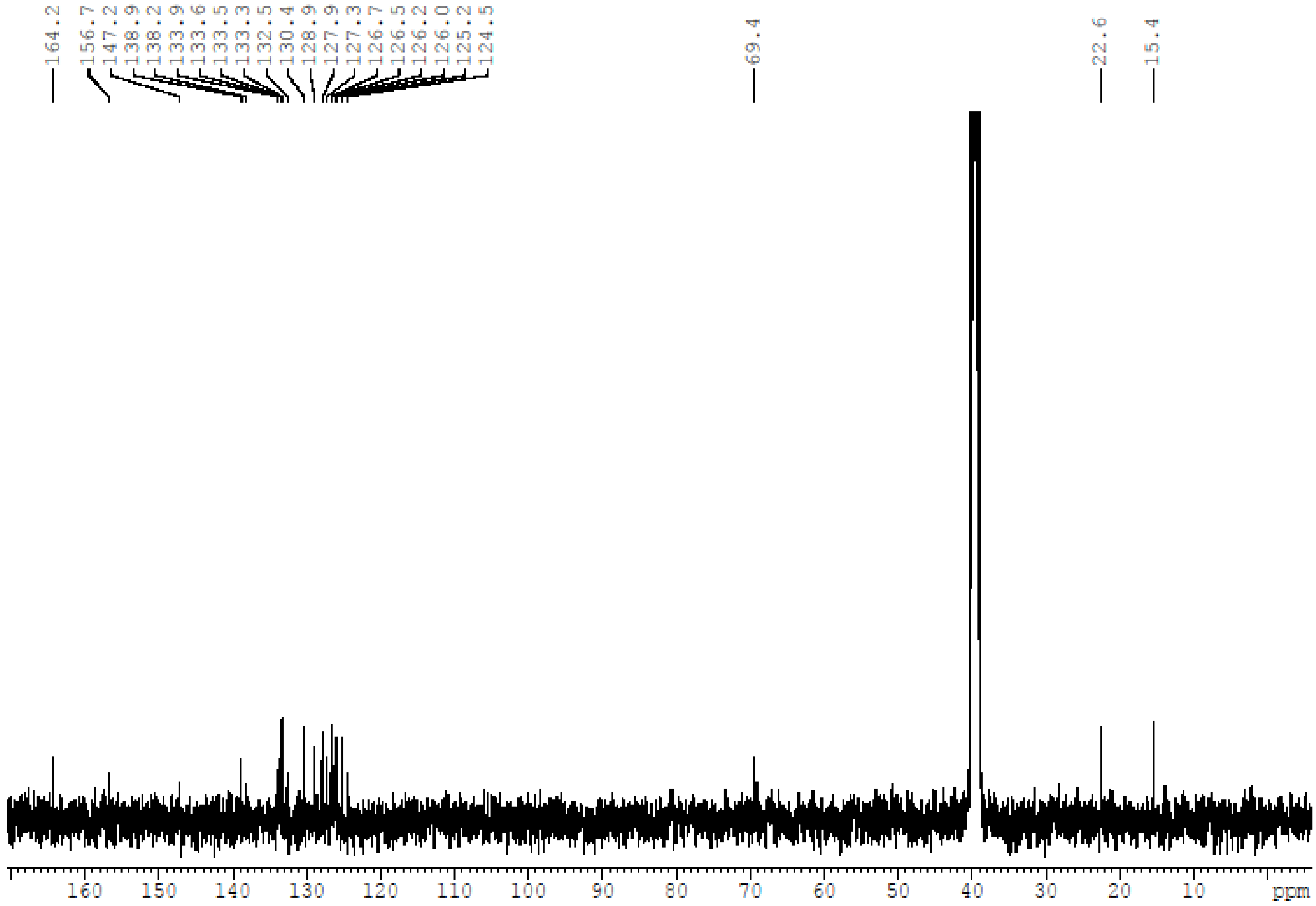

Figure S152: ^{13}C NMR spectrum of **21m** (100 MHz; DMSO-d_6).

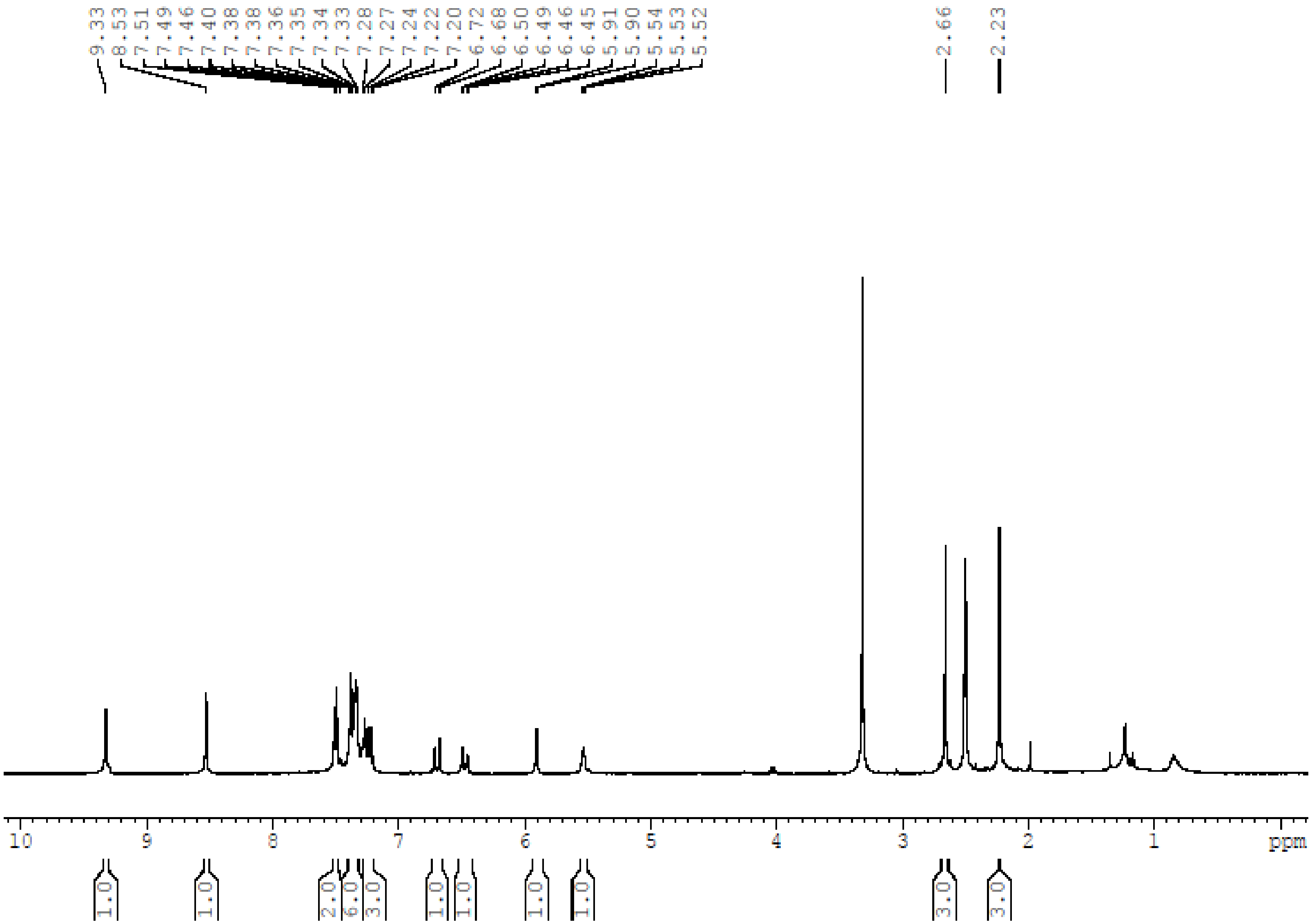

Figure S153: ^{1}H NMR spectrum of **21n** (400 MHz; DMSO-d_6).

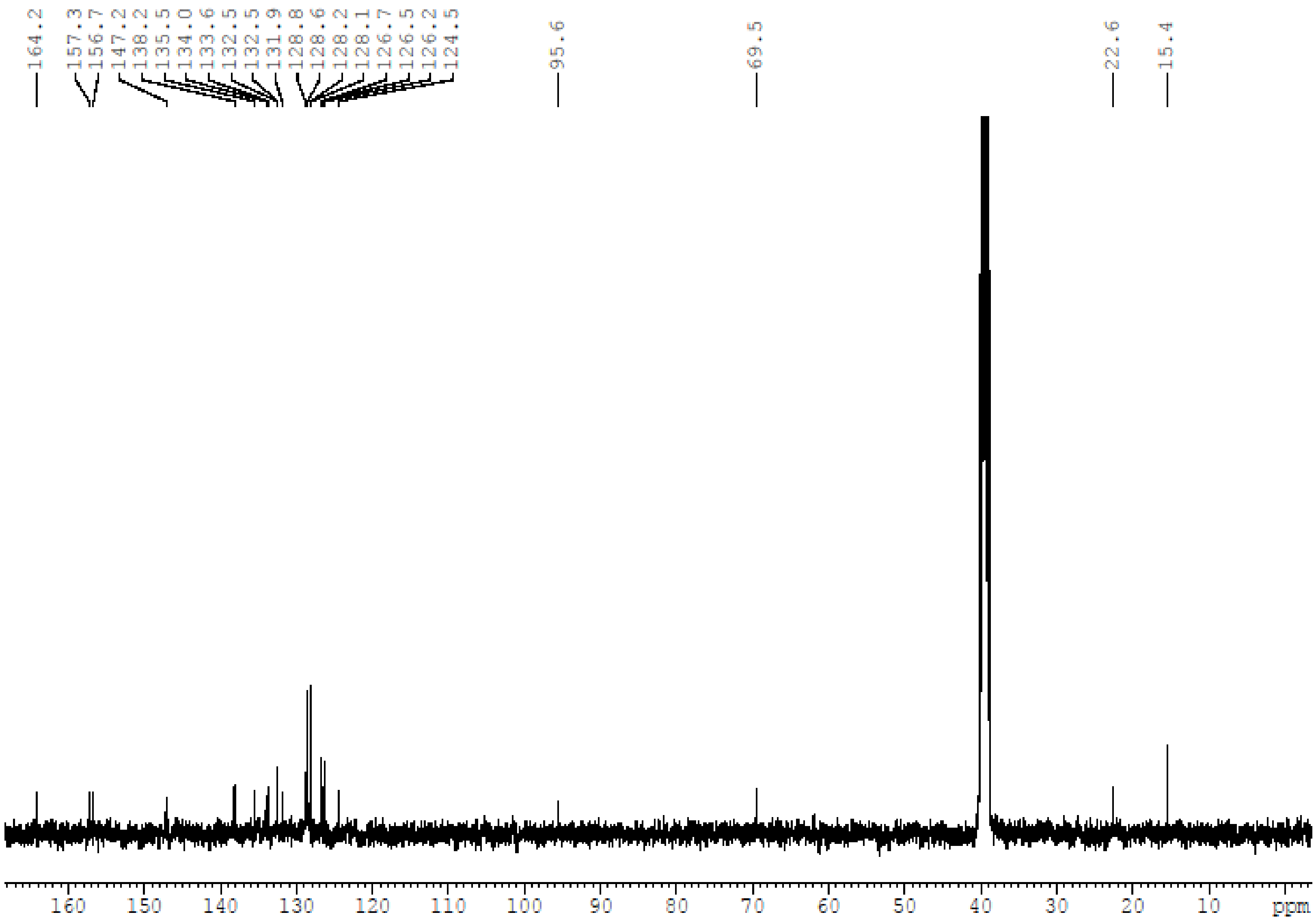

Figure S154: ^{13}C NMR spectrum of **21n** (100 MHz; DMSO-d_6).

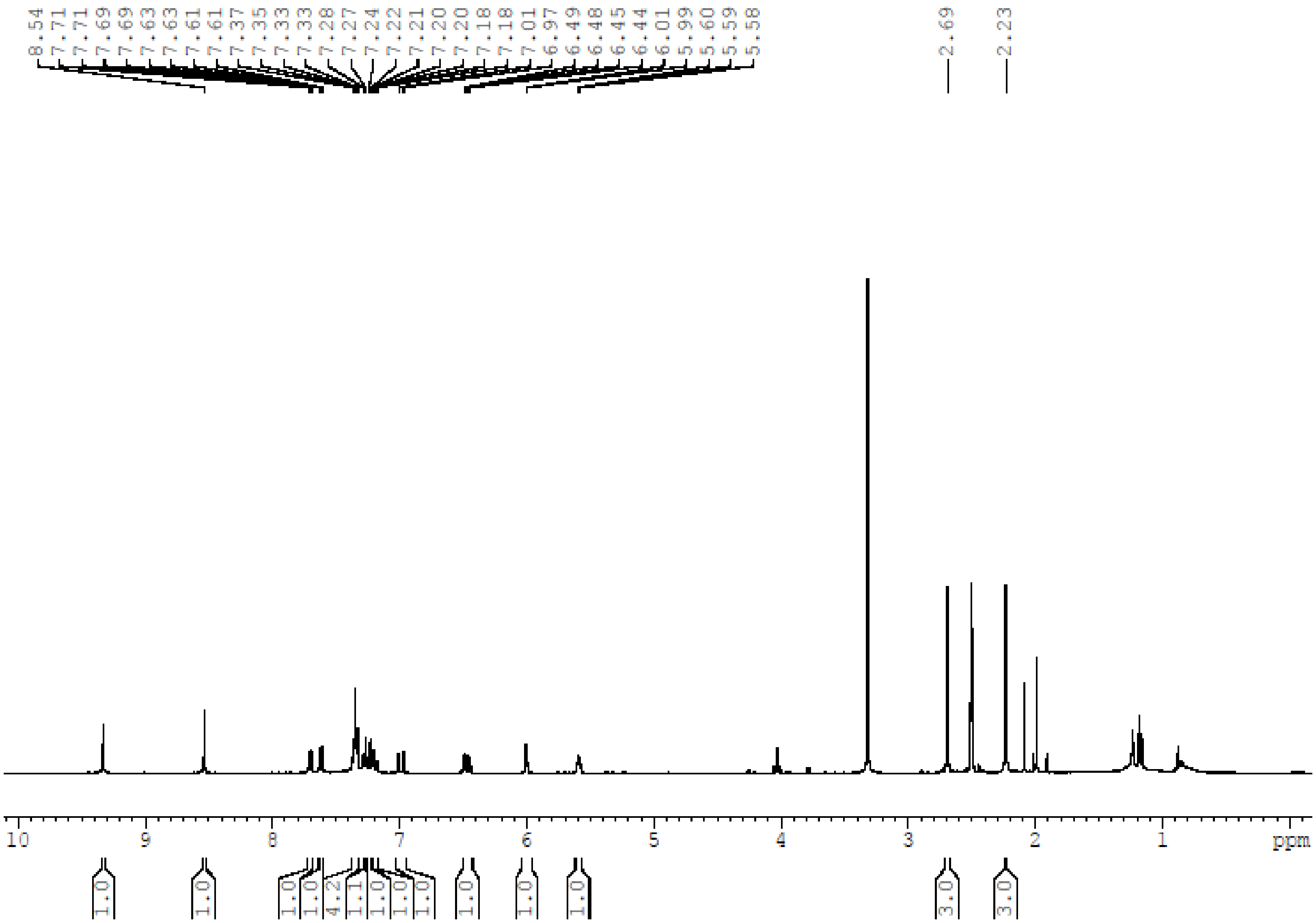

Figure S155: ^{1}H NMR spectrum of **21o** (400 MHz; DMSO-d_6).

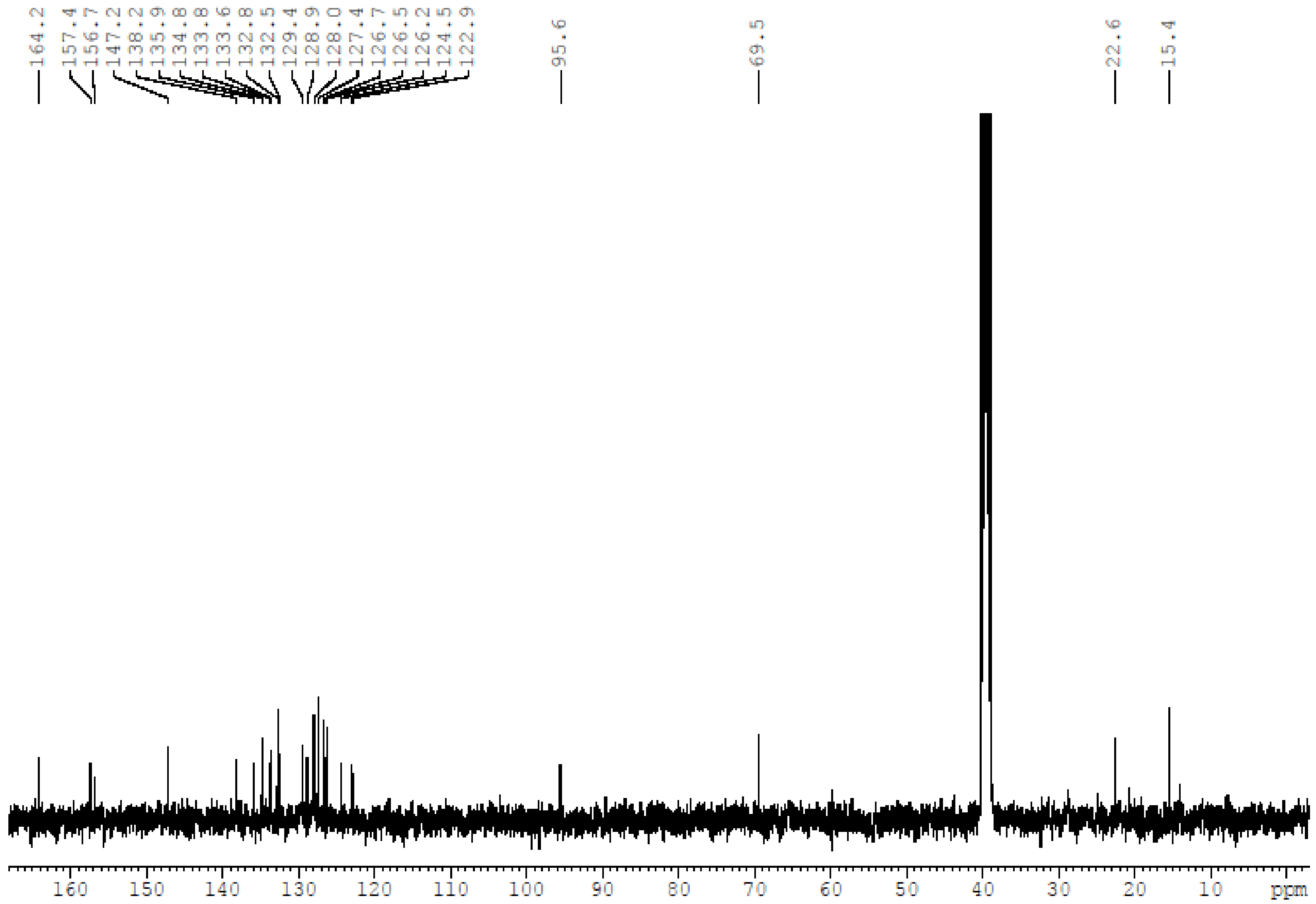

Figure S156: ^{13}C NMR spectrum of **21o** (100 MHz; DMSO-d_6).

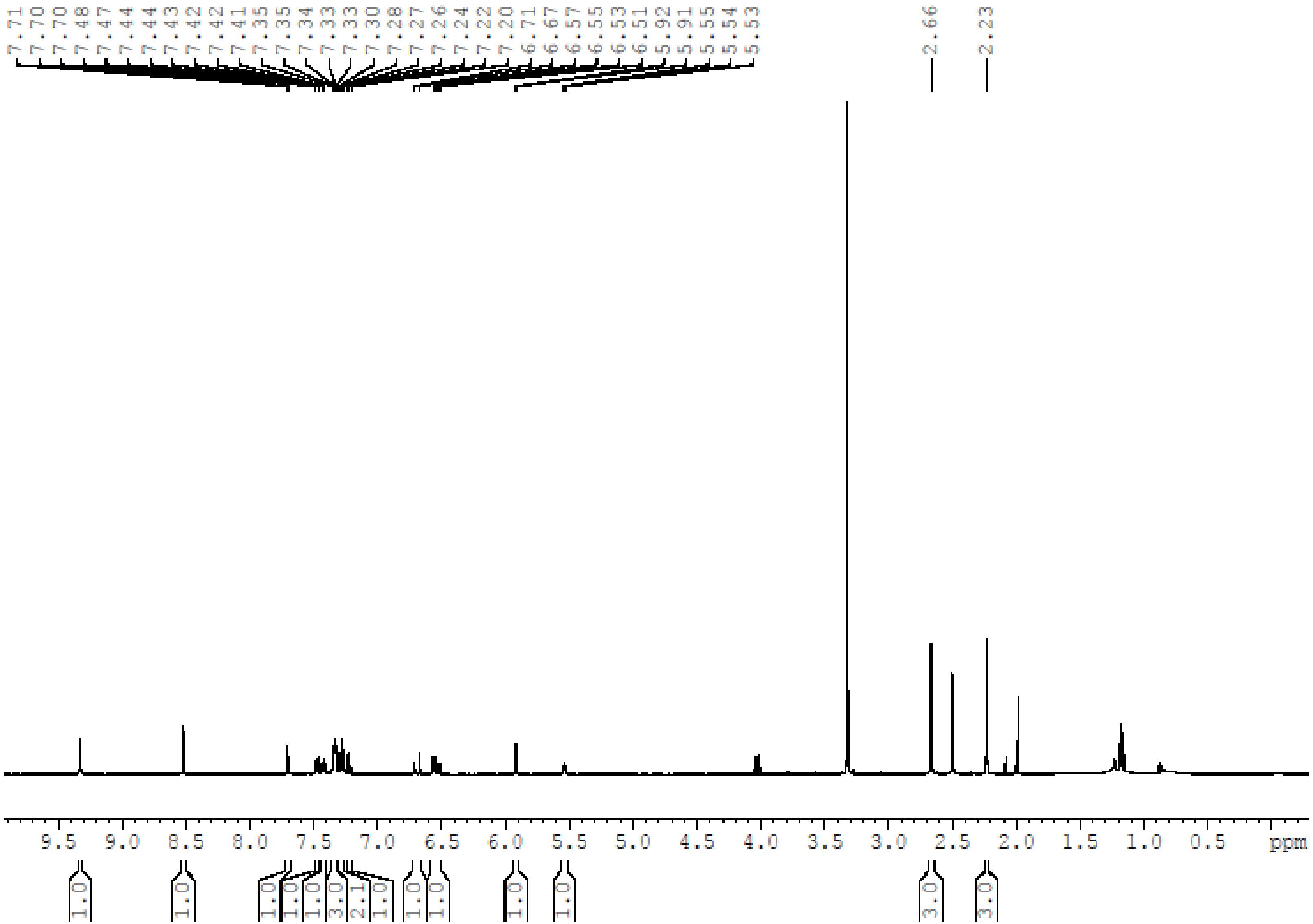

Figure S157: ^{1}H NMR spectrum of **21p** (400 MHz; DMSO-d_6).

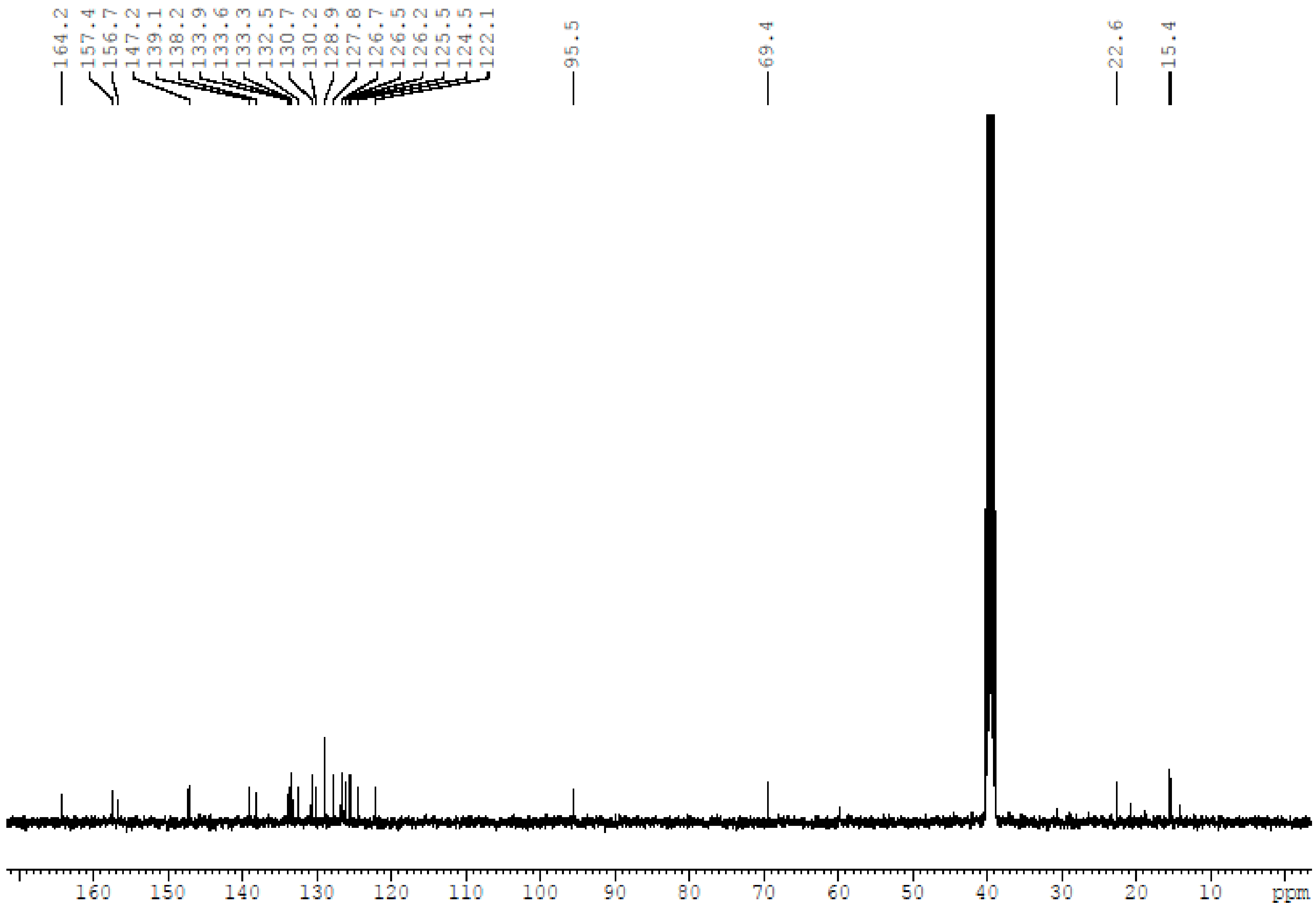

Figure S158: ^{13}C NMR spectrum of **21p** (100 MHz; DMSO-d_6).

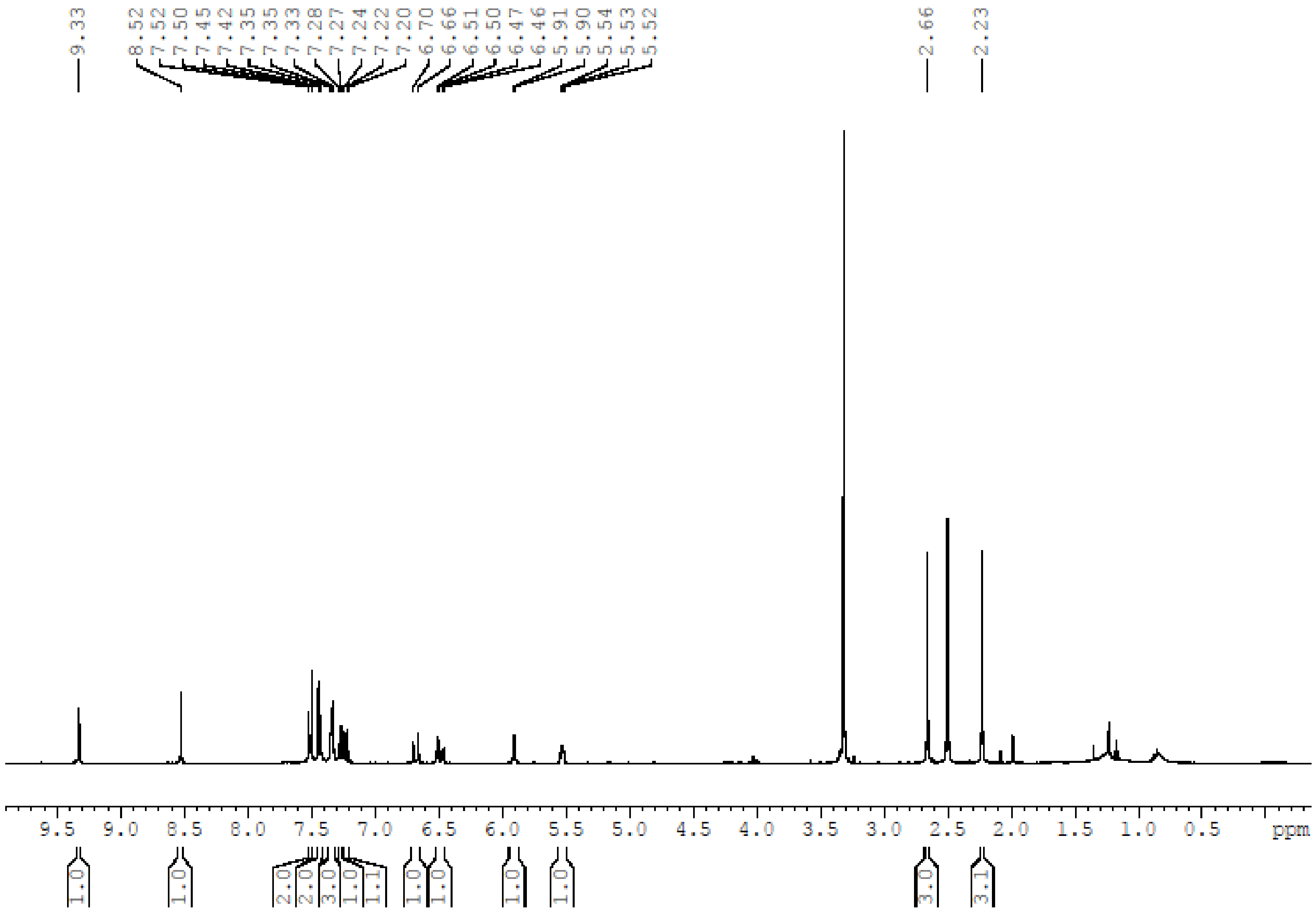

Figure S159: ^{1}H NMR spectrum of **21q** (400 MHz; DMSO-d_6).

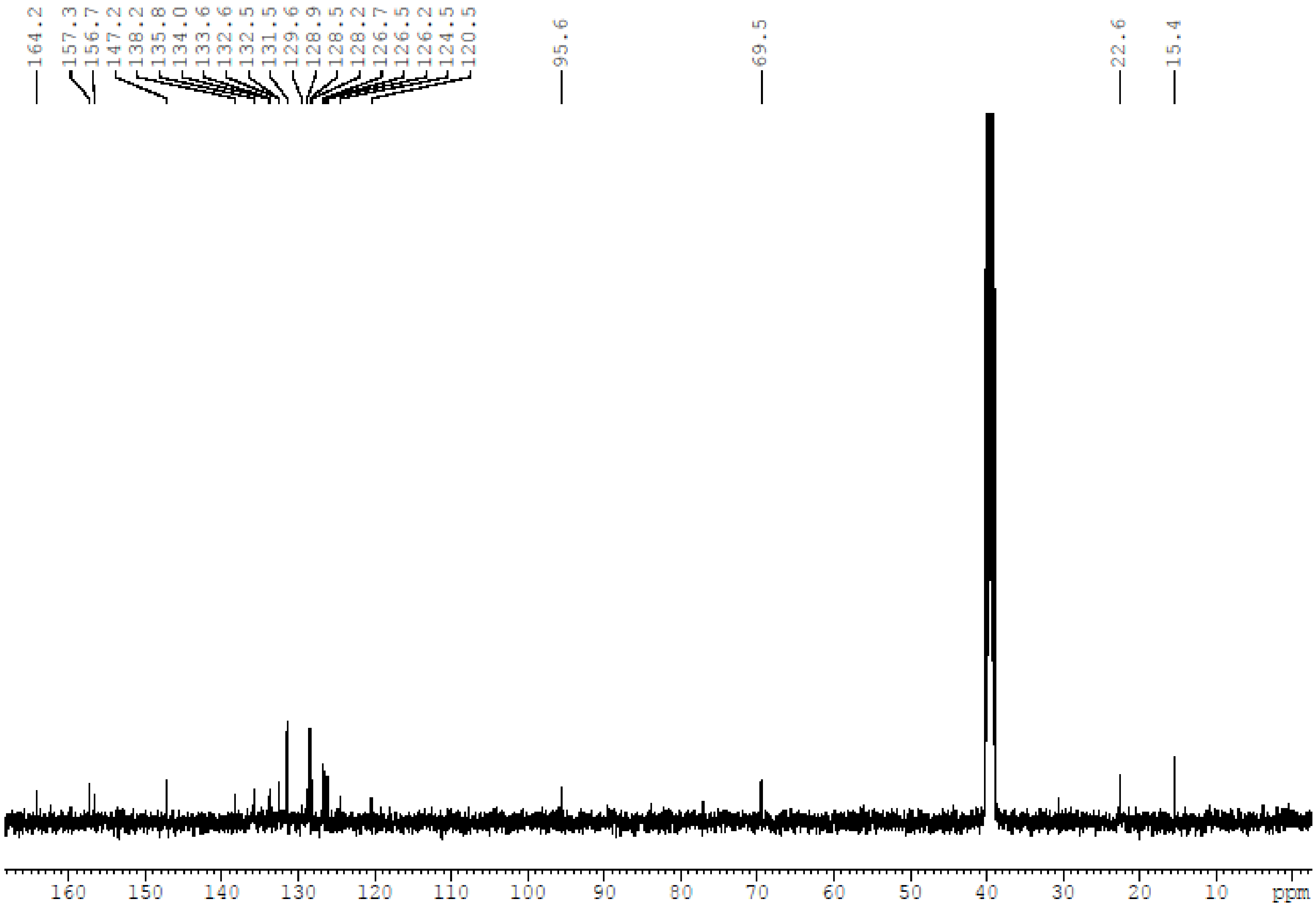

Figure S160: ^{13}C NMR spectrum of **21q** (100 MHz; DMSO-d_6).

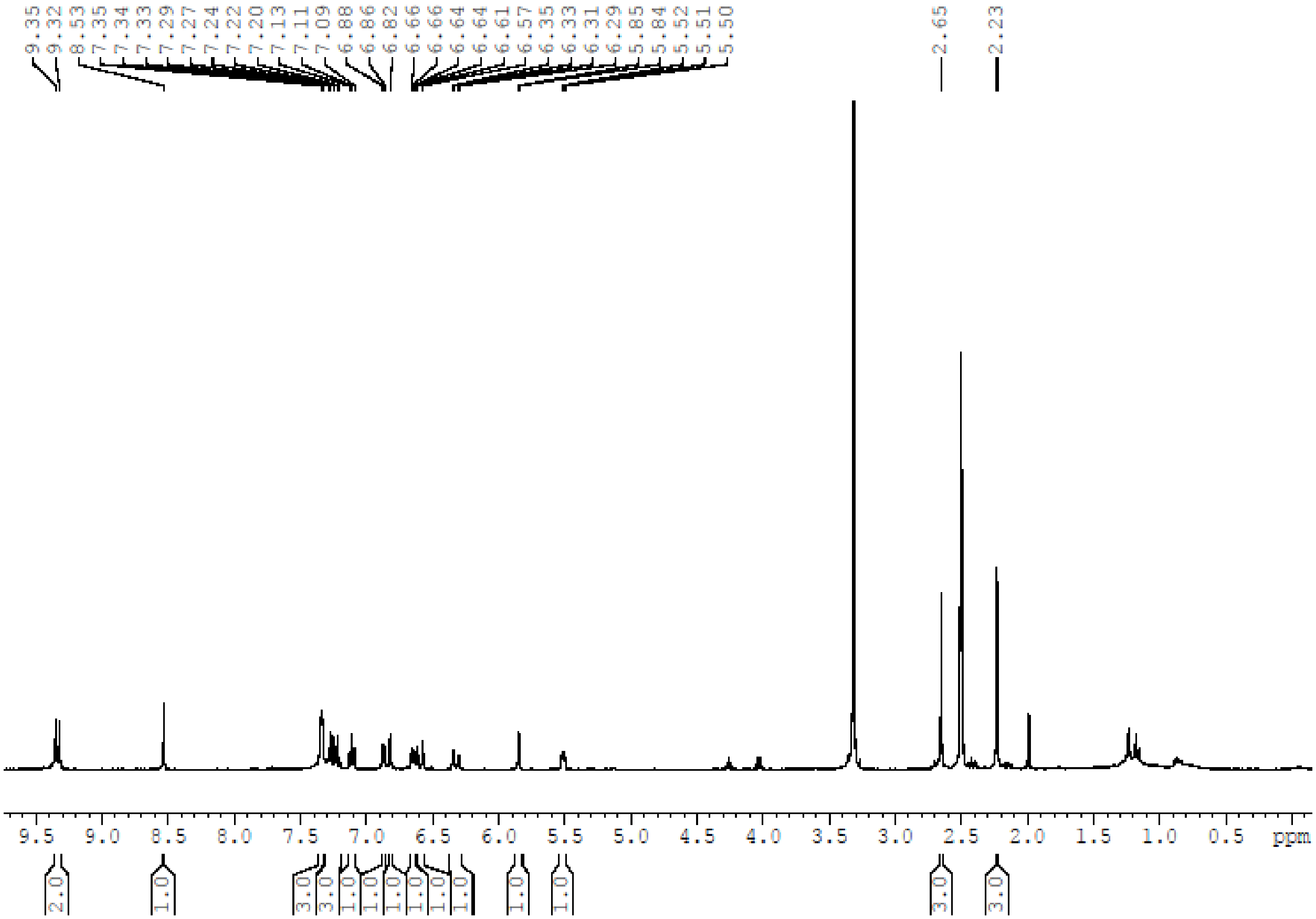

Figure S161: ^{1}H NMR spectrum of **21r** (400 MHz; DMSO-d_6).

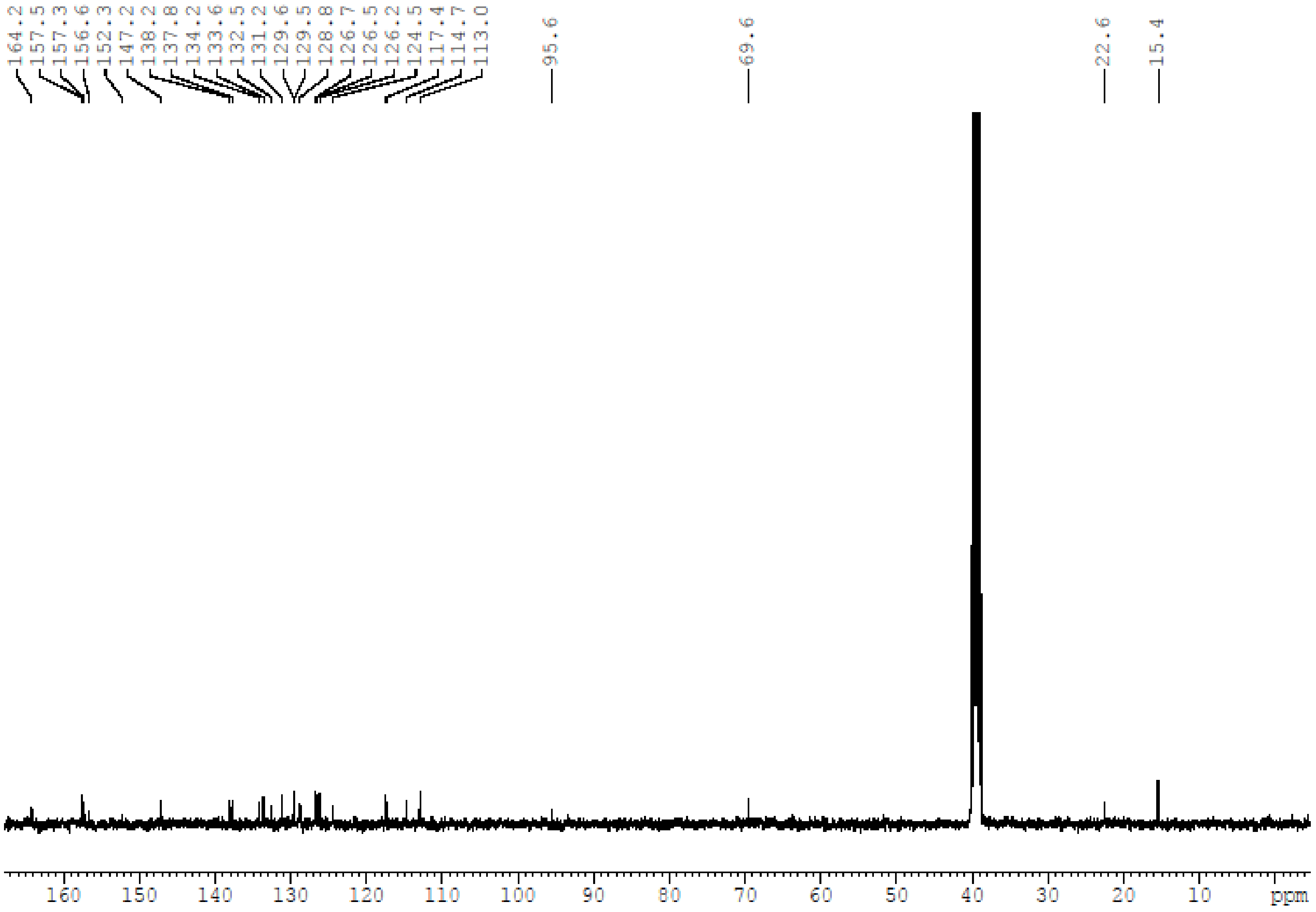

Figure S162: ^{13}C NMR spectrum of **21r** (100 MHz; DMSO-d_6).

References

1. Kim, B.-T.; O, K.-J.; Chun, J.-C.; Hwang, K.-J., Synthesis of dihydroxylated chalcone derivatives with diverse substitution patterns and their radical scavenging ability toward DPPH free radicals. *Bull. Korean Chem. Soc.* **2008,** *29* (6), 1125-1130.
2. Barbasiewicz, M.; Makosza, M., Intermolecular Reactions of Chlorohydrine Anions: Acetalization of Carbonyl Compounds under Basic Conditions. *Org. Lett.* **2006,** *8* (17), 3745-3748.
